# Report on botanical nomenclature—Vienna 2005. XVII International Botanical Congress, Vienna: Nomenclature Section, 12–16 July 2005

**DOI:** 10.3897/phytokeys.45.9138

**Published:** 2015-02-02

**Authors:** Christina Flann, John McNeill, Fred R. Barrie, Dan H. Nicolson, David L. Hawksworth, Nicholas J. Turland, Anna M. Monro

**Affiliations:** 1 Species 2000, Naturalis Biodiversity Center, Leiden, 2333 CR, The Netherlands; 2 Royal Botanic Garden, Edinburgh, 20A Inverleith Row, Edinburgh EH3 5LR, Scotland, UK; and Royal Ontario Museum, Toronto; 3 Missouri Botanical Garden, P.O. Box 299, St. Louis, Missouri 63166-0299, USA (address for correspondence: Botany Department, The Field Museum of Natural History, 1400 S. Lake Shore Drive, Chicago, Illinois 60605, USA); 4 US National Herbarium, National Museum of Natural History, Smithsonian Institution, Washington, DC, USA; 5 Departamento de Biologia Vegetal II, Facultad de Farmacia, Universidad Complutense de Madrid, Plaza Ramon y Cajal, Madrid 28040, Spain; Department of Life Sciences, The Natural History Museum, Cromwell Road, London SW7 5BD, UK; and Mycology Section, Royal Botanic Gardens, Kew, Richmond, Surrey TW9 3DS, UK; 6 Botanischer Garten und Botanisches Museum Berlin-Dahlem, Konigin-Luise-Str. 6-8, 14195 Berlin, Germany; 7 Centre for Australian National Biodiversity Research, GPO Box 1600, Canberra ACT 2601, Australia

## Preface

This is the official Report on the deliberations and decisions of the ten sessions of the Nomenclature Section of the XVII International Botanical Congress held in Vienna, Austria, from 12–16 July 2005. The meetings of the Section took place on these five consecutive days prior to the Congress proper. The Section meetings were hosted by the Institute of Botany, University of Vienna, Austria. Technical facilities included full electronic recording of all discussion spoken into the microphones. Text of all proposals to amend the *Code* was displayed on one screen allowing suggested amendments to be updated as appropriate. The team at the University of Vienna (Christopher Dixon, Jeong-Mi Park, Ovidiu Paun, Carolin A. Redernig and Dieter Reich) ensured that the proceedings ran smoothly and enjoyably for all.

A report of the decisions of the Section was published soon after the Congress (McNeill & al. in Taxon 54: 1057–1064. 2005). It includes a tabulation of the preliminary mail vote on the published proposals, specifying how the Section acted on each and detailing amendments and new proposals approved upon motions from the floor. It also includes the report of the Nominating Committee as well as the Congress resolution ratifying the Section’s decisions, neither reproduced here. The main result of the Section’s deliberations is the *Vienna Code*, which was published as *Regnum Vegetabile* 146, on 20 Sep 2006 (McNeill & al. in Regnum Veg. 146. 2006). It was also published online, on the same date (see http://www.iapt-taxon.org/nomen/main.php).

The present report of the proceedings of the Vienna Nomenclature Section conveys, we believe, a true and lively picture of the event. It is primarily based on the MP3 electronic recordings, with, where necessary, supplementation by the comment slips submitted by most speakers and by reference to parallel tape-recording, particularly where there were gaps in the MP3 record. With these sources combined, and with all motions and voting results double-checked through the soundtrack and published preliminary report of the Section meeting based on two parallel series of notes by the Rapporteur and the Recorder, we are confident that the record published hereunder is accurate and complete as possible. The delayed production of the report has, however, meant that it has not been possible to include the text of some of the proposals made from the floor, particularly those that were unsuccessful, as no permanent electronic record was made and it was not possible to locate written records for some of these.

Before it was cast into its present, final form, this Report went through a succession of phases. The Vienna Section was, as already noted, recorded electronically. One day of each recording was then transcribed by Fred Barrie (Wednesday), Dan Nicolson (Thursday), Nicholas Turland (Friday), and David Hawksworth (Saturday). For the remaining day, Tuesday 12 July, part of the first session was transcribed by John McNeill but the remainder was professionally transcribed by Pacific Transcription, Queensland, Australia and cross-checked and edited by Anna Monro. Apart from some initial editing of the *Acacia* debate and other small portions of text by John McNeill, the entire work of converting the partially edited version of the transcript to report format was accomplished by Christina Flann. At that time some portions were rearranged to ensure that the Report reflects the sequence of relevant provisions in the *Code* even when the order of the debates differed. Deviations from the chronology of events are indicated in the text by italicized bracketed notes. John McNeill then undertook the completion of some missing portions from the tape-recordings and from other sources, but, otherwise, these first two authors took an equal share in proof-reading the final version of the text.

As in the case of previous nomenclature reports, which the present one faithfully follows in style and general layout, the spoken comments had to be condensed and partly reworded, rarely rather drastically. For this reason, indirect speech has been used consistently. Additions by the authors of this Report are placed between square brackets; they include explanatory or rectifying notes, records of reactions of the audience (to illustrate the sessions’ emotional background) and reports on procedural actions, unless they form a paragraph of their own. As in previous reports, the index to speakers has been integrated with the list of registered Section members.

The Section in Vienna attracted 198 registered members carrying 402 institutional votes in addition to their personal votes, making a total of 600 possible votes (detailed by McNeill & al. in Taxon 54: 1057, Table 1. 2005). There were seven card votes, including one pertaining to the controversial *Acacia* issue (see below). The Vienna Congress was fairly conservative in nomenclatural matters in comparison with some earlier Congresses. Relatively few changes were accepted, but a small number of significant ones and many useful clarifications and improvements were adopted. Perhaps the most important decision regarded the publication status of theses submitted for a higher degree. The Congress took the unusual step of accepting a retroactive change in the *Code* by deciding that no independent non-serial publication stated to be a thesis submitted for a higher degree on or after 1 January 1953 would be considered an effectively published work without a statement to that effect or other internal evidence. Several proposals on criteria for valid publication of names were considered and clarifications were accepted. Article 33 on new combinations was also further clarified. Three important sets of changes were accepted applying to names of fossil plants, pleomorphic fungi and fungi that had previously been named under the ICZN. Further details and other changes are outlined in the Preface to the *Vienna Code* itself.

The inclusion for the first time of a Glossary is a notable achievement of the *Vienna Code.* It is very closely linked to the wording of the *Code* and only nomenclatural terms defined in the *Code* can be included. Paul C. Silva initiated the project, prepared the first draft for consideration by the Editorial Committee and worked over several subsequent ones, ensuring precision and consistency.

It is worth noting that, despite the preceding series of controversial articles relating to the recommendation by the Committee for Spermatophyta that the name *Acacia* be conserved with a type from Australia, the debate on the issue was very positive with an opportunity for 15 speakers, representing both sides of the argument, to express their opinions.

Thanks for that are due to Dan Nicolson as President of the Section, who with the other members of the Bureau of Nomenclature, made it all run smoothly. We also thank Pensoft Publishing for agreeing to publish this Report as an issue of *PhytoKeys* and to sponsor its open access. Our thanks also go to the International Association for Plant Taxonomy for contributing to the costs of producing this Report.

Christina Flann & John McNeill

**Note:** The figures given in parenthesis for each proposal in this Report correspond to the result of the preliminary mail vote (Yes: No: Editorial Committee: Special Committee).

**Figure:**
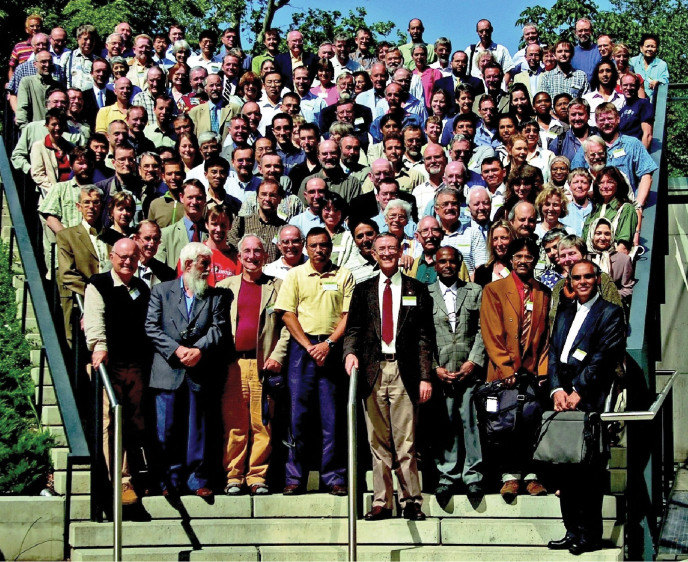
Nomenclature Section of the XVII International Botanical Congress, Vienna, Austria, July 2005. – Photograph by Rudolf Hromniak. Previously published including a key to the persons depicted in Taxon 59(4) 2010 1306–1307.

## Seventeenth International Botanical Congress Vienna 2005

### Nomenclature section

**Bureau of Nomenclature**

*President*: D. H. Nicolson

*Vice-Presidents*: B. Briggs, R. K. Brummitt, H. M. Burdet, W. Gams, P. Silva

*Rapporteur-general*: J. McNeill

*Vice-Rapporteur*: N. J. Turland

*Recorder*: T. F. Stuessy

## First Session

Tuesday, 12 July 2005, 9:00–12:30

**Stuessy** opened the proceedings with the following welcoming remarks: “It is good to see you here. Vienna is a wonderful city. I, Tod Stuessy, have been living here with my family for about eight years and it is great, but that’s no excuse for going to the city and not coming to the Nomenclature sessions. So we expect you to be here and do your homework. In any event, this is in a way a special meeting, this XVII International Botanical Congress is actually a centennial coming exactly 100 years after the second botanical congress in 1905. In fact in our archives we have photographs of the Emperor Franz Josef visiting the exhibition halls with Richard von Wettstein, a famous plant systematist who lived and worked in Vienna. So this meeting has a special sense historically and, of course, there is also a lot of modern plant science that will be going on next week in the main conference centre. The Nomenclature sessions are, of course, important; we are in effect beginning, or kicking off, the IBC today and in this era of pressures toward plant diversity, it is not only important to do the proper inventorying, but to have a proper means of communicating about the inventory that we are developing. Without naming we can hardly be efficient in our communications and hardly provide the proper response to the biodiversity and its pressures. Therefore, there is really nothing more important for us in these discussions than to provide a consistent and stable nomenclature. You know that and that is why you are here. Perhaps the discussions this year will be a little less contentious than in St. Louis. There are issues, of course, as you well know; there are interesting issues such as electronic publication which is getting more and more current and important for us to decide upon; there is the perennial English as a describing language for new taxa issue that gets closer and closer to passing; will it go over this time? And there is the minor aspect of a certain leguminous genus that also has to be resolved. In a sense totally trivial in a biological context but exceedingly contentious, In any event, perhaps it will go more smoothly than in St Louis. and certainly we look forward to very stimulating and productive sessions. We are glad that you are here and if there is anything that we can do to make your stay a little better please let us know and we will try to help out, so on behalf of the Organizing Committee of the IBC a warm welcome and I hope you have a very productive nomenclature session. Thank you.”

**Nicolson**, chairing the session, wanted to reinforce what Tod Stuessy had just said with regard to the 1905 Congress. It was Briquet, the first Rapporteur-general, who had made that Congress really work. With respect to nomenclature, that was truly the first international congress and the occasion of the first international agreements on nomenclature. He hoped this meeting would do as well. Prior to getting the meeting moving, he said he would do what he had done at previous Congresses and that was to bring to the attention of the Section those past members who had died. It was a rather substantial list but he asked the Section to think of some of these people. He then read the names of those taxonomists who had died since the 1999 Congress or whose names had been overlooked in previous listings (Taxon 48: 785–788. 1999; Taxon 42: 929–930. 1993; and Englera 9: 10–11. 1989). The full list appears as Appendix A.

**Nicolson** concluded by expressing his appreciation of the members remembering those folks many of whom contributed a lot to nomenclature. He then asked the Rapporteur-general to introduce some of the jobs that the Section had to do.

**McNeill** welcomed the members of the Section and asked the President to introduce those at the front table and explain how they had come to be appointed as the Bureau of Nomenclature

**Nicolson** introduced Nick Turland, Missouri Botanical Garden, St Louis, the Vice-rapporteur, John McNeill, Edinburgh, the Rapporteur-general, Tod Stuessy, Vienna, the Recorder, from whom the Section had already heard, and he himself from the Smithsonian Institution in Washington.

**McNeill** then noted that the Bureau did not appoint itself, but was appointed as provided in Division III.3 of the *Code* – the Rapporteur-general by the St. Louis Congress, and the others by the Organizing Committee for this Congress, the Vice-rapporteur being appointed on the nomination of the Rapporteur-general.

He went on to say that the Bureau was proposing to the Section a number of appointments that needed to be made. The first was that of Vice-Presidents of the Section. Vice-Presidents might be called upon to assist the President should he so wish, but the appointments also recognized the individuals’ contributions to and expertise in botanical nomenclature. The Bureau proposed the following five: Barbara Briggs (Sydney, Australia); Richard Brummitt (Kew, UK); Herve Burdet (Geneve, Switzerland); Walter Gams (Utrecht, Netherlands); Paul Silva (Berkeley, USA). The Section approved the appointments with loud applause.

The Section also needed to appoint a Nominating Committee to ensure that the various positions required to ensure continuance of nomenclature activity for the next six years were filled appropriately. These included the position of Rapporteur-general for the next Congress, the appointment of the Editorial Committee for the *Code* arising from this Congress and ensuring that the membership of each of the other Permanent Committees described in Div. III.2 was well-balanced. He noted that the Secretaries of the Committees generally provided names of suitable Committee members, but the Nominating Committee’s role was to ensure that the suggested composition of these Committees met the needs of botanical nomenclature. The Bureau recommended the following as the members of the Nominating Committee that was as representative as possible both by geography and discipline: Bill Chaloner, Chair (Egham, UK), Bill Buck (New York, USA), Gerrit Davidse (St. Louis, USA), Karol Marhold (Bratislava, Slovenia), Jefferson Prado (Sao Paulo, Brazil), A. K. S. A. Prasad (Tallahassee, USA), Scott A. Redhead (Ottawa, Canada), Judy West (Canberra, Australia), and Guanghua Zhu (St. Louis, USA). He asked if the Section agreed that these persons form the Nominating Committee; the Section agreed with loud applause.

The next matter to be considered was the Preliminary Mail Vote; members had received a copy of the results of this in their package. According to the *Code* (Div. III.4) this is a guiding vote. There was one way in which this vote was particularly guiding. It had been customary for very many Congresses that any proposal receiving more than 75% “No” votes was not considered further by the Section but ruled as rejected, unless specifically requested by a number of members of the Section. Accordingly he moved that all proposals receiving more than 75% “No” votes be considered to be rejected without further action by the Section, unless discussion is specifically requested. **The motion was accepted.**

To ensure that discussion of a proposal heavily rejected in the mail vote was indeed the mind of the Section it had been agreed at recent Congresses that the number supporting such a request be set at 5. He therefore moved that to be accepted by this Section, such a request for discussion required, not the usual proposer and seconder, but must be supported by a total of five persons, otherwise the proposal was ruled as rejected. **The motion was accepted.**

He then checked with **Stuessy**, the Recorder, if there were any matters relating to the Preliminary Mail Vote that required clarification or correction. There were none; all was in order.

**Demoulin** thought that as the February *Taxon* was only received in May it had been difficult to complete a good and timely mail vote and so it would be more appropriate that only the normal proposer and seconder be required for discussion of a proposal defeated by more than 75% in the preliminary mail vote.

Despite the previous acceptance of the proposal, **Nicolson** asked Demoulin if he was making a formal proposal; **Demoulin** said he was

**Nicolson** asked if there was a seconder to Demoulin’s proposal; there was one. As President he wanted to emphasise that the members of the Section try to understand what they were voting on and whether it had been ruled as having passed or failed. He then asked for a vote on Demoulin’s motion. On a show of hands, the motion was overwhelmingly defeated.

**Stuessy** emphasised that speakers must use the microphones otherwise their comments would not be recorded and included in the Proceedings of the Section.

**McNeill** wanted to talk briefly about the procedures that the Section followed and to invite the support of the Section for certain procedural matters that Nomenclature Sections generally followed but were not enshrined in the *Code*. He said that at any Congress there were a number of people present who had not previously been at a Nomenclature Section meeting. This was why he would like to take a little time to explain how the meeting would proceed. It had been obvious from e-mails and discussions over the past few months that this was quite an arcane topic for quite many active botanists and nomenclaturalists. Those who had been to quite a number of Nomenclature Section meetings could probably still remember that a number of things were not altogether clear to them at their first such meeting. For this reason the Section should take it a little slowly to begin with to ensure that more people did follow what was going on. He was sure that this would be beneficial for the security of plant nomenclature and plant names..

The main business of the Section’s meetings was to consider the 313 proposals that had been made over the past four or five years to amend the *St Louis Code*. The Section would also have to approve (or otherwise) the actions over the past six years of the Permanent Nomenclature Committees appointed in St. Louis mostly on conservation and rejection of names but also on whether names are sufficiently alike to be confused etc. And it would need to appoint a Rapporteur-general for the XVIII IBC in 2011, an Editorial Committee to produce the *Code* incorporating such changes as were made during these sessions (which, fortunately, could be called the “*Vienna Code*” as its predecessor of 100 years ago is entitled the “*Vienna Rules*”) and the membership of the other Permanent Committees that would operate over the next six years. To come up with a suitable slate of persons for these tasks was the role of the Nominating Committee that had just been appointed.

He was sure that eventually all of the members of the Section would have some issues of concern upon which they had a burning need to speak and that indeed was to be encouraged – all members should have the opportunity to express relevant and important concerns on matters that came before the Section. But for this to be done in the time available – and the Recorder had assured him that having late night sessions would be enormously expensive so that must be avoided – people would have to be concise. Moreover, as the Recorder has already emphasised, speakers should wait for the microphone before speaking. McNeill said it was already clear that many people would not be audible without the microphone but its use also ensured that the members’ words of wisdom were not lost but recorded for posterity in the proceedings of the Vienna Nomenclature Section. To facilitate this, speakers would also be given a numbered sheet of paper on which they were asked to write down, even more concisely, what they had just said – or what they had intended to say (not always identical, but in case of conflict the proceedings recorded what was actually said). He asked members who rose to speak to introduce themselves by name and city or institute so that all would know who was speaking

The Rapporteur continued that the procedures followed were basically parliamentary procedures; that is motions were proposed and seconded and there might be amendments to them and so forth. Published proposals (so long as they did not receive more than 75% No votes in the mail vote) were considered to have already been proposed and seconded, so these were on the floor for discussion, but, during consideration of a published proposal, a member might wish to propose an amendment to that proposal or even a quite separate proposal such as referring the whole matter to a Special Committee – such amendments or motions needed to be seconded and once that was done they were discussed in the usual way and amendments to them might be proposed and seconded. Any amendment would then be discussed and, if accepted, the motion as amended would be subject to further discussion and vote.

Decisions were taken by vote, normally by a show of hands. The result was normally quite clear at least from the front but he recognised that this was not always quite so evident for those sitting in the rows and there was also provision for a card vote All delegates had been issued with voting cards, coloured according to the number of personal and institutional votes that the delegate carried; a white card represented 1 vote; green, 2; yellow, 3; and red 5. If the show of hands was sufficiently clear, the chair would rule that the proposal had been accepted or rejected, as the case might be. In other cases the chair might ask for a show of cards to take account of institutional votes, but in his experience this rarely resolved a doubtful result; if the show of hands (or cards) was indecisive, the chair would require a card vote. In addition members of the Section might call for a card vote if they questioned the chair’s ruling on the result of any vote. However, card votes were very time-consuming and should be avoided except where essential for a clear decision. When a card vote was called delegates would be told which of the numbered cards to use for that vote. The counting of votes would be by tellers and would involve those persons missing perhaps 20 minutes or so of discussion when a card vote was held. The Bureau was making three nominations of tellers and inviting nominations for a fourth

The following were then appointed as **Tellers:** Alina Freire-Fierro, Missouri Botanical Garden, St Louis; Elspeth Haston, Royal Botanic Garden, Edinburgh; Nadia Talent, Royal Ontario Museum, Toronto; and Duane Kolterman, Universidad de Puerto Rico, Mayaguez, the last-named proposed from the floor.

He turned then to the matter of voting. The *Code* did not specify anything on the matter of majorities, so, absent any other action, a proposal to amend the *Code* would pass with the standard 50% majority. It had, however, been the practice for a very long time for Nomenclature Sections to require a 60% majority of the votes cast for any proposal to be accepted that was doing something as important as modifying the *Code*. The Bureau believed this practice should be maintained and accordingly he proposed that in order for a proposal to amend the *Code* to be accepted it would require at least 60% of the votes cast. The proposal was **accepted** with applause. He emphasised that this was for proposals to amend the *Code*; it did not relate to procedural matters for which a simple 50% majority would apply. The Section might also decide, on the advice of the Rapporteurs, that when there were two strictly alternative ways of dealing with a particular issue, then, if there was a 60% majority for a change in the *Code*, the choice between the alternative ways of doing so might be determined by a simple (50%) majority.

The Rapporteur noted that the decisions on changes to the *Code* were made by the Section but in the thrust of debate the wording was sometimes not quite perfect, and that was why there was need for an Editorial Committee to put together the decisions and to ensure that they did reflect the will of the Section and also that the *Code* was internally consistent.. The Editorial Committee for the *St Louis Code* had done this and that *Code* had been in use for five years, but it required to be officially adopted and approved. He moved, on behalf of the Bureau, that the *St Louis Code* be given official approval as an accurate reflection of the decisions made at the St Louis Congress.

**Nicolson** thanked the Section for their **acceptance**, with applause, of the *St Louis Code*.

**McNeill** then introduced his last piece of formal business in which he looked forward to the *Vienna Code*. He said that it was important that the Section both give authority to but also put restraints upon the Editorial Committee and in consequence he moved the **motion** that had not changed for many Congresses: “that for the revised *Code* to arise out of this Congress, the Editorial Committee [to be appointed during the final session] be empowered to change, if necessary, the wording of any Article or Recommendation and to avoid duplication, to add or remove Examples, to place Articles, Recommendations, and Chapters of the *Code* in the most convenient place, but to retain the present numbering in so far as possible, and in general to make any editorial modification not affecting the meaning of the provisions concerned”. The motion was **approved** with applause.

**Dorr** noted that in the past the motion relating to the *Code* based on the decisions of the previous Congress had included acceptance of that printed *Code* as the basis for the discussions in the Section.

**McNeill** apologised for this omission and said that it should have been part of his proposal. He thanked Larry Dorr for pointing this out. The addition was accepted by the Section.

**Nicolson** again reminded members to identify themselves

**McNeill** asked if there were any questions on general procedure or on the comments made that morning. There being none, the Section took a short break prior to starting to consider proposals to amend the *Code*.

**Nicolson**, referring to his earlier report on those who had died since the last Congress, asked if anyone in the Section knew of other botanists who had died recently and had been overlooked to please let him know.

**McNeill** reminded the Section that it was customary when certain dramatic procedural matters were put to the vote that a two-thirds majority was required; the one that might possibly arise would be a proposal to discontinue discussion [on a proposal or amendment] and a two-thirds majority would be required for that. He moved on to the first series of proposals. He added that the Bureau had concluded that they would follow the general custom and follow the sequence of the *Code* in dealing with the proposals to amend, which was the sequence that appeared in the synopsis of proposals and the Rapporteurs’ comments. However, the Section would not discuss proposals that were part of a later package where the proposal. was a peripheral component. There were proposals that related, for example, to orthography that appeared quite early and discussion of these would be deferred until the sequence arrived at the main part of the proposals, because they were very much dependent on looking at the issue as a whole, and he suggested that there would probably be a general discussion on the orthography proposals when Art. 60 was reached.

### General Proposals

**Prop. A** (39: 30: *78: 12).

**McNeill** introduced the first proposal, Gen. Prop. A, by Silva which instructed the Editorial Committee to provide a glossary of terms in the *International Code of Botanical Nomenclature*. He reported the preliminary mail vote noting that the 78 for reference to the Editorial Committee had a particular meaning applied to it. He explained that this occurred from time to time when the Rapporteurs suggested that an Editorial Committee vote be the means to identify sympathy or support for aspects of the proposal but not perhaps its full implications.

In this particular case, the Rapporteurs had suggested that an ed. c. vote would indicate support for having a glossary but that the Editorial Committee be instructed to find ways of producing a glossary in a manner that would not prevent rapid publication of the *Code*, which might be that the glossary was published later and separately. He thought that the intent was that it should be an official glossary that reflected the actual wording of the *Code* and had almost the same authority as the *Code* itself.

**Eckenwalder** wondered if that authority also included the possibility that it could be published as part of the *Code* if that could be done expeditiously?

**McNeill** agreed that it most certainly could.

**Rijckevorsel** wished to raise a point about the status of the glossary and more specifically the possibility of making amendments to the glossary as if it were a part of the *Code.* He suggested that a separate booklet was a very good idea and that it should have an intermediate status and that by the next Congress, people could make amendments if they thought that it was wrong. He felt that otherwise there would be a glossary that was either good or wrong and people would have to decide on including it without the possibility of adjusting it.

**Nicolson** understood the suggestion was for a preliminary separate document rather than putting it directly in the *Code*, so that the Editorial Committee try to prepare a glossary and that that could be published separately and then it would be possible to work on it at the next Congress.

**Rijckevorsel** confirmed that was his suggestion. He felt that it was a matter of its status and the possibility of making amendments to it so that the next *Code* could go ahead at its regular pace, not hindered by a glossary published separately but that it should be possible to make amendments to the glossary as if it were a part of the *Code*.

**Nic Lughadha** was concerned about the status of the glossary. Her view was that it should have no status as part of the *Code* and that it should be an explanatory information document. Otherwise she felt there was the potential for a whole series of discrepancies, differences of interpretation and so on. She thought it could be a useful thing to have but it should not be seen as having any particular status in relation to the *Code*.

**Davidse** strongly agreed with the status comment that had just been made but he also believed that it would be much more useful, even if it took a little bit longer to finish the *Code*, to actually include it as part of the *Code* itself. He was afraid that it would get lost if published separately as had been the case with the previously published one. He thought that users of the *Code* would like to have it right there when questions of interpretation came up and he thought it was worth a little bit of time.

**Dorr** wished to follow up on the Kew comment [from Nic Lughadha] and was also very concerned that the status of the document would be destabilizing to the *Code* if it was not clear that the glossary had no status other than helping people interpret the meaning of words.

**Gandhi** agreed that the glossary should not have status, but preferred that it be published in *Taxon*, so that people could comment if there was no clear interpretation of the glossary terms.

**Basu** also supported the idea that a glossary was needed for the research worker.

**McNeill** commented that he thought that the Editorial Committee would take the comments on board. He felt that if it was anything more than just an explanation of the terms in the current index, it clearly could not have the same authority as the *Code.* He added that even if it was produced by the Editorial Committee and included in the *Code* it would clearly be an interpretive document. He felt that what happened to it and its status after the next Congress was up to that Congress to determine. His personal view, which he thought reflected what the proposer had in mind, was that it should be quite a tight glossary, linked closely to the terminology that was actually used and explained in the *Code*. If it were to become more interpretive then he felt that the concerns for authority became important, and that would be borne in mind.

**Nicolson** asked for an indication as to how many people were in favour of the glossary. [The result was quite clear that people wanted to have a glossary.] Then he felt that the question was whether the glossary should be a separate publication as opposed to included in the *Code*.

**McNeill** thought that the question was whether the Editorial Committee should be required to include the glossary in the *Code*. He suggested that alternatively, the Editorial Committee could be free to incorporate it if it could but otherwise would publish it separately if it was going to delay things.

**Nicolson** asked how many people wished to give the Editorial Committee the authority to make the decision, to publish separately or include the glossary in the *Code*. He did not think there was a majority. He then asked how many were opposed to giving the Committee the authority but decided that was a tough question. [Laughter.]

**McNeill** wished to rephrase the question to try to avoid taking a card vote and suggested that those who would require the publication of the glossary in the *Code* vote “yes”. Then he asked for those who did not require it to be in the *Code* but permitted it printed otherwise?

**Nicolson** ruled that the second option had carried.

**West** requested clarification as to what was meant by “in the *Code*” – just published in the book or having the same status?

**McNeill** was talking about it being physically in the book.

**West** suspected that then the vote might be different.

**McNeill** responded by saying “Oh”. [Laughter.] He went on that the point had been made by West that when he used the phrase, “in the *Code*”, people may have thought he meant being treated as having all the authority of the *Code*, which was definitely not his intention. He assumed that the comments had been taken aboard and the situation was simply whether the Editorial Committee was being instructed to produce the glossary as physically part of the *Code*, or was it free to try to do so but not forced to do it? To his mind that seemed to be the one question that the Section was divided on. He wondered whether people would vote “yes” if the question was: do you require that the glossary be included as part of the *Code* but without having the authority of the Articles of the *Code*?

**Funk** thought that two things had been mixed up. She felt that some people would like to see the glossary before it was officially attached in the back of the *Code*, even as an index. She suggested that one thing that had to be decided was whether eventually the Section wanted it to be published as a sort of index in the *Code* or whether they wanted to require that it immediately be done in this version. She did not have any opposition to a glossary, but did not want to see it part of the *Code* until she had seen it.

**McNeill** noted that that was a point that had not actually been expressed previously and was a concern that added another dimension.

As a member of the Editorial Committee, **Barrie** wished to say that it would probably delay the *Code* by at least a year to put the glossary in it. He suggested the other thing to consider was that publishing it first in *Taxon* would give people the opportunity to review it prior to any final decision on it and things could be modified over time that way. He felt that would seem to be a good way to go.

**Stuessy** offered that *Taxon* would be pleased to handle that, but it seemed to him that what was wanted was a glossary to help people. He noted that the black book was not viewed with great enthusiasm by the younger generation of systematists and added that if it at least had a glossary in it that made it a little easier to use and understand, that was really positive and he felt it should be done.

**Silva** had in mind a glossary of terms that occurred in the *International Code of Botanical Nomenclature*. At most there would be 20 definitions, so he saw no reason why there should be a separate publication. He gave two reasons for a glossery: one was to facilitate the users of the *Code* so that they could find out directly instead of going to the index and going back to one or more Articles. The second was to sharpen the focus of the Editorial Committee so that everything within the *Code* was in agreement. He had been on the Committee several times and they had found out that some of their definitions were not in agreement, and that was one of the main purposes of the proposal.

**Rijckevorsel** liked the idea of a separate booklet which was easier to use in combination with the *Code* rather than a publication in *Taxon*, because he felt that the *Taxon* option had the downside that any time you wanted to look in it, you had to look up the relevant issue and he thought that was not handy. He also felt that it was not absolutely necessary that the Editorial Committee would undertake this task and suggested that the topic could have a different committee if the Editorial Committee was too busy or did not want to do it or if other people were more qualified.

**Zijlstra** pointed out that the *Code* lasted until the next Congress, and the glossary should be used – for several decades, so felt it should be a separate booklet in *Regnum Vegetabile*.

**Per Magnus Jorgensen** felt that from a practical point of view if he wanted to know what a word in the book meant, he wanted to find it in that book rather than running around and finding it elsewhere. He agreed that it could be changed at the next Nomenclature Section as “nothing is sacred”. He recollected that as a former member of the Editorial Committee, he remembered cases where the Committee had to sit down and say, “What do we actually mean here?” and we had to say, “Well, we don’t know” and had to find a way of getting around it. The Editorial Committee noted the way they had found to explain the meaning, and he thought that was the important thing, that the Editorial Committee should explain their opinion of the text as they presented it.

**Glen** suggested that the most practical way of handling the situation would be to publish the glossary some time in the next couple of years as a separate paper in *Taxon* and then in the 2011 *Code*, thereby satisfying everybody or annoying everybody, as the case may be.

**Brummitt** thought it important to be sure to make the *Code* and the glossary two quite separate things with no confusion between them. He was in favour of the glossary but felt that it may be controversial. He wondered if there would be proposals to amend the glossary at the next Congress? He pointed out that there was a very good precedent for publishing a glossary, 30 years ago or so, as a part of *Regnum Vegetabile* which had worked very well although it definitely needed updating. He would love to see a new glossary, but not as part of the *Code*.

**Mabberley** had thought that the Section had already made a decision on this and wished to know what the status of the proposal that was passed was.

**McNeill** asked what proposal that was.

**Mabberley** noted that there had been a proposal which he thought the President agreed that he had seen that there had been a clear majority. He wanted to know what the status of that was in view of the round and round discussions since then.

**McNeill** explained that the point was made from the floor that the wording of the proposal was misleading and so it was reworded, and as a result of the rewording the vote was no longer clear. The phrase “in the *Code*” was interpreted in a different way from that which he had intended in the first vote, so that first vote was suppressed by the second.

**Mabberley** still wished to know what the status of that proposal was in the light of that?

**Nicolson** thought it was overruled. He noted that there was a break coming up. [Laughter.]

**Stuessy** suggested that there may be a compromise possible. He had talked with Nicolson and Turland about doing a small booklet on botanical nomenclature for DNA dummies. [Laughter.] Something that tried to really explain the high points of the *Code* for people not so familiar with it and he suggested that it could have a glossary attached to it.

**Rico Arce** supported the idea that a glossary was needed. She noted that there was already one by Rogers McVaugh, which she considered closest to the *Code* and went on to suggest leaving the *Code* as it was and maybe an update of Rogers McVaugh’s nomenclatural glossary would be an easy solution until the next Congress.

**McNeill** felt that in the audience there were many different understandings of the word “glossary”. It was quite clear that some were thinking of the McVaugh model but his impression was that in the original proposal Silva was thinking of a much tighter document that was much more closely linked to every single technical term that appeared in the *Code*, and just as the *International Code of Zoological Nomenclature* had a glossary, so should the botanical *Code*, and this would not be a document that was interpretive but was simply a factual account of what was there. He also noted that, as the Recorder had just mentioned, there was a very great need for something even broader that explained the processes of nomenclature. He felt that much of the confusion as to what was really wanted related to all of those, but felt that the Section was perhaps not sure which were the more important.

**Stuessy** raised a procedural matter concerning the display of the proposals under consideration via the overhead beamer. He noted that people would want to amend the proposals and that it was possible to modify them by editing on screen in red, so that the Section could see the accepted amendments or friendly amendments. He asked that those involved in making amendments, write the change down and hand it in to avoid misunderstandings.

**McNeill** addressed Mabberley’s question about the status of the proposal by saying that his intent in making that proposal was to reflect what he thought at that point was the mind of the Section. He admitted to being wrong and had withdrawn that. What was now on the table now was the proposal by Silva which could either be accepted or rejected or it could be amended. He invited members of the Section to propose any amendments, if they so wished.

**Nicolson** offered a clarification that Silva, as the author of the original proposal, had intended something like 20 terms. He felt that they should be able to agree in the Editorial Committee that they were using the following 20 terms in whatever sense. He suggested that it would be a part of the *Code* but not an Article of the *Code*, just a tool for the Editorial Committee to be sure they were talking about exactly the same thing. He returned discussion to the original proposal and invited those that wished to amend it to write down the amendment so it could be put up on the board.

**Per Magnus Jorgensen** felt that in view of what had been said, he would add the word, “essential” technical terms which he thought better than “limited”.

**Silva** wondered what adding the word “essential” would do, reduce the number of definitions maybe from 20 down to 10 or eight?

**McNeill** asked if Jorgensen’s proposal had been seconded? [The proposal was **seconded.**] He clarified that comments should now be talking to the amendment to add the word “essential”, not to the original proposal.

**Pereira** thought that experts in nomenclature did not need the glossary. He felt that for people living and working in less developed countries and for many students a glossary was very important of the systematic botany such as that published by Frans Stafleu in 1997 and that the glossary should be published separate to the *Code*.

**McNeill** thought this a valuable comment but probably not relevant to the immediate discussion about adding the word “essential”.

**Ford-Werntz** objected to the addition of the word “essential”, because if it was there then every word that was not in the glossary was by definition non-essential. She would rather leave it to the discretion of the Editorial Committee as to what words did or did not go in and then it could be open to discussion, as Funk had pointed out. She preferred to leave the proposal unamended as originally written.

**Per Magnus Jorgensen** agreed and withdrew the amendment. [Laughter and applause.]

**Turland** commented that some concerns were raised about whether the glossary would be sort of legally binding in the *Code.* In the absence of any Article in the *Code* giving the glossary any kind of mandatory status, he clarified that it would not have that status as there would need to be a proposal to add an Article to the *Code* to make it binding and without that, it would simply be supplementary information and the technical terms in the glossary would not be mandated in any way. He thought that any concerns about that were really not necessary.

**Wieringa** suggested adding a first sentence in the glossary that it was not part of the *Code*, only published with it in the same book, so that any doubt whether it is part of the *Code* or not was immediately cleared.

**McNeill** felt that, on the contrary, it would be part of the *Code* because it would be derived from the *Code* but it would not have any mandatory authority. He felt that there was a difference between it not being part of the *Code* – of course, it was part of the *Code*, just like the index was part of the *Code* – it was derived from it, but except where it reflected the wording of an Article, it would have absolutely no standing.

**Turland** wondered if the Section should vote on the original proposal and then if anybody wanted to make an additional proposal about where the glossary should be within or without the *Code*, then that could be an additional proposal.

**Prop. A** was **accepted.**

**Prop. B** (10: 142: 4: 1), **C** (10: 142: 4: 2) and **D** (13: 138: 3: 2) were ruled as **rejected.**

**Prop. E** (28: 62: 59: 0) was referred to the **Editorial Committee.**

[*The following debate, pertaining to Gen. Prop. F took place during the Seventh Session on Friday morning with discussion on Rijckevorsel’s orthography package*. *For clarity, the sequence of the* Code *has been followed in this Report.*]

**Prop. F** (17: 95: 35: 0).

**Buck** wished to bring up Gen. Prop. F to replace the word “forming” with “coining”. As a native English speaker he found “coining” pretty objectionable. He thought of it as slangy and certainly not meaning the same as “forming”, and he did not want the Editorial Committee to suddenly put that in as an editorial thing, so he proposed that the Section vote against it and reject the proposal.

**McNeill** happened to share Buck’s view, but noted that he was only one of the Editorial Committee. He added that the voting should be for outright rejection or referral to the Editorial Committee.

**Prop. F** was **rejected.**

### Article 1

[*The following debate, pertaining to a New Proposal presented by Skog regarding Art. 1.2 and 11.7 took place during the Eighth Session on Friday afternoon*.]

**Skog’s Proposal**

**Skog** introduced a new proposal from herself and some members of the Committee for Fossil Plants and other palaeobotanists. She joked that it seemed that some of the palaeobotanists had been operating in some other universe for about five years. She suggested some may think it 150 years. But she thought that they were now on the same plane and they wished to suggest that Art. 11.7 be amended by adding the prefix “morpho” to the word “taxon” [actually “taxa”].

**McNeill** clarified that it concerned “Fossil morphotaxa”, in the first line.

**Skog** added that, Art. 1.2 would also need an additional sentence, which would read, “Any taxon that encompasses more than one part, life history stage or preservational state is not a morphotaxon.” She explained that there had been a great deal of confusion over the use of the phrase “fossil taxa” and, as she mentioned on Monday, that phrase seemed to have been a holdover from the merging of several proposals at the previous Nomenclature Sessions. The proposers hoped that adding that prefix and adding the sentence to 1.2 would clarify the situation. As it was, she explained that the *Lepidodendraceae*, which was clearly a fossil taxon, could include stems, strobili, leaves, roots, anatomy; in summary it could include multiple preservational states or parts of the life cycle.

**McNeill** asked how we knew that? He wanted to know if the specimen of the type of *Lepidodendron*, whatever species that was, had all these things in it?

**Skog** responded that in some cases they did.

**McNeill** persisted, asking if it did in this case? He continued that if it did not, then how would we know it was not a morphotaxon? His point was that his circumscription of a species, or a genus, or a family, and someone else’s, would be different. So he argued that if two types of names were being distinguished that were fossil taxa that may apply to real taxa, it was necessary to know it from the protologue of the original publication of the type of the name.

**Skog** agreed that that was correct, but did not have an example to hand quickly.

**Nicolson** pointed out that at the moment Skog was on the Editorial Committee and so there might be a chance for her to come up with the specific Example.

**McNeill** suggested “to be any taxon that is described as including” rather than “encompasses”.

**Chaloner** responded that there already was a good Example of this cited in the *Code*, in the *Sigillariaceae* (Art. 11 Ex. 25), referred to by Greuter in his notorious preface of the *St Louis Code*, and Greuter referred to the possibility of that being a natural family, meaning one that can include a number of different organs or stages, as Skog’s amendment included. He noted that it was possible to invent something as silly as a morphofamily which was based entirely on one kind of organ but he did not think any palaeobotanists wanted to do that. The charm of Skog’s proposal to him was that it allowed the concept of a family based on a morphotaxon, but the family would include a whole range of different organs, and that was the case for many important fossil families like the *Caytoniaceae*, for example, which included fruit and then seeds and leaves all believed to belong to the same family, as we would normally use the word family. He supported Skog’s amendment warmly as it recognized that fossil plant families need not be regarded as morphotaxa.

**McNeill** felt that the key proposal was the one in 1.2, and the other would follow. He added that there was also a corollary which was purely editorial; The current Note 4 in Art. 11, would become an Article again. He had some little difficulty with the full meaning of the amendment to Art. 1.2, but suggested it may be possible to improve it editorially; although he philosophized that maybe it would come back to haunt the Section at the next Congress.

**Skog’s Proposal** was **accepted.**

[*Mostly off-microphone discussion about whether the proposal on Art. 11.7 was separate from the one just passed on Art. 1.2*]

**McNeill** thought it was a single proposal and could see no reason for separating it. He concluded that it was one proposal to do the two things.

**Nicolson** suggested that the Section would vote for the second one, 11...

**Turland** felt that some of the Section understood that the vote was to add the prefix “morpho” in Art. 11.7 together with the addition to Art. 1.2 in the previous vote.

**Nicolson** ruled that the Section had voted for the two simultaneously. He had not meant to separate them if they were of same package.

**Skog’s Proposal** to alter “taxon” in Art. 11.7 to “morphotaxa” was **accepted** simultaneously with the vote on her proposal regarding Art. 1.2.

[*Here the record reverts to the actual sequence of events*.]

### Article 3

**Prop. A** (125: 29: 5: 0).

**McNeill** introduced Art. 3 Prop. A and noted that it had received a very strong positive vote in the mail ballot.

**Stuessy** thought that Gerry Moore ought to speak to the proposal because it came out of a workshop to investigate the relationship between this *Code* and the *PhyloCode* and he felt it had some broader implications.

**Moore** thought that a lot of those present were aware that there was a meeting held in Pittsburgh a few years ago and a number of people in the room were at that meeting. He reported that a number of days were spent sort of vetting the *Code* and trying to get at some of the issues that had come up informally in terms of some people feeling that the *Code* may be inconsistent with modern approaches to classification. One of the issues that had come up was some confusion about the sequence of the rank-denoting terms and when it was necessary to assign ranks and when it was not. He explained that that was what led to the proposal to make it clear that although there was a seemingly endless chain of rank-denoting terms there were limits as to what to do when proposing certain names at certain ranks and it was not necessary to classify a particular taxon in all of the ranks. The proposers did not feel that the proposal, or any of the others made as a result of that meeting, changed any of the rules of the *Code*. They felt that it was perfectly compatible with any approach of phylogenetic nomenclature as long as ranks were included. He added that this was one of the areas that was open to discussion, leading to the proposal. He thought that it basically just added some clarification to the procedures, although some sort of guide for students would even be better.

**Brummitt** had a very minor point regarding what was meant by “higher ranks” in the first sentence being explained by the second sentence and he suggested that the Editorial Committee should reverse the sequence of the two sentences, so that it could be read intelligently.

**McNeill** pointed out that a Note was something that expressed something that was inherent in the *Code* but not spelt out elsewhere.

**Prop. A** was **accepted.**

### Article 4

**Prop. A** (23: 49: *85: 1).

**McNeill** moved onto Art. 4, Prop. A and explained that the “ed.c.” vote was one of those which had a special meaning and in this case the Rapporteurs had suggested people might be in favour of the thrust of the proposal with regard to the inclusion of the word “super-” but not of removing the option of having additional terms so long as confusion was not induced. He suggested that the word, “super-” be inserted in a manner such that the option for having additional ranks was not precluded. The Rapporteurs had suggested that “While welcoming the specific recognition of “super’ as the first prefix to be used in the formation of ranks additional to the more familiar ones”, they felt that ranks should still be permitted to be intercalated or added provided that confusion or error was not thereby introduced. He noted that it was a matter that the Editorial Committee would handle in the light of approval of the addition of “super-” being the indication for the first additional rank.

**Watson** confirmed that the wording of the proposed paragraph would not change, it would just be inserted in addition to and not replacing the existing Art. 4.3 and agreed that would be an acceptable, friendly amendment.

**Buck** was concerned in a case like this, that if you wanted to insert a rank between, for example, genus and subgenus, it would be called “super-subgenus” and that seemed a relatively bizarre term to him.

**McNeill** felt it was quite clear that at the moment it was only the primary terms that “sub-” could be added to, the same would apply with “super-”. He assured him that that would be made very clear.

**Buck** pointed out that the proposal did not say that.

**McNeill** had assumed it did. He asked if Buck meant avoiding the principle of “subsecondary” ranks?

**Buck** did.

**McNeill** suggested that Buck may wish to delete “secondary”.

**Turland** did not believe the secondary ranks were the ranks preceded by the prefix “sub-”.

**McNeill** did not think it was an issue as it was quite clear that Art. 3.2 defined the principal ranks and Art. 4.1 the secondary ranks and that these were those that did not involve the word “sub”. He concluded that the wording was perfectly in order and it would not permit “supersub”.

**Nicolson** asked how many were in favour of the proposal as up on the board?

**Redhead** asked if this was an Editorial Committee vote?

**McNeill** clarified that it was a vote on the proposal with the friendly amendment of retaining the Article but adding “super” that the Committee had accepted. So he thought it was the proposal as amended to maintain the existing wording of the Article but add the option of the “super”...

**Turland** disagreed and further clarified that the amended proposal was exactly the same as the proposal which appeared in the synopsis which said “Replace Article 4.3 with the following paragraph”. The amended proposal was to insert the following paragraph in addition to Art. 4.3, which remained unchanged.

**Redhead** was a bit confused with the very first vote taken as to whether it was a “yes/no”, or whether it was an Editorial Committee vote. He pointed out that the Section was again in a situation here where the vote was “yes/no” but it seemed to be for an Editorial Committee vote.

**McNeill** clarified that the amendment had been treated as a friendly amendment, the suggestion of the Rapporteurs had been accepted by Watson on behalf of the Committee for Suprageneric Names.

**Redhead** accepted that.

**Watson** queried whether the proposal was to have Art. 4.3: “Further ranks may also be intercalated or added, providing that confusion or error is not thereby introduced”, full stop, then something like, “The first of these extra ranks will be generated by adding the prefix “super-’ to terms denoting the principal ranks which are immediately subordinate to them”, full stop. He suggested having “super-” as the first of the intercalated ranks.

**Turland** thought it was necessary to say where in Art. 4 the paragraph should go.

**Watson** suggested that was an editorial matter.

**McNeill** assumed so. He added that the Rapporteurs’ suggestion was that it probably precede the present text to indicate that it came first but that would have to be made clear. He outlined that the intention was clearly that “super-” should be used before any additional ranks were put in.

**Turland** clarified for Elvira Horandl who was typing the changes for projection on the screen, that instead of saying “to Article 4”, it should say “before Article 4.3”.

**McNeill** agreed that would be clearer.

**Dorr** raised a point of order that he felt might help move the process along. He noted that there was some confusion as to how people moved on the floor to vote Editorial Committee, he realized in passing motions, typically the motion was “Are you in favour?” or “Are you opposed?”, yet, in the mail ballot, there was also the option of “Editorial Committee” or “Special Committee”. He felt that unless the Chair phrased the motion properly it was very difficult for somebody to vote that something should go to Editorial Committee. He supposed what would have to be done was, voting “Yes, send it to Editorial Committee” or “No, do not send it to Editorial Committee”. He asked the Chair to keep that in mind when dealing with these questions because it seemed that the mail vote, certainly in many instances, favoured having the Editorial Committee resolve whatever minor aspect of the issue it might be.

**McNeill** felt the point was very relevant and very clear, but that in situations where the vote was in favour of the Editorial Committee, the Section could just move that the whole matter go to the Editorial Committee. He elaborated that this was one of those exceptional circumstances in which the Rapporteurs had suggested that the preliminary mail vote “ed.c.” had a special meaning so it could not just be referred to the Editorial Committee because that was a difference in the *Code* from what was proposed. It was beyond the authority of the Editorial Committee to make this change and the Section must make the decision; they had been rather slow in putting out what “ed.c.” meant in terms of the actual change to the *Code* that was what was before the Section in this case. But in the general case of reference to the Editorial Committee he reassured Dorr that his point would be addressed and followed.

**Basu** felt that the term “suprageneric names” was too complicated and could cause confusion or error.

**Hawksworth** suggested that “super” could be added to Art. 4.2 and incorporated there.

**McNeill** noted that this was exactly the type of situation which the Editorial Committee normally had to resolve. He felt that what was quite clearly being proposed was what should be added to the *Code* and how to meld it in most smoothly was the job of the Editorial Committee, while maintaining the meaning of what was about to be voted on.

**Turland** mentioned that that would be changing the intent of the proposal which he felt was that if you wanted to intercalate a rank you use “sub-” and then if you wanted to intercalate yet another rank then you use “super-” and then if you needed to put still more ranks in then he supposed you could make up your own rank. He added that the idea was to leave it open for an indefinite number of ranks, but first use “sub-” and then use “super-”. He gave the example that if you wanted to intercalate a rank above the rank of species but below the rank of genus first you have subgenus, then you could go to superspecies, theoretically, but you would not initially choose superspecies.

**McNeill** pointed out that “section” was available.

**Turland** corrected himself that you would have “section” and “series” and apologized.

**Dorr** was a little concerned about introducing a new hurdle to go through here in the series of ranks because he felt there had been names published where taxonomists had invented new ranks and published names at them. He argued that they were currently theoretically validly published, but if they did not follow this sequence of going through the primary, then the secondary, then the “sub-” and then an additional hurdle of “super-”, he wondered if the requirement would then invalidate those names? He added that sometimes those names then found their way into secondary ranks or other ranks through transfer. He thought it was necessary to be careful about introducing a “super-” requirement here if it was going to invalidate rank names that had been intercalated in the past, as he assumed that it would invalidate all of them.

**McNeill** was puzzled a little by that, as he felt that would suggest that any rank that was intercalated while there was currently a “sub-” option was also not valid.

**Dorr** was trying to get clarification on that issue, he wanted to know what the effect or the penalty was for people who had not followed the correct sequence.

**McNeill** did not think it was something new in the *Code*, as it also applied in the present word of “sub-”. He felt that it was clearly not the intent because the whole thrust of the *Code* took a very different approach where ranks were used that were not one of the ranks specified for validly published names in the *Code*. They were validly published names that only had priority at that [usually undefined] rank but could be used as basionyms or for transfer. [He and Dorr were referring to names published prior to 1953.] His point was that he did not think it [introducing “super-] invalidated any name.

**Schanzer** thought that confusion might arise with regard to superspecies, because species and subspecies were both combinations. He wondered what superspecies would be and by what rules the single names or combinations would be formed.

**McNeill** thought it was a very legitimate point and found superspecies an extremely unhappy concept that he did not see as a terribly useful one to have in the *Code.* He suggested it would have to be a binomial but that was not defined in the [proposed] Article. The proposers should comment on this.

**Barrie** wondered if it would have to be a combination or if it was a rank above the rank of species, which would mean that it was not necessary?

**McNeill** felt that the reason why people would think it was a combination was that in all other disciplines in which this was used, it was treated as such but he found the term a little strange.

**Barrie** thought it was an unfortunate term and hoped people would not take it up.

**Malecot** noted that the proposal was made by the Suprageneric Names Committee, so in his opinion it meant it did not apply to species, varieties, and forms. He suggested amending the proposal reflecting [the mandate of] the Suprageneric Committee so only for primary and secondary ranks above the generic level including the genus.

**McNeill** thought it would be ranks above species, as there was nothing wrong with superseries or supersection. He invited the Committee to comment on whether they wanted to make the proposal apply only to ranks above species, adding that with the wording as it was you could have a supervariety and you could also have a superforma.

**Unknown Speaker** interjected “and a superspecies”.

**McNeill** disagreed, noting that the proposal was that “super-“ apply to ranks above species, so superspecies would not be permitted.

**Watson** personally agreed that it made more sense to be above the rank of species but thought it would be useful to have the other members of the Suprageneric Committee comment on it. He was happy to treat it as a friendly amendment.

**Turland** was happy to accept that as an amendment as well.

**Watson** checked that the amendment was to insert “above the rank of species” after “secondary ranks”?

**Demoulin** would support an amendment that considered that this was a recommendation made by the Committee on Suprageneric Names and it should only concern names above the rank of genus. He thought that the objectionable thing was a superspecies, such as a collective species like *Taraxacum officinale*. He thought that the good thing would be if a greater number of ranks above that of genus was desired, not above the rank of species.

**McNeill** asked if he meant “At the rank of genus or above”? [The amendment was **seconded.**] He clarified that any further discussion should be on the amendment relating to it being at or above the rank of genus.

**Wieringa** seconded “above the rank of species” and was opposed to “above or at the rank of genus”. He felt that for people who might want to include sections or series, it should be possible to have superseries and supersections, but thought the possibility to create a super-regnum should be excluded. [Laughter.]

**Gereau** had a point of clarification: he felt there was no difference between saying “at or above the rank of genus” or “above the rank of species” because there is no secondary rank between the rank of genus and species so it was the same thing.

**Nicolson** suggested subgenus.

**McNeill** noted that section and series were secondary ranks, surely.

**Gereau** retracted his comment.

**Watson** wished to confirm that because you were still allowed to add further ranks, that did not stop people using the term “super-” below the rank of genus anyway.

**McNeill** confirmed that was correct, so long as no confusion would arise thereby.

**Turland** believed that on behalf of the Suprageneric Committee, Dr Watson and he accepted “above the rank of species” as a friendly amendment as that would preclude the use of superspecies.

**McNeill** summarized that it “at or above the rank” was not a friendly amendment, the amendment had been seconded and there had already been some discussion. He added that there was further discussion on restricting the application of “super-” to ranks of genus and above.

**Turland** thought that the proposed wording was becoming too complicated and it would be better simply to vote on the original proposal, as to whether the Section wanted it or not, because even if the original proposal were defeated it would still be possible to use “super-” and he thought what was being introduced into the *Code* was becoming rather trivial and would simply complicate it.

Given that **Demoulin** thought the real problem was that of superspecies, he suggested that there was still another way out; instead of having “above the rank of species” or “.. genus” to simply have “to the term denoting the principal or secondary ranks, species excepted”.

**McNeill** noted that the amendment was not seconded, so discussion returned to the amendment on the board, “at or above the rank of genus”.

**P. Hoffman** was not convinced that Demoulin understood the first amendment correctly as that friendly amendment already precluded superspecies, therefore his amendment was superfluous. She thought he only wanted to preclude superspecies and not supersection and superseries.

**Demoulin** confirmed that was the case.

**P. Hoffman** reiterated that the inclusion of “above the rank of species” already precluded superspecies.

**McNeill** clarified that the amendment was not up for discussion as it had fallen. He added that what it would actually do was allow supervariety and superforma as the only thing it would do that was different from the original proposal but not different from this one.

**Demoulin** entertained the possibility that he may be wrong, but as he had been on the Editorial Committee for 30 years and if with that experience he understood that “above the rank of species” included superspecies, he guessed there would be a lot of people who would understand it that way.

**McNeill** thought the discussion had become semantic, and that the suggested amendment should be forgotten because it had not been seconded and the Section should go to the matter before them, whether the particular specification of “super-” should be restricted to ranks of genus and above or whether it should be allowed for ranks below genus but not including species and below.

**K. Wilson** pointed out that what was on the board did not reflect what was being discussed and noted that “at and above the rank of genus” needed to be added.

**McNeill** agreed.

**Zijlstra** argued that if the amendment were accepted there would be two kinds of ranks with the addition “super-”, those permitted by Art. 4.2 *bis* and those stipulated by Art. 4.3. Supervariety, of course, still would be possible under Art. 4.3 and she considered it quite ridiculous to have two kinds of “super-” ranks.

**Moore** tended to agree with that comment. He felt that if a new prefix was to be introduced it should be parallel to Art. 4.2 and use some sort of prefix other than “sub-”. He thought that “super-” was getting rather super-complicated. His main point was that adding “super” in a manner not parallel to Art. 4.2 was undesirable.

**Turland** suggested going back to the original proposal and simply voting on that, because he was not sure that progress was being made with making amendments. He thought it boiled down to whether the Section wanted to use “super-” at all, to actually include the advice to use “super-” in the *Code* or just leave Art. 4.3 as it was, which would allow it if people wanted to use it.

**Barrie** noted that if the proposal was amended to include “denoting the principal or secondary ranks above the rank of species” that was more of a restriction to the application of the prefix “super-“ than what was currently permitted in the *Code* as it was already possible to use “super-” at any rank.

**McNeill** summarized the state of play noting that Turland had just said that the Committee for Suprageneric Names itself was withdrawing their acceptance of the amendment to restrict the use of “super-“ in order to keep the original proposal, which would include the option of superspecies. However, he went on that there was an amendment and that amendment was seconded so if the proposer of the amendment that said that it should be terms above the rank of species, wanted to speak further now that would be appropriate. He argued that the Committee for Suprageneric Names could not alter an amendment that was actually moved and seconded but then it became a friendly amendment which they were now reneging on.

**Watson** thought that there was a general acceptance for “above the rank of species” because people wanted to have supersection, superseries, supergenus.

**McNeill** felt that there was no general acceptance of anything, so was working strictly on procedure and obviously there was the original proposal, there was an amendment to make it above the rank of species, still another amendment to make it at the rank of genus or above.

**Woodland** felt that nomenclature, as it had been worked on over many years in the *Code*, was to simplify things and make it easier, not make it more complex and difficult. He felt that the proposal for Art. 4.3 for inserting “super-” above the rank of genus did little to improve the *Code* and thought the amendments and original proposal should be rejected.

**Redhead** pointed out that the original proposal unmodified by the Editorial Committee to replace Art. 4.3, was restricting it further because Art. 4.3 as it was currently worded suggested that you may intercalate other terms provided there was no confusion. He argued that if you replaced it with the other, that option was gone, you add “super-” to it and there were no options for any others. He wished to know if that was what the discussion was going back to, the original proposal?

**Turland** apologized for the confusion. He did not mean the original-original proposal. [Laughter.] He meant talking about the proposal as was suggested by the Rapporteurs in the Rapporteurs’ comments. Basically he was suggesting that the Section vote on what was on the screen without the words “at and above the rank of genus”. He continued by clarifying that when McNeill was talking about the Suprageneric Committee reneging on their agreement to a friendly amendment, the friendly amendment was the addition of the words “at and above the rank of species or genus” that you saw on the screen and that had just been removed.

**Rijckevorsel** pointed out that formally it was an amendment and it was seconded, so it should be either withdrawn or voted down and then discussion could return to the original.

**McNeill** asked if he was withdrawing?

**Rijckevorsel** was not withdrawing. He was saying as a point of order that if it was not withdrawn it should be voted on.

**McNeill** agreed that that was exactly his point but he thought the person who had proposed that the application of the prefix “super-” be “at the rank of genus or above” might want to say why they wanted it to be in that way. He suggested that then the Section could take a vote on that amendment and if it was passed, it would become a substantive motion.

**Per Magnus Jorgensen** thought there were two different matters; which rank should it be allowed for and where it should be placed.

**McNeill** clarified that where it should be placed had been dealt with and the discussion was strictly about which ranks.

**Rijckevorsel** explained that he did not understand anything of the proposal but his reason for seconding the amendment was that he felt that if a Committee on Suprageneric names gave advice, it should apply only to the ranks above genus.

**McNeill** suggested moving to the vote on the amendment to restrict the instruction to use “super-” to terms at the rank of genus and above.

[The amendment was **rejected.**]

**Nicolson** instructed that that point should be removed from the screen and the Section move to a vote on the original proposal.

**McNeill** disagreed as he thought the word “species” was still on the table, so it would be “secondary ranks above that of species”.

**Nic Lughadha** wished to check that she understood what was going on. She believed some people may vote for this version on the understanding that it would avoid superspecies. However her understanding was that it would not, it would simply not recommend the use of superspecies.

**McNeill** noted that the provision that might, depending on your understanding of the phrase, argue against superspecies might be deemed to be causing confusion as to what the difference between a superspecies and a species was. He was inclined to think that that was an arguable case but the *Code* did not rule precisely on it.

**Nic Lughadha** thought it just introduced confusion and agreed with Woodland that it did not add value to the *Code*.

**Demoulin** noted that after reading it three times, he agreed that it would be okay to get rid of superspecies, but he thought the Editorial Committee would have to work on it so that everybody read it correctly the first time.

[The amendment was **rejected.**]

**McNeill** returned to the original proposal, as modified by the Rapporteurs and accepted by the proposers.

**Prop. A** as amended was **rejected.**

### Recommendation 5A (new)

**Prop. A** (142: 8: 8: 0).

**McNeill** introduced a proposal to include a new Rec. 5A which had received quite substantial support in the preliminary mail vote.

**Turland** stressed that this was only a Recommendation, therefore it had no mandatory implications and was just there for guidance.

**Prop. A** was **accepted.**

### Article 6

[*The following debate, pertaining to a New Proposal presented by Wieringa regarding Art. 6.2 took place during the Eighth Session on Friday afternoon*.]

**Wieringa’s Proposal**

**McNeill** introduced a new proposal from Wieringa which suggested inserting a Note about creating a name not necessarily defining a particular taxonomic circumscription. He read out the exact wording of the suggested Note for Art. 6.2 “Valid publication creates a name, or in the case of a simultaneously created autonym creates two names, but does not of itself for nomenclatural purposes define any taxonomic circumscription beyond inclusion of the type of the name (Art. 7.1)”.

**Wieringa** explained that it was the Note as the Rapporteurs had suggested it be worded [in their Comments on Art. 22 Prop. C in Taxon 54: 226. 2005], only he had inserted in it the case of autonyms, which was not in their wording for the Note and as it was for these that it was intended, he felt that a bit strange. He also noted that there was some opposition to the proposal because it said that creating a new name did not have any taxonomic implications and so he proposed adding “for nomenclatural purposes”. He thought that it was now clear that it was only for nomenclature that a new name did not have any circumscription.

**P. Hoffmann** thought an autonym was always simultaneously created and felt that “simultaneously created” should be deleted.

**Wieringa** responded by saying that the autonym might already exist. He continued that it was possible that someone was describing a third subspecies, in which case there already was an autonym.

**McNeill** agreed that there might be some minor editorial modification that might be needed.

**Barrie** felt that taxa were not defined for nomenclatural purposes, and that was a problem for him with the proposal.

**Nicolson** suggested changing “define” to “create”, but was not sure.

**K. Wilson** suggested “imply”.

**Nicolson** asked the proposer if that was acceptable. [It was.]

**Turland** had one little suggestion which he suggested may or may not be a friendly amendment. Instead of saying “or in case of a simultaneously created autonym creates two names” he suggested “and sometimes also an autonym” and then just referring to the autonym Article where “autonym” was defined? So “Valid publication creates a name and sometimes also an autonym (reference) but does not itself” *et cetera*. [This was accepted as a **friendly amendment.**]

**Wieringa’s Proposal** was **accepted.**

[*Here the record reverts to the actual sequence of events*.]

### Article 7

**Prop. A** (27: 123: 7: 0) was ruled as **rejected.**

**Prop. B** (26: 114: 13: 1).

**McNeill** moved onto Art. 7, Prop. B which had received 74% “no”: and so was just open for discussion.

**Brummitt** noted that he had learned by bitter experience over many Congresses that if you did not get the Rapporteurs behind you, you had virtually little chance of succeeding with a proposal. He did not want to take up the Section’s time on the basis of getting one percent over the minimum required but as the proposal came from the Committee for Spermatophyta he felt obliged to say something about it. He explained that the case that brought it up was very intricatebecause when the name [*Gilia splendens*] was validated it included a subspecies *grinnellii*, which was based on an earlier specific name. So *Gilia splendens*, when published, included the type of an earlier specific name and so was illegitimate. But he pointed out that the *Code* said that the type of the illegitimate name had to be the same as the type of the name that should have been used, so the type of *Gilia splendens* had to become the type of *Gilia grinnellii*. He felt that this was just nonsense. He described it as two parts of the *Code* conflicting with each other and the proposal was simply to try to get some sense into the *Code*. The comments by the Rapporteurs that the proposal is nevertheless flawed struck him as very odd, because it was not flawed. He had extensive discussion with the Rapporteurs before the proposal was finalized. He felt that what they objected to as being a flawed proposal was in fact the direct thrust of the whole proposal to make it clear that the type of *Gilia splendens* was not perversely the type of an atypical subspecies. He noted that it was an issue that had arisen four times in his experience and it had very little practical effect. He concluded that it did not really matter what the type of an illegitimate name was anyway, as they could not be used he felt it was pointless to argue about it.

**McNeill** noted that the case, as the Rapporteurs had commented, had a certain plausibility. The difficulty they felt with the proposal was that it did not solve the problem clearly, because there was already provision in the *Code* if there was indication of a definite type, but these situations did not apparently indicate this.

**Brummitt** wanted to make another point that if the type of *Gilia splendens* is the same as that of *Gilia grinnellii*, it meant that the combination subspecies *grinnellii* was the typical subspecies, therefore it should have been called subspecies *splendens*, so the name was not even validly published. He argued that, at the present, there was a ridiculous situation with a knock-on effect which made things not even validly published.

**Demoulin** believed that the reason that this was taken as having a negative comment from the Rapporteurs was the last sentence saying that “the proposal would leave typification of an illegitimate superfluous name unresolved in these circumstances”. He did not think this was a reason to oppose the proposal. He strongly opposed the idea of the automatic typification of superfluous names and felt Art. 63 had been a nuisance in the botanical *Code* for 50 years. It was one of the first things he learned from his master Donk when he started doing nomenclature. He stated that the concept of illegitimacy by superfluity was absolutely flawed and he felt it had been causing problems and problems and problems and little by little they were being solved by special rules. He thought it would have been much easier to delete the whole thing. He thought that Brummitt’s proposal was a little improvement in the right direction and he supported it.

**Gandhi** wanted to add a few additional bits of information for the audience who were not aware of the *Gilia* problem. Nearly 14 years ago, he and his colleague John Kartesz found out that *Gilia splendens* was validated by Alva Day and Mason in the 1940s. Day & Mason were thinking that it was validated by Bentham in 1830s, so, under this wrong impression, they included *Gilia grinnellii*, which was published around the early 1900s, in their *Gilia splendens*. With his colleague, John Kartesz, he had sent an article to *Taxon* and then the error was caught by Dan Nicolson [Nomenclature Editor], that under the species name [*Gilia splendens*] an earlier species name, the basionym of the subspecies *grinnellii* was included. Then they realized that although validated in the 1940s, *Gilia splendens* was illegitimate when published and, of course, the article was never published and later on a proposal to conserve the name *Gilia splendens* so that it could be widely used was published [in Taxon 53: 842–843. 2004].

**Prop. B** was **accepted.**

**Nicolson** noted that **Prop. C** (137: 2: 18: 0) was very strongly supported.

**Bhattacharya** felt that in Arts 7.2, 7.7, 7.10 and 7.11 appropriate reference to Art. 14.9 must be added as an editorial note, rather than as a mention under Note 2 of Art. 48.

**Turland** asked him to write down what he was proposing to change so it could be displayed on the screen.

**Barrie** made the point that the majority of the parenthetical references in the *Code* had been added by the Editorial Committee for clarity and they were not things that were voted on in the Congress. He continued that the cross-references were added so people could find other places in the *Code* where things belonged. He felt that adding things was not a problem, if it was brought to the Editorial Committee’s attention it could be put in.

**Turland** did not think that the suggested amendment was a modification of the proposal as it did not seem to be relevant to Prop. C, which was to add a reference to Art. 9.18 to the parenthetic reference currently in Art. 7.11. He was not quite sure what Bhattacharya was proposing to change because the notes he had received did not appear to be relevant to the proposal currently under discussion.

**Bhattacharya** maintained that Art. 14.9 must be mentioned as an editorial note in Arts 7.2 and 7.11 and other places and it was only mentioned in Art. 48.

**Turland** read out what he had suggested: “In Articles 7.2, 7.7, 7.10 and 7.11 appropriate mention of Article 14.9 must be added too as an editorial note”, and then you have “We require it rather than as an omnispective mention under Note 2 of Article 48”. The other Note said “In Article 7.2, appropriate reference of Article 14.9 must be added as an editorial note too”.

**McNeill** thought it sounded like a quite separate proposal, which if the President felt appropriate could be taken from the floor, perhaps at the end of the discussion of this Article.

**Prop. C** was then **accepted.**

**McNeill** thought there were are two alternative texts from Bhattacharya and asked the proposer to indicate which one he would like to have put up on the board and then that could be considered as a proposal from the floor.

**Nicolson** thought that Bhattacharya’s notes were editorial comments that could be referred to the Editorial Committee to consider. [Bhattacharya agreed.]

**Bhattacharya’s** proposals were ruled as referred to the **Editorial Committee.**

[*The following debate, pertaining to a New Proposal in Art. 7 presented by Gandhi to clarify what sort of types were meant in 7.11 took place during the Ninth Session on Saturday morning*.]

**Gandhi’s Proposal**

**Gandhi**, the proposer, considered the proposed change to Art. 7.11 non-controversial. After the St Louis Congress there was some confusion amongst botanists as to whether while citing a holotype they needed to state “here designated”, even though the Article related only to lecto- and neotypification, since the word “type” used there was quite general. He had been contacted by journal editors, and while he had assured them it was not necessary, some journals and authors had started to do this to be on the safe side. In order to avoid ambiguity, the word “type” needed to be replaced by “lectotype, neotype, or epitype”.

**Nicolson** wondered if this was just an editorial suggestion.

**McNeill** concurred, but wondered if “epitype” belonged there. It was a confusion that definitely had occurred and which the Editorial Committee should address. Although not ambiguous to those familiar with the *Code*, it had been misread, and he wondered if Gandhi would be prepared for this to be referred to the Editorial Committee.

**Veldkamp** wondered if the wording might be copied from Art. 8.1.

**Watson** supported the proposal, especially as in the Index to the *St Louis Code* the word “holotype” was incorrectly cross-referenced to Art. 7.11.

**McNeill** acknowledged that that was a mistake in the Index. He was still unsure if “epitype” should be included, as once chosen it was chosen, but it was certainly appropriate for the other two.

**Demoulin** wondered if it would be the best thing to also include a direct indication on holotypes.

**McNeill** reminded him that this provision had nothing to do with holotypes.

**P. Wilson** had corresponded with Greuter and McNeill on this before as it had come up in papers he had had to review where it had been used. From the record of the St Louis Congress, the wording was not exactly that recommended, as the Editorial Committee had evidently felt that because of the cross-references it was not necessary to be as explicit as the St Louis meeting had suggested. It did need to be made more explicit as not all readers were recognizing the import of the cross-references in the Article.

**Gandhi’s Proposal** was referred to the **Editorial Committee.**

[*Here the record reverts to the actual sequence of events*.]

### Article 8

**Prop. A** (78: 30: 8: 28).

**McNeill** moved on to Art. 8 and introduced the first two proposals, which both related to microfossil organisms. He reported that the preliminary mail vote was positive in both cases to some degree.

**Skog** introduced herself as secretary of the Committee for Fossil Plants and reported that the Committee was not in favour of the proposal. There were three positive votes, six “no” and six abstentions on the Committee, which in the mind of the Committee was taken as not representing support for the proposal at all. There were a number of reasons for the lack of support, which she was happy to explain if that was desired.

**McNeill** thought it would be worth explaining why the Committee was opposed to it, adding that he did not think that the Rapporteurs were intending to guide the Section, except to say that if it was something that was seen to be workable by palaeontologists there was no other obstacle in the *Code*.

**Skog** explained that the proposal had been before the *St Louis Code* in a slightly different form. The basic problem was the fact that in that proposal there was no definition of the term “microfossil”. She added that the proposer had defined microfossils in this case, but had defined them in such a way that those people working on dinoflagellates as well as some of the other calcareous algal forms were not supportive because they wished to have a specimen as type, not an illustration as type because an illustration cannot be rotated or examined under different positions. It was also not clear to many people as to which illustration would serve as the type – whether it should be a proximal or a distal view of the microfossil involved. She reported that there had been a lot of question and discussion in the Committee about which view was appropriate and that resulted in a lot of abstentions. In addition, the wording of the proposal would make it possible to never choose a specimen as a holotype but simply go directly to an illustration. Many people felt that it would be okay to choose a lectotype perhaps as an illustration or choose a neotype as an illustration, but not to have the very first designated holotype be an illustration. She concluded that the fossil plant Committee had had a great deal of discussion about the matter and decided that in its current form there was no strong support for this particular proposal.

**McNeill** added that he had had a communication from Al Traverse a month or so before the meeting saying that he was very sorry not to be able to be present to speak to his proposal due to quite serious ill health of other members of his family. He opened the floor for general discussion.

**Brummitt** thought the Section may be surprised to hear him talking about fossils, microfossils.

**Nicolson** joked that “we’re all getting to be fossils”. [Laughter.]

**Brummitt** added that he had to mention algae for the first time in his life as well. He wanted to point out that the whole question of illustrations as types was part of much wider subject which was going to come up under Art. 37.4. He had been in contact with algologists about illustrations as types which they said were absolutely essential. While he personally did not want to oppose the proposals, he wished to point out that the implementation of them in the *Code* would presumably be subject to what came up under Art. 37.4, and so the Editorial Committee would have to equate or evaluate two decisions.

**Skog** was not sure if it was worthwhile or not but was just going to point out that epitypes could be illustrations. Microfossils could have an epitype designated, an illustration to elucidate the actual material, and she thought that part of her Committee’s decision was that that would cause some of their members to purportedly lose sleep if it were to pass.

**Demoulin** wished some palaeobotanists who were on the side of Traverse would speak on that but if no-one was present, he had something to say. [No-one spoke up.] He continued that he was not a palaeobotanist but was often consulted as a nomenclaturalist by palaeobotanists and also as one of the last cryptogamists. He knew the problem from the side of present microorganisms, whether algae as Brummitt said or eventually fungi, and he remembered well the thing presented by Traverse at previous congresses and acknowledged that there was a problem in agreement that was reflected by the large abstention in the Committee. He thought that the most significant thing in their vote was the abstentions. He felt that the problem was a rather technical one, regarding how to be certain that if you wanted one specimen of something that was five micrometers long, it would really be possible to find it on microscopic slide? He suggested specifying coordinates on some given microscope but the problem existed with anything that had to be typified by a permanent slide. He noted that it was especially difficult for the palynologists because they may have preparations which included a much larger number of objects than people who were dealing with living micro-organisms, for which they could choose something which was enriched, eventually came from a culture or was more or less homogeneous and in which case you could designate the whole slide as a type. But he felt that if there was a mixture of diatoms, dinoflagellates, coccolithophorids and anything like that and perhaps 50 different kinds of organisms present, how could you be certain that the specimen was a given one? He explained that was why Traverse and others preferred to have an illustration that represented exactly what the person who named the taxon had in mind. He did not understand the technical objection that you could not rotate or have a proximal or distal view, arguing that a permanent slide was no different from an illustration, except maybe that you could use higher magnification of your microscope. He thought it was time the discussion was closed and given the rather good support, even if there was a lot of abstention in the fossil plan t Committee, he thought the proposal should be passed and stated that he would vote for it.

**Skog** wanted to remind everybody that in fossil plants, even though the specimen was designated as the holotype, they must be accompanied by an illustration in the publication and one of those published illustrations must be of the type material. So the Committee did not see that the proposal would improve the situation as most people did indeed use the illustration that was of the holotype in the publication. The illustration showed most of the characters and the fact that that actual specimen did exist and could be found made a lot of people happy, because apparently in the case of dinoflagellates they could be removed from the slides and re-examined using new technology and new techniques that might not have been available to people in the past. She added that many advances in palaeontology had come about because of the introduction of new techniques and having the actual specimen had allowed them to do so.

**Demoulin** felt it was important to stress that the proposal just said “may serve as type”, not “must be type”, it was like microorganisms for which there was a problem in preserving them.

That was not what **Nicolson** had read.

**Zijlstra** wondered if some people had forgotten Art. 8.2, which said that the type may be multiple smaller individuals together forming one specimen, like a number of, for example, *Dinophyceae* cysts all lying in different directions and then they all formed the type and an illustration was often is only one view of one specimen. So she felt that a slide may give much more information than an illustration because it offered several individuals in different views.

**McNeill** admitted to knowing nothing about these things in practice but his information was that the normal practice was to try to isolate an organism on a slide, because otherwise you may have material that may be quite taxonomically diverse on the slide...

**Demoulin** agreed that exactly what the Rapporteur said was the main point of his intervention. One must stress that with palynological slides you had got many different things. If dealing with organisms that had been cultivated and the slide was prepared from a homogenous culture, he thought it was okay to have a slide as type. He felt that the problem was speaking of things taken from nature, from a rock, and there could be 50 different species in it. So the problem was how could you be certain that the single cell you were looking at was the one the author wanted to be the type?

**Gandhi** noted that his palaeobotanist colleagues were also opposed to the view that illustrations could serve as types to microfossils, nevertheless, as a group they said that the Committee on the fossils should take the lead, whether accepting or rejecting the proposal.

**Prop. A** was **rejected.**

**Prop. B** (77: 26: 12: 28) was ruled as **rejected** as it was a corollary to Art. 8 Prop. A which was rejected.

### Recommendation 8B

**Prop. A** (9: 149: 1: 0) was ruled as **rejected.**

## Second Session

Tuesday, 12 July 2005, 14:00–18:00

### Article 9

**Prop. A** (68: 34: 20: 29) was ruled as **rejected** as it was a corollary to Art. 8 Prop. A which was rejected.

**Prop. B** (36: 104: 15: 1).

**McNeill** introduced Art. 9 Prop. B from Brummitt on syntypes and isosyntypes. He noted the result of the mail vote (see above).

**Brummitt** reported that the proposal was also from the Committee for Spermatophyta and concerned the now famous case of *Gilia grinnellii* and *Gilia splendens*. The question arose in the Committee as to whether a duplicate of a lectotype took precedence over a cited syntype. The exact case was *Gilia grinnellii*, which was based originally on three collections which turned out to be taxonomically different. One was in the Berlin Herbarium, which sadly was destroyed during the Second World War, and the other two collections were elsewhere, extant specimens, but it was the Berlin specimen which was chosen as the lectotype. He asked the Section for guidance on this for the Committee. As they had commented, they felt it was clear in the guide to the choice of types in the early *Codes* but somehow it got lost in the future development. He noted that the Rapporteurs had said that it was sensible as a Recommendation but some may query the desirability of making it mandatory. His feeling was that Recommendations were fine but they did not provide an answer. He added that it was a very small point, that did not arise very often but he felt that clarity was needed in the *Code* and considered it a critical case. As the application of the name depended very much on it and several other cases had come up since, he thought it should be written into the Article of the *Code* and not be just a Recommendation.

**Gandhi** really wondered about the typification of *Gilia grinnellii*, as the whole situation in the case was quite complicated because the existing syntypes did not agree with the protologue even though they were mentioned. Additionally regarding the specimen that was destroyed in Berlin, no specimen could be found at the type locality that fitted the description of the protologue of *grinnellii*. He suggested it might be better to include some other example in connection with this particular proposal.

**McNeill** summarized that it would seem that the proposed amendment would not actually address the particular case, which might be addressed in other ways. He wondered if Barrie wanted to say anything about this in the point of view of “original material”. He suggested this because he felt that the argument being presented in the proposal was that a syntype that had been seen by the author should have precedence in the process of lectotypification over what was also defined nowadays as original material, namely a duplicate that may or may not have been seen.

**Barrie** said that the current wording came in at St Louis and was part of the report of the Special Committee on Lectotypification. His assumption was that isosyntypes were of lesser status than syntypes. But most of the examples he had been thinking about at the time were examples where a collection was cited but not a specific specimen. In that case presumably all the specimens of that collection would have the same status of syntype, no matter where they were. He added that this was a very special situation where someone had cited two or three specific specimens indicating which herbarium they were in. He thought it was safe to assume that the author saw those three specimens and his concept was based on those specimens and that any duplicates in other herbaria we know nothing about whether he saw them or didn’t see them and how should they come into play. He thought the proposal stated what was somewhat the intent of the original Committee when they wrote it. He noted that the Rapporteurs had brought up the issue of whether or not it was going to threaten the typifications of names already typified.

**McNeill** interjected that it would mean the lectotype typification would not be in order and another specimen could take precedence over it.

**Barrie** could not offhand think of any examples of a name like that. He suggested that the same problem existed either way, where in these situations the lectotype was chosen for names because it was the only taxonomically correct element. He continued that if you were forced to look at the other elements and choose one of them then you were changing the meaning of the name and would have to go to conservation or something like that. He concluded that if people found it a useful clarification, then he would support it.

**Gereau** disagreed with characterizing the proposal as a clarification, he felt it was a change in existing practice and a move toward yet another step in a hierarchy of procedures that was already adequately addressed by the current *Code*. He recommended strongly against it.

**McNeill** agreed that it was putting another step in, but whether it was desirable or not to do so he left for the Section to decide.

**Wieringa** thought that it was far more stable for nomenclature if it was possible to choose isosyntypes. He gave the example if one of the syntypes had been chosen as a lectotype and that lectotype was destroyed, that it would be possible to again lectotypify a duplicate of the lost lectotype, rather than having to move to one of the other syntypes which was seen and which might in the end prove to be another taxon and would result in having to go back on the first lectotypification. He advocated giving monographers a bit of freedom in which specimens they could choose from.

This reminded **Brummitt** that when the *Gilia grinnellii* case came up they knew that the holotype had been destroyed at Berlin but did not know where there were any duplicates. He had to write round at least six different herbaria asking “Have you got duplicates of this collection?” and his investigation may not have been exhaustive. He argued that even if you had taken one of the other specimens, if somebody discovered a duplicate of it in yet another herbarium, then the whole position was reversed.

**McNeill** had to say he understood the wording of the proposal and its additional step and perhaps complication, because obviously if you were lectotypifying, it would be nicer to be able to have greater freedom in ensuring that your choice reflected the existing usage of the name. So it seemed to him to require looking at a cited syntype before looking at a duplicate of one, as another duplicate was just as restrictive and might lead to a name change. What he did not understand from the examples was how this has really any bearing on the type. Because if it was a holotype then clearly a duplicate of a holotype is original material in any case. In a numbered collection the duplicates would have to be considered first in any case, so he was not too clear it helped. He added that that was perhaps irrelevant and the key thing was not how it applied to *Gilia* or any other genus, but what it was doing to the *Code*. It was very clear to him that the proposal was increasing the logical steps and he felt it was a question of whether that was desirable or not. It seemed to him that it was an added complication but he did not object to that if the Section wanted to put it in as it would make it perfectly clear.

**Gandhi** wanted to add that at Harvard they had been indexing lectotypifications for quite a long time. He found the proposal palatable if a date was stipulated, because if retroactive it may destabilize what had been already indexed and what had been available to the botanical community. He also wanted to add one more piece of information about *Gilia grinnellii*, even though it was just an example. It was Jepson who cited the first element as the type and he was unaware that he was designating a lectotype. He merely said that Grinnell’s collection was the type. In his research they had contacted about 10 herbaria in the early 1990s regarding whether any duplicate of Grinnell’s collection was available but none of the herbaria contacted seemed to have any duplicates.

**McNeill** asked Gandhi for clarification on his first point? He wished to know if Gandhi had said that from his indexing of lectotypes he knew of cases where adding the proposal would cause a change to lectotypes?

**Gandhi** confirmed that and explained that this was why he was suggesting that it was a good proposal if a date was stipulated as long as it was not retroactive.

**McNeill** wondered if it would be better as a Recommendation than as a rule? He noted that there seemed to be some support for the Rapporteurs’ view that it could be destabilizing in terms of existing lectotypification.

**Prop. B** was **rejected.**

**McNeill** moved on to Art. 9, Props C to M, which had all received more than 75 per cent “no” votes and he reported that unless there was a request for a discussion they would be declared defeated.

**Bhattacharya** wished to have them discussed.

**McNeill** asked if there were others who wished to have the set of proposals by Mukherjee raised?

In the absence of any support for further discussion **Nicolson** was about to move on.

**Bhattacharya** had talked to Mukherjee and he agreed with him and wished to defend the proposals with a few lines.

**McNeill** clarified for Bhattacharya, that there needed to be five people taking up discussion of these proposals, so four others were necessary before they could be considered.

**Nicolson** asked again if there was anyone else who wished to have the proposals discussed more fully? [There was not.] In the absence of other supporters, he ruled that the proposals failed.

**Prop. C** (8: 144: 3: 4), **D** (6: 146: 3: 4), **E** (7: 146: 2: 4), **F** (6: 145: 3: 5), **G** (6: 146: 2: 5), **H** (6: 145: 3: 5), **I** (6: 146: 2: 5), **J** (6: 145: 5: 5), **K** (6: 146: 2: 5), **L** (7: 144: 3: 5) and **M** (6: 144: 4: 5) were ruled as **rejected.**

**Prop. N** (27: 90: 36: 3), **O** (32: 63: 59: 2).

**McNeill** introduced Art. 9, Props N and O as part of the same package but dealing with Examples and noted the voting. He suggested they could be referred to the Editorial Committee or the Editorial Committee could just look at it on its own basis.

**K. Wilson** thought they were worthwhile proposals and moved that they be considered for adoption.

**Nicolson** noted that Prop. N was to amend the Article and delete the first sentence.

**McNeill** added that they were two editorial suggestions. He confirmed that the recommendation was that the two be referred to the Editorial Committee. [The **motion** was **seconded.**] He decided that it would be better to separate the proposals and moved onto dealing with Prop. N, but noted that Prop. O was similarly an editorial matter.

**Nic Lughadha** thought it was a very sensible proposal and wished to support it.

**Nicolson** asked if there was any further discussion and moved to a vote when there apparently was not.

**Unknown Speaker** requested clarification about the vote.

**McNeill** confirmed that the vote was to refer the proposal to the Editorial Committee.

**Nicolson** repeated that it was to refer the proposal to the Editorial Committee.

**McNeill** gathered that there was a desire to have it passed as a proposal.

**Nicolson** asked for a vote of all those in favour of Prop. N. He reported that the vote was very close and it looked like there would be the first show of cards. [Laughter. Aside discussion.]

**Unknown Speaker** suggested that the Section did not understand what they were voting about.

**McNeill** clarified what was being voting on. He had originally suggested that the proposal be referred to the Editorial Committee but actually people wanted to vote on the proposal as it was, so that was what had happened. He noted that while the Editorial Committee could always make the wording better, it could not change the meaning of the proposal, and so referring to the Editorial Committee meant that the thrust should be adopted but the Section were less happy with the wording. However, the point was that a change to the *Code* was being proposed in that particular Article and that was what was being voting on.

**Unknown Speaker** did not understand what the thrust of the proposal was.

**McNeill** asked if somebody who supported it wanted to clarify that for the benefit of the questioner and suggested that Eimear Nic Lughada might as she had said earlier that it was an excellent proposal?

**Nic Lughadha** commented that when they [Kew staff] had looked at it in detail six weeks ago they had supported it, but she had not prepared any notes on it.

**Barrie** felt that the proposals did not change the meaning of anything that was in the *Code*, they were simply editorial. He thought that the question became do you think the wording was clearer than what was in the *Code*? He suggested it was something that might be best referred to the Editorial Committee.

**McNeill** thanked Barrie and added that that was his original thought on the matter, that there was some merit in them that should be looked at but he was not convinced that the wording was necessarily the best. He deferred to the Section.

**Watson** commented on the terms that were being proposed in Prop. O. He thought that the proposal was saying that the supported type could only be a lectotype or the epitype could only be a lectotype or neotype, whereas the epitype could also support a holotype. He argued that you could not just replace the supported type with lectotype and neotype.

**McNeill** pointed out that although he did suggest the proposals belonged together when they were talked about being referred to the Editorial Committee, he thought the Section should just concentrate on N for the moment because they were definitely different things.

**Nicolson** asked for another show of hands just because he was not sure everyone understood exactly what was been asked. He clarified that the Section was considering whether the proposal should be either referred to the Editorial Committee or voted on.

**Prop. N** was referred to the **Editorial Committee.**

**Prop. O** (32: 63: 59: 2) was then taken up.

**Watson** apologized for getting ahead of himself last time he spoke. He explained that the suggestion was for changing “supported type” in Note 4 and replacing it with the words “if the lectotype or neotype is superseded, the epitype has no standing”. He added that, depending on what definition of superseded was used, this would include holotype and a holotype could be superseded if it was destroyed. So he felt the proposal was a definition thing.

**Gandhi** pointed out that Note 4 was not on the screen.

**Turland** clarified that it concerned Art. 9, Note 4. In the context of that Note and the preceding Article, Art. 9.18, it seemed to him that the type could only be a lectotype or a neotype. He added that it talked about superseding the supported type.

**Buck**? noted that Art. 9.7 listed holotype as a possibility for epitypification.

**Turland** pointed out that Prop. O referred to Art. 9, Note 4 and the supported type in the context of [the second sentence of] that Note could not be a holotype.

**McNeill** [noting the first sentence] said that it could in fact be.

**Barrie** thought the discussion showed why Mukherjee had made the proposal, because the Note was not clear. The Note referred to what was happening in Art. 9.18, in that situation if the original holotype was lost the epitype would have no status and a lectotype would have to be designated. He thought that presumably a lectotype that matched the epitype would be designated. He continued that, in fact, you might even designate the epitype as a lectotype, if it were eligible.

**McNeill** suggested that the proposal be referred to the Editorial Committee. He thought that the point was that if in fact – and it was a real situation – an epitype had been designated for a holotype that was a specimen, i.e. not an illustration, and then that specimen was lost, then the question was what was the status of that epitype and presumably the Note still applied there, that you had to choose a lectotype as it would not be possible to automatically treat the epitype as continuing to exist. He concluded that therefore the Note applied to a holotype as well as a lectotype.

**Barrie** thought that was all right.

**McNeill** thought it still may be beneficial wording in the proposal to clarify the issue so he was all for, if it was the mind of the Section, referring it to the Editorial Committee.

**Zijlstra** pointed out that Art. 9.7 said that an epitype could be for holotype, lectotype or previously designated neotype, then in Note 4, she felt that “supported type” was the correct term to catch all those three together. She felt that the proposal should be rejected.

**Demoulin** thought the Note should stay as it was. He stated that the holotype could be superseded by conservation and felt that the Note only dealt with the problem of the type, whatever it was, that had been superseded. He did not feel the need for it and thought that the proposal would considerably change the meaning. He encouraged the Section to vote no.

**Marhold** was happy with the current wording of Note 4 and did not think that the change would improve anything.

**Nicolson** summarized that the Section did not wish to refer the proposal to Editorial Committee but wanted to vote.

**Prop. O** was **rejected.**

**Prop. P** (7: 143: 6: 2) was ruled as **rejected.**

[*The following debate, pertaining to New Proposals by Gandhi and Tronchet to insert Notes in Art. 9 took place during the Ninth Session on Saturday morning*.]

**McNeill** commented that the first proposed Note was from Gandhi, and the second from Tronchet and that this one was related to and overlapped with another proposal coming up shortly. He then invited comments on the proposed Note 1 which was independent of the other two.

**Gandhi’s Proposal**

**Gandhi** considered the proposed new Note 1 not to be controversial. He reported that since at least 1990, the *Gray Index* had been using terms like isolectotype and isoneotype, but their eligibility had been questioned as such terms were not in the *Code*. He noted that in Art. 9.3 there was isotype, and in Art. 9.10 there was isosyntype, but not terms like isoepitype, isoneotype, or isolectotype. If this Note was added he felt there would not be a problem in future.

**McNeill** explained that the intention of the proposal was to add these terms into the *Code*.

**Davidse** strongly endorsed the proposal since the terms were very widespread in the botanical taxonomic literature.

**Watson** wondered if this gave the isolectotype status over the other syntypes if the lectotype was destroyed.

**Barrie** explained that currently duplicates of a lectotype did not have status over syntypes, unless the isolectotype already was a syntype. There could be problems if there was a mixed collection and the lectotype element was the only one that belonged to the element to which the name had been applied. In that case one might want to switch to a syntype, and if isolectotypes ended up with a status higher than other available syntypes one might end up with situations where a conservation proposal was needed, which would not be the case currently.

**McNeill** pointed out that there was nothing in the proposal effecting any change in the status of the terms. This then raised the question as to why definitions which had no nomenclatural significance should be put in the *Code.* It was appropriate that these terms be defined clearly somewhere, but he wondered if they should be in *Code* if they did not have a distinctive nomenclatural status over other specimens? If they were included some might think they had status under the *Code*, so caution was needed.

**Barrie** said the terms were used informally and he did not think the Section would wish to grant them any status. The prefix “iso-” made it clear what the terms meant.

**Nic Lughadha** wished to see these terms in the *Code* because people used them and expected to find them, but would not like to see them in a Note that would have some binding effect. She would not vote for definitions to be included until she saw the exact wording. Perhaps definitions could be drafted by the Editorial Committee as Recommendations?

**Redhead** wondered if a statement should be added to indicate that the use of “iso-” did not change their status.

**McNeill** indicated that the view of the Editorial Committee was that what was in the *Code* was what needed to be, and if this were left to the Editorial Committee the Note would not be included. They belonged in a glossary, not the Glossary in the *Code*, but a broader glossary or a book explaining nomenclatural procedure would be excellent places for such terms.

**Wieringa** was in favour of the proposal, for as soon as the terms were in the *Code* there would no longer be an obstacle to their use.

**Turland** made the point that just because a term was not in the *Code*, that did not mean that its usage was incorrect.

**Demoulin** felt that if there was a vote to Editorial Committee, it should be possible to have a Note to say that the prefix “iso-” could be added to any kind of type to indicate the existence of a duplicate, but that only isotype had a status regulated by the *Code*. [Applause.]

**Hawksworth** pointed out that of the approximately 1100 terms in the draft glossary of terms used in bionomenclature he had prepared, he estimated that about 300 had the suffix “-type”, which were used to varying degrees. To add such definitions to the *Code* could be the start of a road that would have no end.

**Gandhi’s Proposal** was referred to the **Editorial Committee.**

**McNeill** moved to consider the other two new proposals relating to Art. 9.5 that were overlapping.

**Brummitt** explained that about 25 years ago there was a paper in *Taxon* proposing a new term in botanical nomenclature, “paralectotype”. He had replied to it saying that this should be “lectoparatype” not “paralectotype”, and there had been a grotesque sequence of papers on the subject which he hoped the Section would not get into. The proposal was not accepted and never put into the *Code* because it was thought to be superfluous. He felt the present proposal should be dismissed and that long arguments should not be entered into.

**Barrie** agreed as this would cause more confusion. If a lectotype was being selected from among syntypes, the syntypes remained syntypes and did not change to a different status. It was much clearer the way it was.

**Tronchet**, the author of one of the proposals, did not agree. When he saw syntypes he felt there was a need for a lectotype, but if he saw paralectotype or lectoparatype it was clear that a lectotype had already been chosen.

**Gandhi**, the author of the other, was after an opinion on the status of the residue of syntypes. He had been asked this 19 years ago and did not know what to say or what to call the remaining syntypes after a lectotype had been chosen.

**McNeill** pointed out that they remained syntypes as far as their status under the *Code* was concerned.

**Gandhi** did not think this was clear from the *Code*. He had asked Nicolson at the time, and he also indicated that he did not know what term to use. A clarification in the *Code* would therefore be quite useful.

**Ahti** wished to point out that in Art. 9.5 Note 3 there was a sentence stating that when an author designated two or more specimens as types any remaining cited specimens were paratypes and not syntypes.

**McNeill** explained that that Note referred to a different situation.

**Brummitt** added that that was why they should be called lectoparatypes and not paralectotypes. The term lectoparatypes was already well-established in the literature.

**Glen** agreed with Brummitt and Barrie that this proposal could be reduced to total absurdity by considering a duplicate of one of the unchosen syntypes as something like an isoparalectotype, and after that you would need physiotherapy on your tongue!

**McNeill** suggested the two proposals were voted on together as they had the same thrust and any discrepancy could then be dealt with editorially. One introduced the idea and the other spelled it out.

**Tan** was curious about the proposal to change the term paralectotype to lectoparatype and wondered if the Section was to vote on that.

**McNeill** thought that if the proposals were passed, the more appropriate term would be chosen editorially, and explained that the two proposals dealt with the same issue; that from Tronchet was more detailed than that from Gandhi, but he did not think they were in conflict.

**Nicolson**, after calling for the vote, announced that the proposals from **Gandhi** and **Tranchet** had failed.

[*Here the record reverts to the actual sequence of events*.]

### Recommendation 9C (new)

**Prop. A** (11: 139: 4: 4) was ruled as rejected.

### Article 11

**Prop. A** (34: 24: *95: 3) & **Prop. B** (35: 25: *94: 3).

**McNeill** introduced Art. 11, Props A and B, and noted that there was a special meaning attached to the “ed.c.” vote, which was the majority in both cases.

**Moore** had already talked to Turland about it and was in favour of the amendment that the Rapporteurs had suggested. He added some background on the proposal, noting that it came up in the Committee for Spermatophyta but had also come up in conversation with other people. He explained that the proposal was trying to make it clear that Art. 11 was only dealing with cases of synonymy and not dealing with cases of homonymy.

**McNeill** felt it was simply a matter of where it was put as he felt that the suggested wording was established by the Rapporteurs. There could be no suggestion that describing a new taxon or publishing a new name of a taxon of recent plants could somehow make invalid an earlier published name of a fossil plant. The present wording could be misinterpreted quite readily that way and they thought that putting something in to clarify it would be a good thing. The proposer had accepted the suggestion made by the Rapporteurs on page 220 of the Rapporteurs’ comments [i.e. in Taxon 54: 220. 2005].

**Nicolson** thought the proposal was to refer these to the Editorial Committee...

**McNeill** interrupted and disagreed, clarifying that the proposal was that instead of the precise wording that appeared, it should be the wording that appeared on page 220 of the Synopsis of Proposals, which said that “The provisions of Article 11 determine priority between different names applicable to the same taxon; they do not concern homonymy which is governed by Article 53, and which establishes that later homonyms are illegitimate regardless of whether the type is fossil or non-fossil”.

**Turland** asked the proposer, Moore, if he had any comments on what was on the screen, if he had any refinements to that or if that was what he wanted the Section to vote on?

**Moore** agreed that it looked fine.

**Rijckevorsel** pointed out that as it was placed [on the screen] it was an inclusion in Art. 11.7 and he had understood it was to be a Note.

**Turland** apologized and agreed it should be a Note.

**McNeill** also agreed that it was definitely a Note. He added that which part of Art. 11 it went in would obviously be determined by the Editorial Committee.

**Prop. A** was **accepted** as amended.

**McNeill** took it that Art. 11, Prop. B would be treated in exactly the same way because they were just dealing with the different levels in the Article so it was covered by exactly the same proposal.

**Prop. B** was **accepted** as amended.

**Prop. C** (89: 12: 53: 2).

**McNeill** introduced Prop. C and noted that it comprised two Examples.

**Nicolson** noted that the *Ficus* Example was in the conservation proposal.

**Turland** asked what the Permanent Committee had decided on that?

**McNeill** thought it [acceptance of the conservation proposal] had been recommended by both Permanent Committees, so the Editorial Committee would have to take account of that in producing a different Example.

**Skog** stated that this meant the Section could not even vote on it any more.

**McNeill** agreed that it just dropped because it was no longer an Example because by conservation it had been altered. He thought it may be possible to use a wording that still made sense. He thought the *Endolepis* Example was okay.

**Turland** clarified that what was being voted on was Art. 11, Prop. C, the *Endolepis* Example. He noted that the second Example was no longer relevant and mentioned that the Editorial Committee could find another Example at its discretion.

**Barrie** had a question about how the vote was formed, so that he understood exactly what he was going to be voting for. What concerned him was that he thought that what was being proposed was that these be referred to the Editorial Committee rather than included in the *Code* as a voted Example?

**McNeill** agreed that was definitely the case, they were referred to the Editorial Committee; they were not voted Examples.

**Barrie** suggested that when voting on these things with Examples in them it was important to be clear on what was being done, because he was concerned about adding voted Examples unintentionally.

**McNeill** noted that, to his knowledge, the Section had not voted on a single Example and that was the point that was raised earlier by somebody: how do we know we are referring something to the Editorial Committee? He felt that this particular proposal should definitely be a reference to the Editorial Committee, whether to take it into account or not. He added a summary for the benefit of less experienced people about the phrase “voted Example”. He explained that there were in the *Code* a number of Examples which were prefixed with an asterisk and these were termed voted Examples. This meant they were Examples which did not necessarily or did not clearly exemplify a particular Article, but nevertheless they had been decided by the Section as things that should be entrenched in the *Code* rather than trying to fiddle with the wording of the Article because that might create more problems than it solved. So from time to time Sections had taken a particular Example and voted on it, even recognizing that it was not clear that that was what the *Code* ruled. These were Examples that the Editorial Committee could not touch. They may improve the language a little but these things could not be removed. All other Examples in the *Code* were just that, Examples. The Editorial Committee could put in a better one if it knew of one, or it was obligated to take one out if it no longer exemplified the Article. But voted Examples had a status of their own that equated to that of an Article. So the point that Barrie was making was that we should not inadvertently vote on an Example. He emphasized that that was why it was very important when these things were merely Examples that they be referred to the Editorial Committee for appropriate action. Obviously then the Section was commending them to the Editorial Committee and suggesting they take them up, whereas in other cases the Editorial Committee might obtain an Example from anywhere. He concluded that this was a proposal that could be referred to the Editorial Committee.

**Prop. C** was referred to the **Editorial Committee.**

**Prop. D** (55: 22: 35: 30).

**McNeill** noted that the next two proposals also dealt with Examples that particularly applied to one of the recently adopted rules relating to the nomenclature of fossil plants. He invited Judy Skog from the Committee for Fossil Plants to comment on the two proposals intended to clarify the implementation of the morphotaxon concept.

**Skog** outlined that the fossil plant Committee had had a lot of discussion about the two Examples. Most of the discussion revolved around the fact that the Examples seemed to really be more or less a taxonomic decision rather than a nomenclatural decision. Whether you use *Ginkgo* or *Ginkgoites*, it seemed to them, was up to the person doing the description. But they had no problem with them going to the Editorial Committee and having the Editorial Committee decide if it really did clarify the situation. Many of the members of the Committee felt that Prop. D was too restrictive and that the Example in terms of restricting the the use of a genus that has at times been considered an example of a whole plant fossil, in other words not necessarily confined to a morphotaxon, could restrict fossil nomenclature. She concluded that the fossil plant Committee had no problems with Prop. E going to Editorial Committee but they would prefer not to see Prop. D proceed.

**Zijlstra** had a problem with the wording. It said that the leaf morphospecies *Sphenopteris hoeninghausii* could not be placed in the stem morphogenus *Lyginopteris*. She argued that it could, it could be considered as incorrect but it could, so she considered the proposal to be nonsense.

**Skog** said, Thank you! [Laughter.]

**McNeill** thought it sounded as though it would need editorial attention. He thought the point behind it, which had quite important significance beyond those of Examples, was that he was not altogether convinced that all palaeobotanists appreciated the significance of what had been adopted on their behalf in St Louis. He thought that the proposals were intended to emphasize that, because one of the things that was clear in practice was that *de facto* all fossil taxa were morphotaxa which he did not think was what all palaeontologists wanted, but nomenclaturally they had to be treated as such, according to what was in the *Code*. He saw that Skog was shaking her head so maybe this was a little more than just a matter for the Editorial Committee. He noted that for purposes of priority the name of a fossil taxon could only be applied to a morph corresponding to the type. He added that was the reason why it was only a Note that said that any name based on a recent taxon automatically took precedence, because the- type of a fossil taxon name could not apply to the name of a whole organism, according to the wording that was accepted in St Louis. He read out Art. 11.7: “For purposes of priority, names of fossil taxon (diatoms excepted) compete only with names based on a fossil type representing the same part, life history stage or preservational state”. He concluded that it was what that meant that the Examples were intended to develop, rightly or wrongly.

**Skog** agreed that that was what the proposal was intended to achieve. She had gone back through all of her notes from St Louis where there were a variety of terms floating around such as parataxa, form taxa, fossil taxa, et cetera et cetera, to convey the old idea of a form genus. There were a number of wordings that were put forth, some of which had the term “fossil taxa” in them, some of which had the words “parataxa”, some of which had the term “form taxa” in them. Dr Faegri came up with the term “morphotaxon”, which seemed to solve much of the difficulties. She believed when it said “fossil taxa” in 11.7, it was really referring to fossil morphotaxa, not all fossil taxa. She just thought that the “morpho-” somehow slipped off the radar screen.

**McNeill** responded that that was not what it said and added that he felt it had affected other parts of the *Code* because he was afraid the Editorial Committee at St Louis did implement that in changing what had been an Article to a Note. He continued that what was now Note 4 was only a Note because of Art. 11.7, because it could not compete with the name of a recent organism which was by definition that of a whole organism, not of a preservational state. He thought that the topic was probably something that was not appropriate for further discussion within the Section, although the particular proposals should be addressed.

**Demoulin** was convinced it must not be a voted Example but still thought it should be considered at the Editorial Committee. He asked Skog to explain again what it could illustrate in the situation. He felt that it was not possible to simply wipe the problem of *Lyginopteris* under the carpet, if there was a problem of interpretation in this case he argued that it should be addressed. He suggested that it may make a good Example, perhaps not the way it was phrased, but it should be decided what the *Code* really said regarding the issue. He concluded that it should be referred to the Editorial Committee.

**McNeill** summarized that the suggestion, both from Skog and supported by Demoulin, was that Prop. D be referred to the Editorial Committee.

**Bhattacharyya** felt that “widely believed” was an ambiguous term. He gave the example that some people used to widely believe that the sun moved around the earth but others did not. He believed that an ambiguous Example would mislead the situation and the aim of the *Code*.

**Nicolson** mentioned that Skog was on the Editorial Committee and he hoped she would continue to be there along with Demoulin, so there was a chance that there would be further discussion if it was referred to the Editorial Committee.

**Prop. D** was referred to the **Editorial Committee.**

**Prop. E** (69: 9: 30: 35) was referred to the **Editorial Committee.**

**Prop. F** (98: 44: 10: 1) was **accepted.**

[*Skog’s Proposal to alter “taxa” in Art. 11.7 to “morphotaxa” was accepted along with the vote on her motion regarding Art. 1.2 – see above*.]

### Article 13

**Prop. A** (107: 22: 8: 13).

**McNeill** introduced two proposals that he described as interlinked. He noted that they stemmed from the situation in which in preparing the first Names In Current Use list, although it was not called that in those days – the list of conserved names of families that was adopted at the Montreal Congress [the current App. IIB], the working basis for producing the list was the adoption of Jussieu’s *Genera Plantarum* in 1789 as the starting -point. In fact that was never enshrined in the text of the *Code*, so that when Reveal and others prepared lists of family names they began to raise questions as to the status of names that were earlier than 1789 and it was then proposed that the 1789 starting date go into the text of the *Code*. This was not accepted in Tokyo, partly because it was dealing with all family names, not merely those of spermatophytes. Eventually as a result of the decision in St Louis it had to be dropped, as the Congress would not accept 1789 at that point. However it appeared that that was not fully understood by everyone who was there and so there had been some concern to put 1789 back. That was one of the things that the Committee for Suprageneric Names addressed. So he summarized that the suggestion was that the starting-point for family names be changed to 1789, in the case of Art. 13, Prop. A for all suprageneric names, but applying to all groups and that, in the case of Prop. B, that would not include the Pteridophyta. He suggested discussion should start with Art. 13, Prop. A, which received substantial support in the mail vote: 107 in favour, 22 against, 8 Editorial Committee and 13 Special Committee.

**Brummitt** concurred that there was a lot of misunderstanding about this and in his opinion it was a complete accident that 1789 was ever deleted. As Secretary of the Committee which had to deal with family names of flowering plants, he very strongly recommended that the Section go back to 1789 as the starting-point, which he thought would eliminate a lot of potential problems.

**Mabberley** was against the proposal, although he generally agreed with everything Brummitt said. He felt that there were enough dates around as it was. He pointed out that there had been a black book with the family names in question with the earlier dates in and as far as he knew nobody had died as a result. He was interested to know how damaging continuing that would be, as according to Brummitt there were other problems. He felt that changing back and forth was what gave the *Code* a bad name.

**K. Wilson** wanted to actually clarify in the first place what the Committee for Pteridophyta thought, because she felt that had a big bearing on whether to vote “yes” or “no” for Props A or B.

**McNeill** thought that logically if Prop. A was passed an amendment could be proposed to Prop. B that removed “Pteridophyta” and if A was defeated, then the matter would fall. He thought that the Pteridophyte Committee had said that it was divided on the matter and really did not feel strongly; the members were lukewarm about the changes but did not mind whether pteridophytes were included or not.

**Barrie** wished to respond to Mabberley’s comment because he and Turland were the people who looked at the original list from Reveal to decide which ones would go into the *St Louis Code* and which ones should wait for more investigation. He pointed out that the only pre-1789 names introduced into the *Code* Appendix were Adanson’s, but that there was a whole list of other authors for which there were issues about whether or not they were actually referring to families or not in the current sense of the term. He believed that this Committee for Suprageneric Names had spent a long time debating whether or not they be introduced into the Appendix – and they had not yet and so adding the starting-point now really meant taking out Adanson’s names and going back to probably Jussieu as the author for those names. He did not think there were any names that would actually change, just the references.

**Voice:** “What about mosses”

**Zijlstra** reported that the Committee for Bryophyta had expressed the view that they were not against the proposal but they had no cases.

**McNeill** reiterated that that was why the Committee for Bryophyta had no particular position, as there were no family names in *Bryophyta* affected.

**Buck** pointed out that the proposal was to set the Jussieu date for spermatophytes, pteridophytes, and *Sphagnaceae* and *Hepaticae*. But wondered if there were no cases in *Sphagnaceae* and *Hepaticae*; why were they being included?

**Watson** clarified that they were explicitly excluded because at the time it was being put together the Committee for Bryophyta rejected the proposals.

**McNeill** felt there was no reason for not having the starting date for all suprageneric names in all groups. He thought that the point was that with the way the wording of Art. 11 was at the moment, the starting date for mosses was different from that of the other groups, being Hedwig 1801 rather than Linnaeus 1753, mosses just dropped out.

**Demoulin** had never been very much involved in suprageneric nomenclature so was not really decided on the proposal. But he had been very much involved in the later starting-point issue and was afraid to see a new one introduced. He wished to draw attention to the thing that was worked on for a long time before the Sydney Congress. The problem of later starting-point is to find out the first publication after the starting date. He argued that even if there may be problems with the Reveal list, it existed and asked if anyone could tell him of a list of what should be taken up after 1789, if that date was chosen? He also asked for the opinion of Silva who he thought was also worried by the later starting-point but had experience with suprageneric nomenclature.

**Nicolson** asked Silva if he would be prepared to make a statement about the impact of going back to the 1789 date for suprageneric nomenclature and its effect on algae?

Before Silva spoke, **McNeill** wished to point out that the present wording only applied to clauses (a) and (c) of Art. 13, i.e. *Spermatophyta*, and *Pteridophyta*, and the *Sphagnaceae* and *Hepaticae*. He added that it did not affect algae at all, algae would stay at 1753, and the point that Buck made was probably a very valid one, that it would be adding a meaningless but totally innocuous statement in (c). The starting-point for suprageneric names of *Sphagnaceae* and *Hepaticae* could stay at 1 May 1753 if there were no family names – or rather no suprageneric names involved. He felt it just simplified the wording.

**Silva** thought there was only one family name that would be affected and that was *Fucaceae* itself, because up to about 1810 the algae were all considered to belong to one family.

**McNeill** noted that as he had just said, *Fucaceae* was not affected because the proposal was not in fact changing the date for algae.

**Buck** was concerned that in hepatics that meant any family name between Linnaeus and 1789 would just be thrown out, even though there were none in 1789.

**McNeill** noted that they could not be thrown out if there were none.

**Buck** clarified that he was saying that any ones that somebody else may have described between Linnaeus and Jussieu, between 1753 and 1789 would no longer be able to be used.

**McNeill** thought he must have misunderstood what Zijlstra had said, he had assumed from her report...

**Buck** interrupted that what he thought she said was that there were no family names in Jussieu.

**McNeill** had assumed she meant there were none published before 1789.

**Buck** had not understood that.

**McNeill** added that if it was the case then it did mean that the addition to paragraph (c) was pointless, which was what he thought was the point Buck was making.

**Buck** continued that if she actually meant there were none in Jussieu, what that meant was that any pre-Jussieu would be thrown out.

**McNeill** thought that the quickest thing was to turn to Zijlstra and to see what she meant. He asked her if there were any suprageneric names published in the hepatics or *Sphagnaceae* prior to 1789?

**Zijlstra** responded that as far as they knew, no, nothing, they did not have cases.

**McNeill** took the point as being substantively editorial: Why clutter up the *Code* with an exception clause that is meaningless? He suggested that unless the Section disagreed, that would be an editorial decision that would be taken on the advice that had been given.

**Turland** raised the other point that more than one person had mentioned that the proposal was to introduce a new starting-point. He thought that was really not the case, instead the proposal was to reinstate a starting-point which effectively existed right up until the St Louis Congress, when it was removed. He felt that what Art. 13, Prop. A would do was exactly what was afforded by the App. IIB, the introduction to that, and the Art. 14 footnote which existed in the *Tokyo Code*. So that suprageneric names of bryophytes and spermatophytes would effectively have a starting-point of 1789, Jussieu.

**Prop. A** was **accepted.**

**McNeill** thought that regarding Art. 13, Prop. B, unless someone wished to move that the pteridophytes be excluded, it would be ruled as being implicitly covered by Prop. A. So it need not be discussed unless someone wished to propose that the pteridophytes be excluded.

**Turland** pointed out that Prop. B was contingent on Prop. A being defeated.

**Prop. B** (65: 56: 10: 13) was ruled as **rejected.**

**Prop. C** (40: 24: 15: 42).

**McNeill** introduced Prop. C, which he described as being on a rather different topic dealing with starting dates, the later starting date of “*Nostocaceae homocysteae*” and “*Nostocaceae heterocysteae*”. He reported that the Committee on Algae had commented on the proposal and he thought there were differing views from the proposer, Silva, and the Committee of Algae. He wondered if there was someone from the Committee other than Silva who wanted to speak to it.

**Silva** mentioned that the algal Committee did not support his proposal and the opposition came mainly from one member, L. Hoffmann, who had alternatively suggested a Special Committee that would engage the interest of the microbiological people, who treat the blue-green prokaryotes in a very different way, they call them cyanobacteria, we call them *Cyanophyta*. His feeling was that the two groups of people would always do their research in a different way. The ecologists, for instance, like names on the things that they could describe and the microbiologists insist upon having things worked out in culture. He thought that Hoffmann’s proposal for a Special Committee was certainly acceptable. He believed that eventually it may come back to his proposal to eliminate the later starting-point for the blue-green algae.

**Demoulin** wished to raise the issue again from the floor as he felt that the Section could not decide on something like that without access to the Committee report and the votes.

**McNeill** explained that that Special Committee had not been set up, the matter would come on the table later in the sessions. He clarified that there was a proposal from the Committee for Algae that a special joint Committee be established from this body and from the body responsible for what is now called the *Prokaryotic Code*.

**Demoulin** continued that for the problem with the fossil Committee, a report of the votes was given and there were a lot of abstentions. He felt that the Section needed to know if in the algal Committee there was such a vote. He knew the issue quite well, but he was the one that pushed at the Sydney Congress so that there was a clause that allowed the use of some of the names if they were validly published under the *Bacteriological Code*. He thought that this was still an important part of the system. He disagreed entirely that there was a need for further discussion and Special Committees and things like that. He argued that it would facilitate the agreement with the bacteriologists if the Section followed Silva’s proposal, one that was founded on long experience. He added that Silva had anticipated and was already published using the 1753 starting-point. In his opinion, things would be much easier and clearer for coming to an agreement with the bacteriologists. He had discussed this with Oren, who was an experienced member of the bacteriological Committee and they agreed. He thought that making more and more committees was not really what would lead to advancement. He referred to his experience of the orthographic Committee. He felt that it was a good proposal with about as many people in favour of it as there were people wishing to refer it to the Special Committee. If half of the people were in favour of the proposal, he felt it was not possible to consider that there was a majority in favour of the proposal.

**McNeill** noted that the report of the Committee had already been published, which was why he did not think it necessary to give the details, but of course it was published in the May *Taxon* which many people may not have seen. He read the relevant portion of the report for Prop. 222, under discussion, “The later starting-points for some groups of algae have been challenged for a long time but it is not sure that cancelling would be more advantageous than maintaining these later starting-points. The present proposal deals only with the two groups of *Cyanophyta* at a moment when the International Association for Cyanophyte Research is trying, with the help of members working in both the botanical and the bacteriological fields, to harmonize cyanophyte nomenclature under the botanical and the bacteriological Codes. Rather than trying to amend the botanical Code there is a question if it would be wiser to work in collaboration with specialists of bacterial nomenclature to arrive at a common system for the *Cyanophyta*/*Cyanobacteria*”. The Committee did not support the proposal and the vote was 1 in favour, 10 against and 3 abstentions. He concluded that it was pretty emphatic, which was why it had been withdrawn.

**Demoulin** felt that it should not have been that way because the discussion of the Association on Cyanophyte Research. He had the manuscript that Lucien Hoffmann had edited and he thought it explained his action which was laudable, but most of the important things had been done during a discussion organized in Luxembourg. He emphasized that there was no need for more talks. In addition he alluded to all the things that were important to do, but pointed out that most of those things must be done by the bacteriologists. He felt that suppressing the later starting-point made things clearer and easier for the discussion with them, because then we only needed to eventually decide what to do with the list the bacteriologists made, which he suggested was the role of the Special Committee.

**McNeill** felt that Demoulin was straying from the proposal that was no longer even on the floor, having been withdrawn. He thought he should hold his fire on how the procedure should go forward until a proposal to have a joint committee arose. But he thought some relevant points had been made and thanked him.

**Prop. C** was **withdrawn.**

### Article 14

**Prop. A** (70: 78: 3: 1).

**McNeill** moved on to Art. 14, Props A & B. There had been a friendly amendment suggested that would subsume both proposals by proposing to extend conservation to “the ranks of family and below” and he invited Dr Brummitt or Dr Lughadha to speak to this amendment.

**Brummitt** observed that it was possible to conserve names of families, genera and species and to reject any name at all. The difference between the two approaches was, in his opinion, purely accidental and historical, the way the wording had got into the *Code*. The two proposals by Hawksworth were to introduce conservation for infrageneric names and infraspecific names. He pointed out that, on the page in *Taxon* where they were published, there was also another Article, apparently quite coincidentally, by Rijckevorsel about names at infrafamilial rank. He thought that the Section would be glad to know that it was a proposal to make the wording of the *Code* simpler, simply to extend conservation to names at any rank – at family and below. He added that above family there was no need to involve conservation because they had no priority anyway. He acknowledged that of course some people would say, “Well, this will open the floodgates and we’ll have endless proposals”, but he did not think that was going to happen. He pointed out that people had threatened that the floodgates would open for the last 30 years and they had coped with conservation of species names. He did not think many cases were going to come up at the intermediate ranks. He advocated the need for the facility to adopt the proposal, the procedures at those ranks, if and when they came up. He quoted a case, he hoped with permission from Rijckevorsel, who had written about it. The family hitherto *Epacridaceae*, which all the Australians would know all about, had recently been sunk by many people into the *Ericaceae*. One would think that it had to be called the *Epacridiodeae*, which would bring a measure of continuity between the names, but in fact it had to be called *Styphelioideae* on the principle of priority. He reiterated that the facility was needed when strange cases like this came up to do something about it. He had spoken to one or two of the members of the Committee for Spermatophyta, who were the people likely to get the work and nobody seemed terribly worried about it, they did not think it was going to be a terrible amount of additional work and he could not see any reason really why it was not possible to simply open up conservation to anything at all. He argued that then the Committee could just pick and choose which names were considered well-known enough to attract the Committee’s attention. He thought that there was agreement that if the proposal were to go through, a considerable number of proposals under Art. 19, which were coming up, would hopefully be made irrelevant.

**Rijckevorsel** explained that he had made a great deal of proposals from an editorial perspective. He felt that by making proposals you should either make editorial proposals or policy proposals, so he tried to stay away as far as possible from any policy decision as possible. Nevertheless, he felt this was an issue which needed to be addressed, so he put in this other proposal that was technically very good, if he said so himself. [Laughter.] He thought that it would have a minimum of nomenclature impact so it would change as little as possible because he did not want to make the proposals from a general perspective. He was not really going to speak in favour of it because he did not really have any strong feelings about it, but he was certainly not against it. He added that it would be simpler in terms of phrasing and simpler to understand. Regarding the nomenclature impacts, he did not know if it would achieve a similar effect. He noted that there were a few bad cases, besides the case in *Taxon* there was also a very famous case of the subfamily of the apples, *Maloideae*, which was a terrible problem for everybody who worked with apples because he believed that subfamily did not exist and that would be solved by the proposals.

**Hawksworth** endorsed and confirmed that he accepted the friendly amendment. He thought this was a logical extension to the powers of the Committees when they wished to use them. He had come across a particular case last year involving a name where it would have been very nice to conserve a particular varietal name with a conserved type, which was not possible under the rules. It just seemed illogical to have to make a totally different argument, which in fact did go through the Committee, but was much more convoluted and it would have been much neater for the Committee to be able to handle a varietal name in that case.

**McNeill** interjected that the proposal was not to keep the varietal name, it was an elegant way to save making two separate conservation and rejection proposals that were dealing with names at the level of species.

**Hawksworth** agreed that was correct. He explained that it started off as a varietal name, which was the problem, and then was used at species rank. He concluded that the proposal would give that extra flexibility to the Committees.

**Nic Lughadha** supported what Hawksworth had said. She thought that there were cases where what was needed to save the name of a species in commerce – for instance, a carnivorous plant – was actually to conserve the name at varietal level which was not possible and ended up in very convoluted work-arounds. Equally, she suggested that, as the legume people would all be familiar, their systematists were very often focused at tribal level, and they would like to be in a position to conserve some of their tribal names. She pointed out that there were named working groups that sometimes had to change their names and things like that. So she felt there were a small number of cases and of course the same criteria would apply that have always applied. She added that there would need to be a strong case for conservation but that was not always just at family, genus and species.

**Gandhi** also supported the friendly amendment. He wished to add that at least for infrafamilial names there was a major index available on the Web and maintained by Reveal at the University of Maryland. Regarding infrageneric names and infraspecific names, his concern was that there were no major indices available. He did not know whether it was a relevant fact that when conserving an infraspecific name or an infrageneric name, it was unclear how the botanical community would know what the other available competing names were or how widely they were used.

**Friis** reported that both Copenhagen and Aarhus supported a more flexible system for these cases, preferring the model rather than having to choose a convoluted way of rejecting names instead.

**Bhattacharya** was worried that many temperate herbarium botanists without tropical field experience would use wishful species conservation. He felt that adding the word “taxa” would open the floodgates.

**Wiersema** did not necessarily share the feeling that it was not going to lead to additional proposals to be dealt with. As someone who edited the proposals for *Taxon*, he thought that they would receive a substantial or a reasonably substantial number of additional proposals because of it. Especially at infraspecific ranks, where there had not been total indexing over the years and there were many names in use that probably did not have priority, he knew of a number. He felt that there would be attempts to salvage the names in use and there would be a number of instances which would have to be dealt with this way. He thought that the other mechanism that was proposed, under Art. 19, for dealing with some of the subfamilial cases should not be thrown out the window if that would avoid some cases having to result in proposals. If it was possible to solve some of the problems without proposals he thought it should be considered, rather than relying solely on this mechanism for solving those cases.

**Gereau** felt that the real issue was not the number of proposals or the extra work that would be created, but rather one of basic principle. He asked whether the Section wanted to apply a set of basic principles that all could understand and use, or move further and further into the world of special legislation for every case? He submitted that the *Code* as it stood had already moved too far into the world of special legislation and that it was inadvisable to tinker with it further in this way. He added that the original justification and the one that had been constantly put forth for stability of names was the outside community; agronomists would not like it, the weed people would not like it. He felt that the Section had a public to serve and he could agree with a lot of those concerns. He felt that that public had no concern whatsoever with names at ranks other than families, genera and species. He suggested that if it was being done strictly for internal reasons, then it should be labelled so. He concluded that the justification fell far short of desirability.

**Demoulin** thought that the lack of indexing for several of the categories was a strong argument for the proposal (as amended), because of the lack of indexing, he felt there was a bigger problem with instability in the future. He thought the very simple and clear amendment would make things much easier, even if there were a few more proposals to deal with. He added that the problem of the number of proposals was very much a cultural problem of some groups who made more proposals than others and he did not think it was related to the ranks at which it was possible to conserve.

**Pedley** felt that the *Code* was going too far down the road of conservation of existing names. He had no problem at all with *Styphelioideae* instead of *Epacridoideae*, although he didn’t know who used them. He did not think that the field needed to be widened any further.

**Hawksworth** pointed out that the number of proposals for the Committees would not necessarily change in the cases like subspecies, because it was already possible to propose one that had been rediscovered for rejection.

**Nicolson** moved to a vote on Prop. A with the friendly amendment. His response to the outcome of the show of hands was, “Oh dear”. [Laughter.] He then moved to a show of cards reminding the Section that white voting cards indicated one vote and everybody present got one vote, a green card was two votes, a yellow card indicated three votes and red cards were five votes. He told the Section that he would try to look at what he saw and asked everyone to wish him luck. [Laughter.] His response to the outcome of the show of cards was, “Oh”.

An **Unknown Speaker** called for a card vote.

**McNeill** clarified that on the ballot, number one would be used, but just to avoid any possibility of error, it would be appreciated if “yes” or “no” was written on the number one that was detached.

The **amendment** was **rejected** as amended on a card vote (220: 210, 51.2%).

[*The following debate, following on from Art. 14 Prop. A took place later that afternoon, i.e. during the Second Session on Tuesday*.]

**McNeill** felt it would be perfectly in order, should he so wish, for the proposer of the original proposal to determine if either of the proposals were worthy of further consideration. He explained that the proposal that the Section failed to accept was to extend conservation to all ranks and the original proposals were to deal with infraspecific ranks and the second one was ranks of subdivision of genera.

**Hawksworth** thought it was certainly worth looking at, because he thought there were many cases that would come to light around the species level in particular. He suggested that it may be the genus and family additions which were causing the Section concerns so it would be good to get a feeling.

**McNeill** noted that there had already been quite a bit of discussion. He highlighted that it was solely the issue of names below the rank of family being looked at now, and of course it was possible to reject at that level, where he thought it was perfectly clear that proposals for conservation would be strictly as a mechanism of saving a species name. He did not believe below that level that there would be any case that a Committee could look at seriously that would involve disadvantageous nomenclatural change, if so they would be certainly unusual. He summarized that it would be strictly in order to use the mechanism of conservation at a level below that of species in order to conserve names of species or perhaps some vitally important subspecies. He clarified that the vote was on Art. 14, the original Prop. A.

**Landrum** suggested asking how many people would change their vote as he thought that might make things go faster.

**Demoulin** disagreed with the question and did not think it was the same thing at all. He suggested that one may wish to have Prop. A, because certainly those with experience with working with the Special Committees knew that the case existed. He felt that it would probably be something that made their work easier than the fact that we have a few more proposals. But he added that one may also consider that Prop. B was less useful, less necessary, because it was not saving a very important name. Personally, he would vote for Prop. A and abstain or perhaps vote no on Prop. B. He maintained that it had nothing to do with the previous general vote.

**Nicolson** asked how many were in favour of Art. 14, Prop. A, then how many opposed and arrived at the same problem. He moved to a show of cards. He thought it was too close and ruled that it did not pass. He then acknowledged two requests for a card vote.

**McNeill** instructed the Section that it would be card vote number two and as before, it would helpful to ensure no mistakes that “yes” or “no” were written on the paper.

**Prop. A** was **rejected** on a card vote (224: 213, 51.3%).

**Prop. B** (57: 82: 3: 2) was **withdrawn.**

**Prop. C** (83: 22: 48: 1).

**McNeill** moved onto Art. 14, Prop. C, an Example, which he reported had received a fairly positive vote in favour.

**Rijckevorsel** felt that it was a very simple editorial mishap that really did not deserve much treatment, so it should simply be corrected. He added that he would also like to speak to the other two proposals, 14D and Rec. 14A, saying that they had been wildly unpopular so he was not going to say anything about them. [Laughter.]

**Barrie** felt that it was a good proposal but completely editorial so suggested referring it to the Editorial Committee.

**Prop. C** was referred to the **Editorial Committee.**

**Prop. D** (3: 137: 10: 4) was ruled as **rejected.**

### Recommendation 14A

**Prop. A** (28: 30: *96: 2).

**McNeill** introduced Rec. 14A, Prop. A where the Rapporteurs had made a suggestion of a slight change of wording. They thought the thrust and intent of the proposal was good but did not think that the suggested wording was as clear as theirs, which was for the Section to determine. In the Recommendation they suggested adding “usage of names”, which they thought would clarify it. The point that they wanted to focus on was that usage of names should not change, not that one particular type that proved to be technically correct should be preserved even though it was disruptive. He asked if Brummitt accepted that as a friendly amendment. [He did.]

**Nicolson** suggested referring it to the Editorial Committee.

**McNeill** thought it should be voted on because the Editorial Committee vote had the special meaning of applying the Rapporteurs’ wording.

**Woodland** wondered if it meant that the author should refrain from making any changes and follow existing usage until the decision had been made no matter now long it took for the Committee to rule on a proposal.

**Nicolson** confirmed this as pending.

**McNeill** hoped that it could not take more than four years and added that normally the General Committee was a little quicker than that. From the time of the initial proposal, he estimated that the process through the General Committee usually took about a couple of years.

**Prop. A** was **accepted** as amended.

**Prop. B** (2: 148: 5: 0) was ruled as **rejected.**

### Article 16

**Prop. A** (128: 10: 8: 1) was **accepted.**

**Prop. B** (40: 99: 9: 4).

**McNeill** introduced Art. 16 Prop. B and reported that the mail vote was somewhat negative. He noted that it was a proposal originally from the Committee on Suprageneric Names.

**Nicolson** added that it was dealing with names above the rank of family.

**McNeill** explained that it was essentially restricting the use of descriptive names, which were quite widespread but a minority.

**Barrie** pointed out that the proposal was dealing with names that had no priority. Therefore he felt that ruling on them was in some ways pretty meaningless. He did not see any advantage to restricting names that had no priority, so he opposed the proposal.

**McNeill** added to Barrie’s point in that if you did not like descriptive names you did not have to use them, you could pick up a name of your own choosing that was formed from the name of an included genus.

**Brummitt** gave an example, in case people were not clear what it was about, because it took him a little time. He liked the term *Centrospermeae* for a group which was clearly defined and very traditional, but the proposal, he thought, would not allow him to use *Centrospermeae*.

**McNeill** confirmed that was correct.

**Brummitt** concluded that the proposal seemed too restrictive.

**McNeill** was not necessarily sure he agreed with *Centrospermae* being clearly defined, but that it was definitely a commonly used name was unquestionable.

**Prop. B** was **rejected.**

**Prop. C** (47: 102: 11: 1).

**McNeill** introduced Prop. C, that proposed an Example of a case where there was a distinction being made between an improper Latin termination and a non-Latin termination. He reported that the Rapporteurs took the view that if you were to favour this, you would need to vote it as a voted Example because it did not seem to in fact illustrate a criterion that appeared within the *Code* for determining whether or not a name was of that type.

**Prop. C** was **rejected.**

**Prop. D** (82: 15: 57: 1).

**McNeill** moved onto Art. 16, Prop. D and said that he could not understand why there was such a high Editorial Committee vote. He noted that the Rapporteurs did make a suggestion that there might be an editorial change but it was not a special request. He suggested it could be just accepted as a proposal and how the Editorial Committee worded it more clearly was its business.

**Turland** spoke on behalf of the Committee for Suprageneric Names. From his understanding of the proposal when discussed in the Committee, the suggested editorial change would not alter the intent of the proposal. He concluded that it could be referred to the Editorial Committee or simply voted “yes” or “no” and the Editorial Committee would deal with the suggested change by the Rapporteurs.

**Prop. D** was **accepted.**

[*The following debate, pertaining to Art. 16 Prop. E took place during the Fifth Session on Thursday morning with discussion on Art. 33*. *For clarity, the sequence of the* Code *has been followed in this Report.*]

**Prop. E** (71: 54: 23: 3).

**McNeill** introduced Art. 16 Prop. E, which was a possible change in the *Code* that would bring the existing provision for Phylum and Division used at the same time under the rule that had just passed. Art. 33 Prop. N on misplaced ranks.] He felt it was slightly different and did not automatically follow.

**Moore** admitted that it was something he wished he did not have to deal with, but it would seem a natural corollary to what had just passed. He felt that it had to be dealt with, to be logically consistent: What to do when Division and Phylum were used in the same classification? He explained that the rule currently in effect said that neither was validly published when both were used and the proposal would simply change it to both being covered under this informal usage. He added that perhaps sometimes Phylum was used properly but maybe Division was used as an informal rank. He felt that the change would make it logically consistent with Articles elsewhere in the *Code*. He was not too worked up about it, either way, because Division and Phylum were both above the rank of Family so priority was not in effect. He felt it did not really create instability, one way or the other.

**McNeill** believed that previously they would be considered not validly published and under the proposed situation they would be validly published but without rank.

**Moore** agreed that that was correct.

**Prop. E** was **accepted.**

[*A debate following on from the results of the card vote on Art. 14 Prop. A took place here but has been moved to after Art. 14 Prop. A in accordance with the logical order*.]

[*Here the record reverts to the actual sequence of events*.]

### Recommendation 16A

**Prop. A** (97: 31: 26: 1).

**McNeill** moved on to Rec. 16A which came from the Committee on Suprageneric Names and had a strong vote in favour. He felt it was the point at which the Rapporteurs had to point out that they did err in their comments here. He quickly corrected himself that, “No, sorry, we were perfectly correct here”. [Laughter.]

**Turland** thought it was almost an editorial change, it just depended on whether the Section felt that a so-called backdoor rule – where part of an Article mandated a Recommendation – which was the current situation, whether that was preferable to simply converting it into an Article, where it would be an obvious rule. He summarized that the aim of the Suprageneric Committee was to simply make the *Code* more readily understandable.

**Nicolson** noted that it was supported by the Committee – nine in favour and one against.

**Barrie** was not sure why, but the proposal really upset him. It had also upset him in St Louis. He did not see any reason to change it into a rule as he felt it was perfectly good the way it was. He pointed out that again it was dealing with names with no priority and forcing people to do something with names that they did not have to do use them. So even though he thought it was good that people followed it as a Recommendation, he would prefer it not be a rule.

**Turland** made the comment that the current situation in the *Code* mandated those terminations anyway, so there was no change. The proposal did not make a change to what you had to do.

**McNeill** added that referring to Art. 16.3, it was apparent that it was one of those situations in which the Recommendations were mandated by the provision of 16.3, so it was substantially editorial, but perhaps putting a greater emphasis than it did hitherto.

**Turland** did not think it was 16.3. He offered to explain the backdoor rule. He believed it was in Art. 16.1 and it would be in the sixth line, where it said “as specified in Recommendation 16A. 1–3 and Article 17.1”. In other words, he suggested that automatically typified names were formed by replacing the termination -*aceae* in a legitimate name of an included family based on a generic name, by the termination denoting their rank as...

**McNeill** interrupted with apologies to say that it was 16.3, when an automatically typified name above the rank of family had been published with an improper Latin termination, not agreeing with those provided in Rec. 16A. 1–3, the termination must be changed. His point was that they were both saying the same thing, although referring to different Articles.

**Demoulin** felt that there was an important difference between the present situation and the proposal, which he strongly opposed. It is that the Recommendation was general and, for example, *Ascomycetes* was a descriptive name, not an automatically typified one. He thought it was a very good recommendation to have *Ascomycetes* so the present situation should not be changed.

**K. Wilson** agreed with Barrie that she also got hot under the collar but in the opposite direction to him. She objected strongly to Recommendations that were actually mandatory because of something written in the main body of one of the Articles. She was all for including the Recommendation in the Article itself because, as had been pointed out, it was referred to extensively in Arts 16.1 and 16.3.

**Demoulin** said there was an important difference between the proposal and the present situation: Art. 16.3 refers to automatically typified name while Rec. 16A covers also the descriptive names. It is useful to have this Recommendation, *Ascomycetes* is the form to be recommended.

**McNeill** wished to raise a concern with the Chairman of the Committee for Suprageneric Names, that he felt may be unfounded but worried him a little. He wondered if the new Art. 16.4 *bis* would supersede Art. 16.3 and if it did, would it invalidate names which were valid but needed to be corrected? He was not clear on the relationship between the new Art. 16.4 *bis* and Art. 16.3 and wanted to know if Art. 16.3 would have precedence?

**Turland** explained that the proposed Art. 16.4 *bis* replaced the backdoor rule in the sixth line of Art. 16.1 which was clause (a) that applied to automatically typified names, which had to have a termination denoting those specified in Rec. 16A. 1–3. He continued that the reference in Art. 16.3, that basically dealt with names which were published with an improper Latin termination, would be corrected and the name would still be validly published. He noted that the reference to Rec. 16A 1–3 in Art. 16.3 would be changed editorially to refer to the new proposed Art. 16.4 *bis*.

**McNeill** agreed that then he could follow what was being suggested. Apart from the loss of the Recommendation on names that were not automatically typified, to which Demoulin referred, he suspected it made no fundamental difference but was changing the way it was laid out.

**Barrie** followed on from Demoulin’s comment in saying that if it worked the way it was, although there was the inconvenience of having a backdoor rule, he wondered why the Section should change it, if names could be lost because of the change?

**Turland** clarified that Rec. 16A.1–3 currently was only a backdoor rule for automatically typified names, so there would not be any change.

**Barrie** asked him to clarify if his argument was that no names would be lost.

**McNeill** did not think anything would be lost, other than a Recommendation as to what you do with names that are not automatically typified. He did not think it changed anything except that.

**Demoulin** did not see any reason to lose the Recommendation for those not automatically typified names. He felt it was a good Recommendation, with no reason to delete it because some people found it more convenient to. He added that it was a useful way of doing it and a useful part of the Recommendation, so he agreed with Barrie that “if it works, leave it in peace”.

**McNeill** pointed out that that was what the Rapporteurs said, that it worked but it could be changed. He added that if it was changed it had to go after 16.1.

**Malecot** wondered if the wording was correct, because under the proposal the ending for division or phylum was -*phycota*, whereas in the current text it was -*phyta*. It was the same for the ending for subdivision or subphylum, in this proposal the ending was -*phycotina*, whereas in the current text it was -*phytina*. He wondered if this was maybe just an orthographic feature, but to him the proposal was not exactly the text in the Rec. 16A.

**Demoulin** agreed that was perfectly correct and there was one more and very big reason to defeat the proposal. He felt it was absurd.

**Prop. A** was **rejected.**

**Prop. B** (90: 46: 15: 3).

**McNeill** pointed out that there was a typing error– they did finally find an error in the preliminary mail vote, with great difficulty!

**Nicolson** explained that what appeared as Art. 16A was, in fact, Rec. 16A.

**Turland** explained that seeing as Rec. 16A, Prop. A was defeated, the proposal was to add to the existing Recommendation.

**McNeill** explained that it was really adding another series of recommended endings and, as he thought the Rapporteurs had noted, they were not being made mandatory under Art. 16.1.

**Turland** agreed that was correct because the backdoor rule in Art. 16.1 applied to Rec. 16A.1–3 and it would not include four, which would be the paragraph for this proposal if it were passed.

**Demoulin** supposed that at the next Congress the same Committee would make a proposal to turn the Recommendation into a rule. Even as a Recommendation he did not think it was very useful, but that it made the *Code* even more cumbersome and it did not, as the Rapporteur noticed, make any move with uniformization with other Codes. He was definitely against.

**Kolterman** wondered how relevant the proposal was since Art. 4, Prop. A was defeated, so that many of the ranks superclass, superorder, superfamily, supertribe, were not even in the *Code* anywhere.

**McNeill** thought that was a good point.

Probably 10 years or more ago, before the last *Code*, **Buck** had published an article in *Taxon* with Dale Vitt describing superfamilies of mosses. Up until then they had found no use of superfamilies whatsoever and in that article they proposed an ending, which was not the ending here.

**Gandhi** commented that, while indexing these suprageneric names he had come across a situation wherein two different authors used two different endings for the same rank, so just looking at the end one might not be able to guess the rank, so provided it was only a Recommendation he felt it should be okay to have these endings.

**Wieringa** felt that especially since Art. 4 was defeated, now at least “super-” would be available for all ranks when desired; even superspecies were available, so that was not a reason to take all these “super-” names out. He thought it would be most useful to have standard endings for these not-so-often-used levels.

**Prop. B** was **rejected.**

### Article 18

**Prop. A** (121: 28: 6: 0).

**McNeill** moved on to Art. 18 where the mail vote was strongly in favour. He added that Art. 18, Prop. A was one that came from the Committee on Algae and both Prop. A and Prop. B addressed similar situations. Prop. A dealt with the very unusual situation in which you had the possibility of two identical family or other higher rank names having to have the same termination, unless there was some way to avoid their being homonyms even though they were based on different generic names. It seemed to the Rapporteurs to be an elegant solution to the problem.

**Moore** also liked the proposal quite a bit but wanted to raise some issues. He felt that there were two ways to deal with the problem. One of them was to tinker with the word formation, which was being proposed and the other was to permit a homonymy at these ranks. He noted that the issue had been addressed before at the Tokyo Congress. He suggested that the other approach to the problem was what was taken up to deal with subfamily and tribal issues. He pointed out that, in fact, the homonym rule would actually have to be addressed in a later proposal. He noted that the homonym rule was now limited to a name of a family, genus or species, unless conserved, the original rule retained family in the homonym provision. He wanted the Section to consider perhaps extending this kind of logic to the subfamily level and consider restoring the homonym rule back to the way it used to be, which was to cover all the ranks. He thought one of the dangers was doing it one way for the subfamily, infrafamily levels in Tokyo. He felt that doing it a different way at the family level created a complicated *Code*, and suggested that it would actually be possible, in a rare act, to perhaps simplify things. He suggested doing it one way, across the board for the families and then perhaps going back to that broad-based homonym definition because he thought homonyms were something that were taught in basic nomenclature. He felt that the way the rule was now, that had been kind of chipped away at a fair amount.

**Rijckevorsel** was thinking about the same thing and would say that if the proposal was accepted, that it automatically would also reflect into the names of the subfamily, subdivisions of families and that indeed it would have repercussions, or possibilities rather, for the homonym rule, which was changed. He had been thinking about the homonym rule and would have liked to change that but it was pretty complicated so he had stayed away from it. He thought that it would be really nice if at the next Congress it would be possible to deal with that and thought that would be easier if the proposal was accepted.

Linguistically **Gams** found *Dictyosphaeriumaceae* terrible. [Laughter.] Rather than getting stuck with the homonym situation, he wondered if there was not the possibility just to take another generic name for creating a family name?

**McNeill** replied that from his understanding from the proposal that there were some situations, including perhaps this one, in which it was actually impossible because it was a monogeneric family.

**Gereau** thought there seemed to be two alternative solutions. He felt that the current proposal proposed using bad Latin to create near-homonyms, which were still quite easily confused and it did not seem to be a very good solution. The other proposal, whether there was another generic name available or not, was to propose a *nomen novum* because there was a homonym or a near-homonym situation and give it a completely different name based on an included genus, or if not just a *nomen novum* formed arbitrarily if necessary.

**McNeill** responded that a superfluous illegitimate generic name would have to be created to do that, and only if illegitimate names of genera were allowed to be the basis of a family name, which was still to come to. He concluded that there were problems with that solution.

**Wieringa** did not agree, because apparently there were only a few cases where this was a problem. He continued that indeed it was rare to have two family names which were so similarly spelled. He meant if only two such cases existed then maybe there would be five in the future. He felt it was always possible to create a new genus based on only one specimen, which was not a type of anything. It would be a valid generic name and then that name later could be used, in the next publication one day later, for your new family name, so there was no problem.

**Nicolson** had the quick reaction that taxonomy should come before the nomenclature, instead of the nomenclature before the taxonomy.

**Moore** suggested that if the situation went back to the homonym rule the way it was and dealt with these with word formations, he did not think since Tokyo there had been any case where that revised homonym rule had had to be used. In other words, he remembered the actual proposal dealt with the hypothetical situation involving *Caricoideae* that might be based on *Caricaceae* and also the *Caricoideae* based on *Carex* in the *Cyperaceae*. But he pointed out that it was strictly a hypothetical situation and he did not know of a case where there were two homonymous names actually in use, because the later one was not legitimate under the revised Art. 53.1. The way that the proposal dealt with it was the way the zoologists dealt with this basically and they had more experience as the botanical community was actually dealing with it for the first time. He agreed with Nicolson that he did not like the idea of creating a new genus name just to accommodate the family rule. It might be a genus that was in wide usage and it would create a lot of nomenclatural instability. He felt that the fact that they may be close was problematic to some degree, but at least in terms of indexing and what-not they would be different enough so you would not have those problems.

**Turland** wished to make a comment on Moore’s previous point about the infrafamilial ranks. If Art. 18, Prop. A were to be passed and this clause were inserted into Art. 18.1, when he looked at Arts 19.1 and 19.3, it said that the names for the infrafamilial ranks were formed in the same manner as the name of a family and it referenced Art. 18.1. He felt that surely the proposed rule would apply to those infrafamilial ranks as well, not just families?

**McNeill** asked if he was suggesting that it was an even more elegant solution than we [The Rapporteurs] had come up with first?

**Moore** wished to just throw out a hypothetical situation. Under the revised proposal he asked if someone wanted to take the genus *Carex* and make it a family with only that genus included, ignoring the synonyms that could be used, the family name would be *Carexaceae*, was that correct? Then the subfamily name would also be based on the type that would already be available, *Caricoideae*, because it was already in the literature. He did not see it quite as simple as that, but he did see the solution in that direction.

**Nicolson** made the personal statement that he did not like the nominative singular to be used as a part of the stem, adding that if it was only used to avoid homonymy, he thought he would vote for it.

**Prop. A** was **accepted.**

**Prop. B** (95: 36: 22: 0).

**McNeill** introduced Art. 18, Prop. B, which was also from the Committee for Algae. It stemmed from the last proposal but could be passed without the proposal. He thought it probably had to be passed now the proposal has gone through.

Like Gams, there were things **Demoulin** did not like to hear and he was sorry about what they had just done [allowing the nominative singular to be adopted instead of the stem]. He thought it was not as offensive as this one because he thought he was responsible for the expression “full word”, which was deliberate and probably about the time of the Leningrad Congress, because he did not see why there would be a need to speak of a nominative singular in a language where there were no nominative, genitive or whatever else. He thought it was part of a proposal that he made, approved by the Editorial Committee and it stayed there for five congresses. He really did not see why it should be changed now. It was meant to cover all situations in *Ginkgo* and whatever else. He asked, “Why speak of nominative *Ginkgo*? You know what the genitive of *Ginkgo* is?” His issue was with the replacement of “full word” by “nominative singular”.

**Rijckevorsel** felt that the comments by Demoulin were entirely logical, especially as the name of a genus could be derived from any source whatsoever. If something was not really a grammatically correct word then “full word” was a lot safer than “nominative singular”. He supported Demoulin entirely.

**Prop. B** was **rejected.**

[*Discussion of Art. 18, Prop. C was included in a package of proposals on orthography by Rijckevorsel and can be found under Art. 60 in the 6th Session on Thursday afternoon.*]

**Prop. C** (50: 65: 38: 1) was at that time referred to the **Editorial Committee.**

**Prop. D** (10: 136: 5: 0) was ruled as **rejected.**

**Prop. E** (127: 15: 8: 0) was **accepted.**

**Prop. F** (8: 74: 68: 3).

**McNeill** introduced Art. 18, Prop. F as a proposal by the same proposer but on a somewhat different topic. It proposed to elaborate on what a non-traditional or inappropriate Latinized termination was. He explained that the proposal should be considered as a proposal, but should it be favourable the Example should not be considered a voted Example but referred to the Editorial Committee.

**Nicolson** noted that *Lauraceae* was already conserved.

**McNeill** reported on the mail vote; the high Editorial Committee. vote was because the Rapporteurs’ comments implied that the Example could be referred to the Editorial Committee, not being enthusiastic about the wording of the Note.

**Turland** felt he should just make a comment as the members of the Suprageneric Committee who supported it had some concern with one of the terms used in Art. 18.4, the word “improper”. It seemed that there may be some differing interpretations of that word in that context and he believed the proposal was aimed at clarifying what was meant by “improper”. He asked if any of the proposers cared to comment?

**P. Wilson** was one of the proposers and he felt there were some problems with it as written and he thought it did need editorial input. In the first Example use of “non-traditional” was a bit of a problem because *Lauri* was a traditional Latin ending, genitive singular. There was a reason why they were in favour of it, but he thought some of the Examples may need a bit of help because “Carpantheous” could be considered as having a Greek ending, because that was not Latin he suggested that could be deleted. But *Beslerides* was a bit more of an ambiguous situation. He was generally in favour of the proposal but thought that perhaps the Examples needed a bit of assistance from the Editorial Committee.

**Prop. F** was **rejected.**

[*The following debate, pertaining to Art. 18 Prop. G and H took place during the Fifth Session on Thursday morning with discussion on Art. 33*. *For clarity, the sequence of the* Code *has been followed in this Report.*]

**Prop. G** (112: 23: 13: 3).

**McNeill** turned to Art. 18 Prop. G which was in the context of the rule which said that a natural order which was intended to be a family should be treated as if it were a Family.

**Moore** thought that both of the proposals were fairly logical and the Article and the Example was fairly logical. He actually thought it was possible to simplify the language a little bit. He wanted to propose an amendment to the proposal to Art. 18.2. As it currently read, he explained that it said names published with a rank denoted as order or natural order should not be treated as having been published at the rank of family if this would result in a taxonomic sequence with a misplaced rank-denoting term, or if the term family was simultaneously used to denote a different rank in a taxonomic sequence. Since order and family were side-by-side in the taxonomic sequence, he could not envision a situation where converging from order to family would result in a misplaced rank-denoting term. The only case would be if the Section did not adopt the proposal involving sequential use. He changed his mind and decided not to propose a change.

**McNeill** checked that he wished to keep the wording the way it was.

**Moore** agreed to keep the wording as it was. He added that the issue was a source of a lot of discussion in the Special Committee on Suprageneric Names. He thought the Note was fairly intuitively obvious but not everybody had applied the Article that way. Regarding the next proposal, an Example, he reported that the minority opinion in the Committee for Suprageneric Names vis-a-vis the Berchtold & Presl proposal, was that the orders in that particular publication were to be converted to families where there was a rank-denoting term that clearly had to be translated as family so that you started with a order-family sequence and after you invoked the Article you then had a family-family sequence. He felt that, depending how you interpreted that, you had a misplaced rank-denoting term problem and it seemed a little bit tortuous to him. He thought one should just stick with “they were” and not invoke the Article.

**Turland** mentioned that the majority opinion in the Special Committee on Suprageneric Names was, indeed, to treat the ranks as described in the Berchtold and Presl work.

**Atha** wondered if only internal evidence was to be used to determine these problems or if you were supposed to go back to a prior publication to apply the rules?

**Moore** replied that there was nothing in any of the proposals that dealt with that. He thought the general approach was to stay internal to the work. He seemed to recall there was maybe one case in the *Code* where that was not done, but, otherwise he thought it seemed logical to restrict yourself to the work itself or the problem may never be solved.

**McNeill** thought it was a rather woolly Article, not the proposal, which he felt was perfectly clear, and would be solely looking at internal evidence. He felt that the issue of when you know that an order was really meant to be a family was one of the Articles in the *Code* that worked fairly well most of the time but was not well defined. Indeed, he thought that many people did tend to use external evidence for that in terms of what other people at that time were calling families, but the important thing was that natural order and family moved gradually and imperceptibly from natural order to family historically in a fairly imperceptible way. He argued there was just a switch in terminologies which was why we had the provision in the *Code*. He quite agreed with the point that it was not well defined but most of the time he felt it was not a problem. He added that the problems that had arisen were where a person did have an order with the taxonomic content that many people at that time treated as a family but also had a family and he felt that this was being covered quite clearly and sensibly in the proposal.

**Gandhi** referred to Art. 35.5 dealing with publication in different parts or volumes of a publication but not different editions of a works. He wanted to know if it was a situation where different parts of a publication or different volumes of a publication but not different editions of a publication could be used, even if a specific act was not mentioned on a particular name? [No-one seems to have replied to his query.]

**Prop. G** was **accepted.**

**Prop. H** (109: 25: 11: 4).

**McNeill** felt that Art. 18 Prop. H was a logical, simple Example that many... He interrupted himself to say that he should talk to the proposer as now that the last proposal had passed he failed to see why it would need to be a voted Example as it seemed to be quite a necessary corollary of what had just been approved.

**Moore** agreed. The only question he had was whether there was any concern about the translation of the terms as they were not in Latin. He clarified that was just so that it was abundantly clear what was supposed to be done and people could not interpret it a different way. He gave that as a potential reason why it should be a voted Example.

**Turland** explained that there was quite an extensive discussion in the Special Committee for Suprageneric Names about the particular work. He thought the Committee would like it to be a voted Example just to remove any possibility for further ambiguity on the matter.

**Marhold** agreed that it would be useful to have it as a voted Example.

**Demoulin** did not think it was appropriate to vote in a case like this because he felt that the problem was that the Committee was not quite sure how to interpret “rad” and “celed” and in a case like this, it was not up to a Section to decide. He felt that it was something that must be decided with the book, with people with experience of the language and the language of that time. He concluded that it was a problem of special expertise, not a problem for a general discussion by the Section. He argued that democracy had nothing to do with it when it came to translating and seeing the documents and suggested referring it to a Committee and the Committee would look for the advice of competent people. He did not think the Section should vote on an issue like this.

**McNeill** suggested that the Section could, if they wished, vote that if the Editorial Committee thought it needed to be a voted Example it should be or it could just be a regular Example. He felt that the point was that, if in fact, there was no ambiguity in the translation of the two Czech words then it was not a voted Example because it followed immediately from what had just been approved. He argued that if there was doubt about it then, yes, it should be a voted Example.

**Marhold** reported that he had spent a quite a lot time discussing the term with his Czech friends and there was nothing that could be added to this discussion.

**McNeill** checked that he was saying that the words were unambiguous in their meaning.

**Marhold** replied that “celed” meant family today but the question was, what it meant at that time.

**McNeill** asked what did “rad” mean?

**Marhold** echoed that it was order today.

**McNeill** then thought it should be treated as a voted Example.

**Nicolson** felt that might be useful, but acknowledged Demoulin’s point that the difficulty was that historically, words and names had changed their meanings.

**Gams** still had a slight difficulty understanding the Example. He thought that as it stood the normal situation was that it [the term] was sometimes used at a rank below order, but he gathered that it was sometimes used above, sometimes below.

**McNeill** disagreed and thought that the question was whether the rank of order was to be treated as that of family under Art. 18 as some had felt it should be. He thought, having passed the previous proposal, that it was now self-evident and that was why he was wondering if it needed to be a voted Example, but there was no problem in it being one.

**P. Hoffman** had the same problem. As far as just reading it without discussion it says to her that the Czech term family, had sometimes been used below the rank of order, and family was a rank below order so she did not understand the point of the Example.

**Turland** offered a bit of background in the particular Example. At the last Congress in St. Louis, Reveal and Hoogland had proposed a long list of changes to App. IIB in the *Code*, the list of conserved family names. He thought that about 45 of the proposed changes of author and place of publication were to this particular work by Berchtold and Presl. The names that were supposedly published as families in that work were ranked as “rad”. In some cases they were subdivided into subordinate ranks terms termed “celed”. Taken at face value and translated according to at least modern Czech, if not the Czech language of 1820, you had orders divided into families. Reveal’s interpretation was that the term rad or order was intended as family so that could be changed to family under Art. 18.3. But that left you with the problem of the subordinate ranks “celed”, which translated as family. You can not have families subdivided into families unless you treat one of them as a misplaced rank-denoting term and therefore the name was invalid. In fact, Reveal was of the opinion that the ranks termed “celed” were tribes, but there was nothing in the *Code* that allowed you to treat something ranked as family, but supposedly intended as tribe, as a tribe. So the Special Committee for Suprageneric Names deliberated over this at great length and decided eventually by a sizable majority, he thought there were two in the minority, that the ranks in Berchtold and Presl’s work should be treated as ascribed. He noted that if the Section did not follow the view reflected in the Example then it would be necessary to introduce all the Berchtold & Presl names into App. IIB for about 45 family names from that work and, of course, if there was still some ambiguity and disagreement about what the ranks meant then there would be a problem with the Appendix. He concluded that if the Section passed the Example it would basically have a stabilizing effect on App. IIB and the implications were wider than just an Example of the proposal we just passed.

**McNeill** added that in the discussion in the Committee on Suprageneric Names, he thought the minority was wrong in its interpretation of the *Code* as then written. He felt that having the Example in the *Code* would put a seal on that. He reiterated that he thought having it as a voted Example was nonsense because it was clearly a necessary corollary of what had just passed. He argued that it was definitely needed in the *Code* to put the matter totally to rest. The minority view was defensible under the slightly ambiguous wording that existed and he thought the ambiguity no longer existed. He was a little worried about insisting it be a voted Example because then it diluted the meaning of a voted Example.

**Gandhi** requested a clarification from the Example whether the term family was used in the 1820 work to denote either any suborder or subfamily or totally as unranked and ambiguous.

**Turland** asked if the question was “Was the term family used in this work”?

**Gandhi** replied that the Example illustrated that the term family was used below the rank order. What he was asking was whether it was used in the sense of suborder, or subfamily, or totally unranked, so that it was ambiguous.

**McNeill** thought that there were only the two ranks involved, one translated as order and the other as family, and they were used in the correct situation.

**Turland** confirmed that was correct.

**Nicolson** was a little baffled. It appeared to him that the Example would be nice to have in the *Code* but whether it needed to be a voted Example seemed to be the question.

**Per Magnus Jorgensen** felt that if it was a voted Example, it would undermine the understanding of voted Examples which were not good anyway. [Laughter.]. He misunderstood [the concept] until he had to be on the Editorial Committee. He felt there must be a technical way of dealing with it that should be left to the Editorial Committee.

**Nicolson** asked Moore if he would take it as a friendly amendment that it be included as an Example but not as a voted Example.

**Moore** agreed, adding “any way to pass it”.

**Nicolson** moved to a vote on Art. 18 Prop. H which had been modified not to be a voted Example but as an Example.

**Prop. H** was **accepted.**

[*Here the record reverts to the actual sequence of events*.]

**Prop. I** (35: 118: 2: 1) and **J** (17: 136: 2: 1) were ruled as **rejected.**

**Prop. K** (86: 42: 24: 0).

**McNeill** introduced Art. 18, Prop K and noted the results of the mail vote.

**Rijckevorsel** felt that for technical reasons he could only say something about the proposal and explain why the Rapporteurs’ comments were close to being nonsense after doing a presentation.

**McNeill** did not think there was time for a lengthy presentation. He asked if Rijckevorsel would like to explain the error that the Rapporteurs made?

**Rijckevorsel** thought that the discussion had better be transferred to tomorrow.

**Nicolson** noted that a little over ten minutes remained and the proposal was rather strongly supported in the mail vote with 86 “yes” and 42 “no”.

**Rijckevorsel** repeated that he felt strongly about the issue and wished to present the relevant facts before it was decided.

**McNeill** thought it was a proposal that was quite independent of the orthography proposals. It seemed to be dealing with a rather particular issue of some interest and relevance, but quite separate from the main thrust of the other submissions.

[Break for setup.] [I:47]

**Rijckevorsel** began by saying that there had been a miscomprehension that his proposals dealt with orthography exclusively but that was not quite true. This current proposal was in the proposal from the *Vienna Rules* 100 years ago, which was a very good starting point. He was going to start with a nice bit on the historical fact that the Section was here today 100 years after the orthography paragraph was first introduced into the *Code*, but he skipped quickly to the next part. Also from the *Vienna Rules* of 100 years ago and, he felt, a very important provision which went back to Candolle’s *Lois* of 1867, namely, Art. 2. This [again, reference to presentation?] was felt by Candolle to be a very important part of botanical practice and he put it almost as the first Article but just not quite. At the Congress of Vienna it was put in the third place and at the moment it was still in the *Code* but unfortunately hidden away, in a very good spot, in the first line of the *Code*. So he argued that it [unclear what it is from the transcription, presumably clear in his presentation] was pretty basic to the entire nomenclature practice. He went on that the basic consideration to all the proposals, except the ones on Art. 19, was that botanists were not doing all that well, plant species not doing well, herbaria were not doing well. He argued that of the very many things that the Section could not do, there was one thing that we could do and that was to look after the *Code*. He argued that the *Code* had a central place in botany and a change of a few words could make a considerable difference. He thought that Lanjouw said it very well, especially the part where he said “We learned to be careful with regard to the words we used and we realized how difficult it is to express clearly what we have in mind”. Especially also the line from the *Stockholm Code*: “Never before had to go through such a huge pile of scripts and I never before came across so much difference of opinion with regard to so few words and never before have I had to pay so much attention to comma and semi-colons”.

**Nicolson** asked him to please come to the point.

**Rijckevorsel** continued that it was right up in front. A clear illustration of this was provided by the contrary to Art. 32.1, which said a presence in [unclear] doing that. This is one way of doing things: there is a rule and there must be an exception made to the rule and how do we do it? This same matter of doing things was later also included in Art. 19.5 and the other two Articles. He asked the Section to think of all the botanists having to leaf back and forth from Art. 19.5 to Art. 32.1, seeing there “have a form which...”, trying to figure out what that meant. Then going back to Art. 19.5, seeing that they have to go back to Art. 19.1, where they see that the name of the subfamily is formed in the same manner as the name of a family. Then having to go back to Art. 18.1. He argued that it was a very roundabout way of doing things. He felt that the nice thing about the Example was that in some cases it was possible to argue about what was complicated, but not here because he suggested that Art. 19.5 was as dead as a doornail. He argued that it did not do anything, or rather it did do something but not something that was wanted. An exception was made for names that were validly published and which names were validly published? Those names of the subdivision of a family that were illegitimate, the ones that were not the base of a conserved family name. So he continued that if you had a genus as the base of a conserved family name, you could base a subdivision of a family on that. Then that was not validly published, that was not covered here. He reiterated that this was a very roundabout way of doing things, which was so complicated that the Editorial Committee could not handle it.

**Nicolson** was afraid he was going to have to close the discussion because of the extra costs of staying late as it was already six o’clock.

**Rijckevorsel** suggested that he would continue the following day.

**Nicolson** preferred to vote on the proposal.

[*Prop. K was accepted but discussion reopened on Wednesday.*]

## Third Session

Wednesday, 13 July 2005, 09:00–13:00

**Stuessy** hoped that everyone had survived their first night in Vienna. He notified the Section that the group photo would be taken at the beginning of the coffee break. For those who required internet access, he referred to the user name and password needed. He added that the Bureau would keep an eye on those behind computers, as “we know that as soon as you open your computer you will be working on manuscripts etc and not paying attention to the discussion, which will automatically disqualify you from voting”. [Laughter.]

### Article 18 (continued)

**Nicolson** wished the Section a good morning and moved straight on to begin with Rijckevorsel who was finishing his last presentation. He asked if it was possible to finish it from his seat?

**Rijckevorsel** said “No”.

**McNeill** reminded everyone that the presentation was on Art. 18 Prop. K.

**Rijckevorsel** realized that everything had not gone as well as they might the previous day and had noticed that he was quite dehydrated. He continued that there were two reasons why he was quite unhappy with the way things were going. He felt that the heavy mail vote was based on the comments of the Rapporteurs that were contrary to the *Code* and he wished to address that. Secondly, he thought the proposal was connected to Art. 19 Props L & M which he thought had survived the mail vote and could help. He asked that the Section decide whether or not the proposal should be addressed, adding that he was a limited kind of person who could only discuss what he could show [via slides]. He pointed out that there was nothing saying that a proposer could not support their proposals with the aid of a brief presentation. He realised that time was of the essence and assured the Section that he would be as economical as possible.

**Nicolson’s** first response was that almost everyone had read all the proposals and voted so the mail vote expressed its opinion. He suggested that if something was not properly handled it could be revisited but stressed that there was a limited amount of time available and 10 minutes had been spent on the issue the day before. He added that he would still like to see the proposal addressed and asked the Section if they would like to have a continued presentation [the Section did not wish to] or would rather deal with the proposals and let the proposer address any questions that might arise [this was acceptable].

**McNeill** reminded the Section that the proposal to be addressed first was Art. 18 Prop. K, which received a relatively favourable mail vote: 86 “yes”, 42 “no”, 24 Editorial Committee. Once that was addressed he suggested discussion could move on to the others that did not receive such a favourable vote.

**Rijckevorsel** wished to make one brief comment: Props K & L were alternatives. He felt that both would effect an improvement in the *Code*, but Prop. L would effect a greater improvement. He wished to make the point that it was easy to base this on conserved names. He thought that the Rapporteurs knew this as they had made a comment about “presumably already conserved” which is irrelevant because Art. 14.1 states that the *Code* maintains a list of conserved names. He asserted that there was only one *Code*, currently black; he hoped next year that it would be orange [perhaps to honour the Netherlands?]. He suggested that if a name was on the list, then all the provisions dealing with conserved names applied to it and if it was not on the list, then they did not. He thought it seemed quite straightforward...

**McNeill** reminded Rijckevorsel that he was addressing a proposal that was not before the Section, Art. 18 Prop. L, which was defeated by more than 75% in the mail ballot. He added that it could come up later but advised that Rijckevorsel would be much better to consider the proposal that got support on the mail vote, Art. 18 Prop. K, which was not at all related to whether a name was or was not conserved, but to whether a family name could be based on the stem of a generic name that was illegitimate.

**Rijckevorsel** believed that Art. 18 Prop. K was entirely editorial and would effect an improvement in the *Code*.

**McNeill** disagreed and felt that Prop. K was not editorial and required the approval of the Section. He explained that the Editorial Committee could not change such an important thing as requiring a family to be based on a legitimate generic name. He felt that the proposal would simplify a lot of cross-referencing in the *Code* and the Rapporteurs did not see any reason why a family name should be restricted to being based on a legitimate generic name. It did not seem destabilising to make the change that Rijckevorsel had suggested, however, he reiterated that it was not editorial.

**Zijlstra** concentrated on the main point: “In Art 18.1, delete legitimate”. She felt that that was a fundamental change, and thought such a change should only be made if there were compelling reasons to do so and she did not think there were. She felt it would cause uproar [literally she said “rumoer”, which means commotion or uproar in Dutch]. She had looked at the mail vote and also at the Rapporteurs comments, which said that Props K & L were alternatives, and she suggested that one might think that the Rapporteurs did not see a problem with Prop K because it was logical. However, she pointed out that the *Code* was not always logical [laughter] and thought that the Section should not try to make it more logical if it would cause problems. She noted that despite the Rapporteurs’ comments the proposal had quite a lot of negative votes.

**Demoulin** could not understand so much time was being spent on the issue because Props K & L were alternatives. He felt that, although the proposer apparently preferred Prop. L despite the mail vote, Prop. K was preferred by a large majority of people. He did not see any reason why the Section could not make the *Code* simpler and more logical whenever the opportunity arose. He urged that whenever it was possible do that, it should be done. He felt that Prop. K was a good proposal, summarising that it had a good mail vote, it had the Rapporteurs support and it had his support and he hoped to vote on it quickly!

**McNeill** wanted to be clear that when the Rapporteurs said that the proposals were alternatives that there was a third alternative. He then mused about whether one can have three alternatives and concluded that you can in English, although not if you were a purist [remembering previous discussions with the Rijckevorsel on the use of that word]. The third alternative was to leave it just as it was because there was no question that it was clear and it worked. He thought that was what Zijlstra was suggesting. He continued that if there really were issues that the Rapporteurs had overlooked in saying that it would be a simplification that would do no harm, then of course they would like to hear the issues. Other than that he thought that the Rapporteurs view was that you could vote for it as Demoulin had suggested or vote for the status quo. Either way, he felt it would not change the current situation.

**Brummitt** was relying on notes he had made two months ago but it seemed to him that Rijckevorsel was right. He agreed that there was a logical conflict between Art. 18.1, which said that a higher ranked name must be based on a legitimate name, and Art. 18.3, which allowed names based on illegitimate names. He thought that Prop. K had a lot going for it.

**Prop. K** was **accepted.**

**Prop. L** (9: 129: 14: 2) was ruled as **rejected.**

### Article 19

**Prop. A** (24: 75: 31: 0) was referred to the **Editorial Committee.**

**Prop. B** (11: 135: 8: 0) and **C** (11: 135: 8: 0) were ruled as **rejected.**

**Prop. D** (108: 38: 12: 2) was **accepted.**

[*Vote was on Thursday morning during discussion of the Moore package on misplaced ranks*].

**Prop. E** (31: 107: 12: 1) was ruled as **rejected** as it was a necessary corollary to Art. 18 Prop. I which was rejected.

**Prop. F** (52: 87: 12: 0).

**McNeill** introduced Prop. F, which was the first of a series of proposals dealing with the situation where the name of a subdivision of a family did not have any sort of special status even if it included the type of the name of the family. He explained that if that family was combined with another, as in the case of *Epacridaceae* and *Ericaceae*, then the subfamily name *Epacridoideae* did not have precedence over other names that might be competing with it. It was proposed by Rijckevorsel who was going to make a presentation on it.

**Rijckevorsel** had noted earlier, that his proposals mostly offered an editorial kit to tune up the *Code* and he tried to stay as far away as possible from any policy issues. Despite this, he had made these proposals anyway, as he thought that the point was at least worthy of consideration. He felt they were very good proposals and he had tried to be as minimalistic as he could. He explained that what the proposal would do was take the protection afforded by Art. 14.1 for family names and Art. 19.4 for subdivisions of families which were protected. If a taxonomic change occurred then he suggested that such names were left out in the cold. He added that the change would affect very few names and he had made a list that had been available on the internet for a year or so. He took App. IIB and compared it to the latest edition at that time of the well-known reference by Mabberley. He gave the example of another name that would benefit: *Maloideae*, the subfamily of the apples, which was the best-known and most notorious case, which he argued could not be resolved in any other way. He felt that the set of proposals was good, but compared to the larger issue of orthography it did not have any great priority for him.

To **Demoulin** this was much more important than orthography. He felt that there was what he believed was an unfortunate movement in the conceptions of families because of cladistic philosophy. He characterised it as all kinds of splitting and lumping and at our level of nomenclature he urged the Section to try to limit the pernicious effect of this philosophy. He thought it was very important to be able to retain in the subfamilies the names that the large user community was used to. The argued that things like *Epacridaceae* becoming *Staphylloideae* would make the big community of users very unhappy, so the proposal must pass.

**Wieringa** noted one basic thing. If the proposal passed, he thought it would be the first place in the *Code* where priority on one level would give precedence over names on another level, in other words that the proposal would establish priority outside the rank of a published name, which looked to him more like a zoological *Code* thing. He thought it looked like a small shift in that direction and was not sure everyone was aware of that.

**Prop. F** was **rejected** on a show of cards.

**Prop. G** (38: 85: 27: 0) and **H** (37: 85: 28: 0) were **withdrawn.**

**Prop. I** (8: 133: 8: 0) was ruled as **rejected.**

**Prop. J** (28: 89: 34: 0) and **K** (28: 95: 28: 0) were **withdrawn.**

**Rijckevorsel** wished to make the comment that Prop. K addressed Ex. 4, and he understood from Turland that the priorities in the Example meant that it was no longer accurate and would need editorial attention.

**Prop. L** (9: 63: 79: 0).

**Rijckevorsel** introduced the proposal as dealing with a rather awkward point in Art. 19.4 concerning the phrase “generic name equivalent to the type”. He did not understand the phrase until he went back to older editions of the *Code* and discovered that the original wording was “type genus”. He tried to come up with wording to improve this and arrived at these proposals, which he was not really happy about. He submitted them to McNeill, who was also not very happy about them. He had been beating his head against the [hopefully proverbial] wall about the issue and wished to go back and amend the proposal to return to the phrase “type genus”. He noted that the phrase had been in and out of the *Code* for quite a while. The genus once was the type of the family, which it no longer was as the type was currently a specimen, but nevertheless the phrase “type genus” was found throughout plant taxonomy and he felt it would help the wording of the Article and also one of the other ones, and it would also promote general usage. He suggested it could be done in one of two ways. In Art. 18.1 it could be added that the included genus was referred to as the “type genus” or in the *Code* was referred to as the “type genus” or it could be done in Art. 10.6 where the matter of the type of the family…[unintelligible]. He had hesitated a long time before going back to something abolished earlier, but it was abolished by an Editorial Committee, not the Section, and he felt it was a well-known phrase that was unambiguous. So he wished to put it back in.

**McNeill** asked if this was an amendment to what was on the board. [It was.] He requested that the new wording be put on the screen. [This was presumably done, but no-one read it out.] McNeill felt it was clearly a totally new proposal. He thought, in the interest of speed and not being overwhelmingly legalistic, if the Section was willing to deal with it, it would enable faster movement through the proposals for Art. 19. He clarified that it was, of course, perfectly in order for the Section to say that it was “out of order” and not discuss it in which case it could be brought up at the end following the normal procedure. He summarised that the proposal was essentially self-evident and wanted to put into the *Code* a term that was not technically accurate in the sense that the type of the name of a family was a specimen. He elucidated that all types of names were specimens or in some cases illustrations. The proposal intended to demand that the type of the name of a family would be called the type genus.

**Rijckevorsel** felt that it would not go back to the old concept, but would be a phrase of convenience to help in the phrasing of the *Code*. He noted that it would also have to be applied elsewhere in Arts 18 and 19 where relevant.

**McNeill** queried whether he would presumably also suggest insertion of “type species” for the type of a genus in the appropriate Article?

**Rijckevorsel** was not willing to go so far, but thought that might be a matter to consider.

**McNeill** suggested that the Section would have to make up its mind whether mandating something that was clearly illogical should occur in the *Code*.

**P. Hoffmann** wished to know if the Section could also vote on the original proposal or if they had to vote for the amended proposal.

**McNeill** clarified that if the amended proposal was passed, then the original proposal was defeated, but if the amended proposal was defeated, discussion would return to the original proposal.

**P. Hoffmann** was against the amendment.

**Dorr** pointed out that if it was a new proposal or an amendment to the original proposal, then it had to be seconded.

**McNeill** agreed that was correct. [The amendment was **seconded.**] He noted that it was true that this was not strictly following procedure; but simply trying to facilitate moving forward.

**Barrie** thought it was a big step backwards. He was still fighting with people who thought that genera were the types of families. He thought that the *Code* had been deliberately reworded to emphasise the fact that the type was a specimen or illustration; it was an element. And he felt that the current wording, including the sentence “For purposes of designation or citation, the generic name alone suffices” [Art. 10.6] made everything perfectly clear. He argued that it was much easier to explain to people that genera were not the types of family names and that taxa were not types, using the current wording.

**Rijckevorsel** added that the amended wording could be added to Art. 10.6 or to Art. 18.1.

**McNeill** said that would be editorial.

[The amendment was **rejected.**]

**McNeill** returned discussion to the original proposal unless the proposer wished to withdraw it. [He did not.]

**McNeill** felt the need to mention that it was the opinion of the Rapporteurs that the proposal and the following three [M,N,O] were essentially editorial and should be referred to the Editorial Committee or defeated.

**Prop. L** was referred to the **Editorial Committee.**

**Prop. M** (12: 62: 76: 0) was referred to the **Editorial Committee.**

**Brummitt** was slightly confused about what the Editorial Committee was obliged to do? He continued that if hardly anyone was in favour of the proposal, did the Editorial Committee feel obliged to do something, or could it do nothing?

**McNeill** felt that, in light of the discussion, the Editorial Committee would treat this as an editorial matter and use its judgment whether the suggested wording, or some other wording, would improve clarity. He added that this also meant it was free to leave the wording unchanged.

**Prop. N** (14: 59: 77: 0) and **O** (12: 63: 75: 0) were referred to the **Editorial Committee.**

**Prop. P** (11: 82: 68: 0) was **withdrawn.**

### Recommendation 19A

**Prop. A** (16: 55: 79: 0) was referred to the **Editorial Committee.**

**Prop. B** (26: 95: 30: 0), **C** (24: 97: 30: 0) and **D** (25: 93: 33: 0) were **withdrawn.**

### Recommendation 19B (new)

**Prop. A** (8: 84: 62: 0) was **withdrawn.**

### Article 20

**Prop. A** (42: 72: 38: 0).

**McNeill** introduced Art. 20 Prop. A which he felt was not strictly orthography. He believed Rijckevorsel wanted to discuss it with the orthography group of proposals [Rijckevorsel wished to discuss it here.] He added that in the mail vote the proposal had received 42 “yes”, 72 against and 38 Editorial Committee votes.

**Rijckevorsel** felt it was a simple technical matter trying to come to a uniform use of the phrase “binary system of Linnaeus”, which otherwise did not occur in the *Code* and which was not defined, so he would prefer to be rid of it. He emphasised that it was a matter of wording with no change of intention in the Article.

**McNeill** suggested it could be referred to the Editorial Committee.

**Demoulin** did not think it should be sent to the Editorial Committee. In his opinion this should be voted “no”. He felt that the wording was deliberate to refer to all works of the 18th and early 19th centuries and the problem was to decide if those works were Linnaean in philosophy. He thought the wording of the *Code* was good, the Section should not touch it and the Editorial Committee would waste its time discussing it.

**Brummitt** wished to ask McNeill a question. He noted that in the past few weeks there had been a long series of emails going around about the genus name *Cleistogenes*, which was affected by the proposal. He thought that McNeill had suggested that the way to deal with this would be to change the Article. He had lost track of the endless discussion and wished to know if a proposal had been made?

**McNeill** replied that, unfortunately, there was not a proposal made, giving the reason that the person most concerned about it was not particularly involved in nomenclature on a regular basis and was currently involved with completing a vital manuscript for the Flora of China on the *Poaceae*. He added that the genus involved was in the *Poaceae*. He felt that the issue was quite a simple one and had nothing to do with the proposal, except that it was on the same Article. Proposal A was intended to be editorial and if the Editorial Committee found that it had an effect on the meaning of the Article, it would not act on it. He explained that what Brummitt had asked about was that normally all those technical terms that were listed in Examples in the *Code* were Latin; those that were Greek had been Latinized but the exception was *Cleistogenes*. This was an English language term in the singular, cleistogene, and was indeed a technical term at the time the name was published in the 1930’s. A replacement name, *Kengia*, had been proposed for it as it was described by a person named Keng. The issue had divided people for some time as to whether it fell under the Article or not. He thought that the issue would be simply resolved by adding the word “Latin” before “technical term” in the Article and the only reason that it had not appeared was that no one had had the time to do the research to see if any other names would be affected. He was saying this in the hope that someone wanted to do the homework and talk among colleagues in the next few days, it was a proposal that could be submitted at the end of the week when the other business was finished. He summarised that the answer to Brummitt’s question was no, there was no proposal because the person most interested did not submit one. Full stop.

In **Wieringa’s** opinion the proposal did not give a different meaning to the Article, but did seem to make it more clear, so from that point of view, he suggested the Section could vote for it. He was only concerned with having the word “currently”, both in the original and in this version. He felt that as soon as there was a morphological term that fell out of use, it could be resurrected as a genus name. He gave the example that maybe somebody would use a nice, established generic name from 1960 and then start using it as a technical term for something, which could suddenly invalidate the genus name. He proposed deletion of the word “currently” as an amendment, which would eliminate the problem.

**McNeill** thought that this was a legitimate amendment but noted that the proposal would no longer be simply editorial and would have to be voted upon. He mentioned that the issue had been part of the email commentary to which Brummitt referred. In that discussion he reported that there was some suggestion of changing the current wording to something like “in current use at the time of publication of the name”, so that the hazards to which the speaker just referred would be avoided. He added that perhaps simple deletion of “currently” might also meet the need.

**Wieringa** thought that perhaps the suggested wording would be better...

**McNeill** asked if he wished to formulate something along those lines or would it be better from the point of view of the Section if some discussion was allowed behind the scenes. He felt it was really independent of Rijckevorsel’s proposal and a new proposal could be considered at a later session.

**Wieringa** withdrew the amendment and agreed to see what came up in the next few days.

**McNeill** returned discussion to the original proposal.

**Per Magnus Jorgensen** wondered if anyone had an idea of the changes the proposal might cause if accepted? He thought that it looked logical, but as Zijlstra had said earlier, often it had nothing to do with logic exclusively but rather what was practical.

**McNeill** pointed out that Zijlstra had not spoken on this particular proposal; it was Demoulin who made the comment that it was a slightly different meaning. He summarised that if Art. 20 Prop A. was sent to Editorial Committee, they would be quite sure that this was not changing the application of the rule, as they had no power to do that. He assured the Section that if they thought there was a difference, they would not incorporate it.

**Nicolson** asked for a vote in favour; opposed; and to refer it to Editorial Committee? He was tempted to rule that the nays....

**McNeill** interrupted to point out that voting no did not prevent the Editorial Committee from looking at the proposal as they could incorporate it if they believed that it was meritorious and did not change anything. That was always the mandate of the Editorial Committee.

**K. Wilson** found it strange that the “yes” vote and the Editorial Committee vote were not combined. She wished to see the proposal put again with just two alternatives because she thought that the two combined would be well in the majority.

**McNeill** would have to vote “No” in that case, because he did not think this was something the Section wanted to require that the Editorial Committee look into. There had been a suggestion by Demoulin that there might be a change in meaning, which would mean that the change was not editorial.

**Rijckevorsel** just wanted to remove “binary system of Linnaeus”, which was not defined. He certainly did not want any change of meaning. He would feel a lot safer if the Editorial Committee did everything it could to ensure that no change in meaning would result.

**K. Wilson** would be quite happy to change her vote from “yes” to Editorial Committee, so that the alternatives would by Editorial Committee or “no”.

As a member of the Editorial Committee, **Barrie** thought it was safe to say that if “binary system” remained, it was very likely to end up in the glossary. [Laughter.]

**Nicolson** asked for another vote, leaving out the option of Editorial Committee. [Rumblings from audience.]

**Rijckevorsel** clarified that he should leave out the “yes”, which would be much safer. [He did.]

**Prop. A** was referred to the **Editorial Committee.**

[*The following debate, pertaining to a New Proposal in Art. 20 presented by Zijlstra regarding use of Latin technical terms in names took place during the Ninth Session on Saturday morning*.]

**Zijlstra’s Proposal (Option 2)**

**McNeill** explained that there was a proposal from Zijlstra dealing with a matter discussed *en passant* earlier in the week when attention was drawn to the rather strange issue of technical terms currently in use.

**Zijlstra** explained that the list on the screen was not part of the proposal, but was there to illustrate names that had been met with in the last few years. The proposal itself had two alternatives, of which she preferred the second, being more precise. There were two changes in each option displayed, the first was to add “Latin” before “technical term”, and the second “Latin technical term in the nominative singular”. The second change proposed was the same in both options, to cancel the word “currently” and make it more precise and instead of “used” have “in use”.

**Nicolson** felt these seemed editorial and he invited the Section to address the substance of the two proposals.

**McNeill** felt the second should be concentrated on as that was the one Zijlstra preferred and covered both elements.

**Veldkamp** objected to the use of Latin as in the grasses there was a genus *Cleistogenes* that was Greek.

**McNeill** reminded the Section that when discussed earlier *Cleistogenes* was considered an exception as there was a substantial body of grass taxonomists who wished to get rid of *Kengia* and adopt *Cleistogenes* As Latin was specified, this meant that *Cleistogenes* could be used.

**Veldkamp** remarked that he did not wish to use *Cleistogenes*.

**Nicolson** pointed out that *Cleistogenes* was not written in Greek letters but Latin ones.

**McNeill** commented that the term was English and “cleistogene”, and that the genus name was the plural. That term would then become available though there was some but not total support for this from agrostologists. However the proposal was made because one might never know what scientific term in what language might conceivably coincide with an already existing name.

**Gams** wished to consider the example of “*Paraphysis*”. If this were a fungus or red alga this was definitely a technical term, but if it was a phanerogam with just a lateral vesicle he would not consider it a technical term. Perhaps it would be useful to specify “a Latin technical term in the group concerned”.

**Zijlstra** did not accept this as a **friendly amendment.**

**McNeill** understood that Gams wished to have words to the effect of “used in the morphology of the group concerned”.

**Nic Lughadha** disliked the amendment as it weakened the proposal. For example, if she did not use a term in her group *Myrtaceae*, did that mean she could use it as a genus name? What was “the group concerned”, this had not been defined. She favoured the original proposal as it would make the job of deciding how the Article should and should not be applied easier.

The proposed amendment was **rejected.**

**Demoulin** noted there were two points in the proposal, the addition of “Latin”, and “at the time of publication”. He found the last objectionable because a taxonomist could show he had a broad botanical culture and knew what terms were used in the eighteenth century, and he did not think the *Code* should oblige people to do that kind of historical work to see if a word was used at the time or not any more. He favoured the retention of the existing Article with no change at all.

**Printzen** pointed out that “paraphysis” was of Greek origin.

**McNeill** concurred with Printzen, but observed that its usage in classical Latin dictionaries pre-dated that in botanical Latin, and it was indexed as a Latin word in Stearn’s *Botanical Latin*.

**Gereau** saw two problems in the proposal. He considered it full of redundancies and totally unnecessary under the present *Code*. Principle V stated that scientific names of principle taxonomic groups were to be treated as Latin regardless of their origin. Also, the name of a genus by definition was a noun in the nominative singular, so it was also not necessary to specify that. He felt that the proposal did nothing useful that was not already covered by Art. 20.2 and should be dismissed.

**McNeill** said that while he agreed with Gereau, that was not the judgement of one of the Permanent Committees on Nomenclature a few years ago which took the view that this was not confined to Latin technical terms because it did not specifically say so.

**Brummitt** observed that Gereau was talking about names of genera being treated as Latin, but what was being considered here was Latin technical terms. *Cleistogenes* was not a Latin technical term.

**K. Wilson** wondered why specify nominative singular and not any part of the declension.

**Zijlstra** considered the name should be exactly the same as the Latin technical term and she tried to rule out *Cleistogenes* and several other cases that strongly resemble a Latin technical term, but could not list those as she always considered them valid.

**Phillipson** felt there was another important difference between the proposal and the original wording, “at the time of publication” versus “currently in use”. It seemed to him that if a name was published tomorrow and a year later a technical term was coined which uses the name, that generic name under the current *Code* would become invalid.

**Zijlstra’s Proposal (Option 2)** was **rejected.**

**Zijlstra’s Proposal (Option 1)**

**Zijlstra** was unsure why people had voted against Option 2, whether it was because they did not want “nominative singular” or because they did not want “in use in morphology at the time of publication”. The latter phrase was added because it had been pointed out to her that without it one could have the situation where there was a good generic name and that tomorrow someone makes a technical term that is exactly the same.

**Zijlstra’s Proposal (Option 1)** was **accepted.**

[*Here the record reverts to the actual sequence of events*.]

### Recommendation 20A

**Prop. A** (13: 79: 60: 1) and **B** (18: 79: 54: 1) were referred to the **Editorial Committee.**

### Article 21

**Prop. A** (5: 70: 80: 1).

**McNeill** moved to Art. 21 Prop. A, which was not orthographical but was authored by Rijckevorsel.

**Rijckevorsel** introduced the proposal as one of the set along with Art. 32.1. He had great difficulty with the phrase “contrary to Art. 32.1”, listing two major problems. The first was the point he had made the day before that it was cumbersome and difficult to understand. The second was that it created a new category of names. He referred to an example given of a subdivisional epithet published after the name of the genus which meant that there were names for subdivisions of genera that existed in three parts and he felt that this was very unfortunate because the names could not be used, and they had two forms, one that was being used and one that was published [sic, meaning quite unclear]. His point here was that he wished to be rid of the “contrary to Art 32.1” and wanted to compare it to Art. 20.1, where it was stated that the name of a species consisted of two parts, and the epithet could consist of one or more words, which were to be united. He felt that this would be much more straightforward. His intention was that this Article, and Art. 20.4, had wording as simple and as direct as possible. He finished by saying that there was a rule in Art. 21.1 which required an exception, and his aim was to phrase this exception as simply as possible and not go through all the circus of referring to Art. 32.1 and back to Art. 21.1.

**McNeill** noted that the mail vote was 5 in favour, 70 “no”, and 80 to Editorial Committee. The point being that it was editorial, although it was based on a strongly held philosophy that you should not have “contrary to’s” in the *Code*. He reported that the Rapporteurs were not convinced that the new wording was clearer, but obviously that was something that could be looked at editorially. On the other hand, he suggested that the Section might wish to reject it.

**Prop. A** was referred to the **Editorial Committee.**

### Recommendation 21B

[*The following debate, pertaining to Rec. 21B Prop. A took place during the Fifth Session on Thursday morning with discussion of Rijckevorsel’s orthography package*. *For clarity, the sequence of the* Code *has been followed in this Report.*]

**Prop. A** (46: 64: 43: 0).

**McNeill** moved onto to Rec. 21B Prop. A. dealing with the Recommendation applying to generic names also being applied to subgeneric or sectional epithets.

The proposal struck **Gereau** as a useful extension and clarification of what was already in the Recommendation and felt that it went marginally beyond what was purely editorial, and, therefore, as a borderline case of being editorial and something desirable he wished to bring it up for support.

**Gams** felt it was just a Recommendation for everyone coining names in the future and as such he strongly endorsed it.

**Demoulin** pointed out that it was already covered by Art. 21.2 which said that it was in the same form as the generic name so he felt it was possible to consider it a Recommendation for forming generic names that also applied but he had no objection to state it more clearly.

**McNeill** suggested that the proposal was to accept it and give the Editorial Committee freedom. He felt that the Editorial Committee would want to make it even clearer than Demoulin pointed out it already was, if it was passed.

**Nicolson** asked if the vote was to refer the proposal to the Editorial Committee?

**McNeill** thought the vote should be to accept it, because it was more than just marginally not editorial, although given the point that Demoulin had made it probably made it pretty well editorial. [The vote was taken as “yes/no”.]

**Prop. A** was **accepted.**

[*Here the record reverts to the actual sequence of events*.]

### Article 22

**Prop. A** (10: 145: 4: 0) and **B** (14: 139: 5: 0) were ruled as **rejected.**

**Prop. C** (15: 86: *50: 0).

**McNeill** introduced Art. 22 Prop. C as one of the proposals, along with its parallel Art. 26 Prop. A, where the Rapporteurs had made some suggestions as to what might be an alternative wording. The alternative wording received some support in the mail ballot with 50 in favour of the Rapporteurs wording, but 86 against the proposal, so he thought that the Rapporteurs’ suggestion was not widely appreciated in the mail vote. He noted that it was a proposal that arose from the Pittsburgh Group, along with Art. 26 Prop. A, and he did not think anyone had any question as to its truism, but the Rapporteurs wondered a) if it was needed in the *Code* or b) if it should be restricted to autonyms or more generally to any names, which, again, seemed to him a truism. He invited Moore to comment.

**Moore** began by saying the issue was discussed a fair amount and he had had the same discussion with others with respect to autonyms. They thought it would be useful if Art. 22 was modified. He suggested that autonyms were odd in that you could be working in a group far away from the name that was created. He elaborated that if you were working on a particular section in a genus and only working on that section, in what might be a large genus with that section far removed from the type of the genus, taxonomically or phylogenetically, and you were the first to venture into infrageneric taxonomy in the genus, in doing the taxonomy of that section, you would automatically create an autonym for a taxon far removed from the taxa you were dealing with. He reported that the issue that came up at that meeting quite a bit, and had to be explained to the phylogenetic systematists, was exactly what was signified by the autonym, that when you established an autonym, all you were creating was the name itself. Their interpretation was that when you start working in the one area and created the autonym, all the residual taxa not included in the group, because you were dealing with only one section, were somehow circumscribed under the autonym. Consequently, you may be creating a paraphyletic or polyphyletic group. He felt that most people in the Section would see that this was not the case, but many at that meeting interpreted the wording of the *Code* in that way. He explained that that was why they thought that the Article could be placed with the autonyms, because the autonyms were unique in that a name was created for a group you were not working with. He believed that the autonyms were the only case where this occurred and that was why they had added the clarification. He concluded that if the supplementary booklet that actually explained the *Code* was ever written, then autonyms could be explained more fully there, because they were unique in that sense. As a last note he added that he would not lose sleep over it, no matter which way the vote went.

**P. Hoffmann** agreed that it should go into Stuessy’s planned booklet for nomenclature for DNA people, because it was taxonomic not nomenclatural and she thought the Section should vote it down.

**Nicolson** asked for further comments and wondered what the title of that booklet was? [Laughter.]

**Unknown Speaker** suggested that he did not have to repeat it. [More laughter.]

**Nicolson** thought discussion was on the Rapporteurs’ proposal.

**McNeill** explained that because the Rapporteurs had made the comment, and got some votes for it, it was fair that the Section should see it. They were not promoting it vigorously, but merely saying it was an alternative for the Section to consider. He supposed that technically it was an amendment to the proposal and they had put it forward in print and were not withdrawing. He added that it was simply a matter of saying that the proposal applied to all names. He noted that Moore had just spoken to the amendment by saying “yes, it does apply to all names but there’s a very special case for autonyms”.

[Unintelligible comments off mike].

**McNeill** responded that the point was that publishing any name did not define a taxonomic circumscription. He felt that the point had just been made that it need not go into the *Code* for all names, but that it would be useful for autonyms.

**Demoulin** suggested taking care of the problem presented by Moore by adding “One should be especially aware of this fact when dealing with autonyms” to their proposal?

**McNeill** thought the proposal should be left as it was and let the Section decide what it wanted to do.

**Wieringa** thought it was a good proposal, except that it would only clarify valid publication of new names and not include autonyms where you create one name and at the same time create a second new name. He suggested rephrasing it a little bit to indicate expressly that autonyms were included in the note.

**Orchard** thought there was merit in both proposals. He thought the general note was very good, but also agreed with Moore’s position that autonyms were a special case. He would be pleased to vote on both, as separate proposals to be included in the *Code*.

**McNeill** summarized that he was suggesting that the Rapporteurs’ proposal be treated not as an amendment but rather as a separate proposal, in which case, he recommended that the Section return to the original proposal and then address the new proposal.

**Prop. C** was **rejected.**

### Rapporteurs’ Proposal

**McNeill** opened discussion on the Rapporteurs alternative. [The **motion** was **seconded** and **supported** by three others.]

**K. Wilson** agreed with her fellow Australian and thought that this should be in the *Code*. She had so much trouble with students (and some practicing botanists!) who did not know the difference between taxonomy and nomenclature. She added that it was not only the molecular people who had trouble.

**Watson** agreed with Wilson and the Rapporteurs. He felt it was important to have a clear statement early on in the *Code* on the difference between nomenclature and classification.

**Per Magnus Jorgensen** also agreed with Wilson and Watson, but thought that the right place to put a Note was in the Principles. He wondered if this was possible as there had never been a Note attached to the Principles. He suggested that Principle II said what the names in the book were about, and it would be nice to point out there the difference between names and taxonomy. It was one of the first things he was taught when he entered the field, that there was a difference between names and taxonomy. He also felt that it was not only molecular people who did not understand it, so suggested that Stuessy’s book should have a new title. [Laughter.]

**Nee** thought that the intent was O.K. but the reading suggested that the person who validly published a name did not imply any taxonomic circumscription, whereas he felt that they very certainly did have an explicit taxonomic circumscription attached to that name. He thought it was ambiguous and the Section was obviously thinking only about the fact that it was valid publication, the name and the types, etc, but it could also be read to suggest that the author had no taxonomic circumscription beyond the type of that name, which was untrue.

**Nicolson** moved the proposal to the vote, but as the results were unclear he wondered if there was a third option, suggesting that perhaps it could be referred to the Editorial Committee?

**McNeill** did not think there was a third option, although the last point that was made may have some validity and the Editorial Committee may wish to consider a slight rewording. He thought it could be referred to the Editorial Committee because it was a note, but that they would appreciate a clear “yes” or “no” from the Section.

**Wieringa** suggested rephrasing the Note to include autonyms and then revote.

**Demoulin** pointed out that that was what he had originally suggested as a friendly amendment which was not accepted. He believed the best thing to do was to stop the discussion, have several people discuss it among themselves and come back later with a different wording. [This suggestion was approved after the coffee break.]

**Rapporteurs’ Proposal** was **accepted** as an amendment to Prop. C with the following text:

Following Art. 6.2 insert the following Note: “Valid publication creates a name, and sometimes also an autonym (Art. 22.1 and 26.1), but does not itself, for nomenclatural purposes, imply any taxonomic circumscription beyond inclusion of the type of the name(s) (Art. 7.1).”

### Recommendation 23A

**Prop. A** (11: 84: 57: 1), **B** (10: 84: 57: 1) and **C** (15: 81: 55: 1) were ruled referred to the **Editorial Committee.**

### Article 24

**Prop. A** (7: 87: 60: 0) was referred to the **Editorial Committee.**

**Prop. B** (4: 121: 31: 0) was ruled as **rejected.**

### Article 26

**Prop. A** (21: 89: *42: 0) was referred to the **Editorial Committee.**

### Recommendation 26B (new)

[*The following debate, pertaining to a New Proposal by Wieringa regarding Rec. 26B took place during the Eighth Session on Friday afternoon*.]

**Wieringa’s Proposal**

**McNeill** moved onto an additional proposal from Wieringa to add a Rec. 26B “While publishing a name of an infraspecific taxon that will also establish an autonym, the author should list this autonym in the publication.”

**Wieringa** explained why he thought it was important that it was added. He felt that for indexing purposes it might be very useful that indexers would know that next to a subspecies, or whatever it was, an autonym had been created, because from the date of that publication onwards it would have priority. He added that if it was in the publication that would be quite useful. He thought it would be fairly unwise to make it mandatory because people may not be aware in all instances that they were creating an autonym, because they might think that there already was a subspecies, but if it was invalid, they were creating an autonym. He did not want to fall into that pitfall, but felt that having it as a Recommendation might be very useful.

**Davidse** agreed totally with the comments that the proposer had made. In their database, Tropicos, he reported that they did keep track of the establishment of an autonym, in order to know the date, but it was often very difficult to know exactly when the autonym was created, since infraspecific names were so poorly indexed.

**P. Hoffmann** wondered if the same would not be true for subgeneric and subfamilial names?

**McNeill** agreed that it would indeed. He was going to make the comment that the Editorial Committee would have to address that as well for subdivisions of genera, not subfamilial.

**Wieringa** agreed it could be a co-Recommendation there as well. He had only put in “infraspecific” because it referred to Art. 26, and 26 only dealt with infraspecific.

**McNeill** added that a separate Recommendation under Art. 22, would almost certainly be needed.

**Wieringa** fully agreed, adding that the one under discussion might be the most important, but of course it might also be a good idea to have one for infrageneric.

**McNeill** thought the Editorial Committee would assume that was the intent. If the Section decided it was a good thing, he could not see why it would not also be a good thing for subdivisions of genera.

**Bhattacharyya** thought the Recommendation was superfluous because he argued that every taxonomic journal, like *Mycotaxon* or *Taxon* or [*Bulletin of the*] *Botanical Survey of India*, knew when they published a new species or infraspecific taxon, they compared and denoted what were the differences and what were the similarities, and it was obvious. He thought that today taxonomists were all aware of these facts. He felt it would increase the number of pages [in the *Code*] with an unnecessary Recommendation and he did not understand the point.

**Kolterman** was not exactly sure what “list” meant in this context. He thought “at least mention” would be clearer, and it would make clear as well that the author could, if he wanted to, discuss the autonym in detail.

**Basu** supported the proposal.

**Gandhi** wanted to add that the intended proposal was for future publications, because presently, or at least in the last five or six years, IPNI had been indexing all infraspecific names [of vascular plants]. He referred to Davidse’s comment, responded that, of course there were difficulties about the past, but at least not about the present.

**Barrie** commented that since it was only a Recommendation, it was not going to affect anything that had been published before. He suggested that it would read better if it said “When publishing a name of an infraspecific taxon, the author should mention the autonym” and then just delete “in the publication”.

**Nicolson** thought that was editorial.

**Watson** thought the intent was to have a declaration that the author was establishing an autonym for the first time. In which case, as it stood, he argued that all that had to be done was mention an autonym was created, not that this was the first time it was created.

**Moore** wanted to point out he supported the proposals for the reasons he stated earlier. He felt that the business about establishing a date for the autonym was not that important because they had priority over other potentially competing names irrespective of the date they were established [Art. 22.1, 26.1]. He felt the proposal was about making it clear that someone was working on one taxon and they created an autonym in a taxon that they were not working with.

**Wieringa** did not agree that it always had priority because if the species were lumped within a second species then the autonym did not automatically have priority. He argued that it only then had priority from that date onwards, when the other name, the other subspecies, was created, so it was important what the date of an autonym is.

**McNeill** assured the Section that as the wording dealt with the taxon, the Editorial Committee would ensure that it was also reflected in the appropriate place for names of subdivisions of genera [?new Rec. 22B].

**Wieringa’s Proposal** was **accepted** and insertion of a similar Recommendation following Art. 22 was referred to the Editorial Committee.

[*Here the record reverts to the actual sequence of events*.]

### Article 29

**Prop. A** (11: 140: 3: 6) and **B** (9: 141: 3: 3) were **ruled rejected.**

### General Discussion on Electronic Publication

**McNeill** moved onto Art. 29 Props A and B, both from the Committee on Electronic Publication and both received more than 75% “no” votes, so would be ruled as rejected unless someone wished to speak to them, which he was confident someone would.

**K. Wilson** wished to speak to the proposals [The **motion** was **seconded** and **supported** by three others.] She requested that the matter be discussed because of the importance of electronic publication to the future of the *Code*. She thought that the proposals the Committee had put up were likely to be rejected as were the proposals at the previous Congress, because people were so weary of archiving. She thought that a discussion of what was acceptable in electronic publication was needed because the Section had to face the fact that the technology was here to stay. She noted that there was already at least one example of a name published under the botanical *Code* first in an electronic paper, *Psilocybe aesurescens*. She reported that the way that the *Index Fungorum* dealt with it was to print out several hard copies, get the author to sign and date them and put them in several libraries to validate the publication. This was because the name had already been cited, according to Paul Kirk, by several thousand people before they became aware that it was not available in hard copy. She felt that the Committee, as the Rapporteurs had pointed out, were divided, but that they were divided in the way in which they should propose Electronic Publication, there were some that opposed it altogether but most were in favour, but they favoured different methods. So they had provided two alternatives, neither of which was acceptable. What she wished to propose instead was that they came up with a new proposal, after talking to several people and offer it when new proposals were considered. She hoped that in the light of a short discussion now, 10 minutes or so, so that they could discover what was acceptable to people generally. She suggested they could present a different proposal that included the specification that a certain number of hard copies be distributed to libraries. She pointed out that there were already electronic journals, such as *Biota Neotropica* of Brazil, which were only online, but provided hard copies, she thought 14, to institutions listed on their website, to validate new names because they were dealing with both plants and animals. Similarly, under the zoological *Code* they had taken a very general view and proposed only principles as to how to deal with electronic publication. She added that there was a new journal called *Zootaxa* and another called *BioOne*, which handled it by printing out the pdf they put on their website and depositing the copies in libraries. She thought that if the zoological people could do it, she did not see why we could not come up with a similar system in botany. In her view and in the opinion of quite a few other people she had been talking to, it was important to make sure that the *Code* was not bypassed by people, and that was becoming increasingly likely. At the same time she stressed that the Section must make sure that the system was stable for as long as needed. She quoted one of the Committee members as saying that botany had been going 250 years and we needed to be sure that anything we produced could be archived for at least as long again. So she requested some comments that might be incorporated and that anyone with strong feelings talk to her afterwards.

**Nicolson** proposed setting aside 5 minutes and then deciding if more time was needed.

**Bhattacharyya** made the point that large numbers of people reside in Bangladesh, India and China and most of them have no electronic media. He suggested that electronic publications were also not available in the remote areas of India, Bangladesh and Sri Lanka, and Africa. He did not understand how, in this situation, the developed countries could demand a common standard for electronic publication.

**Per Magnus Jorgensen** felt that this was the most important issue at the meeting and more time should be spent on it. He fully supported what Wilson had said. He felt that he knew too little about the technology in the new publication media. There were two principles in this Article that he was adamant must be secured; widespread availability and the stability of published matter. He acknowledged that the first was perhaps not as good as those in the West thought. He also did not know how stable the electronic journals were. He supported the idea that printed versions should be deposited somewhere. He fully supported Wilson’s attempt to come up with a new proposal and felt that the Section could not leave without some resolution.

**Skog** reported that the fossil plant Committee had had a great deal of discussion back and forth on electronic publication. She had a long list of criteria that the Committee would like to see included in any Article. She asked Wilson to see that they were consulted about any forthcoming proposals.

**Gams** returned to the same example of *Matrushima Mycological Memoirs*, that had been cited; he felt that it became essential that hard copies of a paper be deposited to avoid any instability of electronic media in the net or CD’s. He proposed a friendly amendment to the proposal under item three: “identical copies [on paper]” and delete “...electronic or both electronic and...” from the proposal.

**McNeill** thought that his suggested wording should be submitted to Wilson, who would be producing a new proposal. The published proposals had clearly not been favoured in the mail vote. He reiterated that this was a general discussion on the topic of electronic publication and that the original proposals had been withdrawn in favour of the new proposal, to be written.

**Demoulin** thought that the vast majority of people were aware that permanence required the deposition of paper copies and he was sure that Wilson would follow that line. He suggested that it could be very simple, just reaffirm that effective publication can only be linked to the deposition of hard copies in libraries. He added that this did not prevent those who wanted large, fast distribution from using electronic media but felt it was something aside from effective publication. He suggested that if the Section go into detail on what was acceptable or not, as the Rapporteurs had said, by the next Congress everything may change again. He advocated simplicity; explain that effective publication was paper publication, but that did not restrict the use of varied methods of dissemination of information, once the requirements for effective publication were met. The discussion reminded him of that on living types; just because that specimen was need as a permanent type, did not prevent people with cultures from gathering more details about their organisms. There, the problems were with people who wanted just living types and did not want to bother with depositing a dried one whereas here there may be people who did not want to deposit a paper copy and those people must be made to understand that they could not do that.

**Basu** suggested that those with the facilities for electronic media would do it, but those without the facilities would follow the traditional methods of manuscript, hand drawings, etc.

**Marhold** understood the problems for developing countries because the country he came from was sometimes in a similar position. He thought it was important to compare the availability of electronic access to the availability of existing, printed journals. He argued that these were becoming more and more and more expensive and his experience was that the countries with limited funds opted for the electronic journals anyway. He was in favour of the proposal. On the other hand, he was always thinking of the speed of the development of technology, noting that is was not that long since we used floppy disks. He would insist on putting printed versions in libraries. His idea was that it would be better if distributed among libraries in several countries rather than in a few libraries in a local district.

**Nicolson** pointed out that the discussion had used up the five minutes allocated and asked if it was the wish of the Section to continue the discussion? [It was.] He allowed continuation of the discussion.

**Schafer** thought it was very important to maintain a reasonable amount of paper copies. In the *Code*, we have forbidden theses because they were not widely distributed. He did not think it was reasonable to have just a few paper copies and emphasized that the copies should be widely distributed, and distributed by open access on the web.

**Dorr** was very sympathetic to the problem because in Yokohama he had first proposed the establishment of the Committee. Although he had abstained from the discussions over the past few years, he had read them all and several of the comments disturbed him greatly. One was the impression he got that most of the Committee members felt that depositing two copies on paper of any electronic publication would satisfy the *Code*. He thought reducing it to such a small number was meeting the letter but not the intent of the *Code* and felt that the intent was that all of those interested could find the material again. He added that even with paper copies, this became very difficult as many of journals have relatively restricted runs. He gave the example of *Brittonia* where perhaps only 600 copies were distributed throughout the world. Yet he argued that that permitted most to gain access to those copies. He thought that the Section should be extremely careful about suggesting that there was some minimum number of copies that would satisfy the requirements because it was a great chore for people to find publications at times. His second point was that he also felt that no proposal should refer to proprietary software or any other sort of external, commercial process. He remembered at the last Congress when discussing theses, people suggested that ISBN numbers, over which the *Code* had no control, would be the controlling factor. He felt that it was the same here: pdf files were proprietary software, CD’s, DVD’s whatever; he pointed out that today many of the audience members had memory sticks hanging around their necks but by the next congress, they may all be obsolete. He thought it was a difficult issue, that had to be addressed. He was not convinced that the proposals as they were written were the solution but did not know what the solution was going to be, just that it had to be one where many more copies were available to everyone.

**Nicolson** summarized that there were two fundamental points to the communication of new information. One was dissemination, making it widely available to many parts of the world the second was being able to go back to the original publication and see it 100 years. These were the two needs: communication and access.

**McNeill** hoped that the group would come up with something that was acceptable. He agreed with K. Wilson, in particular, about ensuring that what was going to be the normal means of scientific publication within a very few years was not one that considered the *International Code of Botanical Nomenclature* irrelevant to it. He also felt that electronic media, despite the problems described for the Indian subcontinent, were going to be more readily accessible than hard copy in many parts of the world. He knew of one long-standing, classical journal for which there were a number of years of back issues that had been printed but were not being distributed because the institution lacked the funds for mailing. He felt there was one point that the Committee had to address; he totally agreed with Dorr and others that the spirit of the *Code* was to ensure the widespread dissemination of descriptions, of the printed material for new taxa. However, he pointed out that that the letter of the *Code* included no statement of number. He elaborated that the debate went back to the *Cambridge Rules* of 1935, but there had never been agreement. All that the *Code* said was that the publication must be distributed to botanical institutes, in the plural, meaning two. He suggested the new proposal may want to try to amend that as it well. Although he added “Good Luck to you”, because attempts in the past had been unsuccessful.

**K. Wilson** thanked the Section for the discussion time and helpful comments and asked those interested to meet up over lunch to discuss the new proposal.

**Prop. C** (8: 140: 10: 3) and **D** (6: 146: 6: 3) were ruled as **rejected.**

[*The following debate, pertaining to a set of New Proposals by K. Wilson regarding electronic publishing took place during the Ninth Session on Saturday morning*.]

**K. Wilson Proposals 1–5**

**McNeill** reminded the Section that although the proposals on electronic publication had been heavily defeated, the Section had agreed that the group interested in the matter should come back with fresh proposals that might prove more acceptable.

**K. Wilson**, spokesperson for the group, displayed the proposed new wording on the screen, and copies had also been handed out. She felt that electronic publication was the most important challenge facing the Section that week as it already existed and was increasingly being used by journals. The challenge was to integrate electronic publication into the *Code*, proceeding slowly step by step, and hopefully taking the first step. The Special Committee on Electronic Publication had now existed for two terms. The proposals it made to the St Louis Congress were not accepted, and neither were the two made at this Congress. Contrary to the Rapporteurs’ comments, most members of the Committee were in favour of electronic publication but differed in how this should be implemented. The two proposals addressed two different ways of electronic publication, which with retrospect, would have been better not to emphasize technical methods but concentrate on the principles; this is what was done in the zoological *Code.* The two proposals received a heavy “no” in the mail ballot and had been discussed earlier in the week. The main concern for a wide range of people here and elsewhere seemed to be the matter of how to archive electronic publications. This was a valid concern, although equally there was no guarantee of archiving in perpetuity for paper-based publications.

She reported that during the week, an *ad hoc* committee had discussed what approach might be acceptable. [List of participants shown on an overhead.] She thanked the group and many others who had contributed during lunch-time discussions and other times, often over a cool ale. She was now presenting fresh proposals on behalf of the group. They were all independent, but would allow the *Code* to proceed in an orderly fashion towards the eventual acceptance of electronic publication. She emphasized that it was a very important matter and not just in the future as the electronic publication of names was already happening whether the Section liked it or not. She mentioned again the case of the new fungus *Psilocybe azurescens*, which was guaranteed to be a well-known example because of its properties which were not preservable in a type specimen. When *Index Fungorum* became aware that the *Psilocybe* name was only electronically published, it printed out two copies of the paper and deposited them in two libraries. That was a very minimal paper publication but was adequate to satisfy the *Code*’s current provisions on effective publication. Paul Kirk, who would have been here but for his continuing back problem, had said that *Index Fungorum* was prepared to do the same in the future if it had to; that is to deposit copies of the paper signed and dated by the author in two libraries to avoid problems of electronic publication alone. Paul was very well aware that this was a stop-gap measure, to do this rather than to leave the name *in limbo* because it was only published electronically. So which way were the group suggesting the *Code* approached electronic publication? The zoological *Code* accepted electronic publication only on distributable electronic media, that is currently CDs, DVDs, and the question of USB disks would surely come up soon, but excluded online publication. However, scientific periodicals were leading the way in addressing issues of availability and stability of online electronic publications, and the group believed that online publication in scientific periodicals was the way the *Code* should approach electronic publication for the moment. Besides the journals there were other initiatives addressing archiving issues, including the new Mellon Foundation project specifically addressing the issue of archiving electronic scientific journals.

The five proposals made by the group aimed to introduce electronic publication online as an adjunct to hard copy effective publication, with online publication only in periodicals. The hard copy would still remain the basis of effective publication. The proposals guided the *Code* in an orderly and safe way towards effective electronic publication, so indicating to the rest of the world that the *Code* was moving to embrace the technological advances that were widely accepted in the scientific and broader community. She wished to see the proposals discussed in turn, as they were independent.

**McNeill** thought that the proposals should be taken one at a time and the President concurred.

**K. Wilson Proposal 1**

**K. Wilson** stated that the first was only a very minor change to the existing Art. 29.1. The present *Code* excluded publication online or by distributable electronic media. The feeling was that that it would be better to say “any form of electronic publication alone” to better emphasize what was intended without specifying any one form as that could become obsolete exceedingly quickly.

**Redhead** pointed out that with the suggested wording, if there were two forms of electronic publication they would not be “alone” and so be acceptable. It did not specify one must be a printed copy.

**K. Wilson** agreed he was interpreting the wording differently. The intent was that “alone” meant without hard copy.

**Redhead** pointed out that if he could interpret it like that, someone else might, and that was his concern.

**Rijckevorsel** suggested replacing “alone” by “merely” and earlier in the sentence to avoid such misreading.

**K. Wilson** first accepted this as a **friendly amendment**, but later felt it was better voted on.

**Barkworth** felt rewording was not necessary as the second line in Art. 29.1 specified effective publication was only by distribution of printed matter. This meant there had to be printed matter and the proposal could not be read as allowing two forms of electronic publication.

**Norvell** wished to amend the amendment to say “or solely by any form of electronic publication”. [This was accepted as a **friendly amendment.**]

**Nicolson** called for a vote on the that amendment, which was **accepted.** The original proposal as amended was then opened for discussion.

**Watson** felt this was entirely editorial as the Article did not say “solely by... ” before microfilms, or before typescripts in the current wording and he felt it was not needed.

**Nicolson** agreed that if passed this could be looked at by the Editorial Committee.

**Nee** was bothered by the word “publication” at the end of the paragraph since its use was not the same as that of “Publication” as the first word of the paragraph. Electronic “publication” was really distribution, dissemination, or some other word, but he was not sure what.

**K. Wilson**, in answer to a question from Nicolson as to whether that was acceptable as a **friendly amendment**, felt it should be discussed and not simply accepted.

**Davidse** spoke against the amendment as he felt the *Code* was leaning towards the whole idea of electronic publication, so felt that should be left in as the Section was trying to lay the groundwork for the possibility of total electronic publication sometime in the future.

**Knapp** thought that what was meant was “electronic publication” the noun, and not “electronic publication” the verb.

**Nic Lughadha** agreed, but suggested a **friendly amendment**, to use “by any exclusively electronic form of publication”.

**Dorr** felt it was difficult if everyone tried to edit this but thought what was being talked about was the distribution of electronic materials. He agreed with Nee that “publication” should not be used because it was inherently contradictory if we were saying that publication was only by printed material. What was being referred to was the distribution of names in an electronic format, and not accepting those.

**Kotterman** felt that in any case if the word “publication” was left in it would have to be taken into consideration when the glossary was prepared, because if publication was defined as normally understood in the *Code* and it was used differently at the end of this phrase, it would cause a great deal of confusion.

**McNeill** considered it very unwise for the whole Section to try to edit the proposal, though he admitted to doing this himself. The point Knapp made was very reasonable provided the context was clear. The first sentence “Publication is effected” was not a definition of “publication” but of “effective publication”, and later on “any form of electronic publication unless accompanied by printed matter” spelled this out, and this or some of the other suggested wordings might be something the Editorial Committee could use. The minute there was a move to “dissemination”, he felt the point the proposers wanted was being lost. There was a wish to have electronic publication referred to in the *Code*.

**Bhattacharyya** commented that “Publication” in a dictionary definition meant things coming to light in a printed form, but with electronic media there could be hard copy or soft copy, so “electronic publication” was not an appropriate word for effective publication in the *Code*.

**McNeill** asked for clarification as to whether the replacement of “publication” by “dissemination” was a formal amendment. [This was moved and **seconded.**]

**Rijckevorsel** wondered if, as “distribution” was already used in the paragraph, it might be better to use it again instead of “dissemination” as it was unambiguous.

**Nicolson** believed this to be an editorial suggestion.

**Baum** suggested the replacement of “dissemination” by “media” as a different amendment.

**Nicolson** pointed out that in order to proceed further, there should first be a vote on the amendment to the proposal Nee had made, to replace “electronic publication” by “electronic dissemination”. [The amendment was **rejected** and Baum’s proposed amendment was opened for discussion.]

**K. Wilson** felt that because “media” tended to be used for distributable material such as CDs and DVDs, then was more risk of creating problems and of people being confused. She preferred “any form of electronic distribution” or thought “exclusively any form of electronic distribution” would be close to what was needed.

[The amendment to use “media”, being seconded, was then voted and **rejected.**]

**K. Wilson** returned to the original proposal, and indicated that she would be happy to see “electronic publication” replaced by “electronic distribution” as that reflected the mood of the Section.

**Nicolson** accepted this as the proposer’s own amendment and called for a vote.

**K. Wilson Proposal 1** was **accepted.**

**K. Wilson Proposal 2**

**K. Wilson** introduced this as the key to lead the way forward into electronic publication, hopefully at the next Congress. It did not change anything, because it still said that only hard copy effected publication, but set out the kind of conditions that must be met for an electronic publication to be regarded as equivalent to the hard copy version. Points 1–5 of the conditions in the proposal were what the *ad hoc* group had agreed on. The sixth was an **amendment** that Lack suggested and should be dealt with separately.

**McNeill** agreed the last was an amendment and instructed the Section to ignore the sixth condition for the moment.

**K. Wilson** felt the points were self-explanatory, and explained that the fifth was there as geological journals were refusing to mention nomenclatural novelties in abstracts. To have this would mean such journals could be shown this was a requirement.

**McNeill** pointed out that this was not an Article as it did not change anything, and there was no need for the electronic versions to be published on an independent platform, or for electronic versions to be identical, so long as there was a printed version when Art. 29.1 applied, but he fully understood the desire of the group to have those sorts of words in the *Code*. He explained that the date was unnecessary as there was no limiting date, the second part was a Note emphasizing that it was possible to publish in a journal that was distributed electronically, provided that there were also printed copies. He felt that the material that followed would be better as a Recommendation, and he felt that it was perhaps logical to link Point 5 with the latter part of Point 2, because Point 5 was quite dramatic in not recommending publication anymore in journals which do not have an electronic version.

**K. Wilson** was inclined to agree and indicated that the group had considered putting this as a Recommendation, and was unsure if a Note was appropriate.

**McNeill** explained that a Recommendation could be ignored, but that a Note could not. A Note explained something in the *Code* that might not be self-evident. He was worried that by saying “solely by electronic publication” the group might be damning that, and it could emphasize through the Note that electronic publication was perfectly acceptable so long as there was also printed copy.

**K. Wilson** felt that in that case Point 3 could perhaps be united with part of what was under Point 2 if that was all accepted, and would be happy to see this done in that way. None of the group present indicated they objected to that.

**McNeill** felt the general discussion in the Section should not focus on the details, and assumed that the technicalities he found difficult were accepted as resolvable, as he was sure was the case. He emphasized that it was important to know what the Section wanted with respect to the particular things which should or must happen.

**Dorr** appreciated the comments about what should be a Recommendation or Note, but had two concerns. First, he pointed out that some botanists published novelties in Floras and not just periodicals, and secondly while it might be a necessary to have a mechanism to specify mentions in abstracts for some geological journals, not all publications had abstracts. He felt it would be unwise to imply that not having an abstract in some way invalidated a name.

**Chaloner**, as one of the supporters of the motion, wished to make a very general statement. This clearly was the thin end of a wedge. He did not like the fat end of that wedge, but accepted that the thin end was appropriate to take on board at this moment. The thin end of the wedge was the phrase “the electronic version to be regarded as part of the distribution of this work”. It was Wilson’s intention, and that of some of her colleagues, that it become not merely a part but the whole, at the next Congress maybe if they were lucky. He was not too worried, as though he did not like the shape of that wedge, wedges could be cut off. He saw an interesting analogy with, for example, registration, as it came to be handled in St Louis; the thin end of the wedge was started in Tokyo but was cut off. If electronic publication did not take the glorious course some saw, then it could be cut off too. He was in favour, warmly, but with some reservation. He felt that there were a few things, like birth and marriage certificates, that should be on paper, and that this should also be the case for descriptions of new taxa. With respect to novelties appearing in geological journal abstracts, he saw no objection to the phrase that the presence of nomenclatural novelties must be stated. He could see no journal objecting to an abstract saying “ten new species are described in this paper”. What geological journals did not like was to have the new names themselves in italics in the abstract for the very good reason that the abstract in many of those journals goes out ahead of the journal itself, maybe even in a different year, so most very rightly did not want the new names in the abstract.

**Gams** made a minor editorial suggestion, that it was not possible to allow publication from a specified date as it was already happening. He argued that the point was establishing what was necessary for [electronic publishing] to be recognized as effectively published.

**Buck** felt the date was irrelevant as long as there was printed copy, and pointed out that many journals put the electronic versions up prior to the publication of the printed version, but with the understanding that the printed version was the effective one. He also agreed with Dorr that many books and Floras did not have abstracts and suggested changing “must” to “should” to take care of this.

**K. Wilson** wished to clarify that the issue of abstracts only related to journals, and indicated that she had yet to see a journal that did not have an abstract as a part of an Article. Floras were a different matter and she said they were not trying to stop people doing what they wanted in monographs. The safe way forward with electronic publication was with journals and not with Floras, monographs, or whatever. There was no intention to stop people from publishing wherever they wanted. They were only saying that if you wanted to move to electronic publication of names it was suggested to do it through a journal, not in any other form of electronic publication.

**McNeill** felt that what the Section should be making a decision on was whether or not the fundamental Point 5 was acceptable, because if that was the case, it would then become relevant to explain how journals with parallel electronic and printed journals should be constructed or presented.

**K. Wilson** did not like the idea of Point 5 being linked to Point 2.

**McNeill** stressed that unless the *Code* were recommending the use of electronic journals, there was no need for the detailed Recommendations. He thought that could change if it was suggested that the date of publication, as implicit in Point 2, might be that of the distribution of the electronic version, but that was not the case at present. He added that the proposed recommendation in Point 5 indicated something completely new in the *Code*, but the Note could be very brief. Point 2 needed to hang on something, and that could be the proposed recommendation in Point 5. He reiterated that Point 2 was not an Article. He did not consider telling electronic journals how to handle themselves was any part of the Section’s business.

**Demoulin** observed that the Section had heard that the vampire of electronic publication might rise from its grave at the next Congress. He agreed with Chaloner’s suggestion, and recommended that those present sharpen their spikes to kill it now. More seriously, the situation was somewhat similar to that of living types where for a long time people felt they were prevented from working the way they liked, and the conflict was somewhat softened when Rec. 8B.1 acknowledged that cultures were useful in so far as the requirement for a permanent type was satisfied. He agreed with McNeill that Point 2 could not be an Article, also because it introduced “distribution” as a nomenclatural concept which would need to be defined. He argued that what was needed was a Note and a Recommendation showing the Section acknowledged the usefulness of electronic dissemination, especially under certain conditions. This was not nomenclaturally different from what was done when reprints were mailed to colleagues so that they are received earlier than journals.

**Gandhi** indicated that as a part of his job he went through all new books and journals that arrived in the library, and he assured Wilson that he had seen a number of articles without any abstract or key words. He found the citation of names in abstracts wasted less time if the family and not only the name of a new genus was mentioned, but this must not be part of an Article.

**C. Taylor** commented that the reality was that people were already publishing new names in journals with electronic and paper versions, and that the electronic version was often the most used around the world. She was not concerned how this was included, but felt it very useful for the *Code* to authoritatively explain what the important elements were as this would also help prevent confusion between paper and electronic versions.

**Hawksworth** was concerned over the phrase “prior to or simultaneously” as the world’s major scientific publishers were already publishing identical copies online well in advance of the hard copy, and printing the dates papers were published online on the printed copies. Surely such works had to be regarded as effectively published on the date identical copies of the later-to-be-printed versions were placed online?

**McNeill** concurred, but to treat the electronic medium as having the date of effective publication was not the proposal now before the Section.

**Hawksworth** added that there would be a requirement for hard copy to be subsequently deposited. He stressed that this was the way to go and needed to be done now and not to wait. Not to adopt this approach seemed very strange to those routinely working with electronic journals, especially in microbiological groups such as yeasts.

**Alford**, addressing the issue as to whether or not this had to be an Article, felt it would be as there was a change effected. He explained that presently Art. 29 stated that material needs to be distributed “to the general public or at least botanical institutions”, implying at least two. If Point 2 was accepted, he envisioned that a person preparing a personal electronic journal might print and deposit one copy in his or her personal library and distribute it otherwise only by the worldwide web. One copy plus electronic copies that followed the proposed rules would then constitute effective publication, which the Section would surely find undesirable.

**McNeill** confirmed that Alford was perfectly correct as the proposal did state “a printed version”, and it could be just one. There was a difference but he did not think this was the thrust of Wilson’s proposal and she did not intend to permit a single printed copy.

**K. Wilson** indicated that that was definitely not what was intended. She clarified that the existing *Code* should be followed, but they were also suggesting an amendment to the existing arrangements to try and stop people depositing only one or two copies, ten being suggested in Point 4. This would affect all printed publications as it stood at the moment. She maintained that it was trying to set the way to make sure people in the future did not just deposit one printed copy as there had to be some minimum number of hard copies for the publication to be effective.

**Gandhi** reminded the Section that the present Rec. 30A.1 cautioned authors against publishing just in electronic media. Prior to the St Louis Congress a checklist of the North American flora including 35 new combinations had been circulated electronically, assuming that the Congress would support electronic publication. He reported that as it did not, afterwards, identical electronic and hard copy versions were circulated and there was confusion as those who did not receive hard copies thought the combinations were invalid.

**Briggs** noted that it appeared that in the proposed Note, what was covered in the first sentence really did not apply, but the last sentence of the first paragraph, “for the electronic version to be regarded as part of the distribution of this work” and then the five points under that might well be useful as a Note. That sentence and the five points were the essential parts of the proposal and alone might be sufficient. She wondered if removing the first sentence would be taken a **friendly amendment.** [This was later accommodated by Wilson].

**K. Wilson** agreed this should not be an Article, and felt the whole would be better as a Recommendation starting, “For those publishing names in periodicals it is recommended that... ”, and leading into the rest of the material.

**Watson** was concerned at Point 5, as it meant as it currently stood that all authors should publish in periodicals using electronic and printed versions. What it should say was that “Those who publish names in periodicals should as far as possible use periodicals that produce print and electronic versions” and then continue “The electronic version should... ”. That would be meritorious and he would support it.

**McNeill** pointed out that there were two ways these details were appropriate. One was just outlined by Watson, and the other was enunciated earlier by Hawksworth where it would go back to being an Article. If it were intended that the words “for the electronic version to be regarded as part of the distribution of this work” meant that the date of publication was whichever was the earlier date whether electronic or printed, he argued that that would be a huge change and then all the requirements suggested would be appropriate and necessary, but that was not up-front although implied by Hawksworth. He was not sure which option the group preferred.

**K. Wilson** put up some revised wording which addressed the point that this proposal was only for people publishing names in periodicals, and dealt with the “prior to or simultaneously” issue raised by Briggs, now stating “a printed version as well as a matching electronic version”.

**McNeill** wondered why it mattered for both versions to be considered as “part of the work”. It did not seem relevant to the *Code*, unless it was a part of the work in the sense that it determined priority? If the electronic version went out first would that determine the date of publication?

**K. Wilson** conceded that “matching” was perhaps not needed as long as “identical”, etc, was there, but was adamant that the issue of priority was not the intent. It was important to establish some kind of principles for future electronic publication, but she deferred to him as to the best way to do this.

**McNeill** was not objecting to the content, and the “musts” would become “shoulds” in a Recommendation, but simply saying that these could be criteria for the type of periodical in which people were being recommended to publish their novelties. But there was a weasel word, the business of it being part of the distribution of the work, as the only reason that could have any relevance was if it affected the effective date of publication.

**K. Wilson** indicated that it was not the intent to affect the date of publication, because that must be the hard copy, but that it was a question of making the work widely available. If the Section could think of a better way to express the desire to have electronic publication as a way to reach a wider audience, not all as she was well aware that hard copy was essential in some places, but for many people these days electronic copies were easier to get either through the journals or from authors themselves.

**Hawksworth** felt the Section was losing touch with what was happening. What people work with now is up-front publication online, they do not wait three months while something arrives by surface mail. The works are there, identical to the printed copy, in the electronic versions. Further the electronic versions were being archived by many major publishers. He considered that the Section had to make electronic publication effective at this Congress, and that it was unacceptable to leave this for another six years.

**Nicolson** commended Wilson and her group for attempting the difficult task of getting a new idea into the *Code*, and in language that was acceptable. He wondered if the Section would like to continue discussion or not, and asked for a vote. The result was not clear, so he suggested discussion continue to coffee, but requested that speakers try to cut to the chase.

**Rijckevorsel** felt the point was whether electronic publication had any status whatsoever, and was much of the same mind as Briggs. He would like to move an amendment that the second sentence should be an Article with points one and two. This would mean that electronic versions had some status under the *Code*, but only a minimal one as being faithful copies of the really important printed versions.

**McNeill** enquired as to the meaning of “some status”, how it implicated other Articles of the *Code*, and asked what this did for a name?

**Rijckevorsel** stated that this did not affect the name or priority or any other Article of the *Code*, but gave it some very minimal status in that electronic publication was mentioned.

**K. Wilson** did not accept that as a **friendly amendment.**

**McNeill** made the point that it was the decision of the Section whether to make it an Article or not, and he would have to interpret that as doing something. He felt that if it did anything, it would establish the electronic version as being equal to the printed version, and affect the date of publication.

[The amendment was **rejected.**]

**Knapp** recognized that there seemed to be a problem with the sentence regarding the conditions for an electronic version being regarded as a part of the distribution of a work. What she thought was intended was to recommend what sorts of simultaneous electronic journals taxonomists should be thinking of publishing new names in, not what journals should do or where people should publish. She had been approached by BioMedCentral and the Public Library of Science with regard to setting up an electronic taxonomic journal. She had told them this was not what was needed at the minute as the Section had not worked out what it required. Having this Recommendation to taxonomists in the *Code* as to what sorts of journals were acceptable for publication was valuable and would be noted by the journals. She suggested striking the sentence relating to the electronic version being a part of the distribution as that clearly had subtle meanings that could be interpreted in different ways as McNeill had said. Consideration should also be made to allow for electronic monographs, but the requirements really applied to periodicals. This was more a Recommendation to taxonomists of things to take into account in considering what type of electronic journal to publish in should that become much more prevalent than it was today.

**McNeill** felt there were two very important matters to resolve. The way forward Knapp had suggested, and another way – to make the electronic medium part of the publication in the sense that it determined the date of publication. He had the sense that the last view was a minority one, but the Section should be aware of this. He added that the two were not mutually exclusive.

**K. Wilson** suggested some alternative wording for the struck out sentence on the screen, which could be editorially improved, “The features for such a periodical should be... . ” and points one to five.

**Knapp** suggested “periodicals, preferably those that regularly publish taxonomic articles” and that separate works such as monographs should also be allowed for.

**Atha** reminded the Section that journals that are deposited in public libraries were freely available through inter-library loan and go out to anyone who asks for them, while electronic journals do not go out freely through any type of loan process. He was concerned that taxonomic publications might become hostage to journals that did not allow sharing.

**Demoulin** felt the wording was getting better, now that “part of the distribution” had gone, but it was still a Recommendation to publish in periodicals more than in other media, and periodicals that had an electronic version. He found this totally inadmissible. A way had to be found to make it clear that the new Recommendation was only for those who wanted electronic distribution, and not to specially recommend the use of electronic distribution.

**Wieringa** proposed an amendment, to change the fourth Point to “the date of publication of the printed version should be stated in the work”.

**Nicolson** requested that the amendment be held for the moment and that he would come back to him later.

**Nee** drew attention to a phrase nobody had questioned, “periodicals, preferably those that regularly publish taxonomic articles”, and wondered whether this adequately outlawed newspapers like the *New York Times* that also had electronic versions.

**McNeill** replied that the issue of publication in newspapers and ephemeral works was already covered elsewhere in the *Code*.

**Mabberley** suggested that to take care of the point raised by Demoulin, “electronically” be inserted after “publishing names” in the first sentence. This was necessary as otherwise it looked as though the Section were insisting people publish electronically.

**McNeill** suggested the *ad hoc* group meet during coffee, and that Art. 29 be returned to immediately after the break.

After the break **McNeill** reported that the group had met again and had prepared some matters to address on the screen. They had recognized that there we two issues, and there was a proposal for Note 1, and an amendment to it which addressed the second issue, namely whether or not the date of publication should be that of the earlier of the electronic or printed medium. As that was an **amendment** he suggested the Section should probably take that first. He understood it had been seconded.

**K. Wilson** felt the amendment was self-evident. It was not being moved by the group but by someone else and agreed it should be addressed first.

**Atha** pointed out that the *Code* said that effective publication was only in printed form, and that anything that deviated from that was a complete contradiction to what was in the *Code* now and had to be voted on in that way and to be either a completely new Article or rewriting of that Article.

**Hawksworth**, speaking for the amendment, added that in Art. 29.1 as revised, the matter raised by Atha was already taken care of as it made clear that an only electronic medium was unacceptable. The issue here was really just a matter of the date, and whether the Section wished to recognize the actual situation in publishing, that what people used today was what they got online, and there was no question that was when the material was actually distributed in practice.

**Kotterman** wondered whether this should be an amendment to Art. 29 or to the next Article that dealt with the date of effective publication.

**Nicolson** felt that might be an editorial matter.

**Demoulin** sympathized with the idea that the information about a new name might come first to many people by electronic dissemination, but he did not see this as adequate for the reason that the deposit of printed material must be the date and also because of the problem of how in the future the date of dissemination would be determined. It may be indicated somewhere, but copies may be bound in libraries, and in 50 years nobody would be able to find what the electronic date had been. He accepted that many journals had dates printed on them, but could these be accepted at face value when dates on many journals had printed dates that often proved false. The *Code* had always accepted as the date of effective publication that on which a journal really became available. This would be a big departure from what had always been done, and he could not accept it.

**Eckenwalder** pointed out that the phrasing assumed that the electronic publication would be the earlier, but that was not an absolute necessity and should say whichever was the earliest.

**Norvell** wished to make a **friendly amendment** in that regard, to switch it to “whichever of the two was earlier”.

**Wieringa** was very much against the proposal for the simple reason that if somebody published something electronically now and did not print it now it would be invalid, but if someone decided suddenly to print it in 2080 the publication today would retroactively be effective, and that was definitely not wanted.

**Nic Lughadha** requested that the Section think about indexers and the services many of them used for free. Would indexers then be expected to check two dates for each publication to decide which was the earlier? That would add an unnecessary burden for no great advantage.

**Lack** wished to make clear that the amendment was definitely not the position of the *ad hoc* group.

**Demoulin** felt the situation could be similar to things which had for a long time been in the *Code* relating to the date of dissemination and effective publication. If the next week someone at the Congress had a poster with a new taxon, it would be known by a large number of botanists and have a wide dissemination, as may occur with the electronic version of a journal, but the *Code* specifically outlawed the presentations at scientific meetings. He thought the situation was exactly parallel.

**Zhu** wished to draw attention to a special case. The *Flora of China* was published as both hard copy and online versions, and did include novelties. However, the idea behind the online version was that it could be changed, and this happened all the time. Also, most manuscripts appeared in the online version earlier than the date on the printed work.

**Glen** felt there was a logical flaw in the amendment. His understanding of effective publication was that it was the date when all requirements of the *Code* were fulfilled. Before coffee the Section had voted that one requirement was a paper copy. Therefore, if online publication were earlier than the paper copy all requirements would not have been met, and the Section would be contradicting itself. He would vote against the amendment.

**K. Wilson**, commenting on the situation with the *Flora of China*, pointed out that the amendment only applied to periodicals and not other kinds of publication.

The amendment was **rejected.**

**K. Wilson’s Proposal 3**

**K. Wilson** asked the Section to consider Prop. 3 before voting on Prop. 2. This was a general Note, which some would say was stating the bleeding obvious, but it was sometimes important in the *Code* to emphasize its features.

**Buck** wished to speak to the proposal in a general way rather than a specific one. Despite the nay-saying of certain luddites, the reality was that electronic publication was here to stay. He felt the Section could not ignore this and have nothing in the *Code.* People would do this in hundreds of different ways if the *Code* made no Recommendations. Then six years on the Section might take decisions invalidating scores of names. The Section had to face up to the fact now that electronic publication was here to stay, and provide guidelines for those that wished to use it. But this could not be edited by a group of 200, and the Section needed to allow it to be edited by a smaller group.

**Stuessy** did not think the International Botanical Congress really was the body to do this. There were too many parts of the IBC and they really had no authority to judge this. He felt the Section needed to get away from this and say “until it is assured”. It didn’t say by whom, and who would assure was a good question, but he did not think the IBC was what was wanted. He made a **friendly amendment** to eliminate that.

**K. Wilson** found the suggestion acceptable. She preferred also not to specify, but some people who had spoken to her felt there should be some body to determine if acceptable archiving had been achieved.

**Hawksworth** presumed that meant that if a publisher made the assurance that a journal was being archived, that would then be ok.

**Zijlstra** had proposed the amendment because, for say in 2008, electronic specialists might try to convince botanists that permanent archiving of electronic publications had been assured. However, she felt the Section should wait until the next Congress before agreeing that was so. If not there was a danger that the proposed wording might open a back door to electronic publication being sufficient.

**McNeill** did not feel this was really a Note as worded. It was saying that the reason why the clause was inserted into Art. 29.1 was because of the issue of permanence. He was sure that had been a factor for some people, but it may not have been the only reason. It was not clear how the first clause hung onto to anything else in the *Code*, and a Note should explain something that was implicit in the *Code* but needed to be spelled out for clarity. He felt that what needed to be spelled out for purposes of clarity was that just because publication in any electronic medium without printed matter was not effective publication, nevertheless this did not stop people publishing in electronic journals so long as there was printed matter. The Recommendation then followed as to how this should really being done. There was no need to explain why electronic publication alone was not allowed.

**Atha** pointed out that right now there was only one way to effectively publish a new taxon, and that was through printed matter. There were many ways to disseminate that information, and he could get on the radio and announce his new species. He did not think the *Code* should regulate how he made his announcement on the radio. There may be 100 ways to distribute the information, but the *Code* should regulate only one of them, and one at a time.

**K. Wilson’s Proposal 3** was referred to the **Editorial Committee.**

**K. Wilson’s Proposal 2 (continued)**

**K. Wilson** drew attention to changes in Prop. 2 made during the coffee break. These made clear that it related only to people publishing in periodicals that had a print as well as an electronic version, and the number of requirements had been cut down, but still gave guidelines for the future. Otherwise there was a danger of proposals not being made until the next Congress as already pointed out by Buck. She added that Prop. 4 was separate and should be considered after Prop. 2.

**Lack** wished to point out, as one of the proposers, that this was recommending what the group felt was good practice and nothing more. It had no binding effect, but tried to show a way which seemed sensible to proceed, because there were hundreds of ways in which to distribute taxonomic novelties electronically.

**Phillipson** pointed out that some editorial tidying up would be required if this was passed because “should be” appeared in all the numbered points.

**K. Wilson’s Proposal 2** was referred to the **Editorial Committee.**

**K. Wilson’s Proposal 4**

**K. Wilson** introduced the proposal, which referred to all effective publications, that is all hard copies. It was seen as a way of trying to ensure that where electronic publication was used, there would be more than two hard copies printed. They saw ten as reasonable as that would cover copyright libraries, and geological or palaeontological libraries would also be relevant. The group did not feel it should be too limiting, but that copies should be spread around the world and should go to indexing centres such as Kew, Harvard, Canberra, and *Index Fungorum* – to any one of the relevant indexing centres.

**Veldkamp** was very happy to see this proposal, and was very much in favour of it as it would cover Dutch PhD theses of which there were 100 copies widely distributed.

**Gams** also endorsed the proposal, but it was a Recommendation and the libraries were spread, and the “should” would be better dropped.

**Nicolson** accepted that as an editorial suggestion.

**Funk** felt that if the Section really wanted to see copies in ten libraries, this should be made mandatory and not just a Recommendation.

**Nicolson** asked if ten was enough.

**McNeill** wondered, as this was a Recommendation, why the number was being restricted to ten as opposed to “widely” or “very many”. Ten would be a nice minimum, but why not “very widely”.

**Wieringa** wished to make it 50 as it was only a Recommendation, but his proposal was not seconded.

**Dorr** realized it was only a Recommendation but felt it would be unwise to make it more than that. It was difficult enough to meet all the requirements of the *Code*, and the last thing he wanted to do was to canvass libraries to find out if there were ten copies of a publication, to which parts of the world they went, and whether one in Europe and nine in North America was sufficient. He felt this was ridiculous and the Section should stay with the requirements of the *Code* as they existed, though they may be problematic in stating that “copies” must be available.

**Peng** requested clarification as to whether “printed copies” referred to an article *per se* or the journal.

**K. Wilson** explained that this was originally prepared as a corollary to allowing electronic plus hard copy journal publication, so “printed” was probably not necessary at this stage, but some may feel it necessary to emphasize this was not a copy on a CD, a server, or in some other electronic form.

**Lack** thought the Recommendation was very valuable as this was an age-old problem. He recalled *Flora Graeca* printed in 28 copies of which only three or four were in public libraries. That was the early 1800’s, and it was now 2005, so he thought ten was O.K. and made sense.

**McNeill** emphasized that the proposal as written had nothing to do with electronic publication. The Section would not be saying it wanted copies widely available, but that there should be at least ten. This seemed to be switching the number down, although he recognized that legally it was only two, but the impression given was for a wide distribution. He felt this was an unwise Recommendation and better changed to “widely distributed” or “in many libraries”, implying clearly at least ten if not many more.

**Gandhi** informed the Section that the number 10 or 50 did not matter. He had not even been able to index names published in some North American journals because they had not been received.

**P. Wilson** wished to remind the Section of the comment made earlier by Knapp, that she had been approached by concerns who wanted to set up a totally electronic journal. The wording here was aimed primarily at electronic journals to guarantee there were some hard copies. If it was changed to a large number of copies, they would be producing a paper journal again.

**McNeill** considered that in that case the Recommendation should be strictly linked to the previous one, applying only to journals that were widely distributed electronically anyway. He had absolutely no difficulty with that at all. His concern was that it was restrictive if it was a general embellishment on the number of copies.

**Norvell** was concerned if the number of copies was to be inflated beyond ten, as so many libraries were not accepting hard copy unless there was a journal run. Libraries were decreasing stacks and going to electronic copies. The Section had to face the fact that a lot of libraries were moving from hard copy deposition to digital copies, and consequently felt the Section should not go for a number above ten.

**McNeill** enquired whether the feeling was that this Recommendation be restricted to journals produced in electronic and hard copy. He suggested that ten was fine if a journal was also distributed electronically in thousands, but only ten copies of *Systematic Botany* as a medium of publication was weird.

**Orchard** thought the problem was wider than this and also applied to printed matter, and suggested a **friendly amendment** to say “ten and preferably more” and wondered if that would partly meet McNeill’s objection.

**K. Wilson** accepted that as a **friendly amendment.**

**Nicolson** drew attention to Art. 30 on ephemeral publications.

**K. Wilson** felt that what was proposed was much stronger than that, which for her was too weak, and applying to somewhat different questions. Two copies printed out by *Index Fungorum* and placed in two libraries was, however, close to being ephemeral. She accepted Orchard’s friendly amendment.

**McNeill** pointed out that if passed there would have to be some editorial adjustments in relation to Art. 38.1 which was partly overlapping.

**K. Wilson’s Proposal 4** was referred to the **Editorial Committee.** [Applause.]

**K. Wilson’s Proposal 5** was **withdrawn.**

[*Here the record reverts to the actual sequence of events*.]

### Recommendation 29A (new)

**Prop. A** (10: 141: 8: 0) was ruled as **rejected.**

### Article 30

**Prop. A** (27: 52: *77: 1).

**McNeill** noted that Art. 30 Prop. A was one of those where the Editorial Committee vote had a special meaning, but he added that it was not a special meaning that the mail voters thought was an especially clever one. He reported on the vote which was strongly in favour of the Editorial Committee option with 77, 52 against and 27 in favour of the original proposal.

**Brummitt** supposed that he had to say something since he made the proposal. He explained that what he proposed was almost verbatim a proposal that his colleague Alios Farjon made at St. Louis six years ago. From what he recalled, it had received 59% of the votes when it needed 60%, so it failed by just a few votes [but see below]. He added that the long-running debate over whether theses were effectively published or not had never been resolved. He thought it was possible to make clear decisions on the issue and wished to see something that depended on what was written in the thesis. He did not think it was right that a thesis should turn up in the library and you had to write to the author, asking how many copies were produced, which was what was happening. He felt that the evidence need to come from the thesis itself. He had repeated the proposal that the ISBN number should be critical, but the Rapporteurs had come up with an alternative suggestion, which was certainly a fallback position. He had just found out that the Rapporteurs were aware of three such proposals from friends in Greece where the names had been included in international indices and so on. He urged that the proposals should be accepted only if it was clear that the number of currently accepted names that was lost was very small. He highlighted that the proposal was to introduce it from the first of January 2006, so there could not be any possible threats to names published earlier than that. He favoured the ISBN route, but if people did not like that, then he would support the option that took out the ISBN although he thought this was less clear. He wondered if “An explicit statement of internal evidence” was clear? His feeling was that ISBN was absolutely unambiguous and he had looked back through the discussion in St. Louis for a good argument against it and could not find any.

**McNeill** offered a small correction. The proposal in St. Louis that was defeated was actually an amended version that excluded the ISBN [354: 349; 50.4 % in favour – Englera 20: 154. 2000.]. He echoed what Brummitt had said. He also felt that it was a long-standing problem that the proposal would not completely address, as far as the past was concerned. He suggested a general discussion of the issue, without getting into the details of the proposals and only then take them up. He felt that it was a really serious problem as most people, in most countries, with a number of important exceptions, mostly in north-western Europe, and possibly in eastern Europe, did not consider the thesis itself to be effectively published and they [the candidates] went on to publish a paper out of their thesis. He thought that unfortunately, with modern methods of technology and thesis production, this was not reflected in the *Code*. If one took the *Code* literally, as was suggested by Schafer, he thought that one had to reconsider all these theses as media of effective publication, which was not what most of the authors wanted and had not traditionally been the practice in most cases. He concluded that it was very important to address the issue one way or another. The Rapporteurs’ suggestion was only perhaps to facilitate passage. If the Section was happy to include the ISBN number as a criterion, he was fine with that, he just wanted to see some movement on the issue if possible.

**Turland** added that one of the problems, as McNeill had mentioned, was that there were a number of important exceptions. There were some northern European theses that were published in journals with an ISSN and he knew of several cases of theses from the Mediterranean region, one from France and at least two from Greece, where the PhD theses were published very formally and were obviously quite widely distributed, for example, copies in the library at the Missouri Botanical Garden in St. Louis in the United States. New combinations and the names of new taxa were quite formally presented in those publications and, looking at them subjectively, he would say that they were intended as publications, but they contained no explicit statement to that effect and had no ISBN. He thought that such publications could be rendered ineffective and the Section should bear that in mind.

**McNeill** clarified that Brummitt’s proposal was only dealing with the future and such works in the future would not be media of effective publication.

**Funk** was curious what would happen with the current practice in the United States of publishing sections of a thesis separately as different papers. If the whole thesis was put in several libraries and then several papers were later published in different journals, what would be the correct date, if the thesis were considered a publication?

**McNeill** concluded that that was exactly the problem.

**Atha** thought that the ISBN was like a domain name and they were available for purchase. He pointed out it was not a designation regulated by the botanical community or anything other than money.

**Nicolson** was not sure of the answer to that question, but had seen publications with ISBN numbers that he was sure they had made up. [Laughter.]

**P. Hoffmann** followed up what Funk said, by saying that it was not necessary to put an ISBN number in a thesis if you wanted the effective publication to be the subsequent papers. She did not think “some internal evidence” was any better than what was already in the *Code* and already being used. She suggested that the Section could maybe agree on something very specific that needed to be in the thesis, or some specific way that new taxa needed to be presented for them to be accepted as effectively published.

**McNeill** asked for clarification about who was using “some internal evidence” now?

**P. Hoffmann** meant the indexers at Kew who had to decide on whether names were validly published or not, they had to go to the thesis and make a decision or, as Brummitt said, go to the author. She did not think “internal evidence” was adequate.

**McNeill** wished to clarify the “internal evidence” suggestion. He felt that the Section was just picking up the debate from St. Louis. He reported that the sorts of internal evidence that were suggested would be e.g., the ISBN number, because whether it was made up or not it was an indication of a clear intent to publish, as well as inclusion in a serial. He gave the example that many of the Scandinavian theses were published in serials, *Universitatus Uppsaliensis*, for example, that was an indication of intent to publish. He added that at the moment there was no requirement to use internal evidence beyond “was it printed and in two libraries?”, which he felt were plainly inappropriate criteria.

**P. Hoffmann** agreed, but referred to Turland’s comment about theses that looked professionally published and all the indexers had to go on was the internal evidence.

**McNeill** clarified that the point Turland was making was that the proposals they had put forward would actually rule those out if there was no clear, explicit, internal evidence of intent to publish, not just that it merely looked as if it were published, there would have to be an explicit statement. He felt that was the price you would have to pay if this proposal was eventually moved to an earlier date to deal with theses currently in existence. He concluded that this would be a price the Section would have to decide if it were willing to pay.

**P. Hoffmann** suggested “a clear statement” rather than “clear internal evidence” as being a bit more specific.

**McNeill** thought it might, however, rule out published work in journals that were, in fact theses.

**Woodland** wanted to support what was before the Section. He felt that there was a difference between a thesis and a dissertation and there were definitely dissertations published by institutions in dissertation series. He did not think that these were valid publications because they were nothing more than the reprint of a publication and there was no access to them. On the other hand he gave the example of a dissertation in North America which was indexed and all sent off to a particular source. If you wanted to access one, you went to that source and they would send you a bound copy which he thought made it a valid thing. He had in his files two dissertations that were “published” as publications and they were nothing more than dissertations. He was sorry it was not passed in St. Louis and thought it should be passed at this Section.

**Glen** noted that in South Africa, ISBN numbers were quite common and it was possible to organize an ISBN number for a publication with a single phone call. He had “been there, done that” but unfortunately, the money ran out after three copies of the publication were photocopied so he did not consider this properly published. His ideal answer to the issue would be an explicit statement by the author that publication was considered by the author to be a publication or not to be a publication, whichever the case may be. He suggested it might read “This thesis is considered by the author to be effectively published in terms of Art. 30 of the *Code*” or “…not to be effectively published” as the case may be.

**Kolterman** suggested considering changing the date from 1 Jan 2006 to 1 Jan 2007, as in the proposals under Art. 32. He was not sure how widely available the *Code* would be by Jan 2006.

**McNeill** noted that that could be taken into account by the Editorial Committee.

**Gandhi** had recently come across a European publication at Harvard while maintaining a database of botanical publication titles. He wanted more information about the particular title so, using the ISBN number, had contacted the Library of Congress at Washington DC but they did not have any information, which came as quite a shock. He thought ISBN numbers were universal and that some information would be available. As Nicolson mentioned earlier, he concluded that the number was probably made up. He also wished to note that the distinction made in the USA between theses and dissertations was not universal. In India, the PhD submission was called a thesis and hardly anyone knew the term dissertation. Most Master’s degrees were awarded only by annual exam, not for a written submission. As well as this he highlighted that Indian theses were usually sent to a foreign country where English was spoken such as Britain, Australia, New Zealand, Bangladesh, etc. The external examiner had to approve the passage of the candidates and without such approval, the thesis was considered to have failed.

**Hollowell** contributed that for the journals *Novon* and the *Annals of the Missouri Botanical Garden*, an ISSN number, a serial number was assigned but not all their publications carried an ISBN number. She suggested they should uniformly assign a Library of Congress number, which were not consistently represented throughout their monographs and other publications. She thought it was necessary to decouple from the ISBN number because it was not a consistently applied criterion.

**Gams**, when acting as a supervisor, usually discouraged the publication of taxonomic novelties in a dissertation or thesis, for example, discouraging the student from supplying a Latin description. In most cases, his experience was that the student would intend to publish taxonomic novelties separately and felt this should be encouraged. He not only supported the current proposal, but also supported adding a new Recommendation that nomenclatural novelties should not be published in theses.

**Freire-Fierro** wondered how many of these theses were going to be made available as sometimes only a few copies were printed and these were available only in one country. She was thinking particularly of a thesis that included information of interest to her and that if she wanted a copy, it would be $30.00. In Latin America, if you wanted to have the original description, you would have to pay that price.

**Demoulin** did not think it was possible to start the debate begun in St. Louis again, so chose not to tell the story of his own thesis again. He suggested people could consult the Proceedings. He thought that the Rapporteurs’ proposal was a good way out. He felt that ISBN should not be a rule but it was an example of one kind of evidence. He felt a date in the future was fine, but the big problem was not in the future, the problem was in the past and it was important to take care of what had happened in the past 50 years. He gave an example of why this kind of ruling was urgently needed, not for the future but retroactively; theses made up of reprints. He added that there was no problem with a compilation of reprints of papers already published; publication had already taken place. But very often he had seen theses that also included proofs of papers not yet published, or manuscripts that had been submitted, or even not submitted. He argued that if a thesis like that was accepted as an effective publication, then you would have effective publication of something that would later appear [in a different form]. Just like it had been the tradition of many countries, in his country and he thought Brazil, a student made their thesis and submitted it to the jury and, based on what the jury said, they may revise their work and then publish a taxonomic paper. He concluded that the Section should preserve the wording, but without the future starting point.

**Nic Lughadha** wished to quickly return to an earlier point, as she thought the issue of whether an ISBN was made up or not was a red herring; it was a clear statement of intent to have something treated as a publication. She thought her colleague, Brummitt, was prepared to accept the Rapporteurs’ suggestion as a friendly amendment and suggested it would be ideal to have one of the Examples mentioning ISBN or ISSN.

**McNeill** asked if she meant she would like to see the Examples before she voted? He added that there was no question that this would only make sense if the Examples were included in the *Code*.

**Nic Lughadha** was willing to accept the principle with the assurances that the Examples would be in the *Code*.

**McNeill** thought it might help to split it up, summarizing that the initial proposal dealt with the future, while many of the comments dealt with the past. He suggested continuing to deal with the future first, if that was acceptable, then the Section could continue and deal with the past. He referred to Nic Lughadha, assuming she was accepting their amendment but retaining the date in it.

**Barkworth** was basically in favour, but wished to include something out of electronic publication: If you want a thesis accepted as a publication, you state that and you state where the copies were being deposited in libraries. She thought more than two libraries would be appropriate, but that would be internal.

**Chaloner** was surprised that no one had raised the issue that lurked in the background, which was the longevity of the publication. Fifty years ago, a published, printed thing was very clear; it was with carbon-based ink on paper. He was enormously alarmed by the talk we had a few minutes ago of three photocopies and the funds ran out. He argued that the idea that the blessing of an ISBN number or any other registration in some way made the publication secure years from now, one hundred years from now, was a complete illusion. He was worried that that matter had not entered into the discussion at all because he thought it was deluding ourselves that by some formal registration of “a publication”, which was in fact being reproduced photographically, with all the impermanence that that carried, was a serious consideration here.

**West** found the discussion quite disturbing because she really felt we should be doing everything possible to train new taxonomists/systematists and here we were saying that we could publish names in theses. She thought they should be encouraged to publish in journals, where things were properly refereed and properly accepted by peers.

**McNeill** felt that the point was that the current wording of the *Code* permitted it, even though no-one wanted it.

**Orchard** endorsed West’s comments and went one step further and asked if the words “or other internal evidence” were really necessary in the proposed motion. Given that this was only going to apply to theses, and there would be notice given in advance that there would be new regulations for theses, he wondered why not require that there be an explicit statement as part of the regulation instead of leaving it vague? He would require, in a thesis from 2007, a statement “I intend this to be a publication”.

**Turland** wished to add something in the interest of presenting both sides of the argument. He was looking at the Rapporteurs’ wording and placing himself in the hypothetical position of a person who might be publishing a thesis. He suggested that they could read the *Code* and think, “well, I don’t really not want my thesis to be effectively published, I’ll put an explicit statement in, because the *Code* says I should.” However, they may have only two copies produced, one for themselves and one for their supervisor or for their university. Someone had mentioned a Recommendation that it should be more widely distributed. There was already a Recommendation, Rec. 30A.1, that mentioned that it should not be unlikely to reach the general public. He thought that perhaps theses or dissertations should somehow be inserted in that Recommendation, so that it was more explicit.

**Stuessy** thought it may be possible to bring that point in. From his point of view, the Scandinavian series were really the most complicated. The problem was not so much the theses that sat in libraries, as it was the theses that were serials. He suggested striking out “non-serial” from the original proposal, and then picking up part of what was offered by the Rapporteurs. In other words, leaving after “work stated to be, etc” down to “as effectively published”. “Unless it was so affirmed by its author and also distributed to botanical institutions with libraries accessible to botanists generally.” He felt that picked up two points: the author must state that they intended to publish and second that it had to then be broadly distributed, using the wording that was already in the *Code*.

**McNeill** pointed out that that wording was already in the *Code*, so it was unnecessary to bring it in again.

**Stuessy** agreed that it was not needed. Still, the issue as he saw it was that you still had the possibility of people doing their theses that was not in any sort of serial form. They could then distribute this themselves to the botanical community. He argued that at least then they would have to make quite an effort to do that and they would have to state clearly in the thesis that they intended to effectively publish.

**McNeill** thought that that was obviously the route. From some of the theses from one particular university, that he and the Vice-Rapporteur had noted, they habitually treated the thesis without any other comment as something they distributed quite widely, he thought by gift. In the future, they would need to insert a statement in order to meet the requirement.

**Dorr** was having a little bit of trouble with the “explicit statement”. He spoke several languages fairly well but argued that there were a lot of languages in the world and somebody could make an explicit statement in a language that no-one at the Section meeting could read. He thought that when proposing new combinations or new species, the *Code* was very clear that one must use the specific statement, “sp. nov.” or “ comb. nov.”, and have a Latin diagnosis. He continued that there had been a move away from the inadvertent introduction of new names by making it somewhat formulaic, but when it was opened up to any language, any possibility, he felt everyone was back to the point of trying to figure out what somebody intended. He argued that if it was in a journal, then the intent was clear.

**K. Wilson** was brought up, at Sydney University and the University of South Wales, to believe that a thesis should have a statement saying that the thesis was not intended as a publication for nomenclatural purposes, to avoid any possibility of anyone taking such juvenile work, as it often was, as something that should be validly published. She thought that was still true and that most students wanted publications in refereed journals, which were more valuable to them than the dissertation as a publication. She responded to Dorr’s point, by suggesting that maybe, to be really restrictive that we put in the *Code* a statement, in Latin or possibly English, that must be put in a thesis if it was to be accepted as effective publication. She added that if it were to meet Dorr’s objective, it would have to be a precise wording. She suggested “This thesis is intended to be a publication for nomenclatural purposes.”

**McNeill** found it important to have some statement in the *Code* that allowed you to say that your publication was **not** effectively published. He clarified that the criteria for effective publication did not include a person saying their work was effectively published. He thought the president had once made the comment that you can say that you’re not walking on the road, but you can still be run down by a bus. His basic point was that it is not what you say you are doing that matters, but what you do. He considered that to be true for effective publication at the moment.

**Mabberley** wished to reinforce what West had said. He posited that one way to move toward that would be to beef up Rec. 30A, inserting in the strongest possible terms that such theses not be seen as vehicles for the publication of taxonomic novelties.

**Basu** believed the criterion of the ISBN number was a very good idea. It may be considered unwise, but why was it unwise? Why not accept other internal evidence too? He gave the example of the University of Calcutta, where one copy of the thesis had to be sent to a foreign university to establish validity.

**Briggs** pointed out that the suggested requirement that a thesis require a statement that the thesis was not a publication for nomenclatural purposes would be dangerous since the omission of the statement would imply that the thesis was, indeed, a publication for such purposes.

**Landrum** cautioned that one thing the Section may be forgetting was that “effective publication” was something we all understood but a student or maybe a not-so-experienced professor may not understand. He felt that Stuessy’s idea of explaining exactly what was meant by effective publication might be important to include.

**Nic Lughadha** suggested it would be possible to address the Dorr issue of recognizing the explicit statement by asking that people cite the Article, “This thesis was intended to be effectively published according to Art. 30,” or what ever Article it was. She argued that it should make the statement recognizable in any language.

**Malecot** offered a French point of view, that it was not a problem of the effective publication of the thesis but a problem of the valid publication of the names within the document. In his thesis he had made a statement, in French, that said that the names in the thesis were not validly published, even if the thesis was distributed and there was one copy in Missouri and one in Paris. He argued that it was clearly that it was the names that were in the thesis that were either validly published or not validly published rather than a problem of accessibility.

**McNeill** agreed that that was perfectly correct, it was quite possible for an author to write that he did not accept the names appearing in the work but he could not say the work was not effectively published under the present *Code*. He explained that this was because if the author said his names were not validly published, he was not accepting them, but if he said the work was not effectively published, he was just telling a lie, because it was.

He summarized that what was on the table was the original Brummitt proposal with the accepted friendly amendment to remove the ISBN number and insert the words that the Rapporteurs had suggested but still with the date of 2007. Having had the general discussion he thought that was the basis on which the Section should move to decision. He added that if it was passed, he or Demoulin would suggest an earlier date, but that was quite a separate matter. He pointed out that a lot of other things had been suggested and if anyone wished to enshrine them, and modify the proposal, they should move amendments.

**K. Wilson** asked if that meant he wanted to leave “non-serial” or cut that out?

**McNeill** felt that was important but deferred to the proposer, whether he wanted to accept our “publication” underneath and take it out or leave non-serial in.

**Brummitt** wished to leave it in.

**Woodland** recommended taking it out, for the simple reason that he had encountered institutions that took theses, gave them a serial number and published them straightaway which would then be considered a valid publication.

**McNeill** thought that it would have to be moved as an amendment (unless it was considered friendly). He wondered if he was thinking of University Publications [perhaps University Microfilms?] in Ann Arbor as he did not know that they issued theses with a serial number.

**Woodland** was thinking of his own institution, which had an archaic dissertation series that some people had been trying to get rid of. They called it a Dissertation Series, gave it a number, and this was sent out to various libraries and institutions. He emphasized that it was nothing more that an unmodified, or slightly modified, dissertation with a serial number and if this were a science thesis coming out, then it would be a valid publication. He felt that if the proposal were to read “independent work”, without the “non-serial”, it would get rid of the problem.

**McNeill** told him to talk to the proposer. If Brummitt wanted to keep “non-serial” in despite that comment, then it would require an amendment. He thought that if there was an Example that dealt with something like *Symbolae Botanicae Uppsaliensis*, then the word “non-serial” would not be needed, but he recognised the point. From Woodland’s comments he thought that the university intended the dissertations to be published.

**Woodland** agreed that they did, but there were a good number of people that did not feel that they were valid publications. He hoped that his comments would be accepted as a **friendly amendment**, because he supported the concept of the proposal.

**McNeill** clarified that it was **not** accepted as a **friendly amendment.**

**Wieringa** wished “non-serial” to be included, because it would validate series like *Symbolae Botanicae Uppsaliensis.* He thought that it might lead to the strange situation where two of a series were dissertations and names published there would not be validly published while elsewhere in the series, names were acceptable. He described this as a weird situation and suggested that the Section should try to avoid it.

**Redhead** preferred to see “non-serial” in there, because if it was lost, he began to wonder what the word “independent” meant.

**Alford** felt that it was complicating the issue. Since it was dealing with the future, he suggested why not declare that no thesis was effectively published?

**McNeill** replied that this was for the simple reason that in some countries they were intended to be effectively published.

**Alford** wondered why they could not publish them in some other form?

**Dorr** offered an amendment that “explicit statement” be cross-referenced to Art. 30? [This was **accepted** as a friendly **amendment.**]

**Eckenwalder** had one other quibbly thing to say about the ISBN and the serial titles; ISBN does not apply to serials so he felt that needed to be cleaned up.

**Orchard** suggested deleting “or other internal evidence”. [This was **accepted** as a friendly **amendment.**]

**Zijlstra** was against deleting “or other internal evidence” because that would rule out the Dutch dissertations that were published as independent books. If there was a clear, external publisher mentioned, she considered that as internal evidence that the book was effectively published.

**McNeill** thought that that actually was the original reason for putting it in. As the change was accepted as a friendly amendment, he noted that it would need to be voted on, unless the author accepted the change back as a friendly amendment?

**Brummitt** could see that “other internal evidence” was very subjective. His feeling was that it would be better left out but in his heart of hearts he would like to return to the original proposal because it was absolutely simple; if something had an ISBN number, it was in; if it had no ISBN number, it was out.

**McNeill** said that, in that case, he should want “other internal evidence” in, because that was the only way you could use an ISBN number, which was internal evidence. The Example would pick up the ISBN number and link it to other Examples of internal evidence.

**Brummitt** thought McNeill was right and it should be back in.

**McNeill** summarized that Zijlstra’s suggestion was **accepted** as a **friendly amendment.**

**Barrie** was going to argue the opposite of what Brummitt had originally said. He thought there would be problems deciding what was an explicit statement, so leaving “other internal evidence” in as a fudge factor would be very useful.

**Bhattacharyya** pointed out that not only ISBN but other systems were used in other countries and what classification system was used was a matter of library science. He reported that in India they used Ramaswamy, and other countries may also use other kinds of numbering. He felt that stipulation of ISBN was a monopoly affair and the system should be a matter for library science and the various countries themselves.

**Nee** felt that as the proposal was dealing only with theses, that narrowed the issue. He felt that as you had to say “sp. nov.”, and you had to state that a lectotypification was being made in a specific place, rather than relying simply on internal evidence, why not put in the thesis a word such as “validatur” – “let it be validated” or something else very specific. He argued that if that word was absent, it was not validly published. It was not the kind of word that would occur in any other situation, so nobody was going to use it otherwise.

**McNeill** asked if that was proposed as an amendment? He did not think it would be a friendly amendment, but acknowledged that he may be wrong.

**Nee** was just throwing it out as an idea.

**Stuessy** wished to offer an amendment along those lines, returning to what he had said before. He found it a little odd, but he thought that the point just made was that it was the question of whether or not the author considered the name validly published in the thesis that was the issue. He added that it may be distributed worldwide, but that was not the issue. Starting out with what was in the proposal, he did not think “non-serial” was a good thing, so chose to leave that alone. He suggested adding, “Is not to be treated as effectively published unless it includes a statement that the author regards all included names as validly published.” He concluded that it seemed a little odd to have to make a statement about it being validly published in order to have it effectively published, but asked if that was not really the issue?

**McNeill** felt that effective and valid were being mixed up and added that you could not make valid publication a requirement for effective publication. He reported that Brummitt was agreeing with him! [Laughter.]

**K. Wilson** wanted to check that the phrase “other internal evidence” was in the correct place.

**McNeill** responded that it was where it was to begin with and if it had somehow been misplaced while typing, then it would go back to where it should be. He assured her that the wording had not changed in that sense.

**Stuessy** felt that the author did not decide whether it was a publication or not, that was a physical process of printing, and a certain amount of dissemination. He said that had to be modified.

**McNeill** thought that this was creating a criterion for effective publication, which was not currently in the *Code*, but which said that a person had to think it was.

**Stuessy** felt that “regarded as a publication” was senseless as the author could not decide whether or not it was a publication, that was a physical act.

**McNeill** clarified that what the wording said was that the author had to make a statement that it was regarded as a publication under Art. 32, or that there was other internal evidence.

**Stuessy** reiterated that the author could not say that.

**McNeill** replied that what we were saying was that the author had to say that.

**Stuessy** was adamant that he could not do it; that it was a physical thing which the author did not control it.

**McNeill** responded that, first of all, it had to meet the requirements of effective publication; that was axiomatic and this was an extra hurdle that would be required for theses.

**Stuessy** argued that the wording did not work.

**McNeill** thought that the intent of the proposal was clear and if the wording was defective, then of course it would be edited.

**Demoulin** referred to Malecot’s comment that there was a way out through Art. 34.1. He felt that, even if it might be more logical to deal with these issues under valid publication, there were precedents for treating them under effective publication. He gave the example of Art 30.3, which says that “Publication after 1 Jan 1953 (he interrupted himself to say that that would be a good date for us!) in trade catalogues or non-scientific newspapers or in seed exchange lists, does not constitute effective publication.” He thought it may be stretching a bit to use Art. 30 to define what constituted publication, but it had been done before and no one protested about losing trade catalogues. He summarized that it was an easy way out to add theses to the list of publications considered non-effective, even if widely distributed.

**Buck** feared that he had been an editor too long, but was bothered by “a non-serial work” and then, in the last line, saying “a serial title” as evidence? He wondered how a non-serial work could have a serial title?

**McNeill** agreed that would have to go as it was a hangover from the previous wording.

**Buck** knew for a fact that it was possible to buy a block of ISBN numbers and use them as you chose including assigning one to a single copy of a book.

**McNeill** agreed, but felt there were two issues here that were involved. One was the business of distribution and the normal criteria for effective publication and he conceded that the *Code* was not terribly helpful at the moment in that it required only two copies to be distributed, but he emphasized that was not under discussion. He thought the Section recognized that what was there was not perfect but at least it was there and it worked. He asserted that what was being looked at today was an added hurdle for theses, specifically trying to address whether or not the author, or the publisher, intended for the thesis to be effectively published. He added that the current wording was somewhat problematic; but what changes were needed was purely editorial.

**Malecot** suggested that in order to separate the effective publication of the document from the valid publication of the name within the document, he was thinking of a statement that was similar to what occurred in the zoological *Code*. He proposed the following amendment: After “... it is not effectively published,” include the statement “…unless it includes an explicit statement by the author or publisher that it is regarded as a taxonomic work where ICBN rules apply.” He elaborated that within the work were new names and the authors were taking two steps: one, they regarded the names in the work as validly published and, two, that they applied the ICBN rules to the work. He noted that this was similar to the zoological *Code* where they do not say the work was effectively published; not that the names within the work were validly published; they simply say that the rules of the zoological *Code* were followed in the work.

**McNeill** considered that a formal amendment. [The **amendment** was **seconded** and written on the board.]

**Pereira** had advised on many theses from the University of Rio de Janario and was of the opinion that they would have many difficulties if the proposal were approved, he supported retaining Art. 30 as currently written.

**Barrie** did not consider his dissertation effectively published but he did consider it a taxonomic work where ICBN rules applied and he certainly tried to use them. He did not think the amendment was helpful because he felt it would bring back theses that may be excluded otherwise.

[The **amendment** was **rejected.**]

**McNeill** returned discussion to the original Brummitt proposal with the friendly amendment.

**Brummitt** knew it would go to the Editorial Committee, but did not like “is regarded as a publication”. He wondered what kind of publication?

**McNeill** felt it would have to be an effective publication.

**Brummitt** thought that “as such” might resolve the issue.

**McNeill** noted that the suggestion was recorded.

**Zijlstra** suggested a small addition: “ Unless it includes on the title page...” She argued that if you had a thesis in Chinese and saw “30” on the title page, you would understand.

[The **motion** was **seconded.**]

**McNeill** had a little worry about the suggestion as he could imagine formats in which the title page was so fixed that it was not permitted to add anything. He thought the intent to have it in the preliminary material was important. He was not sure whether “title page” or “preliminary material” was the most appropriate. [Aside discussion.] He reported back that the editor of TAXON said you can’t do that; it was “aesthetic matter”.

**Tronchet** suggested instead of title page it would be better to place it in the abstract because you cannot place whatever you want on the title page.

**Stuessy** pointed out that books do not always have abstracts. He listed preface, obverse of title page, end page as some options. But made a plea against using the title page as he felt that was a very special author’s time. [Laughter.]

[The **amendment** was **rejected.**]

**Nicolson** wondered if the Section was ready to vote on the main proposal? He was sure we could do some more editorial things… [Laughter.] Should we add parentheses?... [More laughter.]

**Wieringa** It would be useful to add an Example of a serial work such as *Symbolae Botanicae Uppsaliensis* so it was clear for everyone that series were effectively published.

**McNeill** felt that was an important point, which the Editorial Committee would bear in mind.

**Nic Lughadha** wished to clarify before the vote that the bottom line [on the screen] was not relevant to the vote. It was background information, which it was hoped would be added in Examples.

**McNeill** thought that as long as the wording was clear there was no need for a voted Example, the Editorial Committee could add ones that were appropriate.

**Prop. A** was **accepted** as amended. [Applause.]

**Demoulin’s Proposal**

**McNeill** quipped “So much for the future” and wondered if Demoulin wished to propose an amendment that this provision be applied from an earlier date? [He did.]

**Demoulin** thought this should be placed as a new Art. 30.4, the previous Art. 30.4 should become a new Art. 30.5, with the same date, 1 Jan 1953. He felt this should take care of the photocopy era. He did not think there would be anything before 1953, acknowledging that there may be a few theses which had been carbon typed, but the probability that they ended up in two or three libraries would be slight. He thought that dating theses with newspapers and seed catalogues would be nice for the homogeneity of the *Code*. He thought the suggestion would take care of all the problems and reminded the Section that the real problems were not in the future, they were in the past, especially in the era from 1965 to 1980 when photocopying became common and people were not yet fully aware of the consequences of it.

**McNeill** requested a clarification of the wording.

**Demoulin** read the full proposal, as amended, “Publication on or after 1 Jan 1953 in a thesis submitted to a university or other institution of education for the purposes of obtaining a degree does not constitute effective publication unless it includes a specific statement or other internal evidence that it was regarded as an effective publication by its author or publisher.”

**McNeill** summarized that he was essentially taking what was accepted and...

**Demoulin** finished the sentence with…replacing 2007 with 1953.

**McNeill** felt that was very clear and reiterated that the proposal was the same one but it was retroactive to before the date when multiple copies of theses began to be produced. He added that it was a classic issue that had been discussed at many Congresses and there had been attempts to deal with it by means of the Article that dealt with works that had to be acquired on request, although he was not sure where that was in the *Code* [He was thinking of Art. 29.2 of the *Sydney Code* (“Offer for sale of printed matter that does not exist does not constitute effective publication.”) that was deleted at the Berlin Congress]. He was referring to the Ann Arbor operation in the US that was the largest source of multiple copies of theses being made very effectively available in the sense of being widely distributed, but nevertheless in works not usually intended by their authors to be media of effective publication. As a final note he observed that this would obviously have a negative effect on the three or four publications that had been identified from Greece and France.

**Demoulin** agreed that of course a few things that had been adopted would be lost. But he argued that the benefit would be much bigger because it would close a big cupboard that had not been fully opened. He thought it was only a few cases where it had been opened, where a few Professor McGintys had discovered photocopied copies of a thesis somewhere and decided to change the date and place of publication of names that had been adopted from when they were published in a journal. He felt it was absolutely beneficial to go to the real place of publication. He acknowledged that three or four publications would be lost, but felt that it would get rid of a lot of future problems as well as problems that already existed.

**Lack** was afraid of losing many more names. He argued that there was a rich stock of theses, mainly from developing countries, which had been, in general, accepted and now they would be lost again. He warned against changing 2007 to 1953.

**Demoulin** was not convinced that such a large number of theses would be ruled out by it that had not already been taken into account and if they had been taken into account, what some indexers had done had been accepted by the general scientific public. He suggested that probably a large number of those were Scandinavian theses that would be exempted because they would include internal evidence that they were part of a serial.

**Mabberley** needed some education on what the *Code* was like on 2 Jan 1953, whether anybody preparing a thesis on that date would be able to refer to Art. 30 in the sense that was now meant.

**McNeill** agreed that Mabberley was perfectly correct and that was a very good editorial point that no Editorial Committee would allow in, it would have to be slightly modified to reflect what would make sense in terms of that time. He thought it would probably have to be a reference to the requirement, rather than the Article.

**Wiersema** questioned going back to this earlier date without better information about what the impact was going to be and therefore he would vote against it.

**Challis** explained that as an indexing centre they may or may not receive theses. So whether or not names were taken up in IPNI depended a lot on what was sent to them. She gave the example that in the last month they had not received a thesis, but rather, were informed that palm names from a Danish thesis had been taken up in the palm community. She reported that these were accepted about ten years ago and circulated in palm checklists and it would seem destabilizing if these names were not accepted.

**Gandhi** was also part of the indexing centre and they had been collecting typifications. In quite a number of American Master’s theses and dissertations, typifications had been mentioned in the past. What they had been recording were typifications from journals and books. He thought that if they had to go back to all those theses and dissertations, it would be a Herculean job to determine which typification had priority. He considered a starting point of 1953 to be more appropriate.

**Per Magnus Jorgensen** found the attempt very good, but was sceptical for one reason. He thought that backdating was always dangerous, if one was not fully aware of the consequences. For that reason he would have to vote no.

**Ignatov** opposed the starting point of 1953 because in many Scandinavian theses, they put in some papers that had been submitted but not yet published. He felt this would create confusion about the date of publication.

**E.M. Friis** was also against going back to 1953, she thought it would create problems for Scandinavian theses that included submitted but not published manuscripts.

**McNeill** requested clarification of the last comment. He wondered if she would consider a printed thesis from, her university, Stockholm, with an ISBN number, NOT effectively published?

**E.M. Friis** replied that the thesis was composed of a summary that had an ISBN number and then typically several published papers and then maybe an unpublished paper that was in press or would be published in the next year. She reported that in Stockholm the number was attached only to the summary, which was called the Kopf, the cape, but she did not know how it was elsewhere.

**McNeill** was not sure whether you could consider the whole work to be effectively published or not effectively published. He asked which she wanted it to be considered to be?

**E.M. Friis** wanted it to apply to the summary part, not the whole thesis.

**McNeill** wanted to know if it was distributed as a single work, because it was the work that was effectively published or not. He added that, without the proposal, it would be effectively published, even without the ISBN number and that the proposal would restrict theses that lacked internal evidence from being effectively published.

**E.M. Friis** felt it was very tricky because manuscripts were included in the thesis that would come out in the following year, for example, proofs.

**McNeill** asked if she would then support the proposal because it would restrict such theses from being effectively published.

**E.M. Friis** agreed.

**Demoulin** responded to Jorgensen by saying that he did not think it was in the interest of the botanical community to be obliged to go through gray literature to find out whether a thesis photocopied in 1975 had been deposited in two or three libraries. He pointed out that this would change the publication of the name from a widely distributed journal to an obscure thesis distributed in two or three copies. As these were not fully indexed, he highlighted that it was not possible to say how many names would be lost. He thought that the Rapporteurs comments were a good indication; they found three or four theses that seemed to have been generally admitted that would not be admitted any more. He urged the Section to contrast this with the large number of problems that were known to “still [be] under the carpet”. He referred to a paper in *Taxon* by Brazilian taxonomists that explained the problems for them when, like him, they published their new names in a regular publication after their thesis was submitted and later discovered that some McGintys wanted to push back the publication to the thesis.

**Turland** felt obliged to mention that the Rapporteurs did not carry out an exhaustive search and there could be many more examples that were not found.

**Gams** thought that if the Section accepted Demoulin’s proposal, there may remain a few debatable cases where the Permanent Committees may have to decide whether a particular thesis was to be recognized as validly published or not. He felt that this would be fairly easy to solve.

**McNeill** explained that one of the Rapporteurs responsibilities was to try to advise people on impact and Jorgensen had wisely advised them that where there was uncertainty they should be cautious. That being said, he felt this was a very unusual area in which in most parts of the world, he suggested most of South America, North America, Britain and substantial parts of Europe, the view of the student, the professor and the botanical community had been that theses that were not appearing in a journal as a formal, final dissertation for distribution, were not effectively published. He described them as media that would not be consulted for new taxa, new combinations and so forth, but he pointed out that as soon as they ceased to be typewritten, with carbon copies, they became, under the present wording of the *Code*, effectively published. He felt that the botanical community had conveniently and, he believed, wisely ignored it for the past 40 years. The difficulty that he saw if the proposal was rejected was that he would have to say to Prado and Picuda, the Brazilian authors of the paper mentioned, that he was sorry, whereas previously it was uncertain whether their thesis was a medium for effective publication, should the decision in Vienna be to reject the proposal, it suggested that it was [a medium for effective publication]. He felt that the Section had a dilemma, one that he could not totally advise them on, because it was unknown how many names would become destabilized, but he highlighted that there were enormous numbers of works that would become media of effective publication if the proposal was rejected. He was inclined to think that that was the more severe problem, because implicitly in rejecting the proposal the Section would be saying that the *Code* should be interpreted to mean that theses should be accepted as media of effective publication.

**Nicolson** moved to a vote and concluded that it passed.

**Nic Lughadha** disagreed with the summary, which she felt may have influenced the vote. She did not think that by rejecting the proposal the situation was materially changed but that the current, ambiguous situation remained. She did not interpret it that if the Section rejected the proposal the current ambiguous situation was changed by default.

**McNeill** did not feel that the current situation was ambiguous. He felt it was absolutely clear: If it was seen to be printed material and was in two or more libraries, the *Code* said it was effectively published. He felt that “We’ve just swept it under the rug, wisely so in my opinion”.

**Nic Lughadha** continued that it was often the case with a thesis that it was not easy to know if it was in two libraries or not. She was adamant that the current situation would not be changed by rejecting the proposal.

**McNeill** agreed that the current situation would not change.

**Brummitt** requested a card vote!

**Nicolson** asked for a show of cards even though he felt it never quite worked. He thought it passed. He asked if the Section would accept his ruling, or if there was a request for a formal card vote? [His ruling was accepted.] He thanked the Section.

**Demoulin’s Proposal** was **accepted.**

[*The following debate, pertaining to a New Proposal on Art. 30 presented by Wieringa regarding ISBN and theses took place during the Ninth Session on Saturday morning*.]

**Wieringa’s Proposal**

**McNeill** observed that this related to Art. 30 Prop. A already passed, but suggested the addition of a new Note.

**Wieringa** reminded the Section that the proposal that had been passed concerned theses. The Dutch became nervous about this new Article, though they liked it that some theses were now suppressed. However, he pointed out that the term “thesis” was used quite differently in The Netherlands to most parts of the world, where it was often the premature work of some student who submitted it and later published it. In his country, the final version submitted to a university had to be published to be valid for a PhD. This meant that 50 or even 500 copies were distributed around the world, and these had always been seen as valid publications. As they had been distributed all over the world, there was no sense in publishing them again afterwards. Many of these theses were in series which meant they were still valid under the present *Code*, but this was not the general practice, and there were many theses not part of a series which usually had either an ISBN number or the name of a publisher. He felt that adding this Note would make clear that theses with ISBN numbers or publishers indicated were effectively published. A few would remain which lacked these and their status would be disputable as to whether there was internal evidence or not of intent. This would save quite a few names, for instance in the recent thesis of Chatrou where he introduced several new genera and loads of new species in *Annonaceae*. He reported that the work was immediately picked up by *Index Kewensis* and had an ISBN number, but if the ISBN number was not considered by some enough internal evidence there would still be discussions these names are valid or not. He wanted to prevent uncertainty about such publications.

**McNeill** pointed out that the only difference between this proposal and what was already agreed on was saying this should be a Note rather than included in an Example. He clarified that the Section should address whether that would make it stronger and clearer.

**Brummitt** strongly supported the proposal, and thought it would be very useful, but he did not like the words “supposed to be” and wondered if he would accept their deletion.

**McNeill** noted that “presumed” or “intended” were possible alternatives but that could be treated as editorial.

[This was accepted s a **friendly amendment.**]

**Lack** supported it as it was exactly the same situation in Germany, where a person was only permitted to use the title of doctor after having published and distributed their thesis.

**Tronchet** also supported the proposal but was a little concerned as someone might put on an ISBN number when he really did not have one. Would it be treated as effectively published if the number was not real? Would the ISBN number have to be double-checked?

**McNeill** felt there was no protection against such terrorism.

**Orchard** wondered whether “regarded as” might be taken as the only kind of internal evidence that might be accepted. He wondered whether “regarded as examples of” or words to that effect would be better.

**McNeill** felt it would not as he read the proposal, as it was just making the ISBN citation stronger by having it as a Note and not just in the Examples.

**Nic Lughadha** did not feel there was any need to check the correctness of ISBN numbers as evidence of intent was being looked for. Even if a number had been falsified it would still be evidence of intention.

**Wieringa’s Proposal** was **accepted.**

[*Here the record reverts to the actual sequence of events*.]

## Fourth Session

Wednesday, 13 July 2005, 1400–18:00

### Article 32

**Prop. A** (131: 17: 10: 0).

**McNeill** introduced Art. 32 Prop. A by Brummitt which he reported had received a substantially positive vote in the mail ballot. He elaborated that the proposal was an attempt to rectify the fact that nowhere in the *Code* was it said that names had to be in Latin.

**Brummitt** found it very nice to be the author of a proposal that had received 131 votes in favour. He explained that the proposal arose when he was teaching a course and somebody raised the question: was there any rule against publishing names with names with full stops or numbers in them, or Chinese or Japanese characters? He realised that there was no stated rule that you could not do that and, although he had no evidence that anybody had ever tried it, it seemed to him that prevention was better than cure. He hoped that the proposal would go through.

**Rijckevorsel** wished to make a few observations. First he noted that the Latin alphabet referred to the 26 letters that all understood, however, he had looked up “Latin alphabet” and found out that there were three Latin alphabets that differed in the number of characters. His second point was that the alphabet was already in the *Code*, in the part on older citations, but it was called the Roman alphabet, so there was a conflict there.

**McNeill** thought that was an interesting point and if further research substantiated it, it could be dealt with editorially.

**Prop. A** was **accepted.**

**Prop. B** (27: 97: *22: 11) and **C** (31: 61: *55: 11).

**McNeill** introduced a series of proposals on Art. 32 regarding what was an acceptable description for the valid publication of a new taxon. He suggested that Prop. B and Prop. C, were, to some extent, alternatives where Prop. B took one position and Prop. C added a qualifying clause to it, excluding certain types of situations in which the description was identical between two taxa. He thought it would be beneficial for speed and clarity in the debate to take Prop. C first, because if it was accepted it in its entirety, Prop. B would just fall. He continued that if Prop. C was rejected, Prop. B, which essentially reflected what the *Code* already said with some modifications, could then be looked at. He explained that part of the reason was that this was another situation where the Rapporteurs suggested that an Editorial Committee vote would have a special meaning, that is, it would imply acceptance of the first part of the proposal. He noted that each of the proposals was in two parts, one talked about what would constitute an acceptable description in the past, and the other was an add-on, requiring that future descriptions be diagnostic. The Rapporteurs felt that these were separable things and it might be more beneficial to look at them separately. They had recommended that those who felt supportive of the definition of what constituted a description up until now should vote Editorial Committee. He summarized the overall picture by looking at the “yes” votes plus the Editorial Committee votes. For Prop. B there were 47 votes “yes” + Editorial Committee, versus 97 “no” votes, so he concluded it did not gather much support. Prop. C received 31 “yes”, 55 EC, for a total of 86, versus 61 “no”. He felt it was clear that the mail ballot preferred Prop. C to Prop. B, which was another reason for discussing it first and seeing what happened. He also suggested, for clarity, if the proposer did not object, that the Section first look at the first part of Prop. C, that was looking at the situation up until now, and, if that was agreeable, then consider whether to require that descriptions be diagnostic in the future.

He clarified that this meant in Prop. C, which will add a new paragraph and Examples (but they would be referred to the Editorial Committee), the part that was relevant to the past: “Any statement describing a feature or features of a taxon satisfies the requirements of Art. 32.1(c) for a description or diagnosis, except for any taxa for which the descriptive statement repeats the features as identical for another taxon by the same author in the same work. for which, etc, etc”. He hoped that would narrow down the initial discussion.

**Brummitt** apologized for grabbing the microphone yet again. First of all, he wanted to say that the whole business of nomina subnuda was almost, hopefully, the last area in the *Code* where chaos ruled. He very much hoped, now that the Section had disposed of theses, that it would also be possible to get a decision on nomina subnuda which he felt cropped up so often. He added that all of the proposals by Perry had arisen from discussion in the Committee for Spermatophyta. He had thought of asking for a Special Committee on nomina subnuda, but Perry had researched it and come up with Examples; he commended her as acting as a One Lady Special Committee. He felt that the main thing was trying to define what was the limited interpretive material. On one hand, one could argue that if someone in a horticultural journal said something about “this lovely shrub”, that was a validating description, because “lovely” and “shrub” were descriptions, but most people would not accept it as a scientific diagnosis. He thought it was very difficult to draw the line. He was against both Props B and C, because they would permit “this lovely shrub” to be a description validating a name. It said “any statement describing a feature or features describing a taxon satisfies the requirements of Art. 32.1(c).” He thought it would be a disastrous way to go as there was so much uncombed horticultural literature where all sorts of names could be dragged up, if that were accepted. He acknowledged that it was jumping ahead, but he felt that Prop. J was the important one. He explained that these cases came up in the Committee for Spermatophyta repeatedly, adding that in recent years, there had been a whole succession of them, and it was impossible to make a decision. On one hand, if they rejected a name that was a nomina subnuda, it implied that they accepted it as a validly published name, although most of the Committee believed that it was ridiculous to accept it as validly published. It was important to him, above all else, that the Committee was allowed to make a recommendation to the General Committee on individual cases, in the usual way, to say whether or not a name was validly published. He argued that without that authority, they could not make decisions on conservation proposals because they could not say whether or not a name was validly published. He concluded by saying that he felt both Props B & C would open up a huge can of worms.

**Perry** tended to agree with Brummitt that it would open a can of worms, she wished to point out that whether people liked it or not, the *Code* explicitly said, at least since Edinburgh, that a descriptive statement that described one feature and one feature only, validated a name.

**Zijlstra** agreed strongly with what Brummitt had said and wished to note an additional problem with Prop. C. She thought it would require not only consideration of the name in question, but involve having to look at the next pages to see if the same, short diagnostic statement was used elsewhere in the publication, under any generic or species name. She felt that that was impossible and looking at the name you were interested in should be sufficient. She added that this was especially a problem if you only had a photocopy of the single description, unless you knew that the generic name itself included a unique description.

**Moore** was pessimistic that a lot of the issue could be resolved because he felt it was easy to define “nude” but extremely difficult, as people who wrote decency standards knew, to define “subnude”. [Laughter.] He wondered if the way out of this was to give the Permanent Committees the ability to rule on this matter of valid publication and these subnude cases. He acknowledged that it might be arbitrary, but it was one way to get a ruling, just as with parahomonyms and other problems difficult to deal with.

**Schafer** thought the idea was very good, but was not at all convinced by Props B & C. He thought that they were not really clear enough and wanted the matter clarified before going to a vote.

**McNeill** thought that the problem Brummitt saw was that they were too clear and would make things validly published that he would not wish to see considered as such.

**Pedley** had a problem with the term “diagnosis”. Presumably, he suggested, one compared a taxon with its nearest relative, but this was not always the case. He thought it made it very easy to write a diagnosis if comparing to something remote from the taxon being described. He had a second problem that, in recent years, he had seen cases where three taxa were described and A was compared to B, B was compared to C, and C was compared to A so there was no point of reference.

**McNeill** made the point that “diagnosis” was not actually in the proposal being considered, that there was no suggestion that the diagnosis was required in the portion of the proposal being considering at the moment.

**Pedley** quoted “C: For a description or diagnosis...”

**McNeill** agreed but felt that the point was that that was exactly what the *Code* said throughout and the *Code* made it quite clear that a description need not be diagnostic.

**Bhattacharyya** felt that the wording of the proposal would simply increase the number of pages in the *Code* and increase its cost. He felt it was superfluous because authors followed the *Code* rigorously and distinguished between taxa in their descriptions.

**Watson** queried whether this would mean that if a book published, under separate species, two subspecies with identical diagnoses, they would be threatened. He gave the example “as for the typical subspecies but flowers white.”

**McNeill** assured him this was not the case because the wording said quite clearly, “..and for which there were no other distinguishing features indicated.” He pointed out that if two varieties were put in different subspecies, differences were clearly being indicated. He gave the corresponding example that there could be two “forma albas” under different subspecies.

**Gereau** noted that the *Code* required that description or diagnosis existed but it did not require that they be adequate, truly descriptive or truly diagnostic. He felt that for matters of the past, this was as it should be and for matters of the future, it was the job of editors, not the *Code*. He thought that editors should not be permitting inadequate descriptions or diagnoses; that was not for the *Code* to regulate. He suggested going back to basic principles; if it was clearly the intention of an author, in the past, with the barest attempt at a description or diagnosis, the name was there, it had been validated, use the type method, end of story and move on.

**Gandhi** agreed with Brummitt that “Lovely tree” or “large leaves” should not be sufficient for a diagnosis or description. In the example given by Zijlstra, it would not be easy to go through every page to see if the same characters were repeated elsewhere. He gave the examples of Don’s [actually Sweet’s] *Hortus Britannicus* and also Muhlenberg’s *Catalogue of North American Plants*, or Roxburgh’s *Hortus Bengalensis*, as being quite easy, as the same characters were repeated. He added that they may not be on the same page, but it was quite easy to declare them as nomina nuda, or nomina subnuda. He noted that almost three years ago, in a group discussion of the validity of the name of a composite genus from South America for ING, Zijlstra had declared that it was insufficient, even though about eight characters were used and no comparison was needed because the name was the only one in the article. Only after long discussion was the name accepted as validly published.

**McNeill** thought, if he had understood Gandhi’s argument correctly, that he was discussing the second part. He explained that the discussion had not reached that; that would be a requirement for the future according to the proposer. He thought it was only worth considering a clarification of what the *Code* currently seemed to say.

**Knapp** wanted to support what Brummitt and Zijlstra had said. She agreed that when you worked in a very large genus, it was very difficult to look on all those different pages. She had just completed a monograph of the tomatoes, which was an absolute nightmare for nomina subnuda because so many were proposed in seed lists and agricultural publications. She thought that if the Section were to adopt Props B or C, it would open up a huge can of worms, with all of those names that she currently had listed as nomina nuda. She agreed with Brummitt that the most important one of the proposals was J, which would permit the Permanent Committees to rule on validity.

**Perry** thought that many may have been thinking that a description had to include a diagnosis or that the description, in summation, had to be diagnostic, but she argued that that was the point of the proposal. She elaborated that the fact was that any descriptive statement, one that could not possibly be considered diagnostic, still fulfilled the requirements.

**Brummitt** responded that that was exactly the point he was trying to make. He picked up on what Gereau had said, to note that what mattered in these cases was the intention of the author. He acknowledged that of course it was often very difficult to pick out exactly what an author’s intention was when he wrote something 150 years ago, but very often it was possible. He did not have a problem with the second part of Prop. C, but, as Perry had said, it did expose the *Code* to any description as “Lovely plant” was a description.

**Demoulin** noted that he had yet to quote the great, absent Greuter, who had told him, and maybe the rest of the Editorial Committee as well, that he considered a statement such as, “Nice, pink shell from the tropics” from Sayle’s *Catalogue of Shells* enough of a description. He acknowledged that it was a zoological example, but felt that any gardener’s Catalogue was similar. His point was that for a former Rapporteur-General, any kind of statement was acceptable. He thought that if you were a monographer, you should have the complete list of the species in the genus you were working on. He felt that any proposal, like Prop. C, that limited the current situation could be useful.

**McNeill** wished to elaborate on what Demoulin said and pick up on what Brummitt said. He agreed that it was perfectly true that it was really the only interpretation you could make of the *Code* as it stood. He suggested that it was, quite legitimately, possible to question the word “lovely”, but the point was that any descriptive statement was sufficient to validate a name, according to the *Code*. He saw no alternative, except for those cases covered by Art. 30.2, Ex. 3 as there was no other provision for intent in the *Code*. That was why he thought it would be hard for a Committee to apply Prop. J because a Committee could not make a decision that was contrary to the *Code*. It was also why he found it difficult to make it work, without making the *Code* a little clearer. He reiterated that it was clear that there was no mention in the *Code* of intent except in the special case of names in tabular form. He was not saying it should not appear in the *Code*, just that it presently did not.

**Wieringa** had one comment on Prop. C, which he thought might be a problem. He thought that in a large work, where several genera were covered, it was quite possible that the author might describe a new species of *Papaver* by saying it was the only species “with yellow flowers” and elsewhere describing a species of *Sambucus* using the exactly the same statement and it would be invalid...

**McNeill** interrupted to point out that that had already been addressed. He explained that if they were in different taxonomic groups, there were other indications that there were differences.

**Wieringa** continued that that was only if genera were described, or if a key was presented and if there were no descriptions of families or genera or no key, by this wording, they would both be invalid.

That was not how **McNeill** read the wording. He felt that the “indication” was by placing them in a different taxonomic group, because that was implying all the characters that distinguished those groups elsewhere.

**Wieringa** persisted that it did not say “indication”, it said, “features indicated” and in his example, the features were not indicated.

**McNeill** felt that was clearly an editorial matter to be addressed. He maintained that certainly the intent was when they were in different taxonomic groups, it was a clear indication that it was not the same description.

**Nicolson** asked if the Section was ready to vote on Prop. C, adding that if C passed, then debate would return Prop. B.

**McNeill** clarified that the vote would be on the first part of Prop. C, not the part requiring a diagnosis for the future.

**Nic Lughadha** reminded the team that not all present were English speakers, so it was particularly important that the bit that was being voted on was highlighted on the screen and separated from the text on either side. [This was done.]

**McNeill** explained that the “except as provided” applied to proposals yet to be discussed and may or may not pass, if it did they would be inserted. The “Prior to...” dropped out for the moment, until the vote returned to the second part. So the vote was on “Any statement describing a feature or features of a taxon satisfies the requirement, etc., for a description or diagnosis, except for any taxa for which the descriptive statement reports the features that are identical to those given by the same author for another taxon appearing simultaneously in the same work, and for which there are no other distinguishing features indicated.” He added that that was to cover the situation where they were in different taxa. The second part was the “On or after 1 Jan 2007...” which he felt was a separate concept that should be dealt with separately.

**Brummitt** did not think it was necessary to look at all the nomina subnuda together, rather than picking out one or two here or there. He reiterated that Props B and C, despite their intention to restrict in certain circumstances, would open up accepting descriptions which were very sketchy. In his opinion, that would be disastrous, but, as the Rapporteur had said, some kind of guidance was needed. He asked that the Section look at Props D, E, F and G, where there was guidance, which would not open things up to very minimal descriptions, such as “this yellow shrub”, which were never intended as descriptions.

**McNeill** thought that what Brummitt was suggesting, and he recommended to the President do it before a vote, was spending about five or ten minutes on the topic in general. He clarified that this would not be dealing with any proposal in particular but allowing people to make points arising from them, as Brummitt and several others had already done from Prop J. The Rapporteurs were of the opinion that some of the proposals were quite independent of the others and would be useful additions to the *Code* such as the ones making clear that a statement that mentioned features of a plant, but did not indicate the expression of those characters, and those that talked about properties.

**Dorr** wished, before moving on to the general discussion, to ask that the Chair not unilaterally sever a proposal and force the Section to vote on a portion of it, unless it was done from the floor, with a seconder. He argued that it became very hard for the Section to follow what they were being asked to consider when the proposal was being unilaterally chopped up and divided again. He highlighted that the only things the Section could vote for and understand were either those proposals which were presented as they existed or those that were formally amended from the floor.

**McNeill** took responsibility for that and gave two reasons for doing it. First of all, it was specifically outlined by the Rapporteurs in the Synopsis, so the split was a split the Rapporteurs had suggested, and they said that those who favoured the split should vote Editorial Committee. For Prop. C he reported that the Editorial Committee vote was much higher than the “yes” vote, which suggested that the split had support. That being the case, he had suggested to the President that the discussion be approached that way, with the idea that, for those who wanted a diagnosis in the future, the Section would look at the second part of it.

**Dorr** repeated that his point was really that the proposals were printed and the Section had read them. He argued that the commentary by the Rapporteurs was different as they had not amended the proposals, just said, “Please consider this separately.” He maintained that if the Section was going to consider it separately, then that had to come from the floor; it could not be done in the midst of everything else such that, when it came to a vote, no one was clear what was being voted on.

**McNeill** thought it was pretty clear in the text, but if it was not, he felt that he had made it clear now. He allowed that the Section could certainly say, “Look, we don’t want to vote on only part of it.” If people wanted to take it as a single piece because they were unhappy with the lack of a diagnosis in the future, then he suggested they say so then the whole thing would be taken together. He contended that it was not true that it was not proposed and seconded. The Rapporteurs proposed it in print and it was before the Section before they cast their mail votes and he felt it was obvious that people had taken account of it, judging by the Editorial Committee vote.

It seemed to **P. Wilson** that the discussion was diverging a little bit from the intent of the proposal, which was to deal with identical descriptions. He felt that the Steudel example typified something that needed to be addressed. If necessary by amendment, he wondered if the Section could sever from this proposal the section that Brummitt found objectionable? He suggested removing the general statement, and sticking with the Examples the Section wanted to include.

**McNeill** pointed out that it was still a general discussion.

**Demoulin** thought that this was his sixth Congress, and he reported that Rapporteurs had always split proposals when it made things clearer and here he thought it definitely made things clearer, especially with the new electronic media. He thought such a proposal from the Rapporteur was much clearer than anything coming from the floor, including from himself.

**McNeill** asked if anyone wanted to address the other proposals mentioned by Brummitt?

**Rijckevorsel** supported Brummitt’s position on Prop. J, and he also liked the idea of Prop. E, and suggested that it might be added as a Note to Art. 32.2 independent of Props C and D. He thought that might be an elegant way to do it.

**McNeill** agreed that if it were to be passed, that was something the Editorial Committee would definitely look at.

**Nicolson** asked how the Section wished to proceed?

**Gereau** moved that debate be closed on the whole topic, a vote be immediately taken on the whole of Prop. C and then votes on the other proposals in order, starting with Prop. B.

**McNeill** replied that he knew that there was an objection from Dorr, but all of the discussion was on the proposed and seconded amendment that would restrict Prop. C to the portion dealing with names up to that point and not in the future. He felt that the proposal would be much clearer if it were dealing with Prop. C excluding the later date, because that was moved and seconded, effectively as an amendment, by the Rapporteurs. He checked if that was agreeable to Gereau. [It was.]

**Nicolson** clarified that the vote was on Prop. C without the date.

**Bhattacharyya** felt that mere addition of the word “diagnosis” did not seem useful for the valid publication of a name. He argued that there was the type specimen, a description and the taxonomic position. He wondered why an amateur’s diagnostic word should be accepted as the basis for validation of a name? It made no sense to him.

**Prop. C** was **rejected** both with and without the Rapporteurs amendment removing the date. [*Out of order and left so for ease of understanding.*]

**McNeill** turned to Prop. B, explaining that the difference between Prop. C and Prop. B was that the latter did not contain the component relating to situations where an author did not make his description unique; there may be two or more taxa with the same descriptive material. The Rapporteurs were of the opinion that this expressed the *Code* as it currently stood. They indicated that, whether we liked it or not, it was what the *Code* said already, though it did make it more explicit. They had made the point that in making it so explicit, it could be that names that had been conveniently swept under the rug would rear their ugly heads. They felt that other steps were quite important and there were some other steps, as had been noted. Whether they were enough to commend the proposal to the Section was for the Section to decide.

**Demoulin** felt that Prop. C had been rejected because it seemed that people believed that it would introduce something new, although the present situation was as the Rapporteurs described it. This was made clear in B, so he assumed that the Section must be logical and reject it. He also pointed out to Perry that the Example was not a good one, because *Agaricus cossus* was validated not by the few lines of description but by the plate. He added that this was a very common situation in agaric books of the late 18th Century that they were valid under Art. 44.2, so there was no need to talk about the description.

**McNeill** suggested that the Rapporteurs proposal should logically be taken up, even though, based on the failure of the previous vote which had more support in the mail ballot, he realized that the chances for its success were not high. He, and he thought many others, were opposed to requiring a diagnosis in the future, so he would have to vote against the proposal, but as he believed that the core part said what the *Code* already said so he could support it. He recommended that Prop. B be split the same way Prop. C was split, and the Section vote first on a clarification of what the *Code* currently stated.

**Nicolson** asked for clarification on whether that was without the dates?

**McNeill** confirmed that it was without the dates and with no requirement for diagnosis in the future, although the Section would address that immediately thereafter.

**Zijlstra** thought that Prop. B conflicted with a voted Example, Ex. 3.

**McNeill** noted that a voted Example did not reflect an Article of the *Code* and may even be in conflict with an Article in the *Code*. So voted Ex. 3 would stay as a special case and, he added, for those cases, would override the application of Prop. B.

Since Prop C had failed, **Perry** asked for a poll of the room to see how many believed that a name required a diagnosis to be validly published, as opposed to a description that was clearly not diagnostic.

**Nicolson** asked for a show of hands of how many people would consider a diagnosis as being required as opposed to a description.

**Perry** corrected him, as opposed to a description that was not in any way diagnostic such as “lovely shrub.”

**McNeill** thought “a red flowered herb” was a little better.

**Brummitt** felt that the lovely shrub was the heart of the problem. He argued that there could be a page-long description that contained no diagnostic information, but it was hardly comparable with nomina subnuda. He did not see the point.

**Nicolson** reiterated that Perry had asked for a show of hands and wondered if the Rapporteur-General wanted to speak to this?

**McNeill** highlighted that this was why there was the earlier general discussion, which people dried up on, which surprised him. He felt that it was a situation that all recognized was problematic and not easily resolved. He thought it would be beneficial, though from the mood of the meeting it appeared it would not happen, to clarify to some extent what the *Code* said to establish certain clear situations in which a string of words that looked descriptive were not a validating description, and these were some of the later proposals. The Rapporteurs did think that when it came to publications in special categories, particularly horticultural works, reports on shows, the gray literature, in that area there was probably need for further study because that was where a lot of the problems arose. He also mentioned it occurred to some extent in travel literature. He felt it was perfectly true that there was not an intent to describe a new taxon, although there was an intent to explain why the plant won the prize in the first place; there was an intent to describe, but not an intent to describe a new taxon. They thought that a Special Committee in the area might be very helpful. But before doing that, they thought it might be possible to at least draw to peoples attention what the *Code* seemed actually to say. On the other hand, he suggested that the Section may wish to leave it less clear and clean up a few points later on in the proposals, and either set up a Committee or not.

He thought everyone should vote according to whether they felt, like Brummitt, that clarifying the situation was dangerous, or whether they felt that it would be a sensible first step forward towards grasping this nettle.

**Nicolson** moved to a vote on Prop. B.

**McNeill** clarified that this was on Prop. B as amended by the Rapporteurs, covering the *Code* as it stood, without the requirement for a future diagnosis. [The proposal was **rejected.**]

In case there were those who preferred have the future diagnosis, **McNeill** suggested that the Section should again take another vote on the proposal as originally written, without the amendment proposed by the Rapporteurs. He pointed out that the only difference between this proposal and the one just rejected was that it would not only clarify the current situation but also require a diagnosis in the future. He suggested that if some people wanted the diagnosis as a sop to make them vote, they could do so now. He did not think it would make any difference, but that was for the individual voters to decide.

**Brummitt** pointed out that there were two dates and wished to know which McNeill was considering?

**McNeill** replied that they were the same date, one marked when the current situation ended and the other when the requirement for the diagnosis would begin. He added that they were the dates in the proposal as originally written.

**Basu** proposed an amendment “On or after 1 Jan 2007, such a statement must include a description and a diagnosis...” He suggested that putting “a description and also a diagnosis”, the diagnosis would allow identification of the taxon described correctly. He also thought that the rank should be included. [The **amendment** was not seconded so was not discussed.]

**Nicolson** returned to a vote on the full original Prop. B.

**McNeill** explained that it did not commit the Section to the Example as it had been pointed out that there was a problem with it.

**Prop. B** was **rejected** both with and without the Rapporteurs amendment removing the date. [*Out of order and left so for ease of understanding.*]

**Prop. D** (99: 32: 13: 13).

**McNeill** moved on within the same package of proposals but dealing with separate matters. He wondered if he was right in thinking that the Examples in Prop. D were not relevant because of the failure of Props B and C?

**Perry** [the proposer] felt that Prop. D was quite independent of B or C. She explained that it simply stated that if you indicated by which features two taxa differed without describing how those features differed, it was not validly publishing the name.

**McNeill** thought it was a rather interesting Example of someone who gave a Latin description of the things that were characteristic without saying what expression they took.

**Nicolson** summarized that they differed, but there was no mention of the difference.

**McNeill** suggested it would perhaps be referred to the Editorial Committee?

**Demoulin** thought it was an interesting point, but felt that it belonged with Art. 32.2, not 32.1 and that Art 32.2 would need improvement. He did not know if this could be done editorially. He elaborated that Art. 32.2 was the definition of a diagnosis, which was a statement of that which, in the opinion of its author, distinguished a taxon from others. He was not quite sure that this wording could be understood the way that Dvorak and Dadakova understood it.

**Barrie** remarked that the proposal was one of the reasons why the Section in St. Louis thought there should be a Special Committee to examine the whole issue. He felt that it seemed to conflict with the current concept of a diagnosis as defined in the *Code*. It was one of the concepts he thought should be looked over, along with the whole issue of nomina subnuda. He added that there was nothing in Art. 32.2 that said you had to state what the differences were that separated two taxa, all you had to do was state what characters were felt to separate the taxa, but it was not necessary to describe how those characters were expressed. He concluded that that was the current definition of diagnosis.

**McNeill** thought that would be an interpretation of what “that which” means. He understood “that which” to mean the expression of the features, not the features themselves. He concluded that the comment reinforced, in his mind, the need to have the Example in the *Code*, making clear that “that which” referred to the actual expression of the features which distinguished it. He thought it sounded as though there was an editorial question there. He assumed that the Section believed that a diagnosis should be diagnostic; it should not simply list the features that people saw were different, but how they in fact differed. He was sure that that was the intent of Art. 32.2 and if the intent was unclear, then it was editorial to fix the problem.

What Barrie had said reinforced **Demoulin’s** opinion that clarification of Art 32.2 was needed. For him, the problem was whether it was possible to do it editorially, or should the Section have something right now? He suggested something like “is a statement of how, in the opinion of its author, the taxon can be distinguished from others.”

**McNeill** thought that where the Section could help the Editorial Committee enormously, were the Example to be approved, would be giving clear authority to the Editorial Committee to make any necessary adjustment to the wording of Art. 32.2 to make clear that a diagnostic statement must be diagnostic. If Prop. D was approved, he promised that the Editorial Committee would make sure that it did not need to be a voted Example, that Art. 32.2 would be reworded to say what everyone believed it was meant to say.

**Davidse** noted that that would make the narrow interpretation retroactive, and was worried that a considerable number of names may be lost, possibly names in common usage.

**McNeill** indicated that he would be very surprised because the only situation he could conceive of was someone putting an inadequate diagnosis in Latin, and that was all there was, but people normally provided descriptions. Virtually all diagnosis that he was aware of, though they may not be truly diagnostic, did say what the feature was and its expression.

**P. Hoffmann** thought that the name Davidse just described was not a valid name because it lacked a proper diagnosis. She would interpret the Article to require not only a statement of the character but how it differed, so she would not accept such a name.

**Brummitt** thought that if there was any doubt at all, it was best to have the Example in. If the Editorial Committee could find a good Example, he advocated having it to avoid any conflicts in the future.

**Nicolson** moved to a vote on the proposal, noting that it might be at 32.2 rather than 32.1, but that decision might be editorial. He felt that the question was, whether the Section considered it a good Example to have in the *Code*. He added that the Editorial Committee would probably touch up Art. 32.2 so that it did not disagree with the Example.

**Prop. D** was **accepted.**

**Prop. E** (100: 20: 24: 12).

**McNeill** moved on to Prop. E, which was also independent of the other proposals. He introduced it as a proposal that would make clear that talking about the properties, economic, medicinal or culinary, were not descriptive terms for the purposes of a diagnosis. He thought it was quite an important proposal because, although it did not deal with the whole problem by any stretch of the imagination, it did tackle a number of names where there might be some doubt about whether it was a description.

**Redhead** did not like the proposal because there may be cases for the fungi, particularly the macrofungi, when looking at physiological features to distinguish things, and medicinal or culinary uses could be interpreted as being insufficient, when actually these were the characters that distinguished some of the macrofungi.

**McNeill** requested a clarification from Redhead. He asked if he was saying that if somebody said that his new species was distinguished from its congeners by being poisonous, he would think that was an adequate description, without identifying the compounds involved?

**Redhead** clarified that this was in older descriptions, nothing recent of course. He was a little hesitant, he could think of an example at the moment but hesitated to give carte blanche here.

**Veldkamp** wished to know what the difference was between a feature and a property. Because the discussion had been about features in the previous proposals and he was still wondering exactly what was meant by it. He wondered if it was anatomical, morphological, palynogical, molecular, edible? He felt that if that was all covered by feature, the features given for *Musa basho* were excellent. Moreover, he argued that if you were aware of the characteristics of bananas in the Far East, this was only one species. He added that if it was not clear, the type specimen was in the Herbarium in Leiden. He thought this was also part of the type method that if the description was not very clear, the type identified it.

**Kolterman** thought that that indicated what would need to be done to Prop. E if it was accepted, because the word “feature” evidently referred specifically to Prop. B or Prop. C, neither of which would be in the *Code*.

**McNeill** agreed that that was exactly the type of thing that an Editorial Committee was forever facing, that a proposal was drafted based on assumptions that ultimately turned out to be fallacious. However, he thought that the core was probably still relevant.

**Gandhi** reported that when the Example was discussed in their group, the mycologist told him that sometimes fungal taxa were differentiated solely based on their geographical origin, not on their morphology or any such thing. So he was not in favour of this particular Example.

**Demoulin** did not agree with the implication for fungi. He did not see why fungi should be treated differently from edible higher plants. He stated that there were edible and poisonous higher plants and there were edible and poisonous fungi. He felt that it might be true in some old descriptions that the feature might have been the prominent one, but that was not a reason to argue that it should have been part of the description, because it might have been wrong. If you go to some of some of the old descriptions of Amanitas, people considered in the 18th Century that *Amanita citrina* was a dangerous fungus because they confused it with *Acacia phalloides*. It was just one of the properties that they were attributing to that fungus. He argued that we should not include in a scientific description something that was one property. And on the issue of feature versus property, he thought it was for native English speakers to tell us what to do. He thought he understood the difference and thought that the properties were special features that related to use by man. He thought it was a very good proposal that would eliminate some difficult nomina subnuda and also avoid the need to look at the type of something when unsure what it was.

**Brummitt** suggested that if the word “features” was the problem, he thought the Section should just give the Editorial Committee the authority to change it to “descriptor” or something like that.

**McNeill** agreed that they would have to do that because of the proposals that had just been rejected, but the thrust of the meaning was quite clear. He added that it had to fit into what was acceptable under Art. 32.1 as currently worded.

**Landrum** was worried about the proposal in totality, not just the “features” and “properties”. He was thinking about some descriptions of *Molina* from Chile where the common name and the cultural use pinned down the plant. He could not remember the descriptions exactly, but he thought that may be all, other than that it was a tree. He thought there was a fine line between what was a cultural use and what was something other than that. He argued that the difference between cultural and botanical features was not always clear and gave the examples of hardwood or sweet fruits. He wondered if these were cultural or economic terms, or were they botanical? He opposed the proposal because he did not think it was a good idea.

**Printzen** wondered if the problems that Brummitt had pointed out could be remedied by adding “aesthetic” features to this list? [That was **accepted** as a **friendly amendment.**]

**McNeill** noted that where it was placed was editorial.

**Atha** did not like the word aesthetic. He felt that describing something as pretty was one thing, but he worked with older descriptions of fungi, where odours were described as pleasant or unpleasant. He argued that this may be considered to be an aesthetic judgement, but the terms were used very precisely to distinguish things. If that could be disqualified, then he could not agree to inserting “aesthetic”. He noticed that Demoulin was shaking his head, so thought that maybe he disagreed.

**Demoulin** felt that when it came to scent it was less subjective than the visual aesthetic.

**McNeill** acknowledged that it was terrible to keep amending things during the discussion, but suggested that “purely aesthetic” or “solely aesthetic” were probably the words needed. He felt that if there was an aesthetic element that was also descriptive, that should not be ruled out. He gave the example of “a striking, tall tree” where “tall” was a character.

**Marhold** was pretty happy with the proposal and if the only feature of the description was the origin or the fact that the name was sweet, he gathered that the name was invalid anyway.

**Gandhi** wanted to add that his colleague who worked on the flora of Japan agreed that the Example was acceptable as a nomen nudum.

**Proschold** wondered if it was possible to use molecular data, DNA sequences for example, as a feature for the description of a taxon? He gave the example that in some algae, they had the same morphological characters and could be differentiated only by their gene sequences. He felt that certain signatures were very characteristic for species and general.

**McNeill** replied that as long as the differences could be presented in print, of course that was perfectly acceptable. He pointed out before the vote that the voting on the preliminary mail ballot was 100 “yes”, 20 “no”, 24 Editorial Committee and 12 Special Committee, concluding that it was heavily supported in the mail ballot.

**Prop. E** was **accepted** as amended.

[*The following debate, pertaining to a New Proposal on Art. 32 by Chaloner regarding adding a term to the accepted Art. 32 Prop. E took place during the Ninth Session on Saturday morning*.]

**Chaloner’s Proposal**

**McNeill** explained that this new proposal related to one made by Perry that the Section had already approved regarding terms not regarded as qualifying as a description. Chaloner wished to add one to the list.

**Chaloner** said that the argument was that for a palaeobiologist, the time dimension was really the equivalent of the spatial dimension for biogeographers. Although of course it was of great interest in each case, that the distribution was thus and thus, it should not be treated as an attribute to be included in a diagnosis in that rather technical sense of a feature. The proposal had the support of the Secretary of the Committee for Fossil Plants. [The proposal was to add “geological age”.]

**Chaloner’s Proposal** was **accepted.**

[*Here the record reverts to the actual sequence of events*.]

**Prop. F** (26: 58: 5: 68).

**McNeill** moved to the next proposal, Art. 33 Prop. F which was somewhat different because it was looking to address descriptive statements in certain kinds of work.

**Perry** noted that many of the names that caused the most problems had been published in letters, travel documents, journals and the like. There were many names in such works that were very well described, and she was not arguing that these should not be accepted. Rather, it was the sort of name that occurred when somebody walked down a hill and said “I picked up a new shrub with white flowers and I’m going to name it after my friend Cunningham.” and goes on to call it *Podocarpus cunninghamii*, for instance. She felt they were the sorts of names that caused a lot of trouble. She argued that it was fairly obvious that the person was just giving field notes and had no intent at the time to validly publish a name, often he did not know that his work was going to be published as somebody else picked it up and edited it, and it made its way into the literature. In most cases, these names were validly published later, with descriptions, documented type material and she posited that the application of the name was very easy to decide. In many cases when there was a very short description in letters and the like, it was not possible to decide what they were, and there was rarely type material, so they caused a lot of trouble. She concluded that the proposal was an attempt to find some way of getting rid of those sorts of names.

**Dorr** asked Perry to clarify in the Examples which of the names were currently being accepted by monographers as basionyms of names being used in Australia? Because if he read the Examples correctly, he thought that at least the one on *Capparis gibbosa*, the most recent monographer of the genus *Adansonia* accepted it. He suggested that that was an attempt to fix the name.

**Perry** replied that it had come up before the Committee and that was one of the reasons that the problem had been looked at. She added that it came up, obviously, because the Australians were not very happy [with the acceptance].

**K. Wilson** responded that it was not just that the Australians were not very happy, and thought it needed a little more explanation. She outlined that there was a very well accepted name for the Australian boab and to have the name changed seemed rather pointless when it was coming only from one of those publications that were not intended to be systematic publications. She wondered whether the original statement, “...unless it was clear that it is the intent of the author to describe or diagnose a new taxon.” was clear enough. She noted that the point that was made earlier was that it was not the author’s intention to have it published, and wondered if adding something about intent to publish would make that section clearer.

**Dorr’s** point was not to argue about the past, but the fact was that when the genus *Adansonia* was recently monographed and a presumably stable nomenclature was presented, the monographer accepted the name as the basionym for the Australian species. Amongst the Malagasy species, he also resurrected names that had not been in use in Madagascar and that had been accepted by people working with Malagasy plants. He just did not find that this was encouraging stability. Now that the genus had been monographed, a great number of molecular and biogeographic papers that had come out subsequently using the name. He felt that what was now being proposed with the Example was that this be abandoned and we go back to a different name. He considered it a conundrum, but felt that if the group had been worked through, why throw out the name now?

**McNeill** thought that what was being addressed by Dorr was whether the Example was a good one, but if it was not a good Example then the Editorial Committee would not include it. But he argued that it should not affect the overall issue. The fact that someone had taken it up because he felt the *Code* at that time mandated him to do so and the name had come into common use, and we now clarify the *Code* and change that, to his mind, was simply a case where that name should be looked at seriously for conservation. He felt that the author had done it in good faith, but in order to have a clearer *Code*, and to protect a lot of other names, the Section might want to make the change. He thought individual cases should not be allowed to say we should not have a rule, if it was a good rule, simply because it seemed to be going back on a particular case at the moment.

**Brummitt** added that the case of *Capparis gibbosa* was the subject of a formal proposal to the Committee for Spermatophyta, which had made a recommendation, which would go through the process. It happened to be contrary to the monographer because there was such an outcry from the Australian people who knew the very well known species under a different name. He felt that there was really no argument; the decision had already been taken.

**Demoulin** did not think the discussion should focus on specific Examples but rather look at the general principle. And he felt that the general principle was indeed a good one. However, as the Rapporteur had pointed out, he thought it was bringing back the incidental mention by a back door in Art. 32 and it should really belong in Art. 34. He suggested that as long as the intent of the author was introduced as the main thing to decide, it belonged to Art. 34 and he thought it should not be turned around. He advocated coming back to the issue of incidental mention in Art. 34. He had quite a few additional cases that he had tried to put forward at the Berlin Congress, but at the time Greuter was so powerful and he wanted to kill incidental mention so the Examples were not accepted. He believed that it should go to a Special Committee, but he did not want to belong to it. [Laughter.]

**McNeill** asked if that was a formal proposal.

**Demoulin** replied no, it was your [McNeill’s] proposal.

**McNeill** had hinted at it, but was not proposing it.

**Wiersema** thought it was a slippery slope trying to include intent as a requirement. He felt it was just as difficult to determine intent in many of the cases as it was to determine whether there was a description or diagnosis. He did not see it as an improvement and thought it would be destabilizing to many, many names, if it was accepted and intent was required to be an issue.

**Davidse** wished to second Wiersema’s comments about the difficulty of intent. Even in these specific Examples, in at least three of them, a new name was provided and, to him, that was definitely an indication of intent. He agreed that it was going down a slippery slope if it was adopted.

**Barrie** agree with Wiersema and Davidse that it was often difficult to interpret intent. But he believed that was one of the reasons it was needed in the *Code* because presumably Prop. J was going to pass, which he thought was one of the most important issues before the Section. If it did he felt it would at least give some authority to the Committees to decide what that intent was, so when it was ambiguous, they could at least get a ruling on it.

**Nicolson** asked if he was speaking in favour of the proposal? [He was.]

**Perry** felt that, although it was difficult to define intent, it was surprising how often people could agree on whether or not an author intended to publish a name and she felt that that was really the crux of the matter.

**Prop. F** was **rejected** on a **card vote** (201: 254; 44.2% in favour).

**Nicolson** reported that the tellers had accepted cards with the wrong number but that they would no longer do so.

**Prop. G** (19: 97: 27: 14) and **H** (12: 95: 34: 14) were **withdrawn.**

**Prop. I** (31: 47: 64: 15).

**McNeill** suggested that Prop. I was a separate issue and could be considered in its own right, quite apart from any of the other proposals.

**Perry** added that it was simply a Note stating exactly what was in the *Code*. She thought it may be obvious to most people, but it might be helpful to have it in there.

**Nicolson** moved to a vote which very close and he ruled that it did not pass.

**Demoulin** pointed out that the majority vote in the mail ballot was for Editorial Committee and suggested that the Section should have the opportunity to vote for that option.

**McNeill** noted that the Rapporteurs did suggest that, as a Note, it was within the competence of the Editorial Committee to incorporate it. If the proposal was rejected, of course, they would not do that. He felt it was something that was implicit, that a diagnosis did not have to be separate.

**Nicolson** thought it was an interesting proposal and reported that there were 64 votes for Editorial Committee in the mail ballot, and that combined with the “yes” votes indicated favourable opinion of it. He took another vote on whether or not to send Prop. I to the Editorial Committee.

**Prop. I** was referred to the **Editorial Committee.**

**Prop. J** (43: 83: 7: 18).

**McNeill** moved to Prop. J which he noted had already been discussed a few times. The suggestion was that cases of doubtful validity be reviewed by the Permanent Committees in a manner analogous to cases where there was a question as to whether two names were sufficiently alike to be confused.

**Barrie** had mentioned earlier that he thought this was one of the most important proposals before the Section and wanted to explain why he had said that. He thought that most people may not realize it, but there was nothing in the *Code* giving the Permanent Committees the authority to rule on whether or not a name was validly published. He elaborated that Art. 12 said that a name had no standing if it was not validly published, and if a name had no standing, the Committees could not adjudicate them. He found it surprising how many of the proposals published in *Taxon* included a name, either proposed for conservation or against which a name was proposed for conservation, in which the question arose of whether or not the name was validly published. He argued that the Committees needed the authority to make that decision, before they could make a competent decision on whether such names be conserved or rejected. He strongly urged that this proposal be passed.

**Brummitt** had already spoken about the issue, so felt his views were known. He wished to draw the Section’s attention to the caution in the Rapporteur’s comments. They cautioned against the dangers of excessive workload to the Permanent Committees should this proposal be approved. He felt that it was far from that, and that the spermatophyte Committee was saying, “Please, give us the ability to take decisions. We’re not afraid of the work; don’t worry about that.” He argued that they wanted the ability to make a recommendation to some of these cases. So many cases came up where there was one of these nomina subnuda that would upset a well-established name and he outlined how somebody would submit a conservation or rejection proposal and the Committee was stuck, because they could not decide officially whether it was necessary or not. In the latter cases, he highlighted that it may have a knock-on effect on other names in the same list, and so on. He felt that the doubts expressed over the last proposals on nomina subnuda emphasized the fact that it was necessary that somebody had the power to resolve these cases. Otherwise, he suggested that they were going to drag on and on.

**Wiersema**, too, wanted to strongly support the proposal, because it avoided the need for some other proposals. He hoped that if this ruling could resolve the matter, it would eliminate the need for some conservation and rejection proposals.

For **Rijckevorsel** the previous comments brought to mind a different point. He felt that as the proposal was phrased, the Committee could only make a decision on the validity of a name if the proposal was submitted with that intent. He suggested that it may be wise to rephrase the proposal to indicate that a name proposed solely for conservation or rejection could be ruled as not validly published. He thought that it needed editorial attention, otherwise there would need to be separate categories of proposals and only if a name were submitted in the right category would the Committee be authorized to make a decision.

**McNeill** thought that the point he was making was probably editorial in the sense a name that had been proposed for conservation, which implied a particular status for another name, and which the Committee had to look at, was also being referred to it, albeit not for this purpose. He argued that there had been the appropriate referral and thought that the point could be addressed editorially.

**Marhold** did not want to see it restricted to names proposed for conservation and so forth.

**McNeill** clarified that he meant because they went through the same process, of referral to the General Committee and so forth, even though it was for a slightly different purposes. Where the question of valid publication was inherent in the proposal, he thought that, unless the Section was otherwise minded, this was sufficiently analogous to be broadened to cover that.

**Buck** wondered if there would be an index for these names, as there was for conserved and rejected names? He pointed out that, otherwise, within a lifetime, the Committees may be asked to rule on them a second time.

**McNeill** replied that there was no proposal for an index at the moment.

**Brummitt** responded that if it was a serious problem, he would add an index to the proposal.

**McNeill** wondered where the index would go? He noted that there was an index to decisions on whether or not names or epithets were sufficiently alike to be confused, maintained on the web in a voluntary capacity by the President and he added that it was a very useful index. He suggested that it should be indicated what mechanism should be used, e.g., whether it should be in the *Code*, or on the web.

**Brummitt** thought it was very comparable with other cases mentioned and should presumably be in an appendix to the *Code*.

**McNeill** pointed out that that would be different from the situation with confused names, where only a small number were in the *Code*.

**Brummitt** felt that, so long as the decision was ratified by the General Committee and appeared in the reports, it should be available. Then if some person, like the President at the moment, was willing to continue the invaluable work that he did and keep up an index, so much the better. But he retracted what he had said about putting it in the *Code*. It was not comparable with conserved or rejected names. So long as someone produced an index, that would seem to solve the matter.

**McNeill** checked that it was not going to be part of the proposal?

**Brummitt** confirmed that was the case.

**Nic Lughadha**, although she had not consulted with her Harvard and Canberra colleagues, thought that IPNI could safely offer to flag those names ruled by the General Committee as being not validly published. She added that IPNI was available on the web, though IAPT may want to have them available elsewhere also.

**Demoulin** was not worried by the fact that some proposal might enter the pipeline under the wrong label. In his Committee, at least, and he thought the others had been doing it, they sometimes corrected things and got the advice of the General Committee in situations similar to this one. He thought that it would make things easier for the Committees, to have the option. He suggested they could say to a proposer, well, you should not ask for conservation, you should ask for a ruling on validity under this special provision.

**Redhead** also favoured the proposal, but thought that it may be necessary to add another Article or so in the *Code* to give the Committees the authority to deal with the problem. He was not certain it would be covered solely by the suggested insertion and noted that it may have to appear elsewhere in the *Code*. As an aside, he had once asked the fungal Committee to rule whether a form was a teleomorph or an anamorph and the answer came back that the Committee did not have the authority to make such a decision. He felt it was similar to this validation issue. He supported giving the Committees the power to do something.

**McNeill** felt that it clearly was an interesting proposal, and the arguments in favour of it were well presented. However he felt he must point out to the Section that it would mean taking a new, unique step for botanical nomenclature. He explained that it would be the first time that there had been anything within the *Code* that had allowed interpretation of the *Code* by a Committee as up until now, adopting procedures of the zoological *Code* had been avoided, for example, in which the zoological Commission had all powers. He highlighted that that Commission could suspend any aspect of the *Code* for any particular case, not confined to conservation and rejection. He acknowledged that it may very well be the way forward, but thought that the Section should understand that they were putting an entirely new concept into the botanical *Code*. He went on to say that what there was at the moment with regard to judgment as to whether or not two names were sufficiently alike to be confused was a judgment of whether we as individuals were confused, a human judgment. He argued that this change said: “Is this what the law says?” and would establish a procedure by Committees. He thought, in the circumstances it was, practically, the best way forward, because in practice the Committees did have to do this and they did it simply because they either decided to reject a name or they decided that conservation was unnecessary. By enshrining it here, it would permit an approach before a conservation proposal, so he felt there was a lot of merit in it, but he thought it was his job to point out that it was an entirely new concept in the *Code*. The one thing that worried him was consistency of application and he felt that the General Committee would have to look carefully at the early decisions. He elaborated that it would be intolerable if the fungal Committee, for example, interpreted the *Code* differently from the algal Committee. He thought it was a situation which would have its teething problems, but, as the Rapporteurs said, if this was the price to pay for stability, it was probably a worthwhile price.

**Nic Lughadha** suspected that McNeill was making distinctions that most of the Section would not normally make. She certainly understood that a ruling by a Permanent Committee on whether or not two names were confusable to be a verdict by the Committee as a whole and not an expression of the individual opinions of the Committee members. She expected that verdicts on nomina subnuda would be seen in the same light.

**Redhead’s** feeling, given McNeill’s comments about the expansion of the whole concept and that there may be other cases, was that there should be an Article elsewhere in the *Code* to empower the Committees. He wondered whether the Section should entertain the possibility of forming a Special Committee to look into the question of giving additional powers to the Permanent Committees and write the appropriate Articles.

**McNeill** thought that what he was suggesting was that there should be something in Art. 32.1 allowing the proposal to override Art. 32.1, which it was not clear that it would do. He asked if the proposal for a Special Committee had been seconded. [It had not and was not.]

**Prop. J** was **accepted.**

**Prop. K** (2: 152: 4: 0) and **L** (2: 153: 3: 0) were ruled as **rejected.**

### Recommendation 32B

**Prop. A** (23: 61: 57: 12) was ruled as **rejected** as it was a corollary to Art. 32 Prop. B or C which were rejected.

### Recommendation 32F

**Prop. A** (9: 129: 4: 15).

**McNeill** reported that Rec. 32F Prop. A received more than 75% “no” votes and was ruled as rejected.

**Perry** asked that Rec. 32F Prop. A be reconsidered.

**McNeill** agreed if there were five people to support it. [There were.]

**Perry** wondered if the text could be rewritten “Botanists should consider proposing works...”

**McNeill** checked that that was instead of “Botanists should propose works..?”

**Perry** confirmed that, adding that unfortunately, that was the original wording and it somehow got changed in editing. She explained that it was just there as a reminder that this might be a way of dealing with works that were particularly offensive, that contained lots of names that could be seen as nomina subnuda and that had not be taken up.

**Nicolson** queried if the works would be added to App. V.

**Perry** confirmed they would.

**Nicolson** clarified that App. V was the “Opera utique oppressa”.

**P. Hoffmann** thought it was very obvious that if there was an Appendix to the *Code* listing suppressed works that such publications could be added to it. She did not think an extra provision to say this was needed. She argued that it would just clutter up the *Code* and urged rejection.

**Prop. A** was **rejected.**

### Article 33

**Prop. A** (140: 3: 15: 0).

**McNeill** moved to Art. 33 Prop. A which was a proposal to add an Example to the Article. He reported that it had received very heavy support, 143 “yes”, 15 No. He added that it would, in fact, be an Example added by the Editorial Committee and it was not necessary, nor would it be appropriate, for it to be a voted Example.

**Schafer** considered that, given the time of publication, it was very clear that Tuckerman described it as a new subspecies for *Erioderma chilense* and he did not think that the author had any doubt that the subspecies was not connected to *Erioderma velligerum*.

**McNeill** responded that it was quite clear that his action was not in accord with Art. 33.1, as currently written.

**Hawksworth** noted that it was a situation found in Theodore Magnus Fries as well. He added that there were other cases and it could often depend on the layout, giving the example that it was not uncommon at the time for lichenologists to place such names underneath the species that was intended in the layout. He pointed out that these had been accepted as validly published in those ranks and he was not be happy with the proposal without further study on how many names might be affected.

**McNeill** agreed that, if names were indented under the species name, it fulfilled the requirements of Art. 33.1 and would not be affected, but he had looked at this case and could find no way in which it reflected the Article, albeit the intent was clear.

**Per Magnus Jorgensen** explained that it was a case he had come across when he worked on the genus. He was uncertain what to do with it, according to the *Code* and thought at the beginning that it was valid, but now he was absolutely convinced that Tuckerman did not associate the names despite having a taxonomic opinion about it, but that was a different matter.

**Ahti** was unhappy about the Example. He argued that if the Section wanted good examples of subspecies described without indicating under which species they should be placed, there were lots of good examples under *Hieracium* in Sweden and Finland, where many taxa were recognized at the rank of subspecies in the 1800’s. He felt the suggested Example was very unusual and perhaps questionable.

**Nicolson** had a question for Jorgensen: was the “combinatio-valigerum” a species combination or was that his subspecies?

**Per Magnus Jorgensen** replied that that was the problem and it was not possible to use the *Code* in this case which was why he had approached McNeill about the question. McNeill thought that it was not valid and Jorgensen thought that it was needed as an Example, maybe a voted Example.

**Nicolson** confessed that it did not occur to him that it was not anything but a species name for which the author had neglected to give the subspecies names.

**Per Magnus Jorgensen** believed that what had happened, was that Tuckerman originally thought it was a species but changed his mind while publishing. The type said “sp. nov.”, but he published it as a subsp. nov. which was not a misprint; it was a taxonomic decision and the ruling was about the names, but he clearly did not associate the [specific and subspecific] names which is what had caused the muddle.

**Hawksworth** noted that there were some examples, Saccardo used to do it as well. He thought it was a dangerous idea without more research.

**McNeill** suggested that as there was a strongly positive mail vote, the Section could refer it to the Editorial Committee. His guess was that there would be a lichenologist on it. If this Example was not deemed a suitable Example, the Editorial Committee would add another suitable Example, say a Fries or Saccardo case, where by indentation or other indication the fact that it was associated was illustrated. But that would be a matter of editorial judgment, if the Editorial Committee deemed this Example suitable for inclusion. Given the wide support, he moved that it be referred to the Editorial Committee, but not as a voted Example.

**Per Magnus Jorgensen** offered another Example from the genus.

**McNeill** suggested sticking with the Examples provided, but took the opportunity to note something he would normally have mentioned later; the submission of Examples was welcomed, not just from [matters arising] this week, but also of other items in the *Code*, where people felt that other Examples would be beneficial. He outlined that they could be sent to him or to Turland in the next month or so and exhorted submitters to be sure to provide full documentation.

**Turland** added that a scan of the text or the protologue would be most welcome.

**Prop. A** was referred to the **Editorial Committee.**

**Prop. B** (134: 17: 6: 1).

**McNeill** introduced a series of proposals by Zijlstra and Brummitt, noting that the first, Art. 33 Prop. B, received a very favourable vote.

**Brummitt** explained that the present Art. 33.2 arose from proposals by Zijlstra and himself at the last two congresses, at the last Congress the *Scaveola taccada* Example went straight through and the Section had agreed on the general principle. Since then, further Examples had come to their attention and he and Zijlstra were almost requested by the Rapporteur to look at it and improve the wording. One of the problems he highlighted was that generic names were not combinations, so the rules that would apply to a combination would not apply to a generic name that was based on a subgeneric name. He explained that the wordings related to that and they were really just tidying up the wording of all the Articles.

**Demoulin** had some reservations about the proposals. If they were editorial and if nothing was changed in the *Code*, then he was not convinced that the Article would be clearer. He preferred to maintain things as they were. His main problem was that in Prop. B, prior to 1953, an indirect reference could be anything and an erroneous reference was an indirect reference. He did not think that an indirect reference was logically the same as an erroneous reference. He argued that in the Article as it was now, they were clearly two different things., In his opinion, the 1953 date was not really relevant to erroneous references. He thought it would become especially important for mycologists when the discussion moved to Prop. F, which depended upon Prop. B because there, there was something that had nothing to do with 1953. He conceded that it was possible that he could live with it, but he would need full assurance from the Rapporteurs that one may consider errors in citation as indirect reference, even if there was nothing in the erroneous citation that could lead indirectly to the good one.

**McNeill** did not think that Brummitt meant this. He argued that the proposals were not purely editorial, they were changes to the rules that were not in any way fundamental, except possibly for one or two, but they were ones that extended the rules in a logical fashion. He elaborated that the current wording dealt only with combinations, but generic names could have basionyms and generic names were not combinations, so it dealt with that oversight in the rules. He highlighted that the other change that was being introduced, in an attempt to clarify the Article, was to make different sets of proposals for the period prior to 1953 and for the period from 1953 on as, currently, there was some intermixing. He felt that this particular element was not really covered; it was just taken for granted, what would happen prior to 1953. He thought that summarized what the proposers were trying to do, but felt the Section could discuss it further with the individual cases.

**Zijlstra** thought she should mention one important point that was also a rule; In Art. 33.2 at the end it read “...if it would otherwise be validly published as the name of a new taxon”. If this rule can be accepted for the name of a new taxon, why not accept it for a nomen novum?

**McNeill** pointed out that that was not in Prop. B, but one of the other proposals.

**Gandhi** reported that since the St. Louis Congress, for North American names, in many cases he and his colleagues had been applying Art. 33.4, even though there was no indirect reference. He noted that there were several examples in Alphonso Wood’s *A Class-book of Botany* where, for several infraspecific names, it was not possible to trace any indirect reference to the previous names. However, just based on the identification of the literature and the taxonomic circumscriptions, they thought they were taxonomic synonyms. Prior to the publication of these standards, they had treated them all as taxa nova and as taxonomic synonyms. Since the St Louis Congress they had been treating them as either stat. nov. or comb. nov. His concern was that giving this Article a starting point of 1953 may require them to reverse their previous decisions.

**Nicolson** asked if he had an estimate of how many names were affected, wondering if it was hundreds or tens?

**Gandhi** estimated tens.

**Malecot** offered the information that currently Art. 33.2 was very difficult to apply to some old literature. He explained that when you were looking for a publication you had to decide whether it was the correct publication for the new taxa but you also had to make the taxonomic judgement that the taxon in the first publication and in the second were the same taxon. He argued that it was not always easy to compare descriptions in the old literature. He felt that the current proposal provided help in applying the Article, and was in favour of it.

**Barrie** asked for a point of clarification from Gandhi, wondering if he said names after 1953 or names before 1953? [Before 1953.]

**Prop. B** was **accepted.**

**Prop. C** (65: 75: 11: 0).

**McNeill** introduced Prop. C as the proposal to which several people had already referred, dealing with the rewording of Art. 33.2. He thought it was a very sensible extension, also dealing with generic names although he noted that it did not fare as well in the mail ballot.

**Brummitt** thought the comparative failure in the mail ballot was due to Prop. 33D, which had split the vote. He noted that, although the Rapporteurs comments attributed the proposal to Zijlstra and himself, it was not written by them, it was added by the Rapporteurs. He and Zijlstra had discussed 33D at some length and failed to see the point, because everything was different before and after 1 Jan 1953. He argued that what was suggested in Prop. D could not possibly happen, because after 1 Jan 1953 the requirements for new combinations and nomina nova were very strict, so he did not see the point of Prop. D and believed this was why the vote was split between Props C and D.

**McNeill** responded that the Rapporteurs had made quite clear that Brummitt did not write the proposal but the attribution in the reference to where it could be found, of course, remained the same. He explained that the reason they had put it in was that there had been some discussion in St. Louis and the point was made that there had been application of this to names published in the post-1953 period, although the thrust of the Article was toward pre-1953 names. The point was made, he thought by the previous Rapporteur, and possibly for that reason a comparable proposal had been defeated. He thought it was perhaps important to give the Section the choice of whether to have the clarified wording without the date restriction, or to have the wording exactly as proposed. The interesting thing was, and he found it quite bizarre, that the mail ballot totals were identical for the two proposals!

For **Zijlstra** the most essential parts were still included in Prop. D, but she preferred C. She suggested that if people were confused by the date in Prop. C, the Section could vote on Prop. D first and, if accepted, then vote on C. Regarding Prop. D, she had noticed that in the original proposal [from St. Louis], that was now in Art. 33.6, it ended with the wording “...even if published on or after 1 Jan 1953,” but the “even if” was not in the original proposal.

**McNeill** asked for clarification that she was suggesting that it was not in the original proposal?

**Zijlstra** replied that was so for St. Louis. The proposal that became Art. 33.6 did not include the addition.

[Lengthy pause.]

**McNeill** explained that the Rapporteurs were discussing whether or not to withdraw their proposal.

**Brummitt** wished to reiterate that he did not understand Prop. D, because it could not possibly apply after 1 Jan 1953, because there were a whole raft of restrictive requirements; you had to cite the date and place of publication, and so on. He maintained that it could only happen before 1 Jan 1953, so Prop. C would seem to be the one to go for.

**Turland** pointed out that in Art. 33.3, on the last line, there was a reference “(but see Art. 33.2)” and he wondered if that did not imply that Art. 33.2 was an exception to the requirement of Art. 33.3 and the date requirement for a full and direct reference?

**Brummitt** felt that if that was the intention, then he would suggest that the Editorial Committee delete the reference to 33.2 from the end of 33.3, because that was nonsensical.

**McNeill** thought that was the point, the thing the Section could rationally talk about and the basis for their proposal. He suggested that if Prop. C was accepted, then they would delete the reference in 33.3 and if Prop. D was accepted, the reference would no longer be necessary. He thanked the Vice Rapporteur for pointing out that at the moment Art. 33.2 applied even after 1 Jan 1953. He gave the example that if a person clearly made a new combination but did not meet the requirements and it would otherwise be a validly published name, then Art. 33.2 applied, even if it was published after 1 Jan 1953. He felt that the point was to avoid having names with the same epithet in two different genera, obviously based on the same taxonomic concept and conceivably having two types as a result, which he felt was the basis for 33.2 in the first place. The point that the Rapporteur made in St. Louis was that it could apply to post-1953 names, albeit rarely. He thought that the Section should follow Zijlstra’s suggestion and vote first on Prop. D, and if that was passed, then move to Prop. C. He added that the date could be inserted or not, as the Section decided.

**Brummitt** suggested just making a clear distinction between names before 1953 and those after.

**McNeill** interpreted that as a clear indication that Brummitt supported Prop. C.

**Nicolson** moved onto the proposal to take up D first...

**McNeill** interrupted to say that, actually, he thought it might be better to take up C, because if C passed, D fell.

**K. Wilson** had ended up totally confused. McNeill had just said that Art. 33.2 applied now, not just before 1953 but Prop. C would make it apply only before 1953. She requested clarification on whether or not it should apply after 1953.

**McNeill** replied that that was for the Section to decide. He explained that at the moment, Art. 33.2 applied up to the current day and what Prop. D did was to accept Brummitt & Zijlstra’s modifications to the wording while retaining the applicability of the Article to post-1952 names. Personally he thought the changes were an improvements. On the other hand Prop. C had the same improvements of wording, but would restrict the application of 33.2 to pre-1953 names.

**Wiersema** supported Brummitt’s position and thought the date was necessary. He could see situations where someone did not intend a new combination, but were simply publishing a new name, but it ended up being one and therefore the type was changed because someone could invoke 33.2 after 1953.

**McNeill** wondered why that would be bad if it was a presumed new combination, adding that there had to be some link between the two names.

**Wiersema** replied that anyone could presume that it was a new combination, but the author of the name may not have made that presumption.

**Zijlstra** added that the actual case was that authors considered their new combination so self-evidently based on the basionym that they neglected to mention it. She clarified that it was not the reverse, that an author not intending to do so might publish a new combination.

**Brummitt** had a feeling that some of the problems would be resolved by Prop. G, which covered the case where something that was obviously intended as a new combination was made, but the author accidentally omitted, say, the date of publication, but cited a heterotypic synonym with a full reference. He outlined that the proposed new combination would be validly published as a nom. nov. with a different type. He thought that this was part of the problem that was being discussed.

**McNeill** noted that the Rapporteurs had made the comment that these were alternative ways of proceeding in the matter. They felt that it would be much more sensible to have the same type, which was what Prop. D would do, whereas Prop. G would do something different.

**Brummitt** explained that Prop. G would keep the type for the new combination.

**McNeill** pointed out that the Section was not yet discussing Prop. G, but it did something unusual in that it would treat a name as not validly published even if it would otherwise be validly published which he felt was just a little strange.

**Brummitt** responded that that was because otherwise you would have something that was intended to have one type validly published with a different type.

**McNeill** felt that the point was that they agreed on the problem, but offered different solutions, Prop. D or Prop. G.

**Barrie** needed some clarification as he was a little confused. He thought that 33.3 prevented 33.2 from applying after 1952? He wondered how could Art. 33.2 apply after 1 Jan 1953?

**McNeill** argued that it was because of the cross-reference, “(but see Art 33.2)”.

**Barrie** queried if this meant that Art. 33.2 contradicted Art. 33.3?

**McNeill** replied that that was what “but, see” meant.

**Barrie** suggested deleting that.

**McNeill** agreed that that was what would have to happen if Prop. C passed.

**Zijlstra** felt that the confusion of Barrie illustrated exactly why the strict division on what happened before and after 1953 was needed. She argued that then those working with earlier names could apply one Article and authors working with later names could apply other Articles.

**McNeill** reiterated that this was one of the thrusts of the set of proposals. He thought the Section had a reasonable choice and either solution would work. He added that Prop. D was closer to the current rules and Prop. C would require an additional change.

**Nicolson** found it interesting that Props C and D had such equal representation. He ruled that since C came first, it would be voted on it first.

**Prop. C** was **accepted.**

**Prop. D** (65: 75: 11: 0) was **withdrawn.**

[*The following debate, pertaining to a New Proposal on Art. 33 by Demoulin regarding later starting points took place during the Ninth Session on Saturday morning*.]

**Demoulin’s Proposal**

**Demoulin** indicated that the Committee for Fungi would like the Editorial Committee to pay particular attention to the provision in Art. 33.6 relating to later starting points, so that it was treated in a way that was clear to all mycologists. Because of what the Section had done on that Article, it could be a little more complicated for them.

**Demoulin’s Proposal** was referred to the **Editorial Committee.**

[*Here the record reverts to the actual sequence of events*.]

**Prop. E** (94: 22: 36: 0) was referred to the **Editorial Committee.**

**Prop. F** (97: 30: 27: 1).

**McNeill** noted that Art. 33 Prop. F was predicated on things that had already passed and felt it could certainly be considered in its own right.

**Demoulin** thought that 33.6 was one of the clearest parts of Art. 33, and it would be made less clear by this proposal, which he found totally unnecessary. He argued that efforts had been trying to simplify the *Code* until now; and the proposal would complicate it. He concentrated on the part he knew best, paragraph B which referred to the situation of fungi with a starting point that had been changing. It was illustrated by Ex. 12, which he advised non-mycologists to read attentively. He felt that the situation now was quite easy to understand for mycologists with this problem, and the date 1 Jan 1953 had absolutely nothing do with it. He thought the wording in the *Code* made it clear that it was a general situation that applied before and after 1953. He maintained that if the proposal was approved, then for post-1953 names, the situation would be unchanged; but for pre-1953 names, it would be necessary to refer back to Art. 33.1, to discover that it was the same thing! He elaborated that this was because under Art. 33.1, before 1953, you could have considered it an indirect reference or an erroneous reference, which was the same thing. The problem, he felt, was that you had to make two steps, when up until now there had been a single, clear step! He warned that, for a lot of mycologists, it was necessary to have clear instructions, otherwise they got completely mixed up! [Laughter.]

**Brummitt** explained that the intention of Prop. F was to get rid of the word “reference”, because the word was completely ambiguous. He continued that 33.6 said, “In any of the following cases, reference to a work...” He wondered if that meant a solid reference, like “Taxon 53, page number and date” or was it used in a general sense? He argued that the word was very ambiguous in the Art. 33 paragraphs.

**McNeill** highlighted that there were two elements, and the one Demoulin objected to was the insertion of the date and the other component of the proposal was to change the phrase “In any of the following cases, reference to work...” to “In any of the following cases, a full and direct reference to work...”, which was also predicated on the change of date.

**Brummitt** pointed out that the Article could only apply after the first of January 1953 because before that any reference, direct or indirect, was appropriate. He felt that the date did not really matter and was just automatic because, before 1953, anything goes. For him, the point was to try to focus on when and where the Articles applied Art. 33.6 before 1953 because any indirect reference should go there.

**McNeill** summarized that Demoulin took the view that it did matter because that was the basis for dealing with pre-starting point names.

**Demoulin** did not like it this way, but if the Section wanted it to be there, he suggested separating paragraph B from the rest of 33.6. He hated to do that, but maybe it would be useful.

**McNeill** asked if that was a proposal to separate part B as a new Article?

**Demoulin** did not mean a separate Article but a separate paragraph.

**McNeill** replied that that was what he meant.

**Demoulin** agreed, clarifying that he was suggesting making subparagraph B a separate paragraph to indicate that it had nothing to do with 1953 as a friendly amendment.

**Zijlstra** did not accept it as a friendly amendment. She pointed out that the Section had accepted, with Prop. C the date of 1953. She saw no reason to amend it, if the Section accepted that one set of rules should be before the date and one set of rules after the date.

**McNeill** could see the argument that Zijlstra and Brummitt had put forward: that in some of the Articles there was no clear provision in Art. 33 for what was necessary for a valid publication of a combination prior to 1953. He summarized that the new clause they proposed dealt with that, so that was a clarification. However, he was not convinced that doing anything to the Article, particularly when there had been some doubts expressed as to its application, was really necessary to ensure the completion of the package. He thought it may be slightly untidy from the proposer’s perspective, to leave Art. 33.6 applying across the spectrum, but he failed to see how it in any way weakened the impact of the proposals they were making. He thought that they would still have all they set out to achieve, even if the proposal was defeated.

**Zijlstra** reiterated that their Prop. F was to adhere strictly to the requirement of Art. 33, that after 1952, a full and direct reference was needed. They felt that Art. 36 seemed to open exceptions and she had no examples where such an exception would be useful, so wondered why Art. 36 should apply after 1952? She suggested that if there were exceptions, they could be corrected under Art. 34.

**McNeill** did not follow her comment. He was suggesting that if the proposal were to be defeated, or if they withdrew it, it would not affect the thrust of the set of their proposals except to leave one Article covering the whole span instead of having them all divided between the two periods. In view of the concern that had been expressed as to whether this would make it a bit less clear how to treat some names in which there was an incorrect citation pre-1953, he felt it might be harmless just to leave it. He failed to see, apart from tidiness, what was being gained.

**Wiersema** had often found it rather difficult to decide to what time period this Article applied. He suggested that if it was decided to keep it applicable before and after 1953, it would be useful to reword it in some way to make it clearer that it applied to both time periods.

**McNeill** thought that once you read to the bottom of it, it was clear, though he acknowledged that it was not obvious up front.

**Brummitt** repeated that Art. 33.6 must apply after 1 Jan 1953, because before that, anything went. He argued that all of the very restrictive cases could only apply after 1 Jan 1953.

**Demoulin** thought he had made it clear at the beginning that it would be possible to live with the system of dividing everything into before and after 1953, but it was a big step backward in having in clear provision, at least in this case. He felt it was a case of great importance for a lot of mycologists and instead of having one rule and one Example, they would now need a Note and an additional Example introduced into Art. 33.1, with a case that was before 1953. Otherwise, he thought that the mycological community would not understand what to do.

**McNeill** summarized that the point was that acceptance or otherwise did not actually change the *Code*, but, in some people’s view, it clarified it by making a clear-cut division in date. In other people’s view, it made things more difficult by obscuring the fact that certain provisions applied throughout time, even though only through another Article could one see that they had to.

**Prop. F** was **accepted.**

**Prop. G** (58: 80: 16: 0).

**Brummitt** introduced Prop. G which covered the accidental publication of a new combination without the relevant data, but with a heterotypic synonym in synonymy. He felt it was ridiculous to treat the proposed new combination as a nom. nov. with a new type.

**McNeill** pointed out that, having defeated Art. 34 Prop D, it was important to approve this proposal.

**Redhead** was confused about it before, but as it was explained, the intent was to prevent accidental publication of a nom. nov. when attempting to publish a new combination. He pointed out that, as written, it seemed to say a new combination OR a nom. nov., which was not what was explained. If the concern was that a new combination would end up an unintentional nom. nov., he suggested moving “nom. nov.” from where it was in the proposal to someplace near the end so that it read “...which was validated as a nom. nov.” This was based on his interpretation that the concern was converting a comb. nov. to a nom. nov. by accident.

**Brummitt** felt that if there was a problem he was sure the Editorial Committee could work out appropriate wording.

**McNeill** did not think Redhead’s problem was real in that he was describing an avowed comb. nov. or avowed nom. nov., while the nom. nov. that Brummitt was talking about was the accidental one, from citing a heterotypic synonym. He felt that it was simply making it clear that if people did not do the right thing after 1 Jan 1953, their name was not validly published. He argued that if the Section was going to do anything about it, they should either treat it as a new combination or nom. nov. despite the error, or treat it as not validly published, which meant the first person who came along and treated it properly was quite free to do so. He added that the name did not exist until that time. He thought it was clear enough, but assured the Section that the Editorial Committee would look at it.

**Nicolson** moved to a vote and ruled it passed, although without great enthusiasm.

**Prop. G** was **accepted.**

**Prop. H** (107: 27: 20: 0).

**McNeill** noted that Art. 33, Props H, I and J addressed a small but, for indexers, important point.

**Challis** explained that Prop. H would ensure that all relevant information for publication of a new combination or nomen novum was actually provided in the place of publication. She gave as reason that at the moment, one could indicate a new combination or nomen novum by providing a full reference. They [at IPNI] felt that there was some confusion over the issue. She wished to make a friendly amendment to her own proposal, adding the word “full”. It would then read: “A new combination or nomen novum published on or after 1 Jan 2007 was not validly published unless its full basionym or replaced synonym was cited.” She explained that this was for cases where the basionym was only partially referenced, only the infraspecific part was referenced and she had some examples.

**McNeill** wondered what the difference was between a basionym and a full basionym?

**Challis** elaborated that she had come across cases where someone had published a new combination where the basionym was an infraspecific name and only the infraspecific part was cited, the genus and species epithet were missing.

**McNeill** deemed that then the name was not cited as the name of an infraspecific taxon was a combination of a species name and an infraspecific epithet, joined by a connecting term and they had only cited the epithet, not the name.

**Challis** understood.

**McNeill** was worried that “full basionym” would mean that if, in a monograph on *Poa*, say, someone said “*Poa pratensis* subsp. suchandsuch” that would be ruled out because they did not spell *Poa* out. He thought she should keep with the original wording.

**Watson** had a problem with the way Art. 33.3 was currently phrased. He noted that the basionym or replaced synonym must be indicated in two steps: Step A, clearly indicating it and, B, a full and direct reference to its author and place of publication. He felt that if you were fulfilling step A by doing step B, it did not really make sense. He suggested that the words “clearly indicated” should be replaced by “clearly cited”.

**McNeill** wished to confirm that what he was suggesting was that the existing Article should essentially pick up the wording of the new proposal, insert it within the Article, thereby making it more restrictive?

**Watson** agreed.

**McNeill** asked if that was a formal amendment, so that, instead of adding an extra clause, that the existing wording, “is clearly indicated,” should be changed to “cited.”

**Watson** agreed that it was. [The **amendment** was **seconded.**]

**Brummitt** pointed out that that would be retroactive, whereas the present proposal accepted that the word “indicated” had always been ambiguous and could be argued. The intention of Challis’s proposal was to avoid the ambiguity of the word “indicated” in the future by inserting a starting point.

**McNeill** felt it was a matter of whether you wanted it to be retroactive or just for the future.

**Marhold** commented that if it passed, some Examples would be necessary, because those who had not read the commentary may not understand the difference between clear indication and citation. He guessed that those who were not present at the Section would not understand the difference.

**Zijlstra** added that a clear indication could be to use the English name of a species and give a full and direct reference to the place where the basionym was published without citing the Latin name of that species.

**Nigel Taylor** was concerned that, if the amendment was passed, there would be uncertainty about a considerable number of names where indexers had not been sure how to interpret the term “indicated”. He strongly advised the Section not to accept the amendment.

**K. Wilson** did not think it was only the people outside the Section meeting that had a problem with the difference between “indicated” and “cited”. Her suggestion was that they be included in any glossary.

**McNeill** thought that “cited” was quite clear; and “indicated” was much less clear. He argued that, to be cited, you have to put it there, but clearly indicated, means there was no doubt what was intended but it was not cited.

**Printzen** asked if passage of Prop. H would mean that from 2007 onwards the exceptions mentioned in 33.4 and 33.6 were no longer valid?

**Nicolson** responded that it was his understanding that from that point on, it would be tighter.

**McNeill** repeated that the amendment was to replace the present wording “indicated” in Art. 33.3 by “cited”.

**Watson** withdrew the amendment, as prior to the discussion, he was not aware that there were other forms of indication beyond citation.

**Prop. H** was **accepted.**

**Prop. I** (100: 29: 25: 0).

**McNeill** noted that the correct wording of Prop. I did not appear in the Synopses of Proposals and it was displayed on the board. He added that the proposer assured him that the errors in the Synopses were not substantial and did not affect the meaning of the proposal, therefore the Rapporteurs comments, which were positive, remained relevant.

**Challis** wished to comment before too much time was spent on the proposal. She explained that they had submitted the package of proposals to try to clarify when it was necessary to cite the basionym or replaced synonym. Now that Prop H had been passed, she felt that it was clear that before 2007, as long as the basionym or replaced synonym was indicated, there was no need to cite it. So she was happy with Art. 33.4 as it was in the *Code* and was happy to drop the proposal.

**Prop. I** was **withdrawn.**

**Prop. J** (101: 24: 29: 0).

**Challis** introduced Prop. J as an Example that would add some clarification. She added that there was no example of omission of a basionym and she thought it would be useful to have one in the *Code*.

**Nicolson** commented [referring to the title of the publication in the Example, “Dumpling & His Wife: New Views Gen. Conophytum”]: that she had the strangest botanical literature! [Laughter.]

**McNeill** suggested referring the proposal to the Editorial Committee, to add levity, if not brevity, to the *Code*!

**Prop. J** was referred to the **Editorial Committee.**

### General Discussion on Misplaced Ranks Package of Proposals

**McNeill** suggested a preliminary presentation on a series of proposals on misplaced terms.

**Kolterman** agreed it might be useful to hear a presentation, so he could think about the proposals and be more prepared in the morning.

**McNeill** invited Moore to talk about the general issue and perhaps allude to the specifics, rather than to just one proposal.

**Moore** had dealt with the issue in question involving misplaced ranks for quite a while. In fact, he had first encountered it in graduate school. He came across a number of cases of this and sent it to about six taxonomists who were experts in nomenclature, and he received back about 12 opinions on how to apply the relevant Article. At the time he kind of gave up on it and ignored it. He recounted a small, funny story: Living in the United States, he had come across an issue involving baseball, in which they had line-ups where they must follow the correct batting order. There was one game where they didn’t follow the correct order and it got a lot of attention so the rules were published in the newspaper. As he read about it, he realized, my God!, this was what he needed to be looking at, because they had been looking at this problem for quite a long time. So he found studying the rules of baseball to be a big help in sorting out the issue of misplaced ranks! He noted that, in applying it to botany, there were a few things to think about. He planned to attempt to break it down for the Rapporteurs, too, so that the Section could take the proposals up to some extent separately. First off, he started with the issue of misplaced ranks and exactly how to deal with them. He outlined that the problem with the current Article was that it just said, basically, that a name published with a misplaced rank was not validly published. However, the problem was that if you had a sequence of rank-denoting terms and stuck one in out of place, there really was not just one misplacement, it could be interpreted to be multiple misplacements. He explained that it was not really clear exactly how to treat it, in most cases, because of the relative nature of the ranks. If you put in one mistake, there were also mistakes above it and below it.

He thought that the second issue could be characterized as the colloquial or informal usage of ranks which occurred a fair amount in the early literature. He noted that there was now a fairly rigid set of rank-denoting terms that we were required to follow. Linnaeus, however, used only about five or six ranks. It wasn’t really until maybe the 1900’s that we begin to get the sequence of rank-denoting terms that we have now begun to be used. So in the earlier literature, there were many cases of what we now treat as formal ranks in an informal manner. One of the examples was Bentham & Hooker’s *Genera Plantarum*, where the term “series” was used at a number of different hierarchical levels. He thought it was possible to reduce the number of cases of misplaced rank-denoting terms and better reflect the history of the situation by introducing the suggested concept of informal usage into the *Code*. He felt it would clear up a lot of problems and the way he had proposed it was that if someone was using a rank-denoting term at multiple places in the hierarchy, it could just be passed over and those were not considered to be part of the formal ranking scheme.

He outlined that, lastly, the problem that had to be addressed was the rare case, though it did occur, when there was sequential usage of the same rank denoting term, but clearly done in a hierarchical sense. He gave the example of putting a species within a species or a subspecies within a subspecies. In his first paper on the subject, in the draft he figured, well, everything will have to be rejected because there were species within species. He talked a lot to others and the general consensus after a lot of thinking on this was that, no, those really do not represent misplaced rank-denoting cases, rather there was a hierarchy within a given rank. He went on to say that another problem that could arise if such cases were recognized as misplaced rank-denoting terms was that sometimes it was not obvious when the situation existed as the hierarchy may be indicated by indentation and other, subtle methods. He suggested that if the Section went the other way and declared those to be misplaced rank-denoting terms, there would be the problem in some cases that the situation was not clear, but if the Section went the way that he proposed, it was clear that they were not misplaced. It was before his time, but in one of the earlier *Codes* there was an example, involving Gandoger’s species names which were declared to be species within species names and invalidly published as a result of that. However, that had now been removed from the *Code* and Gandoger’s work at the species level had been suppressed.

That concluded his quick overview. In terms of how to take it up, he suggested discussing the general topic of misplaced ranks, which involved Props 33K, 18G, 18H and 19D. Then take up the issue of informal usage and Props 33N, 33O, 16E and 35A. And sequential usage followed in Props 33L and 33N.

In terms of ranking the issues, he actually thought that the informal usage was the most important because that, in his experience, would clear up a lot of the cases. In many cases, division or forma or section or series were used in an informal sense. He felt that if the Section got that in, then the other cases were much rarer.

[*The report writer noted a great comment slip, the commentator succinctly summarizing what was said and even helpfully referring to himself in the third person: “An overview was given on his set of proposals*”. ]

## Fifth Session

Thursday, 14 July 2005, 09:00–13:00

**Nicolson** wished the Section a good morning and asked everyone to notice his t-shirt which said “Botany Rules”, although he was sad that it was not “Nomenclature Rules”.

**Stuessy** made an announcement about Demoulin’s meeting of the Committee for Fungi to which all mycologists were invited. He outlined that after a short business meeting there would be a discussion of general mycological issues at the Section.

**McNeill** referred to the presentation from Moore the day before, outlining the breakdown of a series of proposals he had on misplaced rank terms.

### Article 33 (continued)

[*Art. 33 Prop. N was discussed before K, L and M which were dealt with later in the day during discussion of the Moore package on misplaced ranks. It has been returned to the order in the Synopsis.*]

**Prop. K** (119: 20: 14: 2).

**McNeill** turned to the second core area of misplaced ranks, Art. 33 Prop. K. He pointed out that it needed to be an Article, not a note.

**Moore** had no objections to the change but noted that there was some question as how to deal with it editorially if it had a binding effect. He explained that the Note gave some detailed guidance on how to deal with misplaced ranks as the existing Article had a lot of different interpretations. He added that it might be a meaning change.

**Prop. K** was **accepted.**

**Prop. L** (110: 28: 13: 3).

**McNeill** moved to the third proposal on misplaced ranks, Art. 33 Prop. L, which he felt was slightly different as it dealt with sequential usage of the same rank-denoting term. He was of the opinion that it was indeed a Note and not an Article and clarified that a Note was something which did not introduce any new concept into the *Code*, but clarified something which might not be immediately obvious.

**Kolterman** had a question relating to the clarification of the proposal that appeared in the next proposal with an Example. He thought it would mean that if an author published subspecies within subspecies that all of them would be treated as validly published at the same rank of subspecies even though the original author did not recognize [them at the same rank].

**Moore** guessed that was sort of a semantic dispute whether or not they were considered at the same rank or not. He felt it could be taken that they were at the same rank, as a hierarchy had just been inserted, either by indentation and use of roman numerals, etc. and letters within that hierarchy. He noted that there were examples of this that had been used. He was curious to see how other people had treated the issue, because he thought it had been inconsistently treated. His view was that this was the more stable way. He added that there were examples where it may involve apomictic species with one large species and then within that people described other species within the species. He suggested that if the Section went the other way and wanted to treat it as a misplaced rank situation where these treatments existed, then he thought you would have to throw everything out, because, it did not make any sense to declare one of those ranks invalid. He felt you had to take them both as it made no sense to declare the first species valid and the second one not since he did not think it was any more logical down a sequence than it was up a sequence. He thought that the source was the Gandoger species problem, although maybe not in any formal discussions. He explained that the work was initially accepted but then later suppressed at the rank of species.

**Prop. L** was **accepted.**

**Prop. M** (107: 27: 17: 2) was referred to the **Editorial Committee.**

**Prop. N** (113: 23: 15: 2).

**Moore** introduced Prop. N, saying that it would introduce a new concept in the *Code*, in this case, an Article. He elaborated that if a rank-denoting term was used at more than one hierarchical position, i.e., it was not successive, it would be considered informal usage and they would not be ranked names. He referred to an example in Bentham and Hooker which explained this situation. He added that it was not all that uncommon in early literature with a number of terms we now considered to be formal rank denoting terms such as division, section, series... He thought it would reflect what was the case in these earlier publications. He argued that it would wipe out a number of cases where otherwise there were misplaced rank-denoting term problems.

**McNeill** noted that the proposal received strong support from the mail ballot.

**Redhead** did not see a time limitation on the proposal to restrict it just to earlier literature. He thought that if it was done today it would not be acceptable, so the discussion was about the older literature.

**McNeill** thought, in fact, that the proposal was to treat them as not validly published.

**Moore** agreed they would not be validly published because if they were in the earlier literature they could be validly published but unranked as the unranked Article would kick in at that point. He noted that there was a time limit on that particular Article, which was cross referenced in the proposal. He concluded that if that were done today it would not be validly published, ranked or unranked.

**Redhead** apologized, claiming it was too early in the morning and he was looking at N instead of M.

**Moore** confirmed that it was N under discussion but perhaps not up on the board, which may have been the problem. He pointed out that it said “see Art. 35.1” which had the date limit of 1953. He added that if it was done in early literature before 1953, they were unranked names.

**Wieringa** found Prop. M unclear. He thought that if you were talking about large publication where 50–60 species were described and only in one place subspecies had been described under a variety instead of subvariety, so in that case subspecies was found in two levels, below and above variety, then all names at the right level might be lost.

**Moore** felt that there was limit to how far it was possible to accommodate difficult situations like this. He pointed out that in the case of Bentham & Hooker, they had used “series” at 11 different hierarchical positions but there were a couple of cases in Bentham and Hooker where they had used it properly. He suggested it was possible to say that one was right and all the rest were wrong. The alternative he offered was to say none were anything but informal ranks. He preferred to look at the whole work and treat them all as informal ranks. He acknowledged that there may be cases, as just presented, where there was one mistake, subspecies misused below variety. He wondered how far the Section wanted to parse it to save some of these difficult situations?

**McNeill** wondered if Wieringa had an actual situation where this had happened?

**Wieringa** did not, it was hypothetical.

**P. Hoffmann** asked if unranked was a term defined in the *Code*, questioning what exactly unranked meant and what its consequences were for priority?

**Moore** suggested that the Editorial Committee could adjust it to make it more consistent with Art. 35.1, which just said that a new name or combination published after 1953 without a clear indication of the rank was not validly published. He felt it could be reworded to make it clearer. He felt that using “series” at several different positions, like Bentham and Hooker did, really was not clear.

**Redhead** pointed out that unranked was used by Fries in his *Systema* with tribes out of order and not in correct rank so taxa were treated as unranked.

**Moore** thought that was an exception to the main rule of Art. 33.7 as they did not use the term they were treated as validly published as subdivisions of genera but also unranked within the infrageneric rank.

**McNeill** felt that Moore was probably correct and it would parallel the existing Articles. He thought the meaning was clear and assured the Section that the Editorial Committee would make sure it was quite unambiguous.

**Redhead** noted that, although it said “see Art. 35.l”, it did not actually declare the names to be invalid. He pointed out that Art. 35.1 said names published without a clear indication of rank were not validly published. He continued that this situation was a series of [names] with rank-denoting terms, being treated as unranked, even though it was cross-referenced, but it did not actually declare them invalid.

**McNeill** felt that the point had already been raised, making it clear that if rank was unclear, you should refer to Art. 35.1. He stated that if accepted, it would editorially be made a clear parallel to Art. 35.4 and, if accepted, 35.3.

**Prop. N** was **accepted.**

**Prop. O** (112: 22: 17: 2) was referred to the **Editorial Committee.**

[*Short discussion of Art. 16 Prop. E, a corollary to the acceptance of Art. 33 Prop. N, occurred here and has been moved to the Second Session on Tuesday afternoon following the sequence in the* Code. *Art. 35 was discussed before Art. 34 but has been moved to the* Code *sequence. Discussion of Art. 18 Prop. G and H also occurred here and has been moved to the Second Session on Tuesday afternoon following the sequence of the* Code. *A vote on Art. 19 Prop. D was taken here with no discussion*.]

### Article 34

**Prop. A** (105: 40: 8: 0).

**McNeill** moved on to Art. 34, noting that the first proposal was a reference which the Rapporteurs suggest be referred to the Editorial Committee. The Rapporteurs felt that both Props A. and B improved the current wording and could therefore be referred to the Editorial Committee but he added that there were strong votes in favour of both.

**Nic Lughadha** thought Prop. A was a substantive change to the *Code*. She could think of examples that had been treated as validly published which would be invalidated. She felt it was a change from looking at internal evidence in the original publication to looking at external evidence at the time of publication, if “upon” was interpreted as meaning “at the time of”. She did not think there was another interpretation. She gave an example: A colleague had a new species, about which he was very excited, had an expensive watercolour plate prepared for publication in *Curtis’s Bot. Mag*. And then it went to press and [during] lead time he subsequently realized that he had made an embarrassing mistake and retracted it in another publication with a shorter lead time. He could not withdraw from the *Curtis’s Bot. Mag*. So, at the time that the *Curtis’s Bot. Mag*. new species appeared, everybody already knew that he did not accept it. But the internal evidence in *Curtis’s Bot. Mag*. was what should be judged and it was validly published. She thought it would be an unfortunate change. It raised a more general concern for her that, when going though and making hundreds of what the Section thought were minor tidying up changes, she thought it was almost inevitable that one or two important substantive things would be missed. She and her colleagues had completely missed this the first time around, as she guessed the Rapporteurs did too, as did most of the people who voted. Therefore she expressed concern at the number of small, tidying-up changes being made. She worried that not all of them would prove to be have been tidying up at the end of the day.

**McNeill** had just looked at his notes and realized that Nic Lughadha was absolutely correct. One of the reasons that he suggested this not be approved but referred to the Editorial Committee was that he was not certain that there was not a change in the meaning. He felt that Nic Lughadha had made it very clear that there was a change and he advised that the Section reject it.

**Alford** also suggested that the Section reject it. He highlighted that the Rapporteur and Vice-Rapporteur were familiar with the case of *Opera Varia* where Linnaeus’s works previous to 1753 were published as a pirated document after 1753. To him it was quite clear as it stood that those name were not valid because in the original publication Linnaeus agreed but then, of course, in the pirated publication there was no evidence.

**McNeill** appreciated Alford’s argument on the subject of rejecting the proposal, although he did not quite buy the *Opera Varia* argument, but felt that that was another matter. He suggested that Alford was interpreting “original publication” to be “original publication pre-starting point” but he did not think that was widely accepted.

**Prop. A** was **rejected.**

**Prop. B** (131: 16: 7: 0) was **accepted.**

**Prop. C** (24: 21: 109: 0).

**McNeill** suggested that Art. 34 Prop. C was editorial and could go to Editorial Committee.

**Zijlstra** felt that it was a special case and she really thought it should be a voted Example.

**McNeill** wondered if Zijlstra would explain why she did not think “ad int.” exemplified the Example?

**Zijlstra** had been searching to find, in the *Code*, what was the meaning of a “voted Example” and could not find it indexed.

**Unknown Speaker** pointed out it could be found in the preface.

For **Zijlstra**, “voted Example” had a stronger meaning than simply “Example”, which could easily be removed again.

**McNeill** responded regarding what a voted Example was. He noted it was pointed out as a footnote to Art. 8: “Here and elsewhere in the *Code* a prefixed asterisk denotes a voted Example accepted by a Congress in order to legislate nomenclatural practice when the corresponding Article of the *Code* is open to divergent interpretation or does not adequately cover the matter.” He felt that the question was really, did the expression “ad. int.”, which was the core of the Example, not exemplify [Art. 34.1 paragraph] “b” when it was merely proposed in anticipation of the future acceptance of the group concerned or for a particular circumscription, position or rank of the group concerned, the so-called provisional name.

**Landrum** reported that he and his neighbours did not know what “ad int.” meant. He felt it should be put in translated as well.

**McNeill** apologized and explained that it stood for “ad interim” in Latin, “for the meanwhile”, “for the moment”. He thought it was conceivable that people might feel that this was not really exemplifying and then it had to be a voted Example. He just wanted to be clear if it was the mind of the Section that this did not exemplify the clause, which he thought it did.

**Demoulin** thought that the fact that some of the people did not know the abbreviation of “ad interim” showed that it was interesting to put what “ad int.” meant in so that everybody knew. He argued that if the Section considered the proposal to be a voted Example then everything would have to be a voted Examples because he could not image something that was not as straight forward an illustration of Art. 34.1 paragraph “b” than this. He felt that it was a real direct consequence of the rule. He concluded that it was a good Example for the Editorial Committee and for those who know “ad int.” but it had nothing to do with a voted Example.

**Turland** commented that if it was “ad int.” “ad interim”, “in the interim”, “for the time being”, presumably it meant that the name was accepted at the time of publication. He thought that the fact that it may not be accepted sometime in the future, would surely not invalidate it.

**McNeill** thought some classical scholars might want to say who used it.

**Schafer** thought it was rather clear because it was a last century example. But he pointed out that it was big problem with the polite writing of botanists in different countries of the 19th century. He noted that in some countries it just was considered polite putting a phrase “if everybody will accept this I propose this name.” He added that, of course the author wanted his name to be accepted, but he considered it impolite to say that “I accept it.” He was quite worried about the general tenor because previously in practice the unexpressed intention had been accepted. He argued that this proposal would just interpret former botanists literally by what they said.

**McNeill** thought that was a very important point that was, to a large extent, covered by “does not apply to names published with a question mark or other indication of taxonomic doubt yet accepted by their author”. He agreed that there were many cases, prior to the 20th century, where people did couch their presentation in the polite terms that had been described (the subjunctive),. On the other hand, he felt they clearly accepted them, by typography and everything else. He did not think these things were covered by the Article, but there were situations, as in the existing Example, which indicated what the intent was. He suggested that more Examples may be needed to deal with Schafer’s point.

**Gandhi** wanted to mention that the proposed Example illustrated a situation that was different from the present Ex. 3 in the *Code* which talked about provisional names for the future, whereas the Example under discussion was about accepted now or maybe for the future. In his opinion it was acceptable. And he pointed out, as he felt everyone knew, no name was permanent giving the proof that of nearly 1.5 million names indexed for IPNI, nearly 1.1 or even more, were synonyms. He concluded that no name was used by everyone.

**Nee** felt the particular Example was exactly parallel to Ex. 4 [Art. 34.1] of provisional names. Provisional names were accepted by the author at the time, but just provisionally, so he argued that that took care of the comment that “ad int.” would be accepted at the same time. He thought it was just a parallel Example to Ex. 4 that would simply make another nice Example to be published in the *Code*.

**Nicolson** wondered if the plan was to vote to refer it to the Editorial Committee?

**McNeill** clarified that in the case where the Section wanted the Example in the *Code* but where it was not a voted Example that would be referred to Editorial Committee. He added that a voted Example must be voted “yes” but it was quite clear that this was not a voted Example.

**Prop. C** was referred to the **Editorial Committee.**

### Article 35

[*Art. 35 was discussed earlier in the day as part of the Moore package on misplaced ranks. It has been placed in the order of the* Code.]

**Prop. A** (124: 18: 11: 2).

**McNeill** introduced Art. 35 Prop. A as making an addition to Art. 35.2.

**Moore** had received one comment that morning and felt that if the proposal was making a substantive change it should be an Article.

**McNeill** pointed out that Art. 35 Prop. A was an Article.

**Moore** apologized and explained he was getting ahead of himself. He felt that the proposal was logically consistent with what the Section had just been dealing with and it tried to clean up some of the language dealing with endings denoting rank in more than one place in the taxonomic sequence.

**Wieringa** thought that if this proposal were accepted and Art. 33 prop. L was also accepted then there would be a [conflict] situation.

**Moore** thought that that was probably a good thing to discuss. If that rank was already used in the classification, either in a successive or non-successive position.

**McNeill** suggested that if Art. 33 Prop. L was passed the Editorial Committee be instructed to make an alteration here. [That was done.]

**Prop. A** was **accepted.**

[*Here the record reverts to the actual sequence of events*.]

### Article 36

**Prop. A** (12: 147: 0: 0) and **B** (5: 151: 1: 0) were ruled as **rejected.**

### Recommendation 36A

**Prop. A** (11: 125: 21: 0) was ruled as **rejected.**

### Article 37

**Prop. A** (1: 150: 2: 0) and **B** (1: 151: 1: 0) were ruled as **rejected.**

**Prop. C** (23: 96: 32: 2).

**McNeill** introduced Art. 37 Prop. C as a proposal from Brummitt and others where he expected some discussion.

**Brummitt** suggested that the topic was something that the Section could get their teeth into and one that had a direct impact on a lot of those present. He thought the Section members may have noticed that there was a row of people from the same institution and, with the President’s permission, when he had had his little say on one aspect of the proposal he was going to pass the baton down the line, and four of them would like to express their views on different aspects of the business. He assured everyone that he was not going to war with the Editorial Committee and that they were all good friends and would continue to be good friends, but pointed out that even among friends there were occasions when there were genuine differences of opinion. He did not want to go back and have arguments over what had happened in the past. He thought it was fair to say that he had argued about the issue for at least 35 years and not resolved the problem. In recent years he knew that Rapporteur McNeill knew absolutely that his [Brummitt’s] views were wrong. On the other hand Brummitt knew absolutely that McNeill’s views were wrong on the issue. So he felt there was no point arguing and no need to go back over past issues. The position they wished to make was firstly that the Editorial Committee did not have the mandate to make the change in the *Code*. Secondly, that it was nonsensical and impossible to put into practice. Thirdly, they would like to see, Art. 37.4 removed now and because different people did have different genuine feelings that illustrations should be allowed as types. If Art. 37.4 could simply be got rid of, in the first place, then it was on to the floor, he thought he had the agreement of the Rapporteur on this, to make proposals for what should happen in the future. Briefly, when the type method was introduced into the *Code* in 1935, there was a sentence saying that you could use an illustration. It did not say that it was only...

**McNeill** interrupted to say delicately, “Brummitt, I wonder”. He thought Brummitt had said that this was what he was not going to get into…

**Brummitt** felt that the Section just needed to have some background. He proposed, with a colleague, at the last Congress, that the sentence was simply meaningless. It was his opinion, but not the opinion of the Editorial Committee members who were present. So he proposed that it be deleted and that failed. He added that there were lots of reasons why a proposal may fail among the people who were discussing this at St. Louis. He thought that the negative vote on his proposal at St. Louis [to delete Art. 8.3 of the *Tokyo Code* apparently limiting an illustration as type] was essentially a vote for no change. However, the Editorial Committee had taken the view that that gave them the right to interpret it in a completely different way which retroactively. devalidated names published from 1958 onwards which were based on illustrations. The *Code* [Art. 8.1] throughout that period had had a definition of a holotype reading “a holotype is a specimen or illustration” with no reference to anything else. He thought that. the Editorial Committee had interpreted this [the rejection of the proposal to delete all of Art. 8.3] as an invitation to have an illustration as a type only if necessary. He concluded that what had now been written into the *Code* was contrary to a widespread interpretation of the *Code* over the last nearly 50 years or so. There were. situations where an illustration was preferable and colleagues would make this point. The interpretation of the negative vote at St. Louis by the Editorial Committee, was never discussed at St. Louis. He and others were absolutely aghast that the Editorial Committee could have made such a change to the *Code* which invalidated many names, particularly in the algae. In the discussion at St. Louis, it was pointed out that in algal literature illustrations were very often used. He summarized that what they would like to see was going back to square one by deleting Art. 37.4. He continued that perhaps he should have made it clearer to those who were not familiar with the details that this was originally in Art. 8.3 and the Editorial Committee moved it to Art. 37.4. He clarified that what they were proposing was deleting something which was originally a completely innocuous sentence in Art. 8.3 which had been moved to Art. 37.4. If that could be removed then he suggested that the Section needed to think about what should happen in the future. Some people would get rid of illustrations completely. Others would say “in some circumstances illustrations should be used as types”. He passed discussion to his left.

**Nicolson** instructed the following speaker to speak directly and briefly like Brummitt.

**Nic Lughadha** endeavoured to be even briefer. She wanted to address the point of the difficulty of interpretation and application of Art. 37.4 as it currently stood. The difficulty was determining when it was impossible to preserve a specimen. She wondered who judged? She reported that they found it was impossible to decide when it was impossible to preserve a specimen. She added that sometimes it was impossible to preserve a specimen of a particularly spiny cactus, if she did not have the appropriate equipment. Whereas, she gave the example that her colleague on her left, Nigel Taylor, would probably collect it with his lips if his hands were otherwise occupied, if necessary. Her point was that it was question of motivation, in some cases. Sometimes she did not have permits and therefore it was impossible to collect a specimen. She wondered whether she needed to document, in her publication of the species, that it was impossible for her obtain a permit or was it impossible because she simply did not wait for the necessary reviews in order to obtain the permits. She continued with the example that a wild animal was chasing her across the field so it was impossible for her to collect a specimen. She concluded that they found the Article impossible to interpret and apply reasonably. Her colleagues would cite some specific examples but she thought that the principle was clear that it was impossible to interpret and apply reasonably.

**Nigel Taylor** wished to briefly echo with a couple of examples what some of his colleagues had said. He reported that they had many of the algae and one of their colleagues from Australia, Roberta Cowan, had provided them with a list of algal names published over two periods, recent and some back in the 80s and early 90s.

**McNeill** interrupted on a matter of fact: the Article only related to a period after 1953, so it was the recent ones.

**Nigel Taylor** confirmed that that was what he was talking about. He acknowledged that clearly illustrations had also had large importance in certain groups of spermatophytes, Nic Lughadha had mentioned cacti, but other groups of succulent plants which were particularly difficult to preserve, not impossible perhaps but particularly difficult. In many cases, if the holotype was an illustration one would be able to interpret the author’s intention much better than from a preserved specimen. He had an example from a colleague, Mike Gilbert, who some years ago, was collecting in Ethiopia. He came across, by accident, two tuberous-rooted species of succulent plants where the annual growths were very ephemeral. He collected them while collecting something else. He took them back to his garden. He grew them on. He flowered them. He photographed them. He described them. He put the material into spirit with a view to publishing these as new species. Unfortunately he subsequently lost the material. But he had the photographs. He would like to write them up for the flora of Ethiopia and Eritrea. But he had a dilemma. Could he use the photographs as holotypes? If he could not then he was not able to describe the new taxa. It may be very difficult to for him to go back and collect them. If he does not happen to be there at the right time of the year his chances of finding the plant were quite small and it would be a pity if science was denied the new taxa. It was not clear that it was impossible but it would be very difficult for him. He may never have a chance. He found it strange that the *Code* allowed illustrations as neotypes but, apparently, only under the very exceptional circumstances. since 1958, were holotypes allowed as illustrations. This seemed inconsistent to him. In the future, he thought the Section should look at what the needs of taxonomists were when designating types for certain groups of plants. He concluded that for the *Code* to rule out, in this manner, illustrations as types was very unfortunate.

**Atha** thought that because somebody did not have a permit and therefore was illegally collecting a plant, was no excuse for using an illustration over a specimen as the holotype. Or if they forgot to bring their gloves or did not have a shovel. He thought that if algae were a special group and the algal group wanted to have illustrations as holotypes then perhaps the *Code* should be amended to except algae.

**McNeill** totally agreed with Brummitt that they would never agree totally on the history of Art. 37 Prop. A. and he was very glad time was not being spent looking back on that. He thought it was far more important to look forward. That being said, he added that the Editorial Committee was not totally cavalier in this. There was a reason and that was that the Rapporteur explained the implications of the deletion of part of the relevant Article at St. Louis and the retention of the other. And that interpretation was not challenged on the floor and it was that interpretation that was implemented by the Editorial Committee. Whether they were right or wrong, fortunately did not have to be pursued at this time. The Section had to address the forward looking picture. He also quite agreed, as he was sure many others would, with what Nic Lughadha had to say about the difficulty of interpreting the phrase “if it was impossible to preserve a specimen” which he felt brought up something that the Section may want to address. However the core issue, he thought, was that which Nigel Taylor brought up whether the Section wanted illustrations as types from 1 Jan 1958 or not. The situation was ambiguous until St. Louis. It was now perfectly clear that for names published prior to 1 Jan 1958 the type could be a specimen or an illustration. There was always some doubt in the wording before as to whether you could have an illustration if there was a specimen. He thought that that had now been completely cleared up to everyone’s satisfaction. He suggested that now the Section was looking at the situation post 1 Jan 1958 when the designation of a type became obligatory. He explained that the issue that Nigel Taylor had raised and the issue that was enshrined in Art. 37.4 was that at the moment you could not have an illustration as type unless it was impossible to preserve a specimen, whatever that meant. It seemed to him that the question that should first be addressed was whether putting a restriction on types after 1 Jan 1958 was desirable. If the Section wanted no restriction, as Nigel Taylor had expressed, then the Article could be deleted and there was no need to address the problem of difficult wording of “impossible to preserve”. But, he continued, if the Section did want to keep a ban on illustrations as types after 1 Jan 1958, then the proposal should be rejected but we might very well want to come back then and address the quite cogent point that Nic Lughadha raised as to circumstances in which we might allow an illustration, the equivalent of “impossible to preserve”. He thought that the first discussion should concentrate on the desirability of having illustrations as types.

**Redhead** reported that, with regard to fungi, the Article had created problems because it had basically invalidated several groups of fungi. He was thinking particularly of chytrids but there were other groups of microfungi which you could not necessarily even preserve in a lyophilized state, if you were thinking of going the cultural route. He felt that if you looked really carefully, you could find groups, genera, species of things like chytrids that were invalid because of this Article. He felt that that even post-1958 it was desirable to allow illustrations as types.

**McNeill** thought his final comment was perfectly valid, but did not understand his first. He thought Redhead said these were chytrids and other groups in which they could not be lyophilized.

**Redhead** agreed you could not.

**McNeill** replied that then those names would not be made invalid.

**Redhead** felt that one could always argue that you could make a smear and have a very poor specimen. There would be generic material there, perhaps, but, from a point of view of what most think of as a specimen, he argued that it was basically useless.

**Nigel Taylor** just wanted the Section to be aware that the supposed clarification, introduced into the *Code* at St. Louis, had retroactively made a number of names invalid that were previously accepted. They had done a study and there were a considerable number of names affected.

**Demoulin** wished to make some link between what Redhead said about fungi and what Brummitt said for the algae because Pierre Compere who discussed the matter in St. Louis was not present. He thought the Section should understand that it was a general problem for microscopic organisms, whether they were algae or fungi as they were impossible to preserve. He pointed out that this always caused big problems because people who considered something was impossible another would say, “you can always can use a good fixative, a good embedding medium, and you’ve got something on an electronic microscope, stuck somewhere that was a specimen”. He added that nobody would have a look at that, of course, everyone would look at the photograph that had been published. Formerly, people said you may preserve something and, because of this situation, there were a large number of names in those microscopic algae and fungi that sometime somebody may decide they did not consider them valid. He thought that the lower plant people who worked on microscopic plants would be on the side of Kew.

**Freire-Fierro** wondered what were the specifications of an illustration? How did people know if the illustration would be enough if it was considered a type?

**McNeill** replied that there were none at the moment in the *Code*.

**Dorr** was following up on Freire-Fierro’s comment.. An article? that he read that was originally submitted to *Taxon* for review spoke about illustrations and in a number of examples the illustrations were photographs of orchids. He thought that up to this point in *Code* when illustrations had been spoken about people had not been thinking of photographs. They were thinking of black and white diagnostic fine art which could be very helpful in interpreting a species. He argued that it became a completely different matter to present photographs or other continuous tone images and felt that it would be very difficult, in the future, to interpret some of the taxa. He was also not opposed to permitting illustrations in situations where it was very difficult, for technical reasons, to preserve material. He thought, in the future, it was going to much more important and it was going to be absolutely impossible to sequence a photograph.

**Nicolson** remembered Dick Korf making that point [at Berlin].

**Prance** strongly supported deletion of this from the *Code*. He thought that it would invalidate a lot of accepted species, as Nigel Taylor said, for example some of the *Bromeliaceae* described by Lyman Smith from Margaret Mee illustrations. He gave a specific example, where he had described a new species from a unicate specimen loaned to New York from Manaus. The box in which he returned it was destroyed in a dock dispute and thrown into the Amazon. This meant that the only thing to typify that species was the illustration, fortunately a good, detailed black and white illustration. He thought that there were many examples which would support allowing illustrations as types when that was appropriate.

**Schanzer** wondered if it was possible for the Editorial Committee or some other Committee to produce an explicit list of higher taxa where illustrations were not possible as types. He suggested angiosperms or gymnosperms. He felt it was inappropriate to mix up groups with microscopic organisms, like microscopic algae or fungi with angiosperms. He thought it was not desirable that an illustration could be a type in angiosperms because it could not be studied further. In microscopic groups the situation was quite different. He felt that maybe it was desirable to separate them explicitly.

**Per Magnus Jorgensen** thought that it would make life easier if it went away but was afraid that it could be misinterpreted so that people started photographing organisms and describing them on the photograph. He wondered if there was some way to prevent that. He supported the deletion.

**McNeill** clarified that there was not current wording to that effect and suggested Jorgensen might ask Prance when he said “when it was appropriate”. He added that if the Section deleted the Article, it would always be appropriate.

**Zijlstra** would only talk of cases for which it was possible to preserve a specimen. For several years she had done editorial work and was struck by how often the type was an illustration, usually not a photograph but a very detailed illustration and it would be disastrous if the Section should say it was no longer possible. She was concerned with cases after 1958.

**L. Hoffmann** also supported deletion of the Article, at least for micro-organisms because, for algae, it was absolutely essential to have the possibility to have illustrations as type. Many of the microalgae, which were unicellular, were very delicate and impossible to preserve and even when it was possible to preserve, many characters and features were lost though preservation. Furthermore, since 1980, he pointed out that if you looked at the literature, many algae were described simply from a figure as a holotype and many would be invalidated. He added that, for many of them, you could show that it would have been possible to have preserved a specimen.

**McNeill** felt that the latter point was extremely valuable but it should be borne in mind that, in order to be validly published, the name of new taxon of a non-fossil algae from 1 Jan 1958 must be accompanied by an illustration. He elaborated that the type must be a specimen, but there must also be an illustration for valid publication which dealt with part of the point.

**Gandhi** supported the deletion of the Article because it appeared to be symbolic. He had come across situations where authors always circumvented the mandatory citation of a specimen. Sometime in the 1990s he indexed an arctic name solely based on an illustration made in 1860. The author who published the name claimed that. no-one could collect any specimen in that cited locality. So, solely based an illustration, a new species name was published. No-one can claim the authenticity of the particular species, whereas it really existed. Everything, like Latin diagnosis, was mentioned and illustration solely as a criterion. He felt that people could always find some way to deviate from the Article. He wished to mention, even for names pre-1915 more weight was given to a specimen rather than to an illustration. Philip Miller, whose binomials were validated in 1768 in his Dictionary, referred to a binomial and gave more weight to a specimen rather than to an illustration, so the binomial was validated in 1768. Later on Aiton, in his Hortus Kewensis, used a different name referring to a figure which was used by Miller and we say that Aiton’s name was not illegitimate because he used the figure but not the specimen. So, in other words he used the specimen but not the illustration.

**Marhold** wondered about deleting the Article and putting some Recommendation in which would strongly recommend preserving a specimen. He suggested it could be referred to the Editorial Committee. He felt that if there was a strong Recommendation in the *Code*, he may be able to try to force an author to put a specimen as a type, if it was at all possible because he obviously liked a specimen much more than an illustration.

**Redhead** noted that there had been a discussion about the use of photographs and there seemed to be an inclination against that. He recommend that the Section not exclude photographs, at least for the microfungi, because he knew that there were certain groups where a photograph, rather than a line drawing, had been used as types for various groups and again he reflected on the chytrids. He did not want see photographs excluded and thought that amongst the algae too, that photographs of diatoms and what-have-you, might be used as types. He was in favour of removing Art. 37.4.

**Pedley**, after an indecipherable anecdote broken by audio gaps, thought that a photograph was O.K. and an illustration was O.K.. A few years ago he was at the BM, looking at some desmodiums, one of which was described by Burmann for the *Flora Indica*. There was an illustration and in the folder there was a note from William Stearn to van Steenis saying that obviously this had to be lectotypified on the illustration, but the illustration was not worth anything. He suggested that, unless it was impossible to preserve a specimen, that there should be a specimen, not an illustration.

**Buck** was very sympathetic to the microscopic algal and fungal groups. He thought that those people should make a proposal to exclude the groups. Basically he felt that we should not throw out the baby with the bathwater. For the vascular plants he was not at all sympathetic to the people from Kew who felt that they were in a preserve with no collecting permit, were running through the field, chased by wild animals, and then got home, thought they saw a new species and could sketch it from memory and expect us then to believe that. He would much rather lose a bunch of names than have a sketch of a specimen which might be fine if it was really a distinctive thing. He argued that many things turned out to be complexes and that no illustration was going to be able to let you distinguish those from others with techniques like leaf anatomy or any number of things. He really thought it was an important thing to leave in the *Code*. If there were problems with microscopic organisms those people needed to make a proposal to make an exception.

**Nic Lughadha** wanted to be really clear, that most of the cases that they were talking about, would not, of course, involve Kew botanists who would never ever be in a reserve without a collecting permit. They were looking at thousands of cases each year because of IPNI and therefore had come across difficult decisions where an illustration had been indicated as the type and they were in a position where they were having to decide whether the illustration was cited simply because it was impossible for some reason or another. It was not meant to be a personal expression of what Kew botanists did or did not do in the field.

**Gereau** pointed out that there already was Art. 9.7 allowing for the designation of an illustration as an epitype and Art. 9.6 allowing for the designation of an illustration as a neotype. If a holotype was inadequate for critical identification, he suggested the researcher designate an epitype. He highlighted that illustrations could not be sequenced, rotated, they could not be otherwise manipulated in many ways that even inadequate specimens could. If Art. 37.4 was flawed in some way it need to be fixed, not removed. He felt that removal was an invitation to irresponsibility.

**McNeill** wished to pick up on the last point. He noted there obviously could be no promises as to what the Section did or did not do and he was not suggesting that he had good wording, but he thought that the issue was clearly of great concern to people who worked with unicellular microorganisms. He thought it was something the Section should seriously address. He suggested something like “if it was technically difficult or impossible to preserve a specimen”, with the caveat that it might be too big a floodgate. As far as he could see it would cover all those situations and therefore ensure that for whole groups of organisms, the names would not become invalid. He thought it was something the Section could certainly look at.

**Gams** refrained from repeating the arguments for the desirability of illustrations for unicellular fungi as he felt that they had been efficiently presented. He pointed out that Art. 37.3 referred to Art. 37.4 which was being debated and that would require some adaptation as there it was stated “when permitted by Art. 37.4”.

**McNeill** felt that there really was no need for that to be emphasized, if and when Art. 37.4 was deleted, the corresponding references would go as well.

**Wieringa** did not really want to vote for deleting the Article if he did not know what it was going to be replaced by, maybe later on. He suggested that it was better to postpone a vote on the Article until there were alternatives and the Section had been told about those alternatives. So instead of deleting it maybe there should be another proposal to replace it by a better text.

The route **McNeill** suggested, though the Section might want to go differently, was to take a vote on it as it stood. He felt that if it was not deleted then the issue should seriously be addressed, particularly, micro-organisms but possibly also other situations.

**Demoulin** felt that everybody agreed that a good original description should include a full description, preferably in Latin, English and even a third language, a good preserved specimen with several duplicates, some material that had been dried in a way that you could extract DNA from it, a good illustration, an interpretive drawing, photographs with an electron microscope, and so on. That was ideal. But, he wanted to remind the Section of the paper earlier in the year in *Science* with a picture, apparently it was the paper that got the most visits on the website of the American Association for the Advancement of Science and was based on a video of a large woodpecker that was supposed to have disappeared from eastern United States and had been found again recently. This worried all the molecular biologists who published in Science they been reading a lot and seeing a lot just based on a video. So when something in natural history was really important to record, I think we may accept a video.

**Smith** strongly supported the proposal to delete. He found himself in complete agreement with colleagues at Kew. He reported that they dealt with thousands of identifications per annum and it was often much easier to work with a good illustration rather than a very bad specimen. He felt that everyone was familiar with the fact that when working with succulent plants the illustration was far more diagnostic, especially when you had to identify thousands of them.

**Nicolson** thought that the discussion was getting to saturation. He suggested three more speakers and then thought a vote was needed.

**Peter Jorgensen** did not think it dealt with identification but with valid publication.

**Per Magnus Jorgensen** wondered if he might make a friendly addition to the deletion? [Laughter.] He wished to add a Recommendation recording the preferability of specimens. He suggested this could be added, at some point, by the Editorial Committee. He felt that was a way of telling that specimens were preferable as everyone agreed on that, even the microscopic people. None of his specimens were macroscopic but they all were in herbaria, so he had never come across the issue. But he acknowledged that there were organisms that were difficult and it was not that easy for microscopic organisms and non-microscopic organisms. He reiterated that he wanted to have an addition to the proposal asking for a Recommendation.

**Proschold** noted that Art. 37.4 contradicted Art. 8.5 [8.1 or 9.1?] which allowed an illustration as a holotype while in this Article it was allowed only if it was impossible to preserve a type. He added that, especially for the algae, it was possible to preserve a specimen, that was not a problem for most of the algae, but it was not recognizable as an alga, only a green spot on some postcard maybe. He felt that a good illustration was needed and maybe, in addition, something preserved in liquid nitrogen was possible.

**McNeill** explained that there was no contradiction. Art. 8 was dealing with the general situation and Art. 37.4 dealt strictly with the period after 1 Jan 1958 in which designation of a type became obligatory. Secondly, at the present, if it was possible to preserve a specimen and not just technically difficult, it had to be preserved. But, for an algae to be validly published there also had to be an illustration again from 1 Jan 1958. In summary, it seemed to him that all those names were actually perfectly O.K. under the present *Code* so long as there was at least that green spot and that illustration. He added that, of course, for older names illustrations were perfectly acceptable as types. However, what he felt was being expressed, at least in part, was that in many groups it would be beneficial if it was not declared to be impossible, because, as so many people had said, what was possible for one person was impossible for another. He agreed that it was a difficult term to define and the wording he suggested was “technically difficult” or “impracticable to preserve a specimen” as he felt that would probably deal with the micro-organism situation. He thought the Section should come to that later, unless somebody wanted to propose it, after getting a feeling for whether people were quite happy to have the freedom to have an illustration as a type for all organisms at all times. He pointed out that Recommendations, although they were nice and pleasant, had no binding force. He clarified that if the proposal was accepted and the Article deleted, the Section was simply saying that they accepted that illustrations were just as acceptable as specimens for types of names currently. If, on the other hand, the general feeling was to keep it, then he thought it very important to discuss the matter further and to look at the special situation of micro-organisms that had been drawn to our attention.

**Nic Lughadha** believed there was a friendly amendment on the table. She thought that the Rapporteur had once again summed up, perhaps not exactly as she would have, and adding a Recommendation, though it may not have the force of law, did make a significant difference, as Marhold had pointed out, to editors, who would then be a in a position to urge authors to choose a specimen, if it was at all possible. She felt it was not a question of making an equivalent specimen.

**Gandhi** wondered how indexers could question an author’s statement that it was impossible to preserve a specimen? When they indexed a name they were determining whether the name was valid and/or legitimate based on the *Code* Articles. But if a statement was made, by an author, in the publication, how could they judge? He argued that they had to go by what the author stated, that it was impossible and they had to accept that it was impossible. Beyond that, as an indexer, he did not think they could question the author’s statement.

**McNeill** had seen the Recommendation pertaining to Art. 8 and had some concerns about it. He felt it would imply that, in choosing a type, say for a Linnaean name, you should go for a specimen in preference to an illustration. He did not have the exact wording and wondered if it was talking only about holotypes or about all types in Art. 8? He thought that would need to be clear before the Section could judge whether it was going to act effectively in discouraging. Of course it would only deal with, as someone said, editors as there was nothing to stop people from publishing privately their names with whatever fuzzy pictures or excellent illustrations that they had. He understood there was a serious problem with cacti, with many other groups, he was just a little concerned that we were considering that “type specimen” was no longer a phrase used in botany, just “type” because type specimens could easily become the exception.

**Per Magnus Jorgensen** responded that that part could be taken away. This was where the type was defined in the *Code*, in Art. 8. He had never thought of this and felt McNeill had a point.

**McNeill** added that, in other words, it was once a holotype had become mandatory, so he thought Jorgensen would like to have it linked to that.

**Jarvis** felt that, obviously, one of the consequences of now moving this back to Art. 8 did open up that situation described, say for Linnaean names, where 25% of the Linnaean names, as presently typified, were illustrations. In general he felt that everyone agreed that, when all things were equal, specimens were preferable as types, but, *de facto*, with a lot of these early names, they were based on illustrations, in many cases. He was not sure that the wording, especially as a Recommendation, necessarily conflicted with continuing to be able to use illustrations in that way. But he concluded that moving it back to Art. 8 obviously did have an impact on much earlier names in that way.

**McNeill** asked if that suggested it go back in Art. 37 or at least be in the context of the requirement for a holotype?

**Nicolson** wondered if there was an amendment or a proposal?

**McNeill** thought it was a friendly amendment.

**Nee** felt that Art. 37, as had been pointed out by the people from Kew, [could be interpreted as preservation being impossible] because you might may be trampled by a buffalo as you were collecting your specimen. However, deleting it, he thought, was one of the worst and most serious changes being made to the *Code* in several sessions, because there had always been a reliance on the actual specimens because of the obvious use of them for characters not seen before and even the best illustration may not bring those out. He had not seen any indication why it was not possible to preserve some of the material of even the most intractable small algae and so on for studying in the future with techniques we may not even have now, even though they were entirely inadequate for most purposes of identification at this time. Ideally what he suggested was that there should be an Article which said “type specimens”, an actual type specimen was what had to be preserved for a new species. Illustrations may be recommended, they may be mandatory and they were highly useful, but to simply say that specimens were preferable to illustrations put things on an equal footing and he thought that was very dangerous in the future. Even for such things as cacti, he argued that you could have a piece sitting there, with the spines and everything, that was not impossible and that was going to be useful, no matter how wonderful the illustration was. He felt that now the illustration may be what everybody used in the future for the identification, for their concept, but you still wanted that physical thing to refer to because it would be there forever and it may have characters that you could not see beforehand.

**Watson** just wanted to make a small comment on the problem of the lack of any type definition of what an illustration was in the *Code*. He thought most people were thinking of an illustration being something that was printed when a name was described, but it could also refer to an original painting housed somewhere, an original piece of artwork. With the current increase in the ease of printing things he felt it could maybe even be extended to inkjet printouts housed in herbaria or colour slides housed somewhere. He argued that these were non-permanent and there may be a bit of a problem. He meant that the type definition of what an illustration was could not really just be pushed into the glossary, because it would have a major effect on how the rulings were made.

**McNeill** thought that the Section was probably ready to vote as to whether to delete the Article. He thought that a lot of genuine concerns had been raised, so that even if the proposal was rejected, which would leave the Article as it presently stood, he thought it was quite open, perhaps not immediately, to bring in additional proposals to protect names that might be seen to be threatened by continuation of the present wording. He summarized that if you wanted to have illustrations freely as types then, of course, you would vote for the proposal and if you felt that specimens should be retained as the norm, as in fact the requirement from 1958 onwards then you would vote against it. He added that this was bearing in mind that some adjustment was always possible for those cases, including cases that had been deemed to be retroactively invalidated, if a case could be made for moving the date forward. Again, that was not something the Section could look at, there would have to be a proposal. He concluded that at the moment there was simply a proposal on the table to delete the Article and have open opportunity for illustrations or specimens and with the added Recommendation.

**Zijlstra** would not like to move the date forward because she thought all definitions about what could be a good type should be under Art. 8. So she wished to have that Recommendation but felt it could only be under Art. 8.

**McNeill** replied that it was a Recommendation relating to holotypes, so it belonged in an appropriate place, and not in Art. 8, which dealt with quite a broader range of types.

**Nicolson** moved to a vote on the proposal to delete and judged that the nays had it.

**McNeill** did not think there was any doubt. [Apparently there was, as a **card vote** was called.]

**Nicolson** moved to a card vote, reminding the Section that it must be number 4.

**Prop. C** was **rejected** on a card vote (151: 330; 31.4% in favour).

[*The following discussion took place prior to the report on the card vote*]

**McNeill** wanted to move onto the next proposal, Art. 37 Prop. D which he thought was automatically rejected because of the defeat of Art. 8 Props. A. and B.

**Redhead** felt that that was moving too fast. He thought that various options had been given if Art. 37 Prop. C failed and put forward that several of the Section would like to see an option of that particular Article.

**McNeill** responded that, should the card vote reflect what the President saw in the hand vote, that the proposal failed, then he thought that it would be appropriate for the people who were concerned, as many were, about the status, for example of micro-organisms, to come up with the form of words that could be discussed at a later session and not to rush into it and bandy words around here but come up with something that was a little coherent. He assured Redhead that there would certainly be time made for that.

[*The following debate took place after debate on Art. 37 Props D, E, & F, and following the result of the card vote on Prop. C.*]

**McNeill** explained that meant that a number of people would be getting together to come up with some form of words that could make the Article more sensible in terms of the portion relating to “impossible to preserve” which clearly applied to micro-organisms and may well apply to other groups.

**Atha** wondered if the Editorial Committee would tinker with the wording of Art. 37.4 again and create the same sort of controversy at the next Congress where some people felt they overstepped their mandate.

**McNeill** clarified that at the moment, the Editorial Committee would clearly do absolutely nothing with Art. 37.4 because the proposal had been defeated. The Editorial Committee would only consider doing something when a proposal was passed. What the Section would be looking at now was perhaps some form of words that would clarify what was meant, to solve the problems that had been suggested in chytrids and in some other groups of micro-organisms of names becoming invalid that had previously been treated as validly published. He reiterated that, at this point, the Editorial Committee had no power to do anything although he certainly hoped that some change in wording would be possible.

**Nicolson** asked people who were directly interested and willing to serve on an ad hoc group, to just hold up their hands and asked Redhead to be in charge.

**Redhead** asked those interested in putting together an alternative Art. 37.4, to meet at the break in the afternoon and then decide where to discuss things.

[*Here the record reverts to the actual sequence of events; the record of the debate on the alternatives proposed by Redhead’s group follow the remaining discussion on Art. 37.*]

**Prop. D** (59: 42: 25: 17) was ruled as **rejected** because Art. 8 Props. A and B were rejected.

**Prop. E** (5: 146: 1: 0) was ruled as **rejected.**

**Prop. F** (68: 31: 51: 1).

**McNeill** moved on to Art. 37 Prop. F which dealt with an unusual situation, suggesting adding an Article to cover designation of a type in a monotypic generic situation.

**Nicolson** noted that the Rapporteur had sent him a message saying that the comments were wrong.

**McNeill** reported that he and the Vice-Rapporteur had discussed it and it was a very unusual situation.

**Turland** agreed it may be a somewhat unusual situation but the circumstances under which the proposal could solve a problem was when the name of new monotypic genus was being published. The question was whether it required two separate type statements. He clarified that this was after 1 Jan 1958, as before 1 Jan 1958 mention of one species name only for a new monotypic genus would be sufficient to typify the generic name and then the new species described in that monotypic new genus would have its own designation of the type for species name. After 1 Jan 1958 if you only had one type statement for the species name, the issue was whether that would also effectively typify the generic name because an explicit statement of typus that applied to both the genus and the species was needed. He suggested that one way of looking at it could be that if you were stating a type for the name of the species, that was automatically also the type of the generic name if it was monotypic. But, of course, if you had a new monotypic genus the single species did not have to be newly described, it could be an existing species. Indeed, you could have more than one species being moved into the “monotypic” [actually unispecific] genus, one of them as a synonym. So, it was a little more complicated than the Rapporteurs initially thought.

**McNeill** thought that the situation was that, in the general case that the authors had in mind, it was already covered by Art. 10, because if there really was only one species name then that was covered. He added that it was possible to have “monotypic” genera, as Turland had just said, in which there was more than one name (a synonym), even though there was only one [accepted] species [i.e. unispecific not monotypic as defined in the *Vienna Code*].

**Karen? Wilson** was wondering why this should be a separate Article rather than just a Note under Art. 37.3 which was dealing with a new genus or subdivision of a genus. It seemed to her to be just one particular case of such a taxon and could it be dealt with as just a Note under that?

**McNeill** asked if she was recommending that this matter just be referred to the Editorial Committee on the understanding that they would likely look at it favourably?

**Karen? Wilson** thought that would be up to the meeting to decide, but that would be quite possible.

**Nicolson** asked McNeill to speak to the question of whether Art. 10.1 was actually applicable, which was about the type of the name of genus and for the purposes of designation of a type, a species name alone being sufficient.

**McNeill** thought that was true when there was only one species name but did not think it was covered if there was more than one species name.

**Gandhi** reported that they had came across a situation, sometime in 2002 or 2003, where a new cactus genus was described from Mexico. A single species was described in that genus, a new species, so there was a new generic name and a new species name and for the new species a holotype was cited. Both the genus and species carried the Latin requirement. However, for the genus, the name of the type species was not mentioned, even though only a single species was included. So based on Art. 37.5 [in consultation with?] the Rapporteur and the previous Rapporteur, they had ruled that the genus was not validly published. Since the genus was not validly published, the species name was also not validly published. Without being aware of this problem someone else from England made a new combination based on that species, which also became invalid. So, the present proposal should take into consideration the names that were already published and remarked as invalid. He suggested that maybe this was useful for something from a future date.

**Govaerts** noted that the *Code* said that you had to indicate what the type of the genus was, these days. He felt that seemed rather unnecessary when there was only one species. He had come across a number of cases now where a new genus was described with one species but the type of the genus was not explicitly indicated. He did not think it would be a useful Note because it was not self-evident that you indicate the type when describing a new monotypic genus.

**Brummitt** had notes of two examples that had come up recently, the generic name *Schunkia* and the generic name *Digitostigma*, both would be ruled invalid and the specific names invalid unless the Note was added in.

**Moore** pointed out that the discussion was entering on Articles dealing with extremely limited cases. He felt that for people that were publishing something so significant as a new genus, for heaven’s sake, please look at all of Art. 37, read all the Articles and abide by them. When it says, in Art. 37.5 you have to indicate typus after 1990 he would hope that people would do that. He argued that if they did not do it he did not know that we needed to try to accommodate them.

**Wieringa** had a warning for the present way it was written, in the case of a new monotypic genus, etc. the correct mentioning of the author reference to the type species name was sufficient. He felt this might be interpreted as you do not need a Latin description, you do not really need anything, only a new name and something like the type of a species name and it was valid. Regarding mentioning the of the word “sufficient”, he suggested that maybe something should be added like “concerning this Article”. He thought that if that was not done it stood for the entire *Code*.

**McNeill** agreed that was absolutely right. He thought that the view (which he shared) was that this should be treated as a note, if it would appear to be in conflict the requirement from 2000 for types, then that was another matter, but it was really looking at the period prior to that and it seemed to him that it was covered by Art. 10 for most cases. Therefore it would appear as a Note but as it was not at all clear, as the validity of names had been questioned, it sounded like something that should go into the *Code*. He added that it obviously would be editorially altered to fit that.

**Nicolson** was did not like the word “monotypic” because he felt it was not counting the numbers of [generic] types, but counting the number of species.

**Prop. F** was **rejected.**

[*The following debate, pertaining to a series of New Proposals by Redhead, followed by New Proposal from Wieringa and Haston regarding Art. 37.4 took place during the Eighth Session on Friday afternoon*. *The exact text of Redhead’s Proposal with Options 1 to 3 was not read out or recorded with the transcripts and must be inferred from the discussion*.]

**Redhead’s Option 1**

**McNeill** returned to considering the amendments to Art. 37.4.

**Redhead** reported that a group of had got together to try and work something out, and had come up with three alternatives, numbered 1, 2, and 3. Their preferred option was number 1. He started by putting forward a motion that the Section entertain options 1, 2, and 3 and asked for a seconder on that. He explained that they were separate options, so would need to be looked at independently of one another. He clarified that if Option 1 was approved, there would be no need to consider Options 2 or 3.

**Nic Lughadha** added that, roughly speaking, they were in order of descending rigour, so the preferred option was Option 1 and Option 2 and 3 were irrelevant unless Option 1 was defeated.

**Redhead** repeated that he put the proposal that the options be entertained.

**Buck** had a question based on one of the exceptions the other day, if someone lost their material before it was described, was that considered a technical difficulty of preservation?

**Redhead** thought that we should first accept the fact that the Section was discussing the proposal here before getting into...[This appears to have been implicitly accepted.]

**Barrie** felt that if someone who had spent several thousand dollars of grant money to go into the deepest Amazon and lost their specimens coming out, and all they had was an illustration, and could not get the material back, he thought that was enough of a technical difficulty that they should be allowed to publish their species based on the illustration.

It seemed to **McNeill** a difficulty, but not a technical one.

**Brummitt** felt that there were two main thrusts in Option 1. Firstly, people were unhappy about names being made invalid back to 1958, so insertion of the date from 1 January 2007 would get rid of that problem because all the names such as the ones Prance talked about, illustrations by Margaret Mee and so on, would now be validly published because the illustration could be the type. The second thrust of the proposal was not based on the very subjective issue of whether it was impossible to preserve something, but on a statement in the protologue, so as soon as you had the protologue you could judge whether something was validly published or not. He felt that was the main advantage of the proposal for the future, as soon as you had something in front of you, you knew whether it was validly published or not. He concluded that if the author did not say why he was choosing an illustration as a type, then his name was not validly published if he had an illustration as a type.

**Skog** thought the position of “fossils excepted” was in the wrong place as fossils must have a specimen. She thought it should say at the end of the option or at the end of the sentence “fossils excepted; see Art. 8.5”.

**Redhead** actually thought that wording was in the present *Code*...

**Skog** disagreed, saying that the type of a name of a species or infraspecific taxon was a specimen and that was always true for fossil plants, they were not exceptions to that.

**Redhead** began to suggest that if she looked at Art. 37.4...

**McNeill** interrupted to point out that this was clearly editorial, and he did not think there was any problem in the meaning.

**Atha** was opposed to the proposal because he thought it was going backward on the concept of a type specimen that took 150 or so years to put in place, and he thought it would cause future generations some of the same problems that we were having now with older specimens and older names.

**McNeill** was a little disturbed by it, not because of the general wording, but because of the date, because despite what had been presented in the initial proposal, a significant number of names had been considered not to be validly published because an illustration was designated as the type, in the 1980’s and 90’s. These were quoted in St. Louis, not the names, but that this was the case, and he had come across one or two. His point was that if people did publish the names with illustrations as types, believing the *Code* permitted it, then yes, these names would not validly be published without that date, but equally there were names that had been treated as not validly published because only an illustration was the type. He did not know where the balance lay in terms of numbers, so it could be the other way around, but he thought that if the date was not in it would certainly preserve the continuity a little better.

**Gereau** still found it completely unacceptable because of the complete subjectivity of “technical difficulties of preservation”. He wondered if we were back to “it was really spiny and too hard to press”? What was a technical difficulty of preservation? A clear statement by the author that it was impossible to preserve the specimen was equivalent to what was in the *Code* now, since the *St. Louis Code*, and would be acceptable and an explicit statement by the author in the protologue would be acceptable, but the “technical difficulties of preservation” was equivalent to allowing the “dog ate my homework” excuse and he argued that it was not acceptable.

**Redhead** responded to both that issue and the date issue. The date, at least for micro-organisms, had to be in because of things like chytrids and other microfungi, where plates had been used as types, and if that date was not there, and there was no statement in the publications, then those names might end up being declared invalid. As far as the micro-organisms went, the date was important. As far as the technical difficulties go, he suggested Gereau may be only thinking of phanerogams, but if he thought of micro-organisms, the technical difficulties could be explained in publications, as these organisms did not lend themselves to forming a type. He explained that was why that wording was there, it was not to say there were technical difficulties in hauling back a plant press, it was aimed toward micro-organisms.

**Brummitt** replied to the Rapporteur’s comments of a minute or two ago, pointing out that for most of the period from 1958 onwards, the *Code* gave an explicit statement that a holotype was a specimen or illustration with no cross reference to anything else. He knew there were different interpretations, but at least it was one possible interpretation and many people did take it at its face value. It seemed very hard to him to retroactively make all those names invalid.

**Nic Lughadha** wished to very briefly add to that. She noted that the Rapporteur may be in doubt about the balance of evidence between names being invalidated or not but the indexers of IPNI were in absolutely no doubt. The Article introduced in St. Louis retroactively invalidated hundreds of names and this amendment would rectify that. She passed to one of the IPNI compilers.

**Challis** was not aware of ever seeing any remarks in any papers that she had looked at where someone had said a name was not validly published because the author designated an illustration as a type.

**McNeill** wished to clarify that she had not indexed such a new name, replacement name or something?

**Challis** replied that in the course of indexing [for IPNI] she saw hundreds of taxonomic papers and was not aware of any names having been treated as not validly published (by subsequent authors) because the author designated an illustration as a type.]

**Demoulin** felt that, again, there was a big difference between higher plants and algae and fungi, and in algae and fungi there had always been discussion on whether it was impossible or not. He thought, as Brummitt had said, that by not trying to decide yourself whether it was possible or not, it was a clever way to say, “O.K., we see what the author says”. The point he wished to make was that he was sensible to the story of the thing that had already been fully documented with notes and pictures and so on, and the specimen got lost when the boat was run down by an anaconda, and he did not see much difference in a situation where the type had been lost before it had been deposited in an herbarium and the very frequent case where the type had been lost when being sent on loan. After a few years he argued that you would be in exactly the same situation, so he agreed we should, as an exceptional situation, allow somebody to describe [a taxon on an illustration] having lost a specimen. He proposed a friendly amendment to replace “is impossible” with “it had been impossible”? It had been impossible because it had been eaten by an elephant or something like that.

**Ahti** was afraid one word had dropped off [from the proposal]. It should be “the type of a name of a *new* species or infraspecific taxon”, like it was in the *Code*. Otherwise, he thought that all lectotypifications of old species could not have a illustration as type any more.

[The **amendments** were accepted as **friendly amendments.**]

What bothered **Buck** most about it was that it was throwing apples and oranges together. He thought that in cases of microfungi and algae, where basically every time something was described it was not going to have a specimen for a type, was one situation, and another was some circumstance for a vascular plant, when it probably could have had a type had the collector not been careless. He would much rather see this as two separate cases: one case where there was never going to be a type no matter what, and one where it was only under bizarre circumstances that there would not be a type.

**Barrie** wished to respond to Ahti’s comment. His point was that Art. 37.4 did not apply to lectotypes, only to holotypes of post-1957 names.

**Redhead** noted that Option 2 dealt with them [unclear what?] split a bit and then there was a third to fall back on.

**McNeill** wondered if the Section should see the other options?

**Redhead** offered to look at the other options.

**McNeill** clarified that he merely meant that, as they had been provided, should we see them. Of course they would have to be voted on one at a time. It was only to provide background information.

**Redhead** added that, of course they could accept other friendly amendments, to adjust it.

**Gandhi** repeated again what he had mentioned yesterday: in the late 90’s he recorded a few orchid names, and the basis for such new names were only sketches made in 1860’s. The publishing author made it clear that he never saw any specimen and he was unable to collect any specimen in the relevant locality. Gandhi asked if it was not a technical difficulty, how should they rule on the publication?

**McNeill** checked that it was after 1958.

**Gandhi** was reporting what he indexed in late 1990’s.

**McNeill** summarized that this concerned describing new species from illustrations/drawings of the last century where they could not obtain any material. He wondered if they were imaginary drawings, perhaps?

**Gandhi** felt that was his question. But, as an indexer, he did not have any choice, he did not question the author, but simply recorded, and the names were in IPNI. He continued that if they were valid they would cause homonymy if anyone wanted to use such names but if they were invalid it was OK, but we knew the ruling.

Option 1 appeared to **Haston** to be the most suitable, but she would like a Recommendation added to it, which would recommend that, where possible, if some material was available for preservation, although it may not be suitable material, it may be used for additional information such as DNA.

**Nicolson** asked if that was a new proposal that needed to be posted?

**Haston** saw it as a Recommendation to be added, if it could be a friendly amendment. [It was **accepted** as a **friendly amendment** but this was later rescinded and dealt with as a separate new motion from the floor later in the proceedings.]

**McNeill** requested some wording on the board, as the Section was just about to vote on it.

**Redhead** added that then they would see how friendly it was when they saw it.

**Peng** wondered, in the case of losing the specimen and keeping the illustration as a substitute, whether the illustration had a voucher collection number and what the status was of the lost type specimen that were found later [after publication], was it a [?lecto-]type of the figure?

**Redhead** was not certain what he meant by the “lost type”.

**Per Magnus Jorgensen** stated that a type was not a type before it was published, elaborating that if it was lost before it was published, it was never a type.

**Gandhi** wondered, regarding an illustration how one would know that it could be an isotype or any other type. The *Code* made it very clear that isotype was always a specimen, Art. 9.3.

**Redhead** pointed out that the Section were still waiting for the wording of the Recommendation.

**McNeill** apologized, suggesting that if it was a Recommendation it could be taken later, but if it was an integral part of the Article then it had to be taken now.

**Redhead** suggested it be treated separately so that the Section could move on.

**McNeill** explained that it was no longer a friendly amendment and would be taken later.

**Atha** was concerned if illustrations were to serve as substitutes for type specimens. He wondered what would be the scientific access to the illustrations because they may be in private collections, they may be in somebody’s drawer, whereas there were generally procedures regarding the curation of herbarium specimens.

**Wieringa** offered a friendly amendment [**Nicolson** interjected “We’ve already got one!”] which he thought would also solve the last problem. He wanted to insert “simultaneously published” before “diagnostic illustration”, so “when a simultaneously published diagnostic illustration may exceptionally be the type”.

**Nic Lughadha** gave the Chair of the group some thinking time. She thought it sounded like it might be part of a friendly amendment, but it could be that one would also want to allow for reference to a previously published illustration that could not be reproduced for some reason, but she deferred to Redhead, the Chair of the group who put this together.

**Redhead** did not accept that as a friendly amendment.

**Marhold** wondered if “a published illustration” solve the problem? Deleting “simultaneous”.

**McNeill** pointed out that the amendment had not yet been voted on, so it would be “a simultaneously or previously published illustration”.

**Tan** had a question for the vote on Option 1, the one on the screen, whether the Section were voting also including the additions, the two statements inside the parenthesis or just voting the main text as Option 1?

**McNeill** wondered if he meant the Examples? He clarified that the Section would be actually dealing with the text when they came to it, but at the moment an amendment to insert “simultaneously or previously published” or words to that effect was under consideration.

**Hawksworth** was against including both “simultaneous” and “published” because you may actually want to refer to an unpublished illustration [Audience groaned.]. He continued that it had certainly happened in the fungi where drawings had been used that were on packets and things separate from the publication.

**Gandhi** wanted to emphasize the fact that technical difficulties in obtaining plate types for verification always existed. He thought that at least some of the audience may know that de Candolle in early 1800s borrowed plates that were made by Sesse and Mocino on Mexican plants, and made copies of those original drawings, and based on copies of those plates new species were published in de Candolle’s *Prodromus*. His point was that for those who wanted to study such duplicate drawings, they needed to go to Geneva, on the other hand, all those published illustrations were much more accessible to the public than borrowing a holotype specimen.

**McNeill** checked that he was arguing in favour that it should be restricted to published illustrations. [He was.]

**Ahti** wanted to second the suggestion that “simultaneously” be dropped A published illustration was enough, since a previously published illustration must be accepted as the type.

**Brummitt** reacted to some whispers put in his ear a minute ago by Buck. If a specimen was designated as a holotype he pointed out that it had to be stated which herbarium it was in, whereas the present wording allowing illustrations could be wallpaper on a living room wall or...

**Nicolson** suggested bedroom...

**Brummitt** accepted the correction to bedroom. He thought a good way around this was to insist that the illustration should be published. He did not know about Redhead, but he personally would accept “published” as a friendly amendment.

**Nic Lughadha** confirmed that the Chair of the working group accepted the friendly amendment. She suggested “published or publicly available diagnostic illustration”, the reason being, that sometimes there were reasons why it was not possible to publish an illustration even though it was already in a public library, for instance.

**McNeill** returned to the point that Brummitt raised about the holotype, he thought that it would still be the holotype.

**Turland** offered an answer too, he thought it was Brummitt, who was losing track slightly, for an unpublished illustration, Art. 37.6 required that the single herbarium or collection or institution in which the type was conserved must be specified, but he mentioned that he had seen unpublished illustrations cited in protologues: in one case it was a colour transparency in somebody’s collection; it did not say that a private collection was not allowed. He added that it should also be borne in mind if a type illustration was not published, it could be electronic. He was arguing in favour of it being published.

**McNeill** summarized that “published” had now become a friendly amendment, adding that if it was not published, as the Vice-Rapporteur had pointed out, Art. 37.6 kicked in, so after 1 January 1990 it had to be in a herbarium or collection or institution.

**Davidse** pointed out that in this day and age, “published” was commonly accepted both electronically as well in print, so he thought that the objection remained.

**McNeill** replied that the Editorial Committee might very well, if it was accepted, in the light of the discussion, use “effectively published” or “effectively published medium”.

**Veldkamp** saw a conflict with “a published or publicly available illustration” with 37.6, where it talked about an unpublished illustration.

**McNeill** felt that was the point: it was either published or else there had to be a statement as to where it was preserved.

It still seemed to **West** that under Art. 37.6, an unpublished illustration, could be in someone’s private collection. It would not be excluded because it said a single herbarium or collection or institution.

**McNeill** responded that it would have to be something that could be described as a collection, exactly the same as was required for a herbarium specimen

**Atha** had a great deal of respect for everyone in the room and admired their scientific integrity, but he thought it was the people who were not in the room that he was mainly concerned about, and if this proposal passed he was afraid there would be a flood of new species published on basically anything. He argued that the Section would be forced to deal with all the superfluous species in the future.

**Nic Lughadha** clarified that the suggestion was to go back to the situation as it was understood by a large number of people before St. Louis. She argued that there were no floods at that stage and she did not expect there to be now.

**Garnock-Jones** wanted to remind the Section of a parallel example under the zoological *Code* about thirty years ago, when a new genus and species was described based on a very blurry photograph, that was published in no less a journal than *Nature*. The organism in question was *Nessiteras rhombopteryx* – the Loch Ness Monster. He wished to endorse what the second-but-last speaker said, that this was opening a can of worms which the Section might regret.

**Nee** felt it was a matter of fact that there had been a flood of published names based on illustrations, rather than specimens, and that included a great number of things from Linnaeus onwards, and they had caused untold problems. He gave the example of Vellozo’s *Flora fluminensis*, in which the illustrations were simply not diagnostic for the majority of the species treated. Even though they were big and they were beautiful, they simply did not work very well [they could not be identified taxonomically], and there were no specimens, so he argued that this was not desirable in the future.

**Nic Lughadha** highlighted that the “flood” that Nee referred to, of *Flora fluminensis* appropriately enough, was shortly post Linnaeus. She did not think it was possible to blame it on any provision of any *Code*.

**McNeill** thought the point was that it was not the type of thing one would want in the future.

**Redhead** responded to the blurry photograph of a Loch Ness Monster, with an example in mycology where the genus *Golfballia* was published, which had an actual specimen based on a burnt golf ball, so there could be fictitious things even without photographs.

**McNeill** felt there was a subtle difference. That was in the *Bulletin of the Kew Guild* and was deliberately tongue in cheek. He did not think that *Nature* knew what it was getting into, but maybe it was on April 1st, he did not remember.

An **Unknown Speaker** clarified that it was created as a joke.

**McNeill** wondered if it was published as a joke?

**Nicolson** thought things were getting exciting! And the discussion was still only on Option 1. [Laughter.]

**Gandhi** wanted to inform the audience that he had recorded a number of lectotypifications which cited the plates from *Flora fluminensis* effectively published in 1833. As an indexer, he did not know whether they were wrong or right, but was just recording, and they were published by the relevant specialist in the particular group.

**Nicolson** felt it was a complex difficulty. Stocks were sitting around for a while and then it got distributed finally, but he recommended not going there.

**Ahti** wondered if all the problems could be overcome by a Recommendation where it was stated that the Article was primarily intended for certain groups of algae and fungi and it was hardly acceptable whether any such case could be present in one of the higher plants.

**McNeill** noted that Recommendations were merely good advice, and his predecessor would have put them all into a book on nomenclature and had none of them in the *Code*. He did not take quite that view, but if it came to something that was determining valid publication, the inclusion of a Recommendation was not really terribly helpful.

**Marhold** reminded the Section that the *Code* did not protect against bad guys or people with bad behaviour. He was recently asked for his opinion on a case when a name was published, a specimen was cited as deposited somewhere but it was intentionally not deposited there and nobody knows where the specimen ended up. We still had lots and lots of names based on illustrations anyway, so he really was not worried about having some exception to have an illustration as a type.

**Glen** requested not only algae or fungi, but also succulent plants be included, thinking particularly of the genus *Conophytum*, where the individual plants were small, spherical, or vaguely cuboid little lump of plant material [**Nicolson** helpfully added the description of “Golf balls!”] with pretty colours and pretty flowers. He explained that when you pressed them there was just about nothing left and so an illustration was really, really helpful to figure out what the original author was thinking of.

**Nee** noted that no one seemed to have even mentioned, let alone explained, what the difficulty was of having a preserved piece of a *Conophytum* or a cactus or algae, which he did not know anything about, as the type and also an illustration that was diagnostic and beautiful and along with it the official type would be the specimen. He wondered what was the problem of preserving it and calling it the type and preserving the usage and the necessity for having type specimens for everything that was possible? In general, he felt it had not been explained and reminded the Section that everything that was an organism had DNA, had organic molecules, which could be and may have to be used in the future for the identification. He thought the strongest possible language was necessary that we should be preserving type specimens and only very specific possibilities where it was not possible. What all of these three options lacked, it seemed to him, was to be carefully thought out with specific exceptions for the types of organisms for which it was impossible, or almost impossible, and those were mainly the ones that most of people at the Section did not have any experience with because they did not work with microscopic algae, for example. He suggested putting those in as the exceptions, very carefully explained, and just did not really see that in any of the options.

**Redhead** replied regarding the micro-organisms and perhaps the microalgae portion. He explained that in some cases these micro-organisms were in a slurry of other organisms, and it was not possible to actually cultivate them in pure culture so you could not even have a smear of a single organism, he was thinking of the rumen chytrids which were grown anaerobically and were very difficult to grow and were mixed in with all sorts of bacteria and protozoans. Making a smear and then trying to determine which dot dried on the slide was the actual organism versus a photograph of what you took to be the organism was the way to go because nobody would ever find it again, and it was very difficult to maintain it in living culture because of the growing conditions, so you could not even lyophilize or freeze-dry them. He was sure there were similar cases amongst some of the microalgae. At least for the micro-organisms he felt there was every reason you would want to have an illustration serving as type, even if you tried to maintain a culture for an ephemeral time. He could not speak towards the phanerogam portion of what was under discussion here, so perhaps someone else could.

**Nicolson** felt that the same problems were being repeated.

**Delwiche** spoke as someone who did work with microalgae, which did not preserve well. He emphasized that researchers wanted a specimen if it was humanly possible to do it. He thought it was desirable to retain language which as strongly as possible disparaged the notion of a description which was based purely on a drawing, and felt that if the door was opened too wide to allow descriptions based purely on drawings it would be regretted. He had to work with these situations and he described it as miserable, adding that the specimens were miserable too, but you were better off when you had a specimen.

**Haston** thought a lot of people had been discussing the possibilities of the use of DNA in specimens, and felt that dependence on DNA possibilities in specimens was perhaps misplaced. She had worked on herbarium material extracting DNA, and thought that maybe a lot of people who were here may agree with her that it was not always possible to extract DNA from plant specimens that were existing already.

**Paun** wondered if he was one of the guys that Stuessy’s book for nearly dummies was intended, as he did not have so much experience in nomenclature, but wanted to point out that if you ever tried to borrow type material to extract DNA from it, you would never get it as it was not intended for DNA and herbarium curators would not allow it.

**Per Magnus Jorgensen** thought Option 1 was better, because the Examples showed which organisms or things that it could be allowed in, rather than having it as a long list in the text as some people seemed to like.

**McNeill** clarified that at the moment there were no Examples, discussion was just about the text. He added that there may be Examples, but the Examples did not limit the Article and it was only the wording of the Article that determined the application.

**Per Magnus Jorgensen**’s point was that the Examples would show what was allowed.

**McNeill** disagreed, explaining that the Examples would show the groups where it was thought to be most applicable. He reiterated that it was the wording of the Article that would determine what was actually allowed for the purpose of valid publication.

**Per Magnus Jorgensen** persisted that his point was that this did not open the door wider than it already was.

**Nicolson** wondered if the Section were ready to vote after a good debate, as it was still dealing with Option 1. He allowed one more comment.

**Freire-Fierro** stated that, for comparison purposes, it would be very difficult for a taxonomist to deal with a new species based on a short diagnosis and a sketch of the plant. Also, although now it was difficult to obtain DNA from type specimens, at least there was an option of doing so in the future. With an illustration instead of the type specimen, this option was completely lost.

**Nicolson** moved to a vote on Option 1.

**Redhead’s Option 1** was **rejected.**

**Redhead’s Option 2**

[*As noted above, the exact text of Redhead’s Options 1 to 3 was not read out or recorded with the transcripts and must be inferred from the discussion.*]

**Redhead** admitted that they were worried that Option 1 would not be accepted, so had Option 2, which attempted to split the levels of requirements for micro-organisms versus the vascular plants, and had slightly different requirements for the two of them. He emphasized that they would certainly entertain friendly amendment of it as well. He thought, ultimately, at least for the micro-organisms, it was essential that illustrations be allowed to serve because it invalidated quite a few species and genera, and his understanding from the vascular plants was that if there was no date, which was sometime in the future or at least the present, that there were many names out there that would be invalidated as they currently existed and had already been published.

**Dorr** moved that discussion be closed on Option 2. [This was seconded.]

**McNeill** clarified that there could be no more discussion until the matter was resolved and voted on. He added that there would normally need to be a two-thirds majority for such a motion to carry.

**Dorr** reiterated that he had moved that discussion on Option 2 be closed and it was seconded. His intent was to force a vote on it.

**McNeill** also reiterated that the motion to terminate discussion on Option 2 in order to take a vote on it must now be put and a two-thirds majority was required for it to pass.

**Nicolson** asked for all in favour of the Option 2...

**McNeill** interrupted to correcting to all in favour of discontinuing any further discussion on Option 2 because it had all been covered and to take a vote at once.

**Nicolson** moved to the vote and concluded that the “ayes” had it. [Pause.] Oh!

**McNeill** thought it was just about two-thirds.

**Nicolson** thought it was.

**Demoulin** strongly opposed what was going on here. First, he felt there was obviously no two-thirds majority. Second...

**McNeill** apologized, agreeing that there was not obviously a two-thirds majority but assured him that it was very close to a two-thirds majority looking at it.

**Demoulin** continued that with a proposal like this, it was extremely unfair to those who had worked on preparing various options. He found it incredible that the Section could not be allowed to discuss all the options. Second, he was going to propose a friendly amendment to Option 2, and he was not allowed to do that, while he suggested that allowing the possibility for amendment may lead to people not being opposed to discussing it.

**McNeill** explained that a vote had been taken and the only thing that could be questioned now was whether in fact there was or was not a two-thirds majority. The Chair had ruled there was, and there was no reason for to doubt his ruling, but that was the issue that could be questioned.

**Nicolson** decided on a show of cards for a vote in favour of closure. He was not sure there was two-thirds.

**McNeill** summarized that the President did not feel there was two-thirds from that show and therefore discussion would be continued.

**Demoulin** wished to present his friendly amendment. [Laughter.] He had listened attentively to what had been said, and he kept feeling that it was an important issue for algae and fungi not to devalidate things which had been done. He referred to a gentleman who said he worked with microalgae and was happy with specimens, and if you had things that grow well in culture of course it was not technically difficult to preserve a useful specimen, at least for DNA studies. So he summarized that they were not concerned by the option. But there were things that did not grow well in culture and which would be studied on mixed sample, and he assured the Section that if there was a mixed sample it was hopeless to think that you had solved the things with DNA studies. He had a student that had been spending a lot of time and money in the last six months trying to find a procedure to extract DNA from all the limited number of one group of algae in a natural sample, and it was impossible. Now, the main issue: he hoped very much the phanerogamists would not stop the algae and fungi people having the things they needed, but when it came to the higher plants he heard that there were lots of, and he thought viable, objections that there should be no abuse of this system, and it was probable that with the wording that was there that it would be abused with the sentence “if an illustration better served the purpose in the eyes of the author”. His friendly amendment was to delete that sentence.

**McNeill** which sentence?

**Demoulin** “... illustration better served the purpose in the eyes of the author”. That was where he thought there was a possibility of abuse.

**McNeill** commented that after doing that he was not clear of the difference between before and after 1 January 200x, presumably 2007. He felt that “impossible to preserve a meaningful specimen” and “impossible to preserve part of the original material” seemed pretty well the same to him.

**Kolterman** was uncomfortable with the use of “original material” in this context, because it obviously did not mean what “original material” was defined as in the *Code*.

**Nicolson** was concerned about coffee break time, but allowed one more comment.

**Wieringa** suggested adding “published” before “illustration” and hoped it would be accepted as a friendly amendment again. [It was.]

**McNeill** had no particular view on it, but just for clarity, he thought that if you just dropped everything after the first “type” in the last line you would have the same meaning. Where “of all the plant it were impossible to preserve a meaningful type”. The meaning seemed the same to him, but whether that was what was wanted, he did not know.

**Barrie** was having a hard time understanding exactly what it meant. How many different dates were there, were they all the same date or were there three different dates?

**Redhead** clarified that they were intended to be the same date but they had not established which year.

**Barrie** was also having problems with the way it was punctuated. He could not tell if algae and fungi were not supposed to have any date, and therefore were separate from the other ones, or what. He found the way the whole thing as written was very confusing to understand.

**Redhead** apologized for his poor grammar. He clarified that the colon was to indicate that there were two different types of requirements coming out: one pertained only to the algae and fungi “if it was technically difficult or impractical to preserve a useful specimen”; and there was supposed to be a semicolon after that, which had disappeared and turned into a comma somehow, “or for other plants up to 1 January [200x] if it was impossible to preserve a meaningful type”. So there were two different sets of criteria.

**McNeill** suggested that the date could disappear for the second one, having decided that the two clauses meant the same, so the date could disappear for the other one.

**Redhead** agreed.

**P. Hoffmann** wondered whether in Option 2 the omission of the requirement to state in the protologue that it was impossible to preserve a specimen (compared to Option 1) was intentional or an oversight?

**Redhead** had phrased it that way because he felt in almost all cases the lack of an actual specimen, at least for the fungi, could mostly be explained by it being technically difficult or impractical to preserve them, rather than being impossible.

**McNeill** asked the proposer why there was a date there at all. It seemed to him that the whole Article should not have a date as it was now presented. The only date was when there was a difference between the treatment for other groups which had been taken out, so it seemed to him applicable right back to 1 January 1958.

**Redhead** explained that, in part he was trying to leave open for the algae and the fungi, the micro-organisms, an indefinite date backwards and forwards. For the vascular plants, one of the primary issues that had come up was the fact that it would invalidate a lot of names in the past, but perhaps the requirement for a specimen could be more rigorous in the future. He was trying to build that into it.

**McNeill** pointed out that he had accepted it as a friendly amendment, the bit that made that distinction; he had been a little surprised that Redhead had accepted it, but he had, and that being the case, McNeill thought the date was in appropriate. He added that what had been “if it was impossible to preserve a specimen”, had been tightened up very slightly by saying “if it was impossible to preserve a meaningful type”.

**Redhead** suggested that perhaps he would take back that friendly amendment. [Groans.]

**Nicolson** decided it was time for break, but as Zhu had not spoken before, he got the last word.

Generally speaking **Zhu** thought Option 2 had a semi-improvement over Option 1, but was still not good enough to be voted “yes”. Besides the problem with the dates, he always found it was difficult to understand how to make a judgment based on an Article which was not exact, and the Article should be as accurate as possible. He felt that it would be better off without the words “useful” and “meaningful”. He argued that a specimen would always be useful but it depended on how much use it was going to be. He added that it may not be useful now, as it was mentioned yesterday, but it could be very useful in the future with the improvement of technology.

**Nicolson** thanked everyone and announced the break. He was very pleased to be getting to his coffee fast enough.

After the break **Redhead** explained that they were going to take option 2A as a friendly amendment and delete Option 2. He added that it was virtually all the same but it was better just to type it all together. He also noted that they were going to eliminate Option 3. He did not want to prolong the debate. He was personally in favour of a vote on option 2A, almost immediately, and then a discussion of Option 3, which separated the issues.

**Funk** was just curious why, when there was no date in the *Code* now, we were putting the date of 1 January 2007 for other plants?

**Redhead** responded that there were requirements for the two different types of groups, and it was more rigorous for the vascular plants.

**Funk** reiterated that there was no date in 37.4, so why introduce a date?

**Wieringa** replied that the reason was that before 2000, it was quite possible, according to the then followed *Code* to publish a name with an illustration as the type only, and all those names had retroactively become invalid. He argued that introducing the date would prevent all these names remaining invalid and make them valid again, because they had only been invalid for five years.

**McNeill** thought that the wording had two possible meanings, or rather it had one meaning but it was not well presented. He thought, from what he had just heard said that was not the intended meaning, because as it read it would be “for other plants only when it was impossible to preserve a specimen and from 1 January 2007 if such was stated in protologue”. That seems to be its meaning, but that was not what he thought was being stated to be its intended meaning.

**Gandhi**, as mentioned earlier, had indexed names in late 1990s which were solely based on sketches, so if this particular date was accepted in the Section then those names would be invalid.

**McNeill** suggested that the first lines would be the same for fungi, but then it would be “or for other plants only if it was impossible to preserve a specimen and after 1 January 2001 if such was stated in the protologue”. He felt that would be clear, but was not certain that was the intended meaning.

**Redhead** agreed that was clear and had the intended meaning.

**Alford** was still going to vote against. He felt some sympathy for Option 3 because people, say chytrid experts, for example, in good faith actually described something with an illustration before the *St. Louis Code*, but presently there already was the epitype option to deal with difficult situations. So he thought, even in the worst possible cases, if a circle was drawn around a spot on a slide, you could still have an illustration as an epitype which, according the *Code*, would serve as the interpretative type. He added that 55 years ago we did not even know the structure of DNA, so 55 years from now we may be able to pinpoint a location on the slide and with particular spectrophotochromatic methods sequence the DNA of a smudge so he thought it was necessary to look to the future and just deal in the simplest way with what was already in the past. He suggested that in the case of the chytrids to let them go but say for the future that a real specimen that we could actually be examined was needed. He added that it was too bad Dick Korf was not present to do this in a more theatrical way, but he certainly supported his position as expressed in St. Louis.

**Atha** wondered, irrespective of algae, in the vascular plants in what situation it was possible to produce a drawing, or painting, or watercolour, or photograph even, but have it impossible to make a specimen. He understood that the specimen might be lost, and that was a particular case where at least an attempt was made to make a specimen, but he did not think we should sanction no attempt at all to make a specimen.

**Demoulin** noted that the amendment he proposed to Option 2, accepted as friendly, still held for what was being discussed here, and that was to replace “it is impossible” by “it had been impossible”.

**McNeill** did not understand the difference.

**Nicolson** suggested change “is” to “it has been”.

**McNeill** did not know what that implied in terms of practicality.

**Demoulin** responded that it was because the sentence “it is impossible” was what had always been in the *Code* for the fungi and algae and had always made problems with the fungi and algae because it was a very subjective matter. He felt that there were people who considered that you could always preserve a specimen, but there were a lot of people who had been very conscientiously working with their groups and would consider that it was meaningless to conserve a specimen. He resisted the urge to teach biology and offer a lot of examples where there was no meaningful specimen possible. He felt that using “it has been” covered the situation as he said before where it was impossible because it had been lost just before you could deposit it.

**McNeill** thought there was an implication that with “has been” that it “now is” possible, which was why he found it puzzling.

**Rijckevorsel** wished to move an amendment to the amendment and...

**McNeill** asked him to wait a minute in order to clarify something. He found what Demoulin said puzzling on a procedural matter, as McNeill was under the impression that 2A was in fact what we had on the board before with the friendly amendment and a bit of recasting. It had then been recast again because he did not quite understand what the date meant, and now do, so there was no going back to some other wording that Demoulin was suggesting.

**Rijckevorsel** suggested instead of “has been” to use “proved”, “proved to be”, “proved impossible”, so in this case...

**McNeill** pointed out that the words that were on the board that he just changed, was “was”, “if it was impossible”, as opposed to “has been”, which was clearly inappropriate. He wondered why it was being changed from the original wording, from “is”, but he acknowledged that it was not his proposal so if it was “was”, so be it.

**Redhead** explained that they had forgotten to put Demoulin’s wording in the revised version so that “has been” was fine, but not “proved”. He felt that it was not desirable to play with it much more, and it was time to test the waters with Option 2A. He reminded the Section that Option 3 had been withdrawn, and discussion would move to Option 4 if 2A did not pass.

**McNeill** continued to wonder why it was not just “was” rather than “has been”? “Has been and now is” was the implication.

**Demoulin** stated that there were two parts in the problem. Algae and fungi, where indeed a change from the present situation was desperately needed, which was Option 3, and the Section hated that, where it said it was impossible, because there were always people who would tell you it was possible. He felt it was a major improvement with the expression “technically difficult or impractical”. There was a problem of the higher plants, and he was very sorry that because the two were mixed something important may be lost for a given community. He explained that that was why he was trying to restrain as much as possible what applied to higher plants, so that the higher plant people who did not want the illustration did not kill the precious algal and fungal part of the proposal. He suggested that another possibility was to split the two things and have one thing for algae and fungi and one thing for higher plants. But he thought “has been” was a good way to say that there had been certain circumstances that made it impossible to preserve a specimen, while saying it was impossible was problematic as other people would come and say, “Oh no, you can”.

**Nicolson** moved to a vote on option 2A. He thought it failed and asked if the Section would accept his ruling? He thought it was 60%.

**Redhead** also thought it failed and suggested, to expedite things, and Option 3 was withdrawn, discussion should move to Option 4. He started to explain that they entertained putting the idea of fungi in the parentheses on the existing Article to make them an exception...

**Dorr** interjected with a point of order, which he noted took precedence. He highlighted that there were three options at the beginning and now there were four. He had seen no motion for a fourth option to be presented to the Session. He wanted procedure to be followed in this place.

**Barrie** [off-microphone] pointed out that this was the third, because the previous third one was withdrawn.

**Dorr** insisted that you could not rename the options and then introduce them as new things. He summarized that there were three options, one of them was amended, the first one was voted down, the second one was voted down, the third one was withdrawn, and they had presented a new option. He maintained that it had to be presented as a motion from the floor, with assent.

**Barrie** moved for discussion of Option 4.

**McNeill** noted that there was a request for a card vote on Option 2.

**Nicolson** thought that would take priority.

**McNeill** ensured that the screen showed Option 2A and instructed the Section to throw vote number 6 out and use vote number 7 as 6 and 9 were not distinguishable.

**Redhead’s Option 2** as amended (2A) was **rejected** on a card vote (171: 313, 35.3%).

**Redhead’s Option 3** was **withdrawn.**

**Hawksworth’s Option 4**

**McNeill** returned discussion to addressing the issue of 37.4. He noted that Option 4 would be an addition to the existing words.

**Redhead** explained that the proposal was put forward by Hawksworth and seconded by the group, and it was an addition to the existing 37.4 which was approved earlier in the meeting, and detached the requirements for the vascular plants from the fungi and algae. They were hoping that the addition of this to the existing Art. 37.4 would pass.

**Barrie** thought it was very important to get it in for algae and fungi, because there were far too many names that were now endangered, that were already in publication or in use, many of which he was sure had important use in medicine and other cultural research. Like most vascular plant people he was not happy unless specimens were glued to a piece of paper, so was quite happy to keep illustrations out for vascular plants in general, but he thought this was needed. His one question was did the Section still want to have “impossible” again?

**Demoulin** agreed that this was better than the present situation, but felt that some of the wording in the first option was better, and why not use the same wording regarding technical difficulties of preservation as was Option 1 in this one, which was so strictly for algae and fungi.

**McNeill** asked if he was proposing an amendment?

**Demoulin** was if the proposers accepted it, as he was not really a member of the group.

**McNeill** noted that it did not strike him as enormous difference in meaning between the general situation and the situation for algae and fungi, as presented, meaning from the type and possibility to preserve a specimen.

**Demoulin** felt it was an improvement, but thought that “technical difficulty” was an even better one.

[The results of the friendly amendment appeared on the screen.]

**Buck** also proposed a friendly amendment, to put the word “micro” [“microscopic” on sheet] before algae and fungi, because if it turned out to be for mushrooms and macroalgae then he was going to vote against it.

**Watson** acknowledged that Hawksworth did not particularly like it, but suggested putting “published” back in front of illustration as a friendly amendment.

**Nicolson** reported that “microfungi” was accepted as a friendly amendment.

[Pause with off-microphone discussion and editing of wording on screen.]

**McNeill** pointed out that it was not altogether clear that the adjective “micro” applied to both algae and fungi.

**Nicolson** asked if the principle was acceptable, because if it could be worked out in Editorial Committee discussion could go on. He also wanted to understand what Watson’s proposal was.

**Watson** explained that his proposal was to insert “published” before illustration as in the previous options.

**McNeill** reported that that was apparently not accepted as friendly, but it could be moved as an amendment if he wished. [The amendment was seconded.]

**Watson** noted that the algal people at Edinburgh really wanted the illustrations to be with the publication and not separate.

**McNeill** stated that the amendment needed to be addressed first.

**Dorr** asked for clarification of what was on the floor. He had been following the argument rather closely but did not have any record of what happened to Option 3. He thought the discussion was solely on Option 4, but it was not at all clear to him that that was what was on the floor.

**McNeill** replied that Option 3 had apparently been withdrawn and it was still on the screen because it was difficult to remove.

**Dorr** pointed out that it should never be *apparently* withdrawn. It was either withdrawn or it was not withdrawn.

**McNeill** apologized and stated that it had been withdrawn. He was **told** it had been withdrawn. These words were additional to the existing Article currently in the *Code*. He added that obviously the Editorial Committee would combine them in some way.

**Buck** again, noted that if the illustration could be a painting that was on his living room wall he was going to vote against it, because that was inadequate! That was exactly how he thought it read now. He thought the Section had never voted on whether it was a published illustration as it was an unfriendly amendment and he strongly felt it had to be published if he was going to vote for the whole thing.

**Nicolson** thought that was a new amendment.

**McNeill** said it was an amendment to the proposal Option 4 to have “published illustration” as opposed to just “illustration”.

**Redhead** accepted that as a friendly amendment.

**Wieringa** also had an amendment to make sure that all descriptions which only used plates, all illustrations before 2000, or 2006, which at that time were valid and were no longer valid, that that could be repaired. He suggested that could be done by adding the sentence “or for other plants only until 31 December 2006”.

**Redhead** did not think that was necessary as he felt it did not invalidate anything.

**Wieringa** continued that the wording was fairly harsh for after 2006 because there was nothing like “impossible” or “impractical”; after 2006 it became impossible to use illustration. He wanted to repair the situation that there were a lot of names from the last century that were published using an illustration and which had now become...

**McNeill** interrupted that the last century was irrelevant as the discussion was only about names that were proposal on or after 1 January 1958...

**Wieringa** countered that that was the last century.

**McNeill** apologized and thanked Wieringa. [Much laughter.]

**Demoulin** said “please, please”…

**McNeill** and **Nicolson** asked [Demoulin] if he was seconding the amendment?

**Demoulin** [Shouting.] “No! Not at all!”

[It was seconded by someone other than Demoulin.]

**Demoulin** [Clearly agitated.] pleaded that the Section not mix up something that was general and that was referring to higher plants with those three lines, which should be absolutely just for algae or fungi. He felt they did not understand what the Section had been doing for one and more hours, explaining that it was just coming down to an addition that was just relevant for microscopic algae and microscopic fungi. He argued that putting in something else was again compromising all that work. He entreated that if there was something with higher plants, it should be a separate sentence that was general or was just for higher plants, but did not interfere with the algae and fungi.

**Nicolson** believed he was speaking against the amendment. [Laughter.]

**Wieringa** offered to make a separate line then if Demoulin liked that better.

**Nicolson** checked that he was withdrawing the amendment? [He was.]

**Nicolson** thanked him.

**Wieringa** added that the Section would come back to it later. [Laughter.] He wanted to make the specification in case somebody had a vote again that the Section would start talking about this case indefinitely.

**Nicolson** returned discussion to the text on the screen.

**Landrum** wondered if it should be “effectively published”, it was “published” but not “effectively”.

**McNeill** thought that was what it should mean from the point of view of the *Code*.

**Nicolson** reported that was accepted as a friendly amendment.

**McNeill** wondered if the Editorial Committee could make it a little more concise?

**Hawksworth** said something inaudible off-microphone.

**McNeill** thanked him, that was what he wanted to know. [But we will never know what it was.]

**Nicolson** moved to a vote on the proposal as it appeared:

Add a paragraph to Art. 37 to read: “For the purpose of this Article, the type of the name of a new species or infraspecific taxon of microscopic algae or microfungi may be an effectively published illustration where there are technical difficulties of preservation or it is impossible to preserve either a meaningful type or part of the original material.”

**Hawksworth’s Option 4** was **accepted.** [Applause.]

**Wieringa’s Proposal**

**Wieringa** asked if he could now have a proposal to add a line for all other plants that the type of a species or infraspecific taxon, fossils excepted, etc. may be a published illustration only until 31 December 2006, which was to repair the situation that completely validly published names before 2006...

**McNeill** pointed out that there was still in the *Code*, unaffected by this proposal that was just accepted, the present wording of Art. 37.4, which was probably what Wieringa would want to amend. It said “The type of the name of a new species or infraspecific taxon, etc., may be an illustration if and only if it was impossible to preserve a specimen.”

**Wieringa** agreed that his proposal would replace that Article, together, of course, with the motion on microalgae, because the problem was...

**McNeill** suggested forgetting the motion on microalgae, that had been accepted and the Editorial Committee would meld them. He suggested that the Section would assume that any proposal Wieringa made excluded microscopic algae and microfungi. So for other groups he would want to amend it in some way.

**Wieringa** felt that the whole point was that the first Article being talked about did not have a starting date, 1958 implicitly...

**McNeill** suggested it would be helpful if the Section could see the proposal in writing. He summarized that the only thing that had been passed was Option 4 as an addition to the existing Article. But if there was a feeling that the Section accepted some further amendment, seeing as so much time had been spent on it, he felt it worth getting the matter settled. However, he did not want to spend time talking about wording, but wanted to see a clear wording because it had been discussed quite enough.

**Wieringa** read out the exact wording to replace 37.4 with “For the purpose of this Article the type of name of a species or infraspecific taxon, fossils excepted (see Art. 8.5), may be a published illustration only until 31 December 2006.” He reiterated that this would be added to the accepted text for algae and fungi and that would not fall if the new proposal was accepted. He explained that if it was accepted, it would remove the retroactive nature of the present Article. He felt it would also improve the current wording, which was quite unclear, with “impractical” and “impossible”, it meant that after 2006 illustrations for higher plants and for non-microalgae would be impossible. So for the future it would be very harsh, but for the past it accepted things which had been created under a then-followed *Code*, because before 2000 illustrations were acceptable, so people were just following the *Code* when they were using illustrations as a type.

**Barrie** thought there were already enough starting points. He also thought the current wording worked fine. He wished to see the Article stay as it was now, with the second sentence added. He thought it was perfectly clear and worked great.

**Nic Lughadha** rebutted that the current wording did *not* work fine. She argued that it created an impossible situation for indexers or anybody to decide whether it was impossible to preserve a specimen or not and left the community in doubt about the validity of many names. She added that it was not the wording that was passed, that wording was not voted on in St. Louis. She felt that the current situation was not fine and the proposal would help a lot, and she supported it.

**Brummitt** added in support of hundreds of names published between 1958 and now, wondering what to do with them? Were they validly published or not? If an author retroactively published a note saying “It was impossible to preserve a specimen”, did that make it retroactively valid? He felt it was a nonsensical situation.

**Nicolson** asked if he was speaking in support of the proposal?

**Brummitt** was indeed.

**Atha** might support the proposal if “a published illustration” was changed to “any illustration, plate, figure, or anything of the kind”, anything but a specimen was unacceptable.

**Nicolson** asked if that was an amendment?

**Atha** [off-microphone] clarified that what it should be, in effect was “must be a herbarium specimen, period, after 31 December 2006”.

**McNeill** thought that was the intention. What the proposed wording was saying was that, for it to be validly published prior to 1 January 2007, it had to be an effectively published illustration, whereas the suggested deletion would just make it any illustration and he was a little surprised Atha wanted that.

**Atha** did not see “specimen” anywhere there, and would like to see “herbarium specimen” mentioned somewhere in the Article.

**McNeill** took the point. He added that, while in the “published” there was a suggestion that unpublished illustrations would go on being available, that was clearly not the intent of the proposal, and that would be made clear, but he thought putting in specimen and after 2007 would resolve that.

**Knapp** wanted to point out to Nicolson that if the word “published” was taken out it actually made the situation much, much worse, and leaving the word “published” in was actually quite important.

**Gandhi** felt that the need was clear that after 2006 an illustration could not serve as a type for macroplants. He argued that it could not hurt to have a statement cited there that it had to be a specimen.

**Bhattacharyya** was worried about the choice of December 2006 in case the new *Code* would not be published and not be available to the general public. In that case he wondered how it would be determined?

**McNeill** could not, of course, say when the *Vienna Code* would actually appear, but all previous *Code* s had appeared about one year or less than one year after the Congress, i.e. the middle of 2006 in this case.

**Freire-Fierro** was a little confused regarding the two lines instead of all the three lines, where it said “Replace Art. 37.4”? She wanted to know if the new proposal 37.4 replaced the one just voted for?

**McNeill** explained that the present Art. 37.4 would be replaced by the red lettering on the board, both what had already been approved and the new proposal. There had been a suggestion, which he thought was accepted as a friendly amendment, that some clear statement that after 1 January 2007 the type must be a specimen be included.

To **Barrie** there seemed to be a contradiction between what was on the screen and what had just been voted on, because it looked to him like the first couple of sentences would then negate using illustrations for microscopic algae or microfungi and it seemed to be logically inconsistent.

**McNeill** highlighted that it was made quite clear in the preamble the proposer made that this was complementary to, and not in conflict with, what had just passed. He acknowledged that there was obviously a need for editorial merging, but it was easier to deal with the existing wording and change that and then bring in the issue for algae and fungi as an exception. He emphasized that the proposal was not in any way invalidating what had just been approved as it was really dealing with other groups of organisms.

**Gereau** felt it could have absolutely no restriction on the use of illustrations as types from 1 January 1958 until 31 December 2006, and that was completely undesirable. He argued that there were retroactive requirements for valid publication all the time giving several examples: Art. 36.1 required a Latin description beginning in 1935, invalidating many names published after 1935 without Latin descriptions; Art. 37.1 required designation of a type specimen beginning in 1958, invalidating many species published after that; Art. 37.6 required the designation of a specific herbarium in which the type was located beginning in 1990; and so forth, and so forth. He thought the effect of Art. 37.4, as currently written, was completely desirable and it should be presented, debated and voted upon six years from now and left alone until then.

**Nic Lughadha** the retroactive requirements quoted for the other Articles were correct, and she would simply point out that all those Articles were clear cut. It was easy to see if a Latin diagnosis was present or not. She argued that you could not see or interpret whether it was impossible to preserve a type.

**Wieringa** responded to Gereau by saying that all those other Articles were implemented from that day onwards, so that date 1 January 1958 for assigning a type had been in the *Code* since that date. It was not that suddenly in 2000 a Section decided that you needed a type since 1958, but during all those years authors who had been publishing names could have been aware, when they had the *Code*, that they should do it. Only in this case, when they had the *Code* in 1980, they were not aware that they were not allowed to use an illustration, and still now we were going to say that they were wrong doing so. He felt that was the whole point with retroactive laws that you were imposing. They should be imposed from the date that you do it, and you should do it afterwards.

**McNeill** wished to clarify the actual situation, noting that the phrase “the type may be an illustration only if it was impossible to preserve the specimen” actually went back to 1935. What only went back to St. Louis was the clear statement that “if and only if it was impossible to preserve the specimen”. There were two alternative and defensible interpretations up until that time. He argued that it was not something that suddenly appeared; it was something that suddenly became clearly mandatory, whereas previously it was open to divergent interpretation.

**Nic Lughadha** begged to differ with the Rapporteur: the “only” was not in there the “if” was there but not the “only”.

**Dorr** felt it may merely be editorial, but was very uncomfortable with having a sentence that said “on or after the 1 January 2007 *it* must be a specimen”. He felt it would never be clear what “it” was unless it stated that “the type” must be a specimen.

**Nicolson** asked if that was a friendly amendment?

**Brummitt** repeated that for most of the period from 1958 through to 2000 the *Code* said a holotype “may be a specimen or illustration”. He felt it was as simple as that, so people made illustrations as types in good faith.

**Funk** pointed out that the date should be 2000, since that was when the stricter regime was implemented, and not 2007.

**Nicolson** asked if that was accepted as a friendly amendment?

**Wieringa** was pretty neutral about it and suggested the Section could even vote on which date might be best. It seemed to him that there was a lot of confusion since 2000 because of the discussions whether or not Art. 37.4 was implemented rightfully or not in the *Code*. He explained that was why he put 2007, but if 2000 was better he could live with that.

**McNeill** suggested that if the Section wished to move the date earlier 2001 would be more consistent.

**Funk** just hated the waffling aspect of “now you can, now you can’t, now you can, now you can’t”, and she thought that since the decision was made in 2000 the Section should stick with the decision made in 2000.

**Nicolson** noted that the *Code* came out 2000.

[McNeill, whispered to Nicolson “Yes, but everything else was 2001...”]

**Wieringa** felt there was only one problem in the wording in the present *Code* so from 2000 until now it was possible to use [an illustration as] type for a higher plant if you thought it was impossible to preserve a type, and with this wording that would again retroactively be made impossible which he felt was not very good, so then 2007 was better.

**Nicolson** asked if there was an amendment that was accepted? He concluded that it was an amendment that had not been accepted. He thought the Section needed to vote on the amendment.

**McNeill** clarified that the proposal was to amend the proposal by inserting 1 January 2001, as against 2007.

**Gandhi** supported the amendment, and 2001 appeared to be the appropriate date because the “Black Code” was available sometime from mid or late 2000.

**McNeill** felt that too many different dates was undesirable, so suggested sticking with 1 January, whatever year it was.

**Mabberley** thought he had lost Wieringa’s point. He thought that Wieringa was saying that people between 2000 and 2007 would have been acting in good faith if they felt it was impossible to preserve a specimen and an amendment to 2001 would act retroactively against their good intentions. Therefore, he did not think that the amendment helped.

**Marhold** thought it should be consistent. If it was possible to publish “if it was impossible to preserve a specimen” until today he felt 2001 should not be used.

**Nicolson** moved to a vote on the amendment to change the date to 2001.

[The **amendment** was **rejected.**]

**Turland** referred to the proposal that was up on the board, looking at where it said “For the purposes of this Article”, in other words after 1958, “the type of the name of a species or infraspecific taxon, fossils accepted, may be a published illustration only until”. He felt that prescribed in favour of it being a published illustration between 1958 and the end of 2006, but it did not actually prescribe against an unpublished illustration, with the current wording, as far as he read it.

**McNeill** agreed it would be necessary to say may be a specimen or a published illustration.

**Nicolson** wondered if that was editorial and could be handled in Editorial Committee?

**Turland** was trying to find out what the proposer wanted to happen, what effects the Article should have on unpublished illustrations between those dates.

**Wieringa** wanted unpublished illustration not to be types in the period.

**Norvell** suggested changing it to “illustration or specimen until 31 December 2006; on or after 1 January 2007 the type must be a specimen” and then go into the microfungi and microalgae. She added that would take out “published illustration”, put “be an illustration or specimen” because it needed to be addressed that both of those were being covered from 2001 until now.

**McNeill** wondered if that was acceptable to the proposer? [It was.]

**McNeill** checked that it would be “specimen or published illustration”.

**Wieringa** thought it was even better worded if it said “may” next to “a specimen be a published illustration”.

**Nicolson** thought that what was there was clear enough, it almost certainly would need some editorial attention to make it more pointed, but he did not think there was any ambiguity as to the meaning.

**Landrum** thought, just to be clear, it should be “effectively published” or take out “published”. He felt that there was a very narrow grey area of published and not effectively published, and that was what was possible now.

**McNeill** asked for confirmation that he was asking “effective” be in.

**Landrum** thought so. [That was accepted as a friendly amendment.]

**Veldkamp** thought it would be more clear if the words were moved around a bit and said “may be either a specimen or until 31 December 2006 an effectively published illustration”.

**McNeill** thought that did not change the meaning, but felt it was a very good editorial improvement there. [That was also accepted as a friendly amendment.]

**Norvell** felt that, as the Article had stood in the past six years, neither “effectively published” not “published” had appeared, and if the aim was to reflect what was in order since 2001, “effectively published” needed to be taken out.

**McNeill** pointed out that it seemed as though the proposer was quite prepared to have that restriction, otherwise he would not have accepted it as a friendly amendment. He checked that Norvell was proposing it as an unfriendly amendment. [She was. The amendment was **seconded**]

**Veldkamp** corrected that what he said was “either a specimen or until 31 December 2006 an effectively published illustration”, pointing out that the date should come before the illustration.

**McNeill** thought it was a great improvement and did not think it changed the meaning. So to facilitate things late in the afternoon he thought the Section would vote on an imperfect version that had the same meaning.

**Mabberley** repeated that he thought the comment from the front of the hall was absolutely right, that people had been acting in good faith with the existing text, which did not refer to “effectively published”. So unless we removed “effectively published” it was discriminating against those persons who had acted in good faith for the last six years.

**Nicolson** moved to a vote on the amendment to the amendment? [The **amendment** was **accepted.**]

**McNeill** summarized that “Effectively published” was removed.

**Nicolson** moved to a vote on the amended proposal:

Replace Art. 27.4 with: “For the purpose of the Article, the type of the name of a new species or infraspecific taxon (fossils excepted: see Art. 8.5) may be either a specimen or only until 31 December 2006 an illustration. On or after 1 Jan 2007 the type must be a specimen.”

**Wieringa’s Proposal** was **accepted.** [Applause.].

**Haston’s Proposal**

**McNeill** introduced another new proposal from the floor on the topic. He did not know how relevant it still was, but it probably was from the point of view of the second section of Art. 37.4. There was a proposal earlier on of a new Recommendation, in 37A. It was displayed on the board and he read it out: “In cases where it had been technically difficult to preserve materials suitable for a type specimen, every effort should be made to preserve material which may be suitable for additional study, e.g. DNA extraction.” He added that the wording would obviously be modified to conform with what had been passed.

**P. Hoffmann** thought it would only have any effect for six months maximum until illustrations were outlawed and specimens had to be a type, and wondered if that was really worth it?

**McNeill** responded that it obviously applied to the algae and fungi.

**P. Hoffmann** apologized.

**McNeill** assumed it was only applicable to that now.

**Demoulin** thought that with what had been passed it was rather meaningless, but as a general Recommendation, taking out “in cases when it had been technically difficult”, because if it had been technically difficult it most probably was also technically difficult to have it viable for DNA extraction. He said that as it was, it made no sense, but he thought a general Recommendation that with *any* type it would be good to preserve material that was suitable for DNA extraction could be a good thing.

**McNeill** checked that he was not proposing a formal amendment, just commenting.

**Demoulin** was just suggesting that the person who had made that suggestion maybe could change it to a general Recommendation to have material that was suitable for DNA extraction, but he was not going to do it himself.

After a rather tortured process to come to a elegant solution to a problem **Dorr** found this to be a little bit appalling. He asked the Section to **not** open up Pandora’s Box again. and stated that the proposal must specify algae and fungi if talking about DNA extracts.

**Hawksworth** suggested changing “extraction” to “extracts”, that would apply to all groups then, because he thought it was very important that it was taken on board by people that worked with plants as well.

**Tronchet** wondered what the DNA extract would be called: was it an isotype?

**Nicolson** did not have an answer.

**McNeill** explained to Dorr that the wording was drafted and was on the screen while the Section were going through the successive options, and felt that it may well be that it was now irrelevant to the present wording and would need to be so changed as not to be reasonable to consider it further now, but he left that to the Section to decide.

**Nicolson** asked if the Section was ready to vote for the amendment?

**McNeill** corrected him to new Recommendation, adding that “extract” was just a minor change.

**Haston’s Proposal** was **rejected.**

[*Here the record reverts to the actual sequence of even* ts.]

### Article 38

**Prop. A** (65: 32: 26: 20) was ruled as **rejected** because Art. 8 Props A and B were rejected.

### Article 39

**Prop. A** (17: 69: *70: 0).

**McNeill** moved to Art. 39 Prop. A, noting that it was one of the cases in which the Rapporteurs suggested a particular special meaning for an Editorial Committee vote. He explained that the proposal started life associated with the proposal to abandon Latin as a requirement. As a means of making it clear when a new taxon was being described, the author Rapini proposed that the word “nov.” appear in the proposal. What the Rapporteurs had suggested was that this was not appropriate as an Article but might better included as a Recommendation, because they felt that putting another hurdle in the path of valid publication might be unreasonable. They did think that saying clearly that the thing was new was a very desirable. He summarized that there were three choices and they probably should be put that way: vote “yes”, “no”, or as a Recommendation.

**Nigel Taylor** pointed out that such a Recommendation was already embodied in Rec. 32 D.

**McNeill** apologized, he was looking at another proposal by Rapini. He agreed that was absolutely right and it was probably irrelevant and should just be defeated. He suggested that the Section may wish to make it mandatory to have an illustration for all groups. It was currently mandatory for fossil plants and algae. The proposal would make it mandatory for all organisms, after that date.

**Printzen** had doubts about the words “showing essential characters”, noting that in lichens there were many cases where the essential characters were chemical characters that could not possibly be depicted in this fashion. Even worse, there were some cases where the essential characters were, for instance, hymenio[?] pigments. The chemical structure of these pigments was not known and the essential character may be a colour reaction, so the pigment may be green in one chemical and red in another. He felt that it could not possibly be put in an illustration.

**Hawksworth** stated that there was a particular problem with the yeasts where you just had assimilation tests, often even just in a table or a long list of chemicals. He felt it would be unworkable to have a meaningful illustration.

**Veldkamp** added a practical point – his institute could not afford illustrations.

**Gandhi** thought an illustration was preferable, especially when the language was not understood by many botanists. He gave the example that he could not follow German so when the description was given in German, he would not know what the author was describing. Botanical illustrations would be useful compared to the diagnosis but it was cost prohibitive for many people, so he felt they should not be mandatory.

**Prop. A** was **rejected.**

### Article 41

**Prop. A** (61: 85: 6: 1).

**McNeill** moved on to Art. 41 Prop. A and this was a point where the Rapporteurs had erred in their comment. There was a requirement for all names to meet the requirement for valid publication under Art. 33. It was not true, as they had said, that just the ranks and form of names were regulated above the rank of family but they were also required to meet the requirements of valid publication. They were not subject to priority. He did not think that had any particular bearing on the vote but they apologized for their oversight and carelessness.

**Turland** clarified that the proposal did not come in the name of the Committee because it did not receive a sufficient majority vote within the Committee, so it was in the name of the individual members of the Committee who did support it.

**McNeill** explained that the Committee took the view if there was a majority in the Committee, they should try to put that forward, even though it required, under the guidelines, a 60% for it go forward in the name of Committee.

**Turland** added that if it was 60% or greater majority, it was put forward in the name of Committee, if it was a simple majority then it went forward in the name of the individuals and if it was 50% or less then it no proposal was made at all.

**Ahti** thought that the example given must be corrected some way because, in light of Art. 49, suprageneric names had no basionyms and, in addition, it meant that they could not have parenthetical author citations either. He made an addition to Art. 49 “a parenthetical author must not be cited for suprageneric names because such names cannot have basionyms, as defined in Art. 49”. He felt that should be taken into account.

**McNeill** explained that there was a proposal from the floor from Ahti on Art. 49 that would be discussed shortly. He was just making the point under the present wording that he believed that parenthetic author citation was not appropriate here. His proposal was to produce a note to clarify. McNeill felt that it dealt with Art. 41 Prop. B, rather than with Prop. A and Prop. A was the core one.

The way that **Demoulin** saw the problem was that there was a general rule that applied to every kind of taxon, Art. 32.1(c) that any name of a taxon must be accompanied by a description, diagnosis or a reference and defined with conditions, in the case of families and subdivisions of families, genera and subdivisions of genera. The recent proposal would extend, somehow, to taxa above the rank of family. He did not know it was desirable. He wondered why limit the conditions for those taxa which were not linked to priority and thought we would live with what we had.

**Turland** explained that it was one of the proposals that was made by Reveal, to the St. Louis Congress where it was referred to the Special Committee on Suprageneric Names. The concern of the original proposer was that under the wording of the *Code*, a suprafamilial name could theoretically be validated by reference to a previously published description of a forma. He believed the proposal stemmed from a feeling that that was somehow undesirable.

**McNeill** thought the Vice Rapporteur had made the situation very clear and it was really a matter of the Section deciding which way they wanted to go. He summarized the option as to tying it down more clearly as it applied in the case of the ranks of genus and below and ranks of species and below and family and below or cover it throughout all groups.

**Prop. A** was **rejected.**

[*The following discussion occurred after Art. 45 but has been moved here to follow the sequence of the* Code.]

**Prop. B** (98: 32: 18: 1) was referred to the **Editorial Committee.**

**Wieringa** pointed out that in Art. 41, Prop. B had been skipped because A was defeated, but he did not think that B had anything to do with Prop. A because it dealt with the level of the family. So it could be a perfect Example of the present *Code*. He thought it should be dealt with.

**Turland** explained that Art. 41 Prop. B, was the proposed Example regarding *Peganaceae* being validly published by reference to the basionym *Peganoideae*. He started to say that under the current *Code* a family name could not be validated by reference to and then apologized and corrected himself as he had misread it. He was afraid the Rapporteurs were under the impression that it could not be validated because the rank of the name attached to validating earlier description was not at the rank of family or below, but it was at the rank of subfamily so that was possible.

**McNeill** agreed that the Example was perfectly right. He assumed it was an Example of what had just been defeated. It turned out it was just a general Example of what was already in the *Code*. He suggested that the Editorial Committee could look at it and did not think further action was needed. He thanked Wieringa for drawing it to their attention.

**Nicolson** moved to a vote on referring it to the Editorial Committee.

[*Here the record reverts to the actual sequence of events*.]

### Article 45

**Prop. A** (35: 100: 16: 0).

**McNeill** introduced Art. 45 Prop. A as another one that stemmed from abandoning the Latin requirement and putting in another requirement for the valid publication of a new taxon. This was the addition of the phrase nov., e.g.: gen. nov., spec. nov., comb. nov., the term novum or the abbreviation of it to be required on or after 1 Jan 2007 for the valid publication of a new taxon. He felt it could be considered on its own merits, quite independent of the Latin matter, which had been rejected.

As an indexer **Gandhi** preferred such flagging. He remembered an example about 6 years ago when a new species was published without any flagging and then a very brief Latin diagnosis involving two or three characters. It had looked as though the author was deliberately not mentioning that it was a new species and it was only accidentally that they noted that it really was a new species. He felt it would be useful if such flagging was done.

**Watson** thought it was good to hear what the IPNI people had to say about it but he thought, from non-indexer’s point-of-view, but sort of a data-baser’s point-of-view it was very useful to have these things in. He thought they were in as a Recommendation anyway but, going through he could not find them. So he wondered whether or not it was better to put them in the *Code* as a Recommendation rather than a rule.

**Kolterman** noted that it said the term novum or an equivalent, the three examples given were abbreviations of the Latin, but, in the absence of a statement that it had to be in Latin he assumed it could be an equivalent in any modern language as well?

**McNeill** agreed that was correct as it stood at the moment.

**Challis** agreed with Watson’s comments. They thought there already was a Recommendation but could not find it. She did not want it to be necessary for valid publication but thought it would be useful as Recommendation.

**McNeill** asked if she proposed that it be accepted as a Recommendation? [She did and that was seconded.]

**Bhattacharya** thought that there was an orthographic error, as there should be a full stop between “comb” and “nov.” It should be “comb. nov.”.

**McNeill** noted that the amendment was to have the proposed wording treated as a Recommendation instead of an Article. He suggested that the Section could vote on that.

**Funk** proposed that “or an equivalent” be omitted.

**McNeill** pointed out that if it was a Recommendation, it did not matter unless somebody wanted to propose that it be the equivalent or an abbreviation. He clarified that that was an amendment to the amendment. [That was seconded.]

**Watson** added that “must” should also be changed to “should”.

**McNeill** assured the Section that that would be done editorially as part of a Recommendation. He explained that the current wording was that of a rule and there was an amendment to make it Recommendation so the Editorial Committee would make the necessary grammatical changes. There was the other more specific amendment to insist that it be in Latin. He thought it would actually be novum or an abbreviation, rather than an equivalent.

**P. Hoffmann** pointed out that it could be nova or novus which was not an abbreviation and wondered if the Editorial Committee would take care of that?

**Zijlstra** highlighted that the part that was in bold could not be a Recommendation.

**McNeill** clarified that it would be a separate Recommendation, not part of the Article at all and the existing Art. 45 would stay exactly as it was. The part that was an addition, was on or after 1 Jan 2001...

**Nicolson** reiterated that the proposal was to make it a Recommendation and it would become an Editorial Committee matter.

**McNeill** noted that there was first an issue of changing the second amendment, that was the amendment to alter “equivalent” to “abbreviation” and that was what he felt the Section should look at first.

**Demoulin** thought that Zijlstra meant that “should” may be too strong for a Recommendation and maybe it should be something like “it was advisable that...”

**McNeill** pointed out that that was not the amendment to the amendment. He did not think anyone wanted “equivalent”, by the sound of it and suggested voting on that.

**Nicolson** moved to a vote on the basic amendment.

**McNeill** clarified that that was the amendment to use abbreviation instead of equivalent, if you did not want it to be in English, Chinese or Russian.

**Dorr** thought it unwise to make a Recommendation that stated that you were only using an abbreviation. He felt it should have the full word and indicate that an abbreviation was acceptable.

**Nicolson** believed that would be editorial.

**McNeill** asked to please get the first amendment dealt with before talking about further things.

[The **amendment** was **accepted.**]

**Dorr** could find only one comparable Article, Art. 7.11, in which the requirements for designating a lectotype were stated and “typus or an equivalent” were inserted. He guessed it was editorial but imagined that whatever Recommendation you had that the language for using a Latin designation or its equivalent, be parallel throughout the *Code*.

**McNeill** thought that seemed to have gone back to what had just been approved. The whole point, he understood, of the people who wanted the Recommendation was that they wanted it in Latin, whereas in the case of the Art. 7 it could be in any language. That was his understanding of the vote.

**Nic Lughadha** thought it was possibly editorial as well but made a plea to take out the phrase “a direct citation” as she felt that just confused people because it did not specify the direct citation of what. She felt that being followed by the term novum or a phrase including the term novum or its equivalent, or its abbreviation, was fine. She felt it was important it should be in Latin because she thought that, eventually, there would be a move to having machines scanning for new taxa instead of people scanning the literature for new taxa and being a little restrictive in the terminology would help five to ten years down the line.

**Per Magnus Jorgensen** offered a minor linguistic thing. He noted that since we were so happy about the Latin, he pointed out that novum was neuter and it was not appropriate.

**McNeill** stated that it would be clearly put in as “novus, nova, novum” and would have to depend on the gender of the name involved.

**P. Hoffmann** wondered if what Nic Lughadha just said was that an amendment or editorial.

**McNeill** thought that, apart from the change from “equivalent” to “abbreviation”, all the other suggestions he had heard would be editorial. He summarized what was to be voted upon as a Recommendation basically the same as the original Article but phrased as a Recommendation, with the change of abbreviation just accepted and with the corrections because it was just neuter and not any other gender and so forth.

**Peter Jorgensen** thought that if we were going the route that Nic Lughadha was suggesting of having computers scan the literature for finding these abbreviations or their equivalent, then it probably should be a rule.

**McNeill** felt that, in that case, it was perfectly straightforward and he was speaking against the amendment. If the amendment was defeated he explained that discussion would go back to the original motion which was to have the rule.

**Alford** had another editorial matter. He thought that if the proposal was taken up he thought in practice most people used the abbreviations in regard to Art. 35.1 in order to indicate the rank. So most people would put “gen. nov.” to indicate it was a new genus. He suggested somehow putting some sort of cross reference there applying to this information.

**McNeill** moved to a vote on having it as a Recommendation and not as an Article.

[The **amendment** was **accepted.**]

**Prop. A** was **accepted** as amended.

**Prop. B** (85: 34: 20: 6).

**Nicolson** moved onto to Art. 45 Prop. B.

**McNeill** introduced it as a proposal that came from the Committee for Algae. It related to one of the small number of significant differences between the zoological *Code* and the botanical *Code*, dealing with what in the zoological *Code* was called “coordinate status”. It had some implications for algae because what we would call validly published names were accepted both the zoological and the botanical *Codes*. He concluded that their rules had some bearing and this was addressing a particular situation.

**Silva** noted that, according to Art. 45.4, available names under the zoological *Code* (ICZN), if they applied to algae, were automatically acceptable under the botanical *Code*. It had recently been pointed out by Alexander Doweld (Moscow) that the Principle of Coordination in the zoological *Code* created many names that would impact botanical nomenclature. These were names that were created automatically within groups: species group, genus group, and family group. He explained that, for instance, an available name was created for a subspecies, the name for the species was automatically created, whether or not it was used and, if the name for a subgenus was made available and then a generic name was also made available at the same time, whether or not it was used. He reported that the proposal of the Committee for Algae was that we accept only those names that were created and actually used, and not accept those that were not used.

**McNeill** explained that “available” in the zoological *Code* was more or less the equivalent of “validly published” under our *Code*.

**Prop. B** was **accepted.**

[*The following debate, pertaining to a New Proposal on Art. 45 by Demoulin relating to later starting points took place during the Ninth Session on Saturday morning*.]

**Demoulin’s Proposal**

**McNeill** reminded the Section that this provision dealt with organisms that were algae but were previously treated under a different *Code*.

**Demoulin** introduced the proposal on behalf of the Committee for Fungi. The Committee had met along with a few other mycologists earlier in the week, and had discussed several issues. There was some kind of anomaly in Art. 45.4 that never bothered people as long as it was apparently of concern to very few organisms. This was the provision that a taxon belonging to the algae which was treated under another *Code* need only meet the requirements of the pertinent other *Code* to achieve status equivalent to valid publication under the botanical *Code*. As the Section would recall from the discussions on illustrations earlier in the week, there was not much difference between situations in the algae and fungi, and there were the same kinds of problems in the two groups, though it was more difficult to cultivate algae. That nobody ever proposed to treat fungi as well as algae here, was because nobody ever supposed there would be more than a few cases. He remembered that it had been difficult to find the Example of *Labyrinthulodyction* given in the *Code* to show that this provision did not concern fungi. The situation had now changed dramatically, as in recent years it has become absolutely convincing through molecular data that some organisms which were considered protozoa were derived from and included within the fungi by some researchers dealing with them. The first case arose with *Pneumocystis*, which medically was an extremely important genus. A more recent case was with the *Microsporidia*, a group with an enormous number of names on which Redhead would speak. Those names now needed to be protected, and a very straightforward way of doing that was to do the same as had been done for dinoflagellates and blue-green algae, just to make a small addition in Art. 45.4, that in the two places where algae are mentioned to add “and fungi”; in the second and the fifth line. Redhead would explain why this was urgent, and this is why the Committee had decided to raise it from the floor.

**Nicolson** felt Demoulin had been so convincing he was not sure that the Section needed further documentation and could vote.

**Redhead** said he would hate it to be rejected without having heard the arguments. It had come to light molecularly that a major group, the microsporidians, intracellular parasites with no mitochondria belonged in the kingdom *Fungi*. There were over 1000 species known and there are predicted to be thousands more, and 150 genera all of which have been considered as protozoans since 1922. Those described before 1935 might have valid names, but almost everything else after that was described without Latin. Molecularly, not using mtDNA as there are no mitochondria, but using nucDNA, it had been shown that these were a group of *Fungi*. Researchers that worked with them had become convinced they were *Fungi*, and then the sudden realization set in, after the euphoria of discovering where their organisms went phylogenetically, that “Oh my goodness they are now covered by the botanical *Code*”. The names were almost all invalid, and it would be a horrible mess to fix that if provisions were not made to save the group. The Committee had considered sticking them in as an exception in the Preamble, where it stated what the *Code* covered, and add “microsporidians” there, but a more eloquent way to cover it was to stick in “and fungi” in the two places in Art. 45.4. Then it would not only cover and save the microsporidians, but it would simplify things for other groups. One of those was the genus *Pneumocystis*, causal agent of a pneumonia primarily in AIDS victims. He had a poster based on a paper he had submitted which had been a horrible, horrible, mess nomenclaturally to clean up. It would be affected by putting this addition into Art. 45.4, and basically he would have to retract his paper, which he would be quite willing to do because it simplified matters immensely. Otherwise the date of validation would have to be changed for yet another medically important organism. *Microsporidia* were medically important in causing a wasting disease in humans and affecting virtually every single phylum of animals from bryozoans and other protozoans through to mammals. The Committee also anticipated other cases, and John David had mentioned another group that molecularly was coming up through the ranks and may prove to be fungal. In one fell swoop by adding in “and fungi” the *Code* could cover these situations. This would only be for organisms that were presumed to be treated by another *Code*. What was not intended was that it refer to all fungi under all circumstances, even those considered as treated under the botanical *Code*, so waiving the requirement for Latin; that would create a backlash of validations of many currently invalidated fungal names.

**Hawksworth** proposed a friendly amendment, to delete Ex. 6.

**Redhead** suggested it could be changed so that it would be valid rather than invalid.

**Hawksworth** amended his friendly amendment to “editorially change Ex. 6”.

[The **friendly amendment** was **accepted.**]

**McNeill** thought the argument had been made very convincingly, but stressed that there should not be the assumption in anyone’s mind that the phylogenetic position of a group of organisms determined the *Code* under which it fells That was an issue of what was going to be most stable. He had originally suggested to the proposers that if people working on *Microsporidia* wanted to continue to work under the zoological *Code* under which they had always operated, then the simple thing was to put this into the Preamble, where it was indicated what was covered by the botanical *Code*; that it did include prokaryotes such as blue-green algae, and also fungi which were not plants. This would make it clear that the *Code* did not cover that group. He made this point not because he wanted to oppose the proposal, as the arguments were very clear and it did affect other areas, but he wanted to avoid the false assumption that just because it was suddenly scientifically discovered that a particular group of organisms was more related to another, that somehow it had to go into a different *Code*. Nomenclature was an arbitrary mechanism, a set of rules to determine the right name for organisms. It was perfectly possible to continue to treat *Microsporidia* under the zoological *Code*, if that were the wish of those that worked on them. It turned out that inclusion in the Preamble was not the best way in this case. He just wanted to stress that the *Codes* were not phylogenetically based.

**Gams** remarked that if the Section adopted the Art. 45 solution, the consequence would be that all subsequently discovered *Microsporidia* would require a Latin diagnosis, while if it adopted the Preamble solution that would not be the case.

**McNeill** indicated that was his understanding of the Article as well, but understood that was not everyone’s understanding.

**Demoulin** explained that there was a long experience of working with Art. 45.4 in the algae, where the major groups of concern were dinoflagellates and blue-green algae. He felt that great attention must be paid to the wording. The first line, “If a taxon originally assigned to a group not covered by this *Code*”, meant that groups that had always been covered by this *Code* are not to be taken into consideration here. There was no point in saying that in future no Latin was needed. His other point was, “If the taxon is treated... ”. This did not rule on how and why something should be treated. As McNeill rightly said, the Section should not have phylogeny deciding. What counted was what people said and were willing to do, and in groups like this there would be people who wanted to continue using the zoological *Code* and not to shift to the botanical *Code*, just as some of those working with dinoflagellates still use the zoological *Code* and others use the botanical *Code*. The Section should make it as easy as possible to transfer names from one category of users to another. He really did not see any problem, as the Section would not be ruling that only one *Code* should be used.

**McNeill** accepted Demoulin’s point that it was worded that way, and agreed.

**Demoulin’s Proposal** was **accepted.**

[*Here the record reverts to the actual sequence of events*.]

### Recommendation 45A

**Prop. A** (124: 20: 10: 0).

**McNeill** moved on to Rec. 45A which was a proposal to delete a Recommendation on the grounds that it was now redundant and inappropriate.

**Rijckevorsel** had recently properly looked at the proposal and was afraid it was quite inaccurate. His problems were that firstly it stated that it came in in 1912 while it came in in 1906. More seriously, when it stated what the Recommendation concerned, it was incorrect, it concerned works in a modern language, which certainly in the phrasing of a century ago, meant works of a popular nature. It mentioned catalogues. Thirdly it stated that, in connection with valid publication, and valid publication, as now defined, came in in 1935 in the Cambridge *Code*. The Cambridge *Code* took quite note of this and altered Recommendations so as to comply with the then new provisions on valid publications, which remained unchanged until now. He had looked a little closer at the Recommendation and originally it was paired with another Recommendation on unpublished names, which was now Rec. 34A. Actually it was sensible Recommendation which had been in the *Code* for 100 years, constantly adjusted over time and he thought it should stay in.

**Wieringa** thought it should go out because it introduced an ambiguous statement. Now it only recommended something that should be done anyway. He acknowledged that it was a Recommendation and Recommendations meant you did not have to comply. He thought that people may argue, when writing a flora, that you did not have to have to comply with requirements for valid publication and still have it validated.

**Rijckevorsel** thought it was actually quite an ambiguous Recommendation. He thought the basic situation would be a publisher asking a botanist to write a book and put in his new taxa but leave out all the technical stuff, the Latin and the expensive figures, so as to keep the cost down and to raise the appeal to the general public. The botanist was advised that this was unwise because it could lead to, firstly taxa that were being described without getting a name formally, and secondly being introduced into unpublished names. He suggested that perhaps the placement could be changed.

**P. Hoffmann** pointed out that any published name at any time needed to conform to a firm set of rules and they must be obeyed or it was not validly published and no Recommendation did anything to it. She thought it should be voted down and it was waste of time to discuss it.

**McNeill** felt that, in so far as it had any conceivable meaning, it would be that instead of publishing your new names, before you get your Flora out, say in Novon, you must publish them in the Flora. Otherwise it had no meaning. He did not think the Section would want to recommend that. He knew that the Flora USSR did this [with valid publication in Appendices] but it was not the only model. It was perfectly reasonable and probably much better to publish names ahead of time for a medium in which Latin was not used. He saw no purpose for keeping it.

**Prop. A** was **accepted.**

### Article 46

**Prop. A** (16: 35: 98: 0).

**McNeill** introduced Art. 46 Prop. A as a proposal that corrected an existing Example, but in a way more concise than the original proposer presented it. He thought it should be passed and referred to the Editorial Committee. The author of proposal suggested that the Example was wrong and, if that was the case, the Editorial Committee certainly should correct it.

**Prop. A** was referred to the **Editorial Committee.**

**Prop. B** (107: 21: 25: 0).

**McNeill** explained that Art. 46 Prop. B was to correct an existing Example, so it was rather similar, and might go to the Editorial Committee. He noted that it was strongly supported.

**Demoulin** thought it may be strongly supported but felt it was not adequate to do this because all the additional, but correct information had nothing to do with the what was illustrated. He thought it was much clearer to retain the Example as it was with just the part of the story that illustrated the Article.

**Zijlstra** suggested that maybe it could be made shorter but anyhow it should be changed. The concept that now was in the Example was “ascription by implication” and she argued that that was not something that was covered by Art. 46.3

**McNeill** assured the Section that the Editorial Committee would work hard to make it as concise as possible aided by the remarks of Demoulin.

**Prop. B** was **accepted.**

**Prop. C** (104: 20: 29: 0).

**McNeill** noted that Art. 46 Prop. C was an Example in the same area, again proposed by Zijlstra & al.

**Gandhi** was sure that the Rapporteur and others would remember that it was a group discussion abou this Example of *Claytonia lanceolata.* As stated in the Example, in Pursh 1831 no name was directly associated with any authorship, only at the end of the description was a reference made to the previous author, Linnaeus or a manuscript author. So in this particular Example at end of the description none was cited. So, he elucidated that the question was whether it was an ex author or there was no ex authorship. In a group discussion in his herbarium they all decided that it should be an ex authorship because that was the procedure Pursh followed, not associating any binomial with any author.

**Nicolson...** asked whether there was a description but not the name.

**Gandhi** replied that that was his [Pursh’s] procedure. He explained again that at the end of every description a reference was made to published publications, because he did not associate any binomial in that work. He suggested that if it was necessary they could produce a photocopy of the particular page and see exactly what was being talked about.

**Nicolson** asked if he was saying that the Example was in error? [No recorded response.] He thought it could definitely be handled in the Editorial Committee rather than on the floor. They would look at the original and be sure it was as advertised.

**Wiersema** felt it was accurate. As far as the original publication, he added that there was no ascription of any names by Pursh in this work. The description or diagnosis was ascribed to Pallas. The question was, without an ascription of a name, direct association, which was the definition of ascription, with the name of the author and the name, how to determine the authorship? He felt it had implications regarding typification. He felt that if Pallas was considered to be the author of the name then the type came from material associated with Pallas. If Pursh was the author of the name then the type came from material associated with Pursh. He argued that it was an important distinction. He noted that there were other works, for instance, *Species Plantarum*, where there was no ascription of authorship anywhere associated with names, but there were many cases where the diagnosis was attributed to someone else. He did not want to have to treat the authorship of those names the same as the author of the diagnosis, so it would seem to be the standard procedure that had been followed.

**McNeill** wished to clarify that he was pointing out that the proposal was, in fact, in accordance with the definition of ascription.

**Wiersema** agreed.

**Gandhi** wanted to address what Wiersema said. They did not just go by the Pallas name alone, but included whatever was cited within the protologue. He did not believe just a single type was involved.

**Brummitt** had some doubts about the proposal. He remembered discussing it with Turland some months ago. When a name was ascribed was not clear if it appeared at the beginning of a paragraph and the ascription was at the end, after the description, was the name also included? He argued that it depended, to some extent, on the format of the book. He felt there were complications in all this and was just a little nervous about accepting these Examples without looking further at it. With all respect to Zijlstra, whose work he valued greatly, he wondered if it may not lead to a little bit of trouble.

**Lack** commented that he had recently published three papers on the issue in the Example. It was definitely more complex than stated in the proposal. He suggested that it be considered by the Editorial Committee how to word it because it was definitely much more complex, i.e. the Humboldt, Willdenow & Schultes business.

**McNeill** reiterated that Examples referred to the Editorial Committee, except voted Examples, were looked at critically, because, if it was not, in fact, an accurate reflection of the *Code*, if there was an ascription there, even though the author of the Example said it was not there they would not use the Example or use it in a different direction.

**Schafer** also considered both Examples most unfortunate.

**Zijlstra** reported that several years ago Wiersema, Reveal, Gandhi and herself had extensive discussions. At last three of them arrived at the conclusion that this was the interpretation in accordance with the *Code*, Art. 46. She explained that one of the cogent points that helped them was concerning the names of 1753. She understood Brummitt’s comment that the format of the book was important but that was in such a way that there was no ascription of species names, then simply, that was the situation. She argued that if the ascription of the description to constitute ascription of name as well, one would have to say that many Linnaean names of 1753 were by author X in L., 1753.

**McNeill** gave the assurance that the Editorial Committee would look very carefully at that and, if necessary, consult with those who were active in indexing and so forth who had expressed concerns. He suggested that to move it forward in a positive manner the Examples be referred to the Editorial Committee for inclusion as further examination determined.

**Prop. C** was referred to the **Editorial Committee.**

## Sixth Session

Thursday, 14 July 2005, 14:00–18:00

### Article 46 (continued)

**Prop. D** (30: 23: 99*: 0).

**Nicolson** thought the Section had been looking forward to this.

**McNeill** introduced Art. 46, Prop. D, a proposal for which there was special meaning for Editorial Committee. [This was not noted with an asterisk in Taxon 54: 1061.] In this case the vote was 34 for, 23 against and 99 Editorial Committee. The Rapporteurs suggested that parts of the proposal were already in the *Code* and that it could be covered more readily by a note, incorporating one part that was less than obvious.

**Brummitt** did not care how the wording appeared so long as it did appear. He felt that whether it was an Article or a Note was irrelevant. He knew that it was possible to argue the position from the existing *Code* but it was very hard for most users. He was anxious to make it clear to people using the *Code* how it operated. The proposal covered the question that he was asked most often about citations. He thought that the wording he had suggested made it absolutely clear. If it was passed to the Editorial Committee that was fine with him but he just wanted to say that identical wording was passed to the Editorial Committee at the Tokyo Congress and that it never got in to the *Code*. He hoped that they would actually put it in.

**McNeill** assured him that if it went to the Editorial Committee they would definitely put the wording in that appeared in the Rapporteurs’ suggestion, which was the first part of Brummitt’s suggested wording because the second part became self-evident. He added that if it seemed not to be obvious, they would make sure that it was made clear. He felt that the point behind the proposal was perfectly sound and reflected quite clearly what the Article said but it did need a Note. He was unhappy about it being another Article because it seemed to him to just repeat what it had already said before. He suggested that if it was referred to the Editorial Committee and the proposer was agreeable, that would move the matter forward well.

**P. Wilson** offered a general comment in response to McNeill’s. He thought that cutting out the last sentence would not be terribly helpful as he had often found with the *Code* that he and others had problems because things that were self-evident to some guru were not self-evident to the rest of the world.

**McNeill** acknowledged that point. He thought that the particular clause applied much more broadly than in the particular case and could probably be included elsewhere as a Note, possibly attached to another portion of Art. 46. He was not certain exactly where but it struck him as so self-evident, but he thought it should go in if it was not self-evident to everybody.

**Gandhi** suggested that the proposed Example was similar to or identical to what was already given in the *Code* Art. 46 Ex. 11?

**McNeill** thought it was slightly different and felt that the Example was worthwhile and did not duplicate anything.

**Schafer** would be happy to vote “yes” to the proposal as it was or refer it to the Editorial Committee.

**Ahti** was very glad to see the proposal because he had been trying to get the idea through and usually nobody had understood it. He found it a very difficult case, which was not clear from the *Code*. He really hoped it could be included in the *Code*.

**McNeill** thought it could be assumed that the Editorial Committee would make sure that the wording of the *Code* fully supported the Example.

**Prop. D** was referred to the **Editorial Committee.**

**Prop. E** (5: 139: 7: 0) was ruled as **rejected.**

**Prop. F** (115: 19: 19: 0).

**McNeill** noted that Art. 46 Prop. F was a proposal for some Examples made by Turland that clarified what was meant by “author of a name”.

**K. Wilson** had some problems with the proposal, as he had said to the proposer beforehand. He suspected that for a lot of people trying to define what a publication was, was not clear, so that if it were passed the Editorial Committee would have to look carefully, because there were so many publications within publications. What was, to her, a more serious matter was that it seemed that it would change radically how people published species. She knew quite a few cases where a new species was described by one person, say Smith, and it was in a publication that is by Smith, Jones and Brown. In other words there were three authors for the whole paper in a journal. She suspected that that was where it differed from what happened in floras, but the principle was the same and she saw no reason why the current practice should change which would be Smith in & al. In terms of citation she felt there was no way it should be ex or any other citation, but she thought that the proposal and the Examples given would end up having that effect unless the section of the publication, relevant to the part in which the name appeared was defined as that single species treatment. In which case you could say that they were a single author. She wanted to hear some other comments where people saw the same problem that he did.

**Turland** responded that for a paper in a journal or an account in a Flora, publication would be defined as the paper or the Flora account and that part would have its author or authors. If the author of name were different from all the authors of the publication he explained that it would be “that author ex... ” or “that author or those authors in”. Although he had seen it done, in the case of a paper in a journal you would not say “Smith in Jones in Taxon” and then a reference.

**McNeill** added that the issue arose when the description was not attributed, which may be overlooked. He felt that was the point. Under Art. 46.2, provided that you ascribe the name and the description, it really did not matter whether that was an author of the paper or not; in the same way when it came to a new combination or a nomen novum this must be ascribed to authors when it was explicitly stated that they contributed in some way, which covered somebody having a chapter heading and also whether at least one author was common to both. He explained that this was a situation where the name was attributed to someone but the description was not, the description was that of the author of the publication. It was defining the publication a little more narrowly than the whole of the Flora of China, for example.

**Buck** had been sent material and asked to describe a new species, he sent them a name, a description and everything but his name was not on the Article. He made it clear when he wrote that they had to put in the acknowledgements of their Article that he actually supplied that information, because he knew that if they did not it would become “Buck ex whoever did it” and his name was potentially just dropped, even if the holotype was in his herbarium.

**McNeill** felt that Buck’s description of the situation was accurate, but they did just have to do that, provide the acknowledgement. He added that they did not even need to do that if they attributed the description to him, as well, as long as both the name and the description was attributed.

**Buck** noted that it usually just ended up saying “Buck sp. nov.” and then there was a description. He did not write his name at the end again, that he wrote two things!

**McNeill** stated that, unfortunately, that was what the *Code* said. He suggested they could always say “The following new species was provided to us by Dr. Buck.” and that would be quite enough.

**Nee** thought that maybe it was his lack of English or maybe he just did not understand. He had been reading it and thought that perhaps a change needed to be made, because “authorship of that part of a publication in which a name appears” was not clear whether it was talking about the author of the publication or a name of the new taxon that appeared. He thought it may be more clear when it was put in context but, as it was, he did not really know what “name” applied to.

**Turland** clarified that it was the name of the taxon.

**Marhold** hypothesized that the author of the publication was person A, then the name was attributed to persons A and B. Let us say persons A and B, together, wrote the description. He wondered if the person who was not the author of the whole paper should be dropped?

**Turland** responded that that was already covered by the current wording of Art. 46 so it would be “A & B in A”.

**McNeill** added that it must be accepted as ascribed when at least one author was common to both.

**Wieringa** thought that Ex. 20*quater*, as was proposed, *Disporum ternstroemioides*, even including this new proposed Note 1*bis*, was not in accordance with the *Code*, because now 1*bis* only clarified what the authorship of the publication was. But in Art. 46.2 the last sentence was about what the authorship was, but before that there was a line “a new combination or nomen novum must be attributed to the author or authors to whom it was ascribed when, in the publication in which it appears, it is explicitly stated that they contributed in some way to that publication.” And being an editor of a flora in which this name was ascribed meant that Wu did contribute in some way and the ascription of the name to Wu alone would still be valid and so he felt it was a bad Example.

**Bhattacharya** noted that a similar situation arose in *Naringi crenulata* (Roxb.) Nicolson (*Rutaceae*) [=*Feronia crenulata* Roxb. 1832]. Nicolson made the comb. nov. but confusion prevailed, as it was edited by Prof. Saldanha in his “Flora of the had san District”, Karnataka, India (1976). This proposal would solve the problem. Gandhi was also associated with that work. It could be cited as a typical Example in ICBN 2006.

**Lack** wished to support the proposal because he was familiar with the situation, in particular in the Flora of Iran with Rechinger as the principal editor and then a subeditor, and then author of the genus and then attribution to a fourth person. He felt it was very appropriate that there was a line on how to deal with these problems.

**McNeill** wanted to make the point before the vote that these were not voted Examples, just Examples.

**Prop. F** was **accepted.**

**Prop. G** (118: 8: 16: 7).

**McNeill** moved on to Art. 46 Prop. G, which was a proposal from Silva and related to the parenthetical citation of pre-starting point authors and was included with his proposal relating to changing of the starting point for algae but was not necessarily linked to that it in any way and could be considered separately.

**Demoulin** reiterated that the proposal had been introduced with the one that had been withdrawn and he was quite surprised it had not also been withdrawn. This was because, although it was true that it could be discussed independently, if the proposal to delete the later starting point had been accepted, this proposal would have been rather innocuous. He argued that since the other proposal had been withdrawn, this proposal was, in his opinion, extremely inconvenient for people working with later starting point, like for many groups of algae, the very late starting point, the end of 19th century. He noted that the deletion was a reversal of the decision of the Berlin Congress which accepted the sentence and the Example, based on a publication in *Taxon*, with all the arguments he did not have to repeat and he felt the Section could not reverse such a well-discussed decision so easily. He thought that L. Hoffman should explain what the position of the Committee for Algae had been, who had been against the proposal, because maybe people had been influenced by the Committee’s position but this was a matter of “may”. He felt that it was only giving the possibility to some of the people working with organisms with a later starting point, to have a system that allowed tracing as correctly and accurately as possible the origin of a name. He repeated for a group *Nostocaceae*, all the names from the 19th century algological literature were concerned. He felt that it had nothing to do with if the bryologists did not want to use the system, the phycologists did not want to oblige them to do so. He added that, even if, among the phycologists, for example, the desmid people, did not want to use the possibility, nobody would force them to do it. But he felt it would be extremely unfair if the desmid or the palaeontologists obliged the group for which it was felt to be very useful provision.

**McNeill** noted that in the Rapporteurs’ comments in the Synopsis that the Committee for Bryophyta had responded and that the Committee for Algae had not, but had now. He invited Hoffmann to tell the Section how the Committee voted.

**L. Hoffmann** elaborated that, as the previous speaker had said, the proposal was not supported by the Committee for Algae with two votes for it and nine votes against it. He also noted that it was not mandatory, so people were free to use it or not. He felt it was certainly useful, especially for the blue-green algae with a later starting point, to find the original place of publication of a taxon that was validated after the starting point. He added that if you had the mention of the first author included in the full citation it was, of course, easier to find the original place of publication if you wanted to go back to the diagnosis, in many cases. He concluded that it was not supported by the Committee for Algae.

**McNeill** thought that the only other Committee involved with a group which had a later starting date, was the Committee for Fossil Plants which, he thought, was divided.

**Skog** agreed that the Committee for Fossil Plants was divided. She reported that those people who used it were mostly people who were doing databases and tracking names. The rest said that, since it was not mandatory to do, they did not have any strong opinion. She would say that were some members of the fossil plants community that did find it useful.

**Turland** pointed out that there was another issue that became relevant after these sessions. Now there was a starting date for suprageneric names of 1789. He thought that some members of the Section may feel that it was something in favour of supporting this proposal because you could have, for example “Durand ex Jussieu” for the authorship for a family name when the same name had been published prior to 1789 by another author.

**Silva** felt that the first sentence of Art. 46.5 gave all the leeway needed to dredge up the pre-starting point nomenclature which was, obviously, invalid. He continued that if we insisted on dredging up the pre-starting point nomenclature, he believed the first sentence took care of it but the second sentence resulted in a very awkward situation. He suggested that if you looked at the Example, it showed that it may be expressed as *Hypocodium glutinosum* (C. Agardh) ex Gomont. He pointed out that in all other binomials when they were a combination, the parenthetic author referred to the basionym and then the combining author, but here there was no combining author.

**Demoulin** was sorry that the Section had to start the discussion again because the discussion had been had in Berlin. He felt it was done with enormous experience with the later starting point that existed at that time with the fungi and he reported that a lot of people had used that system in the fungi and as long as there were such later starting points it was a useful thing to have. He repeated that people who had a 1789 starting point with suprageneric names had no need nor obligation and it did not concern them. He reiterated that it was specifically for groups with a very late starting point and a lot of specific epithets and felt that it worked well. Some people in the fossil group had found it useful. He reported that before the later starting point was removed, it was found useful by a large number of mycologists, so there was a long tradition of doing it. He acknowledged that it may look queer to some people but it was useful to several people. He was not going to take away a tool for having accurate nomenclature because he found it awkward.

**Zijlstra** was in favour of the proposal. She had asked a few palaeobotanists in Utrecht about their opinion and they said “Hmm, what a curious thing was being permitted in the *Code*. What should we do with this?” What she wondered was why all groups with later starting points should not simply do it in the same way, as “Tournefort ex Linnaeus”. Why should you have such an awkward looking thing? They never used it. She was also asked to ask the Committee [on Bryophyta] on the particular phrase. She did not realize that it existed and had never met it in practice which she felt was the problem.

**McNeill** asked a question of Demoulin and others, who supported retention of it. He wondered why it was so important to refer back to what was almost a basionym, when you had to remember that Art. 7.5 was very specific about this; it said “The type of name of a taxon assigned to group with a nomenclatural later starting later than 1753 is to be determined in accordance with the indication of descriptive and other matter accompanying its valid publication.” He felt that what the earlier pre-starting point author may have done in including this epithet in another genus was really not immediately relevant for typification unless it was clearly cited in the work that was post-starting point. He noted that it was not possible to recombine a name from pre-starting point. It may be that there was still a use for it but it did strike him as little surprising.

**Hawksworth** responded that it was because usually typification was through that author because there was no material and it was nearly always the material, in mycology, of the original author.

**McNeill** felt that if there was material, at that time, the previous author could be totally right and if the previous author was cited and his material was cited then, of course, that was part of the original material but it was only part of it.

**Hawksworth** agreed, adding that it was the common practice, though.

**McNeill** thought it was very different from citing a basionym where it was perfectly clear that what the combining author had in his hand was totally unimportant. It was only what the author of the basionym had in hand. In the case of a later starting point, it was what the author after the later starting point referred to or had in hand that mattered, not what the original pre-starting point author of binary designation, which was not a name, happened to have.

**Demoulin** was really, really very sorry to have to come back to this again. One of the reasons here was that it was not a matter of going to the type, it was having the connection to the whole 19th century literature and to avoid people being confused because they may see the same name with different authors. He continued that they were different authors because either they were using the 19th century literature or they were using Silva publications, who had not been using the later starting point system. He felt it was just a way to give information to people and also a way to easily transform the system if you suppressed the later starting point, as was done in mycology. After that he would not say anything more, but felt it extremely unfair for people in other groups, whether it was the fossils, to present something which was a “may”, not a must. He entreated people who were not concerned, who were not interested, to leave the others in peace.

**McNeill** appreciated that was a “may” and that was probably why there was an issue. He was delighted to find Demoulin so passionate about something other than orthography! But, seriously, he did not think his question had been answered, why was the first sentence not sufficient?

**Demoulin** thought that one of the most important things was to make the connection between the literature, which had been using the later starting system, or not. If the name had changed it was important that…

**McNeill** stated that the name had not changed because there never was a name, there was a binary designation that was not identical.

**Demoulin** felt that it allowed people to understand that the *Lyngbya* and the *Hypocodium* all went back to the same thing.

**Nicolson** pointed out that there was a very strong “yes” mail vote and the Section had heard some very strong objections. He moved to a vote and deemed it to be very close. He asked for a show of cards. He thought it failed.

**Demoulin** was adamant that the mail vote should not be taken as an indication. He was on the verge of leaving he was so disappointed. He requested a card vote.

**McNeill** explained to Demoulin that that was out of order as the matter had already been voted and the proposal was defeated. He added “You won!”

**Prop. G** was **rejected.**

### Recommendation 46E (new)

**Prop. A** (22: 130: 1: 0) and **B** (20: 130: 3: 0) were ruled as **rejected.**

### Article 49

**Ahti’s Proposal**

**McNeill** chose at this point in the sequence to take a proposal from the floor from Ahti regarding Art. 49.1 because it had been discussed or mentioned once or twice already.

**Ahti** felt that there was a lot of confusion about the use of parenthetical authors in suprageneric names where some people thought it was all right and were using them and some others did not accept them. He referred to Art. 49 mentioning only generic names and below, so argued that actually suprageneric names had no basionyms as defined by that Article so it was not possible to make so called combinations and transfers either, using parenthetical authors. He added that the Editorial Committee may decide if a reference to Art. 33 was useful.

**Nicolson** wondered if he understood correctly that Art. 49 now spoke of a genus or taxon of lower rank and Ahti was now introducing a taxa of higher rank that they must have...

**McNeill** disagreed and felt he was pointing out that the *Code* did not provide for basionyms at the ranks above genus.

**Barrie** thought it would be a very useful Note because there was a confusion about where parenthetical authorships were used. He explained that what happened at the level being talked about was that people described a higher rank taxon by referring to a lower ranked taxon but they also used both names simultaneously, for example, *Ranunculales* with *Ranuculaceae* under it. He added that you do not lose that lower rank taxon, so it was a confusion of the use of the parenthetic authorship to include it in that situation.

**David** had two points. First, it was not clear to him that Art. 49 actually ruled against higher taxa. It just merely gave the conditions relating to taxa at the level of genus or below. He felt it did not actually make any statement forbidding that for taxa at higher than the genus. The second point was that, certainly at family level, he felt that combinations were made with a reference to a valid description somewhere else at another level. He thought that if you passed this particular provision it would actually inadvertently make certain combinations invalid.

**McNeill** did not think there was any danger of that because they were covered by Art. 41.1, so if there was a description there did not need to be a basionym but it did have a bearing on how that name should be cited and so forth.

**Turland** referred the Section to the *Code’s* definition of a combination in Art. 6.7 which said “the name of a taxon below the rank of genus, consisting of the name of a genus combined with one or two epithets, is termed a combination”. He noted that they had to be below the rank of genus. The way the word basionym was used in the *Code*, it appeared in Art. 33.3 and Art. 49.1, and was defined as name or epithet-bringing synonym or a name or epithet-bringing legitimate name, two slightly different definitions. He felt that was worth taking into account in this context. He noted that, really, suprageneric names were not combinations and did not have basionyms.

**Redhead** asked if parentheses only indicated a new combination? He wondered what indicated a new status, when the status was changed?

**McNeill** replied normally just “stat. nov.”, and the new author’s name, adding that there was no parenthetical citation of a previous author for “stat. nov.”

That had never been clear to **Redhead.** He had always seen stat. nov. attributed to the earlier author at the other level, whatever it was, up or down.

**Turland** thought the only occasion where there was a name that was not a combination where a parenthetic author was cited was with a generic name where the basionym was an infrageneric name.

**McNeill** maintained that the *Code* was quite clear about a generic name being able to have a basionym. That was specifically covered.

**Redhead** thought that everything they were saying was undoubtedly true, but he still got a really uneasy feeling that all the repercussions and ramifications had not been thought through.

**McNeill** thought it was interesting to have it on the table and he hoped a decision would be taken on it because it was indeed a Note and it did reflect what the *Code* said. He acknowledged that, of course, there had been quite extensive usage that had been different.

**Delwiche** thought that his objection to the Article as currently worded involved the word “must”. He would rather see it say “parenthetical authors need not be cited for suprageneric names”. The reason that he felt that way was that it was very common usage for higher level taxa to provide a parenthetic author as sort of an abbreviation for saying “sensu author”, so you often wanted to be able to cite a higher level taxon and then specify in whose sense you were using that name. If the word “must” was in there then he felt it really stated that it was never appropriate to put a parenthetic author after a higher level taxon.

**McNeill** advised him that if he were proposing that as an amendment he would also have propose it as a new Article as it would not be a Note as that was not in accord with the *Code* at the moment.

**Delwiche** asked for clarification that, in the present *Code*, one may never, in the course of running text, state an author following a higher level taxon.

**McNeill** responded that that was what the *Code* wording actually said, although it was not always practiced. On the other hand, there was something that Delwiche had said, if he understood it correctly, that would never be appropriate for a parenthetic author citation, and that was a misidentification, citation of a usage that was not that of the type. He thought that would be very strange.

**Schafer** wanted to know what would happen if the *Code* said that a parenthetic author must not be cited for a suprageneric name and then somebody cited it. Would the name be lost or the citation just be ignored?

**McNeill** replied that it would be the latter as the Article was not one of the requirements for valid publication.

**Kolterman** certainly trusted that was what the *Code* said, but guessed the reason that this proposal confused him was because Art. 41 Prop. B, which had been referred to Editorial Committee, had *Peganaceae* (Engler) and then talked about reference to the basionym *Peganoideae*.

**McNeill** agreed that there were defects in the wording, which he did not want to start talking about until discussion on the proposal because if it were amended in some way, it might be reinstated.

**Turland** answered the previous speaker by saying that, as the *Code* currently stood, and not assuming any outcome of the proposal currently on the screen, the Editorial Committee would deal with any defects in the wording of that Example that was approved earlier on. He also drew the Section’s attention to the complete absence of parenthetic author citations for suprageneric names in the *St. Louis Code*, even names validated by reference to the description or diagnosis of an earlier name or, in some cases, just an earlier name itself, in other words a transfer from an earlier name.

**Buck** was basically going to volunteer stupidity here. He had read Art. 49.1 five times and saw nowhere that it mentioned anything about suprageneric names. He noted that it said, “cannot have basionyms as defined in Art. 49.1”. He thought that 49.1 had no reference to suprageneric names. And then he looked at Art. 33.3 and saw nothing that gave him any indication it was. So that it seems to him that if there was a subfamily that had been described and somebody raised it to family, he had not yet found where he was told that it was not a combination.

**McNeill** said it was not a combination, and that was definite.

**Buck** disagreed, it said it could be called a combination. He felt that that did not mean that other things could not be called a combination. He wanted to believe. He did not want to have faith.

**McNeill** assured him that a combination was defined in the *Code* and it applied to names of subdivisions of genera, names of species, and names of...

**Buck** interrupted to say that where he had been told to look, it said may be or was called a combination. It did not say other things could not be [a combination]. There was nowhere that had been told to him that higher things were not called combinations. He wanted McNeill to tell him. He did not want to take it on faith.

**McNeill** concluded that a glossary was needed. He referred to the definition in Art. 33.3 of a basionym as a name-bringing or epithet-bringing synonym. He argued that neither case applied. There were no questions of epithets for higher categories and the only case where a name could be brought was at the rank of genus. He explained that it was different name, with a different ending for one thing and a basionym was not stem-bringing, it was name-bringing.

**Gandhi** believed it a useful Article. For those who used the suprageneric name index by Jim Reveal he thought they might have seen that most suprageneric names did not have any parenthetic author citation. He acknowledged that a few did and it may have caused confusion among some. He felt that the new Article would definitely clarify the situation. He believed it should be included in the new *Code*.

**Gereau** wished to clarify that combination was defined in Art. 6.7 as the name of a taxon below the rank of genus etc.

**Orchard** appreciated that the statement reflected what was in the *Code* at the moment, but he also took note of the Rapporteurs’ comments that in practice this was not followed. He wondered why it was needed? Was it doing any harm to put the parenthetic authors in? He favoured, for that reason, adding “need” rather than “must”.

**Zijlstra** did not think it was relevant that suprageneric names were [not] combinations. She thought the argument for the proposal was wrong as Art. 49.1 was about names in lower ranks, so it did not concern a basionym in that sense. She thought it still could be considered to be a basionym for a suprageneric name. Nevertheless she felt sympathy for the proposal and preferred to just delete the second phrase, “because etc.”

**McNeill** thought that what she said about Art. 49 was true but that Art. 33 was quite clear in its definition.

**Barrie** pointed out that currently the proposal read “parenthetical authors need not be cited”. He wanted to know if the change to “must” had been accepted?

**McNeill** noted that until there was a formal amendment and that had been seconded, they kept the original proposal on the board.

**Moore** thought the Section was getting confused about the term “combination” which would be good in the glossary. He thought that combination in the *Code* was really referring to combining of two names, the generic name and the species name, the species name and infraspecific epithet, whatever that might be. However, where the confusion came in, was when there were parenthetic authors, because when you have that you were also combining two author names. He thought that was where people just intuitively started calling those things combinations because, where you had a single author you now had two authors, one in parentheses and the other one following it and that looked like a combination, at least not in the *Code*. He had found himself occasionally doing that, looking at a citation like that with two authors and thinking it was a combination.

**Turland** offered some information on what the Special Committee on Suprageneric Names thought about the issue. There were some proposals, he was not sure whether they were deferred from the St Louis Congress or they were additional proposals that arose during the Committee’s discussions but they had looked into the concept of using parenthetic author citations for suprageneric names. He conceded that there were obviously problems about definitions of basionym and combination. Currently the *Code* defined the basionym as name-bringing or epithet-bringing synonym. If, for instance, *Peganoideae* was changed in rank to *Peganaceae* it could not be a name-bringing synonym because the whole name must form the new name. It would not be like an infrageneric epithet becoming a generic name. It was not the whole name involved, only the stem. Similarly it was not an epithet-bringing synonym, it was a stem-bringing synonym. So, if the Section decided it did want parenthetic author citations for suprageneric names some of the definitions in the *Code* would have to be changed. But, putting that aside, the Suprageneric Committee did look at the matter and there was not majority support within the Committee for any proposal to introduce parenthetical author citations for suprageneric names. They considered a proposal but it did not receive majority support within the Committee.

**Malecot** suggested adding at the end of Art. 49.1 a cross-reference like “for suprageneric names see Rec. 19A” rather than a new note.

**McNeill** again assured the Section that if the proposal was accepted the Editorial Committee would look to see what the best place in the *Code* was for it. He did not see how to link with the Recommendation but, if that was the case, it would certainly be looked at closely.

**Ahti’s Proposal** was **accepted.**

### Recommendation 50A & 50B

**Prop. A** (57: 76: 20: 0).

**McNeill** resumed the already submitted proposals and moved to Rec. 50 A and B which were orthography proposals from Rijckevorsel that related to various standardizations of abbreviations. He added that they were, of course, Recommendations.

**Rijckevorsel** explained that the proposals were part of the general number of low-key, non-policy proposals. They arose from two occasions, firstly from orthography comparing that to the citation and secondly there was a discussion at some point by someone who managed a electronic database and had great problems keeping track of unpublished names because they occurred in the literature and he had to put them in his database but did not have the faintest idea of what abbreviations to use. Rijckevorsel could not really help him but felt he had an important point so had looked closely at the section in citations and noticed that it was quite out of synch with the rest of the *Code* with all kinds of provisions and categories of names which were not mentioned in the section and for uniformity’s sake he made the proposals so as to bring the section up to speed. He felt they were very sensible low-key proposals and did not have any strong feelings about them. He just wanted to put the matter up for discussion, suggesting that if there were people who were involved in electronic databases they may have ideas and suggestions. He was also interested in a suggestion on how to proceed. In Rec. 50C Prop. A the Rapporteurs had made a suggestion and secondly on Rec. 50 bis there was comment that there was a conflict between an illegitimate name and a conserved name, but he thought that Art. 14 stated that when a name was conserved it ceased being illegitimate so that could not be a conflict.

**McNeill** thought the proposer had rightly considered that the discussion could range over A through E. He did not think it would be out of order to discuss them, but encouraged not moving on to the others, otherwise the Section might just get confused.

**Rijckevorsel** suggested moving the whole set to the Editorial Committee.

**McNeill** agreed for the whole set of 50 A and 50 B.

**Gereau** felt that the current suggested rewriting for the Recommendations (Rec. 50A & 50B Prop. A–E) was confusing, using many more words and introducing unnecessary terms. He argued it should not go to the Editorial Committee but should be rejected.

**Gandhi** thought that the Recommendations were quite clear and concise and felt there was no need to make it more complicated. Presently, while indexing names for IPNI, he reported that they had started adding that a particular name was invalidly published and giving the reason, whether it was a pro syn. or nomen nudum. He thought people should just follow the Recommendations given currently.

**Demoulin** did not think the Section should judge the rules. In his opinion, each proposal had its own merits or problems and he personally considered that it was not necessary to fuse Rec. A & B. He favoured Prop. B and C, would oppose Prop. D. and approve a part of Prop. E. He therefore felt that each proposal must be discussed.

**McNeill** accepted that and moved to proposal A.

**Prop. A** was **rejected.**

**Prop. B** (59: 75: 19: 0).

**Demoulin** thought that the sense of Prop. A was to fuse two Recommendations. He thought proposal B could stand but leaving the Editorial Committee the role to place it as it thought fit. He felt it was a useful Recommendation to introduce some of the commonly used abbreviations, noting that in the morning session it was discovered that some abbreviations like “ad. int.” were not well understood. For example, “stat. nov.”, which he thought was not in the *Code*, while everybody used it, it would have been easier during the discussion on the change of ranks. He was in favour of the proposal.

**McNeill** explained that it was bringing in “nom. nud.” and “pro syn.”, but they were already in. Because the last proposal had been rejected, he thought this could be ruled as rejected because it belonged to the structuring of the Article just rejected.

**Prop. B** was ruled as **rejected.**

**Prop. C** (57: 76: 21: 0) was **rejected.**

**Prop. D** (34: 98: 22: 0).

**McNeill** moved to Prop. D which was dealing with “nom. oppr.”, referring to a name in oppressed work, an oppressed name, he supposed.

**Wieringa** thought it would be useful to have these abbreviations explained in the *Code*, even the last one. He suggested that maybe these should not be “yes/no” votes but whether or not the Section wanted to direct these proposals to the Editorial Committee, perhaps an entire vote on A–E, just to give the Editorial Committee freedom to adapt the Recommendations, to add more clear abbreviations to these Recommendations. His proposal was to have a general vote on all the proposals to direct them to the Editorial Committee and have them judge on them.

**McNeill** thought that the Section had dealt with the first few quite clearly negatively and as that route had been taken and there were only two left he thought the Section should just finish off dealing with them one at a time.

**Wieringa’s** point was that the last two votes were only “yes” or “no” votes, not to refer to Editorial Committee.

**McNeill** apologized and clarified that the president said that a “yes” vote would be to referred to Editorial Committee and a “no” vote was that it be rejected altogether and that Editorial Committee need not bother with it.

**Gandhi** pointed out that, as the Rapporteur noted, a few the abbreviations may be useful but in a glossary. He felt there was no need for a separate Recommendation or an Article and that the glossary should include such uncommonly used terms.

**Nicolson** clarified that reference to Editorial Committee did not necessarily mean it would be included in the *Code* but that it would be considered.

**Prop. D** was **rejected.**

**Prop. E** (38: 79: 36: 0) was **rejected.**

**McNeill** commented that this was the type of material that, in view of the vote, was the sort of thing that would appear not in the glossary but in a book on terms used in nomenclature, of which there were some around. He noted that these were not confined, of course, to the nomenclature of plants but perhaps other organisms. They were useful and people should know what the terms meant. He concluded that “we don’t want things in our *Code* that we don’t need”.

### Recommendation 50B bis (new)

**Prop. A** (31: 101: 20: 0) was **rejected.**

**Prop. B** (30: 101: 21: 0) and **C** (28: 48: 26: 0) were ruled as **rejected** because Rec. 50B bis (new) Prop. A was rejected.

### Recommendation 50C

**Prop. A** (19: 92: 40: 0).

**McNeill** thought Art. 50C Prop. A was a rewording of the current Article.

**Nicolson** noted that it was a proposal where the Rapporteurs had a suggestion.

**McNeill** explained that they were pointing out that if you just merely wanted to make clear what was meant by later homonym you could provide reference to the two Articles rather than restrict the manner of the citation.

**Prop. A** was **rejected.**

**Prop. B** (18: 58: 74: 0) was referred to the **Editorial Committee.**

### Recommendation 50E

**Nicolson**, following the afternoon break, thought it was time to return to our battles, or give up our battles and start the next battles.

**McNeill** explained that the next proposals were rather similar to the ones the Section had been dealing with that were essentially addressing points in the *Code* that were not particularly orthographic and presumably should be considered at this point rather than wait until the orthography proposals were considered. He thought they were rather clear in recommending the addition of various explanatory abbreviations of the like.

**Zijlstra** felt that with respect to “orth. cons.”, it was against established custom, which said “nom. et orth. cons.”.

**Demoulin** felt it was certainly not established in the literature he used. He felt “orth. cons.” was quite good.

**McNeill** clarified that the *Code* used “nom. et. orth. cons.” for a name proposed for conservation with a particular spelling because the name was also conserved at that point. He noted that things could be abbreviated any way you wanted. He wondered if it was another group that the Section might want the Editorial Committee to look at. He suggested a motion to refer the whole of the Recommendations to the Editorial Committee? [That was **seconded** and **accepted**?]

**Prop. A** (50: 80: 23: 0), **B** (40: 75: 37: 0), **C** (59: 60: 33: 0), **D** (29: 60: 43: 0), **E** (36: 71: 45: 0), **F** (35: 71: 46: 0) and **G** (41: 78: 33: 0) were referred to the **Editorial Committee.**

### Recommendation 50F

**McNeill** noted that these were orthography proposals.

**Rijckevorsel** indicated that he had nothing to add.

**Prop. A** (20: 88: 40: 1), **B** (18: 85: 46: 1) and **C** (19: 86: 44: 1) were **rejected.**

### Article 52

**Prop. A** (18: 51: *85: 0).

**McNeill** moved to Art. 52 and the first proposal from Brummitt who made the point that the wording of Art. 52.2(c) was not at all clear and he offered one method of addressing it. The Rapporteurs had suggested a different one. But they certainly both agreed that the Example certainly was a good one to include in the *Code* and a clarification of the Article was also essential.

**Brummitt** thought it did not seem necessary to add anything more and just hoped it would be referred to the Editorial Committee to correct it.

**Prop. A** was referred to the **Editorial Committee.**

**Prop. B** (28: 23: 102: 0) and **C** (38: 3: 110: 0) were referred to the **Editorial Committee.**

### Article 53

**Prop. A** (136: 13: 3: 1) was **accepted.**

**Prop. B** (13: 22: 118: 2).

**McNeill** introduced Art. 53 Prop. B as a proposal from Rijckevorsel which the Rapporteurs suggested be referred to the Editorial Committee. He reported it was s reference that the mail vote endorsed and it reflected the fact that there was a change in Art. 53 in the *Tokyo Code* and clearly some clarification was needed. The issue had already arisen in the discussions, that was the fact the mechanism for how one dealt with homonymy at levels other than that of family, genus and species was resolved in a particular way, so he felt it certainly had to be addressed editorially. How exactly it was addressed would depend on the outcome of something that he thought was pending.

**Moore** thought that another look at Art. 53.1 was needed and how that was worded now. He did not think that it was the intent of the Tokyo Congress to make it as restricted as it was in limiting homonymy. In editing *Taxon* manuscripts he actually did get a manuscript where someone used a later homonym of an infrageneric taxon. He had to explain the situation and given the current wording of Art. 53.1 that was not easy to do. He knew there was another reference, Art. 53.4 but the wording really was not as good. Art. 53.1 said these were later homonyms but then it only assigned illegitimate status to family, genus or species and did not really say that only those were later homonyms. He thought it needed revisiting because he did not think it was the wish of many people to permit homonyms at the infrageneric ranks or at the infraspecific ranks. He noted that the Section had already addressed the tricky case at the infrafamilial ranks.

**McNeill** agreed that would probably be the best solution because he thought it was a little more than editorial to make that change. But, at the moment this particular formulation could, he thought, be referred to the Editorial Committee and would be acted on in the light of whatever later proposal came to them.

**Prop. B** was referred to the **Editorial Committee.**

**Prop. C** (103: 4: 45: 2) was **accepted.**

### Article 58

**Prop. A** (41: 59: *52: 1).

**McNeill** moved on to Art. 58 Prop. A reporting the preliminary mail vote and noting that the Rapporteurs made a comment that the Example might help illustrate the Article as might a Note along the lines of “in the case of reuse at the same rank of epithets and superfluous names, the type of the name causing the original superfluity must be explicitly excluded.” The Rapporteurs did not think that the thrust of Brummitt’s proposal was anything but appropriate, but that some clarification would be helpful.

**Brummitt** noted that during the afternoon someone had said it may be clear to the few experts on the *Code* but if something was not clear to the average reader that was exactly his point. If you read through the logic you could see why it was clear to some but, hr felt vehemently that it was not clear to the average reader. He explained that their goal was to make it clear so that people could read the *Code* for themselves and see the logic behind it, because it was not a simple matter. Different sorts of illegitimate names were treated quite differently and he could accept that it was implicit in the hidden meaning behind some of the Articles. However, he much preferred to see it laid out clearly so that the Examples that he had given could relate to the wording of the Article itself. It was matter of clarity for users.

**Ahti** wondered if it was changed to “later homonym”, how about “superfluous” as it was another similar case which was very common.

**McNeill** asked if he was arguing against the change?

**Ahti** was not, he was trying to improve it. It was a suggested friendly change.

**Brummitt** wished to separate the means for superfluous names from later homonyms. He acceded that the logic appeared, at first, to be in conflict but felt it was not, so he did not accept it as a friendly amendment, he liked it the way he wrote it.

**McNeill** thought that the difference between what Ahti and Brummitt were saying was that the thrust of the proposal was to separate it into two different areas. The Rapporteurs did not feel that it was essential, that in fact, adding some Examples and clarifying some wording would do it. They certainly did not want the *Code* to get longer than necessary, but if it was necessary then it should be done.

**Zijlstra** was not yet convinced about the proposal but felt that if it was accepted then a small correction should be made to the Example. In the fourth line of the printed text it read “a combination of *Cocculus villosa* (Lam.) DC.” She thought that “(Lam.)” should be removed as the basionym was illegitimate so that the new combination was illegitimate as well.

**McNeill** had an ambivalent feeling about that point, even as Rapporteur, adding that we did not, of course, for a legitimate name include as a basionym an illegitimate name, because there was no priority so there was no parenthetic author citation. He explained that there were two illegitimate names and, again, logically, you should not have a basionym that was illegitimate, on the other hand, the whole thing was illegitimate and what they were trying to point out was that one was derived from the other. He suggested the Editorial Committee would keep to the practice, if it were put in, but make some clarification that it was based on the other name, without parenthetical author citation. He did not think it was a defect in the proposal, but simply a matter a little bit of editorial handling.

**Gandhi** suggested that in this case why not cite the parenthetic authorship in the *Code*. In practice, as already mentioned, parenthetic authorship were not included at all. If it was desired to indicate the illegitimacy he wondered why not cite the parenthetic authorship. That way it conveyed a meaning to readers that there was no necessity to include that.

**Nicolson** took off his presidential hat to make a comment. He thought the proposal dealt with superfluous names, as opposed to other illegitimate names, being used in combinations in which the name causing the superfluity was removed thus making the new combination legitimate.

**Brummitt** explained that the situation was reversed between superfluous names and later homonyms. In the old Art. 72 Note it made it clear that if a later homonym was transferred into a different genus you made a nom. nov. He thought everyone had understood that. But it said nothing about superfluous names. He argued that the same principle applied to superfluous names but not when transferred to a different genus. It. happened when you transfer them to a different rank because then the resulting name was not superfluous because priority did not apply across ranks. All he was trying to do was be clear that the logic behind it was the same whether you moved an illegitimate name to a different position, you made a nomen novum. But in one case, it was transferring it at the same rank into a different generic name, usually, but for superfluous it was when you changed the rank and trying to explain this to people was very difficult. That was why he wanted to lay it out in the *Code*. The Examples, he thought, would be useful, but you had to have Examples of something so he wanted to see the wording in full.

**McNeill** reiterated that the mail vote was 41 for, 49 against and 52 Editorial Committee.

**Nicolson** suggested it would appear that referral to Editorial Committee would be useful.

**Brummitt** was happy to just refer it to the Editorial Committee.

**Prop. A** was referred to the **Editorial Committee.**

### Article 59

**McNeill** introduced Art. 59. as one with a number of proposals that had exercised the Committee for Fungi very vigorously over the past few months and he reported that the Committee had diverse opinions on the matter and some members of that Committee, more particularly mycologists present and mycologists who had submitted some documentation, which would be available to the Section in the morning, regarding this proposal, were meeting in the evening to have discussions to see if they could reach a better agreement, perhaps by making some amendments to what was before the Section. For that reason they had asked, and the Bureau had agreed, that consideration of Art. 59 be deferred until Friday.

[*The following debate, pertaining to proposals relating to Art. 59 took place during the Seventh Session on Friday morning*.]

**Prop. A** (49: 27: 11: 32).

**McNeill** returned to Art. 59 and a series of proposals. He wondered if the proposals should be taken one by one or if there was some general statement being made first?

**Hawksworth** indicated that Demoulin would introduce it.

**Demoulin** noted that there had been a meeting of those members of the Committee for Fungi present which was not the full Committee but a significant number of them, including some past members of the Committee and they had a few points to address probably those which concerned proposals that had to be made from the floor and would be discussed later, but he felt there was an important one...

**McNeill** interrupted to make the quick point that if there was a proposal coming out of the discussion, it would be taken now, not later.

**Demoulin** asked if he wanted a discussion now?

**McNeill** apologized, what he was trying to say was that he knew there were some additional proposals relating to Art. 59 and they should all be included in the present discussion so people’s minds remained focused on it.

**Demoulin** had missed the point whether it was only what was related to Art. 59 or everything that had been discussed yesterday.

**McNeill** clarified that it was what was related to Art. 59.

**Demoulin** thought that when it came to Art. 59, it was rather simple and he was sure the Section would be glad about that. They felt that the issue was so complex that even if the majority of the Committee for Fungi had expressed its vote against the present proposals, there was a need for a Special Committee, an *ad hoc* committee, which would include people who were directly involved in this issue, which did not mean that decisions should not come back to the Committee for Fungi —not only specialists deal with something—but at the moment they preferred that an *ad hoc* Special Committee be set up for those proposals, with one exception. The one exception was Prop. B that related to epitypification and despite the rather heavy negative vote, he thought some people might want to discuss Prop. B right now and perhaps present some amendments. He thought Redhead had some friendly amendment to present on it. He suggested that the Section take a vote on referring the issue to an *ad hoc* committee, including Prop. B in case it failed.

**McNeill** enquired as to what the terms of reference of the Special Committee would be? To consider the proposals made to this Congress on Art. 59, or a broader mandate—consider revision to Art. 59?

**Demoulin** replied: the problem of nomenclature of pleomorphic fungi.

**McNeill** summarized that it would be a Special Committee on the Problems of Nomenclature of Pleomorphic Fungi.

**Demoulin** agreed.

**McNeill** had written “fungi with a pleomorphic life history”, but pleomorphic fungi would so, so that was the proposal and it was coming from a group of people so he assumed it was seconded? [Presumably so.]

**Gams** noted that in the Rapporteurs’ comment on all the proposals there was no statement about the vote of the Committee for Fungi, and it seemed important to him that he communicate this information now to the Section. The proposals made by Hawksworth had been voted upon by the Committee for Fungi as follows: most received a no majority; three “yes” votes, eight “no”, two “undecided”, and tow “for more discussion”. This was also for Prop. B, and Prop. C. Only Prop. E had five “yes” votes and that proposal was to the Editorial Committee to amend cross referencing. [See Committee report in Taxon 54: 830. 2005.]

**McNeill** clarified that that was Prop. E, for the record.

**Gams** asked if he was allowed to read out a few arguments against the proposals from his report.

**McNeill** thought that what would be relevant was whether he was supporting the setting up of a Special Committee.

**Gams** did support that.

**McNeill** thought the Section should address that and then discuss the more general issues.

**Nicolson** reiterated that the proposal was to have an *ad hoc* committee.

**McNeill** corrected him that the usual term was a Special Committee because he was not proposing that they report by tomorrow.

**Demoulin** confirmed that was not the plan and the reason for a Special Committee was the issue was so complex and evolving that it would take years to work out.

**McNeill** summarized that it would be the Special Committee on the Problems of Nomenclature of Pleomorphic Fungi.

A new **Special Committee on the Problems of Nomenclature of Pleomorphic Fungi** was **approved.**

**Nicolson** indicated that Demoulin would take care of the membership and report to the next Congress.

**McNeill** corrected him that that was not the procedure. A Special Committee was established by the General Committee, of course the Committee of Fungi would expect to be a major player in making the Recommendations.

**Nicolson** asked if the Section should carry on further discussion?

**McNeill** replied that there were some proposals that were going to be discussed. He asked the mycologists present whether, given that a Special Committee had been set up, it was useful to have a general discussion of the topic? He thought not, offering the other option of zeroing in on the proposals that were not being withdrawn, which he understood was all proposals apart from Prop. B. He thought, as it was an area that had been discussed very much by the mycologists yesterday and as a Special Committee would be addressing it, a general discussion on the problems of nomenclature of pleomorphic fungi would not be necessary or desirable, but full discussion of Art. 59 Prop. B, and any amendments that were made to it, would be most relevant.

**Buck** pointed out that the Nominating Committee was meeting in the afternoon, and wondered if there was any way that the proposers could come up with people on their Committee by...

**McNeill** interrupted to explain that the Nominating Committee did not deal with Special Committees.

**Buck** apologized.

**Prop. A** was **withdrawn** and **referred to the Special Committee.**

**Prop. B** (63: 14: 10: 32).

**Redhead** had some friendly amendments to the proposition but unfortunately had not typed them in and one of them was fairly lengthy so he requested some assistance. They were not numbered, but he was dealing with the additions to Art. 59.1, not the major paragraph, where it said “except where an epitype had been designated under Art. 59.8”. To begin with it would not be Art. 59.8, it would end up being Art. 59.7, but he had changed that entire statement there to insert the words “or epitypified under Art. 59.7”, and that was in the paragraph Art. 59.1 in the *Code*. The three things he was proposing simplified the wording. [Redhead gave detailed instructions to Elvira Horandl who was typing new wording into laptop attached to beamer]. He felt those changes were minor and they tightened it up and made it more succinct. There was a larger problem with the proposal regarding 59.4 because there were some repercussions of the new way of epitypifying, and there was no cap on it as far as dates went, and it had the potential for upsetting already established names, so there he had a larger friendly amendment, and it actually involved several things. [More and lengthy instructions to Elvira]. He explained that the reason he was proposing that was because in the new proposal, Prop. B, if you epitypified a name with a teleomorph, then the way it was originally worded would make the anamorphic name the holomorph name, and it was possible that if there were competing anamorph names you might have picked a later published one and set a precedent for it, and it was also possible that somebody could epitypify an anamorph name and upset an existing teleomorph-based name, which was pretty complicated. He noted that if people were not working with fungi and anamorphs they probably did not understand what he was saying, but that was the reason he had that in there, and he believed Hawksworth more or less accepted that idea. He was not quite convinced that he had got the wording perfectly straight and that the dates were appropriate, because he was trying to do it at the end of last night and this morning, so he was open to emendations to the emendation.

**Buck** asked if, on the last line, he meant “epityified” rather than “typified”?

**Redhead** confirmed that he did.

[Voice off-microphone asked Redhead a question about a date, 2006]

**Redhead** reiterated that the date was negotiable and asked people to please amend it as they saw fit.

**Hawksworth** thought that the meaning was quite clear but the wording would benefit with some more editorial attention.

**McNeill** thought that as long as it was matters that were not controversial in the fungal community the Editorial Committee would be happy to do the editorial modifications, but not as to substance of course.

**Gams** felt that the whole rather complicated move only made sense if things were really going in the direction of a unified fungal nomenclature, one name for a fungus, no matter whether it was anamorphic or teleomorphic. At the moment he thought that the mycological community obviously did not wish that although it was possible using molecular methods. He felt it was much more practical to stay [with the present rules] as long as fungal taxonomy had not progressed so far that genera of both anamorphs and teleomorphs were perfectly naturally circumscribed so that they coincided; [until then] all of the changes did not really make sense, and there was a majority in the mycological community, phytopathologists usually, ecologists, and others, who still preferred the dual nomenclature. Therefore, even with this elegantly improved proposal, it seemed to him premature to support it.

**P. Hoffmann** asked to see the whole proposal together on the screen. She thought there was more to it than just the paragraph [in view?]. She also requested clarification on whether the proposer specifically wanted to exclude the epitype being an illustration by using the term “epitype specimen” not normally used in the *Code*. If that was not the case, she felt it should be changed to just “epitype”.

**Redhead** responded that it had nothing to do with the illustrations.

**P. Hoffmann** agreed, but pointed out that it said “epitype specimen” and then it said an epitype was an illustration or specimen, so she thought, type specimen was never said.

**McNeill** reformulated the question as, were you in this Article insisting that your epitype be a specimen and not an illustration?

**Redhead** was following the wording that Hawksworth originally came up with.

**McNeill** thought that should be made clear, concluding that in these circumstances you would not permit an illustration to be type, noting that if that was made clear it did not need to be written in at the moment.

**Benitez** thought it would be better that a committee of mycologists decide all the proposal related to Art. 59, including Prop. B.

**Demoulin** offered to elaborate a little bit why they agreed that everything except Prop. B should go to Special Committee. He, like Gams, was in favour of retaining dual nomenclature for those fungi because, in his opinion, the applied mycology world, which was enormous: phytopathology, medical mycology, industrial mycology, would prefer to retain the familiar *Penicillium*, *Aspergillus* and so on names. But he thought Gams had been addressing the general issue, and this might have made him overlook the fact that Prop. B was not something that was linked to the disappearance of the dual nomenclature, it went in the way of making it easier to include with dual nomenclature to have the same epithet for something that could be based on the imperfect or the perfect anamorph or teleomorph stage. He somewhat disagreed with Gams on the fact that the general mycological community did not want that, because there had to be some very elaborate juggling with the *Code* to succeed in conserving *Aspergillus nidulans*, which was a major laboratory organism in molecular biology and genetics, and to retain the epithet *nidulans.* They had to conserve *Stegmatocystis nidulans* based on an anamorph specimen, which was somewhat bizarre, but what was done through conservation could be done much more simply with this proposal. That was why he was in favour of it, and thought it could be discussed and voted on right now.

**Per Magnus Jorgensen** thought it was a small step in the right direction. The original proposal had some weaknesses, but he thought that the friendly amendment took care of it. It lacked several other problems that he thought could be dealt with in between the next Congress with full discussion of the problem, which was extremely complex. He hardly understand it himself because he did not work in the field, but he had to learn about it. He thought it was an elegant solution to a difficult problem and was a first small step, which was not dangerous.

**Wiersema** noted that most of the Section would have before them the comments of some of his colleagues in the Systematic Botany and Mycology Lab of the US Department of Agriculture, which was also the home of the US National Fungus Collection, and these mycologists were strongly supporting the proposal, and with the tightening up that had been done he thought that they would still strongly support this proposal.

**Demoulin** thought that maybe the position of some of the mycologists could be summarized as follows: he and Jorgensen considered it was not a dangerous step toward the suppression of the dual nomenclature, while Gams considered it was a dangerous step. He felt that the controversy was on whether it a dangerous step or it an innocuous step, and he thought it was rather innocuous.

**McNeill** pointed out that they were both hitherto opposed to the proposal.

**Demoulin** thought they were rather in favour.

**McNeill** agreed they were now, but previously?

**Demoulin** agreed they weren’t previously.

**McNeill** felt that was the point.

**Gams** noted that there were simple cases of one anamorph species in a monotypic genus. If a teleomorph was discovered it was perfectly in order to epitypify it. That was the simplest case. In the future probably the date would have to be changed not only to 2007, but 2008 as Hawksworth had it originally. But the situation would become complicated if a large and anamorph-typified genus that might not be homogeneous was involved became holomorphic by epitypification.

**Gandhi** conveyed that of his Mycological colleagues at Harvard, a few were opposed and a few reluctantly supported this proposal.

**McNeill** thought there had been a good discussion from various sides, unless there was some new insight, perhaps someone carrying votes in support or against, he thought the Section should go to the vote.

**Hawksworth** responded to Gams’s comments, that there was a huge range of cases, as he pointed out, but one would expect taxonomists and people actually [peer-]reviewing papers for publication to look at the individual merits of a case and whether one should or should not in fact go and apply this Article; nobody was obliged to use the method, and it would be a matter of looking at it very much at a case-by-case basis when people were doing revisions.

**Wieringa** on a technical matter, thought that the last date, “after 1 January 2007”, should be removed [so as] not to upset present nomenclature. He added that there was a first “1 January” already for the teleomorphic typified names published before, but then subsequently epitypified.

**McNeill** asked if he was saying “on or after”?

**Wieringa** thought that date should be removed because elsewhere an epitypification done today would be possibly upsetting to present nomenclature. He thought that if you took that out there was no problem.

**McNeill** thought it was probably editorial, a matter of whether the other date was really necessary or not. He felt there was no question that this was something that applied as an “on or after 1 January 2007”.

**Redhead** explained that the intention was to protect existing teleomorphic names, lest somebody epitypify an older anamorphic name with a teleomorph and then displace an existing teleomorphic-based name. He was trying to get the wording correct with the dates, so as long as any editorial change made, should the proposal be accepted, reflected that intention, that would be fine.

**McNeill** suggested, for the purpose of voting, to leave the wording as it was presented by Redhead and if it did require editorial attention that could be addressed because he thought it did make the meaning clear that you could not retroactively displace a name in the past, which was what was very important for stability.

**Redhead** returned to the question about the date, and thought the date 2008 was what was in the original proposals, so maybe that should be changed to 2008 everywhere?

**McNeill** asked what the rationale for that was? Normally when a change was made at a Congress the date at which it was implemented was the 1st of January following the date of publication of the *Code*. The *Code* had, for the last three or four editions, been published in the succeeding year, he hoped to keep to that schedule, and in this case that would be 2006, so the normal practice was to have it implemented on the 1st of January immediately after. He added that it would be an anomaly if it was 2008, but there may, for some mycological congress reasons, be a good reason for the anomaly.

**Redhead** believed it was because this was such a major change for mycologists that more time was being allowed.

**Hawksworth** explained that the date was related to the whole package, because this was quite a major thing for mycologists. He reported that very few mycologists belong to IAPT, or ever looked at a *Code*, ever went to a botanical meeting; they went to mycological ones; even the plant pathologists hardly ever went to mycological ones, so a very long lead time was necessary to get the community to actually know, that was the concern.

**McNeill** pointed out that this was enabling legislation rather than enforcing legislation in which the lead time was less important.

**Hawksworth** agreed, so he thought that for the proposal it was less of a problem. The taxonomists likely to do this sort of work would know about IAPT and the *Code*, and would be watching what happened at this particular occasion because they knew it was up for discussion.

**McNeill** felt that if something was enabling, the sooner it was implementable the better to get people gradually to know about it. He felt it was quite different when all of a sudden you had to do something new; at that point it was very important to make sure that everyone knew.

**Redhead** thought there was one other issue which was not quite resolved, and he was not certain how it could be resolved, and whether it involved other parts of the *Code*, but if one were to take an epitypified anamorphic name with a teleomorph that turned out to be very bizarre and for which you wanted a new genus, and you wanted to describe it, what would be the type for the genus? Because the type for the anamorphic name was still the anamorph holotype, to which we have an epitype, and he was not certain how to tweak the *Code*.

It seemed to **McNeill** that this was the sort of thing that should be addressed once the situation arose. He suspected that conservation might deal with some particularly difficult cases, and if it became a regular matter it could be amended at a later Congress. He imagined that Redhead may have a case in mind, but it seemed to be a rather special case.

**Redhead** admitted that one of the reasons that he was supporting the proposal was to test the waters for what else was to come with Art. 59.

**Watson** responded to the Rapporteur’s request for institutional comments, and the mycologists at Edinburgh also supported Prop. B.

**Prop. B** was **accepted** as amended.

(a) A new Art. 59.7 to read: “59.7. Where a teleomorph has been discovered for a fungus previously known only as an anamorph and for which there is no available name for the holomorph, an epitype exhibiting the teleomorph stage may be designated for the hitherto anamorphic name even when there is no hint of the teleomorph in the protologue of that name.”

(b) Revise Art. 59.4 to read: “59.4. Irrespective of priority, names with a teleomorphic type or epitype (Art. 59.7) take precedence over names only with an anamorphic type when the types are judged to belong to the same holomorphic taxon. Priority of competing teleomorphic typified or epitypified names follows Principle III except that teleomorphic typified names published before 1 January 2007 take precedence over anamorphic typified names subsequently epitypified after 1 January 2007 by teleomorphs.”

(c) Insert in Art. 59.1 after “typified” “epitypified under Art. 59.7”. and in Art. 59.2 after “its type specimen” “or its epitype specimen under Art. 59.7” and at the end “(see also Art. 59.7)”.

**Prop. C** (60: 16: 10: 32), **D** (49: 16: 11: 32) and **E** (35: 15: 43: 26) were **withdrawn** and **referred to a Special Committee.**

[*Here the record reverts to the actual sequence of events*.]

### Article 60

**Prop. A** (138: 4: 11: 0).

**McNeill** moved on to Art. 60 and its associated Recommendations Rec. 60B, C, D, E, and F. He thought there was still time to address them before inviting Rijckevorsel to make a presentation. He suggested starting by dealing with Art. 60 Props A, B, and C separately because they were made by other persons. He introduced Art. 60 Prop. A by Wiersema and one Nicolson and reported that it had received very strong support in the mail ballot 138 “yes”, 4 “no”, 11 Editorial Committee.

**Demoulin** contributed that for once he was not very happy with a Nicolson proposal on orthography because he thought it went in the wrong direction, although it probably made things clearer and that was why it got support in the mail vote. It made it clearer in the way of standardization, an issue he felt it was unfortunate to standardize so much and where a tendency to try to work more like other codes do, should be to give more respect to original spelling as zoologists did. It was the most difficult part of the orthography section and the one that had always made the big problems and made him very unhappy during many congresses because when it dealt with the formation of epithets from the name of a person there was a consideration that older authors were always giving, during the 18th and 19th century, as good as possible and respect for the way words were pronounced in the language of the person that you were supposed to honour. He felt that the present tendency to standardize with rules like this one did not really take into consideration, Latin or any language, pronunciation. It was the old story which came back almost every Congress. He alerted the Section to the fact that even if French was derived from Latin, if something was written with -er in French, it was not pronounced the same way as -er in Latin. He gave the example that if you wrote the equivalent of Labillardiere in Latin there should be no final “e”, it should be like Moliere. He pointed out that everybody in the 19th century had tried to be as close as possible to the original way of saying the name and to be as close as possible to good Latin had been making *labillardierus*, *labillardieri*. Changing this, as we have been doing since Sydney was offensive, he thought, to the name of one who contributed to Australian botany and it was pity that it happened in Sydney. He suggested that people may go and do a worse thing now with terminations that are, for example, ending with “ee”, something purely Anglo-Saxon that did not happen in Latin, *Acacia brandegeeana* did not make sense in Latin as you would not have a succession of vowels like that. If this proposal passed he suggested it would affect, for example, *Phycomyces blakesleeanus*, which was an economically important fungus, in which case he would make a proposal for the conservation of the usual spelling with a single “e”. He was very, very much against the proposal.

**Wiersema** noted that there already was a problem in the *Code* that the proposal was attempting to address and that was the conflict between what it said in Art. 60.7 and what was in Ex. 11 under that Article. The history was that at the St. Louis Congress there was a proposal to modify the Article, in fact Nicolson was a co-author, which got defeated along with all the other orthography proposals but yet some of the associated Examples in the discussion of that proposal ended up being incorporated into the Example, which was expanded. This meant there was not adequate coverage in the Article to explain why these changes were necessary. He explained that they had looked at all those cases, suggested modifications of the Article to cover the cases that were present there and looked at some additional cases that were not adequately treated by Ex. 11 or 10. The double “e” was one of those. In Ex. 10 a consonant was converted to another consonant and that was OK, you did not correct those epithets. In Ex. 11 it was where a vowel was changed to another vowel and you did correct those but it said nothing about the case where a vowel or a consonant was dropped. Again, the Article did not tell you what to do. He felt that the Example did not clarify the situation so they had tried to incorporate into the Article a means of accounting for those situations. He elaborated that the one particular case that brought this on was a conservation proposal dealing with *Solanum rantonii* which was being proposed for conservation with the widely used spelling (in horticulture at least) *rantonetii*. Adoption of the proposal would avoid the need for conservation in that case. They had looked in IPNI to find any instances that might be affected and, granted there probably were other terminations of French names or names in other languages that were not considered, but of all the ones that were considered they found no other instances that would be impacted by this, just the single instance. He assured the Section that he had looked extensively at the impact in the case of the other situations and highlighted that it was all presented in the original proposal.

**Gams** had to disagree with Demoulin, in this case. He was very much in favour of having a rule of grammar that solved the problems, as far as possible, rather than judging case by case and, if necessary by conservation. He pointed out that apparently the discussion was on A & B together, including the Examples. In looking at these Examples he was missing one case, Desmazieres, a plural French name. He wondered if that should be *desmazieresii* or *demazierei*. He suggested that perhaps that could be added as a friendly amendment.

**McNeill** asked which he preferred

**Gams** responded *desmazierei* – making it singular and adding -i.

**Nicolson** felt that Demoulin had given a very eloquent point and it would be possible that there could be conservations to overcome these, although it would not be efficient it would be possible.

**McNeill** noted that that would be for where there was clearly a disadvantageous change for a very important and widely used epithet, which was the reverse of the situation described by Wiersema.

**Brummitt** felt it was about time personal epithets were sorted out. He was very strongly in favour of the Nicolson & Wiersema proposals and he very much hoped they would go through as it would solve a lot of problems.

**Nee** wondered if it would conflict with the fact that you could form a name arbitrarily in any manner whatsoever? Or the case where you have the epithet “pennsylvanica” vs. “pensylvanica”, both original and correct for different species named after Pennsylvania that was named after William Penn?

**Nicolson** and **McNeill** both answered “No”.

**Gandhi** also supported the proposal. Since the cited Examples pertained to North American literature, quite often he got curious why the epithet *brandegee* was spelled with single “e” or a double “e” and this proposal solves the problem. If you looked at IPNI and made a query on brandegee you would find nearly 60% with double “e” and about 40% with single “e”. It was always a dilemma for them just to keep it both ways. The other thing he wished to mention was about implicit latinization of names. He thought that people may be familiar with the western names but if you did not know the language, it was very difficult to guess whether the particular ending was the latinized form or not. In India what happened to be the first name may be the family name or the last name may be the personal name. he cautioned against equating every name in the world as equivalent to a western name.

**Wiersema** addressed Nee’s point, noting that it only involved changes to personal names, not geographical names.

**Demoulin** felt there were a few points he needed to address. One was that the problem mostly with the names of the past, when people knew their Latin well and were trying to find the best possible latinization and had never heard about standardization. If in the future one must form the name under a standardized rule, and this would apply to names from many different linguistic origins, he did not think it was a big problem. What he did not like was to apply this to the past. He asked why in the 19th century the French names ending in -iere, were, he thought, universally treated as -*ierei, desmasierieri* or *labillardierei*. Why should we come back on [i.e. change] what people who knew better than we what they were doing? He did not think it was a matter for the future, it could be handled he thought in a rather elegant way through Rec. 60C Prop. G which he thought people may not have looked at attentively because there were so many proposals in 60C. He thought there was a way perhaps toward a general standardization but maintaining some well known exceptions, with the problem that people would fight over their favourite (Labillardiere, Blakeslee, etc.) but he thought the idea was a good one. It was to have epithets commemorating a well-known botanist or naturalist Latin genitive singular, 2nd declension,... Berzelius, Allemand etc. He added that you may have *solandri*, based on Solander... that was a list of exceptions. What he found shocking in the proposal was a consequence as shown by the Example in Prop. B like *Acacia*, he was always shocked by *loureirei* changed to *loureireoi*. He had been fighting this for thirty years and had no perfect solution to offer but this was not, in his opinion, a more perfect solution than anything that had been suggested before.

**Ahti** wished to second the proposal to add a French plural. He remembered another case: *abbayesii* deriving from Henry des Abbayes.

**McNeill** asked if that was something the proposers would accept?

**Nicolson** agreed it was.

**McNeill** noted that it was a friendly amendment and had been incorporated as part of the proposal.

**Perry** wondered if the word “corrected” in the last line of the Article, could be changed to “standardized” as it was not a correction it was just that it was being standardized by this method.

**Nicolson** asked if that was something the Editorial Committee could consider?

**Perry** felt it would conflict with what was generally stated in Art. 60.1.

**McNeill** thought that could be accepted as editorial or alternatively accepted by the proposers. [The proposers **accepted** it as a **friendly amendment.**]

**Nee** had a slightly impertinent question, he asked if anyone could think of any examples of species named after Linnaeus which were latinized from Linnaeus and von Linne as he pointed out it would be sort of embarrassing to put this in and then find out we had to correct Linnaeus’s name. He did not know of any examples himself.

**David** noted that there was a friendly amendment relating to Desmazieres and requested it be written up because he thought it actually ran contrary to the proposal.

**Nicolson** thought it could be referred to Editorial Committee, rather than trying to work it out right here.

**McNeill** could not see it and asked if it was up on the board yet? [No.] He wondered if it was actually relevant to the particular proposal or did it belong in different place? He suggested that it seemed to be quite unrelated and thought it could be looked at later in the general orthography situation.

**Buck** disagreed, for example the original epithet *abbayii* would then be standardized to *abbayesii*.

**McNeill** felt that was his point, that it did not seem to belong here and should be looked at further. He thought it would be much better to stick to the original proposal. There would be more about orthography in the afternoon so he felt there would be an opportunity to put it back if it was important. He proposed dealing with the proposal as originally formulated.

That was also **Nicolson’s** preference. He had no objection to introducing or considering the suggestions but wished to check what original publications did and whether there would be changes or not.

**McNeill** concluded that there had been a rather full discussion and it was a quite clear situation: either the Section standardized, as had been suggested in the proposal even though this caused pain to people who were well classically trained or the Section accepted the alternative point of view and allowed full freedom and the proposal would be rejected. He thought the choice was fairly clear toward standardization or alternatively to retain somebody’s better Latin.

**Nicolson** thought A and B formed a package.

**McNeill** noted that if Prop. A was defeated, Prop. B would automatically fall.

**Prop. A** was **accepted.**

**Prop. B** (138: 4: 11: 0) was referred to the **Editorial Committee.**

**Prop. C** (44: 7: *99: 2).

**McNeill** introduced Art. 60 Prop. C as having 99 Editorial Committee votes, reflecting a suggestion that it might better be editorially incorporated in Rec. 60G.1 and that an Ed Editorial Committee vote would be so interpreted, so an Editorial Committee vote was also a positive vote.

**Brummitt** briefly outlined that the proposal arose from his attempts to teach the principles of nomenclature to students and they found there was no guidance on how make these compounds. The present Art. 60G gave only exceptions without giving the way to do the common standardizations like *aquilegiifolia* and so on. The Rapporteurs had given good support and the vote gave good support so he was keeping his fingers crossed.

**McNeill** asked if he would be happy that it be referred to the Editorial Committee, that was as to placement, not as to comment?

**Demoulin** did not object to discussing it in the Editorial Committee but he drew Brummitt’s attention to the fact that in the Recommendation it may not be so much an Example of common formation and pseudocompound [that’s where there’s a problem] but also they included an Example of how to form a compounding form and once it was understood that *caric*- was a compounding form, let us speak of food, thus for *Carica* and also for *Carex*. There was no problem of adding more Examples but the Examples were there in the bottom.

**Gandhi** supported the proposed Example.

**Prop. C** was referred to the **Editorial Committee.**

### General Orthography discussion

**McNeill** thought it was time to go to the main body of proposals in Art. 60. He realized that there were other proposals, other than those by Rijckevorsel that related to orthography that were yet to be addressed and assured the Section that they would be addressed in due course but thought this was the appropriate time to invite Rijckevorsel to make a presentation.

**Nicolson** asked Rijckevorsel to speak and gave him 15 minutes.

**Rijckevorsel** started by saying that he had many proposals, ranging from very minor editorial proposals to very speculative proposals, so he felt that many things were possible, depending on the mood of the Section. As he did not know what the Section wanted to discuss most he chose to start by addressing the two main points to give the Section an opportunity to decide. He thought the two main issues concerning the orthography were the general format and Rec. 60C.2 which addressed epithets based on personal names. He gave a quick overview of history starting with what was in the *Vienna Rules*, a single paragraph on orthography which was new. He noted that 100 years ago, also in Vienna, there was a big clash between several different people who were quite angry and the rules were changed to look quite like what was in the *Code* now. He reported that in the *Brussels Rules* it was unchanged. But later quite a lot was changed. Recommendations were also added which was not so much the result of new material as the fact that they moved what was now Rec. 60B and 60C out of genus names and specific names. He thought a quite useful point to make was that if you defined orthography as correction of existing names then it belonged in both Art. 18 on family names and Art. 60. He added that, looking at the section on orthography, it contained very many things which actually concerned the formation of names. In the zoological *Code* he pointed out that there was no distinction between orthography and formation because in Zoology, if you made a name that met the criteria of the *Code* then you were in and you were safe. He summarized that there was a big expansion in [the *Cambridge Rules* of] 1935 then nothing much happened in Amsterdam. In the *Stockholm Code* quite a big new paragraph on compounding was introduced, which made a “back door” rule at that moment that if a name did not meet the Recommendation then it should be corrected. At the same point, in 1950, there was also the start of what was now Rec. 60C.2 and also the intentional latinization paragraph which was now 60.7 and which originally addressed only personal names. He explained that in the *Paris Code* the paragraph was renumbered, now 73 and new revisions on diacritical signs were added. The big change was then in the *Leningrad Code*, he thought it was quite a few changes and it stayed much the same although it was again renumbered. This was, of course, also now at this point that the *Code* was mostly used by botanists it was also used by people in general, especially now that it was on the internet. As many more people were engaged in spelling names than in using other parts of the *Code*, he felt that the section on orthography should be the most accessible part of the *Code* and, obviously, it was not so. He suggested that if you talked with people about orthography [they thought] the Article was too long. He felt this was also exemplified by the synopsis of proposals where all the proposals on Art. 60 were put together so the Article was so long that if you wanted to split it up it proved to be fairly easy because there were “back door” rules. That meant that there was a paragraph which stated that was something was obligatory and then you had to turn to a Recommendation which was very unhandy and so you had to put them together. He argued that if that was resolved then the rest straightened out.

The other issue he wished to raise was Rec. 60C2 where a rule, Art. 60.11, made the part of Rec. 60C1 on terminations obligatory but there were exceptions to it. However, he continued that if you looked at Rec. 60C.2 and tried to find out what those exceptions were exactly it was hard going. A reason to address this Recommendation was that knowledge of Latin among botanists was declining. Which meant that it would be handy if the *Code* would offer more guidance. Secondly it was also to be expected that Nomenclature Sections of the future would have less knowledge of Latin. He referred to a paper by Nicolson to the Leningrad Congress highlighting one of the key things about augmentation. The Romans used very short names and only when they belonged to a noble house was an -i- put in as an honorific augmentation which was present in Rec. 60C1 but was not necessarily present in Rec. 60C2. He had looked at the issue, which could only be done today because it was on line and since fairly recently it shifted so that literature references were immediately on the search page, which helped immensely in trying to dig up literature. He acknowledged how difficult it was. So he looked fairly hard on very many cases, mostly on given names and had found interesting facts. He put a development list on the internet. For those had not seen it, there were several phases. For quite a while all the important literature was entirely in Latin, including the authors, all those names were declined in Latin automatically. When people started naming plants after presidents they used those forms. Then in the intermediate period there was a frequent use of the names as they were existing surnames which were not considered to be Latin but were, nevertheless declined. Then given names as a basis for epithets was fairly recent, coming in only after 1850–1860. But the development of given names in Latin were nonetheless used as epithets, but then for other things: geographic but also for surnames. Actually a given name was a very unhandy thing because you’ve got very many different terms to refer to them which don’t all necessarily mean the same thing, certainly not to all people. When the *Code* referred to given names the phrase used was “given names” but in the past it used “Christian names”. Actually given names were also not a single concept which was very nicely exemplified by Francois. If you look at epithets named after Francois then they may be in Latin form *francesci* or may be based on the official given name or it may be based on what might be a vernacular name. The point he wished to make was especially this one. The moment a name was published the person was being honoured. He finally came to five basic categories and found it to be a very useful basic frame for any discussion or new Recommendation. He concluded by saying that if the section was interested there were lots of options but if the Section was not interested then there was not much point in going further.

**Nicolson** thanked him very much for the presentation and being right on time. He asked if proceedings were now at Art. 60 Prop. D–RR?

**Prance** noted that it was a package of various orthographic things, some good and some that needed some debate. He wished to propose that the whole package be referred to the Editorial Committee, rather than spend a lot of time discussing them, because most were things that the Editorial Committee could make good decisions on.

**Nicolson** [loud groan followed by laughter] thought that meant that Prance would be on the Editorial Committee.

**McNeill** checked that it was seconded. [It was.]. Again he assured the Section that it meant that the Editorial Committee would look at it very seriously but it did not mean that any or all would be incorporated. If in the judgment of Editorial Committee, and it certainly was the judgment of the Rapporteurs, there were elements that changed the meaning of the *Code* they would not, and could not, take them.

**Funk** thought that Art. 60 was too long and felt that maybe a few moments of discussion on whether or not we should consider, in the future, doing something like this might be a good idea. She personally was not prepared at this time to make that kind of decision. She did think it warranted a little discussion, maybe just a few minutes, to see what the sense of the meeting was.

**McNeill** felt that could arise independently and then we could have a discussion of where the orthography section of the *Code* should go in the future, that would be perfectly in order.

**Demoulin** thought it was reasonable to do as had been proposed, despite the workload. But, he was worried about a situation when there really was something that could not be handled by the Editorial Committee. He wished that the Section would not follow the Rapporteurs and those that voted no because, what he felt would happen now, was that each time something was “too new, we can’t do anything”, it meant it was postponed to the next Congress. When he prepared his vote he tried to make a distinction between things he wanted to vote “yes”, “no”, or Editorial Committee. It was true that many things he pushed for Editorial Committee, but there were things for which he wished to vote “yes” or “no”, in fact there were many things where he voted “yes” or “no”.

**Nicolson** thought that was a good point and that a number of the Section had done that. He certainly felt that many things could be Editorial Committee but had a few he would definitely say “no” to. But that was personally and not as president.

**Dorr** was curious, if the Section followed Prance’s proposal, would Art. 60 Prop. J which received a 75% negative vote, also convey to Editorial Committee as part of the package or would that drop out?

**McNeill** thought that, clearly that was something that the Editorial Committee would think was not something that they would take terribly seriously, purely by the vote. On the other hand, as somebody had said, if it was a proposal for change, then clearly they just could not touch it. Those proposals that were quite clearly changes in the *Code*, changes to the rules and the application of orthography rules could not be touched editorially. There may be within them an incidental part that clarified the wording but not the thrust. As the published papers indicated, the first set of orthography proposals published up to number 55, were primarily, but not exclusively, editorial and the later ones were primarily, but not exclusively, improvements/changes and obviously the greater amount of editorial modification may come.

**Gereau** felt that if the overall thrust of the entire set of proposals was, indeed, a simplification or clarification of the *Code* then he would readily agree with Prance. On the other hand, having looked all of them through, and having looked at the mock-up of the total results that were on the web that would result from the acceptance of all of them, he did not see it as either clarification or a simplification. That said, he did see some elements of value indicated by the scattering of “yes” votes indicated on his own mail ballot. He would greatly prefer, time-consuming as it may be, to go through them one by one, vote on them as an assembly, those who were interested in doing so.

**Kolterman** understood the Section was discussing Prance’s motion. He just wanted to make clear whether that included the set of proposals on orthography and the additional proposals as well as the other ones deferred from previous Articles or whether it was just Art. 60.

**McNeill** clarified that it was, in fact, the full package of orthography proposals because Art. 60 did include both sets, all the proposals by Rijckevorsel on orthography, including those passed over.

**K. Wilson** thought it was terrific that Rijckevorsel had taken on trying to clarify this section of the *Code*. She did not think it should be left to the Editorial Committee to have to try and make sense. She agreed very much with other speakers that the Section needed, unfortunately, to go into the proposals to try and make sense of what was acceptable and what was not.

**Demoulin**’s position was in between, as he felt he had said. Unfortunately when he read the Rapporteurs’ comments, they said “this proposal is not purely editorial in purpose, it extends beyond editorial and would change the meaning of the *Code*. Such proposals are discussed individually under their respective Articles and Recommendations.” He wondered if he had missed something or if there were some notes that were not included in the report that could help decide what was purely editorial so that the Section should not be discussing here until Saturday, and those where it was felt that there really was a change and he thought should be discussed now, otherwise they would be postponed for six years.

**Unknown Speaker** felt that some of the proposals were so specific, maybe it was necessary to set up a Special Committee.

**Watson** wondered if a way forward would be for those with specific notes to compare those notes and come up with a short list of what they considered to be non-editorial proposals for discussion tomorrow?

**Ahti** had marked seven cases which he thought should be treated here and the others could go to the Editorial Committee.

**McNeill** asked for clarification whether these were proposals that were thought beneficial but which were not editorial?

**Ahti** clarified that he meant those which he thought were not purely editorial.

**McNeill** responded that there were many, many more than seven that were not editorial.

**Ahti** meant those that he would not just leave to Editorial Committee, agreeing that most of them were purely editorial.

**McNeill** thought that what would be worth discussing were proposals that people thought would be improvements in the *Code* that were not editorial. He pointed out that there was no use discussing things that were editorial that people did not think would be an improvement and added that, obviously opinions on that would vary. He assured the Section that the Editorial Committee would not make a change, even if individual members of the Committee thought it was a good idea, if it was a change and had not been endorsed by the Section.

**Nicolson** offered his own notes on what probably was a no and suggested starting there.

**Atha** suggested that the Section just go through the whole thing and if the Committee thought a proposal was going to have no change, they should speak up and say that and if the group accepted it then the Section would move on.

**McNeill** returned to the proposal on the floor to refer all the proposals to the Editorial Committee which had to be dealt with, or withdrawn. He added that it had been seconded. He clarified that the proposal was regarding all the outstanding Rijckevorsel proposals on orthography.

**Wieringa** wanted to know if that would mean then, if the Section passed all of the proposals to the Editorial Committee, if there were any real changes in some of the proposals they could not be implemented because the Section had not voted “yes” for them?

**McNeill** agreed that the Committee would not implement anything that was a change, it would only implement things that seemed a clarification, improved wording. He noted that the Committee would certainly be able to remove the “back-door” component if it could do so without changing meaning and find a happy wording to do so. He reiterated that they certainly would not adopt anything that was definitely a change in the present meaning.

**Wieringa** felt that meant that the Section should actually vote at least on all the proposals that implemented real changes.

**Nicolson** pointed out that there was a proposal to refer all the proposals to the Editorial Committee. He thought that several people were speaking against doing that. When push comes to shove the Section would have to vote on the proposal to send all to the Editorial Committee.

**Unknown Speaker** insisted that that meant an implicit no for all those that were real changes.

**McNeill** agreed that that was correct.

**Nic Lughadha** felt that it could be argued that since Rijckevorsel had proposed them as editorial that any extensive changes were, in fact, unintentional.

**McNeill** did not think that Rijckevorsel said all his proposals were purely editorial.

**Turland** clarified that that was the first set of proposals. He also mentioned that the Rapporteurs pointed out, in the Synopsis of proposals, those proposals that they believed were more than just editorial. Even in the first set, he believed that Prop. J, notably, was a little more than purely editorial. He acknowledged that it was quite possible that the Rapporteurs had overlooked one or two cases where the proposed changes would be more than editorial and when the Editorial Committee came to look at those, if these were referred en bloc to the Editorial Committee, then of course, the changes would not be implemented. But, he felt that if members of the Section here had comments about individual proposals that had consequences that would change the *Code* and if all the proposals were referred to Editorial Committee that information could be lost. He thought it would be valuable to have the views aired.

**McNeill** made a suggestion for moving forward, that there obviously was some feeling that the Section did not want just to say Editorial Committee to them all. What he felt people were interested in were proposals that would be beneficial but which were, in fact, changes, and if the number was modest, 20 or 30 at most, preferably less, then that would be something that could be addressed separately. There would be time tomorrow for individual proposals to be taken up to be approved. He suggested that if members of the Section saw important things that they wanted to have addressed they should be noted. On the other hand, if the number was enormous, then he suggested that the Section might as well deal with all the proposals in sequence one at a time.

**Kolterman** suggested moving on to a couple of other proposals that were not part of the package, and having people, between now and 9 o’clock tomorrow morning, put the numbers up on the board that they considered to be major changes and then there would be an idea of how many there were.

**McNeill** summarized that the proposal was that discussion should be suspended to allow people to put up on the board tomorrow morning the proposals they would like to have discussed, as an in-between stage between referring everything to Editorial Committee and thereby precluding any things from being implemented that people thought were favourable, to having a specific number of proposals identified tomorrow morning to be voted on individually and the rest referred.

**Watson** asked if that was a proposal.

**McNeill** responded that tt was a proposal to defer not a proposal not to deal with the issues

**Nicolson** was prepared to consider that possibility.

**McNeill** said it was really an amendment to the proposal to immediately refer all to the Editorial Committee; it intended to defer implementation of that.

**Nicolson** asked for a show of hands of who would like to work on decisions of which things were purely editorial and which were not?

**McNeill** clarified that they were looking for things that were not purely editorial that commended themselves and reiterated that there was no point in identifying things the Section did not like.

**Nicolson** asked for people who would pick out the proposals that they really felt must be dealt with and not referred to Editorial Committee and thereby lost.

**McNeill** interrupted to first find out if the Section agreed to the strategy of deferring until tomorrow.

**Nicolson** asked if it was the will of the Section to defer the discussion until tomorrow so that people could have notes they would like to communicate? [This was accepted.]

**McNeill** noted that there was nothing against people getting together and discussing so that what they put on the board would have more impact.

**Nicolson** reported that Gereau would be glad to be the chair, the focus point.

**Demoulin** really did not see why things should be complicated. It seemed perfectly straight-forward to him that most would go to the Editorial Committee but, if in there were things that were new the Editorial Committee could not do anything about it and then they would go to the next Congress. If anybody did not need to raise their hand right now, fine... between now and tomorrow morning there was one of those proposals that they felt was a real new proposal and was something they would like to have discussed then tomorrow morning they could say “Please take up Prop. X”. He felt there was no need to say now that we were going to do it tomorrow.

**McNeill** replied that that was exactly what had been decided. The only thing point was suggested that those interested in this might talk about it and thereby have, perhaps, a greater consensus for discussion in the morning.

**Nicolson** concluded that the Section would come back to the issue tomorrow. The motion was to refer all to the Editorial Committee and it would be helpful if people with particular concerns would put it on paper and communicate it to the Bureau, whether functioning as Committee or as individuals.

[*Discussion that followed of Rec. 60C Props A and B and Rec. 60F Prop. A relating to orthography occurred here and have been moved to the Seventh Session on Friday morning following the sequence of the* Code.]

## Seventh Session

Friday, 15 July, 2005, 09:00–13:00

**Stuessy** again had a few announcement regarding the dinner. A number of people had asked, “What’s the dress?” and he explained that “we Europeans tend to be a little more formal about it”, so they would look kind of nice, but people from other countries, not to be mentioned, were welcome to come in their bathing suits or whatever they liked. He reminded those attending that there would be a very exciting auction of IBC memorabilia, so exhorted everyone to save their cash to contribute as any proceeds would go to support student poster awards at Symposia that IAPT was involved with such as at the Latin American Botanical Congress. He also noted that the Nominating Committee would meet at lunch time.

**McNeill** also had a few announcements. First of all, he wished to remind the secretaries of the Permanent Committees that they would be expected to present a brief report towards the end of the proceedings, essentially the work of their Committee over the past six years and the highlights, if they had some, and they should also prepare a written text that could be used in the report of the Proceedings to summarize where the reports were published, composition of the Committee and anything that was important for the long term record. The second announcement was that he would also be inviting the Conveners of five of the seven Special Committees that had been set up in St. Louis to report, adding that two of the Committees had already reported in *Taxon*, but five Committees had yet to report. He acknowledged that the report might of course be that the Committee had done absolutely nothing, but what happened to what was set up in St. Louis should be in the record so that people looking back in time would see what really happened with Division III and with lectotypification of older generic names and various other hot topics in St. Louis. Finally, he had an announcement which did not really have anything to do with the Nomenclature Section but had a great deal to do with nomenclature—a more personal announcement—and that was regarding the nomenclature columns of *Taxon* which he had been editing for the last six years and would continue if the new Editor-in-chief so wished, to a degree. He had been very, very ably assisted in this with general nomenclature papers by Gerry Moore, for proposals to conserve and reject by John Wiersema and Scott Redhead, and for proposals to amend the *Code* by Nick Turland. Particularly in the area of general nomenclature papers he thought it would be useful to have a few other people doing part of the nomenclature editorial work, and he suggested that if anybody wanted to volunteer or to be considered as a volunteer, to please come and see him sometime in the next day or two. He noted that it [appointment as an editor] was not his decision, but that of the new Editor-in-chief, Rob Gradstein.

### Article 60 (continued)

**McNeill** reminded the Section that last night the decision was made that people would look at their notes and determine which of the proposals on orthography from Rijckevorsel they deemed not to be editorial and which of these that they felt would benefit from being included in the *Code.* He understood that what was written on the board was not a consensus but all those that people had identified, and he thought that different people had identified different ones. He suggested just starting at the beginning and working through. He expected the person who added a proposal to say why they felt it was desirable. [After some confusion about what was being discussed.] McNeill explained that if a proposal was not one being supported it would automatically fall by the motion before the Section. The motion was that everything be passed to the Editorial Committee except some items that were worth including that were not editorial. He emphasized that there was no need to discuss proposals that no-one wanted to see in the *Code*, what should be discussed were proposals that people did want to see in the *Code* and that were not editorial.

**West** wished to clarify what McNeill had said and asked if he meant that the Editorial Committee were not going to do anything with everything that was referred to them?

**McNeill** responded that that was not the case and apologized. What he said on Thursday, which he felt reflected the resolution from Prance, was that initially all the proposals go to the Editorial Committee, and they would look at all of them. However, an Editorial Committee had no power to include something that involved a change in meaning in the *Code*, and so that would mean that anything desirable in the proposals that involved a change in meaning could not be addressed, and he had recognized that people felt that that would be most unfortunate, and so there was an amendment, which Prance accepted, that the Section would consider today those proposals that were not editorial that individual members of the Section, or groups of members of the Section, felt would be good to have in the *Code*. So when he had said that we there was no need to discuss those that the Section did not particularly like, the reason for that was that if they were not accepted by the Section, the Editorial Committee, although ir would of course look at them, could take no action, and therefore effectively they did not need to be discussed. He wondered if that was clear and if it was acceptable?

**Nicolson** was not sure.

**Nic Lughadha** felt a little nervous about it because sometimes what seemed purely editorial could be another person’s substantive change in meaning. She thought that if people wanted to make clear that there were changes that they thought were not purely editorial and would be disadvantageous then they should have the option of rejecting certain of these proposals outright and not simply choosing between supporting a proposal or referring them to the Editorial Committee to decide whether to incorporate them or not. She wished to hear which, for instance, Zijlstra thought were not to be included. She did not think the Section should pass the lot through.

**Nicolson** suggested that perhaps discussion of the proposals should begin.

**McNeill** thought the Section should hear what other people had to say first.

**Nicolson** agreed and asked for comments.

**Gams** felt that it was principally editorial but it was a major step that Rijckevorsel was proposing to subdivide Art. 60 and restructure it. He gathered that the Section should formally empower the Editorial Committee to do this or not.

**McNeill** agreed, adding that he thought that something as important as that should well be discussed. He explained that these were not the type of proposals he was suggesting need not be discussed. They were the ones that really there was no support for in the Section and which were manifestly not editorial. He assured the Section that the proposals that were possibly editorial but might be controversial, which he thought Nic Lughadha was considering, would certainly be discussed.

If **Nicolson** understood correctly, the ones that should be discussed because they were not purely editorial were the ones listed on the board. He felt that the trick was to decide if that was acceptable and try to discuss them in order. The first one was Prop. G and he asked the Section if it was acceptable to proceed that way? He added that unfortunately the proposals on the board were not in sequence, but the first one was Art. 60 Prop. G.

**Prop. D** (11: 74: 61: 4), **E** (8: 74: 65: 4) and **F** (9: 73: 66: 4) were later ruled as referred to the **Editorial Committee.**

**Prop. G** (20: 65: 63: 4).

**Demoulin** requested an explanation of the difference between the line at the bottom and what was on the top.

**McNeill** thought it reflected people’s writing on the board, if he understood correctly. He wished to say that looking at Prop. G it did not seem at all editorial and he thought it was something the Editorial Committee would not touch, so unless somebody wanted to propose it should be included, he did not see any point in discussing it. He argued that it was definitely not editorial, and also not terribly helpful..

**Knapp** thought that even if it was not editorial and people wanted to vote “no” the Section should vote because that limited the work that had to be done on the Editorial Committee.

**McNeill** agreed.

**Zijlstra** thought that if the Section should only discuss what was wanted, then the bottom line of 60 G as referred etc. should be cancelled.

**McNeill** asked her to confirm that she did not want any of those?

**Zijlstra** only wanted two proposals [Art. 60 Prop. P and Rec. 60C Prop. K], and especially [not] that bottom line. She felt that those were the worst.

**McNeill** asked if anyone had any comments on the ones along the bottom line, that disagreed with Zijlstra? [Pause.] He just thought if it turned out that nobody else wanted the ones that Zijlstra did not want, that would be excellent advice for the Editorial Committee. He suggested that they could then be dealt with as a block.

**Demoulin** thought there were three opinions. There were people who would like to see everything referred to the Editorial Committee with the risk of potentially losing good things. There were people who would like to discuss everything; he thought that was the minority. And there were those who would like to only discuss things which [involved] a change in the *Code* that they liked and they would like to defend, and he thought that was the list. He also thought there were some people who would like to discuss things they did not like. In order not to keep discussing, he suggested that if there were people who wanted to discuss something because they did not like it they write the number there [on the board]; leave them five minutes and after that it was finished.

**McNeill** agreed that he was also suggesting something like that so that one way or another the Section would deal with all that had been written on the board, because they were the things that people had an interest in. He added that if, at the end of that time, there were other proposals that people wanted to discuss, they could raise them. He thought the discussion could go through them in a considered manner, but not necessarily one-by-one because Zijlstra had provided the information that she was opposed to the whole bottom line of proposals and if that was the case then if there was no one who supported them then, the Section could reject them all together because the proposal came from Zijlstra.

**Wieringa** supported some of them.

**McNeill** decided to take them one by one and asked if there was anybody to speak on Prop. G.

**Nicolson** felt that if there was no further discussion, one person was against it, and he ruled that it failed.

**McNeill** repeated that it was Art. 60 Prop. G and it was rejected. He explained that the plan was to take them in as closely sequential an order as possible and attempted to move on to Art. 60 Prop. J.

**Nicolson** apologized and asked if the Section would like to formally vote? [They did.]

**McNeill** felt that it was editorial. He was not sure what the problem was for Zijlstra as it was the one which said “For citation of a name or epithet not retaining the original spelling, see…”. He felt that either it was correct or it was not correct, and then it was editorial. If it was wrong, that did not mean the Editorial Committee were going to put in a note, it just meant that they *could* put it in. He wondered if there was there a problem with it being editorial?

**Zijlstra** was getting a bit confused with everything stumbling together. Her point was that the diaeresis was not mentioned. It was mentioned in the later proposal but not here and it was left out of the Article in which it always had been included as something that should not be changed. She felt that people might be confused to see the new text.

**McNeill** seriously suggested that there was no need to vote on the proposal at all because he failed to see how it was at all harmful. He thought it was possible that the Editorial Committee would not see any benefit in providing a reference, but: “For citation of a name or epithet not retaining the original spelling, see such and such”, either that was true or not, and it would either go in as being helpful or not; it did not seem to him to have any conceivable change to the *Code* one way or the other.

**Gereau** wished to mention a procedural matter, it seemed to him that a vote of “refer to Editorial Committee” or “reject” was in order, and those who did not want to see it there could throw it out if they wanted to.

**Nicolson** moved to a vote, asking for all those opposed to the proposal...

**McNeill** thought it was better to take Editorial Committee and then no; those in favour were referring it to the Editorial Committee and those in favour of rejecting it outright.

**Prop. G** was referred to the **Editorial Committee.**

**Prop. H** (11: 76: 61: 4) and **I** (7: 81: 61: 4) were ruled referred to the **Editorial Committee.**

**Prop. J** (9: 106: 25: 2) was ruled as **rejected.**

**Prop. K** (22: 63: 63: 4).

**Wieringa** thought the proposal was purely editorial, but still had an amendment for K, or it could be seen as a separate proposal. His proposal would slightly change the meaning of the *Code* so it should be voted on. He felt that Art. 60.6 was quite clear, except for one case. It clearly stated that an *a* became an *ae*, etc., except in case of *e*, *e* and *e* that would become *e*, or sometimes *ae.* He noted that there had been an e-mail discussion about this topic a few years ago in which it was said where *e* would apply, but no one was able to say in which [cases] an *ae* would apply. He had come across the example where Nicolas Halle had been commemorated about twenty times as *hallei* and once as *hallaei*. Clearly one of the two was wrong and should be corrected. But which was wrong? He felt that if it was impossible to tell in which case one of the two applied, it was better to make the rule clear and change Art. 60.6 to “*e*, *e* and *e* become *e*” and strike the rest: “or sometimes *ae*”.

**McNeill** checked that he meant delete the “or sometimes *ae*”. [He did.] McNeill felt it was a very relevant thing to discuss.

**Demoulin** strongly opposed the amendment. He argued that it was introducing one more standardization when there were already too many, and the *Code* was perfectly clear when the original spelling was respected.

**Rijckevorsel** remembered reading about the discussion in the Proceedings, and thought it was somewhere in the literature in the past 50 years about why it was.

**Zijlstra** felt that the proposal mixed up editorial in a quite unwanted move of the German “ss”...

**McNeill** interrupted to remind Zijlstra that the discussion was on the amendment that the words be struck out of the existing text. He felt it really was a separate motion, but decided to take it as an amendment. He kept the discussion to Wieringa’s proposal rather than the original wording.

**Wieringa** responded to Demoulin, who he felt had said, well, in this case you have to stay to the original spelling. But Wieringa argued that that was not what it said, it said that there were some cases where *ae* was acceptable. But his Example gave the same case, the same person being commemorated, done in two different ways. He argued that either one or the other was correct, but they could not both be correct, and the “original spelling” here gave two different options which lead to ambiguity. He suggested that maybe it would be possible to word it in such a way that it was clear in which case an *ae* had to be adopted.

**Demoulin** felt it was a completely different issue: the one of alternative spelling in the original publication was done somewhere in the *Code*. He exhorted the Section not to mix up things!

**McNeill** wished to comment to Demoulin. He felt that the issue was correctable as under Art. 60.1, errors under 60.6 were correctable, so the question was, were you saying in what way was it corrected?

**Demoulin** thought that it meant that in this case the two possibilities existed, the two possibilities were correct, then of course you did not correct it, you maintained the original spelling.

**McNeill** felt that whether it was regardless of the nature of the accent on the *e*; they could all alternatively be either *e* or *ae.* He wondered if that was what Demoulin was suggesting?

**Demoulin** responded that it did not matter and went on to say that it simply meant that if you had a name with an accent, and the people had decided it was better to make clearer that they want a particular kind of sound, so they used *ae*, then you should just leave it as they did it. He really did not see why people wanted to change what old botanists who knew their Latin well had done, while they admitted that we could spell *sylvestris* with an *i* or a *y*.

**Peter Jorgensen** pointed out that there was increasing use of databases, and databases did not have the capacity of looking beyond what was an *a* and what was an *ae.* He gave the example of sorting things and ending up having the same name in the list twice because they were spelled differently and argued that it was a headache to have two possible ways of spelling names. He was in favour of striking “, or sometimes *ae*” from 60.6 as amended from the floor.

**P. Wilson** wanted to point out that the origin of this Example [*e* becoming *ae*] was probably based on Linnaeus’s own name, and that people had latinized Halle’s name in the same way that Linne was latinized to Linnaeus, and that was possibly the origin of this Example.

**Demoulin** thought that the issue of databases was, again, irrelevant. He exclaimed that he did not understand! Alternative spellings were dealt with elsewhere.

**Rijckevorsel** wished to make a quick note that the proposal was about replacing an original spelling and these were very few cases of a name that had been dedicated to a person and had the signs which had to be transcribed and in general the first author who made the change would be followed unless there were big changes and grave reasons. He argued that it was a relatively simple matter.

**Glen** was not sure that in this stage in the twenty-first century the problem of exact spelling for databases was as critical as it had been. Certainly the more recent databases he had seen allowed queries saying “spelled something like this”, and they would pull out variants like the Halle example quite happily, retrieving both “*hallei*” and “*hallaei*. He felt that too much standardization was not needed.

**McNeill** highlighted that the problem was not finding the variants but knowing which was the one that should be correct.

**Demoulin** agreed that, of course, that was the problem, it was just deciding what the right spelling was, and in this case the correct spelling was the original spelling, and once you knew the correct spelling you put it in your database and...

**McNeill** interrupted to point out to Demoulin, that this was dealing with an Article in which the original spelling involved the diacritical sign, which was not permitted in Latin so it had to be corrected. He added that it was not only the name of the person being commemorated that had a diacritical sign, it was that the name was published with it. He then agreed that he saw what Demoulin meant and acknowledged that he had misread it.

**Nicolson** summarized that there was the problem of alternative spellings, not necessarily in the same name, but same epithets in different genera might be spelled differently. He asked for all those in favour of the amendment to strike out the “or sometimes” option. He thought it was very close.

[The **amendment** was **rejected.**]

**Kolterman** did not know whether to propose an amendment but the city where he lived had what looked like a *u* with an umlaut, but it was not, it was a *u* with a diaeresis over it and if it were to become *ue* it would make no sense at all. He explained that this occurred in Spanish and Portuguese after *g* and he believed in Portuguese after *q* as well. He did not know whether the Article should be amended but in those languages that particular point could not be followed. He gave the example of the *u* in Spanish and Portuguese (as in Mayaguez) which he emphasised must not become *ue* (in which case, for example, *mayaguezanus* should not be corrected to *mayagueezanus*).

**McNeill** noted that the diaeresis was permitted in a scientific name.

**Kolterman** responded that it did not indicate that the vowel was pronounced separately from the preceding vowel but that the vowel was pronounced following *g* and in some cases *q*.

**Nicolson** pointed out that that was a latter part of Prop. K; the diaeresis indicating it pronounced separately was *Isoetes* and so on, was permissible.

**Kolterman** reiterated that that was regarding pronunciation separate from the preceding vowel which was not the case in Spanish and Portuguese.

**Zijlstra** explained that her main problem with Prop. K. was that consonants were dealt with in 60.4 and vowels in 60.6. She wanted to know why replace the German *?* [from 60.4 to 60.6]? She felt it made things confusing.

**McNeill** thought it may be useful if anyone wished to support Prop. K, Zijlstra had spoken against it.

**Demoulin** noted that to him K was purely editorial, so felt he must have missed something if it was being discussed. He asked someone to point out what was not editorial in Prop. K?

**Zijlstra** thought it was a matter that was editorial, yet would be awful, and that was why she was against it as it made matters confusing for people if they no longer found all consonants in one Article and vowels in another.

**McNeill** thought that was a point that the Editorial Committee would take aboard.

**Gereau** felt it was exactly the same situation as with Prop. G. Zijlstra wished to have it voted not to go to the Editorial Committee; some other people might wish it to; he pointed out that the Section had agreed to such a vote on Prop. G and suggested another on Prop. K.

**Nicolson** summarized that it had been proposed that the Section vote directly on Prop. K. Up or down. Not to Editorial Committee.

**McNeill** corrected him that the suggestion was it should either be rejected or it should go the Editorial Committee.

**Nicolson** repeated that a vote “yes” would be to refer to Editorial Committee; a vote “no” would be to reject the proposal. He moved to a vote on… “I’ve forgotten where I was!” [Laughter.]

**McNeill** prompted him, “all in favour of Editorial Committee”.

**Nicolson** asked for all those in favour of referring Prop. K to Editorial Committee. He thought it was referred to the Editorial Committee, but it was very difficult.

**Prop. K** was referred to the **Editorial Committee.**

**Prop. L** (6: 77: 64: 4).

**McNeill** moved on to Prop. L, which he noted was editorial but it was substantial as Gams had pointed out in another context, so discussion might be desirable.

**Nicolson** asked if there was discussion? He understood it would be referred to Editorial Committee, but this was the opportunity to communicate what might be added or discussed.

**McNeill** replied that it should not go to the Editorial Committee really, that was what he thought Zijlstra had in mind.

**Demoulin** suggested maybe it would be easier to have the discussion Gams suggested now about whether the Article should be divided or not. He added that in his opinion it might be interesting to split the Article into orthography and typography, but splitting the orthography into various Articles with compounding personal name and so on was going too far.

**Gereau** felt it would be a surprise to everyone that he was agreeing with Demoulin. He felt the splitting into separate Articles, when different numbers in the same Articles, seemed an absolutely pointless editorial exercise that would take up time and add no clarity whatsoever. He did not wish it referred to the Editorial Committee, but wished it to die on the floor.

**Nicolson** explained that in this case a vote “yes” would be to the Editorial Committee; a vote “no” would be to reject the proposal.

**Prop. L** was **rejected.**

**Prop. M** (6: 77: 65: 4) was **withdrawn.**

**Prop. N** (6: 79: 63: 4).

**McNeill** moved onto Prop. N, pointing out that it clearly paralleled Prop. L.

Which **Nicolson** noted had been rejected.

**Wieringa** felt that if the Section discussed Prop. N, they should immediately also discuss Props W and P because these were more or less alternatives, all about 60.11. He added that there was one Note with Prop. N. He thought it was supposed to be the new Article on forming names and epithets based on personal names. However, it would include Art. 60.10, which was about apostrophes, and apostrophes may be present in personal names but also in geographical names, so it would not be entirely on personal names in that case if this was included. And if it would only talk about personal names, it would mean that there would no longer be a rule for apostrophes in geographical names, which would change the *Code* again.

**Zijlstra** had suggested it be rejected because it combined two quite different matters: in fact 60.10 concerned a very special kind of compound forms, with the apostrophe; and 60.11 concerned terminations. She felt they should not be put together.

**Nicolson** explained that a “yes” vote would be to refer to Editorial Committee; a “no” vote would be to reject the proposal.

**Prop. N** was **rejected.**

**Prop. O** (4: 77: 66: 4).

**Redhead** understood from reading the proposal that it was to be formed at the beginning of a new Article, which did not exist, so he saw no reason to have the proposal.

**Prop. O** was **rejected.**

**Prop. P** (20: 60: 67: 4).

**McNeill** had not necessarily scanned the board properly and completely, but thought the next one up there was Prop. U. [in fact it was Prop. P]

**McNeill** confirmed that an alternative proposal to Prop. P was referred to the Editorial Committee the day before and the Vice-Rapporteur’s suggestion was that maybe the same should be done with Prop. P.

**Turland** noted that it was basically an alternative of Rec. 60.C, Prop. A, which had already been referred to the Editorial Committee.

**Prop. P** was referred to the **Editorial Committee.**

**Prop. Q** (8: 58: 82: 4), **R** (7: 72: 69: 4), **S** (14: 65: 69: 4) and **T** (9: 89: 48: 4) were ruled referred to the **Editorial Committee.**

**Prop. U** (7: 89: 50: 4).

**McNeill** thought Prop. U came next, noting that it was linked to another proposal.

**Turland** confirmed that the Section had just voted on Art. 60. Prop. P and the next one up for discussion was Art. 60 Prop. U.

**Funk** asked if there was a problem with erasing the ones that had already been dealt with?

**Nicolson** replied, “Yes, no eraser!”

**Funk** Oh! [Laughter.]

[General chatter about which proposal on the board was indeed next, random letters being uttered, fairly Sesame Street-like atmosphere really.]

**Nicolson** commented, “Isn’t orthography fun?” [Laughter.]

[General chatter about which proposal was indeed next.]

**McNeill** understood that proceedings were now at Prop. U.

**Unknown Speaker** [off-microphone] thought it was linked to Prop. N that was rejected.

**Demoulin** felt it was editorial and it of course referred to the proposal that was rejected, but, or to Art. 60 in the case that it was rejected.

**Wieringa** did not think Prop. U was editorial as it would mean a change to the *Code*, because it made Rec. 60C.2 no longer a Recommendation, but it should be implied.

**McNeill** thought it was therefore very important that the mind of the Section be expressed. He added that for a long time 60C.1 had been correctable but 60C.2 had not.

**Rijckevorsel** agreed it was not an editorial manner and it would give 60C.2 just about the same status as 60C.1. At the moment he felt it seemed that 60C.1 was obligatory, mandatory, so if something did not conform to 60C.1 it had to be corrected, unless it was covered by 60C.2. But his issue was what happened if something *almost* fitted into 60C.2, but not quite? Then he felt it was in limbo; somewhere in between. It meant that it was not really covered by 60C.2, so it should be corrected. He explained that the proposal meant that something should be either under 60C.1 or it should be good Latin, and there were very few cases that would be affected as most of the people who were using Latin were using good Latin.

**Zijlstra** was afraid the proposal would be destabilizing; making people wonder if a text could be Latin and then thinking they should correct under 60C.2. She felt that would be disastrous. Although she did not have examples to hand she felt certain that there were cases that people would believe it would have to be corrected.

**Wiersema** thought there were definitely cases that would need to be corrected if it was changed. He knew of epithets based on Wislizenus, all of which were given intentionally latinized forms; others were not. He noted that the ones that were not would have to be corrected to conform to the latinized form.

**Rijckevorsel** disagreed, saying that the proposal meant that it would have to conform to either 60C.1 or 60C.2. For the example of Wislizenus he concluded you could make an epithet *wislizenii* or *wislizeni*, but it would mean that either of the Recommendations would have to be followed, and followed correctly.

**Nigel Taylor** pointed out that Wislizenus was already latinized, it was not *being* latinized by anybody; it was already in Latin form, which was one of the Germanic names of a family who latinized names, but it was not a botanical author that was latinizing the name, it was *already* Latin. So he did not think that it applied and you could not have variant endings for Wislizenus as it was a Latin word and therefore it must be treated as a Latin noun and its termination formed accordingly.

**Demoulin** was afraid that there was indeed a real eventual change involved here and that people may not be fully ready to vote on it because it was diluted into so many editorial things, and maybe it would be better to instruct the Editorial Committee to make things clearer regarding the relationship between 60C.1 and 60C.2. At the moment that was indicated by the reference “but see 60C.2”, that apparently some people had problems with, and he thought some change in wording of 60C.1, as had been proposed further down, might perhaps make things clearer. Even if he could sympathize with the proposal as it was, he could not see all the consequences and preferred to abstain. He concluded by saying, certainly it was desirable to have some instruction for the Editorial Committee to make it clearer what 60C.2 was in relation to 60C.1.

**McNeill** noted that the Editorial Committee already had that instruction and had to do it, because Rec. 60C Prop. A, which was addressing that very issue was approved.

**Nicolson** suggested that the comment would be to support referring this to Editorial Committee, not as something to be inserted in the *Code*, but to be analyzed and see if it could be incorporated in some way.

**Rijckevorsel** suggested it would make things clearer to take a quick look at Art. 60 Prop. V which was an example of the provision.

**Nicolson** mentioned that was *michaeli*... *miguelii*... He felt that perhaps the best way to proceed was to give a straight “yes” or “no”.

**McNeill** agreed and explained that if the Section referred it to the Editorial Committee that was “no” because there was a change to the *Code* and they could not make a change in the *Code* unless the Section actually passed it, so it would have to be approved in order for them to take action on it. He assured the Section that they would take action on clarifying the relationship between 60C.1 and 60C.2 because that had already been passed.

**C. Taylor** asked for a point of information. She wanted to know if this was made mandatory, what happened to epithets that fell in the last sentence in the third declension? For the group she worked in there were a number of species epithets like that. She wondered if they would have to be changed from *lugonis* to some other form? She felt that they did fall under it and she recommended not doing it, but it was permitted, and there were a number of them so that would require changes.

**Nicolson** explained that the vote would be to accept or to reject. If it was accepted the Editorial Committee would have to deal with it.

**Prop. U** was **rejected.**

**Prop. V** (9: 85: 53: 4) was ruled as **rejected** as it was an Example of Art. 60 Prop. U which was rejected.

**Prop. W** (8: 89: 49: 4) was referred to the **Editorial Committee.**

**Prop. X** (5: 87: 53: 4).

**McNeill** moved on to Prop. X, which was adding a new paragraph so it certainly had to be considered.

**Zijlstra** thought it could be a nice Recommendation on names to be published, but for existing names that were generally well-accepted in a certain spelling it might be harmful.

**Orchard**? [off-microphone] asked what “delatinization” was.

**Nicolson** responded that changing Linnaeus to Linne would be a delatinization.

**Orchard**? wondered if there were any other examples?

**Nicolson** asked for any other examples of desalin-, he corrected himself to delatinization? [Laughter.]

**McNeill** wondered if Zijlstra was proposing that it be treated as a Recommendation as an amendment. [She was not.]

**Nicolson** proposed that a “yes” vote would be to refer to Editorial Committee; a “no” vote would be to reject.

**Prop. X** was **rejected.**

**Prop. Y** (5: 94: 47: 4)

**McNeill** thought there would only be a Note [into which the wording of the proposal could be inserted] had Prop. X been accepted and sought Rijckevorsel’s confirmation.

**Rijckevorsel** also thought so

**McNeill** confirmed that the proposal could have no standing and was *de facto* **withdrawn.** [noted as rej. auto. in Taxon 54(4).].

**Prop. Z** (5: 95: 46: 4), **AA** (9: 89: 49: 4), **BB** (14: 86: 45: 4), **CC** (10: 88: 47: 4) and **DD** (8: 86: 52: 4) were ruled referred to the **Editorial Committee.**

**Prop. EE** (11: 85: 50: 4).

**McNeill** moved to the next proposal noted which was double “E”, Prop. EE [which he went on to pronounce “eh, eh”. – Laughter.]

**Nicolson** exclaimed, “That was unaspirated!” [More laughter.]

**McNeill** explained that his “ee” was not how everybody pronounced the letter.

**Gams** outlined that in the proposal and in subsequent ones the proposer tried to make a differentiation between given names and surnames. He felt that pushed standardization too far. He did not want to see the latinization of a given name ruled differently from that of the surname.

**P. Hoffmann** added that it was also in many cases impossible, or not so easy, to say what was what and many given names could be surnames and so on, giving the examples of Chinese, Indonesian, US American. She was also against the proposal and felt the Section should vote it down.

**Nicolson** thought the question was not necessarily to refer to Editorial Committee, so asked the Section of they wished to vote it straight up, straight down. [They did.]

**Prop. EE** was **rejected.**

**Prop. FF** (10: 85: 50: 4).

**McNeill** thought Prop. FF was the Example to the previous proposal, and presumed therefore that it automatically dropped.

**Unknown Speaker** [inaudible voice off-microphone] said it was a different Example.

**McNeill** apologized.

**Demoulin** thought it was a good Example to go to the Editorial Committee.

**P. Wilson** disagreed, saying it was not a good Example. He explained that the Wollemi pine was deliberately named *nobilis* as a kind of a double meaning. It was named after the collector, Noble, and was also named to indicate it was a noble tree. So there was an intent, he was not sure whether it was actually explicit in the protologue, but the intent was to have that double meaning in the name. So he was not sure it was a good Example for that reason.

**McNeill** commented, not having read the protologue, that he thought it was critical what was in the protologue. If there was no suggestion of the pun in the protologue, [P. Wilson: None] then it may be one to those who know, but on paper it would probably be quite a good Example.

**P. Wilson** had asked his colleague Barbara Briggs if she recalled, but he could definitely remember it being spoken round the herbarium. He asked if it was critical to whether it went in?

**McNeill** confirmed it most certainly was.

**Demoulin** corrected “good” to “interesting” Example [Laughter.]. He wished to point out that when an Example was referred to the Editorial Committee it did not mean it was going to be printed the way it was, and his experience was the Editorial Committee had always checked the protologue before including an Example.

**McNeill** noted that a number of Examples presented to them, and even published in the Synopsis and so forth were manifestly wrong; an undesirably high number, probably about half. Sometimes it was still possible to use them, but not exactly as phrased.

**Rijckevorsel** confirmed that the protologue only spoke of the person so there was no reference whatsoever to the pattern [of tree]. Indeed it was an interesting Example rather than a good one and he felt it may need looking at, depending on what other proposals were passed, since that was rather critical.

**McNeill** asked permission to intrude with a request and that arose from that discussion about Examples. He did not think he had made the announcement before, but the Editorial Committee always welcomed suggestions of Examples in the *Code* particularly of course in areas where it was felt that there were inadequate Examples or insufficient Examples, and these should be sent either to him or to Nick Turland, electronically was the obvious way, sometime in the next couple of months.

**Turland** added that a scan or a photocopy of the protologue would help a lot.

**Printzen** did not really see why the Example should go in the *Code*, because current discussion was dealing with Prop. FF now, and it said “Add an Example to the Note of Prop. 139”. Prop. 139 was Prop. CC; which said add a Note to the paragraph of Prop. 134; 134 was Prop. X and that was voted down.

**Nicolson** feigned an inability to understand the problem! [Laughter.]

**McNeill** felt that the point was made by one of the speakers that it would be put in an appropriate place *if* there were one.

**Nicolson** summarized that Prop. FF was basically an Example and could be referred to the Editorial Committee or voted down. He deemed it was referred to Editorial Committee, but noted it was a hard call, and could see it was controversial.

**Prop. FF** was referred to the **Editorial Committee.**

**Prop. GG** (7: 93: 45: 4) was ruled referred to the **Editorial Committee.**

**Prop. HH** (11: 100: 37: 4).

**McNeill** moved to Prop. HH.

**Gams** stated this was about the barbarian latinization, derivation, of names like *hieronymusii* and so on and strongly recommended that such derivations be avoided. He added that the proposal would sanction barbaric derivations like *martiusii* (instead of *martii*), which should certainly be avoided.

**Demoulin** did not think there was enough information in the proposal to rule on the issue, and in his opinion the *Code* as it was would allow the two kinds of formation and there were a number of Examples that could be referred to the Editorial Committee to see if any of those were really in agreement with the *Code* and would be useful to add.

**Nicolson** explained that a “yes” vote would be to refer to Editorial Committee, a “no” vote would be to drop it.

**Prop. HH** was **rejected.**

**Prop. II** (10: 103: 333: 3) and **JJ** (9: 89: 48: 4) were ruled referred to the **Editorial Committee.**

**Prop. KK** (8: 94: 43: 4), **LL** (10: 91: 46: 4), **MM** (7: 93: 45: 4) and **NN** (9: 89: 46: 4) were discussed as a group with **PP** (10: 89: 45: 4). **Prop. OO** (8: 92: 44: 4) was ruled referred to the **Editorial Committee.**

**McNeill** moved to Prop. KK which seemed to again be making a distinction between given names and surnames, which had already been addressed.

**Glen** wondered if he was being very stupid asking if it perhaps depended on Prop. X, which had already been voted down?

**Malecot** added the information that all the remaining proposals [to be studied, i.e.] KK, LL, PP, MM, NN were all related either directly or indirectly to Prop. X [that was defeated].

**McNeill** asked if the proposer disagreed with the statement? [The proposer did not think so.] McNeill thought it was true that Prop. KK addressed the same issue and thought Prop. LL was similar, but perhaps not quite.

**Zijlstra** suggested that some proposals in several next Articles might be referred to the Editorial Committee if the explanation why it should be that way could be left out. In this KK case, however, she felt it was so clearly an illustration of Prop. X that was rejected, that it should be rejected.

**Demoulin** thought that from Props KK to NN they were related because they were presented in a philosophy that several speakers had opposed and he agreed with them to make distinction between given name and family names. Despite the fact that he thought some of the individual Examples were good, some were already in the *Code* anyway, and others could be added, he thought the rule set reflected a philosophy that he got the general feeling was not acceptable to the Section. He suggested that the Editorial Committee could probably pick up the interesting things, but the rule set was not acceptable as it was at the moment.

**McNeill** agreed that it would be perfectly straightforward to vote down the whole set, because of the preamble versus the presumptions behind them, and as there were already some of those Examples in the *Code* and there was nothing to stop the Editorial Committee picking up appropriate Examples that would illustrate the *existing* wording of the *Code*, but of course not Examples that illustrated the wording that had been rejected.

**Nicolson** pointed out that one of the purposes of the discussion was to be sure that the Section did not overlook something that the Editorial Committee should consider.

**McNeill** agreed it would be very valuable to be sure that good changes in the *Code* were not just forgotten about by referring to the Editorial Committee who were powerless to make those good changes.

**Nicolson** asked the Section if they were willing to vote as a block?

**McNeill** listed the relevant proposals as all double K, L, M, N, and P [i.e. KK, LL, MM, NN, PP]

**Nicolson** reiterated that the plan was to vote on them as a block and either refer them to the Editorial Committee or reject.

**Demoulin** asked which this applied to

**Nicolson** replied L, M, N, P.

**McNeill** corrected him that in each case it was the double letter of K, L, M, N, and P.

**Nicolson** agreed and clarified that it concerned KK, LL, MM, NN... PP [Laughter.]. He added that it must be about break time, come to think of it! [More laughter.] in the absence of screaming “No’s” he asked for a vote of LL through everything except OO—Uh–oh! [Laughter] with a “yes” vote to refer to that Editorial Committee or “no” to reject.

**Props KK, LL, MM, NN** and **PP** were **rejected.**

**McNeill** noted that there were three proposals remaining on the board for discussion and wondered aloud if they could be done before the break? [Voices: Coffee! Coffee!] He concluded that the Section participants wanted coffee.

**Nicolson** agreed that it everyone needed to go for... coffee! [Laughter.]

**McNeill** quipped, “It’s all this PP isn’t it?”

**Prop. QQ** (9: 89: 46: 4) and **RR** (9: 90: 46: 4) were ruled referred to the **Editorial Committee.**

### Recommendation 60B

**Prop. A** (23: 81: 41: 4), **B** (33: 66: 47: 4), **C** (9: 75: 62: 4), **D** (12: 76: 58: 4), **E** (16: 68: 62: 4) and **F** (7: 78: 61: 4) were ruled referred to the **Editorial Committee.**

### Recommendation 60C

[*The following debate, pertaining to Rec. 60C Prop. A and Prop. B, relating to orthography took place during the Sixth Session on Thursday afternoon*.]

**Prop. A** (36: 31: 74: 1).

**McNeill** introduced Rec. 60C Prop. A, from Brummitt. He reported that 74 Editorial Committee preliminary mail votes reflected the alternative suggestion by the Rapporteurs. He noted that the proposal aimed to address the apparent conflict between Rec. 60C.1 which was mandated by Art. 60.11 and Rec. 60C.2, which was not. The Rapporteurs thought that the suggested changes might help to resolve the confusion but that a change to Art. 60.11, similar to that in Art. 60 Prop. B but with some rewording, would be a better option. He concluded that this suggestion seemed to have received support in the mail vote.

**Brummitt** added that it was a rather strange thing that he stumbled on, rather by accident. Art. 60C.1(b) stated that if a personal name ended in a consonant you added -*ii* for the genitive form. So this would mandate that Linnaeus, for example, had to be *linnaeusii*. On the other hand 60C.2, did not actually use Linnaeus, it would recommend *linnaei*. So that there was a conflict between the two. He concluded that because 60C.1 was obligatory and 60C.2 was not, it obligated adoption of *linnaeusii*.

**McNeill** responded that the Rapporteurs’ point was that it did not, because if it was of that form then 60C.2 took priority in the sense that that form was the correct form and it was not correctable. But as Brummitt rightly pointed out, it was not clear in Art. 60.11 and the issue had to be addressed by some change in the wording, on that they agreed, but they thought it was perhaps better actually in the Article than where it was being suggested. He thought they had suggested that some of the wording in Art. 60 Prop. P, one of Rijckevorsel proposals might help.

**Brummitt** summed up that there was some confusion and if the Editorial Committee could sort it out, he would be happy. He did not want to argue the minutiae of it.

**K. Wilson** pointed out that, Brummitt said that the Linnaean Example was not in Rec. 60C.2 but it actually was given there, so that Example was covered.

**Nicolson** suggested that a “yes” vote would be to refer it to the Editorial Committee and a “no” vote was to defeat.

**Prop. A** was referred to the **Editorial Committee.**

**Prop. B** (97: 38: 15: 1).

**McNeill** introduced Rec. 60C Prop. B which related to Art. 60C.2 which dealt with well-established personal names already in Greek and Latin or possessing a well-established Latin form and, among those, was *murielae*, and the proposer was proposing that this be deleted, arguing that Muriel was a modern name. He felt that the matter of given names as opposed to surnames had a long standing tradition of being treated as Latin. The question the Section had to decide was, having established this in two successive *Codes* should it be changed back or not. The argument of the proposer was that Muriel was a relatively modern name and therefore its inclusion was inappropriate. He added that it was obviously put in there to establish what was, certainly in the 19th century, quite customary for most prenames to be latinized more obviously than a surname.

**Nicolson** recollected that it was Stearn who put it in.

**Demoulin** did not remember but that was going to be his question. He knew he had not introduced it, but thought it was somebody who knew this best and he heard it must have been Stearn. He would have said it might have been Greuter but anyway it was proposed by someone who knew. He felt it was a rather futile discussion because if it was removed you would form *murielae* anyway.

**McNeill** thought that the issue was a real one. It involved a particular name of a bamboo that had bounced back and forth on the basis of this and the question really was, was it correct for it to be formed this way or could it be corrected under Art. 60C.1. But this was not in there and if it was treated as a personal name in Art. 60.1 it could be corrected (standardized) otherwise it would retain the *murielae* form.

**Rijckevorsel** had looked it at from several different angles and, depending on how you approached it he felt you could build several different cases and none were really convincing. If you looked at botanical custom then, it really depended on the question of the formulation of the Recommendation and it would favour leaving it in, also it was in the *Code* so its easiest to leave it in.

**Veldkamp** noted that the bamboo which was called *murielae* had his personal interest. He had looked Muriel up according to a Dutch book on children’s names and its latinization was *murielae*. He felt that the argument that the name was made up in the 19th century was false.

**Wiersema** cleared up the matter of who originally proposed it, stating that it was discussed in an amendment from the floor at the St. Louis Congress to a proposal by Stearn, who put forth the particular Example and that it was discussed in some detail in *Englera* [30: 211–217. 2000].

**McNeill** suggested that it was an attempt by the proposer to turn the clock back and the thrust of his arguments had been contradicted by Veldkamp.

**P. Wilson** wanted to make a point that was a bit lateral. He felt that the Examples were for interpretation of how you should spell other epithets based on women’s first names and raised the case of an *Acacia* called *mabellae*. It was named after a woman named Mabell with a double ll, *mabellae*. They wondered how much latitude should there be to play fast and loose with the epithet that people had chosen? The word *bella* was obviously a word with a Latin root and the author of the name obviously chose to form the epithet that way. But the epithet appeared in the literature as -*lliae*, -*lae*, -*liae* and there had to be some way, based on these sort of Examples, to come a decision whether the epithet could be corrected or not. He felt that the Examples must serve as some kind of a guide for people trying to make those decisions.

**Prop. B** was **rejected.**

[*Here the record reverts to the actual sequence of events*.]

**Prop. C** (9: 79: 54: 6), **D** (8: 78: 56: 6), **E** (7: 79: 55: 6), **F** (7: 78: 55: 6), **G** (30: 72: 55: 6), **H** (10: 75: 50: 14) and **I** (10: 74: 50: 14) were ruled referred to the **Editorial Committee.**

**Prop. J** (7: 76: 51: 13).

**McNeill** turned to Rec. 60C Prop. J.

**Demoulin** did not think it was adequate and certainly did not reflect the present *Code*. Camus had nothing to do, he believed, with Latin, so it was one thing, while Magnus was a Latin word, so he felt the two things should not be mixed up, and would not vote Editorial Committee but “no” to the proposal.

**Gams** was entirely on Demoulin’s side and did not feel the need to add anything. Then he added that he would certainly not defend the revision of *magnusii*, but stay with *magni* as a genitive.

**Veldkamp** thought it could not say that correct Latin had to be written as it would be a problem for many, and personally he preferred to have *magni* instead of *magnusii*. He stated that it was not classical training. He considered it fortunate that correct Latin was not required!

**Gandhi** opposed the proposal, giving the reason that even in 1990 there was a discussion as to whether it was really an ancient Latin name or a modern Latin name. He believed that at the time they had contacted Nicolson whether to take that personal name as modern or ancient. If that was the case he felt it would not be easy for everyone to determine whether a particular Latin name was modern Latin name or ancient Latin name.

**Nicolson** explained that a “yes” vote would refer to Editorial Committee a “no” vote would be to reject.

**Prop. J** was **rejected.**

**Prop. K** (25: 72: 47: 10), **L** (8: 74: 58: 8), **M** (13: 72: 54: 8), **N** (7: 76: 55: 8), **O** (10: 76: 53: 8), **P** (6: 85: 48: 8), **Q** (7: 87: 45: 8) and **R** (7: 87: 45: 8) were ruled referred to the **Editorial Committee.**

**Prop. S** (7: 86: 45: 9).

**Demoulin** wanted to raise the proposal after what was done the day before with the very first proposal [Art. 60 Prop. A] that was going to reinforce some automatic standardization some of which he considered highly unfortunate. It could be an interesting way to give more clarity, more emphasis, and allow in the future to perhaps add some category of names in this part of Rec. 60C, which he reminded the Section was the most difficult of the whole orthography section. At the moment 60C.2 dealt simultaneously with names already in Latin or possessing a well-established latinized form. This would give more emphasis to the names with the well-established latinized form, and he believed this category should be a safety valve to avoid some of the very unfortunate consequences of automatic application of some of the rules of 60C.1. During the night, the ghost of Desmazieres appeared to him and gave him some indication of why there always had been a trouble with that kind of name and also asked him to try to avoid the horrible *desmazieresii*. Given the general feeling of the Section against orthography, he chose not to propose what he thought should be the correct amendment to 60C now, leaving it to the next Congress, but he reported that for the last 20 years there had been fighting on those French names in -*ere* or -*eres* and for what he thought was a rather silly reason. He felt it was perhaps useful to give more emphasis to those classically latinized names at the moment, and thought Prop. S was a good way of doing that, and the Examples were not very different from what was already, may be a few were interesting and good, and suggested that perhaps the Section should vote on those Examples after discussing Prop. S.

**McNeill** wished to confirm he was speaking in favour of accepting Prop. S as opposed to sending it to the Editorial Committee?

**Demoulin** responded that he had done what the Rapporteur had asked, write down what he thought should be defended.

**McNeill**, before people started asking the obvious questions about what a “well-known botanist” was, noted that this would be addressed editorially; something as vague as that would not appear in the *Code*.

**Demoulin** felt that some of the sections of the *Code* had borderline cases for which, more and more, including at this Congress, the only way out was to refer the case to the General Committee. He was not going to propose that we do that at this moment with orthography, but perhaps if it had been thought about in the past some of the present problems might have been avoided.

**Nicolson** started to explain that a “yes” vote would be to refer to Editorial...

**McNeill** interrupted to correct him that a “yes” vote would be in favour because it was a new Recommendation in the *Code*, but it was only a Recommendation.

**Nicolson** repeated that a “yes” vote would mean it would go into the *Code*.

**McNeill** pointed out not necessarily with some of the ambiguous wording. He felt that the core of it was non-ambiguous but there was some extraneous wording.

**Nicolson** continued that a “no” vote would be to reject.

**Prop. S** was **accepted.**

**Prop. T** (6: 91: 37: 14).

**McNeill** continued that Prop. T was an Example to the previous proposal, and suggested it could be referred to the Editorial Committee. He noted that there was nodding in the Section.

**Gams** felt that the proposal contained some inconsistencies in that the examples of *bellonis* and *brunonis* were not Latin, but Italian names derived from Latin. They could be latinized: “Bella” meaning “the beautiful” was *bellus* in Latin; “Bruno” meaning “brown” was *brunneus* in Latin, so he felt that if you really wanted to latinize those names you should do it in another way. He added that, of course, names derived as proposed need not be corrected.

**Rijckevorsel** believed that *brunonis* was an extremely well-established Latin form going back to about the fifth century and there was a well-known writer just after the year 1000 who wrote about the Saxon Wars, so as a Latin form it was extremely well established. What exactly it meant was, he felt, a little ambiguous, but volumes could be written about it and it was extremely well established as Latin. The author Robert Brown was also extremely well known and there were lots and lots of epithets named after him, so he thought you could argue quite a bit about the exact linguistic aspects, but the fact was it was well established.

**Gams** clarified that he was not pleading for an accurate latinization of these names.

**McNeill** noted that the Editorial Committee would, of course, only include in the Example those cases that seemed to represent the Recommendation.

**C. Taylor** had a wider interest in the problem. In another part of the *Code* (Rec. 60C.2) it was recommended against using third declension, and here it recommended using it. She wondered if this was useful?

**Demoulin** responded first to Gams, saying that he thought that it would be nice if Gams and anybody who had information on Examples, whether this one or another, would make a short note for the Editorial Committee that they thought some of the Example might not be appropriate. His second comment was about the name in Prop. S. He noted it was not the first time it had been discussed and that there certainly should be some clarification, but the situation was that there was a general Recommendation not to use them —not one that was turned into a rule by some back door. He felt they definitely were admissible and not to be corrected, and in his opinion there were some cases where they would present a real tradition like *brunonis* that he agreed was a regular genitive of a very old saint and could, in fact, be recommended exceptions.

**Nicolson** asked if he was speaking in support of the proposal?

**Demoulin** was and had no problem with the set of Examples, except maybe, as Gams had said, *bellonis*, which might need to be elaborated that some of those genitives which were recommended against but not forbidden. He reiterated the need for some documentation from Gams for that.

**McNeill** assured the Section that the Editorial Committee would certainly make clear that the Recommendations were not in conflict, and there was clarification of where one applied and one did not.

**Mabberley** added a footnote on Robert Brown about whom he professed to know a little. He reported that the specific epithets were all derived originally from the generic name *Brunonia*, which was deliberately used to prevent there being a homonym because *Brownia* already existed; James Edward Smith—as the proposer had pointed out—deliberately chose the Modern Latin name, “Bruno”, as a replacement for Brown, hence *Brunonia* and then *brunonis*, *brunonianus*, etc. He felt it was a very good Example and hoped it would stay.

**Nicolson** explained that a “yes” vote would be to refer the Editorial Committee and a “no” vote would be to reject.

**Prop. T** was referred to the **Editorial Committee.**

**Prop. U** (6: 91: 37: 14), **V** (5: 94: 34: 14), **W** (4: 89: 39: 14), **X** (6: 94: 32: 15), **Y** (10: 90: 33: 14), **Z** (8: 92: 34: 14), **AA** (4: 90: 37: 14), **BB** (7: 91: 35: 14), **CC** (7: 92: 34: 14), **DD** (7: 92: 34: 14), **EE** (7: 88: 38: 14), **FF** (7: 91: 35: 14), **GG** (6: 92: 33: 14), **HH** (6: 90: 37: 14), **II** (7: 89: 37: 14), **JJ** (7: 86: 39: 14) and **KK** (7: 87: 39: 14) were ruled referred to the **Editorial Committee.**

### Recommendation 60D

**Prop. A** (50: 73: 25: 4) and **B** (45: 77: 25: 4) were ruled referred to the **Editorial Committee.**

### Recommendation 60E

**Prop. A** (10: 76: 59: 4), **B** (22: 65: 57: 4) and **C** (17: 97: 30: 4) were ruled referred to the **Editorial Committee.**

### Recommendation 60F

[*The following debate, pertaining to Rec. 60F Prop. A relating to orthography took place during the Sixth Session on Thursday afternoon*.]

**Prop. A** (61: 71: 11: 2).

**McNeill** introduced Rec. 60F Prop. A from Brummitt, describing it as something the Section could get their teeth into. He explained that the main use of the Recommendation was to explain why capital letters were found as the initial in epithets of specific names. It was the one that said that they should be written with an initial lower-case letter, but indicated when an initial capital letter might appear. The idea was that all this discussion about names derived from the names of persons, or vernacular, or non-Latin names, or former generic names being capitalized should be deleted.

**Brummitt** added that it was pretty well established practice to always decapitalize specific epithets, even if they were personal epithets. He wanted to see that as a strong Recommendation in the *Code*, not diluted. He acknowledged that it was only a Recommendation so, of course, you could do what you like, but it was a clear message. To give an example he read a newspaper article about *Wollemia nobilis*, which was so full of errors that he felt like writing a letter to the editor immediately. One of the points he would have made was that he put capital N for *nobilis*. But if you do take it up with an editor, if they have the *Code* with them, which he thought they probably did not [Laughter], they could always come back and say but look... He noted that it applied to a lot of horticulture literature as well. He much preferred to see a clear direction that specific epithets should be decapitalized.

Without questioning Brummitt’s Recommendation, **McNeill** thought in the case of *nobilis*, that it did not fall into any of the categories which might be capitalized.

**Nicolson** pointed out that time was running down and the electricity would be turned off before inviting further discussion.

**Zijlstra** suggested a small change to Rec. 60F.1; to put it into the past tense, to explain that it was not current practice but it was why people did so in the past and if they were desiring to use initial capital letters, where the epithets were directly derived from.

**Nicolson** asked if it was a proposed amendment? [It was and it was **seconded.**]

**Knapp** felt that sort of change could go in an on-line version of how to use the *Code* because introducing the history of why things happened into the *Code* meant the *Code* was going to get longer and longer and longer. She felt that was just the kind of thing that needed to go into some sort of an on-line easy explanation of why we do the things the way we do.

**Nicolson** asked if the Section were ready to vote?

**McNeill** clarified that the vote was on Zijlstra’s amendment to make it a historical portion rather than the original.

**Demoulin** disagreed with the idea that historical explanations should not be in the *Code*. He thought it was necessary to have explanatory things in the *Code* and historical details could be explanatory and were useful. Here he thought there should be some way to, at the same time explain why people may need those capital letters and recommend against them. He believed Brummitt should find a new formulation for tomorrow.

**McNeill** pointed out that the formulation was Zijlstra’s and she was proposing to keep it but modify it.

**Zijlstra** was proposing it as an amendment to Brummitt’s proposal but as there seemed little support she withdrew it and would vote against the proposal.

**Prop. A** was **accepted.**

[*Here the record reverts to the actual sequence of events*.]

**Prop. B** (7: 83: 51: 4) was ruled referred to the **Editorial Committee.**

### Article 61

**Prop. A** (8: 67: 71: 4), **B** (6: 72: 67: 4), **C** (5: 70: 68: 4), **D** (5: 73: 65: 4), **E** (5: 71: 67: 4), **F** (8: 70: 67: 4), **G** (9: 66: 70: 4), **H** (5: 73: 67: 4), **I** (4: 71: 70: 4), **J** (6: 70: 69: 4), **K** (4: 76: 65: 4), **L** (6: 72: 69: 4), **M** (3: 70: 72: 4), **N** (6: 74: 65: 4), **O** (3: 71: 71: 4) and **P** (6: 70: 69: 4) were ruled referred to the **Editorial Committee.**

[*Short discussion of Rec. 21B Prop. A to extend the Recommendation to cover subgeneric or sectional epithets occurred here and has been moved to the Third Session on Wednesday morning following the sequence of the Code*. *Short discussion of Gen. Prop. F, to make a blanket replacement of “forming” with “coining” occurred here and has been moved, similarly, to the First Session on Tuesday morning.*]

**Prance’s Motion**

**McNeill** asked if there was any further discussion on the proposals on orthography? If not the proposal made yesterday afternoon by Prance then kicked in; and all the others would go to the Editorial Committee, with the clear understanding that where they changed the *Code* the Editorial Committee would do nothing and the Editorial Committee would use its good judgment on the others. He repeated that this was the point regarding those proposals specifically raised and these that would not fall under the Prance blanket proposal made the day before and seconded.

**Nicolson** moved to a vote on Prance’s proposal, that all other orthography proposals be referred to the Editorial Committee.

**Prance’s Motion** was **accepted** and the remaining orthography proposals were **referred to the Editorial Committee.**

[*Discussion of proposals relating to Art. 59 occurred here and has been moved to the Sixth Session on Thursday afternoon following the sequence of the* Code.]

### Article 62

**Prop. A** (133: 12: 6: 0).

**McNeill** moved on to Art. 62. Prop. A, which was dealing with the termination –*botrys*. He reported that it had received strong support in the mail ballot.

**David** explained the basis for the proposal was really just a tidying up exercise. It reflected a discrepancy in application and in the use of the termination -*botrys*. He noted that it differed between the strictly botanical community and the mycological community in that the botanical community had generally adopted the classically correct masculine gender for the termination, whereas the mycological community had adopted the feminine gender for the termination. This had implications in that it probably affected the mycologists more in terms of the generic names that would have to change, but based on the numbers of species affected, which was in the end what the judgment was made on, more species epithets would be changed if one had to go to the feminine gender.

**Gams** reported that the Committee for Fungi had voted on the question and supported it with 11 against 3 “no”. When he sent out the ballot he was not aware of the large number of epithets that would be affected, mainly in the two genera *Arthrobotrys*, with 46 epithets, and *Stachybotrys* with about 70. As *Stachybotrys* was a really important, imperfect genus he would rather not support the proposal and hoped that the other botanists would find a mode to conserve masculine use for the higher plants. The mycologists who were using feminine gender for these genera were obviously influenced by the genus *Botrytis*, which obviously was feminine, and these other genera mentioned were coined in analogy and because of the similarity with *Botrytis*.

**Demoulin** had discussed the issue the day before and felt that, of course, mycologists were sorry about *Stachybotrys* and *Arthrobotrys*, but he had voted “yes” and he maintained his “yes” vote perhaps because he was sensitive to the linguistic correction, and thought that maybe if the mycologists felt that the gender should be retained and *Arthrobotrys* and especially *Stachybotrys*, which was really an important and medically important genus, then they could make a proposal for conservation of the gender. He added that, just like you can conserve an orthography, he believed you could conserve a gender. He concluded that since David was a mycologist, did the searching, considered the general usage and the correctness were on the side of masculine, he thought the Section should pass the proposal, and eventually Gams should make up a proposal for conservation of the gender of *Stachybotrys*.

**Prop. A** was **accepted.**

### Division III

**Prop. A** (46: 98: 1: 4).

**McNeill** moved to Division III Prop. A that was dealing with institutional votes and how they should be allocated as Division III described how that was and the alternative was on the board.

**Kolterman** wished to speak, hopefully briefly, to the proposal. He was representing himself and also the Red de Herbarios de Mesoamerica y el Caribe (Mesoamerican and Caribbean Herbarium Network). The President Mireya Correa, was also present in the Nomenclature Section, and he suggested she could offer corrections in a moment. He noted that they represented the interests of several dozen herbaria in southern Mexico, to Panama and the Antilles. Among other goals they were committed to developing the knowledge and skills of their herbarium personnel, including of course the area of plant nomenclature. Correspondingly they sought appropriate participation in the processes outlined in Division III of the ICBN. As was the case everywhere in the world, some of their region’s herbaria had been unstable but others had enjoyed decades of stability and activity. Conservation and growth of the collections, local and international use, databasing, collaboration with botanists within and outside their region, etc. They understood that nine herbaria in their region had a total of 12 institutional votes, while some active institutions had not been assigned institutional votes under Division III.4.(b)(2). They had understood at St. Louis, apparently incorrectly, that the criteria for the assignment of institutional votes would be made public and that institutions would be able to petition for incorporation in the list; however, this did not happen. Prop. A would offer them the possibility of greater participation, though perhaps largely through the delegation of votes, especially for Congresses in places such as Asia, Africa or the Pacific. Alternatively, some other procedure might be developed to allow for a more inclusive and dynamic list of institutional votes. He had the following specific comments or Recommendations regarding Prop. A:

First he suggested that something like “institutional votes.” should be added at the beginning of the proposed new text to maintain parallelism between (b)(2) and (b)(1). Second, it seemed to him that one year was probably too little time, especially as, in their experience, many institutions did not seem to maintain their listings in Index Herbariorum up-to-date. He suggested two years or even longer as being preferable. And finally, in the second sentence where it said “To receive its votes”, he thought that should be changed to something like “To be eligible to vote”, because as he understood it the votes were only received upon registration at the Nomenclature Section. Personally he was not so concerned about the Rapporteurs’ comment that possibly their share of the institutional votes might actually decrease if this proposal was approved. What they were seeking was the opportunity to participate, to share their concerns, especially regarding proposals that might have a particular impact in their region, and to learn from the process. Other institutions elsewhere should of course also have the same opportunity; most of them were from Europe and North America, and should have the greater number of votes in any case.

**McNeill** wished to make one small point. He thought it was said in St. Louis, but it was certainly a fact, that the list of institutional votes was indeed public and was published within a year of the Congress, in the volume of *Englera*. The full list of institutional votes was part of the proceedings; it also included, indicated by an asterisk, those institutions that were represented, and this had been true in every single Congress since about Leningrad and perhaps even before. The Bureau this time sought to look at where it saw some anomalies, and because of the interest and concern in Latin America, those Latin American botanists who publicly expressed interest—that was by the authors of this proposal and a number of others who had written a paper in *Taxon* on the topic about four or five years ago—were all individually consultedon the list for Latin America—being provided not with the total list but that of Latin American institutions. He was sorry to say that the response was actually very small, but they had made some very minor adjustments on the basis of the recommendations received. He was totally at one with the idea behind the proposal that there should be good and adequate representation from all parts of the world; not just Latin America, but everywhere. However, he would be extremely unhappy about the details of the specific proposal being accepted. For one thing, the cost of mailing every single institution, not many of which were on e-mail, was quite substantial. He thought that by her own personal experience, Holmgren, who compiled Index Herbariorum, could advise on who had not replied to her—the so-called dead herbaria—but there was nothing in this proposal that said that Holmgren’s view of what was an active and what was a dead herbarium, however sound it may be, should be invoked. So, he thought the practicalities of the proposal weighed heavily against it, but the wish and the desire that it stemmed from were thoroughly to be commended.

**Demoulin** agreed that the proposal was not possible to pass for two reasons. One was that what would happen with institutions that did not answer? Because in the smaller institution—and he thought the aim was to have more of the smaller institutions to take part—you may have the letter going from one head of department to another, it might be a physiologist, who would put it aside on his desk and no answer would come. And the other thing was that he did not want the General Committee or IAPT to write to the thousands of listed herbaria. In his opinion, if the Section wanted to go in that direction, and he supported the idea, the best thing was to use *Taxon* because if there was nobody in an institution that read *Taxon* he did not think there was a will in that institution to come and vote at a Congress, because they would not have read the proposals. He suggested using a full page of *Taxon* to make a big advertisement that “If you have not received institutional votes or if you were not satisfied with your number of votes, please respond now, after this Congress, so we can adjust our mailing lists”. He thought it was the easiest, cheapest way to do it.

**Freire-Fierro** wondered how many institutions, for example from Latin America, had the journal *Englera* so they knew that they had the opportunity of voting, and also not many institutions had *Taxon* either, so if the paragraph was included in the *Code*, many more institutions who did taxonomy, who had the *Code*, would know that they had the opportunity of participating in the Nomenclature Sessions.

**Landrum** was really in favour of the proposal in some form; it might be changed slightly. Even in his state, he tried to contact the curators, and a couple of them really did not have a clear idea about these meetings, and he thought that, for instance, we might save a little bit of money on *Taxon*. *Taxon* had become a very “shiny” journal; reduce the cost of *Taxon* and send out postcards, for example, to all the herbaria, he thought it would be a good idea. He concluded by saying that if you don’t invite people to your party they were not going to come, and you could not say, “Well, they didn’t come” because they didn’t know about it; if you don’t ask them they won’t come.

**C. Taylor** had actually worked in Latin American institutions, as an employee at one point. She felt that one year’s notice really was not enough because frequently the permission to travel was arranged a year ahead of time and you needed the papers authorizing it at that point.

**Pokle** fully supported the proposal because it would increase the representation from different countries in the Nomenclature Session.

**Nicolson** read from his own notes. These suggested that the text be replaced with three sentences:

1. Notify each institution in Index Herbariorum that they can request votes;

2. Only institutions responding get votes;

3. Votes 1 through 7in the list drawn up by the Bureau and approved by the General Committee.

He emphasized that it was important to get more representation.

**McNeill** wished to outline some things that could, and he felt probably should, be done under the existing wording. He thought the point that Demoulin made should be publicized earlier on in the sexennial span where institutional votes could be found, and with web access now to the IAPT website he thought there was no reason why the list of institutional votes could not appear there. He agreed with notifying in *Taxon* the opportunity to indicate where the institutional votes could be seen, encouraging an opportunity to express a desire to have a vote if an institution did not, and a consideration of the number of votes. He added that it was hard for him to understand how an institution could usefully participate in a meeting if it had no access to *Taxon*, not necessarily hard-copy access but electronic access, as it was where the proposals were published. He found it very hard to see how if someone had no access to *Taxon*, they could usefully participate in a meeting of this type. Therefore he felt that *Taxon* was a legitimate means of communicating, and IAPT had done a great deal to encourage developing countries and he hoped they would continue to do that. Secondly, he thought that it was quite important for the mailing of the final invitations to go out considerably earlier than they traditionally had done. They normally went out in February; this year they were a little late in March, and he was surprised to find that, airmailed in March from Vienna, they still did not get to some places for some months. He pointed out that there was no reason why they should not go out just about a year before the meeting, any earlier than that was much more likely to be forgotten and lost. The announcements of the Congress appeared much earlier, so people did know that it was coming; what was more, they knew they had an institutional vote previously and they knew they had applied for one, so he saw no reason why the General Committee and the Bureau should not take its action at least six to nine months or a year earlier than it traditionally had done. He felt that these two steps should encourage support. However, he did question the ability of, or the usefulness in some cases of, approaching *all* herbaria.

**Rico Arce** asked whether the letters regarding the votes were usually sent to the Director or to the Curator? She thought that sometimes the lack of communication between them was enormous.

**McNeill** acknowledged that everyone knew institutions where problems of that sort occurred, where the Director was in fact someone who was not particularly involved in systematics. It was an institutional vote, however, not an individual vote for the Curator, if just one of a small staff, so the policy that was used was not to use any names but just put the full and correct address of the institution as in Index Herbariorum or with corrections from the institutions themselves, and then say “The Director”. It may be that the person was the President, it may be the Curator was the Director, it may be the Chairman of a Department, it may be the Dean, but they just used the word “Director” as being probably the most universally acceptable. He did not think they could distinguish different titles for different institutions, and if an institution really had its organization so chaotic that it did not know it had seven votes, he suggested that maybe it should not have seven votes.

**Hollowell** noted that the journals *Novon* and *Annals* had good penetration to other continents by the subscribership. They offered to run the IAPT ad *gratis* at whatever fixed interval was decided—one year, two years—so that notification could better receive a global awareness.

**McNeill** responded that that was most welcome and offered thanks!

**Schafer** was very concerned about the necessity for the institution to reply; he thought it would exclude lots of institutions, which would, if it came two years ahead, just think there was time to reply, and the reply would not be sent.

**Glen** pointed out that in view of the ever-increasing costs and, certainly in his part of the world, decreasing reliability of snail mail, e-mail might be preferable.

**Stuessy** agreed, adding that it would be very simple to send out repeated notices through e-mail to all the IAPT members and institutions. He reported that they had over 1,300 individuals and a couple hundred institutions, so that could go out repeatedly and it costs almost nothing. He also offered that they could certainly use blank pages in *Taxon* to advertise visually, more frequently, and in combination with other journals, and do a lot better job. He thought it was quite right that they had not really worked at advertising more effectively beforehand, and could certainly do that for next time.

**Gandhi** added, as Hollowell suggested, that they also could do the same and run the notice in advance from Harvard, in their journal called *Harvard Papers in Botany*.

**Per Magnus Jorgensen** noted that all the good ideas did not bring the Section any further in answer to the question how to vote? He asked if there was a friendly emendation from the President?

**Nicolson** emphatically said no.

**Per Magnus Jorgensen** thanked him.

**Woodland** believe that the editor of *Index Herbariorum* was in the audience, and wondered if the Section could ask her how many of the herbaria she had in her databank had someone with an e-mail address. He thought it may certainly help to facilitate even in developing countries.

**Demoulin** reiterated that in a small institution, mostly universities, the director would be some department president, that changed with reorganization, and he saw many cases where this proposal would have the reverse effect because the letter would not be transmitted by the department president to the interested person, and it could even be worse, if it was an e-mail, and a department president received 30 or 40 e-mails a day, and if they were not directly interested they may very well forget to forward it to the right person. He believed the proposal should be voted down, but something should be done with good advertisement in *Taxon* and other journals who were ready to reproduce it.

**P. Holmgren** introduced herself as one of the editors of *Index Herbariorum*, adding that she had been trying to hide. She had sent McNeill a list of the number of herbaria that they had not even heard from for the last 20 years which was pretty high, although she did not keep those figures in her head. The number of herbaria that had been transferred elsewhere was around 700 or 800 now, so it was kind of a difficult list to send to, because she had a terrible time getting updated information from even major herbaria. Her fundamental point was that it was very, very, very hard to get responses from people. She felt that everyone got so much e-mail now that people just simply ignored it. She did not think the advertisement would make any difference, because her experience with *Index Herbariorum* was that the people who were very active were very active and knew and came to things like this and responded, but otherwise people did not: they would get the e-mail and ignore it because they did not even know what it was about. She thought it was a fine idea to put it in all the journals, it would not hurt anything, but she encouraged everyone present to update their information for *Index Herbariorum*.

**McNeill** added one thing to Holmgren’s comment, noting that she did send him the material because he knew the debate would occur and was not 100% certain whether she would be here. He reported that she had also provided him with e-mail addresses for those where she had them for correspondence, which was a very, very large number. So he felt it would be quite simple for that to be another advertising medium. He did not think it was suitable for the requirements of the proposal, but thought it could be part of a better communication.

**Ford-Werntz** proposed an amendment to remove the sentence “To receive it’s vote(s), each institution must reply expressing it’s desire to vote at the Nomenclature Section.” She thought it added undue complication to the entire process. [The amendment was **seconded.**]

**Eckenwalder** wondered how was the General Committee to know that an institution would like to exercise an institutional vote if that institution did not respond to it in some form?

**Bhattacharyya** felt the amendment was justified.

**Watson** asked whether that brought into the *Code* dependence on something the *Code* had no control over: *Index Herbariorum*?

**McNeill** pointed out that that was not in the amendment, but in the substantive motion.

**Barrie** questioned why, to begin with, there must have been some intent to get it in because actually it was more restrictive than the traditional practice anyway, in which anyone who appeared from an institution as a *bona fide* representative of that institution at a Nomenclature Session, received a vote. They did not have to do anything previous as long as they showed up. He suggested that perhaps it was in because it helped people get money to come with some institutions. He thought maybe Kirkbride could tell the Section, if he was present.

**McNeill** did not think Joe Kirkbride, the original proposer, was present.

**Davidse** explained that the reason that it was in here was to ensure that institutional votes were available as proxy votes to be carried by others, as was often the case with small herbaria from the Third World, when they could not send personal representatives.

**Freire-Fierro** still thought that the expression “*Index Herbariorum*” needed to be inserted in the Division III, because the way it was now, institutions and herbaria, did not know that they could come to these meetings.

**Marhold** wondered if it was really necessary to change what was already there. He felt that if the Section agreed to mention *Index Herbariorum* this was something like PDFs that it had been decided should not be in the *Code*. He wished to keep the wording of the ICBN as it was and advertise the possibility to take part at the Nomenclature Section and to get institutional votes.

**Nicolson** returned focus to the amendment.

**Tronchet** wondered if it was possible to have a web page which gave all the herbaria who were contacted by IAPT for the Congress, and if they could be personally advised if they did not answer? Either giving the votes to someone else or try to come. At Paris they had tried to contact several herbaria in France, just to ask them if they wanted to come or not, and if they wanted to give their votes, and they could not figure out which herbaria had votes.

**Nicolson** moved to a vote and asked for all those in favour of the deletion that had been proposed?

The amendment was **rejected.**

**Demoulin** pointed out that having a system writing with a request, and then writing back to confirm it would involve additional mailing to 3,000 institution and cost at least €2000. He suggested that the money could certainly be much better used in giving some kind of grant to a Third World country person to come to the Congress.

**Domina** reminded the Section that the vote was a right, and could not be deleted if someone at the institution was too busy or lazy in replying.

**Landrum** did not have to reply and did not think anyone had to reply.

**McNeill** explained that it was a change in the *Code* to force institutions to do so.

**Landrum** asked for clarification that from now on everyone would have to reply?

**McNeill** responded that that was what the proposal said, elaborating that if the director at Kew was away for a little while and did not reply, he supposed that Kew did not get any votes. [Laughter.] He added Edinburgh, too, seemingly as an afterthought.

**Nic Lughadha** hoped it failed but only because there was no time limit. She could reply the day before the Section and say “yes please” or an institution could reply even minutes before, and still be entitled to claim that vote.

**Nicolson** asked if she wanted an amendment?

**Nic Lughadha** responded that she did not, she wanted the proposal to fail, adding that the amendment was off the table.

**Nicolson** moved to a vote on the proposal on the board.

**Unknown Speaker** apologised for his poor English. He went on to say one year per year to issue International Botanical Congress if institution accepted by General Committee could he ask for participation in Section of Nomenclature so this institution for the future’s Congress? [sic]

**McNeill** asked if his amendment was to change the proposal to require each institution that currently received an institutional vote to apply for one for the subsequent Congress?

**Nic Lughadha** interpreted that the intention was that those who did not have a vote had to apply for one, so that should open the opportunity for institutions who were not currently listed to apply for a vote a year beforehand.

**McNeill** felt that could actually be a proposal independent of the rest of the text as it would be replacing the whole text, so he suggested maybe the Section should take it, once Prop. A had been disposed of, maybe we should take it right away as an additional proposal, as a new proposal. If it was seconded of course.

**Prop. A** was **rejected.**

**Fontella Pereira’s Proposal**

**McNeill** suggested that with Nic Lughadha’s help some words could be got together for the new proposal that was suggested, which he understood would try to enshrine it the *Code* things that he had said the Bureau would probably do voluntarily i.e. the right to institutions to request a vote.

**Funk** checked that she could take it as a given that the suggestions about the advertisements through journals were going to be followed through, so that there would be more advertisement to the community in general and an increased effort to contact institutions and inform them that they could apply for a vote?

**McNeill** was actually going to make that statement until the new proposal came up, which might affect it. He agreed with the sentiment and did not see any reason why the *Index Herbariorum* electronic list should not also be part of that advice. He felt he had to say, however, something that had not been mentioned at all in the discussion, what the criteria were for an institution to receive a vote. Basically, taxonomic activity was what they had been looking at, and there were rules of thumb that had been used in the past: if it had 100,000 specimens and it was the national herbarium clearly it was important. Another rule of thumb was if an institution was sufficiently active to have a representative at the Congress then it was accorded a vote even if it was not actually on the list. But what he thought had been a common point of view by successive Bureaux of Nomenclature was that this was not a second vote for every curator if the curator was the sole person in the place and it was a tiny little collection and really was not very taxonomically active. There was a balance, but he felt that the Bureau would tend on the side of generosity, in his personal view, with regard to developing countries in particular.

**P. Holmgren** noted that they [New York] could also send to each correspondent of each herbarium an advertisement.

**McNeill** thought it was much better if New York did it.

**P. Holmgren** agreed, adding that that way it went out by e-mail although this offered a problem if people had not kept their e-mail addresses up-to-date. She concluded that that was their problem, indicating that they were not part of the community if they had not kept things up-to-date. She felt that contact at periodic intervals was easy enough for them to do at really no cost and IAPT could guide them on how often that should be.

**Davidse** asked for a point of clarification: under the current rules, if a herbarium was not going to send a representative to the International Congress, but would still like a vote, an institutional vote assigned to someone else from their country who was going, was that routinely granted, was that impossible to grant, or what was the situation?

**McNeill** replied that it was a right, elaborating that an institutional vote, once granted, could be transferred to any other individual so long as no one individual carried more than 15 votes including his or her own. He added that that was as soon as it was on the list prepared before the Congress, but somebody turning up at the Congress clearly could not transfer a vote, but those who were on the list, agreed by the General Committee before the Congress and generally somewhere in the autumn of the year before, were entitled to transfer.

**Davidse** responded that that was not really what he was asking.

**McNeill** apologized.

**Davidse** wanted to know if you were not on the list, but wanted to be on the list, but you were only able to vote by means of proxy and were initiating the whole process.

**McNeill** explained that the previous Congress’s list was clearly the basis for the next Congress’s list, but it was not the same list. In other words, when he said the list, he was referring to the list drawn up by the Bureau of Nomenclature and approved by the General Committee, and that approval normally took place about nine or ten months before the Congress. Any institution on that list had full right to transfer the institutional vote to another institution, to any other delegate, with the restriction that no one delegate could have more than 15 votes. So there really was only one date that of the revision of the previous Congress’s list.

**Barrie** added that when an institution wrote and asked if they could have a vote they did not have to say they were coming to the Congress, all they had to say was that it was an active institution with X number of specimens, X number of people working, and a certain number of students, and they would like a vote to be listed on the list of institutions that had the institutional vote. What they did with that vote afterwards was entirely up to them. There was no requirement that they were going to send someone to the Congress, the criteria for getting the votes had nothing to do with whether they attend or not.

**Marhold** highlighted that it was hard to estimate the taxonomic activity of the institution. Using the rule of thumb that the number of specimens corresponded to current activity was a problem, he thought for instance in some European projects where people thought if an institution had enough specimens, they were good in taxonomy meant that activity in the 17th, 18th, and 19th century determined today votes, which did not make too much sense sometimes.

**McNeill** emphasized that there was never any rule that you had to have any particular number. It was simply adopted in trying to expand the number of institutions with votes, which took place prior to the Tokyo Congress, where the number went up by about 30%; mainly from Asiatic countries and from the developing world. One way to do this, where perhaps the detailed knowledge was not available to the Bureau, was to say that if an institution had 100,000 specimens, or if it was a national herbarium, that meant it was important, and in a developing country. He felt that was probably an appropriate criterion as they did not have herbaria in the 18th century, but it was not applicable across the board nor did it mean that they were not very excellent and active botanical institutions that should be represented, that were very, very small herbaria in terms of specimen number.

**Luckow** asked if it was possible on the IAPT website to have something about institutional votes such as a little link and to actually have an application there, because there was a lot of information that people may not just have, or know that they needed to provide in order to get an institutional vote and they might be able to do it electronically quite easily.

**McNeill** noted that that was a form of the advertising that had been talked about. He thought it should provide as much information as possible and found the suggestion reasonable.

[*The following debate, pertaining to a New Proposal by Fontella Pereira, and two New Proposals from the General Committee regarding Div. III took place later in the day during the Eighth Session on Friday afternoon*.]

**McNeill** returned to the proposal for an addition of a Footnote in Division III on institutional votes that someone had available.

**Nic Lughadha** asked the Chair’s permission for Fontella Pereira to say something very briefly in Portuguese and she would translate.

**Nicolson** agreed.

**Fontella Pereira** spoke in Portuguese.

**Nic Lughadha** translated and explained that Fontella Pereira was making his proposal with the desire to rectify what he saw as some deficiencies of the past, in particular the imbalance between large collections with large numbers of specimens but no active or few active taxonomists and no fully qualified people and other, much smaller collections, which had active taxonomists, and he cited as an example the Municipal Museum in Curitiba (MBM), with more than 100,000 specimens but only one active taxonomist, who did not have a doctorate, and the Federal University of Parana (UPCB), which had a much smaller collection but many active taxonomists and a postgraduate course in taxonomy, and he thought that by adding this footnote it would be possible to alert those who might wish to be better represented at the Section to the policy of requesting votes. The proposal would make clear to researchers at such un- or under-represented institutions the means by which they could request that their institution had a vote at the Nomenclature Section.

**Marhold** had a small point about whether some time should be indicated, because immediately before the Congress somebody may come and that could cause a problem. He suggested six months or something like that.

**Nicolson** suggested something innocuous like “in advance”.

**McNeill** asked if the Section would agree to some modification that made it clear that it had to be “in advance”, without specifying any precise date, as he thought that would probably meet the need.

**Nic Lughadha** responded that Fontella Pereira was happy to leave that to the discretion of the Editorial Committee.

**Nicolson** moved to a vote and deemed the proposal to have passed. [Applause.]

**Fontella Pereira’s Proposal** was **accepted.**

**General Committee’s Proposal**

**McNeill** put forward a proposal from the General Committee which he thought may save some time the next day when dealing with the General Committee and Committee for Pteridophyta reports. He reported that the Committee for Pteridophyta had six proposals to conserve or reject between 1993 and 1999, and between 1999 and 2005 it had half that number to consider, only three proposals to conserve or reject. Moreover, the present Secretary to that Committee was not in a position to continue as Secretary, and no member of the Committee was prepared to take on this enormously onerous task. [Laughter.] Therefore he reported that the Committee recommended that it not be continued as a separate Permanent Committee under Division III of the *Code*. Moreover, the present Secretary of the Committee for Spermatophyta did not feel that an additional three proposals in six years, even if it were a little more than that, would burden that Committee in any noticeable way. Consequently the General Committee had accepted the request from the Committee for Pteridophyta and was proposing that the Permanent Committee for Pteridophyta be deleted from Division III of the *Code* and what had been the Committee for Spermatophyta be renamed. To keep it consistent he was proposing the word “Tracheophyta”.

Before saying goodbye to the Committee on Pteridophyta, **Atha** wished to commend them for their respect of the Rule of Priority.

**Wiersema** was wondering if there would be some commensurate representation among the pteridophyte people on the new Committee?

**McNeill** obviously could not speak for what the Nominating Committee would provide the next day, but he did know that none of the existing members of the Committee for Pteridophyta wished to serve on the expanded Committee. He thought that the workload might have frightened them. A number of them had expressed a willingness to be consulted and steps had been taken to ensure that there was indeed appropriate representation.

**Bhattacharyya** thought it was better to say “Committee for Vascular Plants” as “Tracheophyta” was an unusual term, though it was meaningful, yet vascular plants was very popular term.

**McNeill** asked if that was a formal proposal, adding that it was perfectly in order to make it as a proposal to amend “Tracheophyta” to “Vascular Plants”.

**Bhattacharyya** agreed it was. [The amendment was **seconded.**]

**Brummitt** did not want to drag on the discussion, but there was a point that had nagged at the back of his mind for a very long time. These things were just called “Committee for Spermatophyta”, and when he used to fill in an annual report in his institution, people wondered what on earth this “Committee for Spermatophyta”, was and he had had to explain, well, it was actually a Nomenclatural Committee. He would personally prefer that the Committees be called “Committee for *Nomenclature of* Spermatophyta” as being a bit more explicit as to what they were all doing.

**McNeill** noted that that was a separate proposal from the one that was before the Section, so it would be returned to after considering the amendment.

**Nicolson** outlined that there was a proposal to change the name of the current Committee for Spermatophyta.

**McNeill** elaborated that the proposal was an amendment to the amendment which would have “Vascular Plants” instead of “Tracheophyta”. He had no strong personal views, and felt that he should go with what was currently in the *Code* for everything else except fossil plants, so thought it was better the Section made that judgment.

**Demoulin** explained that looking at the six Committees there were three *Archegoniatae* with division terminations and three (Algae, Fungi and Fossil Plants) with more general colloquial designations, so he preferred “Vascular Plants”, which was better understood than *Tracheophyta*.

**Nicolson** asked if the Section was ready to vote on the proposal to move the Pteridophyta...

**McNeill** interrupted to correct him that the proposal was on the Committee for Vascular Plants.

**Nicolson** clarified that it was an amendment to the amendment to change the Committee for Spermatophyta to the Committee for...

**McNeill** finished his sentence with “Vascular Plants”.

[The **amendment** was **accepted.**]

**McNeill** moved onto the substantive proposal, namely the abolition of the Committee for Pteridophyta and the establishment of the Committee for Vascular Plants.

**Nicolson** asked for all in favour?

**Skog** [off-microphone] “Extant” [Laughter.].

**McNeill** asked if she was proposing to change all the other Committee names to “Extant”? [Skog indicated she was not.] He referred to the proposal just voted on, checking that it had passed. He make a quick comment apropos of Brummitt’s point. He thought it was essential for all communications about these Permanent Committees to use the small “n”, nomenclatural committee for such and such, but within the context of Division III these were described as “Permanent Nomenclature Committees were established” and then under that appeared the word “Committee for Pteridophyta”. Otherwise he thought they were quite entitled to call themselves that because it was implicit in the structure of the Article.

**Nicolson** queried whether the title was “The Permanent Nomenclature Committees”.

**Brummitt** agreed that was clear from the *Code*, but when you had to publish something in *Taxon* and it just comes out as “Report for the Committee of Spermatophyta” it was not clear that it was a nomenclatural committee. He thought it should be stated in the title of the Committees.

**Barrie** felt that before dealing with Brummitt’s issue, the Section should finish voting on the proposal?

**McNeill** apologized as he thought the Section had.

**Barrie** thought the vote got stopped in the middle.

**Nicolson** noted that there had been a “yes” vote, but...

**Barrie** continued with no “no” vote.

**Nicolson** answered “Yes”. [Laughter.]

**Barrie** queried whether he meant “Yes, we had a “no” vote” or “no we”...

**Nicolson replied**, “ Yes, we had no vote!” and asked for how many opposed to the proposal?

**General Committee’s Proposal** was **accepted.**

**McNeill** acknowledged that he had jumped too fast. He noted the point that was made was very good advice to the Nomenclature Editor in *Taxon* to make sure that he put the word “nomenclature” in future, and perhaps the Secretaries might do the same.

[*Here the record reverts to the actual sequence of events*.]

### Article H.3

**Prop. A** (37: 99: 14: 2).

**McNeill** introduced Art. H.3 Prop. A, which had some negative voting, was in connection with positioning of the multiplication sign.

**Govaerts** felt that people from the Low Countries were rather pragmatic, and they liked to make the rules how practice was, and he thought in most cases people left a space even if they used a multiplication sign because it was often very much clearer, even in most publications by the Royal Horticultural Society, who he was sure knew the *Code*. That was the reason he had put forward the proposal, to put in law what was common practice.

**McNeill** thought one of the reasons for the Editorial Committee vote being significant may have been because the Rapporteurs drew attention to the fact that Rec. H.3A, Prop. A was addressing the same issue, but in a somewhat different way, so that the Section should probably take a look at that in coming to a conclusion on how to vote on this proposal.

**David**, in terms of representing the horticultural community, to some extent anyway through the Royal Horticultural Society, on nomenclature and taxonomy strongly endorsed the return of the space between the “?” and the nothogeneric name or the nothospecies name. He reported that it had been a practice which they had followed, and the change in the *Code* had caused them considerable problems.

**Mabberley** wished to reinforce that. From his own work, he got letters all the time in connection with *The Plant Book* with respect to the matter, and hoped very much that either this proposal, to H.3, or the second string, the Recommendation [Rec. H.3A Prop. A], was passed.

**Nicolson** noted that his wife, who was the real taxonomist in the family, would also like to have it. [Laughter.]

**Demoulin** thought Prop. A to H.3 was not a bad proposal, but Prop. A to Rec. H.3A was a better proposal, so felt that was the one that should be adopted.

**McNeill** thought the *Code* should avoid getting into rulings on typography, except where it was necessary to ensure clarity of the scientific name, and he personally thought, that if it could be left to people’s good discretion it would certainly be preferable than to force a typographical rule, which was not necessary for clarity of the scientific content.

**K. Wilson** was wondering in light of what the Rapporteur-general had just said, whether the proposer would accept an amendment, so that instead of saying “a single letter space”, change it to “the equivalent of a single letter space”, which could then be interpreted depending on the kerning or whatever in the printing.

**Nicolson** asked if there were there any comments on the proposal to amend?

**Stuessy** responded that from an editorial standpoint it made him just a little bit nervous. In a journal, then, there could be both approaches. He was not sure this was what was needed. He thought it was a nice idea, but in practice was going to look inconsistent. He preferred it be consistent either one way or the other.

**Nicolson** clarified that the proposal was that there would be a space, it would just be equivalent to a space, it might be a big space in one place, it might be a smaller space.

**Barrie** followed up on what Stuessy said, and wondered if this would put authors at the mercy of editors.

**Nicolson** said there would be a space if it passed.

**McNeill** explained that at the moment you just had to have the multiplication sign associated with it. It did not say whether it was one space, two spaces or right up against it, it just had to be associated with it, that was the wording.

**Nic Lughadha** requested clarity as to the wording of the proposed amendment.

**McNeill** checked that the amendment was seconded. [It was.] He asked if it could it be clarified, as there was some difficulty in its wording.

**Nicolson** understood that the proposal was to replace the phrase “a single letter space” with “a space equivalent to a letter space”.

**K. Wilson** agreed that was correct.

**Nicolson** explained that would mean that some cases it would be a bigger gap, just like sometimes there was a bigger gap between words.

**K. Wilson** did not see any problem with that, personally, because in the scale of the infelicities in publications these days, in editing, she thought it was a very minor matter whether it was a large or small space, but the key thing was to have a space, so she would agree with that.

**Wiersema** thought it would be useful to know exactly what it said in the “Cultivated Code” [i.e. the *International Code of Nomenclature for Cultivated Plants* (*ICNCP*)] about the issue. His suspicion was it was exactly the same as what was in the ICBN, but changing it had implications about what happened with the “Cultivated Code”. He did not have a copy.

**McNeill** did have an electronic copy, but it would take him five minutes to get it out. [A copy was produced.]

**David** informed the Section that the “Cultivated Code” had actually deleted the space in accordance with the ICBN and that was the reason why they would like to have the space re-included because it had caused them so many problems, but they had loyally followed the ICBN in this respect.

**Govaerts** suggested that, instead of making the wording more complicated, it might be simpler to just say “a space”?

**McNeill** pointed out that at the moment there was no requirement for a space or not a space, it said that the multiplication sign should be *before* the name or the epithet; not before without a space.

**Govaerts** was commenting on the amendment that was just made.

**Nicolson** clarified that the proposal now as amended would be “a space is left after the multiplication sign”.

**Kolterman** returned to what some people had said in the past. He really thought the idea of legislating typography in a rule was not a good step to take, and urged voting down this proposal and rather approving Prop. A under Rec. H.3A, which he thought was much more flexible.

**Moore** did not really think any Recommendations on spacing were needed. That was a matter of typography. He added that it said in the Article that the multiplication sign had to be immediately before the name and everyone knew that this was done differently by different journals even though there was a Recommendation that it should be so. The reason for the “immediately” was that a multiplication symbol had two roles in the *Code*: one actually indicated crosses, in some cases between genera, as in some of the Examples; in the other case it was used as an indicator that a name was a hybrid; so it had two roles. He preferred getting rid of the Recommendation that was in there, just leaving the Article as it was, and letting editors edit the way they wanted, either with the space or without it.

**McNeill** asked if the Wilson amendment was still on the table? [Voices: Yes.] He continued that, in that case, he thought the Section should leave the friendly change to the original wording until it was got rid of, or consider the amendment. [Laughter.]

**K. Wilson** thought she had agreed with the Chairman to accept the friendly amendment to just change it to “a space”.

**McNeill** summarized that the Section had just one proposal in front of them, simply the original proposal modified by removing the single letter.

**Brummitt** felt that clear guidance on what to do was needed and it should not be left to individual people. He very strongly urged the present proposal.

**Gandhi** reported that his colleagues supported having a space before the epithet as when the name was in italics, then the “x” sign, or the multiplication sign, clearly indicated the hybrid nature of the name, but when the name was in Roman letters, then the letter “x” in front of the epithet may not always be easy to indicate the hybrid nature.

**McNeill** really thought the Section was getting into areas that were not necessarily part of the rules of the Nomenclature. He knew that Art. H3 was not a condition of valid publication, but if a person did not do it, he asked the rhetorical question, “Was there any penalty”?, giving the rhetorical answer, “No, there was not”. He wondered why the Section would insist on this as a rule? Why was a rule on typography needed?

**Rijckevorsel** felt that it was much better as a Recommendation, as at the moment it was recommended not to have a space and some of the publishers had dutifully followed that, and if they were suddenly obligated to have the space then the publishers who had faithfully followed the present Recommendation would have books that did not conform to the rules. For the sake of consistency he argued that it was better not to make too big a change and secondly this was a topic on which feelings were running very strongly, so there would always be people who would not exactly follow it, therefore he felt it better left as a Recommendation. He added that Stearn wrote to the Congress advocating the use of both small and large multiplication signs to distinguish between formulas and epithets, so it was a topic on which there were a huge range of opinions.

**Peng** liked the proposal because for digitization projects, which most herbaria were working on, a space left after the multiplication sign served to distinguish hybrids from epithets beginning with “x”.

**Zijlstra** agreed it would be much better as a Recommendation. She felt that as it was presently worded it was simply a statement that did not say anything. If one would have it as a rule, a space *must* be left, and there was no punishment or sanction if one did not? She felt that as a Recommendation it could be worded by a small change in the present Recommendation: “a single letter space *should* be left between it and the epithet if this helps to avoid ambiguity”.

**Nicolson** was inclined to agree. He moved to a vote.

**Prop. A** was **rejected.**

## Eighth Session

Friday, 15 July 2005, 14:00–18:00

### Article H.3 (continued)

[*Discussion of Rec. H.3A Prop. A was begun before Art. H.3 Prop B and C but has been moved to follow the sequence of the* Code.]

**Prop. B** (15: 41: 95: 0).

**McNeill** introduced Art. H.3 Prop. B as making clear that nothospecific names were subject to the provisions of conservation. He felt the only question was whether it was already implicit in the *Code*, and therefore required a Note, or whether it required an Article.

**Brummitt** noted that everyone was getting to the end of a long day, a long week, and he did not want to spend time on the issue, he asked if he might speak to B and C together.

**McNeill** replied by all means, as they were mirror images.

**Brummitt** explained that Prop. B came from the Committee for Spermatophyta, as they had a case proposed recently of conservation of an interspecific hybrid and questions were raised whether this was allowable under the *Code*. He agreed completely with what McNeill said that it was implicit in the *Code* but it was not explicit, so in order to try to eliminate any doubts, he made the proposal. He felt the Section should not discuss it, if the Editorial Committee would be happy to put it in, that was fine; if they did not, his assessment was that there was not much lost.

**McNeill** definitely thought they would put it in, or a version of it.

**Brummitt** continued that Prop. C came up at the same time because members of the Committee said, well, if we conserve interspecific hybrids, can we also conserve intergeneric hybrid names? In his experience, that had never been attempted and there would be major difficulties about doing so because a two-genus intergeneric hybrid had to be part of one name and part of another name stuck together, and it had no type. The wording of the present *Code* was completely inappropriate for conserving intergeneric [hybrid names] and he hoped that the proposal would be straightforward. But there was a complication that had been raised with him since it was published. In the orchids there could be up to seven genera in intergeneric hybrids, and these days in the orchid nomenclature, with a seven-genus hybrid, the chances of one of them getting a new name were pretty high. So the orchid people were in a very difficult position: every time somebody changed a generic concept in the orchids it had a great knock-on effect in the -*ara* names, which could be applied to hybrids involving four or more genera. Now there was no mechanism to deal with this, and he did not want to introduce one unless anybody else present wanted to, but the possibility might exist to have some mechanism for conserving -*ara* names as having certain genera which would fix the usage of the name, and all the changes of the nomenclature, and so on, would be irrelevant. He just left that as a comment, if anyone else wanted to take up that idea, it might be worth discussing.

**McNeill** had not heard of that situation. Unfortunately, the reason why a nothogeneric formula could not be conserved was because it was a formula and it did not have a type. It seemed to him that the solution the orchid people probably need would be that names derived from multigeneric hybrids not to be hybrid formula but to be named as genera, which there was nothing to stop them naming them as genera, but they would of course have to be in some form or other that was in the *Code*.. He thought there would have to be a proposal to amend the *Code* to permit it, as it was a proposal to change the nature of those multigeneric hybrid names, which then automatically would provide the conservation mechanism. He suggested there was something for somebody to think about for the next Congress.

**Demoulin** was not an expert in hybrid nomenclature, but wished to understand why ?*Laeliocattleya*, for example, could not be conserved. What he found in Art. 14 that related to conservation of types was that the application of most conserved and rejected names was determined by nomenclatural types, so for the application of ?*Laeliocattleya* it would be determined by the type of *Laelia* and the type of *Cattleya*. He wondered where the problem was?

**Nicolson** pointed out that that was more than *a* type.

**McNeill** explained further that there was no type for that formula itself. The formula indeed was derived from two generic names, both of which had types, but it itself was necessarily the formula for all hybrids between species that were considered by the taxonomist to fall within those genera, so that one person would use one formula, and one another, depending on his circumscription but there was no type of that formula.

**Demoulin** persisted that each of the generic names in the formula had a type and to him that was all you needed to satisfy Art. 14.

**McNeill** responded that in terms of dealing with Brummitt’s Prop. B, then Demoulin would be making that point in the Editorial Committee to make sure it was an Article and not a Note.

**Moore** thought it might be useful to try and answer that question, explain why the nothospecies were allowed to be conserved. He thought it was because of Art. 40.1, which provided that, to be validly published, names of hybrids of specific or lower rank with Latin epithets must comply with the same rules as names of non-hybrid taxa. He thought this because looking at the Article for conservation there was no mention of hybrids being conserved at all, so hybrid at the species level got in through 40.1, but there was no provision there for anything higher than that.

**McNeill** felt that the *Code* was quite clear that nothospecies and lower hybrid ranks were the equivalent of species in terms of their requirements and so forth and that was not true at the nothogeneric level.

**Nicolson** explained that a “yes” vote was to refer to Editorial Committee; a “no” vote was to reject.

**Prop. B** was referred to the **Editorial Committee.**

**Prop. C** (17: 25: 107: 0) was referred to the **Editorial Committee.**

### Recommendation H.3A

[*Discussion of Recommendation H.3A Prop. A was begun before Article H.3 Prop B and C but has been moved to follow the sequence of the* Code.]

**Prop. A** (67: 76: 8: 0).

**Nicolson** introduced the Recommendation that had the same mission as Art. H.3 Prop. A

**McNeill** added that it was rewording the existing Recommendation on the same matter.

**Rijckevorsel** found it rather appropriate to be speaking to the last proposal on the Synopsis. When he first saw the Synopsis he was a little unsure if he should attend, and even now he was not quite sure if he made the right decision in coming, but certainly it had been quite an experience, and he was quite appreciative of the honour of addressing the historic meeting several times. Speaking to the proposal, he referred to the earlier comment that people from the Low Countries tended to be pragmatic, and there were a lot of people who had been quite vehement positions on the issue, so he thought that the *Code* should be pragmatic and try to accommodate them and just try to steer them in the right direction, and for a long while there was someone on the Editorial Committee who thought that there should definitely not be a space, which he did not quite understand. His feeling was that most people liked a space, so we should let them, but there was a big publisher in the United States which followed the *Code* and which left out a space, and they used exactly the right font, and that looked good, so he was quite happy not to have a space, if it was done tastefully. What he did not like were the “x”s, and the capital “X”s, and the italicized capital “*X*”s, so he thought it should be as clear as possible without being dogmatic.

**David** proposed an amendment to Rijckevorsel’s proposal, to read as follows: “The multiplication sign indicating the hybrid [nature] of a taxon should be placed with a space between it and the initial letter of the name or epithet...” all remaining text should be deleted, and then following on. [The **amendment** was **seconded.**]

**Atha** wondered if there was some other place in the *Code* that specified or discussed the symbol for the hybrid?

**Nicolson** did not think so.

**McNeill** replied to his knowledge not outside the Hybrid Appendix.

**Eckenwalder** requested that the current Recommendation H.3A appear on the overhead. [That was done.]

**Peter Jorgensen** suggested that the verb “should” should probably be changed to “may” as it was a Recommendation. [The **amendment** to the amendment was **seconded.**]

**McNeill** felt that, of course it may be, but as a Recommendation it had to say what should be done. He did not see why one would have “may” in a Recommendation, it was just statement of fact so he guessed he was speaking against the amendment to the amendment.

**P. Wilson** asked for clarification whether Rijckevorsel considered it friendly or unfriendly.

**Rijckevorsel** considered it unfriendly, and also thought it would not be a good thing because some publishers had followed the present *Code* and they had dutifully left out space and they would in this case suddenly be left with large stocks of books which would then be quite out of fashion, and he thought that for the sake of consistency the Section should not make this big a change, and...

**Nicolson** thanked him, returning discussion to the proposal that the word should be “may”, as opposed to “should”.

[The **amendment** to the amendment was **rejected.**]

**Govaerts** whole-heartedly supported the amendment and the Recommendation, because it was closer to what he proposed in the first place, and the reason he did that was to give clear guidance, and he thought the amendment gave much better guidance to people than the vague wording in the original proposal.

**McNeill** commented that the only thing that mattered from a nomenclatural point of view was the point made by Moore that the positioning of a multiplication sign or an alternative x was that it was clearly associated with the name or epithet involved and that it was not so spaced that it might be confused with a multiplication sign serving for a hybrid formula described in Art. H.1. He suspected, though he did not remember the details now, because that was his point, that it was quite a long time ago that the present Rec. H.3A entered the *Code*. So this was not something new and there was no question but that the present wording gave a clear position. He pointed out that if the Section accepted the amendment that would be a turn around. Personally, so long as there was some way that it was not confusable with a hybrid formula, and there was no wording here that made that clear, then he thought there was no problem which way you had it, but questioned whether something that had been in the *Code* for a long time should be changed?

**P. Hoffmann** commented on the comment that the gentleman had made earlier, agreeing that for databasers it would very useful to have the space so it could be clearly differentiated from epithets starting with “x”. She noted that it was a nomenclatural matter as it affected clarity of names.

**Govaerts** felt that even though it may be a big step for the *Code* to change it, it was a small step for the general public, as the Recommendation was rarely followed. It was sometimes followed, as Rijckevorsel had pointed out in that American publication, and they could still do that, of course, as it was only a Recommendation, but he felt it would not change most of the current use.

**Kolterman** suggest that a possible disadvantage of the change from the current was that if a usual space was used in a word processing document then it was not unlikely that the multiplication sign or the “x” was going to appear at the end of one line and the generic name or epithet was going to appear at the beginning of the next line. He hoped that editors would not allow that to happen.

**Nicolson** exclaimed, “Hear! Hear!” and asked if the Section was ready to vote on the proposal as it was up on the board?

**McNeill** corrected him to on the amendments.

**Nicolson** moved to a vote on the amendment? He thought it passed.

**McNeill** expressed doubt, in the form of an, “Um...”. He thought there was definitely a majority in favour of the amendment but whether it was a 60% majority he was not quite certain.

**Nicolson** asked for another vote again, going quickly to a show of cards, to judge whether it was 60-40. He thought it had passed, but deemed a card vote necessary with apologies.

**McNeill** instructed the Section that it would be number 5 and to please put “yes” or “no” on as well.

[*Here the record reverts to the actual sequence of events*.]

**McNeill** announced the results of the vote on the amendment to Rec. H.3A Prop. A were available.

**Nicolson** reported that the **amendment** was **rejected** on a card vote (264: 210; 55.7% in favour).]

**McNeill** returned to Rec. H.3A.1 Prop. A, the proposal of Rijckevorsel to change the existing Recommendation that the multiplication sign be against the name, and that if it was an “x” it be one space away, a more flexible Recommendation. He explained that essentially the part that had been crossed out on the screen was what was now being voted on, the material in the Synopsis.

**Nicolson** agreed that it was back to the original proposal.

**Prop. A** was **accepted.**

**McNeill** thought that the decision probably let you leave a space if you wanted it. He was really was concerned about the confusion with hybrid formula, with A ? B.

### Other Proposals

[*Discussion of a series of New Proposals presented by Redhead, followed by New Proposals from Wieringa and Haston, to define more precisely the impossibility of preserving a specimen regarding Art. 37.4 occurred here and have been to the Fifth Session on Thursday morning following the sequence of the* Code. *Discussion of two New Proposals by Wieringa regarding Art. 6.2 and Rec. 26B occurred here and have similarly been moved to the First Session on Tuesday morning and the Third Session on Wednesday morning respectively*. *Discussion of a New Proposal by Skog regarding Art. 1.2 and 11.7 occurred here and has been moved similarly to the First Session on Tuesday morning*. *Discussion of a New Proposal by Fontella Pereira, and two New Propoosals from the General Committee regarding Div. III occurred here and have similarly been moved to the Seventh Session on Friday morning*.]

**McNeill** stated that the Section had now completed the sequence through the *Code*, but there were a number of proposals for which, when they were discussed, it was indicated that, stemming from the proposal, there would be some addition, or change, or modification that would perhaps be beneficial. He outlined that these would be dealt with now, and he had a list of them, but may not have them necessarily in the right order. One of the first arose from Art. 22 Prop. C and Art. 26 Prop. A dealing with autonyms, and the desirability of having some Note in the *Code* indicating that specific autonyms did not create a taxon *per se*, and he thought that Wieringa had a wording. While waiting for display of the text on the board he suggested moving onto another that already was up by Bhattacharyya.

**Bhattacharyya’s Proposal**

**Bhattacharyya** requested the Editorial Committee consider the following two Recommendations for the inclusion in ICBN. Prop. 1: “Rec. 14B. Authors should generally follow Principle III with the exception to the names proposed and accepted for conservation.” Prop. 2: “Rec. 60A.3. Scientific names are not to be transliterized [sic!] in any other vernacular script.” (e.g. Rabatnoy “Fytotsenologia”, published by Moscow University, 1983, though there was an index indicating names in Latin; other examples from publication in Hindi, BSI Calcutta, India.) *Ficus* L. was “Phikus” or “Fecus” in other vernacular script in High School, Undergraduate, Postgraduate books of Bengali vernacular script was not clear. Indexing was greatly helped when scientific names were written in Latin.

[*The following continuation of debate, pertaining to a New Proposal on Rec. 60A by Bhattacharyya regarding using only Latin script took place during the Ninth Session on Saturday morning*.]

**Bhattacharyya**, introducing the proposal, explained that people other than taxonomists also used scientific names, and in publications names had to be used to indicate the identity of experimental material. Indexing was greatly helped when scientific names were written in Latin, but occasionally publications in languages other than English use scientific names printed in a particular script such as Russian, Hindi, or many others. A stipulation to this effect might force authors, editors, and publishers to write scientific names in Latin. The practice in undergraduate and postgraduate studies was to use the national or mother language, and transliterations often caused misunderstandings between the teachers and the taught. Publications of textbooks in national or regional scripts should also require Latin scientific names in Roman script. Students also needed to learn to write scientific names only in Latin script.

**Basu** seconded the proposal that scientific names should be written in Latin as in the *Code* and not be transliterated into vernacular scripts.

**McNeill** did not consider that the *Code* could legislate for what popular works chose to do. For example, it was perfectly common for a French wildflower book to use only names in French for plants, and as these tended to be in the binomial form this could be thought of as being like a scientific name, but this was not the business of the *Code*. He did not know the situation with all the languages referred to, but it seemed this would be outside the Section’s mandate.

**Nicolson** observed that there were editorial adjustments that could be made to the proposal, but it was the essence of the proposal that was before the Section.

**Bhattacharyya’s Proposal** was **rejected.**

[*Here the record reverts to the actual sequence of events*.]

**Nicolson** ended the day by announcing that somebody had left a manuscript on bromeliads.

## Ninth Session

Saturday, 16 July 2005, 9:00–13.00

**McNeill** informed the Section that in this Session the deferred business on electronic publication would be taken first and it would then proceed in sequence through the *Code* with proposals from the floor. Those making proposals were asked to ensure that they had electronic versions of their texts to display on the screen. After that the Committee Reports would be considered. He explained that there was to have been a proposal from Bhattacharyya regarding Recommendation 14B.2 deferred from the previous afternoon, as it had not then been available on the screen. As it was still not available, he suggested the Section move forward as it had been made clear that anything to be discussed had to be put up that morning. He understood there was a new proposal on Art. 60, Ex. 6 from Nic Lughadha.

**Nic Lughadha** stated that this had been **withdrawn.**

[*Discussion of a series of New Proposals on Art. 29 by K. Wilson regarding electronic publishing occurred here and have been moved to the Third Session on Wednesday morning following the sequence of the* Code. *Discussion of a New Proposal on Art. 7 by Gandhi regarding clarifying the kinds of types covered in 7.11 occurred here and has been moved similarly to the Third Session on Wednesday morning*. *Discussion of two New Proposals on Art. 9 by Gandhi and Tronchet regarding inserting Notes occurred here and has been moved similarly to the Second Session on Tuesday afternoon*. *Discussion of a New Proposal on Art. 20 by Zijlstra regarding use of Latin technical terms in names occurred here and has been moved similarly to the Third Session on Wednesday morning*. *Discussion of a New Proposal on Art. 30 by Wieringa regarding the use of ISBN and theses occurred here and has been moved similarly to the Third Session on Wednesday morning*. *Discussion of a New Proposal on Art. 32 by Chaloner regarding adding a term to the accepted Art. 32 Prop. E occurred here and has been moved similarly to the Third Session on Wednesday morning*. *Discussion of a New Proposal on Art. 33 by Demoulin relating to later starting points occurred here and has been moved similarly to the Fifth Session on Thursday morning*. *Discussion of a New Proposal on Art. 45 by Demoulin relating to later starting points occurred here and has been moved similarly to the Fifth Session on Thursday morning*. *Discussion of a New Proposal on Rec. 60A by Bhattacharyya regarding using only Latin script occurred here and has been moved similarly to the Eighth Session on Friday afternoon*.]

### Other Matters

**McNeill** indicated that the report of the group that worked on electronic publication asked that a Special Committee on Electronic Publication be established by this Section to report to the next Congress.

The Proposal was **accepted.**

### Reports of the Special Committees

*Committee on Electronic Publication*

**McNeill** drew attention to the report of the Committee published in *Taxon* 53: 592–594 (2004).

*Committee on Early Pre-1935 Lectotypification of Generic Names*

**McNeill** reported that this Committee had been convened by Nicolson, with Secretary L. Skog.

**Nicolson** apologized for not preparing a report.

*Committee on Suprageneric Names*

**McNeill** noted that the Committee had published two reports, in *Taxon* 53: 1081–1089. 2004, and *Taxon* 54: 491–499. 2005.

**Watson** commented that the Committee had been very active over the last six years, using a website and electronic publication to conduct its business, resulting in several proposals to this Congress.

*Committee on Effective Publication*

**McNeill** indicated that the convener of the Committee, Peter Jorgensen, had informed him that it had not made any progress, although some progress was made at this Congress.

*Committee on Hybrid Names in the Botanical and Cultivated Plant Codes*

**McNeill** reported that this Committee had been convened by Trehane, who had contacted him recently to say there was no report, and that the issue did not seem to be as important as it had appeared to the Section in St Louis.

*Committee for Liaison with Other Codes*

**McNeill** noted that this Committee had been convened by Knapp, with Stevens as Secretary. He had been told by Stevens some months ago that there had been no progress, and he assumed that was still the situation.

*Committee on Division III*

**McNeill** stated that this Committee had been set up with West as Convener and Davidse as Secretary, and he imagined that the situation was the same.

**McNeill** went on to say that if the Section wished to re-establish any of these Committees, as had happened already with that on electronic publication, there would be an opportunity to do that after lunch.

### Report of the Nominating Committee

**Chaloner**, as Chair of the Committee, indicated he would display the results of the Committee’s Proposals for each of the Permanent Committees. First he presented the list for the General Committee, which includes the secretaries of the Permanent Committees, and others, to which they had added a Chinese and Brazilian interest.

*General Committee on Botanical Nomenclature*

**Nicolson** observed that the General Committee was the subject for election.

**McNeill** noted that the Secretary of the Editorial Committee should be added, but that was automatic.

The composition of the General Committee was **approved.**

*Committee for Vascular Plants (“Committee for Tracheophyta”)*

**Chaloner** then introduced the proposals for membership of the former Committee for Spermatophyta that was now the Committee for Tracheophyta [it was later agreed that this should be called the Committee for Vascular Plants], which was incorporating the work of the former Committee for Pteridophyta. As a gesture to that, accepted by its Secretary, Brummitt, a pteridologist from South Africa had been added to cope with any problems from abandoning the Pteridophyte Committee.

The composition of the Committee for Tracheophyta [now the Committee for Vascular Plants] was **approved.**

*Committee for Bryophyta*

**Chaloner** reported that the Committee for Bryophyta now had 14 members, including two additions, one from China and one from the USA.

**Tan** enquired what the qualifications were to be considered for membership of a Permanent Committee. He presumed it must be someone familiar with the *Code*, or perhaps many publications on nomenclature, but wondered if the Chair could explain the basis of nominations.

**Chaloner** explained that the Committee had felt disposed to accept the nominations made to it, but if any were felt to be inappropriate, there was an opportunity now to make changes.

**McNeill** added that the requirements were an understanding of the *Code* in order to assess proposals to conserve and reject, and knowledge of the literature, practice, and usage in the particular group. The tradition was for the members and secretaries of the Committees to generate suggestions for new members and to determine which were willing to continue, but the Section had the final decision.

**Tan** knew the Chinese candidate who was just completing his PhD, but did not know if he would be willing to serve. He had a better suggestion from China, an experienced hepaticologist with a lot of publications on nomenclatural issues.

**Nicolson** remarked that it was helpful to have 15 members to secure 60% majority votes, so the addition could be helpful.

**Buck** indicated that from what he had now heard he would substitute the hepaticologist for the recent PhD candidate.

**Zijlstra** explained that the Committee for Bryophyta had come up with 12 candidates. She felt they must be sure that the two additional people proposed would be willing to serve, and at the moment she had no idea whether that was the case.

**McNeill** considered further proposals from the floor inappropriate, two more having been made. There had been a Nominating Committee and it had completed its work. If members appointed to Committees did not perform, there were procedures for them to be replaced by proposing this to the General Committee. The purpose of the Nominating Committee was to avoid discussing individual persons in such a large meeting. There was one proposed addition in this case and that seemed fine. The Committees also had the power to co-opt if they so wished.

The composition of the Committee for Bryophyta was **approved** with 15 members.

*Committee for Fungi*

**Chaloner** then put the proposals for the Committee for Fungi on the screen, with 16 members including an addition from China. The Nominations Committee had been mindful that Chinese representation was pretty thin or absent on many of the Committees so had been disposed to add Chinese members where they were assured these were appropriate. It had not been possible in the time available to check on the credentials of everybody they had added to the Committees.

**Demoulin**, as Chair of the Committee for Fungi, was very uneasy at what was going on. It was the first time in 30 years that he had witnessed discussions on the composition of Committees in six Congresses. The way the composition of the Committee for Fungi was arrived at was quite elaborate. They tried to maintain the 15 member number, and every time someone stepped down from the Committee suggestions were asked for and there was an election. Just before this Congress they had had a very tight election. The Committee also tried to be as geographically representative as possible and the Section would realize it also had to cover a wide taxonomic field, including lichens. It was true at the moment that there was no longer a representative from China; they had had a representative from China who had resigned and who had not suggested someone to replace him. That may be unfortunate, but he was very uneasy to have to take up in the group someone that he at least did not know, while all the people who had been candidates for this had given their cvs. They had paid great attention to getting appropriate people. His personal feeling was that the addition should not be accepted. He would like to have details of the proposed candidate so that he could be considered at the next election, that may not be too far away and maybe this person could be added. Or, the Committee should cancel the last election and replace the last addition from New Zealand by the new person from China, but he was very unhappy with that.

**McNeill** endeavoured to clarify the situation. The Permanent Committees generally generated names of persons whom they were proposing to add to the Committee for the ensuing six years, but the prerogative of the final composition was that of the Section. There was also no need for a Committee to be stuck at 15, there could perfectly well be 16 or more members; there was no limit, just a matter of convenience. The only role the Nominating Committee and the Section exercised generally speaking was to ensure balance in terms of discipline and geography. With all due respect, he felt that the method used by the Committee for Fungi was likely to lead to a loss of balance with respect to geography. The Section was there to provide a check and balance of this. The issue was whether the Section thought the imbalance such that it wished to override the wishes of the Committee, which it had every right to do.

**Hawksworth** stated that if the Section wished the Committee to have a representative from China, it could come up with a whole raft of well-known mycologists in China who would be more appropriate than the proposed person. It would seem extremely strange to many Chinese mycologists to find this person suddenly added to the list.

**Chaloner** was sorry that the mycologists felt that way. The Nominating Committee had been groping around in a brief lunch hour to try and obtain national balance which patently wasn’t too strong on that Committee. He would like to suggest that if they took this chap on board and didn’t feel he was doing his job, the job they wanted, or that his cv did not come up to scratch, perhaps they could find a couple of Chinese people with better cvs and co-opt them. He did not see a problem.

**Redhead** then spoke as a member of both the Nominating Committee and the Committee for Fungi. He had been surprised at the Nominating Committee meeting; it was very rapid, very efficient, and covered an awful lot of ground. He did mention to the Chair that the Committee for Fungi had been operating a voting system and was surprised that nominations could be taken in that hour. He also did not know the person either so he was a mycological unknown suggested to round-up the geographic coverage. Additionally, he was uncertain from the IAPT Constitution that the Nominating Committee even covered the different Permanent Committees. He was on soft ground as to whether to support this person as he was not known to the mycologists present.

**McNeill** said it was perfectly clear that Committees were appointed by the Congress. There was a proposal from the Nominating Committee the Section had set up to advise it, and the Section needed to vote to accept the list proposed or to remove one of the names they had suggested.

**Prance** pointed out that there were an awful lot of algologists and mycologists in Brazil, and suggested that the Chairs of both Committees consider co-opting people from Brazil as there was little representation from Latin America in general, although there was a huge amount of research in those areas.

**Gams** indicated that the Chinese candidate was also completely unknown to him. The Committee for Fungi had had a ballot with four candidates very recently, and there was a slight imbalance for Asia-Australasia but now they had voted in a New Zealand mycologist to improve the balance.

**Demoulin** stated that the Committee had also felt the first thing to take into consideration was the taxonomic coverage. In this case it was not known what group the person proposed worked with. The geography was a second thing. The point made by Prance just showed the can or worms opened when political nominations were introduced. After China, Brazil was mentioned; then you have to mention India, and after Africa, and the former USSR. The Committee would end up with 32 persons!

**Zhu** reported that the person he had nominated, was a top professor, and the leading mycologist of definitely the largest mycological herbarium in China, and probably in Asia.

**Nicolson** wished first to have the Section vote as to whether the Chinese candidate should be excluded from the list.

The Section **agreed** that the Chinese candidate be included.

**Demoulin** proposed that, in order to maintain an optimum number in the Committee, it should also include so it would be at 17, the person who almost made it during the last ballot, who was based in Spain.

**Chaloner** indicated that he would be very happy to accept that idea.

**Nicolson** then asked the Section to vote on the list as now posted with 17 members.

The composition of the Committee for Fungi was then **approved.**

*Committee for Algae*

**Chaloner** hoped the Committee for Algae would be more tranquil. The 15 members listed were as proposed to the Nominating Committee.

The composition of the Committee for Algae was **approved.**

*Committee for Fossil Plants*

**Chaloner** indicated that there had been a few new additions building the number up to 13, with no Chinese and a rather strong US and UK representation. Nominations from the Secretary to the Committee, J. Skog, had been accepted.

**Gandhi** informed the Section that there was an Indian palaeobotanist who was interested in being on the Committee, and if acceptable he would like him to be nominated.

**Nicolson** asked Gandhi to communicate that to the Chair of the Committee for Fossil Plants so that he could be considered, but as it was not necessary to add him just at that moment.

The composition of the Committee for Fossil Plants was **approved.**

*Editorial Committee*

**Chaloner** introduced the list and wished them luck. This was largely the composition as for the last one or two rounds, and in some cases the last three or four rounds of the revision of the *Code.* The Nominating Committee had added a Brazilian, and that was the only change they had made.

**McNeill** pointed out that there were three other new members on the list that had been suggested to the Nominating Committee. The Committee would now be one member larger than before. The list included four persons who had been responsible for translations of the *Code* into other languages: French, Chinese, Slovakian, and Portuguese.

The composition of the Editorial Committee was **approved.**

*Nomination of Rapporteur-General for the XVIIIth International Botanical Congress*

**Chaloner** indicated that the Nominating Committee had no great difficulty in suggesting McNeill as Rapporteur-General the next time round, though he thought the organizers of the next Congress, which he understood would be in Australia, might have some say in the matter.

**McNeill** stated that this was the decision of the Section. The organisers of the next Congress would appoint the rest of the Bureau on Nomenclature, but the Rapporteur-General was to be appointed now by this body.

**Chaloner** thanked McNeill for the correction, and he hoped that if he had misinformed his Committee the members would be equally happy with that information. [Laughter.]

**McNeill** added that if this were approved the Australians would be lumbered with him.

The nomination for the position of Rapporteur-General at the next Congress was then **approved.** [Applause.]

## Tenth Session

Saturday, 16 July 2005, 14:00–16.05

### Reports of the Permanent Committees

**Nicolson** proposed that if there was a vote questioning a particular item arising from the Reports it should require a 60% majority. That was the percentage used by the Committees and in the sessions of the Section and he wished to propose that. He also wished to suggest if it be the will of the Section that there should be some kind of a limit, perhaps 10–15 comments on a particular item and then the Section would be ready to vote. He then proposed 15.

This procedure and number of comments was **approved.**

**Gereau** wished to confirm that if the Section was questioning the Report of a Committee, this was a 60% vote to approve the Report.

**Nicolson** said it was 60% to overturn a Report.

**McNeill** clarified that it was 60% to reverse a recommendation in a Report as that would already have been approved by 60% within the Committees.

*Committee for Algae*

**Silva**, Chair of the Committee, reported that as constituted in St Louis the Committee was well balanced both taxonomically and geographically. The number of proposals to conserve generic names had decreased, while those to conserve or reject specific names had increased. Four reports had been published [in *Taxon* 48: 811–814. 1999; 52: 339–340. 2003; 53: 1065–1067. 2004; and 54: 523–524. 2005]. The Committee also recommended that *Helminthopsis* Heer (fossil) and *Helminthiopsis* J. Agardh (red algae) be treated as confusable. The Committee had supported two proposals to modify the *Code* made on its behalf, but not one to abandon later starting points for the nomenclature of *Cyanobacteria*/*Cyanophyta*. It had also suggested that a Special Committee be set up with delegates from the International Association for Cyanophyta Research to work towards harmonization of the nomenclature of blue-green prokaryotes under the two pertinent *Codes*.

The Report of the Committee was **accepted.**

**Hawksworth** wondered whether the proposed Special Committee should be set up together with the International Commission on the Systematics of Prokaryotes, the counterpart of the Section, rather than name a particular Association.

**Demoulin** hoped to be on that Committee and would ensure that besides the people working on this group there should be one person involved in each of the two *Codes*.

**McNeill** stated that representation on the botanical side would be finally appointed by the General Committee, but it would be foolish not to take on board those people keen and anxious to work in it.

The establishment of a Special Committee on the Harmonization of the Nomenclature of Blue-green Prokaryotes was **approved.**

**McNeill** suggested that the Committee Chairs or Secretaries give brief reports here and that their full reports would be printed.

*Committee for Fungi*

**Gams**, Secretary of the Committee, reported that it had been very consistent in its activities. It had now published 13 reports, those since the last Congress had appeared in *Taxon* **48** (4): 807–810 (1999), **50** (1): 269–272 (2001), **51** (4): 791–792 (2002), **53** (4): 1067–1069 (2004), and **54** (2): 520–524 (2005). He was pleased he could hand over the Secretaryship to Norvell with a good conscience that most problems had been satisfactorily solved. The major problem they had been faced with was ruling on the nomenclature of *Coprinus* and related genera; there was considerable conflict between two alternatives, but the proposal to change the type was not successful so he reported that *Coprinus* stayed as *Coprinus comatus*, and the three genera introduced by Redhead and co-workers stayed as they were. The Committee also supported the establishment of a Special Committee for Art. 59, about which it had not yet reached conclusive results.

The report of the Committee for Fungi was **accepted.**

**McNeill** drew attention to lists that were open for people to indicate they wished to be considered as members of the Special Committees being set up by the Section. Volunteers were not automatically placed on a Committee, but their names would be considered by the General Committee when determining their final compositions.

*Committee for Bryophyta*

**Zijlstra**, Secretary of the Committee, reported that during the last six years the Committee had considered seven proposals to conserve generic names, and eight for species names. Discussions were still on-going on one species name, and an unpublished request to give an opinion on a correct spelling of a species name. Four reports had been published, in *Taxon* 48: 563–565, 815–816. 1999, 51: 793–794. 2001 and 54: 525–526. 2005.

The report of the Committee for Bryophyta was **accepted.**

*Committee for Pteridophyta*

**McNeill** reported that the Secretary of the Committee was not able to be present and there was no member present to report on her behalf. The Committee had one proposal carried over from the previous Congress because of some ambiguity, but that proposal had become unnecessary because of a change in the *Code* at St Louis and so was rejected. The Committee had received and acted on three new proposals to conserve names (*Taxon* 54: 831–832. 2005). It had been unable to find a new Secretary, and the recommendation was that the Section be dissolved which had already been dealt with.

The report of the Committee for Pteridophyta was **accepted.**

*Committee on Spermatophyta*

**McNeill** observed that this would be the last report of the Committee before it changed its name to the Committee for Trachaeophyta. [The change was actually made to “Committee for Vascular Plants”].

**Brummitt**, Secretary of the Committee, reported that reports had been published in *Taxon* 49 (2): 261–278 (2000), 49 (4): 799–811 (2000), 50 (2): 559–568 (2001), 51 (4): 795–799 (2002), 53 (3): 813–825 (2003), 53 (4): 826–829 (2003), and 54 (4): 527–536 (2005). Communication was in hard copy sometimes with e-mail attachments, and the Committee had had about 800 pages of discussion in the last six years. A letter was sent out about every two months and the members were given 4–6 weeks to reply. The Committee was up-to-date with all proposals received to the end of 2004. In addition to published proposals, the Committee had received a continual inflow of informal requests for recommendations on homonymy; it was not required that those be published in *Taxon*. When a decision was reached, he endeavoured to send an e-mail report on each proposal to the person who had made it. They were fortunate to have a good active Committee where members always voted.

The report of the Committee for Spermatophyta was **accepted.**

*Committee on Fossil Plants*

**Skog**, Secretary of the Committee, reported that it had published four reports, in *Taxon* 48 (4): 817–819 (1999), 50 (1): 273 (2001), 52 (2): 341 (2003), and 54 (1): 175–176 (2005). It had successfully dealt with all but one of the proposals placed before it, which it was understood may be withdrawn. They would also work to bring the Committee up to 15 active members.

The report of the Committee for Fossil Plants was **accepted.**

*Editorial Committee*

**Hawksworth**, Secretary to the Editorial Committee for the *St Louis Code*, reported that the *St Louis Code* had been published. He wished to thank those on the Committee and named on the title page, but also others not named there or who helped in particular ways. Particularly Mrs Rosemary Zeigler (Berlin) who initially typed the transcripts of the Section meetings prior to editing by Barrie, McNeill and himself. Greuter had done an excellent job in guiding the Committee through the *Code*, and had prepared a draft with all the different proposals in it which had greatly facilitated the Committee’s work. He felt all owed him a great debt for that, and that affected the speed with which the *Code* came out, achieved in June 2000.

**McNeill** interjected that it was probably a record, and was certainly out within a year.

**Hawksworth** continued, recognizing those who worked on the Appendices, particularly Demoulin, Nicolson, Silva and J. Skog on the Committee, but also Zijlstra and Isoviita for work on the bryophyte names. Barrie and Turland also did a sterling job in correcting dates of flowering plant family names, which he assumed would now need altering again. The subject index had been primarily the responsibility of Trehane. As Secretary of the Committee, he wished to thank all those different people for their tremendous efforts in producing the “black” *Code* in such an efficient and timely manner.

The report of the Editorial Committee was **accepted.**

*Report of the General Committee*

**McNeill** explained that this report was to be presented in two parts. The first would deal with all matters referred to the Committee with the exception of Committee for Spermatophyta Report No. 55, and the second would take up that report as it was understood there would be some discussion of details in that report.

**Barrie**, as Secretary of the Committee, reported that copies of the report had been circulated to the Section. The typical role of the General Committee was to review the reports submitted to it by the various Permanent Committees to check that all the requirements of the *Code* had been met, and look at any larger questions that might be an issue. It then made a decision to approve or not approve the Committee report, and sometimes referred items back to the Committees or considered them further in the General Committee.

This time one report was handled a little differently, that of Special Committee 3C set up at the Berlin Congress to look at a specific group of Linnaean generic names and their lectotypes; there were about 72 names that had competing typifications, and it was thought that some effort should be made to evaluate them and decide which types were most appropriate for maintaining usage. Jarvis was Chair of the Special Committee, did most of the work, and deserved a great deal of credit for the results it came up with. One of the problems was that the list was generated and published in *Taxon* 41: 552–583. 1992, prior to the Tokyo Congress and before the general realization of the implication of what Voted Examples meant. So at the time the report was being generated and the research being done, it had not been entirely clear that it was required that Britten & Brown?s 1913 typifications not be used and would not compete with later typifications as they were ruled as mechanical. A significant number of the genera did not now need conservation because the Britten & Brown typifications were ruled out, and the later typification was acceptable to maintain current usage. At the Tokyo Congress, the report was dumped directly on the Committee for Spermatophyta for review. They recommended that all the genera except *Briza* should be accepted. However, the General Committee wondered how appropriate it was to conserve names that really did not need it. Nicolson teased out which names actually needed conservation and which did not; these were listed in the report to the Section.

The Committee had reviewed 23 Permanent Committee reports since the St Louis Congress, all of which were approved, although several specific proposals were still under discussion or referred back to Permanent Committees for reconsideration.

**Rijckevorsel** noted some errors in the distributed report. On p. 6 (case 1400) the wrong family name was given, on p. 6 (case 1528) had an error in a conserved spelling, and on p. 10 (case 1564) he wondered if an omission was deliberate.

**Barrie** recognized that there were typographical and other errors in the distributed document, and indicated that he would be very happy to receive information on these and correct them in the printed version. Those matters did not affect the validity of the decisions.

**McNeill** thought the omission in case 1564 [to conserve the name *Platonia insignis* against *Moronobea esculenta*] was deliberate.

Report of the General Committee, excluding Committee for Spermatophyta Report No. 55, was then **accepted** and the recommendations therein **approved.**

**Barrie** explained that the Committee for Spermatophyta Report No. 55 was the one that proposed the conservation of *Acacia* with a conserved type. The General Committee had considered this very carefully and had received a great deal of communication on the matter. The Committee for Spermatophyta had voted to approve by 9: 6 (60%), as did the General Committee 14: 6 (60%). There was nothing technically wrong with the proposal.

**McNeill** stated that this was the point where any that did not agree with acceptance of that report should speak.

**Schrire** wished to give a brief reflection of the views of those opposed to Proposal 1584. The issues on both sides of the debate had been published in recent issues of *Taxon*, and the main objections to the proposal for retypification of *Acacia* from an African to an Australian species focussed on two aspects. Firstly, the science was not yet sufficiently adequate to justify the proposal of conserving *Acacia* with an Australian type, and secondly there was a strong sentiment among the international community that conservation was not justifiable in this case.

As regarded the science, the mimosoid tree was based on chloroplast sequence data and had yet to be complemented by nucDNA data. Further, species sampling was poor; based on published evidence, the mostly Australian *Phyllodineae* had had only 70 of about 970 species sampled, about 7%. In addition, the section containing the newly proposed type species *Acacia peninnervis*, was the largest and least sampled of the sections. Also, support for subgenus *Phyllodineae*, again based on published evidence, was only 86% – a figure generally considered as marginal as a basis on which to make such an important decision. In addition, critical genera in the tribe *Ingiae*, as well as of *Acacia* subgen. *Aquiliferum*, had yet to be included as outgroups in the most recently published analyses. It was therefore possible that nuclear data and more detailed and critical species sampling of subgenus *Phyllodineae* and outgroups might resolve the Australian *Acacia* species in different areas of the *Acaciengae* alliance. This could involve having to move numbers of Australian acacias into other genera, so defeating the object of this retypification. Thus, conserving *Acacia* with an Australian type in the interest of stability based on species numbers alone was highly premature, given the knowledge that the science behind the proposal was based on inadequate sampling.

Conservation was generally not deemed justifiable in this case because there was a general misconception that this was largely an Africa versus Australia issue. This could not be further from the truth, as the genus was pantropical with comparable numbers of species in the neotropics and Africa, and widely dispersed in tropical Asia. Rejection of Proposal 1584 has received widespread international support. For example, less than 10% of the world’s 80 practicing legume systematists support this proposal, and the 37-authored rebuttal paper published in *Taxon* 54: 513–519. 2005 was testimony to the breadth of support. An acceptance of recognizing the generic name *Racosperma* in Australia had already been shown in that all combinations for Australian *Acacia* species had already been published under *Racosperma*. The retypification had not yet been sufficiently well proved to be necessary, and such exceptions to the *Code* should only be considered when there was overwhelming supporting evidence. Otherwise, this would compromise the future predictability of the system of botanical nomenclature. It therefore seemed best to let simple priority and normal typification rules decide this issue, when there was so much opposition to it.

In conclusion, he said that many tropical-based colleagues had asked to convey their sentiments against the proposal to the meeting, and have the Section vote on the matter. The decision made should reflect those colleagues’ views as it was they who would be most affected by the proposed changes. In order to avoid any ambiguity, he wished to ask the Section that a card vote be taken on a motion to reject conservation and retain *Acacia* with an African type.

**Luckow** indicated that to her the issue transcended the nationalistic feelings that had been raised, although it was a part of the problem. The issue was controversial on a global level, and it was not just a question of Australians *versus* Africans. Conservation was for special cases, as written in the *Code*, and had mostly been used in the past in very clear-cut cases. She was not trying to question the motivation and capabilities of either the Committee for Spermatophyta itself or the General Committee. She thought they did an incredible job sorting through nomenclatural issues, but felt that they may not have had some of the information her and her colleagues had when making their decision. Priority was designed for when there were going to be hard feelings no matter what the decision was; conservation on the contrary was not designed to do that. The Committee for Spermatophyta had already said in a previous report, when working with *Myrica*, that when there was a good case to made on either side that simple priority should decide the issue. She argued that the proposal would have large repercussions for the nomenclatural system in that it would demonstrate a departure from priority in what was clearly a controversial case.

**Pedley** had been involved, had lived, with the issue for quite a long time, and was actually surprised that the conservation proposal went through. The Preamble of the *Code* stated that it aimed at a stable method of naming taxonomic groups, avoiding and rejecting the use of names that caused error or ambiguity, or threw science into confusion. Next in importance was the avoidance of the useless creation of names. Other considerations, such as more or less prevailing custom, were relatively accessory. Notwithstanding the molecular evidence or lack of it, he believed *Acacia* must be split up, but did not believe there was any justification for moving the type. That would cause confusion, and about 160 new combinations would have to made under *Vachellia*, a name that a lot of people might have to use. So far *Vachellia* had been used for about five species. Another object of the *Code* was to put the nomenclature of the past into order and to provide for the future. He felt it had made a pretty good job of clearing up names from the past and avoiding confusion, but usually cases were clear cut. The only real reason for conservation was to get rid of a name dredged up from somewhere. But *Acacia* has not been dredged up, and had been used in the vernacular for millennia. He saw no justification for moving the type from Africa to Australia.

On the other hand, Australia had a small but well-educated population, and consequently could absorb name changes fairly readily. Not only that, the Australian acacias, or racospermas, a dreadful name, were more or less confined to the Australian continent so it was dead easy just to change to *Racosperma*, and there would only be about a 1% chance of being wrong, whereas in Africa there would be a mixture. He felt that the Australians should bear the brunt of this business, accept it, make the changes, and let the rest of the world get on with it.

**Orchard** considered that the discussion had to be about the stability of nomenclature and not parochial self-interests. He agreed with the first speaker that this was a global problem and needed a proper global solution, and did not think rejecting the conservation proposal was the way to get a sensible global solution. In Tokyo, there had been spirited debates on a range of topics. One of those was the perception that taxonomy and nomenclature were getting a pretty bad PR. The user community it was serving were getting pretty fed up with constant name changes and were losing patience with taxonomy and nomenclature. The Section had felt so strongly about this issue, that it massively increased the conservation and rejection provisions of the *Code*, and went so far as to pass a Resolution not only within the Section but up to the Plenary Session of the entire Congress. This said that taxonomists were going to do everything they possibly could to minimize the number of name changes that were being imposed on the user community by taxonomic advances and changes. For many years, Permanent Committees had been set up to look into conservation and rejection proposals and other taxonomic matters. He noted that those Committees spent weeks, months, and sometimes years, working through individual cases; looking at the evidence, consulting colleagues, and inviting communications from other people, to come up with the best possible solution to each case as presented. He felt rather uncomfortable about the procedure now being used, that a case which had received months of attention by a Permanent Committee, which had been appointed to do a job, could be overturned on the basis of a few minutes debate. If the present motion succeeded, he saw real problems for the future. He was not aware how many cases had been challenged on the floor of a Nomenclature Section, but knew it was very small, if indeed it had ever happened before. This would be seen as a precedent, and he feared that in future the Section could be looking at challenge after challenge to particular cases some people did not like. Decisions made on *Centaurea*, *Hedysarum*, and *Leucaena* in the same batch could also be challenged as based on the same Articles in the *Code*.

He offered to put the matter into perspective for those not intimately involved, noting that *Acacia* had about 1350 species, and was divided into three main groups: *Acacia* (161 spp.), *Phyllodineae* (about 980 spp.), and *Aculeiferum* (203 spp.). The proposal was to split the genus into five groups, and the major impact would be on Africa and the Americas, with smaller impacts elsewhere. Of the 161 species of *Acacia*, 60 were in Central and South America, 73 in Africa, and 36 in Asia and Australia; the *Phyllodineae* included 980 species, of which about 960 were in Australia, ten in Asia, and two in Madagascar. It seemed to him if the split was to go ahead, that the typification should move with the biggest group. This was a test for the Resolution passed in Tokyo, was the Section really serious about trying to prevent the uptake of nearly 1000 new combinations?

If the *Code* was allowed to take its course, the biggest impact would be on Australia. It was important to look at that impact. From the e-mails displayed in the foyer, it was clear that public feeling was really very, very, strong. The 960 species in Australia were part of a flora of 18 000 species, so *Acacia* was 5.5 % of the entire vascular flora; in an area of 7.6 million sq. km, that was about one species for every 7600 sq. km. Taking Africa as the opposing example, after the split they would have about 73 species in a flora of about 50 000 species, that was 0.13 % of the flora, or one species for every 425 000 sq. km. He felt those two sets of numbers gave some feel for the impact on the two continents, and he believed very similar numbers to the African ones would come up if the same analyses were done for South America and Asia. *Acacia* outside Australia was a very minor component of the flora. Within Australia, it was not surprising that an important genus which dominated the entire flora had tremendous iconic and economic importance. *Acacia pycnantha* was the national flower, part of the coat of arms, its sporting colours (green and gold in the Olympic Games), and also a major part of the Order of Australia awards. In Australia, *Acacia* was in every vegetation type, had enormous impacts in terms of land reclamation and soil conservation, dry land forage, land cover, etc., and in many areas were the dominant vegetation type.

Overseas, the Australian *Acacia* species were also tremendously important economically. About 157 Australian species had or were being trialled in over 71 countries, mainly in Africa, the Middle East, Asia, and South America, for large-scale industrial timber, fibre, and tannins. And on a much smaller but important scale, also for fodder, soil conservation, human food, firewood, floriculture, and similar things. It was interesting to note that in South Africa, the most profitable forestry species was currently *A. mearnsii.* Plantations in Brazil, China, South Africa, and Vietnam generated around US$ 71 million per annum. Other species, were also planted in a whole range of other countries, and there was currently 1.5 million ha of plantations in China, Indonesia, Malaysia, and Vietnam generating US$ 900 million from pulp alone. *Acacia saligna* in North Africa, the Middle East, Asia, and Chile covered about 0.5 million ha. Also, *Acacia colei* was being developed as a food in sub-Saharan Africa. All these species would change their names if the type was not moved.

He therefore urged the Section to endorse the properly constituted decisions of the Committee for Spermatophyta and the General Committee, and allow the type to be re-designated.

**Fortunato** said that for now for her it was one genus, but she wished to inform the Section that Marta Caccavari, a specialist in the group, had recently published a fossil record of *Acacia* subgen. *Acacia* pollen from South America. This represented the most ancient report and was where the genus began. This was another reason to show how artificial the proposal was to change the type to another group of the genus.

**Brummitt** stressed that this was an extremely important case, and requested that the Section listened carefully to all the arguments. He wished first to make some general comments about Committee procedure, to refer to three particular issues that had come up, and then to briefly go through the arguments that persuaded two Committees to accept this proposal. In all this, his dearest wish was to find some way everybody agreed with everybody else, but he did not have a magic wand and could not do it. Something had to give somewhere. Speaking as Secretary of the Committee for Spermatophyta, he explained that the proposal had been supported by a clear majority in the General Committee after, almost uniquely, a long discussion of the case among its members. He did not believe that a recommendation of the General Committee had ever before been challenged on the floor of a Congress Nomenclature Session. This was a unique occasion. Clearly if the Committee asked for approval of a recommendation, it must also invite disapproval, if appropriate. He had always said that democracy must be seen to be done, and that was why *Acacia* was being debated that day. But he added that if the Section were to overthrow the recommendation of two Committees, it needed a very strong case indeed. Otherwise it would undermine the whole Committee structures and established procedures. He would look for additional evidence that the Committees had not previously considered, but he had not heard that so far, quite the contrary.

The Committee for Spermatophyta had considered the case at great length, and all comments submitted had been circulated, more than 50 pages over six months, before a vote was taken, and the correspondence had continued to grow since. When he first circulated the Committee, the first thing he had said was that this going to be the highest profile case it had ever conducted, and it seemed he was right. The second thing he said, was that that there were many personal views attached to the case, and that the Committee should consider above all the facts of the matter and ignore personality conflicts and personal opinions. He urged all those there that afternoon to try to do that also. The next thing he had said to the Committee was that they had to declare any personal interests they had, so he felt he should declare his also to the Section. When he first worked as a taxonomist, he was put to work on the *Mimosoidae* for *Flora Zambesiaca*, though not on *Acacia* as his colleague Pat Brenan had a prior interest in that. He had been on the fringe of *Acacia* taxonomy from 40 years ago, and was aware of disagreements over splitting the genus, over publication of the *Flora of Australia* account, and who should publish the new combinations. These personal differences were very evident to anyone who was involved. Later he had developed an active field interest in Africa, and developed close ties to at least one African institution. He had travelled and collected plants in every country from Ethiopia to the Cape, so had seen many African acacias, and had many friends in Africa, and was biased from the start.

When this proposal was in the making, he had to visit Australia for a meeting, and let it be known to the two main protagonists in Australia that he was coming. He spent time in the field and stayed with each of them, and so learnt quite a lot about *Acacia* in Australia, though they were kind to him and did not pester him too much about it. It could be said that he gladly laid himself open for any bribery and corruption equally from both sides. Since then he had struggled to accommodate two completely conflicting views in his own mind. His first reaction was that the case for moving the type to Australia was entirely justified from a consideration of the facts, and he had to put aside his natural inclination to favour Africa.

There were three points raised as arguments he wished to comment on. It had been said repeatedly that the cost or re-curating specimens in herbaria was going to be enormous and prohibitive in the African herbaria. He had worked in African herbaria, and what would happen was that they would have to get out all their *Acacia* material and divide it into two piles, one would be *Senegalia*, and there was nothing they could do about that, and the other would be either *Acacia* or *Vachellia*. They would strike out *Acacia* on one of these if the proposal goes through, and have to insert *Vachellia*. How long would that take? Half an hour, one hour, two hours, maximum a day. Yet people talked about this as an impossible financial burden on herbaria in Africa and other developing countries. He would therefore dismiss the cost of curating herbarium collections, though live plants would be a completely different matter.

He had listened to the arguments from the opposition for a long time, and read very carefully the paper in the May *Taxon* [54: 513–519. 2005], but simply found nothing in it to change the situation. The main argument in that paper was the number of people in the different continents. But amongst the e-mails in the foyer was one from the Forestry Department of Indonesia; the noun “acacia” had been incorporated into their language, but for the Australian and not their native species of *Acacia* – they wanted to keep the name for the Australian, which is what the proposal would do. This would also apply to China, so two billion people could be transferred from one side of the argument to the other.

There was a third point, which he had thought long and hard about raising, but had decided he should do so. He had seen hidden statements that the Committee for Spermatophyta was biased, and dominated by people from the developed world who were dictating nomenclature to suit their own interests. When you heard something like that, you had to laugh or cry. His reaction was complete disgust that anyone should raise this as an international issue. This was a letter that had been circulated around the world to recruit support, implying that the Committee of which he was Secretary was biased against the developing world. He took great exception to this, which had been a personal problem for him in his own institution and elsewhere. There should be no place in nomenclature for political accusations like that. He liked to be politically correct, but there was a limit to political correctness. Of the 15 members of the Committee who voted, two were from Asia, one from Africa, and one from South America. In addition, one of the members was born and spent his early career in Asia and now lives in America, and one of the American members had spent a long time working in the field in Asia. Of the European members, three had spent nearly all their careers working for the African *Floras*, with many very strong African contacts. One of the other members had spent a lot of time in South America. So far from this group being biased against the developing world, they had no members whose inclination would be to favour developed countries. There was only one Australian on the Committee to push their side. There was thus a heavy bias against Australia in the Committee, if one wanted to look at it that way. But the Committee had done what he thought was right, and looked at the facts, and not these other issues.

He wondered how he could get it into the heads of the opposition that there were very good solid reasons why the Australian group should retain the name *Acacia*. He had been disappointed to hear in the Section that the issue of the taxonomy was being raised again. The Committee was faced with a taxonomic position where the genus was being split into three major groups, with two smaller ones in Mexico. There was nothing it could do to adopt a different taxonomy, that must be accepted, and the earlier raising of this issue seemed to be merely an attempt to delay a decision for perhaps six years from now when the name *Racosperma* would have been taken up and the whole argument would be completely different. He therefore dismissed the taxonomic arguments altogether.

He had placed downstairs a paper on why the Committee had voted for conservation with a new type. Firstly, there were almost 1000 species in Australia that would otherwise be called *Racosperma*, which constituted by far the biggest genus on the continent, much larger, for example, even than *Eucalyptus*. He wondered how botanists in other countries would react if they had a genus of 1000 species and the name of those was threatened; would they not feel protective about 1000 species names? Secondly, there was a multi-billion agro-forestry industry based on the Australian species, which were now being grown on a vast scale in other tropical countries. This was just the sort of situation where it was vital for nomenclaturalists to adapt nomenclature to the user’s needs. Thirdly, the name *Acacia* had a much higher profile amongst the general public in Australia, where it was the national symbol as Orchard had explained, than in any other country, including Africa, as evidenced by the e-mail letters posted downstairs.

With respect to the e-mail letters displayed, he had not asked for those. There had been repeated newspaper articles and interviews about the name *Acacia*, and his e-mail address had been announced on national Australian radio. That is why he had received 150 e-mails in the space of three weeks! One was from the Minister for Environment and Heritage of Australia, but it even went higher in Australia than that, it was such an important issue for them, that the Prime Minister was informed about the debate in which the Section was engaged in Vienna that afternoon.

Fourthly, there was a massive horticultural industry in Australia based on their native species, which were used in a very great number of ways.

Fifthly, many of the 1000 or so species in Australia were restricted endemics which had attracted local and national legislation, and nomenclatural changes would affect the large numbers of scientists and administrators the government employ in connection with the genus. Australia had a huge investment in *Acacia*.

He wished to make one further observation, which he hoped would not offend anybody. He did not speak vernacular Australian, and was not sure of its significance, but it had been repeatedly reported in the e-mails that the name *Racosperma* had unfortunate connotations for a lot of Australians. He did not feel he need to go into that any further, and wondered if some Australian present could tell him just what connotation the word had.

If this proposal were not accepted, there were 14 times as many species in Australia that would have to change their names as in Africa. Not just twice, or five times as many, but 14 times as many. The figures were absolutely compulsive. Furthermore, outside Australia, 55% of the native species were going to change to *Senegalia* anyway, whatever decision was taken. Because of the cultivation and escape from cultivation of many Australian species outside the continent, many people in those countries already thought of *Acacia* as the Australian species. Retaining the name *Acacia* for fewer than half the species outside Australia would, in his opinion, lead to a worse situation than changing the whole lot. It would cause endless confusion in future in Africa, Asia, and South America if half the species there were real acacias and the other half were something different. That was a recipe for disaster in his opinion.

His final point was that nomenclaturalists must take note of the needs of those who use the names of plants. It was the users that mattered in all this, and he hoped he had presented the evidence to the section on this multi-billion dollar industry. He saw no case whatsoever for overthrowing the recommendation by two Committees. The Australian people were waiting for the Section to finally take the right decision; radios and newspapers were lined up in Australia to record what was decided. He had said that if this proposal was not supported, it would be the biggest injustice to users of plant names that he had ever witnessed. He believed that the Committee decisions were absolutely right and asked all the Section to support them, and when casting their votes to think – 1000 species and a multi-billion dollar industry.

**Smith** pointed out that reference had been made in some of the previous presentations to the fact that two learned Committees took a decision supporting 60% of the proposal as published in 2004. Barrie, he was sure, provided good-spirit guidance to the General Committee when he mailed out documentation. One such comment was that to move the matter forward it would be wise for the General Committee to vote in favour of this particular proposal. It could therefore easily be construed that 60% support had been solicited from the members. He was therefore very careful to regard it as two strong Committees independently making that decision. Beyond that, that the Section would require a 60% majority to overturn, would certainly be read very widely beyond the meeting as the nomenclatural taxonomic fraternity placing yet another hurdle placed in the path of taking a decision that would overturn the proposal to conserve. It was widely believed that decisions to change or amend the *Code* would require a 60% majority, but beyond that a simple majority would be required. As a final note, he pointed out that the national tree in South Africa was the Yellow-wood tree; it does not state which scientific species. The genus was *Podocarpus*, which was split sometime ago, so even in South Africa they were used to having the taxonomic difficulty of a national tree that sat in two genera.

**McNeill** stated that Smith was quite right that the 60% vote in the Committee for Spermatophyta was based on the merits or otherwise of the case to conserve with a new type. The 60% majority of the General Committee was based on an assessment of whether the Committee for Spermatophyta had taken account of all the facts, had followed the *Code*, and done all the right things. The General Committee was not making a judgement as to whether the decision was the right one. One was geared to the facts of the case, while the other was to ensure no facts had been overlooked and that the procedures had been duly followed, so there was a distinction. One Committee had looked at it expertly, and the other had checked that Committee had done its job properly.

**Unknown Speaker** was an Australian legume systematist who had worked all his career on *Acacia*; his PhD had included ecological studies on Australia’s most widespread species, *Acacia aneura*, mulga. He wished to make clear this was not a simple matter of parochialism, as there were Australians including himself who were opposed to moving the type of *Acacia* to Australia. He did not wish to go over the arguments that had been made already, but wished to clear up a couple of misconceptions by previous speakers. First, it was true that *Acacia* was an iconic species in Australia and the national flower, but what the Section had not been told was that it was not known as *Acacia* in the public mind, but was known as wattle; other names were used, such as mulga and brigalow for widespread and ecologically important species, and the majority of the public would not be aware that the generic name had been changed or not. Second, much had been made of the number of species in Australia, but the great majority were very restricted in distribution, very obscure, and known by very few people. It was just a few that were economically very important in cultivation overseas or were community dominants, and too much had been made of the sheer number of species. Third, above all countries that had *Acacia*’s occurring naturally in them, Australia was probably in the best position to deal with a large number of changes; their herbaria were well-curated and well-databased, so it would be a relatively small matter to co-ordinate the name changes.

**Arce Rico** had been working with *Acacia* for 30 years, and for the last 20 with *Ingeae*, the next tribe. Her heart was with her neotropical *Acacia* knowledge. In the neotropics acacia was the common name for *Acacia*’s. As she saw it, the subgenus *Phyllodineae* was the largest and where it started. Why retypify a genus with a species from a poorly sampled group? *Acacia penninervis*, suggested as the species to be used in the retypification, had not been sampled. Although she was not an expert on the subgenus, she had done her homework for the last 20 years when she studied and made mistakes with the *Ingeae*, and her best guess was that it would go with the *Botryocephalae* group. Orchard had mentioned that that the most profitable species was *Acacia mearnsii*, which belonged to the same group as *Acacia penninervis*. But *Acacia botrycephala* had bipinnate leaves and two phyllodes; she wondered if we were going to give our community the option of having bipinnate or phyllodenous acacias?

**Murphy** was also working on *Acacia* and the *Ingeae* group, and wished to raise a couple of points about the science. Earlier the point about chloroplast *versus* nucDNA had been raised. Now they had sampled roughly the same number of *Acacia* subgen *Phyllodineae* species in Australia and had almost exactly the same results for the ITS and ETS trees. The subgenus was well supported as a group. The taxonomy should not be in the debate, as it was very similar to what the Committee for Spermatophyta were presented with; three major groups equivalent to the subgenera traditionally recognized, plus two smaller segregates within the *Senegalia* group. Also, he wished to raise the point that within Australia acacia and wattle were equally well-used as popular names with no preference for either. Finally, there was no support from morphological or molecular evidence to show that the *Ingeae* would fall into subgenus *Phyllodineae*. He also wished to dispel the idea that this would not attract much attention in Australia; he thought it would raise a lot of attention if the name was changed.

**Gandhi** had described legumes from a part of India that had included scrub and rainforest. He was also serving as a consultant on the forthcoming checklist of the vascular plants of India, and had seen them as very common plants. The change would affect them considerably, but not as compared to the Australian number. Considering the overall picture, he had supported Brummitt’s position and still did.

**Pedley** wished to read two lines of a copy of an e-mail from Brummitt: “Dear Bruce, Sorry to be a bit slow in replying, any support for the *Acacia* recommendation can of course be passed on to me. As many as you can recruit would be helpful, particularly *Acacia* people outside Australia.”

**Linder** noted that much had been said about *Acacia* being the icon for Australia. The next time members saw a giraffe with a funny flat-topped tree and Mt Kilimanjaro in the background, maybe it would no longer be an *Acacia*, so that argument applied to Africa as well. Another thing which struck him, was that Africa and many third world countries did not have a nice strong developed lobby to send e-mails with carefully articulated and long arguments. The Section must be very careful about bringing too much of the public pressures from e-mails and phone calls from Prime Ministers into the argument. The argument was whether there was a sufficiently strong case to transfer a type from where it has been to another group.

**Hawksworth** wished to say that he had been in South Africa at the Forestry and Biotechnology Institute in Pretoria about three weeks earlier, and that they were extremely concerned about this issue, and were well-aware of the economically important trees. They had asked him to voice their strong objection to the current proposal to the Section.

**Mabberley**, like Brummitt, had started cutting his botanical teeth in Africa, and many of the acacias discussed with relation to Africa were in any case going to become *Senegalia*, in the same way that *Acacia albida* was lost to *Faidherbia* a few years ago. Having sat on the fence about this for some time, and having to compile a book that had to do with all sorts of plants besides *Acacia*, he had come down on the side of Australia because of the very simple argument that Brummitt had put, that about 13 times as many names would be saved by going along with the proposal. He was therefore very much in favour of it, not merely because of Australia, but because of their economic importance right the way round the world, and also because of their importance as weeds in many parts of the world, including Europe. To change the name of those to *Racosperma* would in his view be a backward step, so he was fully behind the two Committees.

**Luckow** wished to clear up that it was not 13 times, but somewhere around 6–7 times as many of species; that was a factual error. Also, the Committee for Spermatophyta in its first report acknowledged that very strong economic arguments could be made on both sides, but there was no one present to argue the economic importance for the African acacias. However, two people who live and work with *Acacia* at the Oxford Forestry Institute had found the idea that the Australian species were of much greater economic importance than the African ones to be laughable. In fact, one of the most economically important species was *Acacia nilotica*, the current type of the genus. She encouraged everyone not to get distracted by the political issues that were being presented, or the personal issues or attacks, but to consider whether or not when there was something clearly so controversial, if they wanted conservation to take precedence over priority. She felt that was the nomenclatural issue that should be addressed.

**Nicolson** stated that the Section had now heard 15 speakers, and he wished to know the sense of the meeting, and asked who would like to hear more before going to a vote. On a show of hands, the Section indicated that it was ready to vote, and a card vote was taken.

**McNeill** explained that a “yes” vote would be to approve the second part of the General Committee’s report (which included Committee for Spermatophyta Report No. 55), but that there would have to be a 60% “no” vote not to accept the Committee report.

The votes were 203: 247, but as the majority to overturn the second part of the Committee report was 54.9%, that part of the report was **approved.** The decision of the Committee on Spermatophyta to typify *Acacia* with the Australian species *Acacia penninervis* was therefore **confirmed.** [Applause.]

### Other Business

**McNeill** indicated that it was necessary for the Section to pass a motion to the following effect: “The Section instructs the Rapporteur-General to present a resolution to the Resolutions Committee of the XVII International Botanical Congress, to the effect that the Congress should approve the decisions of the Nomenclature Section.” The resolution would then be proposed by the Bureau of Nomenclature.

The motion was **approved.**

**Stuessy** drew attention to the IAPT Business Meeting that would follow immediately after the end of the session. The main business was to pay appreciation to those who had helped. The graduate students who had trained heavily for months so members did not have too many delays waiting for microphones, Jeong-Mi Park, Carolin A. Rebernig, Dieter Reich, and Ovidiu Paun. Chris Dixon managed the CD and tapes. Alessandra Ricciuti Lamonea helped on all organizational aspects, Elvira Horandl and Veronika Mayer. Turland and Nicolson kept the business moving along. The hot-spot was the Rapporteur-general?s position, which involved not only paying attention to and interpreting what was going on, but adding points where necessary and carrying the process forward. He could not figure when a better job had been done in all the sessions he had been to; a friendly attitude but the strength when needed – McNeill had done a fantastic job, and he was to be congratulated. [Loud applause.]

**McNeill** thanked Stuessy for his thanks. There was something that he might have said at the beginning, that he thought it was true that the longest standing member of the Section, who had even attended the Paris Congress, was Paul Silva. He wished the Section to recognize his long commitment to nomenclature, and that it would long continue. [Applause.]

**Nicolson**, in closing the Nomenclature Section of the XVII International Botanical Congress, thanked everyone for their contributions. He hoped he had not interfered, and that members were satisfied to have had so large a group together for such a long time. He bid all “goodbye for now”.

## Appendix A

**Taxonomists who died since the XVI International Botanical Congress in St Louis in 1999 (or overlooked in previous lists)**

Compiled by Dan H. Nicolson

This listing of deceased botanists tries to include in addition to the birth and death years:(1) the place and date of birth, (2) the date and place of death, and (3) the source(s) of information., as was done in prior listings (Taxon 48: 785–788. 1999; 42: 929–930. 1993; Englera 9: 10–11. 1989, the last with names only).

* = previously overlooked.

Abdallah, Moustafa Sayed-Ahmed (8 Apr 1918- 22 Oct 2001 Cairo, Egypt) – Taxon 51: 219. 2002.

Adams, Charles Dennis (23 Mar 1920- 27 Mar 2005 London, UK) – e-mail from Kew via G. Davidse.

Aguilar, Norma O. (- 2 Nov 2003 age 69 Los Banos, Philippines) – Fl. Males. Bull.13(3): 243. 2003.

Anderson, Edward Frederick “Ted” (Covina, California 17 Jun 1932- 29 Mar 2001 Phoenix, Arizona) – Taxon 50: 939–942. 2001.

Andersson, Bengt Lennart (Alingsas, Sweden 18 Sep 1948- 1 Jan 2005 Goteborg, Sweden) – Taxon 54: 1108. 2005 [2006].

Andrews, Henry Nathaniel, Jr. (Melrose, Massachussetts 15 Jun 1910- 3 Mar 2002 Concord, New Hampshire) – Pl. Sci. Bull 48: 48–49. 2002.

Baker, Herbert George (Brighton, UK 23 Feb 1920- 2 July 2001 Oakland, California) – FNA Newsletter 15(3): 20. 2001; Taxon 50: 1249–1253. 2001.

*Baden, Claus (1952–1990). Danish.– Note from Rosemary Davies (Kew).

Bangerter, Edward Benedict (London 1911- 2001 Aukland, N.Z.) – Watsonia 24: 128–129. 2002.

Barkley, Theodore Mitchell “Ted” (1934- 24 Jul 2004) – ASPT Newsl. 18(2): 3–4. Dec 2004.

Barneby, Rupert Charles (Monmouthshire, England 6 Oct 1911- 5 Dec 2000 Bronx, New York) – Fl. N. Am. Newsletter 14(4): 23. 2000; Sida 19: 745–751. 2001; ASPT Newsletter 15(1): 3. 2001; Taxon 50: 285–292. 2001 (bibl.).

Barroso, Graziela Maciel (Corumba, Mato Grosso 11 Apr 1912- 5 May 2003 Rio de Janiero) – Taxon 53: 225–226. 2004.

Bassett, Ivan John (Lethbridge, Alberta 27 Sep 1921- 7 Aug 2000 Ottawa) – BEN 255.

Baytop, Turhan (Uskudar (Scutari) Turkey 1920- 25 Sep 2002 Istanbul) – Plant Talk 30: 11. 2002.

Beamish, Katherine (‘Kay’) I. (Winnipeg, Manitoba 25 Jun 1912- 4 Feb 2003 Chemainus) – BEN 306.

Beauglehole, Alexander Clifford (26 Aug 1920- 19 Jan 2002 New Zealand?) – Australian Syst. Bot. Soc. Newsl. 111: 13. June 2002.

Benjamin, Richard Keith (9 Apr 1922- 30 Apr 2002) – Taxon 51: 430. 2002.

Benl, Gerhard (Nurnberg 18 Dec 1910- 29 Aug 2001 Munich) – Sendtnera 8: 195–204. 2002 (bibl.).

Bernardi, Luciano (Bologne, Italy 6 Mar 1920- 1 Dec 2001 Geneva) – Fl. N. Am. Newsletter 15(4): 23. 2001; Candollea 56(2): 363–376. 2002 (portr., bibliogr., eponymy).

Birdsey, Monroe Roberts (1922- 15 Jan 2000 Miami, Florida) – [International Aroid Society] Newsletter 22(1): 5. 2000.

Boomsma, Clifford David (20 Oct 1915- 13 Jan 2004) – Australian Syst. Bot. Soc. Newsl. 118: 12–15. (bibl.)

Bos, Jan Justus (28 Jul 1939- 24 Mar 2003) – Bull. Bot. Tuinen Wageningen 43: 2–8. 2003 (n.v.).

Bowra, John Charles (1918- Aug 2002) – Watsonia 24: 589–591. 2003 (bibl.).

Brewis, Anne Beatrice Mary (26 Mar 1911- 31 Mar 2002)- born Anne Palmer – Watsonia 24: 24: 464–465. 2002.

Cannon, Margaret Joy (1928- 2002) – Watsonia 24: 587–594. 2003.

Canoso, Michael Anthony [Mike] (Diamondville, Wyoming 29 Jul 1920- 29 Aug 2005 Cambridge, Massachusetts) – ASPT Newsletter 19(2): 4–5. 2005.

Carvalho, Andre Mauricio Vieira de (5 Oct 1951- 16 Nov 2002) – Taxon 52: 147–148. 2003.

Cernohorsky, Zdenek (Chroustovice 27 Dec 1910- 5 Sep 2001 Prague) – Preslia 74: 85–86. 2002.

Chang Kiaw Lan (Aceh, Sumatra 31 Jul 1927- 14 Aug 2003 Singapore) – Fl. Males. Bull. 13(3): 261. 2003; Gard. Bull. Singapore 55: 309–315. 2003; Fl. Males. Bull. 13(4): 349–350. 2005.

*Chaney, Ralph Works (1890–1971) – Ma in Aliso 21: 74. 2004.

*Cheng, Wan-Chun (Wan-Jun Zheng) (1904–1983) – Ma in Aliso 21: 74. 2004.

*Chun, Woong Young (Chen HuanYung) (Hong Kong 6 Jun 1889- mid Jan 1971).

Constance, Lincoln (Eugene, Oregon 16 Feb 1909- 11 Jun 2001) – ASPT Newsletter 15(1): 3. 2001.

Coombe, David Edwin (9 Mar 1927- 28 Jun 1999) – Watsonia 23: 360–367. 2000 (bibl.).

Correa, Maevia Noemi (Buenos Aires 14 Feb 1914- 2005) – Bonplandia 14: 321–322. 2005 (photo).

Craig, William Greene (Geneva, New York 1949- 2 Oct 2003 Bar Harbor, Maine) – ASPT Newsletter 17(2): 7. 2003.

Crum, Howard Alvin (Mishawaka, Indiana 14 Jul 1922- 30 Apr 2002 Green Bay, Wisconsin) – BEN 289, Bryologist 106(1): 9–23. 2003.

Culberson, William Louis (1929- 8 Feb 2003) – Lichenologist 35: 93–95; Pl. Sci. Bull. 49(2): 49. 2003.

D’Arcy, William (29 Aug 1931- 16 Dec 1999 St. Louis, Missouri) – Jan-March & July 2000 ASPT Newsletter, p. 4.

Davidian, Hagop Haroutune (1 Apr 1907- 15 Apr 2003 Edinburgh) - J. Amer. Rhododendron Soc. 57(4): 209–210. 2003; Rhododendrons Camellias Magnolias 2005: 79–80. 2004 (portr.), n.v.

Davies, Elizabeth ‘Liza’ Winsome [married name Woodward] (1924–2002: married in 1958) – Watsonia 24: 461–462. 2003.

DeFilipps, Robert Anthony (Chicago, Illinois 4 Mar 1939- 4 Jul 2004 Washington, DC) Plant Press (Smithsonian), n.s., 7(3): 6. 2004.

Dennis, Richard William George (Thornbury, UK 13 Jul 1910- 7 Jun 2003) – Mycologist 96: 187–189. 2004.

de Wit, Hendrik Cornelis Dirk (24 Oct 1909- 16 Mar 1999) – Taxon 48: 847–848. 1999.

Dickison, William Campbell (Flushing, New York 12 Mar 1941- 22 Nov 1999 Chapel Hill, North Carolina), plant anatomist – Taxon 49: 390. 2000.

*Dore, William G. ‘Bill’ (1912- 17 Apr 1996 Ottawa, Canada) – BEN 135.

Dostal, Josef (Prague 20 Dec 1903- 11 May 1999 Prague) – Taxon 48: 852–854. 1999.

Douglas, George Wayne (New Minister, British Columbia 22 Jun 1938- 10 Feb 2005 British Columbia) – BEN 344.

Druce, Anthony Peter (18 Jun 1920- 15 Mar 1999) – New Zeal. J. Bot. 37: 573–577. 1999.

Ducker, Sophie Charlotte (nee Klemperer) (Berlin 1909- 20 May 2004 Melbourne) – Austral. Syst, Bot. Newsl. 120: 20–25. 2004 (with bibl.).

Duncan, Wilbur Howard (Buffalo, New York 15 Oct 1910- 25 Mar 2005 Athens, Georgia) – Plant Sci. Bull. 51: 51. 2005; ASPT Newsl. 19(1): 3. Jun 2005; Sida 21: 1941–1950. 2005.

Dwyer, John Duncan (Newark, New Jersey 1915- 6 Dec 2005 St. Louis, Missouri)-

El-Hadidi, Mohammed Nabil (1934- 2 Sep 2003 Cairo) – Taxon 53: 607–609. 2004 (bibl.).

Emygdio de Mello Filho, Luiz (31 Oct 1913- 16 Jun 2002 in Rio) – Taxon 51 825. 2002 [2003].

Ewan, Joseph Andorfer (Philadelphia, Pennsylvania 24 Oct 1909- 5 Dec 1999 Mandeville, Louisiana), botanical historian. – Taxon 49: 107–112. 2000.

Ewan, Ada Nesta Dunn (Saskatchewan, Canada 8 Nov 1908- 8 Dec 2000 Mandeville, Louisiana) – Taxon 49: 817–818. 2000.

Faegri, Knut (Bergen, Norway 17 Jul 1909- 10 Dec 2001 Bergen) – Taxon 51: 391–392. 2002; Watsonia 24: 462–464. 2003.

Fernandes, Rosette Mercedes Saraiva Batarda Fernandes (1916–2005), widow of Albilio Fernandes of Coimbra. – e-mail from R. K. Brummitt.

Ferreyra Huerta, Ramon Alejandro (Lima, Peru 26 Feb 1910- 4 Jun 2005 Lima, Peru) – e-mail with bibliogr.

Fischer, Tharl Richard (1921- 1999) – ASPT Newsletter Mar 2000.

Foldats Andins, Ernesto (Liepja, Latvia 15 May 1925- 16 Jan 2003 Caracas, Venezuela) – Speckmaier, Orchidee 54(3): 288–289. 2003..

Foreman, Donald (‘Don’) Bruce (1945- 2004) – Austr. Syst. Bot. Soc. Newsl. 118: 12. 2004. (mentioned); l.c. 119: 7–18. 2004 (publ., taxa); Fl. Males. Bull. 13: 350. 2005.

Frankton, Clarence (‘Clarrie’) (6 Feb 1906- 11 Jun 2000 Ottawa, Ontario) – BEN 253.

*Fu, Shu-Hsia (Fu ShuXia) (1916- 1986) – Ma in Aliso 21: 75. 2004.

Geissler, Patricia (25 Jul 1947- 29 Mar 2000 Geneva, Switzerland) hepaticologist.

Gervais, Camille (Montreal 2 Dec 1933- 28 Nov 2002 Sainte-Foy) – Naturaliste Canad. 128: 128(1): 5–17. 2004 (photo, bibl.)

Given, David Roger (1943- 27 Nov 2005 Christchurch, New Zealand) – e-mail.

Godfrey, Robert (‘Bob’) Kenneth (1911- 6 Feb 2000) – Jan-Mar ASPT Newsletter p. 4 & 6; ASPT Newsletter 15(1): 4–5. 2001.

Goodman, George Jones (Evaston, Wyoming 5 Nov 1904- 23 May 1999).

Graf, Alfred Byrd (1901- 14 Dec 2001) – Taxon 51: 249. 2002.

Grassi, Marta Maria (Buenos Aires 17 Dec 1921- 18 Jun 2005 San Miquel de Tucuman). algologist – Bonplandia 14: 323–324. 2005.

Greene, Craig W. (?- 20 Oct 2003 at Mount Desert Island, Maine) – Fl. N. Am. Newsl. 17(2): 15. 2003.

Gregory, Luis Enrique (San German, Puerto Rico 1913?- 15 Sep 2004 Lanham, Maryland) – Washington Post obituary 11 Oct 2004.

Grolle, Riclef (8 Aug 1934- 12 Jun 2004 Jena, Germany) – Trop. Bryol. Group 19: 1. 2004.

Gubanov, Ivan (14 Jan 1933- 12 Feb 2005)

Haas, Robert (1920- 27 Nov 2005 Boblingen, Germany) – letter (studied succulents).

Hadac, Emil (Bohdanec 10 May 1914–23 Apr 2003 Prague, Czechoslovakia) – BEN 311.

Hamer, Fritz (Hamburg, Germany 22 Nov 1912- 13 Jan 2004 Sarasota, Florida) – Selbeyana 25: 245–247. 2005.

Hansen, Bertil (Varde, Denmark 27 Mar 1932- 25 Apr 2005 Malaga, Spain) – from Ib Friis.

Hart, Henkt ‘t- see ‘t Hart, Henkt

Heimsch, Charles (Dayton, Ohio 4 May 1914- 23 Apr 2003 Moscow, Idaho) – Plant Sci. Bull. 49(2): 51–53. 2003.

*Henderson, Douglass Mackay (Long Beach, California 9 Jul 1938- 24 Jul 1996 Moscow, Idaho) – BEN 142.

Hendrych, Radovan (1926–2004) – Preslia 77: 333–337. 2005. (biblio, portr.)

Henty, Edward Ellis “Ted” (Hamilton, Victoria Oct 1915- 23 Feb 2002 East Keilor near Melbourne, Australia) – Austral. Syst. Bot. Soc. Newsl. 110 (Mar): 8–9. 2002: Fl. Males. Bull. 13(2): 161–168. 2002.

Holub, Josef (5 Dec 1930- 23 Jul 1999) – Taxon 48: 849–850. 1999.

Howard, Richard Alden (Stamford, Connecticut 1 Jul 1917- 18 Sep 2003 Cohasset, Massachusetts) – Taxon 52: 871–872. 2000; ASPT Newsletter 17(2): 5–7. 2003; Rhodora 106: 178–184. 2004.

Hsueh, Chi-Ju see Xue, JiRu

Hu, Hsen-Hsu (see Hu, Xian-Su)

*Hu, Xian-Su (1894- 1968) – Ma in Aliso 21: 75. 2004.

Hunziker, Armando Theodoro (Chacabuco, Argentina 29 Aug 1917- 12 Dec 2001 Cordoba, Argentina) – ASPT Newsletter 16(1): 3. 2002; Taxon 51: 393–403. 2002 (bibl.).

Hunziker, Juan Hector (1925- 17 Mar 2003) – Honorary Director Darwinion Institute, Buenos Aires, Argentina.

Hylmo, Bertil (1915- 20 Jun 2001 in Bjuv, Sweden). – Specialist in *Cotoneaster*. FNA Newsletter 15(3): 20. 2001.

Inoue, Ken (1948- 2003) – J. Jap. Bot. 79: 73–77. 2004.

Isely, Duane (1918- 6 Dec 2000 Des Moines, Iowa) – Fl. N. Am. Newsl. 14(4): 24. 2000; ASPT Newsletter 15(1): 3. 2001.

Jalas, Arvo Jaakko Juhani (Hameenlinna, Finland 7 May 1920- 1 Dec 1999 Helsinki) – Taxon 49: 321–323. 2000.

Joseph, J. (1928- 14 Aug 2000) – Rheedea 10: 161–162. 2000.

Katende, Tony B. (?- 2005 Uganda) – e-mail.

Khan, Mohammad Salar (1924- 30 Sep 2002 at Dhaka, Bangladesh) – e-mail.

*Kiah bin Haji Mohamed Salleh (- 12 Sep 1982 Singapore) – Fl. Males. Bull. 13(4): 350. 2005.

Kitamura, Siro (22 Sep 1906- 21 Mar 2002) – Acta Phytotax. Geobot. 53: 89–94. 2002.

Knobloch, Irving William “Knobby” (Buffalo, New York 1907- 27 Dec 1999 in East Lansing, Michigan [?]) – pteridologist.

Kozhenvikov, Yury Pavlovich (1942- 2002) – Bot. Zhurn. 88: 141–147. 2003 (bibl.).

Krug, John Christian (1938- 29 Aug 2005 Ontario, Canada) – mycologist, e-mail.

*Lakela, Olga Korhoven (Kestila, Finland 11 Mar 1890- 1980) – (Englera 2: 7. 1982, Englera 9: 10–11. 1989 and Taxon 42: 929–930. 1993).

Lamoureux, Charles H. (1933- 16 Oct 2000) Director of the Lyon Arboretum in Manoa Valley, part of the University of Hawaii).

Lange, Morten (Fyn, Denmark 24 Nov 1919- 10 Nov 2003 Holte, Denmark) – Nordic J. Bot. 23(2): 153–154. 2004.

Leenhouts, Pieter Willem (1926- 1 Mar 2004) – Blumea 49: 3–10. 2004 (portr., bibliogr.)

Lewalle, Jose (Liege, Belgium 1931- 5 Apr 2004 Rabat, Morocco) – Syst. Geogr. Pl. 75: 3–6. 2005 (bibl., photo).

Lisowski, Stanislas (Vilnius 11 Feb 1924- 2 May 2002 Poznan, Poland) – AETFAT Bull. 47: E49. 2003 & Syst. Geogr. Pl. 74: 9–16. 2004 (recent bibl.).

Little, Elbert Luther (Fort Smith, Arkansas 18 Oct 1907- 18 Jun 2004 Eugene, OR) – announcement rec’d 20 Aug 2004.

Llano, George Albert (born Jorge Alberto Cecilio Perez y Llano in Cuba 22 Nov 1911- 8 Feb 2003 on cruise ship to Falkland Islands). – The Bryologist 107: 388–391. 2004.

Lothian, Thomas Robert Noel (Mont Albert, Melbourne, Victoria Australia 25 Dec 1915- 24 Sep 2004 Adelaide?) – Austral. Syst. Bot. Newsl. 120: 26. 2004.

Lourteig, Alicia (Buenos Aires 17 Dec 1913- 30 Jul 2003 Paris) – Bonplandia 12: 157–161. 2003 (portr., bibl.); Adansonia ser. 3, 25: 149–150. 2003 (portr.).

Love, Doris Benta Marie (Kristianstad, Sweden 2 Jan 1918- 25 Feb 2000 ?San Jose, California) – BEN 242.

Lozano-Contreras, Gustavo (Bogota, Colombia 22 May 1938- 10 Jul 2000 Bogota, Colombia) – Caldasia 23(2): 343–344 & 345–350. 2001. (bibl.)

Lundin, Roger (1955 - 15 Nov 2005 Stockholm, Sweden). – E-mail.

Manning, Wayne Eyer (Toledo, OH 12 Apr 1899- 8 Feb 2004 Lewisburg, Pennsylvania) – ASPT Newsletter 18(1): 3. Jul 2004; Pl. Sci. Bull. 50: 51. 2004.

Matteri, Celina Maria (1943–30 Nov 2004 Buenos Aires, Argentina) – Cryptog., Bryol. 26(1): 121. 2005; Bonplandia 14: 103. 2005 (portr.).

Matthew, Koyapillil Mathai (16 Mar 1930- 16 Apr 2004 Tiruchirapalli, Tamil Nadu) – Plant Talk 36: 10. 2004; Taxon 53: 1107–1008. 2005 (‘2004’).

Mayer, Ernst (1904–3 Feb 2005) – Washington Post 5 Feb 2005 obituary.

McClintock, David Charles (4 July 1913- 23 Nov 2001) – Bamboo 23(1): 23–24. 2002; Watsonia 24: 257–266. 2002 (bibl.).

McClintock, Elizabeth (Los Angeles, California 1912- 19 Oct 2004 Santa Rosa, California) –FNA Newsl. 18(2): 14. 2004; Fremontia 33(2): 30. 2005.

Meijer, Willem (The Hague, Netherlands 27 Jun 1923- 22 Oct 2003 in Lexington, KY) – Fl. Males. Bull. 13(3): 243. 2003; Sandakania 15: 1–24 2004.

(Mello, Luiz Emygido de) see Emygdio de Mello, Luiz

Menitsky, Yuri Leonardovich (1937- 2001) – Bot. Zhurn. 87: 145–151. 2002.

Mohamed Shah bin Mohamed Noor (Singapore Bot. Garden 22 Jan 1935- 18 Jun 2003 Singapore). – Fl. Males. Bull. 13(3): 262. 2003.

Monson, Paul Herman (Fargo, North Dak. 29 Sep 1925- 9 Aug 2003 Duluth, Minn.) – Fl. N. Am. Newsl. 17(2): 15. 2003.

Moore, Ted (?- 2003? at 95) – Australian Syst. Bot. Soc. Newsl. 117: 24–26. 2003.

Morley, Thomas (1917- 2 Feb 2002 Minneapolis, Minn.) – ASPT Newsletter 16(1): 3. 2002; Pl. Sci. Bull 48: 48. 2002.

Morris, Denis Ivor (1924–2005) – Australian Syst.Bot. Soc. Newsl. 124: 8. 2005.

Morton, Alan Gilbert (Prescot, Lancastershire 12 Mar 1910- 19 Mar 2003, Edinburgh?) – Soc. Hist. Nat. Hist. Newsl. 81: 17–18. 2005.

Moseley, Maynard Fowle (Boston, Massachusetts 15 Jun 1932- 16 Jan 2003, Santa Barbara, California) – Plant Sci. Bull. 49: 7. 2003.

Moser, Meinhard Michael (Innsbruck, Austria 13 Mar 1924–30 Sep 2002) – Mycol. Research 107(4): 506–508. 2003 (n.v.).

Normand, Didier (Troyes, Champagne, France 30 Jul 1908- 6 Aug 2002) – AETFAT Bull. 47: F25–27, E49. 2003.

Nowak, Janusz (1931- 24 Dec 2004 Krakow, Poland) – lichenologist, e-mail forwarded by Rosemary Davies.

Nyholm (nee Tufvesson), Elsa Cecilia (Nordana 1911- Autumn 2002 Lund?) – J. Bryol. 25: 145–146. 2003.

Obermeyer-Mauve, Anna Amelia (Pretoria 30 Jul 1907- 10 Oct 2001 Pinelands, Cape Town) – Bothalia 32: 127–130. 2002.

Oliver (nee Nitzsche), Inge (South Africa 18 Dec 1947- 7 Jul 2003 Stellenbosch) (wife of E. G. H. Oliver) – http://www.stellenboschwriters.com/olivering.html

Ormonde, Jose Eduardo Martins (1943- 12 Jan 2004 Azores) – Botanica Complutensis 27: 267. 2003.

Ornduff, Robert (Portland, Oregon 13 Jun 1932- 22 Sep 2000 Berkeley, California) – ASPT Newsletter 14(3): 3. 2000; 14(4): 24–25. 2000; Taxon 49: 819. 2000.

Palamarev, Emanuel (Sofia 13 Apr 1933- 28 Jan 2004 Sofia) – Taxon 53: 605–606. 2004; Feddes Repertorium 115: 217–219. 2004; Festschrift: Phytologica Balacanica 9(2): 2003 (bibl.).

Palmer, Anne: see Brewis, A. B. M.

Pancho, Juan Valles (Philippines 30 Jan 1925- 20 May 2000 Philippines) – Fl. Males. Bull. 12: 356. 2001.

Panigrahi, Gopinath (India 27 Feb 1924- 15 Dec 2004) – info ftom R.K. Brummitt 7 Mar 2005.

Parham, John Willoughby (Christchurch, NZ 30 Mar 1929- 27 Sep 2002 Sandy Bay, Tasmania) – Austrobaileya 6(3): 575–579. 2003.

Parks, James C. (Altoona, Pennsylvania 9 Aug 1942- 23 Dec 2002 Millersville, Pennsylvania) - Plant Sci. Bull. 49: 8. 2003; Bartonia 62: 109–111. 2004.

Pavlick, Leon Edward (1939–2003) – BEN 319.

Pedersen, Troels Myndel (Denmark 26 Sep 1916- 5 Feb 2000 Corrientes, Argentina) – Taxon 49: 573–575. 2000.

Pedralli, Gilberto (Bento Goncalves, RS 7 Nov 1953- 4 Dec 2003 Belo Horizonte) – Pabstia 15: [9]. 2004.

Philipson, William Raymond (Newcastle upon Tyne 6 Dec 1911- 28 Mar 1997 New Zealand).

Pichi-Sermolli, Rodolfo Emilio Giuseppe (Florence, Italy 24 Feb 1912 - 21 Apr 2005 Montagnana (Florence) Italy) – G[roup of]E[uropean]P[teridologists] News-13: 4–7. Dec 2005.

Port, Peter John (1926- 2003) – J. Bryol. 26: 153. 2004.

Porter, Cedric Lambert ‘Ted’ (Jugranwala, now Pakistan 15 Jan 1906- 8 Jan 2000 Peoria, Arizona) – Taxon 49: 577–580. 2000 (bibl.); Fl. N. Am. Newsl. 15(1): 7. 2001.

Potzdal, Eva Hedwig Ingeborg (22 Dec 1924- 2 Jul 2000) – Taxon 49: 606. 2000.

Prochazka, Frantisek (30 Mar 1939 - 29 Sep 2004) – Preslia 77: 327–329. 2005. (bibl.)

Rauh, Werner (1913- 7 Apr 2000), University of Heidelberg.

Read, Robert William (Woodbury, New Jersey 13 Dec 1931- 15 July 2003 Naples, Florida) – Taxon 52: 869. 2003.

Redeuilh, Guy (Bellou, Charentes, France 6 Mai 1937- 5 Oct 2004 St. Germain-en-Laye (near Paris), France) – info rec’d at 2005 Congress.

Reese, William Dean (10 Sep 1928- 4 Feb 2002) – FLA Newsletter 16: 3. 2002; J. Bryol. 25: 231–232. 2002 [2003] (bibl.); Sida 20(3): 1335–1338. 2003.

Renz, Jany (25 Jun 1907- 10 Aug 1999) Orchidologist – Taxon 48: 845–846. 1999.

Roberts, Richard Henry (‘Dick’) (Llanllechid, Wales 1910- 2003) – Watsonia 25: 221–223, 2004 (bibl.)

Robyns, Andre (Louvain, Belgium 13 May 1935- 2003) – Syst. Geogr. Pl. 74: 3–8. 2004 (bibl.)

Rogerson, Clark Thomas (2 Oct 1918- 7 Sep 2001 Ogden, Utah) – FNA Newsletter 15(3): 20. 2001; Taxon 51: 249. 2002.

Ross, Robert (Pinner, Middlesex 14 Aug 1912- 25 May 2005 England) – London Times obit..

*Rossler, Wilhelm (Mailand, Austria 10 Oct 1909- 14 Sep 1995 Graz?) – Mitt. Naturwiss Ver. Steiermark 126: 17–19. 1996.

Royen, Pieter van (Lahat, Sumatra 8 Jan 1923- 1 Dec 2002 Portland, Oregon) – Fl. Males. Bull. 13: 262. 2003.

Rudd, Velva Elaine (Fargo, N. Dak. 6 Sep 1910- 9 Dec 1999 Reseda, California) – Plant Press n.s. 3(1): 8. 2000.

Russell, Dekalb (Washington, DC 16 Mar 1918 Washington, DC- 24 Nov 2001 Clearbrook, Virginia) – Taxon 52: 641–642. 2003.

Ryan, Bruce Douglas (Los Angeles, Calif. 13 Sep 1950- 21 Jan 2004 Tempe, Arizona) – lichenologist BEN 322.

Saldanha, Cecil John (Bandra, India 27 Dec 1927- 7 Apr 2002 Bangalore, India) – Taxon 51: 585–587. 2002.

Satomi, Nobuo (2 June 1922- 2002) – J. Jap. Bot. 77: 357. 2002.

Savile, Douglas Barton Osbourne (Dublin, Ireland 19 Jul 1909- 1 Aug 2000 Ottawa, Canada) – BEN 256; Mycol. 93: 807–813. 2001; Canad. Field-Nat.. 115(2): 357–364 (tribute) & 365–380 (autobiogr.). 2001

Schroeter, Alexej I. (Bashkiria 15 Mar 1918- 22 Jul 2002 Moscow, Russia) – Taxon 52: 149–150. 2003.

Schubert, Bernice Giduz (Boston, Massachusetts 6 Oct 1913- 14 Aug 2000 Cambridge, Massachusetts) – Fl. N. Am. Newsl. 14: 16–17. 2000; ASPT Newsletter 14(3): 5. 2000; Taxon 49: 821–827. 2000 (bibl.).

Schultes, Richard Evans (Boston, Massachusetts 12 Jan 1915- 10 Apr 2001 Boston) – Boston Globe 14 Apr 01, Prance in Econ. Bot. 55: 347–362. 2001 (photo, bibl.).

Schulze, Gerhard (Dresden, Germany 16 Feb 1912- 9 Jul 2005 Altlussheim, Germany) – G[roup of] E[uropean] P[teridologists] News 13: 2–3. Dec 2005.

Sedova, Tatyana Vladimirovna (1934- 2002) – Bot. Zhurn. 88: 181–185. 2003 (bibl.).

Seidenfaden, Gunnar (Bornholm, Denmark 24 Feb 1908- 9 Feb 2001 Borsholmgard) – Thai For. Bull., Bot. 29: 188–189. 2001 (portr.); Taxon 51: 405–411. 2002 (bibl.).

Senghas, Karlheinz (1928- 4 Feb 2004) – Orchid Review 112: 143. 2004.

Senn, Harold A[rchie?] (Caledonia, Ontario 12 Jan 1912- 22 Jan 1997 Victoria, British Columbia) – overlooked BEN 165.

*Skabichevsky, Alexandr Pavlovitch (1904- 1990) – Bot. Zhurn. 88: 142–147. 2003.

Skutch, Alexander Frank (20 Mai 1904 Baltimore, Maryland- 12 May 2004 Costa Rica) – New York Times, 7 Jun 2004 obituary.

Slavik, Bohumil (14 Jan 1935- 13 Jan 2004) – Preslia 77(1): 137. 2005.

Smith, Philip Morgans (Halesowen, West Midlands 1941- 14 Jan 2004 Edinburgh) – Watsonia 25: 225–227. 2004.

Smythies, Bertram Evelyn ‘Bill’ (Naini Thal, India 11 Jul 1912- 27 Jun 1999) – Watsonia 23: 370–371. 2000; Fl. Males. Bull. 13: 134. 2002.

Stearn, William Thomas (Cambridge, UK 16 Apr 1911- 9 May 2001, Kew) – London Times; Taxon 50: 1255–1276. 2001 (bibl.); Watsonia 24: 123–124. 2002.

Stebbins, George Ledyard (Lawrence, New York 6 Jan 1906- 19 Jan 2000 Davis, California) – Jan-Mar. ASPT Newsletter, p. 6; Taxon 49: 581–583. 2000.

*Stefanov, Boris (Sophia, Bulgaria 8 Jun 1894- 12 Dec 1979 Sofia[?]) – Phytol. Balcanica 11(1): 3–23. 2005.

Streimann, Heinar (Tartu, Estonia 19 Dec 1938- 29 Aug 2001 Canberra, Australia) – Taxon 51: 413–414. 2002.

Sundberg, Scott Donald (Eugene, Oregon 10 Feb 1954 - 30 Dec 2004 Corvallis, Oregon), coordinator, Flora of Oregon Project – FNA Newsl. 18(2): 14. 2004; BEN (Bot. Electr. News) 343 10 Feb 05; Pl. Sci. Bull. 51: 52. 2005.

‘t Hart, Henk (1944- 22 Jul 2000 Utrecht) – Taxon 49: 829–830. 2000.

Taylor, Raymond John (1930- 2004) – Iridos 15(3): 14. 2004.

Teryokhin, Edward Semenovitsa (1932- 1 Dec 2002)

Tharl, Richard Fischer (1921- 11 Feb 2000 in Queenstown, NZ on vacation from Bowling Green, OH). – ASPT Newsl. 14(2): 3. 2000.

Thieret, John William (1926- 7 Dec 2005 in Highland Heights, Kentucky) – E-mail.

*Tippo, Oswald (Milo, Maine 27 Nov 1911- 10 June 1999 Santa Barbara, California).

Tryon, Rolla Milton, Jr. (Chicago, Illinois 20 Aug 1916- 20 Aug 2001 Tampa, Florida) – Rhodora 104: 92–95. 2002; Amer. Fern J. 92(1): 1–9. 2002 (bibl., portr.).

Valdes Guttierez, Javier (2 Oct 1931- 17 Jun 2004) – Bol. Soc. Bot. Mexico 75: 143–146. 2004.

Vartak, Vaman Dattatraya (1925–2001) – Botanist at Pune (Poona) e-mail from Gandhi.

Villiers, Jean-Francois (Paris, France 29 Mar 1943- 22 Mar 2001).

Volk, Otto Heinrich (1903- 2002 Wurzburg, Germany) – AETFAT Bull. 47: E49. 2003.

Wagner, Warren Herbert, Jr. “Herb” (Washington, DC 29 Aug 1920- 8 Jan 2000 U. Michigan) – Taxon 49: 585–592. 2000 (bibl.).

Wang, Zhan (Dandong, Liaoning Prov., China 4 May 1911- 30 Jan 2000) – Taxon 49: 593–601. 2000.

Ward, George Henry (1916–2003) – ASPT Newletter 17(2): 7–8. 2003.

Webster, Grady Linder, Jr. (Ada, Oklahoma 14 Apr 1927- 27 Oct 2005 Davis, California) - ASPT Newsl. 19(2): 3–4. 2005; Syst. Bot. 31(1): 1. 2006 (in press),.

Werth, Charles Richard (Soeul, Korea 1948- 27 July 2001 Alexandria, Virginia) – obit in Washington Post: C8. 5 Aug 2001. FNA Newsletter 15(3): 20. 2001.

Wiehler, Hans Joaquim (Klettendorf, East Prussia [now Poland] 8 Jul 1930- 1903

Williams, John Beaumont (Sydney, Australia 12 Feb 1932- 2005 Armidale, Australia) – Australian Syst. Bot. Newsl. 124: 9–14. 2005 (bibliogr,)

Whitmore, Timothy Charles (Ruislip, UK 9 Jun 1935- 14 Feb 2002 Cambridge, UK) – Fl. Males. Bull. 13: 134–136. 2002; Palms 46(2): 56. 2002.

Woodbury, Roy Orlo (Montpellier, N. Dakota 29 Nov 1913- 21 Sep 2002 in Martin Co., Florida) – Taxon 52: 391. 2003; l.c. 53: 227. 2004.

Woods, Patrick (Ballinderry, N. Ireland 3 Jul 1932- 28 May 2004) – Orchid Rev. 112: 314–315. 2004 (portr.).

Woodward: see Davies, E. W.

Wright, Jorge Eduardo (1922–4 Jan 2005 Corrientes, Argentina) – Bonplandia 14: 101–102. 2005 (portr.).

Wyatt-Smith, John (Swatow, China 29 Jan 1917- 30 Oct 2002 Oxford, U.K.) – Fl. Males. Bull 13: 268. 2003.

Vanden Berghen, Constant (Brussels, Belgium 21 May 1914- 5 Nov 2004) – Syst. Geogr. Pl. 75: 7–24. 2005.

Xue, Ji-Ru (1921–1999) also known as Hsueh, Chi-Ju – Ma in Aliso 21: 75. 2004.

Yurtsev, Boris Alexandrovich (15 Mar 1932- 14 Dec 2004 St. Petersburg, Russia) – Fl. N. Am. Newsl. 19(1): 9. 2005.

Zahulskyy (Zagulsky), Mykhaylo (Pidlyssya L’viv Oblast, Ukraine 10 Aug 1960- 27 Jan 2002 Lviv, Ukraine) – Phyton (Horn) 44(2): 309–318. 2004 (photo, bibl.).

Zhu, Guanghua (Manzhouli, Inner Mongolia, People’s Republic of China 17 Jan 1964- 2 Nov 2005 St. Louis, Missouri) – ASPT Newsletter 19(2): 5. 2005; Rodriguesia 56(88): [v-vii].2005 [2006].

Ziesenhenne, Rudolf Christian (Chicago, Illinois 15 Feb 1911- 19 Oct 2005, Los Angles, California) – American Begonia Society website.

## Appendix B

**Institutional votes**

In early 2005, the Bureau of Nomenclature, after consultation with a number of botanists with particular knowledge and interest, reviewed the list of institutional votes allocated for the XVI International Botanical Congress in St Louis and recommended to the General Committee that four institutions in China that were not included in the St Louis list be allocated a vote and that the allocation be adjusted for 10 other institutions, 7 of which were in China. The General Committee accepted the Bureau’s recommendation (cf Div. III.4(b)(2)).

In reviewing the list of institutional votes allocated at St Louis, the Bureau took note of the fact that there was an extensive review of Institutional Votes prior to the Tokyo Congress in 1993 which saw a major shift toward better representation from developing countries and from China and the former Soviet Union, resulting in a 22% increase in votes from 671 to 819. For the St. Louis Congress the votes of only 53 herbaria changed, 10 votes being lost and 45 being gained, including 33 institutions that had not previously been allocated a vote. The present Bureau recognised that for some parts of the world, notably China, there had been a lack of detailed knowledge at the time of the pre-Tokyo review and the voting changes proposed for Vienna were primarily to remedy this.

The following list includes all institutions that were entitled to vote at the Vienna Congress, The four institutions that were added to the list since the St Louis Congress are preceded by a “#” symbol. An asterisk (*) denotes the institutions the votes of which were exercised by delegates or vice-delegates at the Nomenclature Section in Vienna. The total number of institutions allocated votes for the Vienna Congress was 501 with an allocation of 866 votes in all. Of these, 171 institutions, bearing 404 votes, were represented in Vienna.

Institutions are identified by the name of the city in which each is located, followed by the standard herbarium code used in *Index herbariorum* (http://sweetgum.nybg.org/ih/). Where the name of the city has changed from that given in Appendix B of the Report on the Nomenclature Section in St Louis (Englera 20: 247–253. 2000), this is indicated by provision of cross-references. The number of votes allotted to each institution is given in the right-hand column. The “+” symbol is used when two or more herbaria are treated together as a single institution in the wide sense of the term (Div. III.4(b)(2) (second sentence)), e.g., A + FH + GH are treated as a single institution with six votes.

**Table:** List of institutions

*	Aarhus (AAU)	4
	Aberdeen (ABD)	1
	Abidjan (UCJ)	1
*	Addis Ababa (ETH)	1
*	Adelaide (AD)	4
	Akureyri (AMNH)	1
	Albany (NYS)	1
	Albuquerque (UNM)	1
	Allahabad (BSA)	1
	Alma-Ata (AA)	1
	Ames (ISC)	2
	Amherst (MASS)	1
	Amsterdam (AMD)	1
	Ankara (ANK)	1
*	Ann Arbor (MICH)	4
	Antananarivo (TAN)	1
	Aquila (AQUI)	1
	Arcata (HSC)	1
	Ashkhabad (ASH)	1
	Asuncion (FCQ)	1
	Athens, Greece (ATHU)	2
	Athens, USA (GA)	
*	Auckland (AK)	1
*	Auckland (AKU)	1
	Auckland (PDD)	1
*	Austin (TEX)	4
	[Baarn (CBS), see Utrecht]	
	Baghdad (BAG)	1
	Baku (BAK)	1
	Bangi (UKMB)	1
	Bangkok (BKF)	2
*	Barcelona (BC)	2
*	Barcelona (BCC)	1
*	Barcelona (BCF)	2
	Bari (BI)	1
	Basel (BAS)	1
	Baton Rouge (LSUM + LSU)	2
	Beijing (CAF)	1
	Beijing (CMMI)	1
	Beijing (HMAS)	5
	Beijing (PE)	7
	Belem (IAN)	1
	Belem (MG)	1
*	Beltsville (BARC)	1
*	Beltsville (BPI)	3
*	Beograd (BEO)	1
*	Bergen (BG)	2
*	Berkeley (UC)	5
*	Berlin (B)	7
	Berlin (BHUPM)	1
	Bern (BERN)	1
	Birmingham (BIRM)	1
	Blacksburg (VPI)	1
	Bloomington (IND)	1
	Bogor (BO)	4
	Bogota (COL)	3
	Bologna (BOLO)	1
	[Bombay (BLAT), see Mumbai]	
	Bonn (BONN)	1
	Boulder (COLO)	1
	Brasilia (IBGE)	1
*	Brasilia (UB)	1
*	Bratislava (BRA)	1
*	Bratislava (SAV)	1
	Bratislava (SLO)	2
*	Brisbane (BRI)	4
*	Brno (BRNM)	1
*	Brno (BRNU)	1
	[Bronx (NY), see New York]	
*	Brooklyn (BKL)	1
	Bruxelles (BRVU)	1
	Bucuresti (BUCA + BUCM)	2
*	Budapest (BP)	5
*	Buenos Aires (BA)	3
*	Buenos Aires (BAA)	1
*	Buenos Aires (BAF)	2
*	Buenos Aires (BAFC)	1
	Buffalo (BUF)	1
*	Burdwan (BURD)	1
	Burlington (VT)	1
	Cairo (CAI)	2
	Cairo (CAIM)	1
	[Calcutta (CAL), see Kolkata]	
	Calgary (UAC)	1
	Cambridge, UK (AAS)	1
*	Cambridge, UK (CGE)	1
*	Cambridge, UK (CGG)	1
*	Cambridge, USA (A + FH + GH)	6
	Camerino (CAME)	1
	Campinas (UEC)	2
*	Canberra (CANB)	4
*	Canberra (CBG)	3
	Cape Town (BOL)	2
*	Cape Town (NBG)	2
	Caracas (VEN)	2
*	Carbondale (SIU)	1
	Cardiff (NMW)	1
*	Castelar (BAB)	1
	Catania (CAT)	1
	Cayenne (CAY)	1
	Champaign (ILLS)	1
	Changsha (HNNU)	1
	Chapel Hill (NCU)	2
	Chengdu (CDBI)	2
	Chengdu (SZ)	1
*	Chicago (F)	5
	Chongqing (HWA)	1
	Chongqing (SM)	1
*	Christchurch (CANU)	1
*	Christchurch (CHR)	3
*	Claremont (RSA)	2
*	Clermont-Ferrand (CLF)	1
	Cluj-Napoca (CL)	1
	Coimbatore (MH)	2
	Coimbra (COI)	3
*	College Park (MARY)	1
	College Station (TAES)	1
*	Columbus (OS)	2
	[Como (PERTH), see Perth]	
	Concepcion (CONC)	1
*	Copenhagen (C)	7
*	Cordoba (CORD)	2
	Corrientes (CTES)	1
	Corvallis (OSC)	1
	Curitiba (MBM)	1
	[Dacca (DACB), see Dhaka]	
*	Darwin (DNA)	1
	Davis (DAV)	1
	Dehra Dun (DD)	2
	Delaware (OWU)	1
	Dhaka (DACB)	1
	Dublin (DBN)	1
*	Dublin (TCD)	1
*	Durham (DUKE)	2
	Dushanbe (TAD)	1
	East Lansing (MSC)	2
*	Edinburgh (E)	6
	Edmonton (ALTA)	1
	Edmonton (UAMH)	1
*	Egham (IMI)	4
	Fairbanks (ALA)	1
*	Fairfax (GMUF)	1
*	Firenze (FI + FT)	4
	Fort Worth (BRIT)	3
	Frankfurt (FR)	1
	Fuzhou (FNU)	1
	Gainesville (FLAS)	2
	Gatersleben (GAT)	2
*	Geneve (G)	7
	Gent (GENT)	1
	Georgetown (BRG)	1
	Giessen (GI)	1
	Goteborg (GB)	4
	Gottingen (GOET)	1
	Grahamstown (GRA)	1
	Granada (GDA)	1
	Graz (GJO)	1
*	Graz (GZU)	2
	Guadalajara (GUADA)	1
	Guangzhou (IBSC)	4
	Guangzhou (SYS)	1
	Guelph (OAC)	1
	Guilin (IBK)	2
	Guiyang (HGAS)	1
*	Habana (HAC)	2
*	Habana (HAJB)	3
	Halle (HAL)	2
	Hamburg (HBG)	3
	Hangzhou (HZU)	1
*	Hanoi (HN)	2
*	Hanoi (HNU)	1
*	Harare (SRGH)	2
	Harbin (NEFI)	1
*	Helsinki (H)	7
	Hiroshima (HIRO)	2
*	Hobart (HO)	1
#	Hong Kong (CUHK)	1
	Honolulu (BISH)	2
	Iasi (I)	1
	Ibadan (FHI)	1
	[Indooroopilli (BRI) see Brisbane]	
	Innsbruck (IB)	1
	Islamabad (ISL)	1
	Islamabad (RAW)	1
*	Istanbul (ISTE)	1
*	Istanbul (ISTF)	1
*	Ithaca (BH)	3
	Ithaca (CUP)	1
	Izmir (EGE)	1
	Jaca (JACA)	1
	Jaipur (RUBL)	1
	Jena (JE)	3
	Jerusalem (HUJ)	2
	Johannesburg (J)	1
*	Kalaheo (PTBG)	1
*	Kampala (MHU)	1
	Kanazawa (KANA)	1
*	Karachi (KUH)	2
	Karlsruhe (KR)	1
	Kathmandu (KATH)	1
*	Kew (K)	7
	Kiev (KW)	5
	Kingston (IJ)	1
	Kishinev (CHIS)	1
	Knoxville (TENN)	1
	Kolkata (CAL)	5
	Kornik (KOR)	1
	Krakow (KRA)	2
	Krakow (KRAM)	3
*	Kuala Lumpur (KEP)	1
*	Kuala Lumpur (KLU)	1
	Kuching (SAR)	1
	Kunming (KUN)	5
#	Kunming (PYU)	1
	Kunming (SWFC)	1
#	Kunming (YUKU)	1
*	Kyoto (KYO)	2
	La Laguna (TFC)	1
	La Paz (LPB)	1
	La Plata (LP)	1
	La Plata (LPS)	1
	Lae (LAE)	1
	Lafayette (LAF)	1
	Lanzhou (NWTC)	1
	Laramie (RM)	1
	Lausanne (LAU)	1
	[Lawai (PTBG), see Kalaheo]	
	Lawrence (KANU)	1
*	Leeds (LDS)	1
	Legon (GC)	1
	Leicester (LTR)	1
*	Leiden (L)	7
	Leipzig (LZ)	1
	[Leningrad (LE, LECB, WIR), see Saint Petersburg]	
*	Liege (LG)	2
	Lima (USM)	2
	Linz (LI)	1
*	Lisboa (LISC)	2
	Lisboa (LISI)	1
*	Lisboa (LISU)	2
	Lisle (MOR)	1
*	Liverpool (LIV)	2
	Ljubljana (LJU)	1
	Lodz (LOD)	1
	Logan (UTC)	1
	London, Canada (UWO)	1
*	London, UK (BM)	7
	Los Angeles (LA)	1
	Los Angeles (LAM)	1
	Los Banos (CAHUP)	1
	Louvain-la-Neuve (MUCL)	1
*	Lublin (LBL + LBLC)	1
	Lucknow (LWG)	2
	Lund (LD)	6
	Lushan (LBG)	1
	Luxembourg (LUX)	1
	Lviv (LW)	1
	Madison (CFMR)	1
*	Madison (WIS)	2
*	Madrid (MA)	4
*	Madrid (MAF)	1
	Magadan (MAG)	1
	Mainz (MJG)	1
	Malaga (MGC)	1
	Manassas (ATCC)	1
	Manaus (INPA)	1
	Manchester (MANCH)	1
	Mangilao (GUAM)	1
	Manhattan (KSC)	1
	Manila (PNH)	1
*	Maputo (LMA)	1
*	Maracay (MY)	1
	Marseille (MARSSJ)	1
	Matsumoto (SHIN)	1
*	Mayaguez (MSM)	1
	Medellin (HUA)	1
*	Meise (BR)	6
*	Melbourne (MEL)	4
*	Melbourne (MELU)	1
*	Merida (CICY)	1
	Mesa de Cavacas (PORT)	1
*	Mexico City (ENCB)	3
*	Mexico City (FCME)	1
*	Mexico City (MEXU)	5
*	Mexico City (UAMIZ)	1
*	Miami (FTG)	2
	Milwaukee (MIL)	1
	Minsk (MSK)	1
	Mississippi State (IBE)	1
	Missoula (MONTU)	1
	Montecillo (CHAPA)	1
	Monterey (UNL)	1
	Montevideo (MVM)	1
*	Montpellier (MPU)	2
*	Montreal (MT)	2
	Montreal (MTMG)	1
	Morehead City (IMS)	1
*	Morgantown (WVA)	1
	Moscow, Russia (MHA)	2
	Moscow, Russia (MW)	2
*	Moscow, USA (ID)	1
	Mumbai (BLAT)	1
*	Munchen (M)	5
*	Munchen (MSB)	1
	Murcia (MUB)	1
*	Nairobi (EA)	2
	Nairobi (NAI)	1
	Nanjing (N)	1
	Nanjing (NAS)	2
	Nanjing (NF)	1
*	Napoli (NAP)	1
	Neuchatel (NEU)	1
	New Delhi (HCIO)	1
	New Haven (YU)	1
	New Orleans (NO)	1
*	New York (NY)	7
	Nichinan (NICH)	2
	Norman (OKL)	1
	Novosibirsk (NS)	1
	Novosibirsk (NSK)	1
	Oeiras (LISE)	1
	Orange (DAR)	1
*	Oslo (O)	5
*	Ottawa (CAN + CANA +	
	CANL + CANM)	5
*	Ottawa (DAO)	3
*	Ottawa (DAOM)	3
	Oulu (OULU)	1
	Oviedo (FCO)	1
*	Oxford, UK (FHO)	
*	Oxford, UK (OXF)	
*	Oxford, USA (MU)	
	Padova (PAD)	2
*	Palermo (PAL)	3
*	Panama (PMA)	1
*	Paris (P)	4
*	Paris (PC)	2
	Patras (UPA)	2
*	Patzcuaro (IEB)	1
	Pavia (PAV)	2
	Peoria (NRRL)	2
	Peradeniya (PDA)	1
*	Perth (PERTH)	3
	Perugia (PERU)	1
	Philadelphia (ANSP)	1
*	Philadelphia (PH)	2
*	Pisa (PI)	2
*	Pittsburgh (CM)	2
	Plovdiv (SOA)	1
	Porto (PO)	1
	Porto Alegre (HAS)	1
*	Porto Alegre (ICN)	1
	Potchefstroom (PUC)	1
*	Poznan (POZ + POZG)	2
	Praha (PR + PRM)	2
*	Praha (PRC)	3
*	Pretoria (PRE)	4
*	Pretoria (PREM)	2
*	Pretoria (PRU)	1
	Provo (BRY)	1
	Pullman (WS)	1
	Pullman (WSP)	1
	Pune (AHMA + AMH)	1
	Pune (BSI)	1
	Quebec (QFA)	1
*	Quito (QCA)	1
	Quito (QCNE)	1
	Rabat (RAB)	1
*	Raleigh (NCSC)	1
	Reading (RNG)	2
	Recife (URM)	1
	Regensburg (REG)	2
	Reykjavik (ICEL)	1
	Riga (RIG)	1
	Rio de Janeiro (HB)	1
*	Rio de Janeiro (R)	2
	Rio de Janeiro (RB)	5
	Riverside (UCR)	1
	[Rockville (ATCC), see Manassas]	
	Roma (RO)	1
	[Rydalmere (DAR), see Orange]	
*	Saint Andrews (STA)	1
*	Saint Augustine (TRIN)	1
*	Saint Louis (MO)	7
	Saint Paul (MIN)	2
	Saint Petersburg (LE)	7
	Saint Petersburg (LECB)	1
	Saint Petersburg (WIR)	1
	Salaspils (LATV)	1
	Salt Lake City (UT)	1
	Saltillo (ANSM)	1
	San Diego (SD)	1
	San Francisco (CAS, DS)	4
	San Francisco (SFSU)	1
*	San Isidro (SI)	3
*	San Jose (CR)	1
	[San Lorenzo (FCQ), see Asuncion]	
	San Miguel	
	Tucuman (LIL)	3
	Sandakan (SAN)	1
	Santa Barbara (SBBG)	1
*	Santiago (SGO)	1
*	Santo Domingo (INB)	1
*	Santo Domingo (JBSD)	1
*	Sao Leoopoldo (PACA)	1
*	Sao Paulo (SP)	4
*	Sao Paulo (SPF)	2
	Sapporo (SAP)	1
	Sarajevo (SARA)	1
	Saskatoon (SASK)	1
*	Sassari (SS)	1
	Seattle (WTU)	2
	Sendai (TUS)	1
	Seoul (SNU)	2
	Sevilla (SEV)	2
#	Shanghai (FUS)	1
*	Shanghai (HSNU)	1
	Shanghai (SHM)	1
	Shenyang (IFP)	2
	Shillong (ASSAM)	1
	Singapore (SING)	2
	Sofia (SOM)	3
	Stellenbosch (STE)	1
	Stevens Point (UWSP)	1
	Stockholm (S)	6
	Stockholm (SBT)	1
	Stockholm (SUNIV)	2
	Storrs (CONN)	1
	Stuttgart (STU)	2
	Suva (SUVA)	1
	[Sverdlovsk (SVER), see Yekaterinburg]	
*	Sydney (NSW)	6
	Taipei (HAST)	1
	Taipei (NTUF)	1
*	Taipei (TAI)	1
	Taipei (TAIF)	1
	Tallahassee (FSU)	1
	Tampa (USF)	1
	Tartu (TAA)	2
*	Tartu (TU)	2
	Tashkent (TASH)	2
	Tbilisi (TBI)	2
*	Tegucigalpa (EAP)	1
	Tehran (IRAN)	2
*	Tehran (TARI)	1
	Tel Aviv (TELA)	1
*	Tempe (ASU)	1
	Thessaloniki (TAU)	1
	Tiruchirapalli (RHT)	1
	Tokyo (MAK)	2
*	Tokyo (TI)	4
*	Tokyo (TNS)	5
	Tomsk (TK)	1
	Torino (TO)	1
*	Toronto (TRT)	1
	Toronto (TRTC)	1
	Toru_ (TRN)	1
	Tottori (TMI)	1
	Toulouse (TL)	1
	Trieste (TSB)	2
	Tripoli (ULT)	1
	Trondheim (TRH)	2
	Trujillo (HUT)	1
	Tsukuba (TKB)	1
	Tubingen (TUB)	1
*	Tucson (ARIZ)	1
	Turku (TUR)	2
	Ulan Bator (UBA)	1
	Umea (UME)	2
	University Park (PAC)	1
*	Uppsala (UPS)	4
	Uppsala (UPSV)	1
	Urbana (ILL)	1
	Urumqi (XJA)	1
	Urumqi (XJBI)	1
	Urumqi (XJU)	1
*	Utrecht (CBS)	4
*	Utrecht (U)	3
	Vacratot (VBI)	1
	Valencia (VF)	1
	Vancouver (UBC)	2
	Victoria (DAVFP)	1
*	Victoria (V)	1
	Vilnius (BILAS)	1
	Vladivostok (VLA)	1
*	Wageningen (WAG)	4
	Warszawa (WA)	3
	Washington (NA)	1
*	Washington (US)	7
*	Waterloo (WAT)	1
*	Wellington (WELT)	1
*	Wellington (WELTU)	1
*	Wien (W)	3
*	Wien (WU)	5
*	Windhoek (WIND)	1
*	Wisley (WSY)	1
*	Wolfville (ACAD)	1
*	Wroclaw (WRSL)	1
	Wuhan (CCNU)	1
	Wuhan (HBI)	1
	Wuhan (HIB)	1
	Wuhan (WH)	1
*	Xalapa (XAL)	2
	Xining (HNWP)	2
#	Xishuangbanna (HITBG)	1
	Yangling (WUK)	2
*	Yaounde (YA)	1
	Yekaterinburg (SVER)	1
	Yerevan (ERE)	3
	Zagreb (ZA)	1
*	Zapopan (IBUG)	2
	Zomba (MAL)	1
	Zurich (Z)	3
	Zurich (ZT)	1

## Appendix C

List of registered members of the Nomenclature Section

This list is also an index to speakers as recorded in the preceding report, with page references to all relevant entries.

Abrol, Sanjeev Kumar (India)

Ahti, Teuvo (Finland)... 42, 147, 183, 186, 188, 206, 218, 223, 233, 244, 249, 250

Akhundzadeh, Najibeh (Iran)

Alford, Mac (U.S.A.)... 99, 116, 163, 193, 210

Amuh, Samuel (Ghana)

Antwi, Philip (Ghana)

Arendse, Sumaya (South Africa)

Arnold, Trevor (South Africa)

Atha, Daniel (U.S.A.)... 66, 102, 103, 105, 109, 138, 169, 178, 182, 185, 187, 194, 200, 250, 286, 294

Atiemo, Samuel (Ghana)

Barker, Christine (U.K.)

Barkworth, Mary E. (U.S.A.)... 94, 112

Barrie, Fred R. (U.S.A.)... 3, 13, 22, 25, 27, 29, 35, 36, 39-42, 44, 45, 48, 56, 57, 59, 60, 77, 80, 88, 117, 119, 136, 141, 142, 149, 152, 181, 183, 192, 195, 196, 199, 200, 224, 226, 281, 284, 287, 289, 308-309, 316

Baum, Bernard (Canada)... 96

Baxter, Lynne (New Zealand)

Belem, Mamounata (Burkina Faso)

Bell, Hester (U.S.A.)

Bellu, Francesco (Italy)

Bita-Nicolae, Claudia (Romania)

Bogdanovic, Sandro (Croatia)

Bolourian Kashy, Mohammadreza (Iran)

Bonde, Suresh (India)

Breitwieser, Ilse (New Zealand)

Briggs, Barbara (Australia)... 100, 114

Brummitt, Dick K. (U.K.)... 14, 19, 28, 29, 32, 35, 36, 42, 47, 52, 56, 57, 74, 77, 78, 79, 82, 108, 110, 112, 115-117, 119, 124, 126-131, 134-136, 138, 141, 143, 144, 148-156, 166-168, 180-183, 186, 199, 201, 216, 217, 231-234, 243, 245, 268, 273, 286, 287, 290, 292, 307, 313

Buck, Bill R. (U.S.A.)... 16, 20, 39, 48, 49, 61, 98, 104, 118, 144, 173, 181, 183, 186, 196, 197, 219, 225, 226, 236, 237, 245, 301

Burdet, Herve (Switzerland)

Cafferty, Steve (U.K.)

Challis, Katherine (U.K.)... 121, 156, 157, 183, 208

Chaloner, Bill (U.K.)... 18, 97, 112, 139, 300-304

Chapman, Alex R. (Australia)

Chesselet, Pascale (South Africa)

Christenhusz, Maarten (Finland)

Correia, Ana Isabel (Portugal)

Crandall-Stotler, Barbara (U.S.A.)

David, John (U.K.)... 224, 244, 275-288, 294

Davidse, Gerrit (U.S.A.)... 11, 41, 87, 95, 136, 141, 186, 281, 283, 284

Delwiche, Charles (U.S.A.)... 189, 225

Demoulin, Vincent (Belgium)... 7, 23, 24, 26, 29, 31-33, 40, 41, 46, 48, 50, 51, 54, 55, 59-61, 64, 67, 73, 75, 78, 81, 83-85, 91, 98, 102-104, 111, 115, 118, 120-122, 130, 132, 133, 135, 136, 138, 141, 142, 144, 148, 152-154, 164, 170, 174, 183, 190, 191, 194-196, 198, 204, 207, 209, 211, 213, 215, 220-223, 228, 230, 234-236, 238, 239, 241, 243, 245, 248, 249, 252, 255, 257-260, 262, 263, 265-267, 269-272, 274, 276-277, 280, 282, 286, 288, 293, 298, 301-303, 305

Dhabe, Arvind (India)

Dixon, Chris (Austria)

Domina, Gianniantonio (Italy)... 282

Dominic, Mary (Nigeria)

Dorr, Laurence (U.S.A.)... 10, 12, 21, 22, 77, 91, 92, 95, 97, 106, 114, 116, 131, 132, 140, 171, 190, 195, 197, 201, 204, 209, 248

Dressler, Stefan (Germany)

Eckenwalder, James (Canada)... 11, 103, 116, 281, 294

Emadzade, Khatere (Iran)

Enayatmehr, Nasrin (Iran)

Fischer, Markus (Germany)

Flann, Christina (Netherlands)

Flores, Hilda (Mexico)

Flores, Miguel (Guatemala)

Ford, Kerry (New Zealand)

Ford-Werntz, Donna (U.S.A.)... 16, 281

Fortunato, Renee (Argentina)... 312

Freire-Fierro, Alina (U.S.A.)... 111, 171, 189, 200, 278, 281

Friis, Else Marie (Sweden)... 122

Friis, Ib (Denmark)

Fu, Dai-Li (China)

Funk, Vicki A. (U.S.A.)... 13, 16, 106, 109, 193, 202, 208, 248, 261, 262, 283

Gandhi, Kanchi (U.S.A.)... 12, 29, 30, 34, 35, 37, 39, 40, 42, 53, 61, 66, 68, 69, 88, 99, 100, 107, 111, 121, 129, 137, 139, 149, 165, 172, 175, 180, 184, 185, 187, 193, 200, 202, 206, 208, 215-217, 226, 228, 229, 233, 239, 243, 245, 270, 280, 290, 303

Gao, Tiangang (China)

Garnock-Jones, Phil (New Zealand)... 187

Gereau, Roy (U.S.A.)... 23, 36, 54, 63, 81, 83, 129, 130, 133, 173, 182, 201, 226, 228, 249, 252, 257, 260, 305

Ghahremaninejad, Farrokh (Iran)

Ghanavati, Farangis (Iran)

Gillespie, Lynn (Canada)

Godin, Christophe (France)

Govaerts, Raphael (U.K.)... 180, 288, 289, 295

Grether, Rosaura (Mexico)

Gucel, Salih (Cyprus)

Gutierrez, Raul (U.S.A.)

Haston, Elspeth (U.K.)... 184, 189

Hawksworth, David L. (Spain)... 3, 21, 41, 52, 53, 55, 99, 101, 103, 105, 147, 185, 196, 198, 205, 206, 212, 222, 234, 237-240, 302, 305, 307, 318

Henderson, Rod (Australia)

Henry, Teke Mosinga (Cameroon)

Hoch, Peter (U.S.A.)

Hoef-Emden, Kerstin (Germany)

Hoffmann, Lucien (Luxembourg)... 50, 172, 221

Hoffmann, Petra (U.K.)... 27, 76, 85, 87, 109, 110, 136, 146, 162, 192, 204, 209, 210, 214, 237, 238, 264, 295

Hollowell, Victoria (U.S.A.)... 111, 279

Holmgren, Noel (U.S.A.)

Holmgren, Patricia (U.S.A.)... 280, 283

Homchuen, Samang (Thailand)

Horandl, Elvira (Austria)

Hristovski, Nikola (Former Yugoslav Republic of Macedonia)

Ignatov, Michael (Russia)... 122

Irving, Rosie (U.K.)

Jamzad, Ziba (Iran)

Jarvis, Charlie (U.K.)... 176

Jin, Hong (China)

Jorgensen, Per Magnus (Norway)... 14-16, 26, 69, 79, 86, 90, 122, 147, 171, 174, 176, 184, 189, 210, 238, 280

Jorgensen, Peter Moller (U.S.A.)... 174, 210, 258, 294

Kallunki, Jacquelyn (U.S.A.)

Klopper, Ronell (South Africa)

Knapp, Sandra (U.K.)... 95, 101, 102, 129, 200, 255, 274

Koekemoer, Marinda (South Africa)

Kolterman, Duane (Puerto Rico)... 61, 88, 111, 137, 158, 160, 191, 208, 225, 249, 251, 259, 276, 289, 295

Kool, Anneleen (Sweden)

Kovtun, Irina (Ukraine)

Krivka, Pavel (Czech Republic)

Lack, H. Walter (Germany)... 104, 106, 107, 121, 124, 216, 220

Landrum, Leslie (U.S.A.)... 55, 114, 138, 164, 198, 203, 278, 282

Linder, Peter (Switzerland)... 318

Liu, Ho-Yih (Taiwan)

Liu, Maochun (China)

Lo, Eugenia (Canada)

Logosu, Derrick (Ghana)

Loizeau, Pierre-Andre (Switzerland)

Lorence, David (U.S.A.)

Mabberley, David (U.S.A.)... 14, 15, 47, 48, 75, 103, 114, 121, 202, 204, 272, 288, 318

Malecot, Valery (France)

Marhold, Karol (Slovakia)... 40, 67, 91, 139, 143, 156, 172, 175, 185, 188, 202, 219, 281, 284, 285

Mayer, Veronika (Austria)

Mazzola, Pietro (Italy)

McNeill, John (U.K.)... 6-28, 30-33, 35-53, 55-69, 72-74, 76-89, 91-103, 105-126, 128-158, 160-241, 243-246, 248-290, 292-309, 316, 319

Mimba, Kkini Alice (Cameroon)

Mitic, Bozena (Croatia)

Moore, Gerry (U.S.A.)... 19, 24, 43, 58, 62-67, 69, 84, 88, 128, 158, 160-162, 166, 180, 227, 232, 290, 293

Morales Can, Julio (Guatemala)

Muellner, Alexandra Nora (U.K.)

Mukhtar, Fatima Batul (Nigeria)

Munawar, Muhammad (South Korea)

Murphy, Dan (Australia)

Musienko, Mykola (Ukraine)

Nagamasu, Hidetoshi (Japan)

Nee, Michael (U.S.A.)... 86, 95, 102, 117, 165, 176, 187, 188, 219, 243, 244

Nic Lughadha, Eimear (U.K.)... 11, 26, 38, 39, 41, 53, 81, 95, 104, 112, 115, 120, 123, 125, 131, 144, 145, 163, 168, 173, 175, 181, 183, 185-187, 199, 201, 209, 250, 254, 255, 282, 285, 289, 298

Nicolson, Dan H. (U.S.A.)... 6-7, 10-12, 14, 15, 17, 18, 20, 23, 26, 27, 29, 30, 32, 33, 37-40, 43-44, 46, 49, 55-57, 59, 61, 63-65, 67, 69-72, 79-81, 83, 85, 86, 88, 90-92, 95, 96, 101-103, 106, 107, 109, 111, 120, 123, 124, 131, 133-135, 137, 142, 146, 147, 149, 151, 152, 155, 157, 158, 160, 165, 168, 171, 174, 176-180, 184-205, 207, 209-211, 215, 217, 220, 223, 224, 229, 230, 233-236, 243-248, 250-252, 254-257, 259-275, 278, 280, 282, 285-289, 291, 293-297, 299, 300, 303, 305, 319, 320

Norvell, Lorelei L (U.S.A.)... 95, 104, 107, 203

Oliver Osagie, Iguare (Nigeria)

Omorogbe, Victory (Nigeria)

Omoseyindemi, Olubunmi (Nigeria)

Orchard, Tony (Australia)... 85, 107, 113, 116, 125, 226, 264, 311, 315

Pabon Mora, Natalia (Colombia)

Park, J-M (Austria)

Paun, Ovidiu (Austria)... 189

Pearson, Hugh (U.K.)

Pedley, Les (Australia)... 54, 128, 172, 310, 318

Pedroche, Francisco (Mexico)

Peng, Ching-I (Taiwan)... 106, 184, 290

Pereira, Jorge (Brazil)... 15, 119, 285

Perry, Gillian (Australia)... 129-129, 133-135, 139, 140, 142, 146, 244

Peterson, Paul (U.S.A.)

Pokle, Dileep (India)... 278

Prado, Jefferson (Brazil)... 123

Prance, Ghillean (U.K.)... 171, 181, 248, 302

Prasad, A. K. S. K. (U.S.A.)

Prince, Linda (U.S.A.)

Printzen, Christian (Germany)... 81, 138, 157, 205, 265

Prockow, Jaroslaw (Poland)

Rampho, Tebogo (South Africa)

Ramsankar, Basu (India)

Ranjbar, Massoud (Iran)

Rebernig, Carolin (Austria)

Redhead, Scott A. (Canada)... 20, 25, 41, 94, 116, 137, 144, 145, 155, 161, 162, 170, 172, 178, 181, 182, 184-188, 190, 192, 193, 195-197, 211, 212, 224, 236-240, 261, 302

Reich, Dieter (Austria)

Reier, Ulle (Estonia)

Ricciuti Lamonea, Alessandra (Austria)

Rico Arce, Maria De Lourdes (U.K.)... 14, 279, 317

Rijckevorsel, Paul van (Netherlands)... 11, 13, 26, 43, 52, 56, 62, 64, 69-73, 75-78, 80, 82, 83, 94, 96, 101, 126, 132, 143, 194, 213, 214, 228, 231, 246, 254, 257, 258, 262-265, 268, 269, 272, 290, 295, 308

Romanov, Mikhail (Russia)

Roux, J.P. (South Africa)

Sanchez-Ken, Gabriel (U.K.)

Schafer, Peter A. (France)... 91, 128, 146, 165, 216, 218, 225, 280

Schanzer, Ivan (Russia)... 22, 171

Schrire, Brian (U.K.)... 309

Silva, Paul C. (U.S.A.)... 13, 15, 49, 50, 210, 221, 305, 307

Skog, Judith (U.S.A.)... 17, 31-33, 44-46, 90, 181, 182, 221, 287, 307

Smith, Gideon F. (South Africa)... 174, 316

Somlyay, Lajos (Hungary)

Soreng, Robert (U.S.A.)

Sosef, Marc S.M. (Netherlands)

Stainton, Marion (U.K.)

Starlinger, Franz (Austria)

Stotler, Raymond E. (U.S.A.)

Stuessy, Tod (Austria)... 5, 7, 13-15, 18, 72, 105, 113, 117-119, 160, 253, 280, 288, 319

Talent, Nadia (Canada)

Tan, Benito (Singapore)... 42, 185, 300

Taylor, Charlotte (U.S.A.)... 99, 157, 263, 272, 278

Taylor, Nigel (U.K.)... 157, 168-171, 205, 262

Tidke, Jaykiran (India)

Till, Walter (Austria)

Toolin, Laurence (U.S.A.)

Tran, Thi Hoa (Vietnam)

Tronchet, Frederic (France)... 42, 119, 125, 205, 282

Tsang, Che Wah Anita (Hong Kong)

Turland, Nicholas (U.S.A.)... 16, 18, 20-30, 39, 41, 43, 44, 49, 58-61, 63, 65-69, 109, 113, 123, 150, 165, 179, 186, 202, 203, 206, 207, 218, 219, 221, 224, 225, 227, 250, 261, 265

Vitek, Ernst (Austria)

Voglmayr, Hermann (Austria)

Wagner, Warren (U.S.A.)

Ward, Jo (New Zealand)

Watson, Mark (U.K.)... 19, 20, 23, 25, 31, 39, 41, 48, 86, 88, 95, 100, 129, 156, 157, 177, 196, 197, 208, 241, 249, 251, 281, 299

West, Judy (Australia)... 12, 113, 186, 254

Wieringa, Jan (Netherlands)... 16, 23, 27, 36, 41, 61, 63, 75, 79, 85-89, 102, 104, 106, 116, 120, 124, 130, 131, 161, 162, 166, 174, 180, 181, 185, 191, 193, 197-199, 201-203, 207, 214, 219, 229, 239, 250, 256, 257, 261, 262

Wiersema, John H. (U.S.A.)... 54, 121, 141, 143, 151, 154, 215, 216, 238, 242, 243, 253, 262, 269, 286, 289

Willner, Wolfgang (Austria)

Wilson, Karen L. (Australia)... 24, 27, 38, 48, 60, 80, 82, 86, 89, 93-107, 114, 115, 117, 140, 151, 157, 179, 218, 249, 268, 288-290

Wilson, Peter (Australia)... 31, 65, 107, 132, 217, 258, 265, 269, 295

Woodland, Dennis (U.S.A.)... 25, 56, 110, 115, 116, 280

Zhu, Guanghua (U.S.A.)... 7, 104, 192, 303

Zijlstra, Gea (Netherlands)... 13, 24, 33, 40, 45, 48, 49, 73, 74, 80-82, 105, 116, 119, 128, 129, 134, 149-152, 154, 157, 164, 171, 177, 209, 215, 216, 222, 226, 230, 233, 255-257, 259-262, 264, 266, 274, 290, 301, 306

